# Expanding the Structural Diversity at the Phenylene Core of Ligands for the von Hippel–Lindau E3 Ubiquitin Ligase: Development of Highly Potent Hypoxia-Inducible Factor-1α Stabilizers

**DOI:** 10.1021/acs.jmedchem.3c00434

**Published:** 2023-09-14

**Authors:** Lan Phuong Vu, Claudia J. Diehl, Ryan Casement, Adam G. Bond, Christian Steinebach, Nika Strašek, Aleša Bricelj, Andrej Perdih, Gregor Schnakenburg, Izidor Sosič, Alessio Ciulli, Michael Gütschow

**Affiliations:** † Pharmaceutical Institute, Pharmaceutical & Medicinal Chemistry, University of Bonn, An der Immenburg 4, D-53121 Bonn, Germany; ‡ Centre for Targeted Protein Degradation, School of Life Sciences, University of Dundee, 1 James Lindsay Place, Dundee, Scotland DD1 5JJ, U.K.; § Faculty of Pharmacy, University of Ljubljana, Aškerčeva 7, SI-1000 Ljubljana, Slovenia; ∥ National Institute of Chemistry, Hajdrihova 19, SI-1000 Ljubljana, Slovenia; ⊥ Institute of Inorganic Chemistry, University of Bonn, Gerhard-Domagk-Straße 1, D-53121 Bonn, Germany

## Abstract

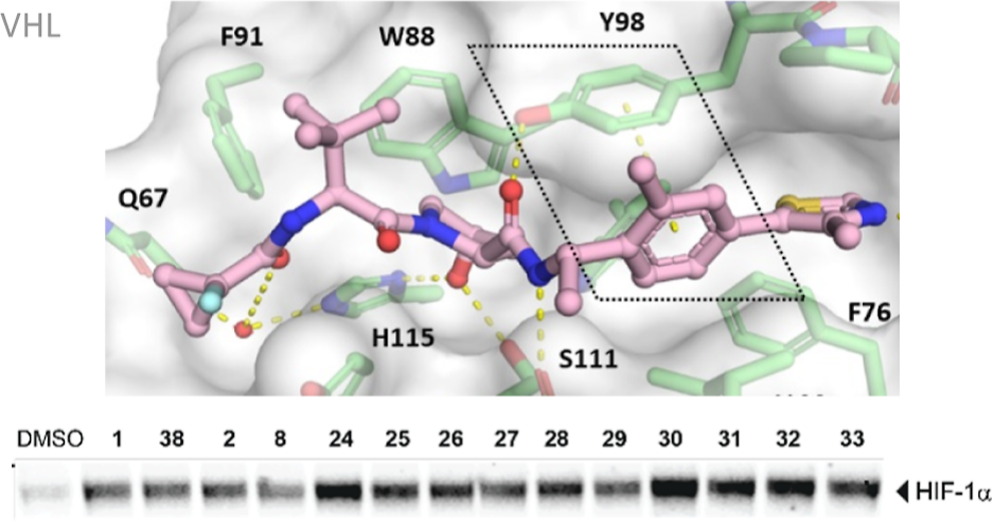

Hypoxia-inducible factor-1α (HIF-1α) constitutes the principal mediator of cellular adaptation to hypoxia in humans. The HIF-1α protein level and activity are tightly regulated by the ubiquitin E3 ligase von Hippel–Lindau (VHL). Here, we performed a structure-guided and bioactivity-driven design of new VHL inhibitors. Our iterative and combinatorial strategy focused on chemical variability at the phenylene unit and encompassed further points of diversity. The exploitation of tailored phenylene fragments and the stereoselective installation of the benzylic methyl group provided potent VHL ligands. Three high-resolution structures of VHL–ligand complexes were determined, and bioactive conformations of these ligands were explored. The most potent inhibitor (**30**) exhibited dissociation constants lower than 40 nM, independently determined by fluorescence polarization and surface plasmon resonance and an enhanced cellular potency, as evidenced by its superior ability to induce HIF-1α transcriptional activity. Our work is anticipated to inspire future efforts toward HIF-1α stabilizers and new ligands for proteolysis-targeting chimera (PROTAC) degraders.

## Introduction

The von Hippel–Lindau (VHL) protein is a tumor suppressor which functions as the substrate recognition component of the multi-subunit Cullin RING E3 ubiquitin ligase complex (CRL2^VHL^). Besides VHL, the CRL2^VHL^ complex includes the central scaffold subunit Cullin 2, the adaptor subunits Elongin B (EloB) and Elongin C (EloC), and RING-box protein 1 (Rbx1).^1−3^ As the largest family of E3 ligases, CRLs are responsible for ∼20% of all ubiquitination events through the ubiquitin–proteasome system (UPS), a cellular machinery implementing the degradation of intracellular protein targets, such as short-lived, damaged, misfolded, and also oxidized proteins.^2,4^ The conjugation of the small protein ubiquitin to the target proceeds via a three-step cascade mechanism. In the first step, the carboxyl group of Gly76 of ubiquitin is ATP dependently attached to a cysteine of a ubiquitin-activating enzyme (E1). Subsequently, the activated ubiquitin is transferred by a transacylation reaction to a cysteine residue of a ubiquitin-conjugating enzyme (E2) and then irreversibly transferred to a lysine residue of a target protein, a key step that is catalyzed by an E3 ligase. The consecutively generated polyubiquitin chain serves as a tag for target recognition and degradation by the 26S proteasome.^5^

Aberrant regulation of CRLs and the UPS pathway is linked to a wide range of human diseases, such as cancer, diabetes, neurodegenerative disorders, and inflammation. The importance of therapeutic interventions is evidenced by the development of proteasome inhibitors, which, however, have several limitations as they lack specificity and may lead to the accumulation of a variety of cellular proteins.^2,6^ However, there are attractive therapeutic targets upstream of the proteasome, in particular, E3 ligases, which endow the UPS system with high specificity. Respective drugs can act by disrupting or modulating the interaction of E3 ligases with their natural substrates.^5^

**Figure 1:**
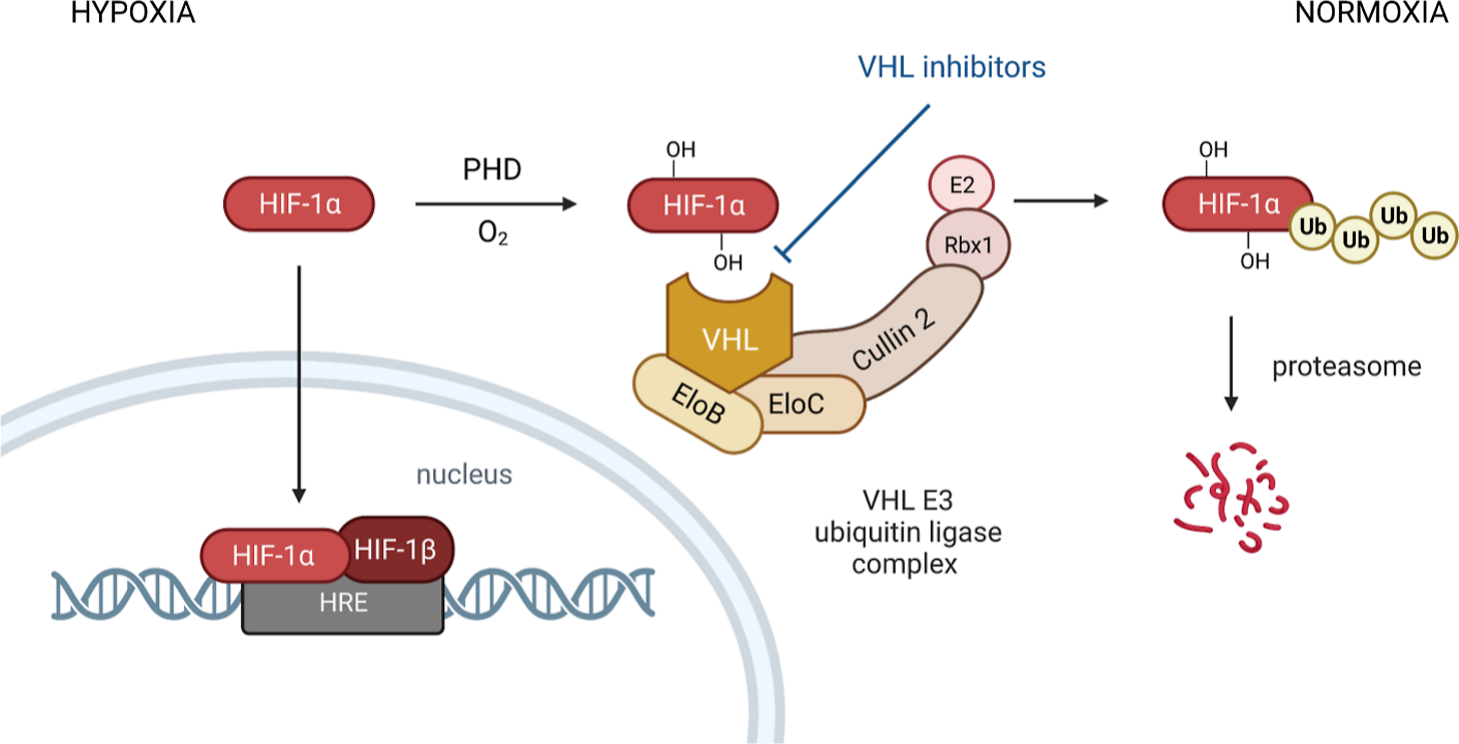
Mechanisms of oxygen-regulated activity of HIF-1α. Under normoxic conditions, PHDs use oxygen to hydroxylate HIF-1α, which is recognized by the CRL2^VHL^ complex, followed by ubiquitination and degradation. Under hypoxic conditions, non-hydroxylated HIF-1α accumulates and dimerizes with HIF-1β to a transcriptionally active complex.

In recent years, the E3 ubiquitin ligase CRL2^VHL^ has attracted enormous attention, in particular, because of its key role in oxygen and hypoxia sensing.^7−9^ One of the most well-characterized substrates of VHL is the hypoxia-inducible factor-1α (HIF-1α). This transcription factor regulates numerous human genes, including those related to the maintenance of oxygen homeostasis. HIFs serve as master regulators of hypoxic signaling. They function as heterodimers consisting of two subunits, the oxygen-dependent HIF-α subunit, of which three isoforms are known in humans (HIF-1α, HIF-2α, and HIF-3α), and the constitutively expressed oxygen-independent HIF-β subunit. Under normoxia, two proline residues (Pro402 and Pro564) of HIF-1α undergo post-translational modification by oxygen- and iron-dependent prolyl hydroxylase domain (PHD) enzymes, members of the EglN family of dioxygenases. HIF-1α hydroxylation triggers molecular recognition by VHL, leading to ubiquitination and subsequent degradation of HIF-1α via the proteasomal pathway.^7,8^ In contrast, under hypoxic conditions, PHDs are inactive and HIF-1α remains unhydroxylated and escapes VHL recognition, leading to the accumulation of HIF-1α. Stabilized HIF-1α can translocate to the nucleus, where it forms a heterodimer with the HIF-1β subunit, which binds to specific hypoxia-responsive elements (HREs) promoting the transcription of target genes (Figure 1).^1,9−12^ HIF stabilization and concomitant alterations in gene expression constitute a further opportunity for therapeutic intervention. It can be elicited with PHD inhibitors, which prevent the hydroxylation of HIF. Such drugs are already in clinical use for the treatment of renal anemia in multiple countries, and very recently, the U.S. Food and Drug Administration approved the PHD inhibitor daprodustat in adults on dialysis suffering for anemia caused by a chronic kidney disease.^1,10^

Several chemical strategies have been developed to affect redox homeostasis by HIFs. However, broad-spectrum activities and off-target effects of, for example, proteasome inhibitors or iron chelators provided the impetus for an alternative approach, which is based on interfering with the binding of HIF-1α to VHL.^3,13^ This strategy relies on the blockade of the VHL/HIF-α protein–protein interaction downstream of HIF-α hydroxylation by PHD enzymes and upstream of proteasomal degradation. Small-molecule VHL binders can act as competitors to the native substrate HIF-1α and stabilize HIF-1α levels, upregulating genes involved in the hypoxic response, consequently affecting hypoxia signaling.^3,13^ The successful development of VHL inhibitors has emphasized the importance of VHL as a therapeutic target for the treatment of conditions that occur in anemia, ischemic, inflammatory, or mitochondrial diseases.^3,10^

The rational design of breakthrough VHL inhibitors exploited the structure of the native substrate, that is, hydroxylated HIF-1α, and its molecular recognition by VHL as a starting point.^14−16^ The first co-crystal structure (PDB: 1LM8) of a 20-residue HIF-1α peptide bound to the VHL–EloC–EloB complex (VCB) showed HIF-1α in an extended β strand-like conformation. Hydroxyproline Hyp564, which originated from post-translational hydroxylation, was inserted into a groove in a hydrophobic core formed by buried, mostly aromatic residues.^17^ Hyp564 comprised the essential element for the recognition of HIF-1α derivatives by VHL and served as a central motif for the design of new VHL ligands. The molecular scaffold was extended to both sides of Hyp564 by appending right-hand side (RHS) fragments at the carbonyl group and left-hand side (LHS) fragments at the nitrogen of Hyp564.^3^

**Figure 2:**
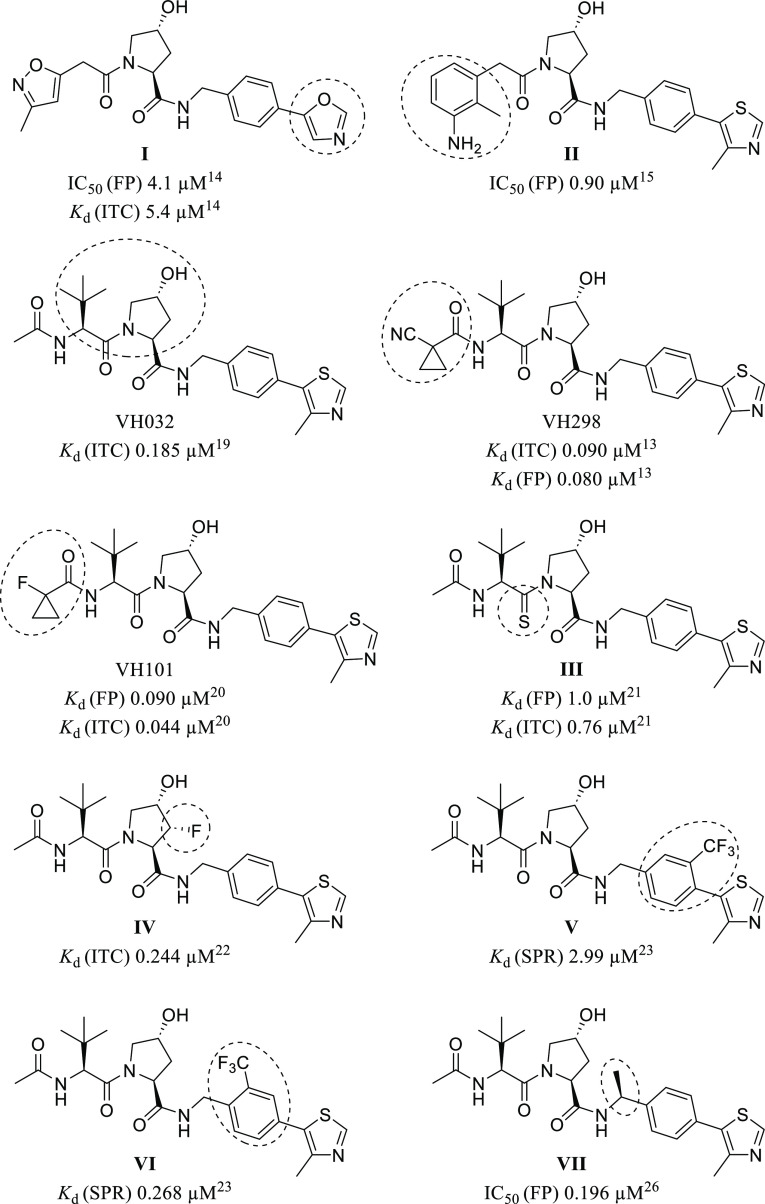
Structures of exemplary VHL inhibitors. Characteristic structural features are highlighted.

Representative compounds that bind to and inhibit VHL are exemplified in Figure 2. Ligands **I** and **II** already contained an RHS benzylamine moiety equipped with a five-membered heteroaromatic ring, a feature that was maintained in the course of further structural optimizations.^14,15^ In ligand **II**, an anilinic LHS fragment was introduced, as well as methylthiazole, a characteristic RHS moiety found in several potent VHL inhibitors.^15,18^ Further structure–activity relationship (SAR) studies identified a *tert*-butyl residue to be advantageous as part of the LHS fragment. Acetylated amino acids other than *tert*-leucine in VH032 caused a reduced binding affinity to VHL.^19^ A constrained cyclopropyl ring with a cyano group or a fluorine substituent was employed, leading to the VHL inhibitors VH298 and VH101, respectively, with VH298 being a widely used benchmark compound, while application of VH101 is limited due to its cytotoxicity.^13,20^ Bioisosteric O-to-S replacements have been performed, for example, to achieve the thioamide derivative **III**, which showed reduced affinity to VHL in comparison to its counterpart VH032.^21^ By introducing fluorohydroxyprolines, other derivatives of VH032 were generated, and all the four 3-fluoro-4-hydroxyproline stereoisomers, for example, the (3*R*,4*S*)-configured derivative **IV**, were investigated to study the influence of hydroxyproline fluorination on VHL binding.^22^ Trifluoromethyl groups were attached at different positions of the inhibitor scaffold, for example, in reporters **V** and **VI**, to be used as ^19^F NMR spy molecules for the hydroxyproline binding site of VHL.^23^ An additional beneficial contribution arose from the stereoselective methylation at the benzylic position within the RHS fragment,^24,25^ and **VII** exhibited an improved IC_50_ value in comparison to the parent VH032.^26^

These examples of VHL inhibitors highlight a route of continuous improvement through structure- and SAR-based optimization. The developed compounds have been demonstrated to be applicable chemical probes and have been successfully employed to assemble proteolysis-targeting chimeras (PROTACs), heterobifunctional molecules capable of exploiting the UPS machinery for targeted protein degradation.^27−29^ However, there are still further opportunities to improve the binding affinity of VHL ligands. In particular, the SAR of the RHS phenylene core has remained largely unexplored so far.^30^ In this study, we aimed to investigate a combinatorially generated library of VHL ligands with high structural diversity at the phenylene core. We conceived a structure-guided and bioactivity-driven design and devised a systematic survey to analyze the chemical space of VHL ligands.

## Results and Discussion

### Analysis of the VH298 Binding Mode to VHL in the VCB Complex

**Figure 3:**
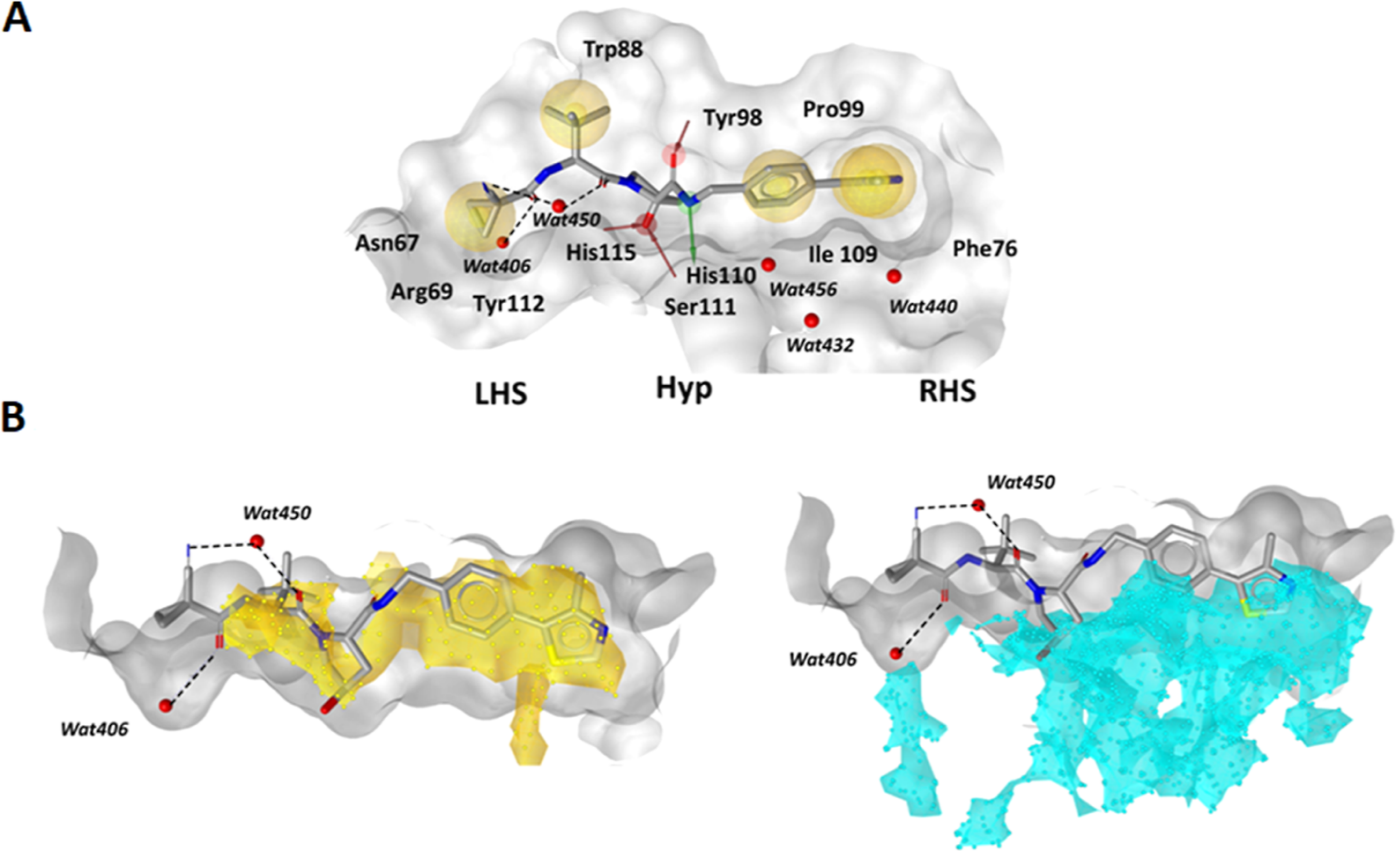
(A) Structure-based pharmacophore of the VH298 ligand bound to the VCB complex. Red and green arrows denote hydrogen bond acceptors and donors, respectively, and yellow spheres indicate areas that enter into hydrophobic interactions (PDB: 5LLI). (B) Calculated hydrophobic MIF (yellow) and buriedness area (cyan) in the active site.

Initially, we utilized the co-crystal structure of the ligand VH298 (Figure 2) bound to the VCB complex (PDB: 5LLI)^13^ to analyze its binding mode and to derive a 3D structure-based pharmacophore (Figure 3A). VH298 is bound to the surface of VHL via multiple hydrogen bonds as well as hydrophobic contacts. A hydrogen bond network is established by the residues Ser111 and His115 with the OH group of the central hydroxyproline of VH298, which further interacts with Tyr98 and His110. The *tert*-butyl and cyclopropyl moieties of VH298 interact with Trp88 and Tyr112, located at the LHS binding pocket, and Phe76, Tyr98, Leu101, and Ile109 from the RHS region form hydrophobic patches with the biaryl motif of VH298. Two interactions of structural water molecules are of particular interest, one of Wat450 with the nitrogen of the cyano group and the *tert*-leucine carbonyl oxygen, the other of Wat406 with the cyclopropanecarbonyl oxygen mediating the contacts with the LHS residues Asn67, Arg69, and His115.^13^ The interactions with the LHS region and the hydrogen bond pattern of hydroxyproline appear better exploited than the interactions with the hydrophobic RHS pocket. Here, several hydrophobic residues such as Phe76, Tyr98, Pro99, Leu101, Ile109, and Trp117 provide ample opportunity to further enhance the binding affinity (Figure S1). To further explore the nature of the binding site, we calculated molecular interaction fields (MIFs)^31^ and estimated the buriedness parameter to evaluate the accessibility of the binding site. In particular, the RHS region showed favorable interactions with the hydrophobic probe, and the buriedness contour confirmed the region surrounding the phenylene core to be capable of accommodating larger moieties (Figure 3B). Overall, this analysis supported our intention to specifically introduce structural variability at the phenylene core of VHL ligands by incorporating additional substituents or even replacing it with bicyclic moieties.

### Synthesis of the First Series of VHL Inhibitors

**Scheme 1:**
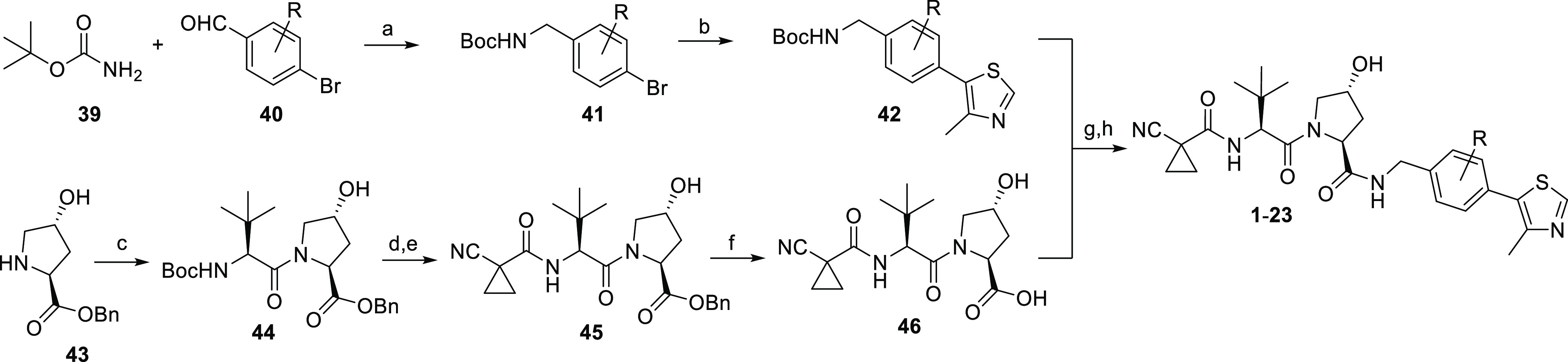
Synthesis of the First Series of VHL Ligands Reagents and conditions: (a) Et_3_SiH, TFA, CH_2_Cl_2_, MeCN, rt, 18 h; (b) 4-methylthiazole, KOAc, PdCl_2_(PPh_3_)_2_, dimethylacetamide, 130 °C, 4 h; (c) Boc-Tle-OH, *O*-(7-azabenzotriazol-1-yl)-*N*,*N*,*N*′,*N*′-tetramethyluronium hexafluorophosphate (HATU), DIPEA, DMF, rt, 18 h; (d) TFA, CH_2_Cl_2_, rt, 2 h; (e) 1-cyano-1-cyclopropanecarboxylic acid, HATU, DIPEA, DMF, rt, 18 h; (f) 10% Pd/C, H_2_, EtOH, rt, 18 h; (g) **42**, TFA, CH_2_Cl_2_, rt, 2 h; and (h) **46**, HATU, DIPEA, DMF, rt, 18 h.

Arising from the structure-based analysis, we conceptualized new VHL ligands with different substituents at the phenylene core. In the first series of compounds, the cyanocyclopropyl group on the LHS was maintained to allow comparability of the biodata within the series and with those of the parent compound VH298 (**1**). The convergent synthetic route to final compounds **1–23** is shown in Scheme 1. Access to such VHL ligands has already been demonstrated by employing readily available 4-bromobenzaldehyde derivatives **40** in a triethylsilane-promoted reductive amination with *tert*-butyl carbamate (**39**).^32^ Our synthesis pursued this protocol, which differed from synthetic entries applying reagents that are available with limited structural variability, that is, 4-bromobenzonitrile^19^ or 4-bromobenzylamine derivatives.^25^ Intermediates **41**, obtained by reductive amination, were subjected to Heck coupling, leading to protected benzylamine derivatives **42**. By incorporating one or two residues at different positions of the arene, a broad substitution pattern was realized in order to achieve structural diversity of the first series of VHL ligands (Table 1). The set of key building blocks **42** included compounds containing naphthalene or quinoline in place of the benzene moiety, ultimately leading to compounds **22** and **23** harboring a bicyclic aromatic substructure (Table 1). For the LHS part of the VHL ligands, benzyl-protected hydroxyproline (**43**) was converted in two uronium salt-mediated coupling reactions via dipeptide **44** to intermediate **45**, followed by hydrogenolytic cleavage of the benzyl ester. The resulting free acid **46** was the sole fragment to be combined through an amide bond with the varying RHS building blocks of type **42** to finally assemble VHL ligands **1–23**.

**Table 1:**
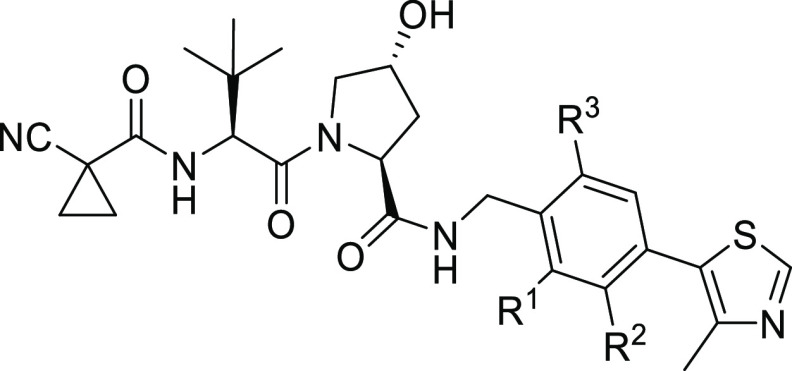
Chemical Structures, Dissociation Constants, Distribution Coefficients, and PPB Properties of VHL Inhibitors **1–23**

inhibitor	R^1^	R^2^	R^3^	*K*_d_ FP (nM)^a^	e log *D*_7.4_^b^	PPB (%)^c^
**1**	H	H	H	129 ± 7^d^	2.3	88
**2**	Me	H	H	149 ± 28	2.0	90
**3**	OMe	H	H	183 ± 22	1.9	88
**4**	F	H	H	297 ± 34	1.9	88
**5**	Cl	H	H	245 ± 20	2.2	91
**6**	H	Me	H	496 ± 62	2.0	89
**7**	H	OMe	H	163 ± 22	1.9	87
**8**	H	F	H	97 ± 11	1.9	88
**9**	H	Cl	H	220 ± 11	2.1	91
**10**	Me	H	Me	4110 ± 560	2.2	90
**11**	OMe	H	OMe	6240 ± 620	2.0	88
**12**	F	H	F	2130 ± 150	1.9	86
**13**	Cl	H	Cl	8150 ± 680	2.4	91
**14**	H	Me	Me	528 ± 16	2.2	90
**15**	H	OMe	OMe	1270 ± 150	1.9	86
**16**	H	F	F	281 ± 46	2.0	88
**17**	H	Cl	Cl	883 ± 68	2.5	93
**18**	Me	Me	H	322 ± 38	2.3	91
**19**	F	F	H	1450 ± 180	2.2	89
**20**	OH	F	H	141 ± 8	1.9	89
**21**	OMe	F	H	305 ± 59	2.1	89
**22**	–CH=CH–CH=CH–		H	397 ± 70	2.3	93
**23**	–N=CH–CH=CH–		H	330 ± 93	1.7	88

a Dissociation constant, determined by FP. Values are mean ± S.E.M. from three independent repeats.

b Experimental distribution coefficient at pH 7.4.

c PPB; experimentally determined percentage of the compound bound to human serum albumin.

d Value is the mean ± S.E.M. from five independent repeats.

**Scheme 2:**
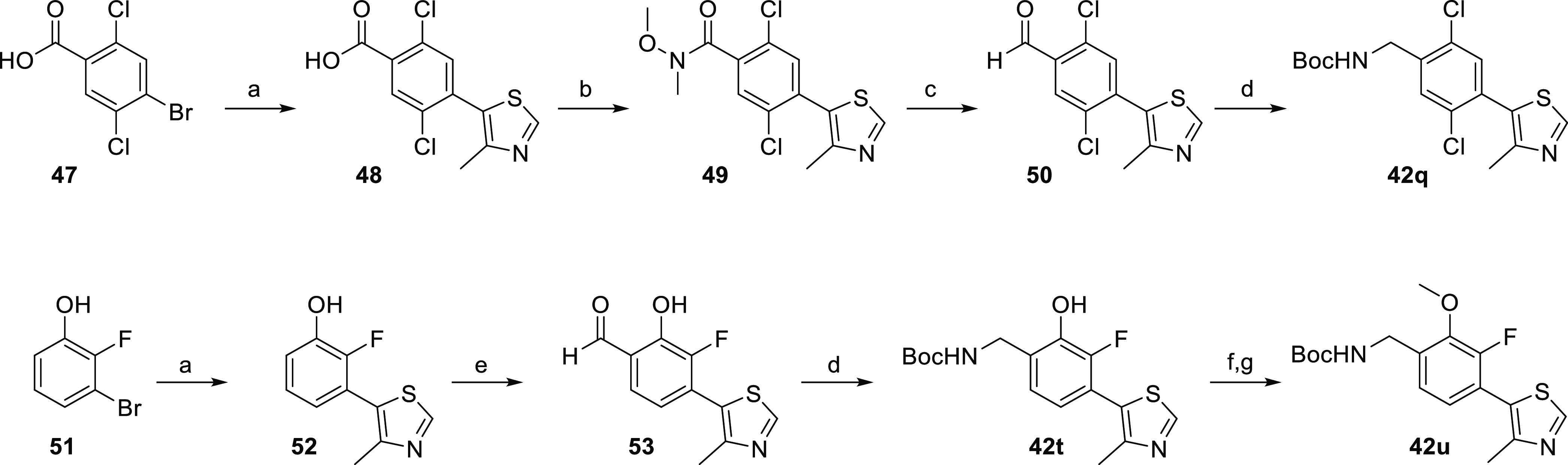
Generation of Three Precursors for VHL Ligands Reagents and conditions: (a) 4-methylthiazole, KOAc, PdCl_2_(PPh_3_)_2_, dimethylacetamide, 130 °C, 4 h; (b) *N*,*O*-dimethylhydroxylamine, EDC × HCl, Et_3_N, CH_2_Cl_2_, rt, 18 h; (c) LiAlH_4_, THF, 0 °C, 1 h; (d) *tert*-butyl carbamate, Et_3_SiH, TFA, CH_2_Cl_2_, MeCN, rt, 18 h; (e) (CH_2_O)_*n*_, Et_3_N, MgCl_2_, THF, reflux, 18 h; (f) Cs_2_CO_3_, DMF, 45 °C, 1 h; and (g) MeI, DMF, rt, 18 h.

Of note, specific entries were elaborated to enable access to defined precursors of type **42** (Scheme 2). To introduce chloro groups at the 2- and 5-position of the phenylene core, we started from 4-bromo-2,5-dichlorobenzoic acid (**47**). After a cross-coupling reaction at an early stage, the biaryl carboxylic acid **48** was submitted to a carbodiimide-assisted conversion with *N*,*O*-dimethylhydroxylamine to give the Weinreb amide **49**. Subsequent reductive cleavage with lithium aluminum hydride furnished aldehyde **50**. The following two steps comprised a reductive amination to **42q** and the generation of the envisaged VHL ligand **17** (Table 1). Alternative access toward **42q** was also examined, where **47** was first converted to the Weinreb amide, followed by reduction and reductive amination. This less advantageous route was terminated at the stage of the corresponding bromobenzylamine of type **41** (Scheme S1). To receive a vicinal hydroxy-fluoro disubstitution (Scheme 2), 3-bromo-2-fluorophenol (**51**) was reacted with 4-methylthiazole to **52**. Subsequently, *ortho*-formylation of the phenol was performed using magnesium chloride, triethylamine, and paraformaldehyde,^33^ giving salicylaldehyde **53**. After reductive amination, the desired intermediate **42t** was obtained and, in turn, *O*-methylated in the presence of cesium carbonate to yield **42u**, a second required intermediate. Both RHS fragments were applied to finalize VHL ligands **20** and **21** (Table 1). An alternative attempt to prepare **42t** from **51** by a formylation-reductive amination-Heck coupling sequence was not successful (Scheme S2). Overall, except for four substitution patterns (Scheme S2), most of the envisaged first-series VHL ligands were successfully synthesized.

### Biophysical Evaluation of the First-Series VHL Inhibitors

To assess the binding affinities of compounds **1–23**, we employed a competitive fluorescence polarization (FP) assay.^19,20^ Binding of a ligand to the HIF binding site of VHL displaces a fluorescein-labeled, 19-mer HIF-1α oligopeptide, leading to a change in polarization of emitted light upon excitation of the competing fluorescent probe. According to our FP measurements (Table 1), ligand **8** showed the highest binding affinity to VHL (*K*_d_ = 97 nM), being in the same range than that of the established VHL inhibitor VH298 (**1**) without a fluorine atom at the R^2^ position. Monosubstitution at the R^1^ position in compounds **2–5** led to *K*_d_ values below 300 nM, with the methyl derivative **2** exhibiting high potency (*K*_d_ = 149 nM). Unsurprisingly, R^1^ substitution was well tolerated since this vector is a suitable linker attachment point in VHL-addressing PROTACs.^3,34−36^ Compounds **10–13** with two residues R^1^ and R^3^ at the positions adjacent to the benzylic moiety were the weakest binders of this series, which might be due to a reduced molecular flexibility. The introduction of R^2^ and R^3^*para* to one another on the phenylene core in **14–17** revealed a disadvantageous effect of the residues’ bulkiness, likely due to a steric clash of R^2^ with the methylthiazole preventing the optimal dihedral angle for the bioactive conformation. In general, when two substituents (R^1^ and R^3^ or R^2^ and R^3^) are located on opposite sides of the arene, one would point inside the protein and would potentially clash with amino acid residues that form the pocket. Actually, when comparing **10–17**, the difluoro derivative **16** with the smallest substituents was more tolerated than those ligands with larger substituents.

The R^1^–R^2^ disubstitution pattern in **18–21** caused unexpected differences in affinities. The vicinal difluoro substitution in **19** may lead to an unfavorable electron-withdrawing effect, which could reduce the efficiency of T-stacking with Tyr98. Such edge-to-face interactions, where the hydrogen of one aromatic system points perpendicular to the center of an aromatic plane, are preferred in protein–ligand binding events.^37,38^ The two ligands with bicyclic arylidene cores, **22** and **23**, had a similar moderate affinity to VHL. Their additional aryl ring is likely pointing out toward the solvent. Overall, in the majority of our first-series VHL ligands, modifications at the phenylene core did not improve affinity, indicating a rather narrow window for improvement. Among the introduced substituents, fluorine appeared to be the most promising, which we took forward into the design of the second series of VHL inhibitors. Two selected VHL ligands of the first series, **2** and **8**, have also been investigated with respect to their ability to stabilize HIF-1α in a cellular context; the data is discussed below.

To assess the druglikeness of the VHL binders, parameters of one physicochemical property, that is, lipophilicity at physiological pH (log *D*_7.4_), and one pharmacokinetic property, that is, plasma protein binding (PPB), are provided in Table 1. Both were experimentally obtained employing HPLC-based protocols.^39,40^ Expectedly, the introduction of two chloro substituents resulted in the most lipophilic compounds (**13** and **17**), whereas the replacement of naphthalene by quinoline (**22** vs **23**) reduced lipophilicity. PPB values were determined in view of how they could impact future pharmacokinetic studies of compounds as VHL inhibitors, or once conjugated into bifunctional PROTACs, for which PPB can be a limiting factor. However, noteworthy differences were not observed within this series of compounds.

### Synthesis of the Second Series of VHL Inhibitors

Based on the SAR of the first-series VHL ligands, we tried to further optimize them with regard to binding affinity by introducing two additional points of diversity. An (*S*)-methyl group at the benzylic position was added, as respective VHL-based PROTACs have previously shown improved VHL binding affinity and as a result also better target protein degradation potency.^24−26,41,42^ Furthermore, at the LHS terminus, besides the cyano group, an α-fluoro substituent at the cyclopropyl moiety has been employed for PROTAC technology,^41,43,44^ and the cyano-to-fluoro replacement caused a moderate improvement in binding affinity to VHL.^20^ Both structural modifications were considered for our second series of VHL ligands, and compounds **2** and **8** were used as starting points for the following structural diversification.

**Scheme 3:**
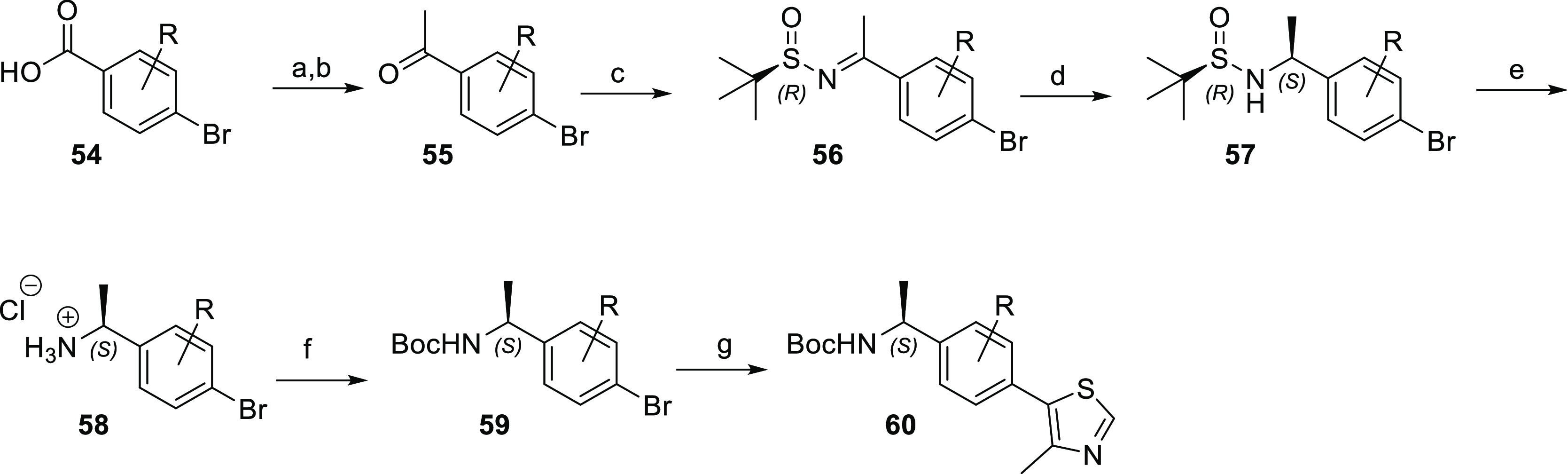
Stereoselective Introduction of the Benzylic Methyl Group into VHL Ligand Precursors Reagents and conditions: (a) *N*,*O*-dimethylhydroxylamine, TBTU, Et_3_N, CH_2_Cl_2_, 0 °C to rt, 18 h; (b) MeMgI, THF, −20 °C to rt, 18 h; (c) (*R*)-(+)-2-methyl-2-propanesulfinamide, Ti(O*i*Pr)_4_, THF, reflux, 24–48 h; (d) L-selectride, THF, 0 °C, 3 h; (e) HCl in dioxane, rt, 2 h; (f) Boc_2_O, NaHCO_3_, EtOAc, H_2_O, 0 °C, 2 h; and (g) 4-methylthiazole, KOAc, PdCl_2_(PPh_3_)_2_, dimethylacetamide, 130 °C, 4 h.

**Scheme 4:**
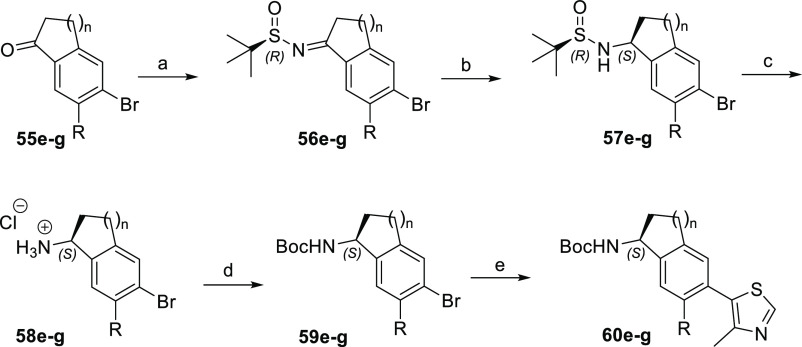
Synthesis of Enantiopure Bicyclic VHL Ligand Precursors Reagents and conditions: (a) (*R*)-(+)-2-methyl-2-propanesulfinamide, Ti(O*i*Pr)_4_, THF, reflux, 24–48 h; (b) L-selectride, THF, 0 °C, 3 h; (c) HCl in dioxane, rt, 2 h; (d) Boc_2_O, NaHCO_3_, EtOAc, H_2_O, 0 °C, 2 h; and (e) 4-methylthiazole, KOAc, PdCl_2_(PPh_3_)_2_, dimethylacetamide, 130 °C, 4 h.

To introduce the (*S*)-configured methyl group, a versatile preparative strategy toward multi-substituted benzylamines with a stereochemically defined methyl group at the benzylic position was accomplished. We started from substituted 4-bromobenzoic acids **54**, which were initially converted to **55** through Weinreb ketone synthesis applying methylmagnesium iodide or directly from the corresponding ketones **55** (Scheme 3). To introduce a chiral center,^45^ compounds **55** were subjected to a condensation reaction with Ellman’s sulfinamide as chiral ammonia equivalent in the presence of Ti(O*i*Pr)_4_ as an additive leading to *N*-sulfinyl imines **56**. These intermediates underwent L-selectride-mediated asymmetric reduction to compounds **57**. As reported, L-selectride gave the opposite sense of induction in comparison to NaBH_4_.^46,47^ Accordingly, the desired (*R*,*S*)-configured sulfinamides **57** were produced and purified by column chromatography to obtain single diastereomers. The auxiliary could easily be cleaved under mildly acidic conditions, and the resulting ammonium chlorides **58** were Boc-protected and coupled with 4-methylthiazole, yielding the building blocks **60**. The (*S*)-configuration at the benzylic position of an exemplary compound of type **59** was confirmed by X-ray crystallography and inspection of the Flack parameter using Bayesian statistics on Bijvoet differences (Figure S9). Particular representatives of type **60** (Scheme 4) were designed to contain the stereogenic center as part of a fused cycloaliphatic ring. By applying the same enantioselective synthetic strategy, the corresponding partially hydrogenated indenone (*n* = 1) or naphthalenone (*n* = 2) derivatives **55e–g** were used as starting materials. The five-step route afforded the bicyclic building blocks **60e–g**.

**Scheme 5:**
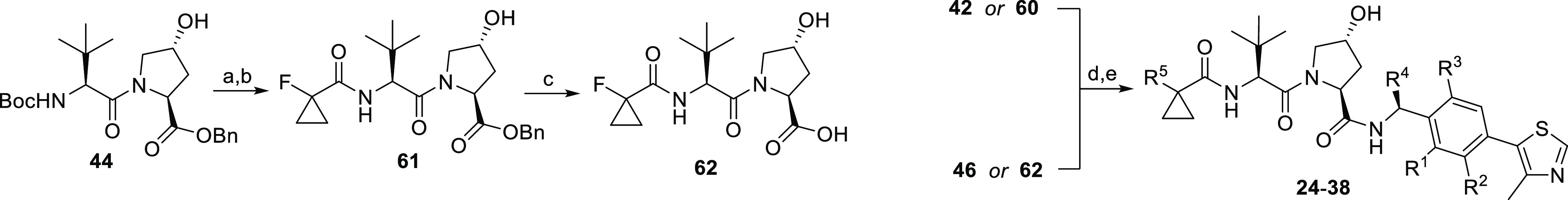
Synthesis of the Second Series of VHL Ligands Reagents and conditions: (a) TFA, CH_2_Cl_2_, rt, 2 h; (b) 1-fluoro-1-cyclopropanecarboxylic acid, HATU, DIPEA, DMF, rt, 18 h; (c) 10% Pd/C, H_2_, EtOH, rt, 18 h; (d) **42** or **60**, TFA, CH_2_Cl_2_, rt, 2 h; and (e) **46** or **62**, HATU, DIPEA, DMF, rt, 18 h.

To introduce the fluorocyclopropyl in place of the cyanocyclopropyl group, dipeptide **44** was coupled to 1-fluoro-1-cyclopropanecarboxylic acid, and the resulting ester **61** was deprotected to the free acid **62** (Scheme 5). With the required building blocks in hand, we could enter the convergent part of the synthesis of the second-series VHL ligands **24–38**. These were combinatorially generated from either the RHS fragments **42** (23 examples; Scheme 1) or **60** (8 examples; Schemes 3 and 4) and from the LHS fragments **46** (Scheme 1) or **62**.

### Biophysical Evaluation of the Second-Series VHL Inhibitors

**Figure 4:**
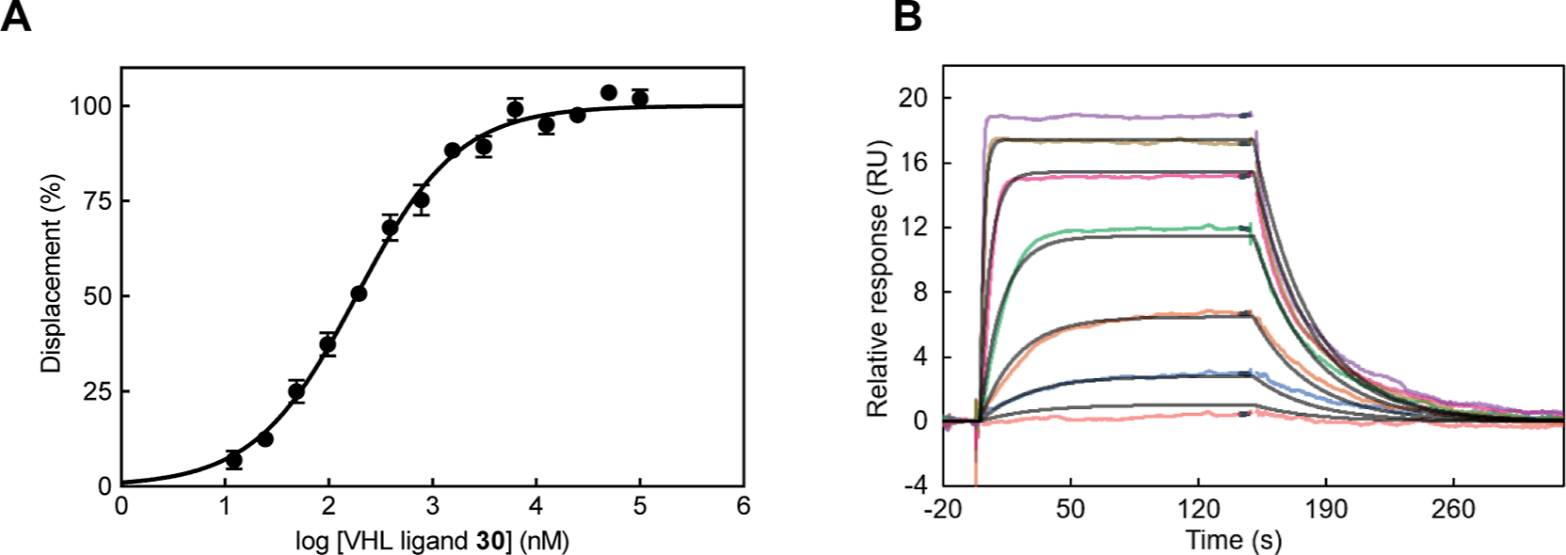
Biophysical characterization of binary complex formation between inhibitor **30** and VCB. (A) Competitive FP binding assay curve, monitoring the displacement of the labeled HIF-1α peptide from VCB by inhibitor **30**. Data show mean ± S.E.M. from one representative experiment in triplicate. (B) SPR sensorgrams monitoring real-time interaction of immobilized biotin-VCB protein with **30** (from top to bottom, 1000, 330, 110, 37, 12.3, 4.12, and 1.37 nM). Association and dissociation rate constants are listed in Table S2. Data was fitted to a 1:1 binding model.

The results of a variety of biophysical, cellular, physicochemical, and pharmacokinetic assays obtained with **1** and **24–38** are listed in Table 2. The exchange of the terminal cyano group by a fluoro substituent provided around 2-fold improvement in binding affinity to VCB in some cases (**30** vs **24**, **31** vs **25**), which is, broadly consistent with the effect of the known compounds VH101 and VH298 (i.e., **38** vs **1**).^20^ However, in other cases (**26** vs **2**, **27** vs **8**), no improvements in binding affinity were observed. Compounds **30–33** are distinguished from their analogues **26–29** by the presence of the methyl group at the (*S*)-configured benzylic carbon, and a minor effect of the (*S*)-methylation was recognizable by comparing their FP data. Gratifyingly, the occurrence of the stereochemically defined methyl group provided four VHL ligands with *K*_d_ values lower than 80 nM. Considering the results from this assay,^19,20,25^ compound **30** (*K*_d_ = 37 nM) constituted one of the most potent VHL ligands known so far (Figure 4A). The subgroup comprising **34–37** included ligands with an alkyl bridge installed from the benzylic carbon to the adjacent phenylene carbon. The induced structural rigidity reduced the affinity (e.g., **36** and **37** vs **31**) of these locked compounds (*K*_d_ > 1 μM).

**Table 2:**
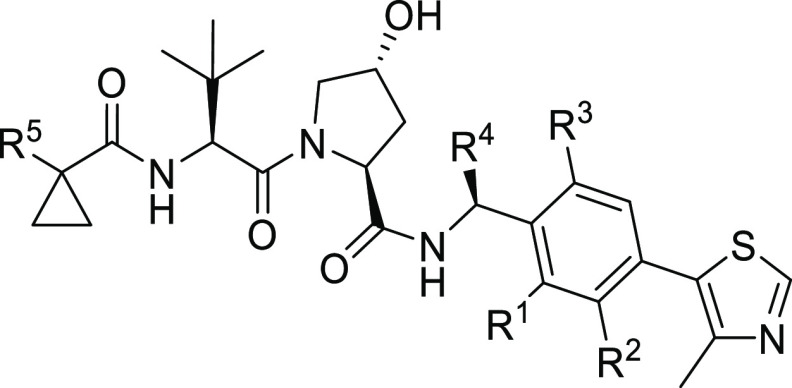
Chemical Structures, Dissociation Constants, Distribution Coefficients, PPB Properties, and HIF-1α Stabilization Capabilities of VHL Inhibitors **1**, **2**, **8**, and **24–38**

								HIF-1α stabilization (%)^c^		HIF-1α-OH stabilization (%)^d^			
inhibitor	R^1^	R^2^	R^3^	R^4^	R^5^	*K*_d_ FP (nM)^a^	*K*_d_ SPR (nM)^b^	HeLa	HEK 293	HeLa	HEK 293	e log *D*_7.4_^e^	PPB (%)^f^
**1**	H	H	H	H	CN	129 ± 7	52^g^	100	100	100	100	2.3	88
**2**	Me	H	H	H	CN	149 ± 28	n.d^h^	89	78	88	66	2.0	90
**8**	H	F	H	H	CN	97 ± 11	n.d.	83	18	74	20	1.9	88
**24**	Me	H	H	Me	CN	86 ± 20	n.d.	155	90	221	110	2.3	88
**25**	H	F	H	Me	CN	186 ± 40	n.d.	105	48	123	52	2.2	88
**26**	Me	H	H	H	F	112 ± 29	41 ± 10	116	78	120	69	2.2	89
**27**	H	F	H	H	F	162 ± 45	66 ± 3	107	89	108	75	2.1	88
**28**	H	F	Me	H	F	80 ± 23	72 ± 8	124	110	153	76	2.3	90
**29**	H	F	OMe	H	F	134 ± 35	34 ± 3	95	73	98	68	2.2	88
**30**	Me	H	H	Me	F	37 ± 10	25 ± 5	182	224	263	208	2.5	88
**31**	H	F	H	Me	F	73 ± 19	45 ± 6	120	92	177	102	2.3	88
**32**	H	F	Me	Me	F	53 ± 7	41 ± 1	155	152	217	137	2.5	89
**33**	H	F	OMe	Me	F	63 ± 9	44 ± 6	128	145	179	137	2.5	89
**34**	H	H	–(CH_2_)_2_–		CN	3920 ± 420	n.d.	n.d.	n.d.	n.d.	n.d.	2.2	90
**35**	H	F	–(CH_2_)_2_–		CN	4040 ± 530	n.d.	n.d.	n.d.	n.d.	n.d.	2.3	90
**36**	H	F	–(CH_2_)_2_–		F	2550 ± 510	n.d.	n.d.	n.d.	n.d.	n.d.	2.3	92
**37**	H	F	–(CH_2_)_3_–		F	1040 ± 240	n.d.	n.d.	n.d.	n.d.	n.d.	2.6	92
**38**	H	H	H	H	F	90 ± 10^g^	16^g^	98	104	105	92	1.8	86

a Dissociation constant, determined by FP. Values are mean ± S.E.M. from three independent repeats.

b Dissociation constant, determined by SPR. Values are mean ± S.E.M. from two independent repeats.

c HeLa or HEK 293 cells were treated with 50 μM of the respective inhibitor, and HIF-1α stabilization levels were detected by western blotting after 2 h treatment. HIF-1α/tubulin protein ratios were normalized to those observed with inhibitor **1** (100%). The mean values of two biologically independent experiments are noted. In the absence of inhibitors, HIF-1α values of 24% (HeLa) and 16% (HEK 293) were obtained.

d HeLa or HEK 293 cells were treated with 50 μM of the respective inhibitor, and HIF-1α-OH stabilization levels were detected by western blotting after 2 h treatment. HIF-1α-OH/tubulin protein ratios were normalized to those observed with inhibitor **1** (100%). The mean values of two biologically independent experiments are noted. In the absence of inhibitors, HIF-1α-OH values of 11% (HeLa) and 9% (HEK 293) were obtained.

e Experimental distribution coefficient at pH 7.4.

f PPB; experimentally determined percentage of the compound bound to human serum albumin.

g Data from ref (20).

h Not determined.

Next, we decided to orthogonally assess the VHL–ligand interaction of eight selected compounds (**26–33**) in a direct binding assay using surface plasmon resonance (SPR) (Table 2). Biotinylated VCB protein was immobilized onto a streptavidin-functionalized sensor chip allowing real-time measurements of the changes in the refractive index upon binding of ligands to the protein.^20^ Our SPR investigations provided double-digit nanomolar values for all selected compounds. As in the FP assay, ligand **30** again possessed the highest affinity to VCB with an SPR-derived *K*_d_ value of 25 nM (Figure 4B). For the eight compounds, second-order rate constants for the association of the binary complexes were between 1.0 × 10^6^ and 1.7 × 10^6^ M^–1^ s^–1^ (Table S2). The strong affinity of the ligands was reflected by these similarly high association rate constants and dissociation half-lives in a narrow range of 8–20 s. Our kinetic data were in line with those of previously investigated, structurally related VHL ligands.^13,20^

The binding of ligands to VHL was also examined computationally by molecular docking to refine a 3D structure-based pharmacophore. MIFs using various molecular, for example, hydrogen bond acceptor/donor and hydrophobic, probes were applied beforehand to identify favorable interaction regions. Following the initial docking setup with two incorporated structural water molecules (Wat406 and Wat450), by a subsequent in-depth examination of the binding pocket, three further structural water molecules (Wat432, Wat440, and Wat456) were identified, located at the top of the binding site, which interact with the system via a network of hydrogen bonds (Figures S1 and S2).^48,49^ Docking poses with compounds **24**, **32**, and **33** were only slightly different, when two or five structural water molecules were incorporated (Figure S3).

In general, VHL ligands used clustered areas for hydrophobic interactions with the target (for examples, see Figure S4). The substituents introduced at the phenylene core of the de novo synthesized active compounds formed favorable hydrophobic contacts with amino acid residues, in particular, Phe76, Tyr98, and Ile109, located in the RHS subpocket (Figure S4).

The replacement of the cyano group (e.g., in **24**) by fluorine (e.g., in **32** and **33**) had no substantial effect on the binding mode since both moieties were docked to occupy a similar position in the LHS subpocket (Figure S4). Structurally related, high-affinity ligands for VHL have been reported both with a terminal fluorocyclopropyl and cyanocyclopropyl moiety,^13,20,34^ but the presence of neither a fluoro nor a cyano group reduced the affinity to VHL.^20^

In our docking approach, only a modest contribution to the overall binding was ascribed to the (*S*)-configured methyl group, which pointed away from the protein (Figure S4). Based on the herein obtained FP results, most desmethyl derivatives were somewhat less potent VHL binders than their methylated counterparts (**2**, **26–29** vs **24**, **30–33**), a result being in accordance with other studies.^24−26,41^ However, certain VHL ligands with an unaltered benzylic position also possessed high affinity to VHL.^13,19,20,34^ These findings reflect the impact of structural plasticity of the target protein on ligand binding,^50−52^ which necessitates the tailored combination of structural features to optimize ligand affinity.

Table 2 encompasses a variety of highly potent VHL ligands, all of which bear either hydrogen or fluorine in place of R^2^. We suppose that sterically demanding substituents are not well tolerated at this position, as they might restrict the rotation about the aryl–aryl axis. To detail the binding mode of these high-affinity ligands, we paid attention to the orientation of their phenylene substituents. The R^1^ methyl group of **24**, the R^2^ fluoro substituent of **32**, and the R^3^ methoxy group of **33** are solvent-exposed. In contrast, the R^3^ methyl group of **32** and the R^2^ fluoro substituent of **33** are directed toward the protein.

The second-series ligands have also been analyzed to determine PPB properties and log *D* values. Consistent with its structure, the bicyclic trimethylene derivative **37** had the highest lipophilicity (Table 2). As with the first series, the PPB values of all compounds within this second series were higher than 85% (Table 2).

**Figure 5:**
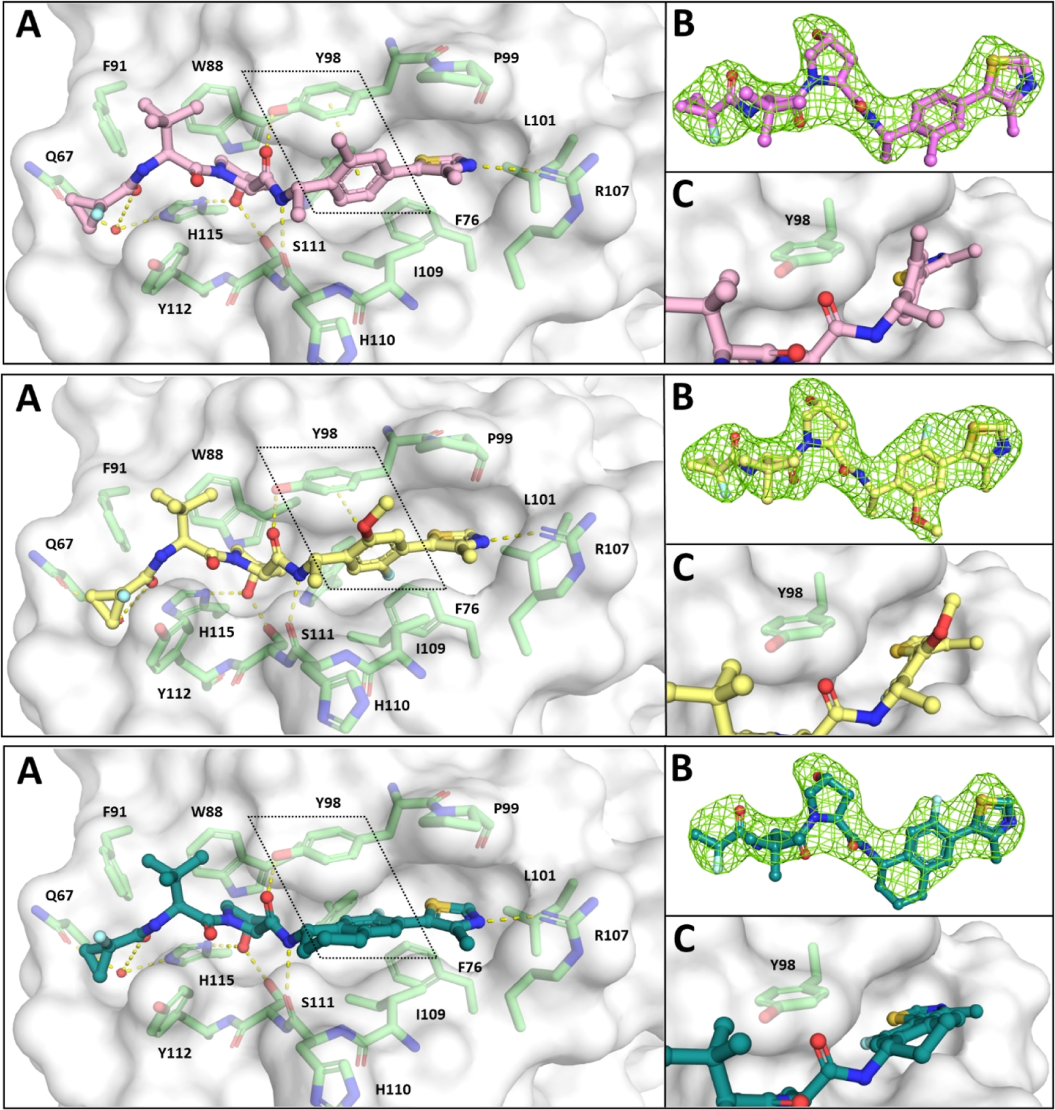
Co-crystal structures of VCB in complex with ligands **30** (top, PDB: 8CQK), **33** (middle, PDB: 8CQL), and **37** (bottom, PDB: 8CQE). (A) Overall binding mode with VHL shown as a white surface and green sticks; the key π–π interaction of the RHS is highlighted. (B) Polder OMIT map (*F*_o_ – *F*_c_) is shown in green contoured at 3σ around each ligand. (C) Close-up view of the phenylene core and Tyr98, highlighting the relative positions of both aromatic rings.

To learn more about the structural features driving affinity to VCB and to verify the binding mode of this class of ligands, X-ray crystallographic analyses were performed. We selected with **30** and **33** two of the most potent compounds, along with **37**, a bicyclic derivative which showed a drastic decrease in affinity. The compounds were soaked into crystals of VCB protein, and ligand-bound structures were successfully solved to 2.6 Å (**30**, PDB: 8CQK), 2.4 Å (**33**, PDB: 8CQL), and 2.9 Å (**37**, PDB: 8CQE) resolutions (Figure 5). In general, these ligands adopted the typical binding mode expected for hydroxyproline-based VHL ligands, maintaining the key interaction network between the protein and the LHS of the ligand. The co-crystal structures revealed, for instance, that the phenylene core of the best compound **30** (Figure 5, top) was situated in a perpendicular orientation to Tyr98 which is optimal for a T-shaped π–π interaction.^37,38^ The three methyl groups of **30** pointed into the solvent and were not engaged in specific interactions with the binding site of the protein, which might have affected the dihedral angles. Hence, the experimental dihedral angles around the phenylene connection to both the benzylic carbon (271°) and the thiazole carbon (60°) were very close to the calculated minima (Figure S5). It could be concluded that this region of the ligand was optimally preorganized into a conformation which allowed for T-stacking with Tyr98 while maintaining the binding mode of the methylthiazole moiety (see also Figure 3A and S4).

The crystallographic complex obtained with **33** (Figure 5, middle) was in good agreement with the corresponding docking pose (Figure S4). The experimentally obtained dihedral angles at the phenylene core (290 and 41°) were in the same range as in the complex with **30**, and also **33** was found to sit in the minimum energy regions (Figure S5). The T-stacking angle was slightly offset, which might be influenced by the fluorine atom pointing toward the protein surface and preventing a more perpendicular orientation. The methoxy group located opposite to the fluoro substituent had the same orientation as the methyl group of **30**.

In the case of compound **37**, which was a weaker binder than **33** and **30** (Table 2), the T-stacking interaction was completely lost due to the inherent rigidity of the bicyclic ring system (Figure 5, bottom). In addition, the planes of the methylthiazole and the aromatic core were almost coplanar, and the dihedral angle of 15° was calculated to be extremely energetically unfavorable (Figure S5). This data emphasized the importance of the interaction with Tyr98 and the advantages that can be gained by preorganizing the protein-bound ligand in a low-energy conformation that is compatible with optimal protein–ligand interactions.

### Determination of the HIF-1α Stabilization by VHL Inhibitors

**Figure 6:**
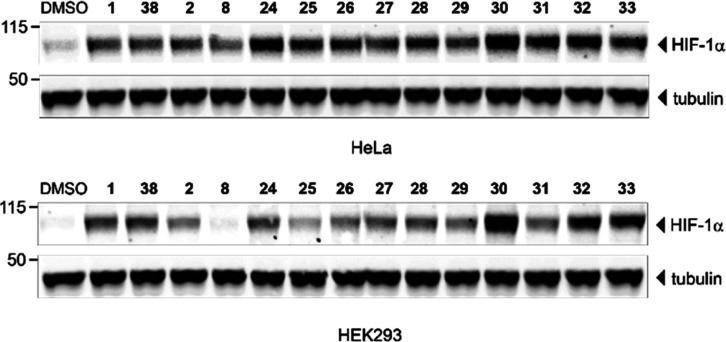
Representative immunoblots of HIF-1α stabilization in HeLa and HEK 293 cells treated with 50 μM of selected VHL inhibitors and 1% DMSO for 2 h.

Next, it was intended to confirm the VHL-inhibiting activity of selected ligands in a cellular environment. All of the tested 14 ligands had an FP-derived *K*_d_ value lower than 200 nM. The corresponding assay is predicated on the blockade of the HIF-1α-OH binding site of VHL by a synthetic ligand, at which successful competition with endogenous HIF-1α-OH protein prevents ubiquitination and hence leads to an accumulation of HIF-1α-OH. The ability of inhibitors to stabilize HIF-1α was inspected both in HeLa and HEK 293 cells by detecting HIF-1α protein levels by means of western blotting analysis (Figure 6). The experiment involved treating cells at an inhibitor concentration of 50 μM for 2 h; these appropriate conditions were adapted from previous studies with related VHL inhibitors.^13,20^ We used primary antibodies against HIF-1α and HIF-1α-OH (Pro564). The quantified results of the immunoblotting experiments (Table 2) were normalized to reference compound **1** (VH298). The HIF-1α antibody used for immunoblotting is unspecific regarding Pro564 hydroxylation and will bind to both HIF-1α and HIF-1α-OH.^13^ As the cells were handled under normoxic conditions, Pro564 hydroxylation will continue to take place, generating HIF-1α-OH, which gets accumulated due to blockage of its binding site at VHL. The HIF-1α-OH antibody, on the other hand, specifically interacts only with HIF-1α-OH hydroxylated at Pro564.^13^

All investigated compounds exhibited HIF-1α stabilization activity compared to the DMSO control (Table 2). Unambiguous SARs could be deduced, which were consonant with the results from our FP and SPR studies. The most promising candidate, ligand **30**, also caused a maximum enhancement of HIF-1α and HIF-1α-OH protein levels in both HeLa and HEK 293 cells. The three most potent HIF stabilizers, that is, **30**, **32**, and **33**, all exhibiting a comparatively high log *D* value of 2.5, outperformed the reference compound **1** considerably (Figure 6 and Table 2), indicating their outstanding cellular activity. Since the applied anti-HIF-1α antibody does not discriminate between the hydroxylated and non-hydroxylated HIF-1α species, the immunoblotting data reflected the increased overall HIF-1α abundance. When employing a specific anti-HIF-1α-OH (Pro564) antibody, congruent data were obtained (Figure S6 and Table 2). The increase in the HIF-1α-OH level can be attributed to two interconnected events: (i) the inhibition of VHL-catalyzed ubiquitination and subsequent proteasomal degradation and (ii) the under normoxic conditions ongoing generation of HIF-1α-OH by PHD, whose activity is controlled by a complex regulation.^53^ As noted in a previous report, the phenylene-unsubstituted compound **38** (VH101) behaved as a superior VHL inhibitor.^20^ The cellular activity of this benchmark compound was considered for reasons of comparability. Encouragingly, compound **30** was shown to be approximately 2-fold more active than **38**, both in HeLa and HEK 293 cells, and with respect to HIF-1α and HIF-1α-OH stabilization. The VHL inhibitor **30** represents the most potent low-molecular-weight HIF-1α stabilizer known so far. Generally, compounds’ overall cell permeability influences their capability for HIF-1α stabilization. However, in most of our inhibitors, the binding affinity and cellular potency correlated well, thus suggesting that permeability was not substantially altered by the subtle structural changes.

**Figure 7:**
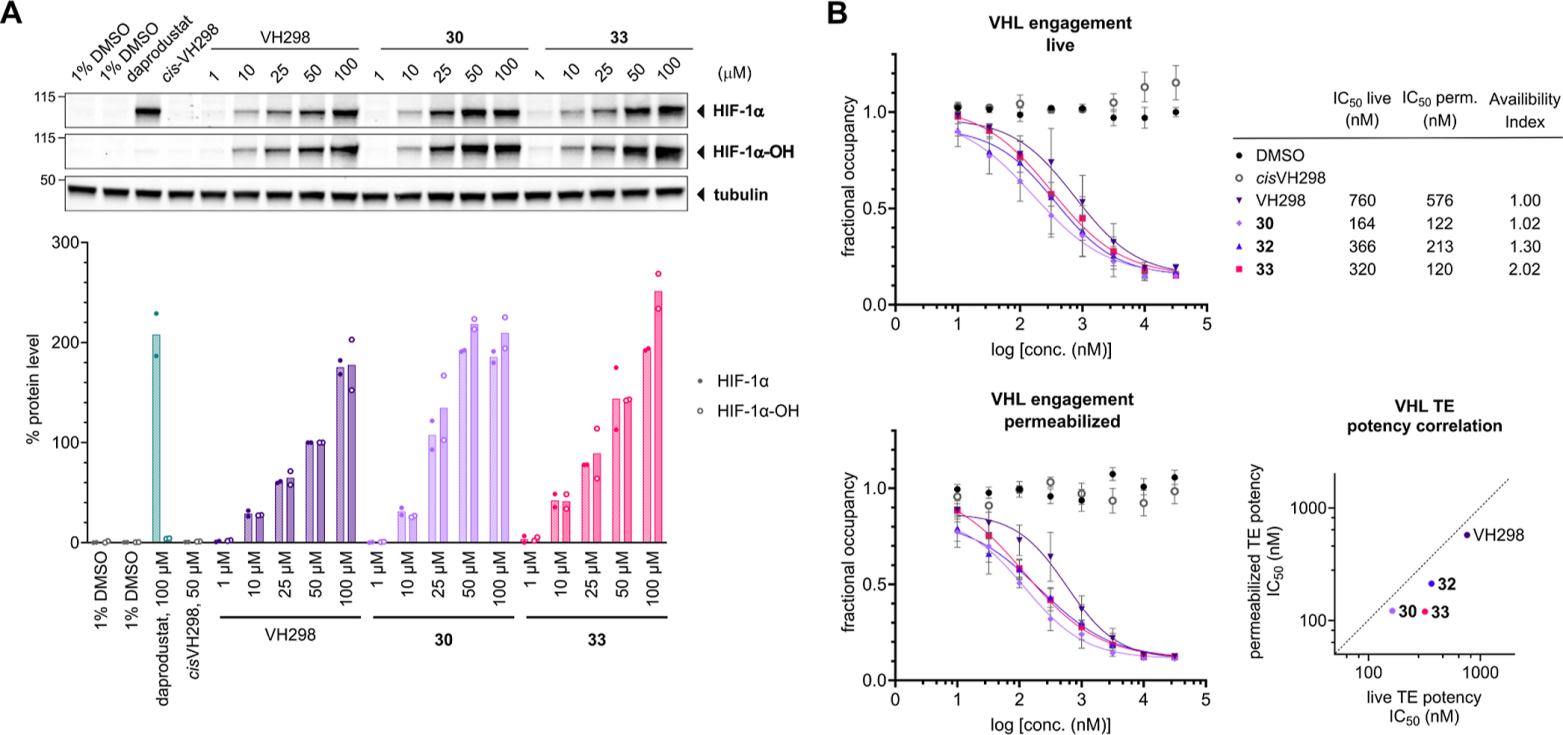
VHL inhibitors **30** and **33** induce increased HIF-1α-OH accumulation compared to VH298. (A) Dose-dependent treatments of HeLa cells with increasing concentrations of the VHL inhibitors **30**, **33**, and **1** (VH298), 100 μM daprodustat, 50 μM *cis*VH298, and 1% DMSO for 1 h. Top: representative immunoblots of HIF-1α and HIF-1α-OH levels after inhibitor treatment. Bottom: Quantification. HIF-1α/tubulin and HIF-1α-OH/tubulin protein ratios were normalized to those observed with **1** (VH298) at 50 μM (100%). Mean values of two biological replicates are depicted. (B) NanoBRET target engagement assays of HEK 293 cells transiently transfected with the VHL–NanoLuc fusion vector in permeabilized and live cell formats. Cells were treated with a fluorescent VHL tracer and incubated for 30 min at room temperature with the indicated compounds across the indicated concentration range to measure competitive displacement. Fractional occupancy was plotted against the concentration of **30**, **32**, **33**, as well as **1** (VH298), *cis*VH298, and 1% DMSO. Mean values ± SEM from three independent experiments are depicted.

To further evaluate the activity of the highly potent compounds **30** and **33**, dose-dependent stabilization of HIF-1α and HIF-1α-OH was studied. HeLa (Figure 7A) and HEK 293 (Figure S7) cells were treated for 1 h with increasing concentrations of compounds **30** and **33**, as well as compound **1** (VH298) as reference, and levels of HIF-1α and HIF-1α-OH were independently monitored by immunoblotting. The PHD inhibitor daprodustat was included as an additional positive control and *cis*VH298, the stereoisomer of **1** with (*S*)-configuration at the hydroxy-substituted carbon, as a negative control. A clear dependency of the compound concentrations ranging from 1 to 100 μM on the protein levels of HIF-1α and HIF-1α-OH was observed with all VHL inhibitors, while the non-binding epimer *cis*VH298 had no effect at 50 μM. As for VH298 (**1**), the levels of HIF-1α and HIF-1α-OH were enhanced by compounds **30** and **33** from as low as 10 μM concentration. At concentrations from 25 up to 100 μM, compounds **30** and **33** outperformed **1** (VH298), with compound **30** already inducing higher HIF-1α and HIF-1α-OH levels at 25 μM than compound **1** at 50 μM.

To verify the cell permeability of **30**, **32**, and **33**, together with **1** (VH298) and *cis*VH298, a bioluminescence resonance energy-transfer (NanoBRET) target engagement assay was conducted. This method relies on measuring the displacement of a fluorescent NanoBRET tracer by the test compound under live-cell and permeabilized-cell conditions. HEK 293 cells expressing VHL fused to luciferase were treated with the unlabeled test compound and a VH298–BODIPY conjugate as the tracer. The outcome of this experiment is shown in Figure 7B. While compounds **30**, **32**, and **33** showed tighter binding to VHL in both live and permeabilized mode compared to **1**, with **30** being the most potent binder, the correlation of their IC_50_ values in live and permeabilized mode indicates an overall similar permeability of these compounds, with **30** possessing a slightly better availability index than **33** (1.02 vs 2.02; Figure 7B).

**Figure 8:**
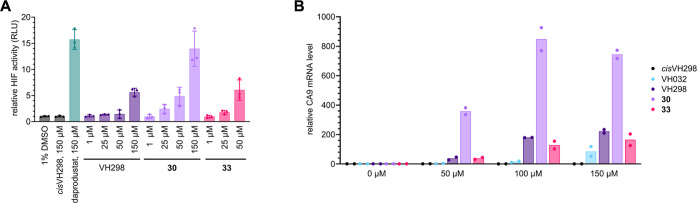
VHL inhibitors **30** and **33** induce increased HIF-α transcriptional activity compared to VH298. (A) HRE-luciferase reporter assay. HeLa cells stably expressing an HRE-luciferase reporter plasmid were treated under indicated conditions for 32 h. The results of treatments with **33** at 150 μM had to be excluded due to apparent cytotoxicity of **33** at this concentration. The mean values ± SEM of three biological replicates are depicted. (B) CA9 mRNA expressions in HeLa cells treated with **30**, **33**, **1** (VH298), VH032, and *cis*VH298 under indicated conditions. After 16 h treatment, mRNA was collected, reverse-transcribed, and analyzed by quantitative real-time PCR. The CA9 mRNA levels were normalized to those of β-actin and depicted relative to 1% DMSO. The mean values of two biological replicates, each representing the mean of three technical replicates, are shown.

Stabilization of HIF-1α and its translocation to the nucleus induces target gene transcription and expression. To monitor the ability of compounds to promote HIF-1α transcriptional activity, a luciferase reporter assay was performed in HeLa (Figure 8A) and U2OS (Figure S8) cells stably expressing an HRE-luciferase reporter.^13,36^ Treatment with compounds **1**, **30**, and **33** caused a concentration-dependent increase in HIF-dependent luciferase activity, while as expected, no activity was observed for *cis*VH298. Inhibitors **30** and **33** proved to be more efficacious than the benchmark compound **1**, with compound **30** even matching the HRE-dependent luciferase activity of the highly potent PHD inhibitor daprodustat at 150 μM.

The cellular activity of **30** and **33** was further validated in a quantitative real-time polymerase chain reaction (qPCR) assay monitoring the relative mRNA level of carbonic anhydrase 9 (CA9), a known HIF target gene and hypoxia-regulated marker (Figure 8B).^13^ Response to the treatment with both compounds as well as **1** (VH298) and VH032, a first-generation VHL inhibitor (Figure 2), was assessed in a dose-dependent manner in HeLa cells. While VH032 upregulated the target gene CA9 only moderately at high concentrations, a significant increase in CA9 mRNA levels was observed with compound **1** (VH298) and inhibitors **30** and **33** already at 50 μM concentration. Despite inducing strong stabilization of HIF-1α and HIF-1α-OH on the protein level, compound **33** did not surpass the performance of **1** in inducing CA9 mRNA levels. Compound **30**, however, greatly outperformed VH298 in the same assay. The stronger effect of **30** in comparison with **33** probably reflects its higher binding affinity and greater cell permeability combined (Figure 7B). Together, the cellular data qualifies compound **30** as the most potent VHL inhibitor.

## Conclusions

In this study, we designed new ligands to explore the chemical space of VHL. Particular attention was paid to the RHS phenylene part of prototypical VH298 analogues. Inspired by the computational insights into specific VHL–ligand interactions and driven by the obtained biodata, an iterative optimization of the ligand structures was realized. However, sole modifications of the substitution pattern at the phenylene unit did not provide a groundbreaking improvement, indicating only limited space for structural alterations at this site. Therefore, a combinatorial assembly was undertaken, which included the LHS cyano-versus-fluoro replacement and the introduction of an (*S*)-configured methyl group at the benzylic position of the ligands. For the latter variation, a stereoselective synthetic entry to diversely substituted 1-phenylethan-1-amine building blocks was accomplished. The stepwise improvement related not only to the affinity of the ligands to VHL, as it was monitored by biophysical techniques, but also to their cellular activity as a measure of the compounds’ ability to penetrate cells, stabilize the protein level of HIF-1α, and induce HIF transcriptional activity.

**Figure 9:**
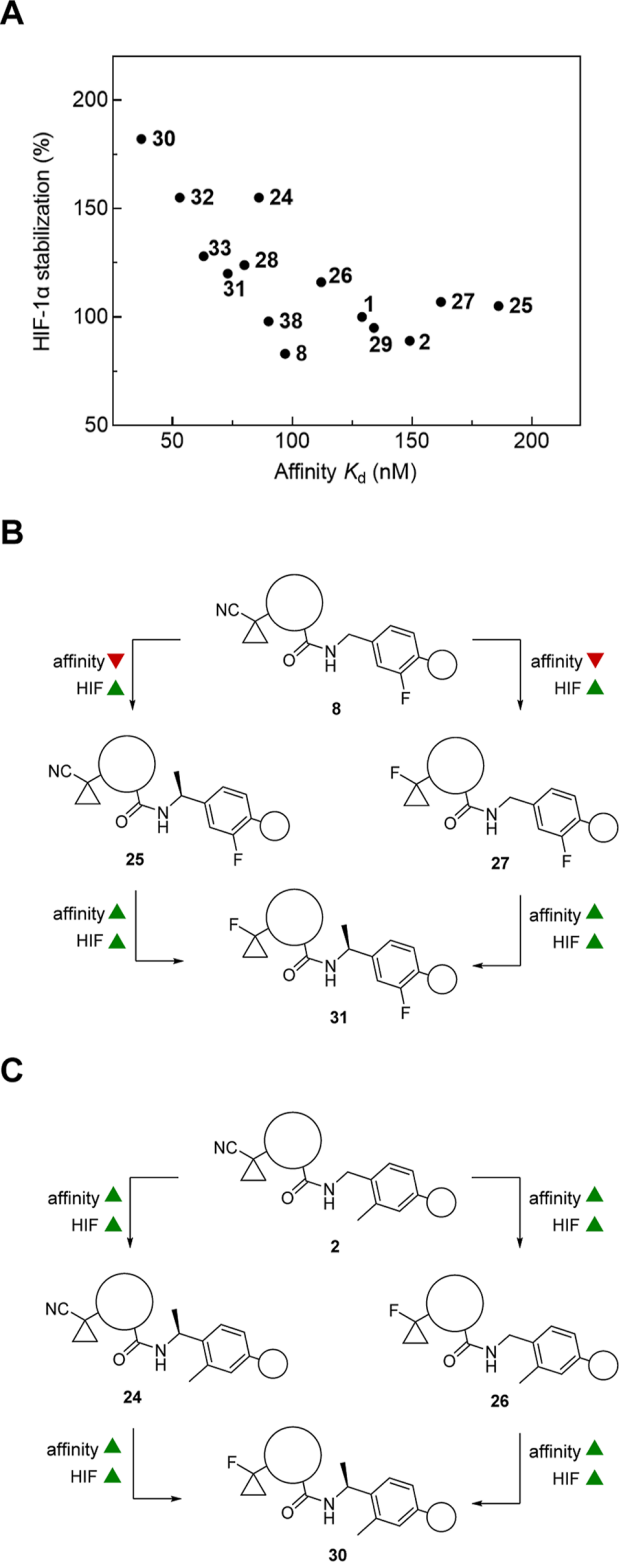
(A) Correlation of the compounds’ binding affinity to VCB as determined by FP and their ability to stabilize HIF-1α in HeLa cells as determined by immunoblotting. (B,C) Bioactivity-driven design pathways to VHL ligands gave rise to (B) compound **31** and (C) compound **30**. Structural optimization is shown regarding (i) FP-derived affinity to VCB, and (ii) HIF-1α and HIF-1α-OH stabilization. Circles exemplify the unaltered substructures. Green and red triangles indicate improvement and deterioration, respectively.

The correlation of binding affinity and cellular potency of selected compounds is shown in Figure 9A. The monofluoro substitution pattern of **8** was maintained en route to **25** and **27**, including either the benzylic (*S*)-methylation or cyano-to-fluoro replacement. These modifications were accompanied by ∼2-fold lower binding affinity, yet consistently higher capability for HIF-1α stabilization (Figure 9B), probably arguing for a better cell permeability in cases of **25** and **27** compared to **8**. The readjustment of the second point of diversity resulted in **31** with improved properties. The second design pathway (Figure 9C) provided an even clearer picture. Both structural modifications were additive and advantageous for VHL affinity as well as cellular HIF-1α stabilization. In compound **30**, both features were realized to generate an exceptional VHL inhibitor capable of most potently increasing the intracellular level of HIF-1α. Moreover, we characterized **30** as an efficacious, small-molecule inhibitor of VHL inducing the HIF transcriptional activation pathway and demonstrated the superiority of **30** above and beyond both previously reported VHL ligands and all other new ones reported herein. Inhibitor **30** is expected to serve as a valuable tool compound to study the hypoxia signaling pathway, which is predominantly governed by HIF-1α regulation. Inhibitor **30** may also act as a lead compound for developing drugs to be applied under conditions where the adaptive cell response against hypoxia would be beneficial.

Our work aimed to employ an extended series of compounds to scan the ligand binding site of VHL. An additional essential facet of this study arose from the opportunity to utilize the structural variability of ligands for expanding the repertoire of VHL-recruiting PROTACs. We explored SARs of ligands whose LHS-terminal amino moiety is blocked by the well-described fluoro- or cyanocyclopropyl capping group. However, there are further exit vectors on VHL ligands accessible for linker attachment which can be employed for PROTAC design. Such approaches, beyond the linkage at the LHS amino moiety or at a phenolic group of the RHS phenylene core, have recently emerged as attractive strategies to efficiently hijack VHL. These alternative exit vectors include a thioether connection at the side chain of *tert*-leucine or the appendage of the linker via an acetamide portion at the benzylic position.^3,34,54^ For future PROTAC design, such linker attachments might be pursued by exploiting the structure of the optimized VHL ligand **30**. The assembly of the so-designed compounds is currently under investigation in our laboratories.

## Experimental Section

### Chemistry

#### General Synthetic Methods and Materials

Commercially available starting reagents for each reaction were purchased from Sigma-Aldrich, Fluorochem, TCI, ABCR, Merck, or Acros Organics and used without further purification. All reactions were carried out using anhydrous solvents. Preparative column chromatography was performed on Merck silica gel (0.063–0.200 mm, 60 Å) or using an automated flash column chromatography system puriFlash XS520Plus (Interchim, Montluçon, France). Thin-layer chromatography was carried out on Merck (Darmstadt, Germany) aluminum sheets, silica gel 60 F_254_. Detection was performed with UV light at 254 nm. Retention factors (*R*_f_) are indicated. Melting points were determined on a Büchi (Essen, Germany) 510 oil bath apparatus. ^1^H NMR and ^13^C NMR spectra were recorded on a Bruker AVANCE 400 MHz NMR spectrometer, a Bruker AVANCE DRX 500 MHz NMR spectrometer, or a Bruker AVANCE III 600 MHz NMR spectrometer. NMR spectra were processed and analyzed in MestReNova. Chemical shifts are given in parts per million (ppm), coupling constants *J* are given in hertz (Hz), and standard abbreviations are used to indicate spin multiplicities. In the case of rotamers, only the peaks for the major rotamer are given. Assignments were made based on one- and two-dimensional NMR techniques, which include ^1^H, ^13^C, DEPT, HSQC, and HMBC experiments. LC–MS analyses were carried out on an API2000 (Applied Biosystems, Darmstadt, Germany) or an Expression CMSL (Advion, Ithaca, NY, USA) mass spectrometer coupled to an Agilent (Santa Clara, CA, USA) 1100 or 1260 Infinity II LC system. An EC50/2 NUCLEODUR C18 Gravity 3 μm column (Macherey-Nagel, Düren, Germany) or a XBridge BEH C18 3.5 μm column (Waters, Eschborn, Germany) was used. Samples (1 mg/mL) were dissolved in MeOH containing 2 mM ammonium acetate or MeOH. A volume of 8 or 10 μL was injected into the column at 25 °C or at 40 °C. The flow rate was 0.3 mL/min or 1.5 mL/min. Unless stated otherwise, the mobile phase was a gradient of 90% H_2_O to 100% MeOH containing 2 mM ammonium acetate in 10 min and then 100% MeOH containing 2 mM ammonium acetate to 20 min. For purity determination, diode array detection (DAD) was applied in the range of 220–400 nm. Positive total ion scans were observed from 150 to 800 *m*/*z*. High-resolution mass spectrometry (HRMS) spectra were recorded on a Thermo Scientific Q Exactive Plus mass spectrometer (Thermo Fisher Scientific). LC–MS was used to determine the purity of the compounds. The area under the curve of all peaks, except of the injection peak, were added and set to 100%. The purity of all the final compounds was confirmed to be ≥95%, as analyzed by LC–MS. *cis*VH298 was available from previous studies.^13^ Daprodustat was obtained from Biosynth (UK).

#### General Procedure A. Reductive Amination

*tert*-Butyl carbamate (**39**; 3 equiv) and the corresponding benzaldehyde derivative **40** (1 equiv) were dissolved in CH_2_Cl_2_ (2 mL/mmol) and MeCN (6 mL/mmol). Et_3_SiH (3 equiv) was added, followed by the dropwise addition of TFA (2 equiv). After stirring for 18 h at rt, the mixture was quenched with saturated aqueous NaHCO_3_ (10 mL/mmol), and the aqueous layer was extracted with CH_2_Cl_2_ (3 × 10 mL/mmol). The combined organic phases were washed with brine (10 mL/mmol), dried over Na_2_SO_4_, filtered, and concentrated in vacuo.

#### General Procedure B. Heck Coupling

The corresponding bromoaryl compound **41** (1 equiv), PdCl_2_(PPh_3_)_2_ (0.1 equiv), and KOAc (4 equiv) were dissolved in *N*,*N*-dimethylacetamide (5 mL/mmol). 4-Methylthiazole (4 equiv) was added, and the mixture was heated to 130 °C under an argon atmosphere for 4 h. Subsequently, the mixture was allowed to cool to rt, diluted with H_2_O (25 mL/mmol), and extracted with CH_2_Cl_2_ (3 × 25 mL/mmol). The combined organic layers were washed with brine (25 mL/mmol), dried over Na_2_SO_4_, filtered, and concentrated in vacuo.

#### General Procedure C. Boc-Deprotection and HATU-Promoted Amide Coupling

The corresponding Boc-protected amine (1 equiv) was dissolved in anhydrous CH_2_Cl_2_ (5 mL/mmol), and TFA (5 mL/mmol) was added. The mixture was stirred at rt for 2 h and then concentrated under high vacuum. The deprotected amine was dissolved in anhydrous DMF (5 mL/mmol), and the appropriate acid (1 equiv) was added. DIPEA (4 equiv) was added, followed by the addition of HATU (1.1 equiv) after 5 min. The mixture was stirred at rt for 18 h, after which H_2_O (50 mL/mmol) was added, and extracted with EtOAc (3 × 25 mL/mmol). The combined organic phases were washed with brine (50 mL/mmol), dried over Na_2_SO_4_, filtered, and concentrated in vacuo.

#### General Procedure D. Preparation of *N*-Sulfinyl Imines

The corresponding ketone (1 equiv) and (*R*)-(+)-2-methyl-2-propanesulfinamide (1.5 equiv) were dissolved in dry THF. Ti(OiPr)_4_ was added, and the mixture was stirred under reflux for 1–2 days. Reactions were monitored by LC–MS. After the reaction was complete, saturated aqueous NH_4_Cl (25 mL/mmol) and EtOAc (25 mL/mmol) were added to the mixture, and phases were separated. After extraction with EtOAc (3 × 25 mL/mmol), the organic phases were combined, washed with brine (25 mL/mmol), dried over Na_2_SO_4_, filtered, and concentrated in vacuo.

#### General Procedure E. L-Selectride-Promoted Reduction

The corresponding *N*-sulfinyl imine (1 equiv) was dissolved in dry THF (10 mL/mmol) and was cooled to 0 °C. L-selectride (1.0 M in THF, 3 equiv) was slowly added, and the solution was allowed to warm to rt over a 3 h period. The solution was then concentrated under high vacuum. CH_2_Cl_2_ (25 mL/mmol) and 10% aqueous citric acid (25 mL/mmol) were added to the residue, and after separation of the phases, the organic phase was washed with brine (25 mL/mmol), dried over Na_2_SO_4_, filtered, and concentrated in vacuo.

#### General Procedure F. Deprotection of Sulfinamides

The corresponding sulfinamide (1 equiv) was dissolved in dry dioxane, and HCl (4 M in dioxane, 3 equiv) was added. After stirring for 2 h at rt, the suspension was concentrated in vacuo. Then, Et_2_O (25 mL/mmol) was added to the residue, and the product was filtered off and washed with Et_2_O.

#### General Procedure G. N-Boc Protection

The corresponding amine (1 equiv) was dissolved in H_2_O (5 mL/mmol). NaHCO_3_ (1.1 equiv) and a solution of Boc_2_O (1.6 equiv) in EtOAc and H_2_O (1:1) were added to the mixture at 0 °C. After stirring for 2 h at 0 °C, the phases were separated, and the organic phase was washed with saturated aqueous NaHCO_3_ (25 mL/mmol) and brine (25 mL/mmol), dried over Na_2_SO_4_, filtered, and concentrated in vacuo. The product was used in the next step without further purification.

##### (2*S*,4*R*)-1-((*S*)-2-(1-Cyanocyclopropane-1-carboxamido)-3,3-dimethylbutanoyl)-4-hydroxy-*N*-(4-(4-methylthiazol-5-yl)benzyl)pyrrolidine-2-carboxamide (**1**)

This compound was synthesized as described previously.^13^

##### (2*S*,4*R*)-1-((*S*)-2-(1-Cyanocyclopropane-1-carboxamido)-3,3-dimethylbutanoyl)-4-hydroxy-*N*-(2-methyl-4-(4-methylthiazol-5-yl)benzyl)pyrrolidine-2-carboxamide (**2**)

Following general procedure C, compound **2** was obtained using Boc-protected amine **42b** (type **42**, R = 2-Me; 127 mg, 0.4 mmol) and acid **46** (135 mg, 0.4 mmol). The residue was purified by flash column chromatography using a gradient from 0 to 10% MeOH in CH_2_Cl_2_ to afford **2** as a white solid. Yield: 48 mg (22%); mp 164–166 °C; *R*_f_ = 0.50 (CH_2_Cl_2_/MeOH 9:1); ^1^H NMR (600 MHz, DMSO-*d*_6_): δ 0.95 (s, 9H), 1.46–1.53 (m, 2H), 1.58–1.66 (m, 2H), 1.88–1.94 (m, 1H), 2.03–2.10 (m, 1H), 2.30 (s, 3H), 2.44 (s, 3H), 3.12–3.16 (m, 1H), 3.60–3.65 (m, 1H), 4.22 (dd, *J* = 15.5, 5.4 Hz, 1H), 4.31–4.37 (m, 2H), 4.47–4.54 (m, 2H), 5.14 (d, *J* = 3.7 Hz, 1H), 7.23 (dd, *J* = 7.9, 1.9 Hz, 1H), 7.28 (d, *J* = 2.0 Hz, 1H), 7.35 (d, *J* = 8.9 Hz, 1H), 7.41 (d, *J* = 7.8 Hz, 1H), 8.49 (t, *J* = 5.7 Hz, 1H), 8.97 (s, 1H); ^13^C NMR (126 MHz, DMSO-*d*_6_): δ 12.4, 13.7, 15.9, 16.7, 18.1, 26.1, 36.2, 37.9, 41.8, 56.6, 57.3, 58.7, 68.9, 120.1, 126.1, 127.9, 129.8, 130.3, 131.1, 136.4, 136.9, 147.6, 151.3, 164.4, 168.6, 171.4; LC–MS (ESI) (90% H_2_O to 100% MeCN in 10 min, then 100% MeCN to 15 min, DAD 200–600 nm), *t*_R_ = 5.68 min, 97% purity, *m*/*z* calcd for C_28_H_35_N_5_O_4_S [M + H]^+^, 538.25; found, 538.5. HRMS (ESI) *m*/*z*: calcd for C_28_H_35_N_5_O_4_S [M + H]^+^, 538.2482; found, 538.2482.

##### (2*S*,4*R*)-1-((*S*)-2-(1-Cyanocyclopropane-1-carboxamido)-3,3-dimethylbutanoyl)-4-hydroxy-*N*-(2-methoxy-4-(4-methylthiazol-5-yl)benzyl)pyrrolidine-2-carboxamide (**3**)

Following general procedure C, compound **3** was obtained using Boc-protected amine **42c** (type **42**, R = 2-OMe; 100 mg, 0.3 mmol) and acid **46** (101 mg, 0.3 mmol). The residue was purified by flash column chromatography using a gradient from 0 to 10% MeOH in CH_2_Cl_2_ to afford **3** as a white solid. Yield: 23 mg (14%); mp 168–170 °C; *R*_f_ = 0.50 (CH_2_Cl_2_/MeOH 9:1); ^1^H NMR (600 MHz, DMSO-*d*_6_): δ 0.95 (s, 9H), 1.46–1.53 (m, 2H), 1.59–1.66 (m, 2H), 1.89–1.94 (m, 1H), 2.05–2.10 (m, 1H), 2.47 (s, 3H), 3.56 (d, *J* = 10.8 Hz, 1H), 3.63 (dd, *J* = 10.8, 3.9 Hz, 1H), 3.85 (s, 3H), 4.18–4.30 (m, 2H), 4.31–4.36 (m, 1H), 4.48–4.54 (m, 2H), 5.14 (d, *J* = 3.6 Hz, 1H), 6.96 (dd, *J* = 7.7, 1.6 Hz, 1H), 7.01–7.04 (m, 1H), 7.38 (dd, *J* = 23.8, 8.3 Hz, 2H), 8.48 (t, *J* = 6.0 Hz, 1H), 8.98 (s, 1H); ^13^C NMR (151 MHz, DMSO-*d*_6_): δ 13.7, 16.0, 16.6, 16.8, 26.1, 36.2, 37.1, 37.8, 55.5, 56.6, 57.3, 58.8, 68.9, 110.9, 120.1, 120.7, 126.8, 127.9, 131.0, 131.3, 147.9, 151.4, 156.5, 164.4, 168.7, 171.7; LC–MS (ESI) (90% H_2_O to 100% MeCN in 10 min, then 100% MeCN to 15 min, DAD 200–600 nm), *t*_R_ = 5.58 min, 96% purity, *m*/*z* calcd for C_28_H_35_N_5_O_5_S [M + H]^+^, 554.24; found, 554.3. HRMS (ESI) *m*/*z*: calcd for C_28_H_35_N_5_O_5_S [M + H]^+^, 554.2432; found, 554.2431.

##### (2*S*,4*R*)-1-((*S*)-2-(1-Cyanocyclopropane-1-carboxamido)-3,3-dimethylbutanoyl)-*N*-(2-fluoro-4-(4-methylthiazol-5-yl)benzyl)-4-hydroxypyrrolidine-2-carboxamide (**4**)

Following general procedure C, compound **4** was obtained using Boc-protected amine **42d** (type **42**, R = 2-F; 97 mg, 0.3 mmol) and acid **46** (101 mg, 0.3 mmol). The residue was purified by flash column chromatography using a gradient from 0 to 10% MeOH in CH_2_Cl_2_ to afford **4** as a pale brown solid. Yield: 32 mg (20%); mp 136–138 °C; *R*_f_ = 0.27 (CH_2_Cl_2_/MeOH 9:1); ^1^H NMR (600 MHz, DMSO-*d*_6_): δ 0.94 (s, 9H), 1.45–1.53 (m, 2H), 1.58–1.67 (m, 2H), 1.86–1.92 (m, 1H), 2.04–2.09 (m, 1H), 2.46 (s, 3H), 3.57 (dt, *J* = 11.0, 1.7 Hz, 1H), 3.63 (dd, *J* = 10.8, 3.9 Hz, 1H), 4.26–4.40 (m, 3H), 4.46–4.54 (m, 2H), 5.15 (d, *J* = 3.7 Hz, 1H), 7.22 (dd, *J* = 7.9, 1.8 Hz, 1H), 7.27–7.40 (m, 2H), 7.54 (t, *J* = 8.0 Hz, 1H), 8.64 (t, *J* = 5.9 Hz, 1H), 9.02 (s, 1H); ^13^C NMR (151 MHz, DMSO-*d*_6_): δ 13.7, 15.9, 16.6, 16.8, 26.0, 35.8 (d, ^3^*J*_F,C_ = 4.1 Hz), 36.2, 37.8, 56.6, 57.3, 58.7, 68.9, 115.2 (d, ^2^*J*_F,C_ = 22.5 Hz), 120.1, 124.7 (d, ^4^*J*_F,C_ = 2.4 Hz), 125.7 (d, ^2^*J*_F,C_ = 14.3 Hz), 129.8 (d, ^3^*J*_F,C_ = 5.2 Hz), 132.0, 132.0, 148.5, 152.0, 159.7 (d, ^1^*J*_F,C_ = 245.4 Hz), 164.4, 168.7, 171.8; LC–MS (ESI) (90% H_2_O to 100% MeOH in 10 min, then 100% MeOH to 20 min, DAD 220–400 nm), *t*_R_ = 10.58 min, 99% purity, *m*/*z* calcd for C_27_H_32_FN_5_O_4_S [M + H]^+^, 542.65; found, 542.4. HRMS (ESI) *m*/*z*: calcd for C_27_H_32_FN_5_O_4_S [M + H]^+^, 542.2232; found, 542.2232.

##### (2*S*,4*R*)-*N*-(2-Chloro-4-(4-methylthiazol-5-yl)benzyl)-1-((*S*)-2-(1-cyanocyclopropane-1-carboxamido)-3,3-dimethylbutanoyl)-4-hydroxypyrrolidine-2-carboxamide (**5**)

Following general procedure C, compound **5** was obtained using Boc-protected amine **42e** (type **42**, R = 2-Cl; 101 mg, 0.3 mmol) and acid **46** (101 mg, 0.3 mmol). The residue was purified by flash column chromatography using a gradient from 0 to 10% MeOH in CH_2_Cl_2_ to afford **5** as a white solid. Yield: 13 mg (8%); mp 160–162 °C; *R*_f_ = 0.55 (CH_2_Cl_2_/MeOH 9:1); ^1^H NMR (600 MHz, DMSO-*d*_6_): δ 0.94 (s, 9H), 1.47–1.54 (m, 2H), 1.59–1.67 (m, 2H), 1.92 (ddd, *J* = 13.1, 9.0, 4.5 Hz, 1H), 2.06–2.12 (m, 1H), 2.45 (s, 3H), 3.55–3.59 (m, 1H), 3.64 (dd, *J* = 10.8, 3.9 Hz, 1H), 4.30 (dd, *J* = 16.4, 5.7 Hz, 1H), 4.34–4.37 (m, 1H), 4.39 (dd, *J* = 16.5, 6.2 Hz, 1H), 4.49–4.55 (m, 2H), 5.16 (d, *J* = 3.6 Hz, 1H), 7.34–7.40 (m, 2H), 7.54 (d, *J* = 1.9 Hz, 1H), 7.61 (d, *J* = 8.0 Hz, 1H), 8.72 (t, *J* = 6.0 Hz, 1H), 9.03 (s, 1H); ^13^C NMR (151 MHz, DMSO-*d*_6_): δ 13.7, 15.9, 16.6, 16.8, 26.1, 36.2, 37.8, 56.6, 57.3, 58.8, 68.9, 120.1, 127.5, 128.9, 129.1, 129.5, 131.7, 132.2, 136.0, 148.6, 152.1, 164.4, 168.8, 171.9; LC–MS (ESI) (90% H_2_O to 100% MeCN in 10 min, then 100% MeCN to 15 min, DAD 200–600 nm), *t*_R_ = 5.88 min, 96% purity, *m*/*z* calcd for C_27_H_32_ClN_5_O_4_S [M + H]^+^, 558.19; found, 558.4. HRMS (ESI) *m*/*z*: calcd for C_27_H_32_ClN_5_O_4_S [M + H]^+^, 558.1936; found, 558.1936.

##### (2*S*,4*R*)-1-((*S*)-2-(1-Cyanocyclopropane-1-carboxamido)-3,3-dimethylbutanoyl)-4-hydroxy-*N*-(3-methyl-4-(4-methylthiazol-5-yl)benzyl)pyrrolidine-2-carboxamide (**6**)

Following general procedure C, compound **6** was obtained using Boc-protected amine **42f** (type **42**, R = 3-Me; 95 mg, 0.3 mmol) and acid **46** (101 mg, 0.3 mmol). The residue was purified by flash column chromatography using a gradient from 0 to 10% MeOH in CH_2_Cl_2_ to afford **6** as a white solid. Yield: 25 mg (16%); mp 88–90 °C; *R*_f_ = 0.40 (CH_2_Cl_2_/MeOH 9:1); ^1^H NMR (600 MHz, DMSO-*d*_6_): δ 0.95 (s, 9H), 1.46–1.52 (m, 2H), 1.58–1.65 (m, 2H), 1.88–1.93 (m, 1H), 2.05–2.10 (m, 1H), 2.12 (s, 3H), 2.17 (s, 3H), 3.55–3.59 (m, 1H), 3.64 (dd, *J* = 10.8, 3.9 Hz, 1H), 4.18–4.22 (m, 1H), 4.35 (ddt, *J* = 6.1, 4.2, 2.3 Hz, 1H), 4.41 (dd, *J* = 15.7, 6.5 Hz, 1H), 4.46–4.50 (m, 1H), 4.52 (d, *J* = 8.9 Hz, 1H), 5.15 (d, *J* = 3.6 Hz, 1H), 7.14–7.20 (m, 2H), 7.33 (dd, *J* = 5.3, 3.6 Hz, 2H), 8.59 (t, *J* = 6.0 Hz, 1H), 9.04 (s, 1H); ^13^C NMR (151 MHz, DMSO-*d*_6_): δ 13.6, 15.2, 16.6, 16.8, 19.7, 26.1, 36.3, 37.8, 41.7, 56.6, 57.3, 58.90, 68.9, 120.0, 124.5, 128.7, 128.9, 129.6, 130.8, 136.9, 140.1, 149.0, 152.2, 164.3, 168.6, 171.6; LC–MS (ESI) (90% H_2_O to 100% MeCN in 10 min, then 100% MeCN to 15 min, DAD 200–600 nm), *t*_R_ = 5.68 min, 95% purity, *m*/*z* calcd for C_28_H_35_N_5_O_4_S [M + H]^+^, 538.25; found, 538.3. HRMS (ESI) *m*/*z*: calcd for C_28_H_35_N_5_O_4_S [M + H]^+^, 538.2486; found, 538.2482.

##### (2*S*,4*R*)-1-((*S*)-2-(1-Cyanocyclopropane-1-carboxamido)-3,3-dimethylbutanoyl)-4-hydroxy-*N*-(3-methoxy-4-(4-methylthiazol-5-yl)benzyl)pyrrolidine-2-carboxamide (**7**)

Following general procedure C, compound **7** was obtained using Boc-protected amine **42g** (type **42**, R = 3-OMe; 100 mg, 0.3 mmol) and acid **46** (101 mg, 0.3 mmol). The residue was purified by flash column chromatography using a gradient from 0 to 10% MeOH in CH_2_Cl_2_ to afford **7** as a white solid. Yield: 17 mg (10%); mp 105–106 °C; *R*_f_ = 0.25 (CH_2_Cl_2_/MeOH 9:1); ^1^H NMR (600 MHz, DMSO-*d*_6_): δ 0.95 (s, 9H), 1.45–1.53 (m, 2H), 1.59–1.67 (m, 2H), 1.87–1.94 (m, 1H), 2.06–2.12 (m, 1H), 2.27 (s, 3H), 3.57 (d, *J* = 10.9 Hz, 1H), 3.64 (dd, *J* = 10.9, 3.8 Hz, 1H), 3.84 (s, 3H), 4.19 (dd, *J* = 15.8, 5.1 Hz, 1H), 4.33–4.38 (m, 1H), 4.46–4.53 (m, 3H), 5.16 (d, *J* = 3.6 Hz, 1H), 6.93–6.97 (m, 1H), 7.14–7.17 (m, 1H), 7.22 (d, *J* = 7.7 Hz, 1H), 7.28 (d, *J* = 8.9 Hz, 1H), 8.64 (dd, *J* = 6.9, 5.1 Hz, 1H), 8.98 (s, 1H); ^13^C NMR (151 MHz, DMSO-*d*_6_): δ 13.6, 15.9, 16.6, 16.8, 26.1, 36.4, 37.8, 41.8, 55.6, 56.7, 57.4, 59.0, 68.9, 110.2, 117.9, 118.9, 120.1, 126.7, 131.1, 142.0, 149.3, 151.9, 156.6, 164.3, 168.7, 171.5; LC–MS (ESI) (90% H_2_O to 100% MeOH in 10 min, then 100% MeOH to 20 min, DAD 220–400 nm), *t*_R_ = 10.64 min, 99% purity, *m*/*z* calcd for C_28_H_35_N_5_O_5_S [M + H]^+^, 554.24; found, 554.5. HRMS (ESI) *m*/*z*: calcd for C_28_H_35_N_5_O_5_S [M + H]^+^, 554.2432; found, 554.2430.

##### (2*S*,4*R*)-1-((*S*)-2-(1-Cyanocyclopropane-1-carboxamido)-3,3-dimethylbutanoyl)-*N*-(3-fluoro-4-(4-methylthiazol-5-yl)benzyl)-4-hydroxypyrrolidine-2-carboxamide (**8**)

Following general procedure C, compound **8** was obtained using Boc-protected amine **42h** (type **42**, R = 3-F; 97 mg, 0.3 mmol) and acid **46** (101 mg, 0.3 mmol). The residue was purified by flash column chromatography using a gradient from 0 to 10% MeOH in CH_2_Cl_2_ to afford **8** as a white solid. Yield: 23 mg (17%); mp 98–100 °C; *R*_f_ = 0.38 (CH_2_Cl_2_/MeOH 9:1); ^1^H NMR (600 MHz, DMSO-*d*_6_): δ 0.95 (s, 9H), 1.46–1.53 (m, 2H), 1.58–1.66 (m, 2H), 1.88–1.94 (m, 1H), 2.05–2.11 (m, 1H), 2.32 (d, *J* = 1.1 Hz, 3H), 3.56–3.59 (m, 1H), 3.64 (dd, *J* = 10.8, 3.8 Hz, 1H), 4.24 (dd, *J* = 16.1, 5.6 Hz, 1H), 4.34–4.37 (m, 1H), 4.43–4.54 (m, 3H), 5.16 (d, *J* = 3.6 Hz, 1H), 7.22–7.24 (m, 1H), 7.31–7.36 (m, 2H), 7.40 (t, *J* = 7.8 Hz, 1H), 8.70 (t, *J* = 6.1 Hz, 1H), 9.09 (s, 1H); ^13^C NMR (151 MHz, DMSO-*d*_6_): δ 13.7, 15.7, 16.6, 16.8, 26.0, 36.2, 37.8, 41.4, 56.6, 57.3, 58.9, 68.9, 114.4 (d, ^2^*J*_F,C_ = 23.0 Hz), 116.9 (d, ^2^*J*_F,C_ = 15.3 Hz), 120.1, 123.1 (d, ^3^*J*_F,C_ = 2.9 Hz), 123.8, 131.7 (d, ^4^*J*_F,C_ = 2.3 Hz), 143.2 (d, ^3^*J*_F,C_ = 7.6 Hz), 150.1, 153.1, 158.9 (d, ^1^*J*_F,C_ = 246.6 Hz), 164.4, 168.7, 171.9; LC–MS (ESI) (90% H_2_O to 100% MeCN in 10 min, then 100% MeCN to 15 min, DAD 200–600 nm), *t*_R_ = 8.28 min, 97% purity, *m*/*z* calcd for C_27_H_23_FN_5_O_4_S [M + H]^+^, 542.22; found, 542.4. HRMS (ESI) *m*/*z*: calcd for C_27_H_23_FN_5_O_4_S [M + H]^+^, 542.2232; found, 542.2233.

##### (2*S*,4*R*)-*N*-(3-Chloro-4-(4-methylthiazol-5-yl)benzyl)-1-((*S*)-2-(1-cyanocyclopropane-1-carboxamido)-3,3-dimethylbutanoyl)-4-hydroxypyrrolidine-2-carboxamide (**9**)

Following general procedure C, compound **9** was obtained using Boc-protected amine **42i** (type **42**, R = 3-Cl; 135 mg, 0.4 mmol) and acid **46** (135 mg, 0.4 mmol). The residue was purified by flash column chromatography using a gradient from 0 to 10% MeOH in CH_2_Cl_2_ to afford **9** as a white solid. Yield: 12 mg (5%); mp 157–159 °C; *R*_f_ = 0.44 (CH_2_Cl_2_/MeOH 9:1); ^1^H NMR (600 MHz, DMSO-*d*_6_): δ 0.95 (s, 9H), 1.46–1.54 (m, 2H), 1.58–1.66 (m, 2H), 1.90 (ddd, *J* = 13.1, 9.1, 4.4 Hz, 1H), 2.05–2.11 (m, 1H), 2.23 (s, 3H), 3.54–3.58 (m, 1H), 3.64 (dd, *J* = 10.8, 3.8 Hz, 1H), 4.24 (dd, *J* = 16.0, 5.4 Hz, 1H), 4.33–4.38 (m, 1H), 4.43–4.53 (m, 3H), 5.15 (d, *J* = 3.6 Hz, 1H), 7.31–7.40 (m, 3H), 7.60 (d, *J* = 1.6 Hz, 1H), 8.69 (t, *J* = 6.1 Hz, 1H), 9.09 (s, 1H); ^13^C NMR (151 MHz, DMSO-*d*_6_): δ 13.6, 15.5, 16.6, 16.8, 26.1, 36.3, 37.8, 41.3, 56.6, 57.3, 58.9, 68.9, 120.0, 125.9, 127.3, 128.0, 128.2, 132.4, 133.3, 142.6, 150.2, 152.9, 164.3, 168.7, 171.8; LC–MS (ESI) (90% H_2_O to 100% MeOH in 10 min, then 100% MeOH to 20 min, DAD 220–400 nm), *t*_R_ = 10.64 min, 98% purity, *m*/*z* calcd for C_27_H_32_ClN_5_O_4_S [M + H]^+^, 558.19; found, 558.3. HRMS (ESI) *m*/*z*: calcd for C_27_H_32_ClN_5_O_4_S [M + H]^+^, 558.1936; found, 558.1938.

##### (2*S*,4*R*)-1-((*S*)-2-(1-Cyanocyclopropane-1-carboxamido)-3,3-dimethylbutanoyl)-*N*-(2,6-dimethyl-4-(4-methylthiazol-5-yl)benzyl)-4-hydroxypyrrolidine-2-carboxamide (**10**)

Following general procedure C, compound **10** was obtained using Boc-protected amine **42j** (type **42**, R = 2-Me, 6-Me; 100 mg, 0.3 mmol) and acid **46** (101 mg, 0.3 mmol). The residue was purified by flash column chromatography using a gradient from 0 to 10% MeOH in CH_2_Cl_2_ to afford **10** as a white solid. Yield: 28 mg (17%); mp 106–110 °C; *R*_f_ = 0.42 (CH_2_Cl_2_/MeOH 9:1); ^1^H NMR (600 MHz, DMSO-*d*_6_): δ 0.96 (s, 9H), 1.44–1.52 (m, 2H), 1.56–1.66 (m, 2H), 1.82–1.89 (m, 1H), 1.96–2.03 (m, 1H), 2.35 (s, 6H), 2.46 (s, 3H), 3.51–3.56 (m, 1H), 3.64 (dd, *J* = 10.8, 4.0 Hz, 1H), 4.24 (dd, *J* = 14.0, 4.5 Hz, 1H), 4.30–4.34 (m, 1H), 4.38–4.42 (m, 2H), 4.51 (d, *J* = 8.9 Hz, 1H), 5.10 (d, *J* = 3.6 Hz, 1H), 7.16 (s, 2H), 7.28 (d, *J* = 8.9 Hz, 1H), 8.08 (t, *J* = 5.0 Hz, 1H), 8.97 (s, 1H); ^13^C NMR (151 MHz, DMSO-*d*_6_): δ 13.7, 16.0, 16.6, 16.8, 19.3, 26.1, 36.2, 36.7, 37.9, 56.6, 57.3, 58.6, 68.8, 120.1, 128.3, 130.3, 131.1, 134.7, 138.2, 147.7, 151.4, 164.4, 168.5, 170.9; LC–MS (ESI) (90% H_2_O to 100% MeOH in 10 min, then 100% MeOH to 20 min, DAD 220–400 nm), *t*_R_ = 11.23 min, 99% purity, *m*/*z* calcd for C_29_H_37_N_5_O_4_S [M + H]^+^, 552.26; found, 552.6. HRMS (ESI) *m*/*z*: calcd C_29_H_37_N_5_O_4_S [M + H]^+^, 552.2639; found, 552.2634.

##### (2*S*,4*R*)-1-((*S*)-2-(1-Cyanocyclopropane-1-carboxamido)-3,3-dimethylbutanoyl)-*N*-(2,6-dimethoxy-4-(4-methylthiazol-5-yl)benzyl)-4-hydroxypyrrolidine-2-carboxamide (**11**)

Following general procedure C, compound **11** was obtained using Boc-protected amine **42k** (type **42**, R = 2-OMe, 6-OMe; 109 mg, 0.3 mmol) and acid **46** (101 mg, 0.3 mmol). The residue was purified by flash column chromatography using a gradient from 0 to 10% MeOH in CH_2_Cl_2_ to afford **11** as a yellow solid. Yield: 90 mg (51%); mp 102–104 °C; *R*_f_ = 0.42 (CH_2_Cl_2_/MeOH 9:1); ^1^H NMR (500 MHz, DMSO-*d*_6_): δ 0.94 (s, 9H), 1.43–1.52 (m, 2H), 1.55–1.67 (m, 2H), 1.84–1.99 (m, 2H), 3.51 (d, *J* = 9.7 Hz, 1H), 3.61 (dd, *J* = 10.8, 4.3 Hz, 1H), 3.82 (s, 6H), 4.20 (dd, *J* = 13.1, 3.8 Hz, 1H), 4.27–4.32 (m, 1H), 4.35 (dd, *J* = 13.1, 5.6 Hz, 1H), 4.44 (t, *J* = 7.9 Hz, 1H), 4.50 (d, *J* = 8.9 Hz, 1H), 5.06 (d, *J* = 3.8 Hz, 1H), 6.72 (s, 2H), 7.26 (d, *J* = 8.9 Hz, 1H), 7.65 (t, *J* = 4.7 Hz, 1H), 9.00 (s, 1H); ^13^C NMR (126 MHz, DMSO-*d*_6_): δ 13.6, 16.0, 16.6, 16.7, 26.0, 31.4, 36.1, 37.5, 56.0, 57.3, 58.4, 68.7, 105.1, 113.5, 120.0, 131.4, 132.2, 148.2, 151.6, 158.5, 164.3, 168.6, 170.5; LC–MS (ESI) (90% H_2_O to 100% MeOH in 10 min, then 100% MeOH to 20 min, DAD 220–400 nm), *t*_R_ = 10.91 min, 96% purity, *m*/*z* calcd for C_29_H_37_N_5_O_4_S [M + H]^+^, 584.25; found, 584.7. HRMS (ESI) *m*/*z*: calcd for C_29_H_37_N_5_O_4_S [M + H]^+^, 584.2537; found, 584.2531.

##### (2*S*,4*R*)-1-((*S*)-2-(1-Cyanocyclopropane-1-carboxamido)-3,3-dimethylbutanoyl)-*N*-(2,6-difluoro-4-(4-methylthiazol-5-yl)benzyl)-4-hydroxypyrrolidine-2-carboxamide (**12**)

Following general procedure C, compound **12** was obtained using Boc-protected amine **42L** (type **42**, R = 2-F, 6-F; 102 mg, 0.3 mmol) and acid **46** (101 mg, 0.3 mmol). The residue was purified by flash column chromatography using a gradient from 0 to 10% MeOH in CH_2_Cl_2_ to afford **12** as a white solid. Yield: 83 mg (49%); mp 84–86 °C; *R*_f_ = 0.42 (CH_2_Cl_2_/MeOH 9:1); ^1^H NMR (500 MHz, DMSO-*d*_6_): δ 0.92 (s, 9H), 1.43–1.53 (m, 2H), 1.56–1.67 (m, 2H), 1.79–1.87 (m, 1H), 1.94–2.01 (m, 1H), 2.48 (s, 3H), 3.52 (d, *J* = 10.8 Hz, 1H), 3.61 (dd, *J* = 10.8, 4.0 Hz, 1H), 4.23–4.32 (m, 2H), 4.37–4.46 (m, 2H), 4.49 (d, *J* = 8.9 Hz, 1H), 5.09 (d, *J* = 3.7 Hz, 1H), 7.21–7.29 (m, 3H), 8.38 (t, *J* = 5.3 Hz, 1H), 9.06 (s, 1H); ^13^C NMR (126 MHz, DMSO-*d*_6_): δ 13.6, 16.0, 16.6, 16.7, 26.1, 30.4, 36.1, 37.6, 56.5, 57.3, 58.5, 68.7, 111.9 (d, ^2^*J*_F,C_ = 14.1 Hz), 111.9 (d, ^2^*J*_F,C_ = 27.3 Hz), 113.6 (t, ^2^*J*_F,C_ = 19.8 Hz), 120.0, 128.8, 133.2 (t, ^3^*J*_F,C_ = 11.0 Hz), 149.3, 152.6, 160.9 (dd, ^1^*J*_F,C_ = 248.7 Hz, ^3^*J*_F,C_ = 9.7 Hz), 164.3, 168.5, 170.9; LC–MS (ESI) (90% H_2_O to 100% MeOH in 10 min, then 100% MeOH to 20 min, DAD 220–400 nm), *t*_R_ = 10.73 min, 97% purity, *m*/*z* calcd for C_27_H_31_F_2_N_5_O_4_S [M + H]^+^, 560.21; found, 560.4. HRMS (ESI) *m*/*z*: calcd for C_27_H_31_F_2_N_5_O_4_S [M + H]^+^, 560.2138; found, 560.2135.

##### (2*S*,4*R*)-1-((*S*)-2-(1-Cyanocyclopropane-1-carboxamido)-3,3-dimethylbutanoyl)-*N*-(2,6-dichloro-4-(4-methylthiazol-5-yl)benzyl)-4-hydroxypyrrolidine-2-carboxamide (**13**)

Following general procedure C, compound **13** was obtained using Boc-protected amine **42m** (type **42**, R = 2-Cl, 6-Cl; 112 mg, 0.3 mmol) and acid **46** (101 mg, 0.3 mmol). The residue was purified by flash column chromatography using a gradient from 0 to 10% MeOH in CH_2_Cl_2_ to afford **13** as a white solid. Yield: 66 mg (37%); mp 138–140 °C; *R*_f_ = 0.49 (CH_2_Cl_2_/MeOH 9:1); ^1^H NMR (500 MHz, DMSO-*d*_6_): δ 0.96 (s, 9H), 1.45–1.53 (m, 2H), 1.57–1.65 (m, 2H), 1.85–1.92 (m, 1H), 1.96–2.03 (m, 1H), 2.48 (s, 3H), 3.51–3.55 (m, 1H), 3.63 (dd, *J* = 10.7, 4.1 Hz, 1H), 4.29–4.34 (m, 1H), 4.41–4.52 (m, 3H), 4.63 (dd, *J* = 13.8, 5.8 Hz, 1H), 5.09 (d, *J* = 3.7 Hz, 1H), 7.27 (d, *J* = 8.8 Hz, 1H), 7.61 (s, 2H), 8.20 (dd, *J* = 5.7, 3.7 Hz, 1H), 9.08 (s, 1H); ^13^C NMR (126 MHz, DMSO-*d*_6_): δ 13.6, 15.9, 16.6, 16.8, 26.1, 36.1, 37.8, 38.4, 56.5, 57.3, 58.5, 68.7, 120.0, 128.0, 128.4, 132.9, 133.5, 135.9, 149.6, 152.9, 164.3, 168.5, 170.8; LC–MS (ESI) (90% H_2_O to 100% MeOH in 10 min, then 100% MeOH to 20 min, DAD 220–400 nm), *t*_R_ = 11.47 min, 100% purity, *m*/*z* calcd for C_27_H_32_Cl_2_N_5_O_4_S [M + H]^+^, 592.15; found, 592.2. HRMS (ESI) *m*/*z*: calcd for C_27_H_32_Cl_2_N_5_O_4_S [M + H]^+^, 592.1547; found, 592.1540.

##### (2*S*,4*R*)-1-((*S*)-2-(1-Cyanocyclopropane-1-carboxamido)-3,3-dimethylbutanoyl)-*N*-(2,5-dimethyl-4-(4-methylthiazol-5-yl)benzyl)-4-hydroxypyrrolidine-2-carboxamide (**14**)

Following general procedure C, compound **14** was obtained using Boc-protected amine **42n** (type **42**, R = 2-Me, 5-Me; 98 mg, 0.3 mmol) and acid **46** (101 mg, 0.3 mmol). The residue was purified by flash column chromatography using a gradient from 0 to 10% MeOH in CH_2_Cl_2_ to afford **14** as a white solid. Yield: 78 mg (47%); mp 96–98 °C; *R*_f_ = 0.47 (CH_2_Cl_2_/MeOH 9:1); ^1^H NMR (500 MHz, DMSO-*d*_6_): δ 0.95 (s, 9H), 1.46–1.53 (m, 2H), 1.58–1.67 (m, 2H), 1.91 (ddd, *J* = 13.2, 9.0, 4.5 Hz, 1H), 2.05–2.08 (m, 1H, 3-H), 2.09 (s, 3H, CH_3_), 2.17 (s, 3H, CH_3_), 2.22 (s, 3H), 3.56 (d, *J* = 10.7 Hz, 1H), 3.64 (dd, *J* = 10.8, 3.9 Hz, 1H), 4.15 (dd, *J* = 15.6, 5.2 Hz, 1H), 4.32–4.38 (m, 2H), 4.48–4.53 (m, 2H), 5.13 (d, *J* = 3.6 Hz, 1H), 7.04 (s, 1H, Ar-H), 7.32 (d, *J* = 8.8 Hz, 1H), 7.36 (s, 1H), 8.47 (t, *J* = 5.8 Hz, 1H), 9.02 (s, 1H); ^13^C NMR (126 MHz, DMSO-*d*_6_): δ 13.6, 15.2, 16.6, 16.8, 17.8, 19.1, 26.0, 36.3, 37.9, 56.6, 57.3, 58.9, 68.9, 120.0, 128.6, 129.3, 129.6, 132.1, 132.9, 134.1, 137.5, 148.8, 152.0, 164.3, 168.6, 171.4; LC–MS (ESI) (90% H_2_O to 100% MeCN in 10 min, then 100% MeCN to 15 min, DAD 200–600 nm), *t*_R_ = 5.92 min, 97% purity, *m*/*z* calcd for C_29_H_37_N_5_O_4_S [M + H]^+^, 552.26; found, 552.5. HRMS (ESI) *m*/*z*: calcd for C_29_H_37_N_5_O_4_S [M + H]^+^, 552.2639; found, 552.2637.

##### (2*S*,4*R*)-1-((*S*)-2-(1-Cyanocyclopropane-1-carboxamido)-3,3-dimethylbutanoyl)-*N*-(2,5-dimethoxy-4-(4-methylthiazol-5-yl)benzyl)-4-hydroxypyrrolidine-2-carboxamide (**15**)

Following general procedure C, compound **15** was obtained using Boc-protected amine **42o** (type **42**, R = 2-OMe, 5-OMe; 110 mg, 0.3 mmol) and acid **46** (101 mg, 0.3 mmol). The residue was purified by flash column chromatography using a gradient from 0 to 10% MeOH in CH_2_Cl_2_ to afford **15** as a white solid. Yield: 52 mg (30%); mp 104–106 °C; *R*_f_ = 0.38 (CH_2_Cl_2_/MeOH 9:1); ^1^H NMR (500 MHz, DMSO-*d*_6_): δ 0.93 (s, 9H), 1.46–1.52 (m, 2H), 1.59–1.67 (m, 2H), 1.88–1.95 (m, 1H), 2.07–2.13 (m, 1H), 2.31 (s, 3H), 3.57 (d, *J* = 10.9 Hz, 1H), 3.64 (dd, *J* = 10.9, 3.7 Hz, 1H), 3.78 (s, 3H), 3.83 (s, 3H), 4.13 (dd, *J* = 16.5, 5.0 Hz, 1H), 4.34–4.37 (m, 1H), 4.41 (dd, *J* = 16.6, 7.0 Hz, 1H), 4.49–4.55 (m, 2H), 5.15 (d, *J* = 3.6 Hz, 1H), 6.90 (s, 1H), 7.23 (s, 1H), 7.25 (d, *J* = 8.9 Hz, 1H), 8.55 (dd, *J* = 7.0, 5.1 Hz, 1H), 8.98 (s, 1H); ^13^C NMR (126 MHz, DMSO-*d*_6_): δ 13.5, 15.9, 16.6, 16.9, 26.0, 36.4, 36.9, 37.8, 55.9, 56.2, 56.7, 57.4, 59.1, 68.9, 111.7, 113.6, 118.0, 120.0, 126.8, 128.9, 149.4, 149.9, 150.6, 151.9, 164.2, 168.7, 171.6; LC–MS (ESI) (90% H_2_O to 100% MeOH in 10 min, then 100% MeOH to 20 min, DAD 220–400 nm), *t*_R_ = 10.92 min, 98% purity, *m*/*z* calcd for C_29_H_37_N_5_O_6_S [M + H]^+^, 584.25; found, 584.5. HRMS (ESI) *m*/*z*: calcd for C_29_H_37_N_5_O_6_S [M + H]^+^, 584.2537; found, 584.2531.

##### (2*S*,4*R*)-1-((*S*)-2-(1-Cyanocyclopropane-1-carboxamido)-3,3-dimethylbutanoyl)-*N*-(2,5-difluoro-4-(4-methylthiazol-5-yl)benzyl)-4-hydroxypyrrolidine-2-carboxamide (**16**)

Following general procedure C, compound **16** was obtained using Boc-protected amine **42p** (type **42**, R = 2-F, 5-F; 102 mg, 0.3 mmol) and acid **46** (101 mg, 0.3 mmol). The residue was purified by flash column chromatography using a gradient from 0 to 10% MeOH in CH_2_Cl_2_ to afford **16** as a white solid. Yield: 79 mg (47%); mp 129–130 °C; *R*_f_ = 0.50 (CH_2_Cl_2_/MeOH 9:1); ^1^H NMR (600 MHz, DMSO-*d*_6_): δ 0.93 (s, 9H), 1.46–1.53 (m, 2H), 1.58–1.66 (m, 2H), 1.87–1.93 (m, 1H), 2.05–2.10 (m, 1H), 2.34 (d, *J* = 1.0 Hz, 3H), 3.55–3.59 (m, 1H), 3.64 (dd, *J* = 10.8, 3.9 Hz, 1H), 4.23 (dd, *J* = 16.3, 5.3 Hz, 1H), 4.34–4.38 (m, 1H), 4.41–4.53 (m, 3H), 5.17 (d, *J* = 3.6 Hz, 1H), 7.35 (d, *J* = 8.9 Hz, 1H), 7.38 (dd, *J* = 9.9, 6.0 Hz, 1H), 7.49 (dd, *J* = 10.4, 6.2 Hz, 1H), 8.76 (t, *J* = 6.0 Hz, 1H), 9.12 (s, 1H); ^13^C NMR (151 MHz, DMSO-*d*_6_): δ 13.7, 15.7 (d, ^5^*J*_F,C_ = 2.0 Hz), 16.6, 16.8, 26.0, 35.9 (d, ^3^*J*_F,C_ = 3.6 Hz), 36.2, 37.7, 56.6, 57.3, 58.9, 68.9, 116.1 (d, ^3^*J*_F,C_ = 5.1 Hz), 116.3 (d, ^3^*J*_F,C_ = 4.9 Hz), 117.7 (dd, *J* = 25.1, 2.2 Hz), 118.4 (dd, ^2^*J*_F,C_ = 18.2 Hz, ^3^*J*_F,C_ = 9.1 Hz), 120.1, 122.7, 129.0 (dd, ^2^*J*_F,C_ = 17.2 Hz, ^3^*J*_F,C_ = 7.9 Hz), 150.7, 153.6, 155.2 (dd, ^1^*J*_F,C_ = 242.5 Hz, ^2^*J*_F,C_ = 25.9 Hz), 164.4, 168.8, 172.2; LC–MS (ESI) (90% H_2_O to 100% MeOH in 10 min, then 100% MeOH to 20 min, DAD 220–400 nm), *t*_R_ = 10.78 min, 100% purity, *m*/*z* calcd for C_27_H_31_F_2_N_5_O_4_S [M + H]^+^, 560.21; found, 560.4. HRMS (ESI) *m*/*z*: calcd for C_27_H_31_F_2_N_5_O_4_S [M + H]^+^, 560.2138 found, 560.2134.

##### (2*S*,4*R*)-1-((*S*)-2-(1-Cyanocyclopropane-1-carboxamido)-3,3-dimethylbutanoyl)-*N*-(2,5-dichloro-4-(4-methylthiazol-5-yl)benzyl)-4-hydroxypyrrolidine-2-carboxamide (**17**)

Following general procedure C, compound **17** was obtained using Boc-protected amine **42q** (type **42**, R = 2-Cl, 5-Cl; 110 mg, 0.3 mmol) and acid **46** (101 mg, 0.3 mmol). The residue was purified by flash column chromatography using a gradient from 0 to 10% MeOH in CH_2_Cl_2_ to afford **17** as a white solid. Yield: 59 mg (33%); mp 188–190 °C; *R*_f_ = 0.38 (CH_2_Cl_2_/MeOH 9:1); ^1^H NMR (600 MHz, DMSO-*d*_6_): δ 0.93 (s, 9H), 1.45–1.52 (m, 2H), 1.58–1.66 (m, 2H), 1.87–1.94 (m, 1H), 2.06–2.13 (m, 1H), 2.24 (s, 3H), 3.57 (d, *J* = 10.8 Hz, 1H), 3.64 (dd, *J* = 10.9, 3.7 Hz, 1H), 4.22 (dd, *J* = 16.7, 5.2 Hz, 1H), 4.34–4.39 (m, 1H), 4.44–4.54 (m, 3H), 5.18 (d, *J* = 3.5 Hz, 1H), 7.33 (d, *J* = 8.9 Hz, 1H), 7.59 (s, 1H), 7.87 (s, 1H), 8.82 (dd, *J* = 6.8, 5.3 Hz, 1H), 9.12 (s, 1H); ^13^C NMR (151 MHz, DMSO-*d*_6_): δ 13.6, 15.4, 16.6, 16.8, 26.1, 36.4, 37.7, 56.6, 57.3, 59.1, 69.0, 119.9, 126.0, 129.5, 129.9, 130.1, 132.4, 132.5, 139.0, 150.7, 153.4, 164.3, 168.7, 172.2; LC–MS (ESI) (90% H_2_O to 100% MeCN in 10 min, then 100% MeCN to 15 min, DAD 200–600 nm), *t*_R_ = 6.10 min, 98% purity, *m*/*z* calcd for C_27_H_31_Cl_2_N_5_O_4_S [M + H]^+^, 592.15; found, 592.3. HRMS (ESI) *m*/*z*: calcd for C_27_H_31_Cl_2_N_5_O_4_S [M + H]^+^, 592.1547; found, 592.1536.

##### (2*S*,4*R*)-1-((*S*)-2-(1-Cyanocyclopropane-1-carboxamido)-3,3-dimethylbutanoyl)-*N*-(2,3-dimethyl-4-(4-methylthiazol-5-yl)benzyl)-4-hydroxypyrrolidine-2-carboxamide (**18**)

Following general procedure C, compound **18** was obtained using Boc-protected amine **42r** (type **42**, R = 2-Me, 3-Me; 98 mg, 0.3 mmol) and acid **46** (101 mg, 0.3 mmol). The residue was purified by flash column chromatography using a gradient from 0 to 10% MeOH in CH_2_Cl_2_ to afford **18** as a white solid. Yield: 60 mg (36%); mp 131–132 °C; *R*_f_ = 0.58 (CH_2_Cl_2_/MeOH 9:1); ^1^H NMR (500 MHz, DMSO-*d*_6_): δ 0.95 (s, 9H), 1.43–1.54 (m, 2H), 1.58–1.66 (m, 2H), 1.87–1.94 (m, 1H), 2.03–2.09 (m, 4H), 2.15 (s, 3H), 2.20 (s, 3H), 3.56 (d, *J* = 10.8 Hz, 1H), 3.64 (dd, *J* = 10.8, 3.9 Hz, 1H), 4.28 (dd, *J* = 15.4, 5.5 Hz, 1H), 4.31–4.38 (m, 2H), 4.50 (dd, *J* = 16.1, 8.4 Hz, 2H), 5.12 (d, *J* = 3.6 Hz, 1H), 7.02 (d, *J* = 7.9 Hz, 1H), 7.24 (d, *J* = 7.9 Hz, 1H), 7.32 (d, *J* = 8.8 Hz, 1H), 8.43 (t, *J* = 5.8 Hz, 1H), 9.03 (s, 1H); ^13^C NMR (126 MHz, DMSO-*d*_6_): δ 13.6, 14.9, 15.1, 16.5, 16.7, 16.8, 26.0, 36.2, 37.9, 41.1, 56.6, 57.3, 58.7, 68.8, 120.0, 125.3, 128.0, 129.1, 130.7, 135.2, 135.8, 137.4, 148.9, 152.0, 164.3, 168.6, 171.2; LC–MS (ESI) (90% H_2_O to 100% MeCN in 10 min, then 100% MeCN to 15 min, DAD 200–600 nm), *t*_R_ = 5.96 min, 100% purity, *m*/*z* calcd for C_29_H_37_N_5_O_4_S [M + H]^+^, 552.26; found, 552.4. HRMS (ESI) *m*/*z*: calcd for C_29_H_37_N_5_O_4_S [M + H]^+^, 552.2639; found, 552.2638.

##### (2*S*,4*R*)-1-((*S*)-2-(1-Cyanocyclopropane-1-carboxamido)-3,3-dimethylbutanoyl)-*N*-(2,3-difluoro-4-(4-methylthiazol-5-yl)benzyl)-4-hydroxypyrrolidine-2-carboxamide (**19**)

Following general procedure C, compound **19** was obtained using Boc-protected amine **42s** (type **42**, R = 2-F, 3-F; 102 mg, 0.3 mmol) and acid **46** (101 mg, 0.3 mmol). The residue was purified by flash column chromatography using a gradient from 0 to 10% MeOH in CH_2_Cl_2_ to afford **19** as a white solid. Yield: 57 mg (34%); mp 159–162 °C; *R*_f_ = 0.58 (CH_2_Cl_2_/MeOH 9:1); ^1^H NMR (600 MHz, DMSO-*d*_6_ δ 0.93 (s, 9H), 1.45–1.54 (m, 2H), 1.58–1.66 (m, 2H), 1.86–1.93 (m, 1H), 2.04–2.10 (m, 1H), 2.35 (s, 3H), 3.54–3.58 (m, 1H), 3.63 (dd, *J* = 10.8, 3.9 Hz, 1H), 4.30–4.39 (m, 2H), 4.42 (dd, *J* = 15.9, 6.1 Hz, 1H), 4.45–4.54 (m, 2H), 5.15 (d, *J* = 3.6 Hz, 1H), 7.17–7.25 (m, 1H), 7.30–7.39 (m, 2H), 8.70 (t, *J* = 6.0 Hz, 1H), 9.14 (s, 1H); ^13^C NMR (151 MHz, DMSO-*d*_6_): δ 13.7, 15.7, 16.6, 16.8, 26.0, 35.9, 36.1, 37.7, 56.6, 57.3, 58.7, 68.9, 119.4 (d, ^2^*J*_F,C_ = 11.7 Hz), 120.1, 122.6 (d, ^3^*J*_F,C_ = 2.3 Hz), 124.1 (d, ^3^*J*_F,C_ = 3.6 Hz), 126.0 (d, ^3^*J*_F,C_ = 2.9 Hz), 129.0 (d, ^2^*J*_F,C_ = 11.8 Hz), 146.5 (dd, ^1^*J*_F,C_ = 189.9 Hz, ^2^*J*_F,C_ = 13.2 Hz), 148.1 (dd, ^1^*J*_F,C_ = 189.2 Hz, ^2^*J*_F,C_ = 13.3 Hz), 150.7, 153.8, 164.4, 168.7, 171.9; LC–MS (ESI) (90% H_2_O to 100% MeCN in 10 min, then 100% MeCN to 15 min, DAD 200–600 nm), *t*_R_ = 5.63 min, 100% purity, *m*/*z* calcd for C_27_H_31_F_2_N_5_O_4_S [M + H]^+^, 560.21; found, 560.5. HRMS (ESI) *m*/*z*: calcd for C_27_H_31_F_2_N_5_O_4_S [M + H]^+^, 560.2138; found, 560.2130.

##### (2*S*,4*R*)-1-((*S*)-2-(1-Cyanocyclopropane-1-carboxamido)-3,3-dimethylbutanoyl)-*N*-(3-fluoro-2-hydroxy-4-(4-methylthiazol-5-yl)benzyl)-4-hydroxypyrrolidine-2-carboxamide (**20**)

Following general procedure C, compound **20** was obtained using Boc-protected amine **42t** (type **42**, R = 2-OH, 3-F; 135 mg, 0.4 mmol) and acid **46** (135 mg, 0.4 mmol). The residue was purified by flash column chromatography using a gradient from 0 to 10% MeOH in CH_2_Cl_2_ to afford **20** as a white solid. Yield: 33 mg (15%); mp 152–154 °C; *R*_f_ = 0.53 (CH_2_Cl_2_/MeOH 9:1); ^1^H NMR (600 MHz, DMSO-*d*_6_): δ 0.93 (s, 9H), 1.45–1.53 (m, 2H), 1.57–1.65 (m, 2H), 1.86–1.94 (m, 1H), 2.02–2.09 (m, 1H), 2.32 (s, 3H), 3.52–3.58 (m, 1H), 3.62 (dd, *J* = 10.8, 3.9 Hz, 1H), 4.20–4.29 (m, 2H), 4.31–4.36 (m, 1H), 4.45–4.53 (m, 2H), 5.14 (d, *J* = 3.7 Hz, 1H), 6.78 (dd, *J* = 7.9, 6.6 Hz, 1H), 7.14 (d, *J* = 8.0 Hz, 1H), 7.32 (d, *J* = 8.9 Hz, 1H), 8.64 (t, *J* = 6.1 Hz, 1H), 9.07 (s, 1H), 9.95 (s, 1H); ^13^C NMR (151 MHz, DMSO-*d*_6_): δ 13.7, 15.8, 16.6, 16.8, 26.0, 36.2, 37.4, 37.8, 56.6, 57.3, 58.7, 68.9, 117.8 (d, ^2^*J*_F,C_ = 13.2 Hz), 120.1, 120.7, 123.3 (d, ^3^*J*_F,C_ = 2.6 Hz), 124.2, 129.3 (d, ^3^*J*_F,C_ = 1.7 Hz), 142.6 (d, ^2^*J*_F,C_ = 14.4 Hz), 148.3 (d, ^1^*J*_F,C_ = 241.3 Hz), 149.9, 152.9, 164.4, 168.7, 172.2; LC–MS (ESI) (90% H_2_O to 100% MeCN in 10 min, then 100% MeCN to 15 min, DAD 200–600 nm), *t*_R_ = 5.34 min, 95% purity, *m*/*z* calcd for C_27_H_32_FN_5_O_5_S [M + H]^+^, 558.22; found, 558.4. HRMS (ESI) *m*/*z*: calcd for C_27_H_32_FN_5_O_5_S [M + H]^+^, 558.2181; found, 558.2176.

##### (2*S*,4*R*)-1-((*S*)-2-(1-Cyanocyclopropane-1-carboxamido)-3,3-dimethylbutanoyl)-*N*-(3-fluoro-2-methoxy-4-(4-methylthiazol-5-yl)benzyl)-4-hydroxypyrrolidine-2-carboxamide (**21**)

Following general procedure C, compound **21** was obtained using Boc-protected amine **42u** (type **42**, R = 2-OMe, 3-F; 105 mg, 0.3 mmol) and acid **46** (101 mg, 0.3 mmol). The residue was purified by flash column chromatography using a gradient from 0 to 10% MeOH in CH_2_Cl_2_ to afford **21** as a pale orange solid. Yield: 57 mg (33%); mp 82–83 °C; *R*_f_ = 0.40 (CH_2_Cl_2_/MeOH 9:1); ^1^H NMR (600 MHz, DMSO-*d*_6_): δ 0.94 (s, 9H), 1.46–1.54 (m, 2H), 1.58–1.65 (m, 2H), 1.87–1.94 (m, 1H), 2.04–2.10 (m, 1H), 2.34 (s, 3H), 3.55–3.59 (m, 1H), 3.63 (dd, *J* = 10.8, 3.9 Hz, 1H), 3.90 (s, 3H), 4.27–4.40 (m, 3H), 4.46–4.54 (m, 2H), 5.14 (d, *J* = 3.7 Hz, 1H), 7.07–7.11 (m, 1H), 7.27 (d, *J* = 8.1 Hz, 1H), 7.34 (d, *J* = 8.9 Hz, 1H), 8.57 (t, *J* = 6.0 Hz, 1H), 9.11 (s, 1H); ^13^C NMR (151 MHz, DMSO-*d*_6_): δ 13.7, 15.7, 16.6, 16.8, 26.0, 36.2, 36.9, 37.8, 56.6, 57.3, 58.8, 61.3 (d, ^4^*J*_F,C_ = 4.8 Hz), 68.9, 118.7, 120.1, 123.6 (d, ^2^*J*_F,C_ = 10.2 Hz), 123.6, 125.5, 134.5, 145.1 (d, ^2^*J*_F,C_ = 11.4 Hz), 150.3, 151.8 (d, ^1^*J*_F,C_ = 248.1 Hz) 153.3, 164.4, 168.7, 171.7; LC–MS (ESI) (90% H_2_O to 100% MeCN in 10 min, then 100% MeCN to 15 min, DAD 200–600 nm), *t*_R_ = 5.49 min, 99% purity, *m*/*z* calcd for C_28_H_34_FN_5_O_5_S [M + H]^+^, 571.23; found, 572.5. HRMS (ESI) *m*/*z*: calcd for C_28_H_34_FN_5_O_5_S [M + H]^+^, 572.2337; found, 572.2330.

##### (2*S*,4*R*)-1-((*S*)-2-(1-Cyanocyclopropane-1-carboxamido)-3,3-dimethylbutanoyl)-4-hydroxy-*N*-((4-(4-methylthiazol-5-yl)naphthalen-1-yl)methyl)pyrrolidine-2-carboxamide (**22**)

Following general procedure C, compound **22** was obtained using Boc-protected amine **42v** (type **42**, subst. phenylene = 1,4-naphthylene; 106 mg, 0.3 mmol) and acid **46** (101 mg, 0.3 mmol). The residue was purified by flash column chromatography using a gradient from 0 to 10% MeOH in CH_2_Cl_2_ to afford **22** as a pale brown solid. Yield: 65 mg (38%); mp 127–129 °C; *R*_f_ = 0.40 (CH_2_Cl_2_/MeOH 9:1); ^1^H NMR (500 MHz, DMSO-*d*_6_): δ 0.96 (s, 9H), 1.45–1.56 (m, 2H), 1.57–1.66 (m, 2H), 1.91–2.00 (m, 1H), 2.03–2.10 (m, 1H), 2.17 (s, 3H), 3.55–3.60 (m, 1H), 3.66 (dd, *J* = 10.8, 4.0 Hz, 1H), 4.34–4.41 (m, 1H), 4.48–4.58 (m, 2H), 4.76–4.85 (m, 2H), 5.13 (d, *J* = 3.7 Hz, 1H), 7.33 (d, *J* = 8.9 Hz, 1H), 7.46 (d, *J* = 7.2 Hz, 1H), 7.54–7.69 (m, 4H), 8.13–8.19 (m, 1H), 8.68 (t, *J* = 5.8 Hz, 1H), 9.16 (s, 1H); ^13^C NMR (126 MHz, DMSO-*d*_6_): δ 13.7, 15.4, 16.6, 16.7, 26.1, 36.2, 37.9, 56.6, 57.3, 58.8, 68.9, 120.0, 124.1, 124.5, 125.5, 126.4, 126.7, 127.7, 128.4, 128.7, 131.0, 131.8, 135.9, 150.0, 152.8, 164.4, 168.6, 171.5; LC–MS (ESI) (90% H_2_O to 100% MeOH in 10 min, then 100% MeOH to 20 min, DAD 220–600 nm), *t*_R_ = 6.21 min, 97% purity, *m*/*z* calcd for C_31_H_35_N_5_O_4_S [M + H]^+^, 574.24; found, 574.4. HRMS (ESI) *m*/*z*: calcd for C_31_H_35_N_5_O_4_S [M + H]^+^, 574.2485; found, 574.2477.

##### (2*S*,4*R*)-1-((*S*)-2-(1-Cyanocyclopropane-1-carboxamido)-3,3-dimethylbutanoyl)-4-hydroxy-*N*-((5-(4-methylthiazol-5-yl)quinolin-8-yl)methyl)pyrrolidine-2-carboxamide (**23**)

Following general procedure C, compound **23** was obtained using Boc-protected amine **42w** (type **42**, subst. phenylene = quinoline-5,8-diyl; 107 mg, 0.3 mmol) and acid **46** (101 mg, 0.3 mmol). The residue was purified by flash column chromatography using a gradient from 0 to 10% MeOH in CH_2_Cl_2_ to afford **23** as a pale brown solid. Yield: 61 mg (36%); mp 116–118 °C; *R*_f_ = 0.40 (CH_2_Cl_2_/MeOH 9:1); ^1^H NMR (500 MHz, DMSO-*d*_6_): δ 0.93 (s, 9H), 1.45–1.56 (m, 2H), 1.58–1.66 (m, 2H), 1.95–2.01 (m, 1H), 2.07–2.15 (m, 1H), 2.18 (s, 3H), 3.58 (d, *J* = 10.8 Hz, 1H), 3.65 (dd, *J* = 10.9, 3.9 Hz, 1H), 4.34–4.39 (m, 1H), 4.53 (d, *J* = 8.9 Hz, 1H), 4.59 (t, *J* = 8.2 Hz, 1H), 4.90–5.03 (m, 2H), 5.15 (d, *J* = 3.6 Hz, 1H), 7.36 (d, *J* = 8.9 Hz, 1H), 7.54 (d, *J* = 7.4 Hz, 1H), 7.61 (dd, *J* = 8.5, 4.1 Hz, 1H), 7.88 (d, *J* = 7.4 Hz, 1H), 8.03 (dd, *J* = 8.6, 1.7 Hz, 1H), 8.68 (t, *J* = 6.1 Hz, 1H), 9.00 (dd, *J* = 4.2, 1.7 Hz, 1H), 9.19 (s, 1H); ^13^C NMR (126 MHz, DMSO-*d*_6_): δ 13.7, 15.5, 16.6, 16.7, 26.0, 36.2, 37.8, 56.6, 57.3, 58.9, 68.9, 120.1, 122.1, 126.2, 126.5, 127.1, 127.3, 129.2, 133.7, 137.9, 145.4, 149.9, 150.3, 153.2, 164.4, 168.8, 171.9; LC–MS (ESI) (90% H_2_O to 100% MeOH in 10 min, then 100% MeOH to 20 min, DAD 220–400 nm), *t*_R_ = 10.61 min, 98% purity, *m*/*z* calcd for C_30_H_34_N_6_O_4_S [M + H]^+^, 575.24; found, 575.3. HRMS (ESI) *m*/*z*: calcd for C_30_H_34_N_6_O_4_S [M + H]^+^, 575.2435; found, 575.2430.

##### (2*S*,4*R*)-1-((*S*)-2-(1-Cyanocyclopropane-1-carboxamido)-3,3-dimethylbutanoyl)-4-hydroxy-*N*-((*S*)-1-(2-methyl-4-(4-methylthiazol-5-yl)phenyl)ethyl)pyrrolidine-2-carboxamide (**24**)

Following general procedure C, compound **24** was obtained using Boc-protected amine **60a** (type **60**; R = 2-Me; 100 mg, 0.3 mmol) and acid **46** (101 mg, 0.3 mmol). The residue was purified by flash column chromatography using a gradient from 0 to 10% MeOH in CH_2_Cl_2_ to afford **24** as a white solid. Yield: 58 mg (35%); mp 110–112 °C; *R*_f_ = 0.40 (CH_2_Cl_2_/MeOH 9:1); ^1^H NMR (600 MHz, DMSO-*d*_6_): δ 0.95 (s, 9H), 1.35 (d, *J* = 6.9 Hz, 3H), 1.47–1.53 (m, 2H), 1.59–1.67 (m, 2H), 1.70–1.77 (m, 1H), 2.01–2.06 (m, 1H), 2.33 (s, 3H), 2.45 (s, 3H), 3.49–3.59 (m, 2H), 4.24–4.28 (m, 1H), 4.46 (t, *J* = 8.3 Hz, 1H), 4.50 (d, *J* = 8.9 Hz, 1H), 5.02–5.09 (m, 1H), 5.11 (d, *J* = 3.6 Hz, 1H), 7.23–7.26 (m, 1H), 7.28–7.32 (m, 2H), 7.39 (d, *J* = 8.0 Hz, 1H), 8.43 (d, *J* = 7.7 Hz, 1H), 8.97 (s, 1H); ^13^C NMR (151 MHz, DMSO-*d*_6_): δ 13.6, 16.0, 16.6, 16.8, 18.5, 21.0, 26.1, 36.2, 37.6, 44.4, 56.6, 57.3, 58.6, 68.7, 120.1, 125.3, 126.6, 129.6, 130.5, 131.1, 135.5, 142.6, 147.6, 151.3, 164.3, 168.5, 170.1; LC–MS (ESI) (90% H_2_O to 100% MeCN in 10 min, then 100% MeCN to 15 min, DAD 200–600 nm), *t*_R_ = 5.98 min, 97% purity, *m*/*z* calcd for C_29_H_37_N_5_O_4_S [M + H]^+^, 552.26; found, 552.4. HRMS (ESI) *m*/*z*: calcd for C_29_H_37_N_5_O_4_S [M + H]^+^, 552.2639; found, 552.2637.

##### (2*S*,4*R*)-1-((*S*)-2-(1-Cyanocyclopropane-1-carboxamido)-3,3-dimethylbutanoyl)-*N*-((*S*)-1-(3-fluoro-4-(4-methylthiazol-5-yl)phenyl)ethyl)-4-hydroxypyrrolidine-2-carboxamide (**25**)

Following general procedure C, compound **25** was obtained using Boc-protected amine **60b** (type **60**; R = 3-F; 101 mg, 0.3 mmol) and acid **46** (101 mg, 0.3 mmol). The residue was purified by flash column chromatography using a gradient from 0 to 10% MeOH in CH_2_Cl_2_ to afford **25** as a white solid. Yield: 62 mg (37%); mp 198 °C; *R*_f_ = 0.47 (CH_2_Cl_2_/MeOH 9:1); ^1^H NMR (600 MHz, DMSO-*d*_6_): δ 0.95 (s, 9H), 1.39 (d, *J* = 7.0 Hz, 3H), 1.47–1.53 (m, 2H), 1.59–1.67 (m, 2H), 1.75–1.81 (m, 1H), 2.06–2.11 (m, 1H), 2.33 (s, 3H), 3.51–3.61 (m, 2H), 4.27–4.32 (m, 1H), 4.47 (dd, *J* = 9.0, 7.7 Hz, 1H), 4.51 (d, *J* = 8.9 Hz, 1H), 4.89–4.97 (m, 1H), 5.13 (d, *J* = 3.6 Hz, 1H), 7.20–7.32 (m, 3H), 7.44 (t, *J* = 7.8 Hz, 1H), 8.50 (d, *J* = 7.6 Hz, 1H), 9.10 (s, 1H); ^13^C NMR (151 MHz, DMSO-*d*_6_): δ 13.7, 15.7 (d, ^5^*J*_F,C_ = 1.9 Hz), 16.6, 16.8, 22.3, 26.1, 36.2, 37.7, 47.6, 56.6, 57.3, 58.7, 68.8, 113.3 (d, ^2^*J*_F,C_ = 22.9 Hz), 116.9 (d, ^2^*J*_F,C_ = 15.3 Hz), 120.1, 122.1 (d, ^3^*J*_F,C_ = 2.8 Hz), 123.8, 131.9 (d, ^2^*J*_F,C_ = 2.5 Hz), 148.5 (d, ^3^*J*_F,C_ = 7.3 Hz), 150.1, 153.1, 158.8 (d, ^1^*J*_F,C_ = 246.4 Hz), 164.3, 168.6, 170.5; LC–MS (ESI) (90% H_2_O to 100% MeCN in 10 min, then 100% MeCN to 15 min, DAD 200–600 nm), *t*_R_ = 5.80 min, 95% purity, *m*/*z* calcd for C_17_H_22_N_2_O_2_S [M + H]^+^, 556.24; found, 556.4. HRMS (ESI) *m*/*z*: calcd for C_17_H_22_N_2_O_2_S [M + H]^+^, 556.2388; found, 556.2388.

##### (2*S*,4*R*)-1-((*S*)-2-(1-Fluorocyclopropane-1-carboxamido)-3,3-dimethylbutanoyl)-4-hydroxy-*N*-(2-methyl-4-(4-methylthiazol-5-yl)benzyl)pyrrolidine-2-carboxamide (**26**)

Following general procedure C, compound **26** was obtained using Boc-protected amine **42b** (type **42**; R = 2-Me; 127 mg, 0.4 mmol) and acid **62** (132 mg, 0.4 mmol). The residue was purified by flash column chromatography using a gradient from 0 to 10% MeOH in CH_2_Cl_2_ to afford **26** as a yellow solid. Yield: 137 mg (66%); mp 107–108 °C; *R*_f_ = 0.48 (CH_2_Cl_2_/MeOH 9:1); ^1^H NMR (500 MHz, DMSO-*d*_6_): δ 0.97 (s, 9H), 1.17–1.26 (m, 2H), 1.29–1.43 (m, 2H), 1.87–1.96 (m, 1H), 2.04–2.12 (m, 1H), 2.30 (s, 3H), 2.45 (s, 3H), 3.60 (d, *J* = 10.8 Hz, 1H), 3.66 (dd, *J* = 10.7, 3.9 Hz, 1H), 4.21 (dd, *J* = 15.6, 5.3 Hz, 1H), 4.31–4.38 (m, 2H), 4.50 (t, *J* = 8.2 Hz, 1H), 4.56–4.62 (m, 1H), 5.13 (d, *J* = 3.7 Hz, 1H), 7.20–7.30 (m, 3H), 7.41 (d, *J* = 7.9 Hz, 1H), 8.47 (t, *J* = 5.7 Hz, 1H), 8.97 (s, 1H); ^13^C NMR (126 MHz, DMSO-*d*_6_): δ 12.6 (d, ^2^*J*_F,C_ = 10.5 Hz), 12.9 (d, ^2^*J*_F,C_ = 10.1 Hz), 15.9, 18.4, 26.1, 36.0, 37.9, 54.8, 56.5, 56.6, 58.7, 68.9, 78.1 (d, ^1^*J*_F,C_ = 232.6 Hz), 126.1, 127.9, 129.8, 130.2, 131.1, 136.3, 136.9, 147.6, 151.3, 168.0 (d, ^2^*J*_F,C_ = 20.1 Hz), 168.8, 171.4; LC–MS (ESI) (90% H_2_O to 100% MeCN in 10 min, then 100% MeCN to 15 min, DAD 200–600 nm), *t*_R_ = 5.77 min, 99% purity, *m*/*z* calcd for C_27_H_35_FN_4_O_4_S [M + H]^+^, 531.24; found, 531.3. HRMS (ESI) *m*/*z*: calcd for C_27_H_35_FN_4_O_4_S [M + H]^+^, 531.2436; found, 531.2430.

##### (2*S*,4*R*)-*N*-(3-Fluoro-4-(4-methylthiazol-5-yl)benzyl)-1-((*S*)-2-(1-fluorocyclopropane-1-carboxamido)-3,3-dimethylbutanoyl)-4-hydroxypyrrolidine-2-carboxamide (**27**)

Following general procedure C, compound **27** was obtained using Boc-protected amine **42h** (type **42**; R = 3-F; 129 mg, 0.4 mmol) and acid **62** (132 mg, 0.4 mmol). The residue was purified by flash column chromatography using a gradient from 0 to 10% MeOH in CH_2_Cl_2_ to afford **27** as a white solid. Yield: 155 mg (74%); mp 92–94 °C; *R*_f_ = 0.48 (CH_2_Cl_2_/MeOH 9:1); ^1^H NMR (600 MHz, DMSO-*d*_6_): δ 0.96 (s, 9H), 1.17–1.25 (m, 2H), 1.31–1.41 (m, 2H), 1.88–1.94 (m, 1H), 2.05–2.12 (m, 1H), 2.32 (s, 3H), 3.58–3.63 (m, 1H), 3.67 (dd, *J* = 10.8, 3.9 Hz, 1H), 4.23 (dd, *J* = 16.1, 5.5 Hz, 1H), 4.33–4.38 (m, 1H), 4.44–4.51 (m, 2H), 4.55–4.61 (m, 1H), 5.16 (d, *J* = 3.6 Hz, 1H), 7.19–7.28 (m, 2H), 7.33 (dd, *J* = 11.4, 1.6 Hz, 1H), 7.40 (t, *J* = 7.8 Hz, 1H), 8.69 (t, *J* = 6.1 Hz, 1H), 9.09 (s, 1H); ^13^C NMR (151 MHz, DMSO-*d*_6_): δ 12.7 (d, ^2^*J*_F,C_ = 10.1 Hz), 12.9 (d, ^2^*J*_F,C_ = 10.2 Hz), 15.7, 26.1, 36.0, 37.8, 41.4, 56.5, 56.6, 58.8, 68.9, 78.1 (d, ^1^*J*_F,C_ = 231.9 Hz), 114.4 (d, ^2^*J*_F,C_ = 23.0 Hz), 116.8 (d, ^2^*J*_F,C_ = 15.4 Hz), 123.1 (d, ^3^*J*_F,C_ = 3.3 Hz), 123.8, 131.7 (d, ^3^*J*_F,C_ = 2.8 Hz), 143.2 (d, ^3^*J*_F,C_ = 7.6 Hz), 150.1, 153.1, 158.9 (d, ^2^*J*_F,C_ = 246.3 Hz), 168.0 (d, ^2^*J*_F,C_ = 21.0 Hz), 168.9, 171.9; LC–MS (ESI) (90% H_2_O to 100% MeCN in 10 min, then 100% MeCN to 15 min, DAD 200–600 nm), *t*_R_ = 5.59 min, 100% purity, *m*/*z* calcd for C_26_H_32_F_2_N_4_O_4_S [M + H]^+^, 535.22; found, 535.3. HRMS (ESI) *m*/*z*: calcd for C_26_H_32_F_2_N_4_O_4_S [M + H]^+^, 535.2185; found, 535.2179.

##### (2*S*,4*R*)-*N*-(5-Fluoro-2-methyl-4-(4-methylthiazol-5-yl)benzyl)-1-((*S*)-2-(1-fluorocyclopropane-1-carboxamido)-3,3-dimethylbutanoyl)-4-hydroxypyrrolidine-2-carboxamide (**28**)

Following general procedure C, compound **28** was obtained using Boc-protected amine **42x** (type **42**; R = 2-Me, 5-F; 100 mg, 0.3 mmol) and acid **62** (99 mg, 0.3 mmol). The residue was purified by flash column chromatography using a gradient from 0 to 10% MeOH in CH_2_Cl_2_ to afford **28** as a white solid. Yield: 111 mg (67%); mp 85–86 °C °C; *R*_f_ = 0.51 (CH_2_Cl_2_/MeOH 9:1); ^1^H NMR (500 MHz, DMSO-*d*_6_): δ 0.95 (s, 9H), 1.18–1.28 (m, 2H), 1.30–1.42 (m, 2H), 1.87–1.96 (m, 1H), 2.05–2.12 (m, 1H), 2.26 (s, 3H), 2.32 (d, *J* = 1.2 Hz, 3H), 3.61 (d, *J* = 10.8 Hz, 1H), 3.67 (dd, *J* = 10.8, 3.8 Hz, 1H), 4.14 (dd, *J* = 16.2, 5.2 Hz, 1H), 4.33–4.42 (m, 2H), 4.51 (t, *J* = 8.2 Hz, 1H), 4.56–4.62 (m, 1H), 5.16 (d, *J* = 3.6 Hz, 1H), 7.21–7.28 (m, 2H), 7.35 (d, *J* = 11.3 Hz, 1H), 8.62 (t, *J* = 5.9 Hz, 1H), 9.08 (s, 1H); ^13^C NMR (126 MHz, DMSO-*d*_6_): δ 12.6 (d, ^2^*J*_F,C_ = 10.2 Hz), 12.9 (d, ^2^*J*_F,C_ = 10.1 Hz), 15.6 (d, ^5^*J*_F,C_ = 2.8 Hz), 17.5, 26.1, 36.0, 37.8, 56.5, 56.6, 58.8, 68.9, 78.1 (d, ^1^*J*_F,C_ = 232.3 Hz), 114.4 (d, ^2^*J*_F,C_ = 23.7 Hz), 116.3 (d, ^2^*J*_F,C_ = 15.2 Hz), 123.9, 131.5 (d, ^3^*J*_F,C_ = 3.4 Hz), 132.7 (d, ^3^*J*_F,C_ = 2.3 Hz), 140.4 (d, ^3^*J*_F,C_ = 7.2 Hz), 149.9, 152.8, 157.4 (d, ^1^*J*_F,C_ = 244.0 Hz), 168.0 (d, ^2^*J*_F,C_ = 20.2 Hz), 168.9, 171.8; LC–MS (ESI) (90% H_2_O to 100% MeCN in 10 min, then 100% MeCN to 15 min, DAD 200–600 nm), *t*_R_ = 5.84 min, 99% purity, *m*/*z* calcd for C_27_H_34_F_2_N_4_O_4_S [M + H]^+^, 549.23; found, 549.4. HRMS (ESI) *m*/*z*: calcd for C_27_H_34_F_2_N_4_O_4_S [M + H]^+^, 549.2341; found, 549.2334.

##### (2*S*,4*R*)-*N*-(5-Fluoro-2-methoxy-4-(4-methylthiazol-5-yl)benzyl)-1-((*S*)-2-(1-fluorocyclopropane-1-carboxamido)-3,3-dimethylbutanoyl)-4-hydroxypyrrolidine-2-carboxamide (**29**)

Following general procedure C, compound **29** was obtained using Boc-protected amine **42y** (type **42**; R = 2-OMe, 5-F; 141 mg, 0.4 mmol) and acid **62** (132 mg, 0.4 mmol). The residue was purified by flash column chromatography using a gradient from 0 to 10% MeOH in CH_2_Cl_2_ to afford **29** as a white solid. Yield: 128 mg (55%); mp 88–90 °C; *R*_f_ = 0.44 (CH_2_Cl_2_/MeOH 9:1); ^1^H NMR (600 MHz, DMSO-*d*_6_): δ 0.94 (s, 9H), 1.17–1.27 (m, 2H), 1.30–1.41 (m, 2H), 1.87–1.96 (m, 1H), 2.04–2.11 (m, 1H), 2.33 (d, *J* = 1.2 Hz, 3H), 3.60 (d, *J* = 10.8 Hz, 1H), 3.65 (dd, *J* = 10.8, 3.8 Hz, 1H), 4.11 (dd, *J* = 16.8, 5.3 Hz, 1H), 4.25–4.39 (m, 2H), 4.49 (t, *J* = 8.3 Hz, 1H), 4.58 (d, *J* = 9.2 Hz, 1H), 5.15 (d, *J* = 3.6 Hz, 1H), 6.97 (d, *J* = 5.9 Hz, 1H), 7.24 (dd, *J* = 9.2, 2.8 Hz, 1H), 7.36 (d, *J* = 10.6 Hz, 1H), 8.60 (t, *J* = 6.0 Hz, 1H), 9.08 (s, 1H); ^13^C NMR (151 MHz, DMSO-*d*_6_): δ 12.7 (d, ^2^*J*_F,C_ = 10.4 Hz), 12.9 (d, ^2^*J*_F,C_ = 10.1 Hz), 15.7, 26.1, 36.0, 37.1, 37.8, 56.1, 56.5, 56.6, 58.9, 68.9, 78.2 (d, ^1^*J*_F,C_ = 232.3 Hz), 112.9, 114.9 (d, ^2^*J*_F,C_ = 25.7 Hz), 116.7 (d, ^2^*J*_F,C_ = 16.7 Hz), 124.1, 130.0 (d, ^3^*J*_F,C_ = 7.4 Hz), 150.2, 152.3, 153.0, 153.3 (d, ^1^*J*_F,C_ = 238.8 Hz), 168.0 (d, ^2^*J*_F,C_ = 20.6 Hz), 169.0, 172.1; LC–MS (ESI) (90% H_2_O to 100% MeCN in 10 min, then 100% MeCN to 15 min, DAD 200–600 nm), *t*_R_ = 5.82 min, 95% purity, *m*/*z* calcd for C_27_H_35_F_2_N_4_O_5_S [M + H]^+^, 565.23; found, 565.4. HRMS (ESI) *m*/*z*: calcd for C_27_H_35_F_2_N_4_O_5_S [M + H]^+^, 565.2291; found, 565.2284.

##### (2*S*,4*R*)-1-((*S*)-2-(1-Fluorocyclopropane-1-carboxamido)-3,3-dimethylbutanoyl)-4-hydroxy-*N*-((*S*)-1-(2-methyl-4-(4-methylthiazol-5-yl)phenyl)ethyl)pyrrolidine-2-carboxamide (**30**)

Following general procedure C, compound **30** was obtained using Boc-protected amine **60a** (type **60**; R = 2-Me; 100 mg, 0.3 mmol) and acid **62** (99 mg, 0.3 mmol). The residue was purified by flash column chromatography using a gradient from 0 to 10% MeOH in CH_2_Cl_2_ to afford **30** as a white solid. Yield: 118 mg (72%); mp 196 °C; *R*_f_ = 0.42 (CH_2_Cl_2_/MeOH 9:1); ^1^H NMR (500 MHz, DMSO-*d*_6_): δ 0.97 (s, 9H), 1.19–1.28 (m, 2H), 1.32–1.41 (m, 5H), 1.71–1.78 (m, 1H), 2.01–2.07 (m, 1H), 2.33 (s, 3H), 2.46 (s, 3H), 3.53–3.61 (m, 2H), 4.24–4.30 (m, 1H), 4.46 (t, *J* = 8.2 Hz, 1H), 4.55–4.60 (m, 1H), 5.03–5.09 (m, 1H), 5.10 (d, *J* = 3.6 Hz, 1H), 7.21–7.26 (m, 2H), 7.31 (dd, *J* = 8.0, 2.0 Hz, 1H), 7.39 (d, *J* = 8.0 Hz, 1H), 8.40 (d, *J* = 7.7 Hz, 1H), 8.97 (s, 1H); ^13^C NMR (126 MHz, DMSO-*d*_6_): δ 12.6 (d, ^2^*J*_F,C_ = 10.4 Hz), 12.9 (d, ^2^*J*_F,C_ = 10.4 Hz), 16.0, 18.5, 21.0, 26.2, 36.0, 37.6, 44.4, 56.5, 56.5, 58.6, 68.7, 78.1 (d, ^1^*J*_F,C_ = 232.5 Hz), 125.3, 126.5, 129.6, 130.5, 131.1, 135.5, 142.5, 147.6, 151.3, 167.9 (d, ^2^*J*_F,C_ = 20.1 Hz), 168.7, 170.1; LC–MS (ESI) (90% H_2_O to 100% MeCN in 10 min, then 100% MeCN to 15 min, DAD 200–600 nm), *t*_R_ = 6.13 min, 98% purity, *m*/*z* calcd for C_28_H_37_FN_4_O_4_S [M + H]^+^, 545.26; found, 545.4. HRMS (ESI) *m*/*z*: calcd for C_28_H_37_FN_4_O_4_S [M + H]^+^, 545.2592; found, 545.2586.

##### (2*S*,4*R*)-*N*-((*S*)-1-(3-Fluoro-4-(4-methylthiazol-5-yl)phenyl)ethyl)-1-((*S*)-2-(1-fluorocyclopropane-1-carboxamido)-3,3-dimethylbutanoyl)-4-hydroxypyrrolidine-2-carboxamide (**31**)

Following general procedure C, compound **31** was obtained using Boc-protected amine **60b** (type **60**; R = 3-F; 101 mg, 0.3 mmol) and acid **62** (99 mg, 0.3 mmol). The residue was purified by flash column chromatography using a gradient from 0 to 10% MeOH in CH_2_Cl_2_ to afford **31** as a white solid. Yield: 129 mg (78%); mp 198–200 °C; *R*_f_ = 0.42 (CH_2_Cl_2_/MeOH 9:1); ^1^H NMR (500 MHz, DMSO-*d*_6_): δ 0.97 (s, 9H), 1.19–1.24 (m, 2H), 1.32–1.45 (m, 5H), 1.75–1.82 (m, 1H), 2.06–2.11 (m, 1H), 2.34 (d, *J* = 1.1 Hz, 3H), 3.54–3.63 (m, 2H), 4.28–4.32 (m, 1H), 4.47 (t, *J* = 8.3 Hz, 1H), 4.58 (dd, *J* = 9.3, 1.3 Hz, 1H), 4.90–4.97 (m, 1H), 5.13 (d, *J* = 3.6 Hz, 1H), 7.17–7.29 (m, 3H), 7.42–7.47 (m, 1H), 8.47 (d, *J* = 7.6 Hz, 1H), 9.09 (s, 1H); ^13^C NMR (126 MHz, DMSO-*d*_6_): δ 12.6 (d, ^2^*J*_F,C_ = 10.2 Hz), 12.9 (d, ^2^*J*_F,C_ = 10.1 Hz), 15.7 (d, ^5^*J*_F,C_ = 2.6 Hz), 22.3, 26.2, 36.0, 37.7, 47.5, 56.5, 56.6, 58.6, 68.8, 78.1 (d, ^1^*J*_F,C_ = 232.4 Hz), 113.3 (d, ^2^*J*_F,C_ = 22.8 Hz), 116.9 (d, ^2^*J*_F,C_ = 15.4 Hz), 122.1 (d, ^3^*J*_F,C_ = 3.0 Hz), 123.7, 131.9 (d, ^3^*J*_F,C_ = 2.8 Hz), 148.4 (d, ^3^*J*_F,C_ = 7.1 Hz), 150.1, 153.1, 158.8 (d, ^1^*J*_F,C_ = 246.5 Hz), 168.0 (d, ^2^*J*_F,C_ = 20.2 Hz), 168.8, 170.5; LC–MS (ESI) (90% H_2_O to 100% MeCN in 10 min, then 100% MeCN to 15 min, DAD 200–600 nm), *t*_R_ = 5.94 min, 98% purity, *m*/*z* calcd for C_27_H_34_F_2_N_4_O_4_S [M + H]^+^, 549.23; found, 549.3. HRMS (ESI) *m*/*z*: calcd for C_27_H_34_F_2_N_4_O_4_S [M + H]^+^, 549.2342; found, 549.2336.

##### (2*S*,4*R*)-*N*-((*S*)-1-(5-Fluoro-2-methyl-4-(4-methylthiazol-5-yl)phenyl)ethyl)-1-((*S*)-2-(1-fluorocyclopropane-1-carboxamido)-3,3-dimethylbutanoyl)-4-hydroxypyrrolidine-2-carboxamide (**32**)

Following general procedure C, compound **32** was obtained using Boc-protected amine **60c** (type **60**; R = 2-Me, 5-F; 103 mg, 0.3 mmol) and acid **62** (99 mg, 0.3 mmol). The residue was purified by flash column chromatography using a gradient from 0 to 10% MeOH in CH_2_Cl_2_ to afford **32** as a white solid. Yield: 122 mg (72%); mp 142–145 °C; *R*_f_ = 0.40 (CH_2_Cl_2_/MeOH 9:1); ^1^H NMR (600 MHz, DMSO-*d*_6_): δ 0.96 (s, 9H), 1.19–1.23 (m, 2H), 1.31–1.41 (m, 5H), 1.70–1.77 (m, 1H), 2.03–2.09 (m, 1H), 2.30 (s, 3H), 2.33 (s, 3H), 3.54–3.61 (m, 2H), 4.26–4.31 (m, 1H), 4.44 (t, *J* = 8.3 Hz, 1H), 4.57 (d, *J* = 9.3 Hz, 1H), 5.00–5.07 (m, 1H), 5.13 (d, *J* = 3.6 Hz, 1H), 7.20–7.29 (m, 3H), 8.44 (d, *J* = 7.7 Hz, 1H), 9.09 (s, 1H); ^13^C NMR (151 MHz, DMSO-*d*_6_): δ 12.6 (d, ^2^*J*_F,C_ = 10.1 Hz), 12.9 (d, ^2^*J*_F,C_ = 10.4 Hz), 15.8 (d, ^5^*J*_F,C_ = 3.1 Hz), 17.6, 20.8, 26.2, 36.0, 37.6, 44.6, 56.5, 56.6, 58.7, 68.8, 78.1 (d, ^1^*J*_F,C_ = 232.4 Hz), 112.4 (d, ^2^*J*_F,C_ = 23.0 Hz), 116.6 (d, ^2^*J*_F,C_ = 15.2 Hz), 123.8, 131.2 (d, ^4^*J*_F,C_ = 3.2 Hz), 133.2, 146.0 (d, ^3^*J*_F,C_ = 6.5 Hz), 150.0, 153.0, 157.5 (d, ^1^*J*_F,C_ = 244.1 Hz), 168.0 (d, ^2^*J*_F,C_ = 19.9 Hz), 168.8, 170.3; LC–MS (ESI) (90% H_2_O to 100% MeCN in 10 min, then 100% MeCN to 15 min, DAD 200–600 nm), *t*_R_ = 6.38 min, 98% purity, *m*/*z* calcd for C_28_H_36_F_2_N_4_O_4_S [M + H]^+^, 563.25; found, 563.4. HRMS (ESI) *m*/*z*: calcd for C_28_H_36_F_2_N_4_O_4_S [M + H]^+^, 563.2498; found, 563.2492.

##### (2*S*,4*R*)-*N*-((*S*)-1-(5-Fluoro-2-methoxy-4-(4-methylthiazol-5-yl)phenyl)ethyl)-1-((*S*)-2-(1-fluorocyclopropane-1-carboxamido)-3,3-dimethylbutanoyl)-4-hydroxypyrrolidine-2-carboxamide (**33**)

Following general procedure C, compound **33** was obtained using Boc-protected amine **60d** (type **60**; R = 2-OMe, 5-F; 110 mg, 0.3 mmol) and acid **62** (99 mg, 0.3 mmol). The residue was purified by flash column chromatography using a gradient from 0 to 10% MeOH in CH_2_Cl_2_ to afford **33** as a white solid. Yield: 52 mg (60%); mp 115–116 °C; *R*_f_ = 0.42 (CH_2_Cl_2_/MeOH 9:1); ^1^H NMR (600 MHz, DMSO-*d*_6_): δ 0.97 (s, 9H), 1.19–1.25 (m, 2H), 1.28–1.40 (m, 5H), 1.74–1.81 (m, 1H), 2.07–2.13 (m, 1H), 2.36 (s, 3H), 3.54–3.62 (m, 2H), 3.83 (s, 3H), 4.29–4.32 (m, 1H), 4.48 (t, *J* = 8.3 Hz, 1H), 4.58 (d, *J* = 9.2 Hz, 1H), 5.11–5.18 (m, 2H), 7.01 (d, *J* = 6.1 Hz, 1H), 7.17 (d, *J* = 10.6 Hz, 1H), 7.25 (dd, *J* = 9.3, 2.9 Hz, 1H), 8.42 (d, *J* = 7.9 Hz, 1H), 9.10 (s, 1H); ^13^C NMR (151 MHz, DMSO-*d*_6_): δ 12.6 (d, ^2^*J*_F,C_ = 10.4 Hz), 12.9 (d, ^2^*J*_F,C_ = 10.2 Hz), 15.8 (d, ^5^*J*_F,C_ = 1.9 Hz), 21.1, 26.2, 36.1, 37.6, 42.7, 56.2, 56.5, 56.6, 58.6, 68.8, 78.1 (d, ^1^*J*_F,C_ = 232.6 Hz), 113.0 (d, ^2^*J*_F,C_ = 25.1 Hz), 113.6, 117.0 (d, ^2^*J*_F,C_ = 16.5 Hz), 123.9, 135.7 (d, ^3^*J*_F,C_ = 6.5 Hz), 150.3, 151.9, 153.1, 153.2 (d, ^1^*J*_F,C_ = 238.9 Hz), 168.0 (d, ^2^*J*_F,C_ = 20.2 Hz), 168.8, 170.3; LC–MS (ESI) (90% H_2_O to 100% MeCN in 10 min, then 100% MeCN to 15 min, DAD 200–600 nm), *t*_R_ = 6.21 min, 99% purity, *m*/*z* calcd for C_28_H_36_F_2_N_4_O_5_S [M + H]^+^, 579.24; found, 579.5. HRMS (ESI) *m*/*z*: calcd for C_28_H_36_F_2_N_4_O_5_S [M + H]^+^, 579.2447; found, 579.2445.

##### (2*S*,4*R*)-1-((*S*)-2-(1-Cyanocyclopropane-1-carboxamido)-3,3-dimethylbutanoyl)-4-hydroxy-*N*-((*S*)-5-(4-methylthiazol-5-yl)-2,3-dihydro-1*H*-inden-1-yl)pyrrolidine-2-carboxamide (**34**)

Following general procedure C, compound **34** was obtained using Boc-protected amine **60e** (type **60**; R = H, *n* = 1; 99 mg, 0.3 mmol) and acid **46** (101 mg, 0.3 mmol). The residue was purified by flash column chromatography using a gradient from 0 to 10% MeOH in CH_2_Cl_2_ to afford **34** as a white solid. Yield: 59 mg (36%); mp 102–104 °C; *R*_f_ = 0.39 (CH_2_Cl_2_/MeOH 9:1); ^1^H NMR (500 MHz, DMSO-*d*_6_): δ 0.97 (s, 9H), 1.45–1.53 (m, 2H), 1.57–1.65 (m, 2H), 1.85–1.92 (m, 1H), 1.92–1.99 (m, 1H), 2.01–2.10 (m, 1H), 2.39–2.46 (m, 4H), 2.80–2.89 (m, 1H), 2.93–3.02 (m, 1H), 3.52–3.58 (m, 1H), 3.65 (dd, *J* = 10.8, 3.9 Hz, 1H), 4.31–4.37 (m, 1H), 4.38–4.45 (m, 1H), 4.52 (d, *J* = 8.9 Hz, 1H), 5.11 (d, *J* = 3.7 Hz, 1H), 5.23–5.30 (m, 1H), 7.19–7.25 (m, 1H), 7.26–7.31 (m, 2H), 7.34–7.36 (m, 1H), 8.34 (d, *J* = 8.3 Hz, 1H), 8.96 (d, *J* = 1.8 Hz, 1H); ^13^C NMR (126 MHz, DMSO-*d*_6_): δ 13.6, 15.9, 16.6, 16.8, 26.1, 29.6, 32.7, 36.2, 38.1, 53.4, 56.6, 57.3, 58.7, 68.8, 120.0, 124.2, 125.1, 127.3, 130.6, 131.4, 143.8, 144.1, 147.7, 151.3, 164.3, 168.6, 171.2; LC–MS (ESI) (90% H_2_O to 100% MeCN in 10 min, then 100% MeCN to 15 min, DAD 200–600 nm), *t*_R_ = 5.85 min, 95% purity, *m*/*z* calcd for C_29_H_35_N_5_O_4_S [M + H]^+^, 550.25; found, 550.4. HRMS (ESI) *m*/*z*: calcd for C_29_H_35_N_5_O_4_S [M + H]^+^, 550.2486; found, 550.2481.

##### (2*S*,4*R*)-1-((*S*)-2-(1-Cyanocyclopropane-1-carboxamido)-3,3-dimethylbutanoyl)-*N*-((*S*)-6-fluoro-5-(4-methylthiazol-5-yl)-2,3-dihydro-1*H*-inden-1-yl)-4-hydroxypyrrolidine-2-carboxamide (**35**)

Following general procedure C, compound **35** was obtained using Boc-protected amine **60f** (type **60**, R = F, *n* = 1; 104 mg, 0.3 mmol) and acid **46** (99 mg, 0.3 mmol). The residue was purified by flash column chromatography using a gradient from 0 to 10% MeOH in CH_2_Cl_2_ to afford **35** as a white solid. Yield: 77 mg (45%); mp 163–165 °C; *R*_f_ = 0.38 (CH_2_Cl_2_/MeOH 9:1); ^1^H NMR (600 MHz, DMSO-*d*_6_): δ 0.97 (s, 9H), 1.47–1.53 (m, 2H), 1.58–1.66 (m, 2H), 1.91–2.00 (m, 2H), 2.05–2.11 (m, 1H), 2.32 (s, 3H), 2.41–2.47 (m, 1H), 2.79–2.86 (m, 1H), 2.92–2.98 (m, 1H), 3.53–3.58 (m, 1H), 3.66 (dd, *J* = 10.8, 3.9 Hz, 1H), 4.34–4.37 (m, 1H), 4.42 (dd, *J* = 8.9, 7.7 Hz, 1H), 4.52 (d, *J* = 8.9 Hz, 1H), 5.14 (d, *J* = 3.6 Hz, 1H), 5.23–5.28 (m, 1H), 7.06 (d, *J* = 9.7 Hz, 1H), 7.30–7.36 (m, 2H), 8.43 (d, *J* = 8.0 Hz, 1H), 9.09 (s, 1H); ^13^C NMR (151 MHz, DMSO-*d*_6_): δ 13.7, 15.7 (d, ^5^*J*_F,C_ = 3.0 Hz), 16.6, 16.8, 26.1, 29.0, 33.0, 36.2, 38.0, 53.8, 56.6, 57.3, 58.8, 68.8, 111.3 (d, ^2^*J*_F,C_ = 23.3 Hz), 117.7 (d, ^2^*J*_F,C_ = 16.7 Hz), 120.1, 124.3, 127.6, 139.1, 147.2 (d, ^3^*J*_F,C_ = 7.7 Hz), 153.0, 158.1 (d, ^1^*J*_F,C_ = 244.4 Hz), 164.4, 168.6, 171.3; LC–MS (ESI) (90% H_2_O to 100% MeCN in 10 min, then 100% MeCN to 15 min, DAD 200–600 nm), *t*_R_ = 6.00 min, 99% purity, *m*/*z* calcd for C_29_H_34_FN_5_O_4_S [M + H]^+^, 568.24; found, 568.4. HRMS (ESI) *m*/*z*: calcd for C_29_H_34_FN_5_O_4_S [M + H]^+^, 568.2388; found, 568.2382.

##### (2*S*,4*R*)-*N*-((*S*)-6-Fluoro-5-(4-methylthiazol-5-yl)-2,3-dihydro-1*H*-inden-1-yl)-1-((*S*)-2-(1-fluorocyclopropane-1-carboxamido)-3,3-dimethylbutanoyl)-4-hydroxypyrrolidine-2-carboxamide (**36**)

Following general procedure C, compound **36** was obtained using Boc-protected amine **60f** (type **60**, R = F, *n* = 1; 105 mg, 0.3 mmol) and acid **62** (99 mg, 0.3 mmol). The residue was purified by flash column chromatography using a gradient from 0 to 10% MeOH in CH_2_Cl_2_ to afford **36** as a white solid. Yield: 131 mg (78%); mp 109–111 °C; *R*_f_ = 0.38 (CH_2_Cl_2_/MeOH 9:1); ^1^H NMR (600 MHz, DMSO-*d*_6_): δ 0.98 (s, 9H), 1.16–1.25 (m, 2H), 1.30–1.42 (m, 2H), 1.89–2.00 (m, 2H), 2.05–2.11 (m, 1H), 2.32 (s, 3H), 2.40–2.46 (m, 1H), 2.77–2.86 (m, 1H), 2.91–2.98 (m, 1H), 3.56–3.61 (m, 1H), 3.68 (dd, *J* = 10.8, 3.9 Hz, 1H), 4.34–4.38 (m, 1H), 4.42 (t, *J* = 8.2 Hz, 1H), 4.57–4.61 (m, 1H), 5.14 (d, *J* = 3.6 Hz, 1H), 5.22–5.29 (m, 1H), 7.04–7.09 (m, 1H), 7.26 (dd, *J* = 9.4, 2.8 Hz, 1H), 7.32–7.36 (m, 1H), 8.41 (d, *J* = 8.0 Hz, 1H), 9.09 (s, 1H); ^13^C NMR (151 MHz, DMSO-*d*_6_): δ 12.6 (d, ^2^*J*_F,C_ = 10.3 Hz), 12.9 (d, ^2^*J*_F,C_ = 9.9 Hz), 15.7 (d, ^5^*J*_F,C_ = 2.6 Hz), 26.2, 29.0, 33.0, 36.0, 38.0, 53.8, 56.5, 56.6, 58.8, 68.8, 78.1 (d, ^1^*J*_F,C_ = 232.7 Hz), 111.3 (d, ^2^*J*_F,C_ = 23.2 Hz), 117.7 (d, ^2^*J*_F,C_ = 16.3 Hz), 124.3, 127.6, 139.1 (d, ^4^*J*_F,C_ = 2.6 Hz), 147.2 (d, ^3^*J*_F,C_ = 7.6 Hz), 150.1, 153.0, 158.1 (d, ^1^*J*_F,C_ = 244.5 Hz), 168.0 (d, ^2^*J*_F,C_ = 20.2 Hz), 168.8, 171.3; LC–MS (ESI) (90% H_2_O to 100% MeCN in 10 min, then 100% MeCN to 15 min, DAD 200–600 nm), *t*_R_ = 6.18 min, 100% purity, *m*/*z* calcd for C_28_H_34_F_2_N_4_O_4_S [M + H]^+^, 561.23; found, 561.3. HRMS (ESI) *m*/*z*: calcd for C_28_H_34_F_2_N_4_O_4_S [M + H]^+^, 561.2342; found, 561.2337.

##### (2*S*,4*R*)-*N*-((*S*)-7-Fluoro-6-(4-methylthiazol-5-yl)-1,2,3,4-tetrahydronaphthalen-1-yl)-1-((*S*)-2-(1-fluorocyclopropane-1-carboxamido)-3,3-dimethylbutanoyl)-4-hydroxypyrrolidine-2-carboxamide (**37**)

Following general procedure C, compound **37** was obtained using Boc-protected amine **60g** (type **60**, R = H, *n* = 2; 103 mg, 0.3 mmol) and acid **62** (99 mg, 0.3 mmol). The residue was purified by flash column chromatography using a gradient from 0 to 10% MeOH in CH_2_Cl_2_ to afford **37** as a white solid. Yield: 159 mg (95%); mp 78–82 °C; *R*_f_ = 0.38 (CH_2_Cl_2_/MeOH 9:1); ^1^H NMR (600 MHz, DMSO-*d*_6_): δ 0.98 (s, 9H), 1.13–1.28 (m, 4H), 1.30–1.41 (m, 2H), 1.68–1.78 (m, 2H), 1.87–1.94 (m, 2H), 1.95–2.00 (m, 1H), 2.04–2.11 (m, 1H), 2.32 (s, 3H), 3.55–3.61 (m, 1H), 3.69 (dd, *J* = 10.7, 4.0 Hz, 1H), 4.34–4.39 (m, 1H), 4.43 (t, *J* = 8.2 Hz, 1H), 4.55–4.62 (m, 1H), 4.90–4.96 (m, 1H), 5.14 (d, *J* = 3.6 Hz, 1H), 6.95–7.04 (m, 1H), 7.19–7.29 (m, 2H), 8.41 (d, *J* = 8.5 Hz, 1H), 9.09 (s, 1H); ^13^C NMR (151 MHz, DMSO-*d*_6_): δ 12.7 (d, ^2^*J*_F,C_ = 10.3 Hz), 12.9 (d, ^2^*J*_F,C_ = 10.4 Hz), 15.7 (d, ^5^*J*_F,C_ = 2.7 Hz), 26.2, 27.8, 29.1, 36.0, 37.9, 38.2, 45.8, 46.6, 56.6 (d, *J* = 5.3 Hz), 58.9, 68.8, 78.1 (d, ^1^*J*_F,C_ = 232.3 Hz), 114.6 (d, ^2^*J*_F,C_ = 22.0 Hz), 117.3 (d, ^2^*J*_F,C_ = 15.5 Hz), 123.8, 132.0, 133.6 (d, ^3^*J*_F,C_ = 3.2 Hz), 140.6, 150.1, 153.1, 157.1 (d, ^1^*J*_F,C_ = 244.7 Hz), 168.0 (d, ^2^*J*_F,C_ = 20.1 Hz), 168.8, 171.0; LC–MS (ESI) (90% H_2_O to 100% MeCN in 10 min, then 100% MeCN to 15 min, DAD 200–600 nm), *t*_R_ = 6.51 min, 96% purity, *m*/*z* calcd for C_29_H_36_F_2_N_4_O_4_S [M + H]^+^, 575.25; found, 575.4. HRMS (ESI) *m*/*z*: calcd for C_29_H_36_F_2_N_4_O_4_S [M + H]^+^, 575.2492; found, 575.2498.

##### (2*S*,4*R*)-1-((*S*)-2-(1-Fluorocyclopropane-1-carboxamido)-3,3-dimethylbutanoyl)-4-hydroxy-*N*-(4-(4-methylthiazol-5-yl)benzyl)pyrrolidine-2-carboxamide (**38**)

This compound was synthesized as described previously.^41^

##### 4-Bromo-5-fluoro-2-methoxybenzaldehyde (Type **40**, R = 2-OMe, 5-F)

2-Bromo-1-fluoro-4-methoxybenzene (2.05, 10 mmol) was dissolved in dry CH_2_Cl_2_ (30 mL), and it was cooled to 0 °C. Titanium(IV) chloride solution (1 M in CH_2_Cl_2_, 10 mL) was added, and it was stirred for 15 min. Subsequently, dichloromethyl methyl ether (1.1 mL, 12 mmol) was added, followed by a second portion of titanium(IV) chloride solution (1 M in CH_2_Cl_2_, 10 mL). Stirring was continued at 0 °C for 2 h. The reaction mixture was poured onto crushed ice and stirred for 5 min. It was extracted with CH_2_Cl_2_ (3 × 50 mL), and the combined organic layers were washed with brine (50 mL), dried over Na_2_SO_4_, filtered, and concentrated in vacuo. The crude product was purified by column chromatography (gradient from 10 to 20% CH_2_Cl_2_ in cyclohexane) to give the title compound as a colorless solid. Yield: 1.51 g (65%); mp 94–96 °C; *R*_f_ = 0.30 (30% CH_2_Cl_2_/petroleum ether); ^1^H NMR (600 MHz, DMSO-*d*_6_): δ 3.93 (s, 3H), 7.53 (d, *J* = 8.4 Hz, 1H), 7.62 (d, *J* = 5.3 Hz, 1H), 10.24 (s, 1H); ^13^C NMR (151 MHz, DMSO-*d*_6_) 57.2, 114.2 (d, ^2^*J*_F,C_ = 24.2 Hz), 116.60 (d, ^2^*J*_F,C_ = 23.0 Hz), 118.46, 124.50 (d, ^3^*J*_F,C_ = 5.3 Hz), 152.94 (d, ^1^*J*_F,C_ = 239.8 Hz), 158.0, 187.9; LC–MS (ESI) (90% H_2_O to 100% MeCN in 10 min, then 100% MeCN to 15 min, DAD 200–600 nm), *t*_R_ = 6.17 min, 99% purity, *m*/*z* calcd for C_8_H_7_^79^BrFO_2_ [M + H]^+^, 232.96; found, 233.1.

##### *tert*-Butyl (4-Bromobenzyl)carbamate (**41a**)

This compound was synthesized as described previously.^32^

##### *tert*-Butyl *N*-((4-Bromo-2-methylphenyl)methyl)carbamate (**41b**)

This compound was synthesized as described previously.^32^

##### *tert*-Butyl *N*-((4-Bromo-2-methoxyphenyl)methyl)carbamate (**41c**)

This compound was synthesized as described previously.^32^

##### *tert*-Butyl *N*-((4-Bromo-2-fluorophenyl)methyl)carbamate (**41d**)

This compound was synthesized as described previously.^32^

##### *tert*-Butyl *N*-((4-Bromo-3-chlorophenyl)methyl)carbamate (**41e**)

This compound was synthesized as described previously.^32^

##### *tert*-Butyl *N*-((4-Bromo-3-methylphenyl)methyl)carbamate (**41f**)

This compound was synthesized as described previously.^32^

##### *tert*-Butyl (4-Bromo-3-methoxybenzyl)carbamate (**41g**)

This compound was prepared using general procedure A and 4-bromo-3-methoxybenzaldehyde (type **40**, R = 3-OMe; 1.08 g, 5.0 mmol). The crude product was purified by column chromatography (EtOAc/*n*-hexanes 1:5) to obtain a white solid. Yield: 1.24 g (79%); mp 62–63 °C; *R*_f_ = 0.18 (EtOAc/*n*-hexanes 1:9); ^1^H NMR (400 MHz, DMSO-*d*_6_): δ 1.39 (s, 9H), 3.82 (s, 3H), 4.10 (d, *J* = 6.2 Hz, 2H), 6.76 (dd, *J* = 8.1, 1.9 Hz, 1H), 6.99 (d, *J* = 1.8 Hz, 1H), 7.42 (t, *J* = 6.2 Hz, 1H), 7.49 (d, *J* = 8.1 Hz, 1H); ^13^C NMR (101 MHz, DMSO-*d*_6_): δ 28.2, 43.1, 56.0, 77.9, 108.5, 111.30, 120.3, 132.6, 141.6, 155.2, 155.8; HRMS (ESI) *m*/*z*: [M + H]^+^ calcd for C_13_H_19_^79^BrNO_3_, 316.0543; found, 316.0546.

##### *tert*-Butyl *N*-((4-Bromo-3-fluorophenyl)methyl)carbamate (**41h**)

This compound was prepared by using general procedure A and 4-bromo-3-fluorobenzaldehyde (type **40**, R = 3-F; 1.02 g, 5.0 mmol). The crude product was purified by flash chromatography (gradient from 10 to 40% EtOAc in cyclohexane) to give a colorless solid. Yield: 0.49 g (32%); mp 92–94 °C; *R*_f_ = 0.46 (petroleum ether/EtOAc 6:1); ^1^H NMR (500 MHz, DMSO-*d*_6_): δ 1.38 (s, 9H), 4.10 (d, *J* = 6.1 Hz, 2H), 7.03 (dd, *J* = 1.9, 8.2 Hz, 1H), 7.19 (dd, *J* = 2.0, 9.9 Hz, 1H), 7.42 (t, *J* = 6.4 Hz, 1H), 7.63 (t, *J* = 7.8 Hz, 1H); ^13^C NMR (126 MHz, DMSO-*d*_6_): δ 28.3, 42.7, 78.2, 105.8 (d, ^2^*J*_F,C_ = 20.8 Hz), 115.2 (d, ^2^*J*_F,C_ = 22.3 Hz), 124.7 (d, ^3^*J*_F,C_ = 3.3 Hz), 133.4, 143.1 (d, ^3^*J*_F,C_ = 6.1 Hz), 155.9, 158.2 (d, ^1^*J*_F,C_ = 244.6 Hz); LC–MS (ESI) (90% H_2_O to 100% MeOH in 10 min, then 100% MeOH to 20 min, DAD 220–400 nm), *t*_R_ = 11.33 min, 99% purity, *m*/*z* calcd for C_12_H_16_^81^BrFNO_2_ [M + H]^+^, 306.03; found, 306.1.

##### *tert*-Butyl *N*-((4-Bromo-3-chloro-phenyl)methyl)carbamate (**41i**)

This compound was synthesized as described previously.^32^

##### *tert*-Butyl (4-Bromo-2,6-dimethylbenzyl)carbamate (**41j**)

This compound was prepared using general procedure **A** and 4-bromo-2,6-dimethylbenzaldehyde (type **40**, R = 2-Me, 6-Me; 0.99 g, 4.65 mmol). The crude product was purified by column chromatography (EtOAc/*n*-hexanes 1:7) to obtain a white solid. Yield: 0.96 g (65%); mp 91–93 °C; *R*_f_ = 0.25 (EtOAc/*n*-hexanes 1:5); ^1^H NMR (400 MHz, DMSO-*d*_6_): δ 1.37 (s, 9H), 2.30 (s, 6H), 4.11 (d, *J* = 5.4 Hz, 2H), 7.02 (t, *J* = 5.3 Hz, 1H), 7.20 (s, 2H); ^13^C NMR (101 MHz, DMSO-*d*_6_): δ 19.1, 28.2, 37.8, 77.7, 119.8, 130.2, 134.9, 140.0, 155.5; HRMS (ESI) *m*/*z*: [M + H]^+^ calcd for C_14_H_21_^79^BrN O_2_, 314.0750; found, 314.0749.

##### *tert*-Butyl (4-Bromo-2,6-dimethoxybenzyl)carbamate (**41k**)

This compound was prepared using general procedure A and 4-bromo-2,6-dimethoxybenzaldehyde (type **40**, R = 2-OMe, 6-OMe; 1.0 g, 4.08 mmol). The crude product was purified by column chromatography (EtOAc/*n*-hexanes 1:5) to obtain a white solid. Yield: 1.30 g (92%); mp 77–79 °C; *R*_f_ = 0.22 (EtOAc/*n*-hexanes 1:5); ^1^H NMR (400 MHz, DMSO-*d*_6_): δ 1.36 (s, 9H), 3.77 (s, 6H), 4.09 (d, *J* = 5.1 Hz, 2H), 6.40 (t, *J* = 5.2 Hz, 1H), 6.82 (s, 2H); ^13^C NMR (101 MHz, DMSO-*d*_6_): δ 28.3, 32.6, 56.2, 77.4, 107.5, 113.3, 121.5, 155.2, 158.9; HRMS (ESI) *m*/*z*: [M + H]^+^ calcd for C_14_H_21_^79^Br N O_4_, 346.0649; found, 346.0647.

##### *tert*-Butyl (4-Bromo-2,6-difluorobenzyl)carbamate (**41L**)

This compound was prepared using general procedure **A** and 4-bromo-2,6-difluorobenzaldehyde (type **40**, R = 2-F, 6-F; 1.0 g, 4.52 mmol). The crude product was purified by column chromatography (EtOAc/*n*-hexanes 1:7) to obtain a white solid. Yield: 0.53 g (37%); mp 61–62 °C; *R*_f_ = 0.32 (EtOAc/*n*-hexanes 1:5); ^1^H NMR (400 MHz, DMSO-*d*_6_): δ 1.35 (s, 9H), 4.12 (d, *J* = 5.5 Hz, 2H), 7.31 (t, *J* = 5.6 Hz, 1H), 7.43 (d, *J* = 6.9 Hz, 2H); ^13^C NMR (101 MHz, DMSO-*d*_6_): δ 28.2, 31.8, 78.0, 114.6 (t, ^2^*J*_F,C_ = 19.3 Hz), 115.3 (dd, ^2^*J*_F,C_ = 20.6 Hz, ^3^*J*_F,C_ = 8.8 Hz), 120.4 (t, ^2^*J*_F,C_ = 12.9 Hz), 155.2 (CO), 161.0 (dd, ^1^*J*_F,C_ = 251.7 Hz, ^3^*J*_F,C_ = 9.6 Hz); HRMS (ESI) *m*/*z*: [M + H]^+^ calcd for C_12_H_15_BrF_2_NO_2_, 322.0249; found, 322.0248.

##### *tert*-Butyl (4-Bromo-2,6-dichlorobenzyl)carbamate (**41m**)

This compound was prepared using general procedure A and 4-bromo-2,6-dichlorobenzaldehyde (type **40**, R = 2-Cl, 6-Cl; 0.27 g, 1.06 mmol). The crude product was purified by column chromatography (EtOAc/*n*-hexanes 1:9) to obtain a white solid. Yield: 0.18 g (48%); mp 110–112 °C; *R*_f_ = 0.38 (EtOAc/*n*-hexanes 1:5); ^1^H NMR (400 MHz, DMSO-*d*_6_): δ 1.37 (s, 9H), 4.32 (d, *J* = 4.9 Hz, 2H), 7.13 (t, *J* = 5.1 Hz, 1H), 7.76 (s, 2H); ^13^C NMR (101 MHz, DMSO-*d*_6_): δ 28.2, 40.2, 77.9, 121.1, 130.8, 133.3, 136.5, 155.2; HRMS (ESI) *m*/*z*: [M + H]^+^ calcd for C_12_H_15_^79^BrCl_2_NO_2_, 353.9658; found, 353.9661.

##### *tert*-Butyl (4-Bromo-2,5-dimethylbenzyl)carbamate (**41n**)

This compound was prepared using general procedure A and 4-bromo-2,5-dimethylbenzaldehyde (type **40**, R = 2-Me, 5-Me; 1.04 g, 4.89 mmol). The crude product was purified by column chromatography (EtOAc/*n*-hexanes 1:9) to obtain a white solid. Yield: 1.29 g (84%); mp 85–87 °C; *R*_f_ = 0.58 (EtOAc/*n*-hexanes 1:5); ^1^H NMR (400 MHz, DMSO-*d*_6_): δ 1.39 (s, 9H), 2.20 (s, 3H), 2.28 (s, 3H), 4.03 (d, *J* = 6.0 Hz, 2H), 7.11 (s, 1H), 7.29 (t, *J* = 6.0 Hz, 1H), 7.35 (s, 1H); ^13^C NMR (101 MHz, DMSO-*d*_6_): δ 17.6, 22.0, 28.2, 40.9, 77.8, 121.8, 129.8, 132.9, 133.9, 135.3, 137.4, 155.7; HRMS (ESI) *m*/*z*: [M + H]^+^ calcd for C_14_H_21_^79^BrNO_2_, 314.0750; found, 314.0749.

##### *tert*-Butyl *N*-((4-Bromo-2,5-dimethoxyphenyl)methyl)carbamate (**41o**)

This compound was synthesized as described previously.^32^

##### *tert*-Butyl (4-Bromo-2,5-difluorobenzyl)carbamate (**41p**)

This compound was prepared using general procedure A and 4-bromo-2,5-difluorobenzaldehyde (type **40**, R = 2-F, 5-F; 1.0 g, 4.52 mmol). The crude product was purified by column chromatography (EtOAc/*n*-hexanes 1:7) to obtain a white solid. Yield: 0.95 g (65%); mp 62–63 °C; *R*_f_ = 0.38 (EtOAc/*n*-hexanes 1:5); ^1^H NMR (400 MHz, DMSO-*d*_6_): δ 1.38 (s, 9H), 4.12 (d, *J* = 6.0 Hz, 2H), 7.23 (dd, *J* = 9.1, 6.3 Hz, 1H), 7.46 (t, *J* = 6.0 Hz, 1H), 7.68 (dd, *J* = 9.1, 5.7 Hz, 1H); ^13^C NMR (101 MHz, DMSO-*d*_6_): δ 28.1, 36.4 (d, ^4^*J*_F,C_ = 3.7 Hz), 78.3, 106.3 (dd, ^2^*J*_F,C_ = 23.5 Hz, ^3^*J*_F,C_ = 10.1 Hz), 116.2 (dd, ^2^*J*_F,C_ = 25.3 Hz, ^3^*J*_F,C_ = 5.5 Hz), 120.0 (d, ^2^*J*_F,C_ = 27.2 Hz), 128.8 (dd, ^2^*J*_F,C_ = 17.5 Hz, ^3^*J*_F,C_ = 6.5 Hz), 154.8 (dd, ^1^*J*_F,C_ = 241.75 Hz, ^4^*J*_F,C_ = 3.1 Hz), 155.1 (dd, ^1^*J*_F,C_ = 245.1 Hz, ^4^*J*_F,C_ = 2.1 Hz), 155.6; HRMS (ESI) *m*/*z*: [M – H]^−^ calcd for C_12_H_13_^79^BrF_2_NO_2_, 320.0103; found, 320.0107.

##### *tert*-Butyl (4-Bromo-2,3-dimethylbenzyl)carbamate (**41r**)

This compound was prepared using general procedure A and 4-bromo-2,3-dimethylbenzaldehyde (type **40**, R = 2-Me, 3-Me; 1.04 g, 4.90 mmol). The crude product was purified by column chromatography (EtOAc/*n*-hexanes 1:7) to obtain a white solid. Yield: 1.30 g (84%); mp 83–85 °C; *R*_f_ = 0.40 (EtOAc/*n*-hexanes 1:5); ^1^H NMR (400 MHz, DMSO-*d*_6_): δ 1.39 (s, 9H), 2.22 (s, 3H), 2.34 (s, 3H), 4.08 (d, *J* = 6.0 Hz, 2H), 6.96 (d, *J* = 8.3 Hz, 1H), 7.31 (t, *J* = 6.0 Hz, 1H), 7.39 (d, *J* = 8.3 Hz, 1H); ^13^C NMR (101 MHz, DMSO-*d*_6_): δ 15.7, 19.6, 28.2, 41.9, 77.8, 123.1, 126.7, 129.2, 135.3, 136.5, 137.3, 155.6; HRMS (ESI) *m*/*z*: [M + H]^+^ calcd for C_14_H_21_^79^BrNO_2_, 314.0750; found, 314.0749.

##### *tert*-Butyl (4-Bromo-2,3-difluorobenzyl)carbamate (**41s**)

Following general procedure A, compound **41s** was obtained from **39** and 4-bromobenzaldehyde (type **40**, R = 2-F, 3-F; 0.66 g, 3.0 mmol). The crude product was purified by column chromatography (PE/EtOAc 9:1) to obtain a colorless solid. Yield: 130 mg, (14%); mp 78–81 °C; *R*_f_ = 0.16 (petroleum ether/EtOAc 9:1); ^1^H NMR (500 MHz, DMSO-*d*_6_): δ 1.38 (s, 9H), 4.17 (d, *J* = 6.0 Hz, 2H), 7.05–7.15 (m, 1H), 7.41–7.48 (m, 1H), 7.49–7.55 (m, 1H); ^13^C NMR (126 MHz, DMSO-*d*_6_): δ 28.1, 36.8, 78.2, 107.3 (d, ^2^*J*_F,C_ = 17.5 Hz), 125.0 (d, ^3^*J*_F,C_ = 4.9 Hz), 127.8 (d, ^3^*J*_F,C_ = 4.0 Hz), 129.2 (d, ^2^*J*_F,C_ = 11.8 Hz), 146.4 (dd, ^1^*J*_F,C_ = 136.0 Hz, ^2^*J*_F,C_ = 14.0 Hz), 148.4 (dd, ^1^*J*_F,C_ = 140.3 Hz, ^2^*J*_F,C_ = 14.0 Hz), 155.6; LC–MS (ESI) (90% H_2_O to 100% MeCN in 10 min, then 100% MeCN to 15 min, DAD 200–600 nm), *t*_R_ = 7.48 min, 95% purity, *m*/*z* calcd for C_12_H_14_^79^BrF_2_NO_2_ [M – H]^−^, 320.01; found, 320.1. HRMS (ESI) *m*/*z*: calcd for C_12_H_14_^79^BrF_2_NO_2_ [M + H]^+^, 322.0071; found, 322.0069.

##### *tert*-Butyl ((4-Bromonaphthalen-1-yl)methyl)carbamate (**41v**)

This compound was prepared using general procedure A and 4-bromo-1-naphthaldehyde (type **40**, subst. phenylene = 1,4-naphthylene; 0.60 g, 2.55 mmol). The crude product was purified by column chromatography (EtOAc/*n*-hexanes 1:9) to obtain a pale-yellow solid. Yield: 0.56 g (65%); mp 73–74 °C; *R*_f_ = 0.18 (EtOAc/*n*-hexanes 1:9); ^1^H NMR (400 MHz, DMSO-*d*_6_): δ 1.40 (s, 9H), 4.58 (d, *J* = 6.0 Hz, 2H), 7.32 (d, *J* = 7.7 Hz, 1H), 7.52 (t, *J* = 6.0 Hz, 1H), 7.69 (dddd, *J* = 19.1, 8.3, 6.8, 1.3 Hz, 2H), 7.86 (d, *J* = 7.7 Hz, 1H), 8.15–8.21 (m, 2H); ^13^C NMR (101 MHz, DMSO-*d*_6_): δ 28.2, 41.2, 78.0, 120.9, 124.3, 125.6, 127.0, 127.2, 127.6, 129.6, 131.1, 132.0, 135.9, 155.7 (CO); HRMS (ESI) *m*/*z*: [M + H]^+^ calcd for C_16_H_19_^79^BrNO_2_, 336.0594; found, 336.0569.

##### *tert*-Butyl *N*-((5-Bromo-8-quinolyl)methyl)carbamate (**41w**)

This compound was prepared by using general procedure A and 5-bromoquinoline-8-carbaldehyde (type **40**, subst. phenylene = quinoline-5,8-diyl; 0.98 g, 4.16 mmol). The crude product was purified by flash chromatography on spherical silica gel (gradient from 0 to 10% acetone in petroleum ether) to give a yellowish solid. Yield: 1.02 g (73%); mp 94–96 °C; *R*_f_ = 0.31 (petroleum ether/EtOAc 8:1); ^1^H NMR (500 MHz, DMSO-*d*_6_): δ 1.41 (s, 9H), 4.75 (d, *J* = 6.1 Hz, 2H), 7.36 (t, *J* = 6.0 Hz, 1H), 7.52 (d, *J* = 7.8 Hz, 1H), 7.69–7.75 (m, 1H), 7.96 (d, *J* = 7.6 Hz, 1H), 8.48–8.54 (m, 1H), 8.97–9.02 (m, 1H); ^13^C NMR (126 MHz, DMSO-*d*_6_): δ 28.4, 78.1, 119.5, 123.1, 126.6, 127.2, 130.3, 135.2, 138.2, 146.1, 150.6, 156.0; LC–MS (ESI) (90% H_2_O to 100% MeOH in 10 min, then 100% MeOH to 20 min, DAD 220–400 nm), *t*_R_ = 11.71 min, 98% purity, *m*/*z* calcd for C_15_H_18_^81^BrN_2_O_2_ [M + H]^+^, 339.05; found, 339.1; HRMS (ESI) *m*/*z*: calcd for C_15_H_18_^79^BrN_2_O_2_ [M + H]^+^, 337.0546; found, 337.0539.

##### *tert*-Butyl (4-Bromo-5-fluoro-2-methylbenzyl)carbamate (**41x**)

This compound was prepared using general procedure A and 4-bromo-5-fluoro-2-methylbenzaldehyde (type **40**, R = 2-Me, 5-F; 0.98 g, 4.5 mmol). The crude product was purified by flash column chromatography (gradient from 10 to 20% EtOAc in petroleum ether) to obtain a white solid. Yield: 0.21 g (15%); mp 108 °C; *R*_f_ = 0.26 (petroleum ether/EtOAc 9:1); ^1^H NMR (500 MHz, DMSO-*d*_6_): δ 1.40 (s, 9H), 2.23 (s, 3H), 4.06 (d, *J* = 6.0 Hz, 2H), 7.07 (d, *J* = 10.0 Hz, 1H), 7.34–7.40 (m, 1H), 7.48 (d, *J* = 7.2 Hz, 1H); ^13^C NMR (126 MHz, DMSO-*d*_6_): δ 17.3, 28.1, 40.8, 78.0, 105.0 (d, ^2^*J*_F,C_ = 20.5 Hz), 114.7 (d, ^2^*J*_F,C_ = 22.7 Hz), 133.4 (d, ^3^*J*_F,C_ = 3.5 Hz), 134.0, 140.2 (d, ^3^*J*_F,C_ = 5.9 Hz), 155.7, 156.5 (d, ^1^*J*_F,C_ = 241.8 Hz); LC–MS (ESI) (90% H_2_O to 100% MeCN in 10 min, then 100% MeCN to 15 min, DAD 200–600 nm), *t*_R_ = 7.85 min, 96% purity, *m*/*z* calcd for C_13_H_17_^79^BrFNO_2_ [M – H]^−^, 316.03; found, 316.0. HRMS (ESI) *m*/*z*: calcd for C_13_H_17_^79^BrFNO_2_ [M + H]^+^, 318.0499; found, 318.0495.

##### *tert*-Butyl (4-Bromo-5-fluoro-2-methoxybenzyl)carbamate (**41y**)

This compound was prepared using general procedure A and 4-bromo-5-fluoro-2-methoxy-benzaldehyde (type **40**, R = 2-OMe, 5-F; 0.70 g, 3.0 mmol). The crude product was purified by flash column chromatography (gradient from 0 to 10% EtOAc in petroleum ether) to obtain a white solid. Yield: 0.84 g (84%); mp 118–220 °C; *R*_f_ = 0.38 (petroleum ether/EtOAc 9:1); ^1^H NMR (500 MHz, DMSO-*d*_6_): δ 1.39 (s, 9H), 3.81 (s, 3H), 4.04 (d, *J* = 6.1 Hz, 2H), 7.05 (d, *J* = 9.4 Hz, 1H), 7.21–7.30 (m, 2H); ^13^C NMR (126 MHz, DMSO-*d*_6_): δ 28.1, 37.9, 56.3, 78.0, 105.4 (d, ^2^*J*_F,C_ = 22.4 Hz), 114.7 (d, ^2^*J*_F,C_ = 24.6 Hz), 115.0, 129.7 (d, ^3^*J*_F,C_ = 5.6 Hz), 152.6 (d, ^1^*J*_F,C_ = 236.7 Hz), 153.1, 155.7; LC–MS (ESI) (90% H_2_O to 100% MeCN in 10 min, then 100% MeCN to 15 min, DAD 200–600 nm), *t*_R_ = 7.58 min, 99% purity, *m*/*z* calcd for C_13_H_17_^79^BrFNO_3_ [M – H]^−^, 332.03; found, 331.9. HRMS (ESI) *m*/*z*: calcd for C_13_H_17_^79^BrFNO_3_ [M + H]^+^, 334.0449; found, 334.0448.

##### *tert*-Butyl *N*-((4-Bromo-2-hydroxy-3-methoxyphenyl)methyl)carbamate (**41α**)

This compound was prepared by using general procedure A and aldehyde **66** (0.35 g, 1.5 mmol). The crude product was purified by flash chromatography on spherical silica gel (gradient from 50 to 100% CH_2_Cl_2_ in petroleum ether) to give a colorless solid. Yield: 64 mg (13%); mp 108–110 °C; *R*_f_ = 0.43 (CH_2_Cl_2_); ^1^H NMR (500 MHz, DMSO-*d*_6_): δ 1.38 (s, 9H), 3.70 (s, 3H), 4.05 (d, *J* = 6.1 Hz, 2H), 6.79 (d, *J* = 8.5 Hz, 1H), 7.00 (d, *J* = 8.2 Hz, 1H), 7.21 (t, *J* = 6.2 Hz, 1H), 9.40 (s, 1H); ^13^C NMR (126 MHz, DMSO-*d*_6_): δ 28.4, 38.5, 60.4, 78.1, 114.5, 122.4, 124.1, 128.0, 144.7, 148.6, 156.2; LC–MS (ESI) (90% H_2_O to 100% MeCN in 10 min, then 100% MeCN to 15 min, DAD 200–600 nm), *t*_R_ = 6.77 min, 97% purity, *m*/*z* calcd for C_13_H_17_^79^BrNO_4_ [M–H]^−^, 330.03; found, 330.1; HRMS (ESI) *m*/*z*: calcd for C_13_H_19_^79^BrNO_4_ [M + H]^+^, 332.0492; found, 332.0487.

##### *tert*-Butyl *N*-((4-Bromo-2,3-dimethoxyphenyl)methyl)carbamate (**41β**)

Precursor **41α** (0.49 g, 1.46 mmol) was dissolved in dry acetone (20 mL), and K_2_CO_3_ (0.40 g, 2.92 mmol) and MeI (0.18 mL, 2.92 mmol) were added. The mixture was stirred in the dark at rt for 16 h and at 60 °C for 3 h. Solid materials were removed by filtration, and the filtrate was diluted with EtOAc (50 mL). It was partitioned between NH_4_Cl (50 mL) and extracted again with EtOAc (50 mL). The combined organic layers were dried over Na_2_SO_4_, filtered, and concentrated in vacuo. The crude product was purified by flash chromatography on spherical silica gel (gradient from 2 to 20% EtOAc in petroleum ether) to give a colorless oil. Yield: 0.47 g (92%); *R*_f_ = 0.32 (CH_2_Cl_2_); ^1^H NMR (500 MHz, DMSO-*d*_6_): δ 1.38 (d, *J* = 1.3 Hz, 9H), 3.78 (dd, *J* = 1.4, 10.4 Hz, 6H), 4.09 (d, *J* = 6.0 Hz, 2H), 6.90 (d, *J* = 8.5 Hz, 1H), 7.30–7.35 (m, 2H); ^13^C NMR (126 MHz, DMSO-*d*_6_): δ 28.4, 38.0, 60.5, 60.8, 78.0, 115.1, 124.3, 127.5, 134.4, 149.8, 151.4, 155.8; LC–MS (ESI) (90% H_2_O to 100% MeCN in 10 min, then 100% MeCN to 15 min, DAD 200–600 nm), *t*_R_ = 7.42 min, 98% purity, *m*/*z* calcd for C_14_H_21_^79^BrNO_4_ [M–H]^−^, 344.05; found, 344.1; HRMS (ESI) *m*/*z*: calcd for C_14_H_21_^79^BrNO_4_ [M + H]^+^, 346.0649; found, 346.0643.

##### *tert*-Butyl *N*-((4-Bromo-2,5-dichlorophenyl)methyl)carbamate (**41γ**)

This compound was prepared by using general procedure A and aldehyde **68** (1.73 g, 6.8 mmol). The crude product was purified by flash chromatography on spherical silica gel (25 g, 15 μm, gradient from 0 to 30% THF in petroleum ether) to give a colorless solid. Yield: 0.37 g (15%); mp 240–242 °C; *R*_f_ = 0.38 (petroleum ether/THF 9:1); ^1^H NMR (600 MHz, CDCl_3_): δ 1.41 (s, 9H), 5.64 (s, 2H, CH_2_), 6.20 (t, *J* = 7.8 Hz, 1H), 7.59 (s, 1H), 7.62 (s, 1H); ^13^C NMR (151 MHz, CDCl_3_): δ 28.3, 59.5, 80.8, 122.5, 129.4, 131.3, 133.3, 134.3, 137.8, 154.3; LC–MS (ESI) (90% H_2_O to 100% MeOH in 10 min, then 100% MeOH to 20 min, DAD 220–400 nm), *t*_R_ = 12.33 min, 94% purity, *m*/*z* calcd for C_12_H_15_^79^BrCl_2_NO_2_ [M + H]^+^, 353.97; found, 354.0.

##### *tert*-Butyl *N*-((4-Bromo-3-fluoro-2-hydroxyphenyl)methyl)carbamate (**41δ**)

This compound was prepared by using general procedure A and aldehyde **69** (3.29 g, 15.0 mmol). The crude product was purified by flash chromatography (gradient from 50 to 100% CH_2_Cl_2_ in petroleum ether) to give a colorless solid. Yield: 1.59 g (33%); mp 132–134 °C; *R*_f_ = 0.20 (petroleum ether/EtOAc 8:1); ^1^H NMR (500 MHz, DMSO-*d*_6_): δ 1.38 (s, 9H), 4.07 (d, *J* = 6.2 Hz, 2H), 6.86 (d, *J* = 8.5 Hz, 1H), 7.06 (dd, *J* = 8.3, 6.2 Hz, 1H), 7.26 (t, *J* = 6.2 Hz, 1H), 10.03 (s, 1H); ^13^C NMR (126 MHz, DMSO-*d*_6_): δ 28.3, 38.3, 78.2, 106.4 (d, ^2^*J*_F,C_ = 18.5 Hz), 122.3, 124.0 (d, ^3^*J*_F,C_ = 3.5 Hz), 129.7, 143.1 (d, ^2^*J*_F,C_ = 14.8 Hz), 148.2 (d, ^1^*J*_F,C_ = 239.2 Hz), 156.1; LC–MS (ESI) (90% H_2_O to 100% MeOH in 10 min, then 100% MeOH to 20 min, DAD 220–400 nm), *t*_R_ = 11.07 min, 99% purity, *m*/*z* calcd for C_12_H_16_^81^BrFNO_3_ [M + H]^+^, 322.02; found, 322.1; HRMS (ESI) *m*/*z*: calcd for C_12_H_14_^79^BrFNO_3_ [M–H]^−^, 318.0147; found, 318.0147.

##### *tert*-Butyl *N*-((4-Bromo-2-fluoro-3-methoxyphenyl)methyl)carbamate (**41ε**)

This compound was prepared by using general procedure A and 4-bromo-2-fluoro-3-methoxybenzaldehyde (**70**, 1.00 g, 4.29 mmol). The crude product was purified by flash chromatography on spherical silica gel (gradient from 50 to 100% CH_2_Cl_2_ in petroleum ether) to give a colorless oil. Yield: 0.33 g (23%); *R*_f_ = 0.44 (petroleum ether/EtOAc 8:1); ^1^H NMR (600 MHz, DMSO-*d*_6_): δ 1.38 (s, 9H), 3.84 (s, 3H), 4.13 (d, *J* = 6.0 Hz, 2H), 6.97 (t, *J* = 7.8 Hz, 1H), 7.37–7.43 (m, 2H); ^13^C NMR (151 MHz, DMSO-*d*_6_): δ 28.3, 37.1 (d, *J* = 5.2 Hz), 61.5 (d, *J* = 4.3 Hz), 78.3, 115.0, 124.5 (d, ^3^*J*_F,C_ = 4.5 Hz), 127.9 (d, ^3^*J*_F,C_ = 4.2 Hz), 128.6 (d, ^2^*J*_F,C_ = 13.4 Hz), 144.6 (d, ^2^*J*_F,C_ = 13.2 Hz), 153.5 (d, ^1^*J*_F,C_ = 249.6 Hz), 155.8; LC–MS (ESI) (90% H_2_O to 100% MeOH in 10 min, then 100% MeOH to 20 min, DAD 220–400 nm), *t*_R_ = 11.34 min, 97% purity, *m*/*z* calcd for C_13_H_17_^79^BrFNO_3_ [M + H]^+^, 334.04; found, 334.1; HRMS (ESI) *m*/*z*: calcd for C_13_H_17_BrFNO_3_ [M + Na]^+^, 356.0268; found, 356.0263.

##### *tert*-Butyl (4-Bromo-3,5-dimethoxybenzyl)carbamate (**41ζ**)

This compound was synthesized as described previously.^32^

##### *tert*-Butyl *N*-((4-(4-Methylthiazol-5-yl)phenyl)methyl)carbamate (**42a**)

This compound was synthesized as described previously.^32^

##### *tert*-Butyl *N*-((2-Methyl-4-(4-methylthiazol-5-yl)phenyl)methyl)carbamate (**42b**)

This compound was synthesized as described previously.^32^

##### *tert*-Butyl *N*-((2-Methyl-4-(4-methylthiazol-5-yl)phenyl)methyl)carbamate (**42c**)

This compound was synthesized as described previously.^32^

##### *tert*-Butyl (2-Fluoro-4-(4-methylthiazol-5-yl)benzyl)carbamate (**42d**)

Following general procedure B, compound **42d** was obtained from **41d** (type **41**, R = 2-F; 0.618 g, 0.2 mmol). The crude product was purified by flash column chromatography (gradient from 10 to 30% EtOAc in petroleum ether) to obtain a colorless oil. Yield: 233 mg (36%); *R*_f_ = 0.22 (PE/EtOAc 4:1). ^1^H NMR (600 MHz, DMSO-*d*_6_): δ 1.40 (s, 9H), 2.46 (s, 3H), 4.21 (d, *J* = 6.0 Hz, 2H), 7.31 (d, *J* = 9.4 Hz, 2H), 7.38 (t, *J* = 7.8 Hz, 1H), 7.43 (t, *J* = 6.1 Hz, 1H), 9.02 (s, 1H); ^13^C NMR (151 MHz, DMSO-*d*_6_): δ 15.9, 28.2, 36.9, 78.0, 115.3 (d, ^2^*J*_F,C_ = 22.6 Hz), 125.0 (d, ^4^*J*_F,C_ = 3.2 Hz), 126.5 (d, ^2^*J*_F,C_ = 15.1 Hz), 129.6 (d, ^3^*J*_F,C_ = 5.2 Hz), 129.8 (d, ^4^*J*_F,C_ = 1.7 Hz), 132.0 (d, ^3^*J*_F,C_ = 8.5 Hz), 148.5, 152.0, 155.7, 159.7 (d, ^1^*J*_F,C_ = 245.5 Hz); LC–MS (ESI) (90% H_2_O to 100% MeOH in 10 min, then 100% MeOH to 20 min, DAD 220–400 nm), *t*_R_ = 11.01 min, 99% purity, *m*/*z* calcd for C_16_H_19_FN_2_O_2_S [M + H]^+^, 323.12; found, 323.1. HRMS (ESI) *m*/*z*: calcd for C_16_H_19_FN_2_O_2_S [M + H]^+^, 323.1224; found, 323.1222.

##### *tert*-Butyl (2-Chloro-4-(4-methylthiazol-5-yl)benzyl)carbamate (**42e**)

Following general procedure B, compound **42e** was obtained from **41e** (type **41**, R = 2-Cl; 961 mg, 0.3 mmol). The crude product was purified by column chromatography (gradient of PE/EtOAc 9:1 to 4:1) to obtain a colorless oil. Yield: 247 mg (49%); *R*_f_ = 0.45 (PE/EtOAc 4:1). ^1^H NMR (500 MHz, DMSO-*d*_6_): δ 1.41 (s, 9H), 2.46 (s, 3H), 4.24 (d, *J* = 6.1 Hz, 2H), 7.40 (d, *J* = 8.0 Hz, 1H), 7.44–7.49 (m, 2H), 7.53 (d, *J* = 1.8 Hz, 1H), 9.02 (s, 1H); ^13^C NMR (126 MHz, DMSO-*d*_6_): δ 15.9, 28.2, 41.0, 78.1, 127.8, 128.6, 128.9, 129.5, 131.7, 132.1, 136.7, 148.6, 152.1, 155.7; LC–MS (ESI) (90% H_2_O to 100% MeOH in 10 min, then 100% MeOH to 20 min, DAD 220–400 nm), *t*_R_ = 11.41 min, 96% purity, *m*/*z* calcd for C_16_H_19_ClN_2_O_2_S [M + H]^+^, 339.09; found, 339.1. HRMS (ESI) *m*/*z*: calcd for C_16_H_19_ClN_2_O_2_S [M + H]^+^, 339.0928; found, 339.0926.

##### *tert*-Butyl (3-Methyl-4-(4-methylthiazol-5-yl)benzyl)carbamate (**42f**)

Following general procedure B, compound **42f** was obtained from **41f** (type **41**, R = 3-Me; 90 mg, 0.3 mmol). The crude product was purified by column chromatography (gradient of PE/EtOAc 9:1 to 4:1) to obtain a colorless oil. Yield: 155 mg (15%); *R*_f_ = 0.25 (PE/EtOAc 4:1). ^1^H NMR (500 MHz, DMSO-*d*_6_): δ 1.41 (s, 9H), 2.12 (s, 3H), 2.17 (s, 3H), 4.15 (d, *J* = 6.2 Hz, 2H), 7.13 (dd, *J* = 8.0, 1.7 Hz, 1H), 7.19–7.22 (m, 2H), 7.38 (t, *J* = 5.8 Hz, 1H), 9.04 (s, 1H); ^13^C NMR (126 MHz, DMSO-*d*_6_): δ 15.2, 19.7, 28.2, 43.0, 77.8, 124.4, 128.7, 128.8, 129.5, 130.9, 136.9, 140.8, 149.0, 152.1, 155.8; LC–MS (ESI) (90% H_2_O to 100% MeOH in 10 min, then 100% MeOH to 20 min, DAD 220–400 nm), *t*_R_ = 11.12 min, 97% purity, *m*/*z* calcd for C_17_H_22_N_2_O_2_S [M + H]^+^, 319.15; found, 318.9. HRMS (ESI) *m*/*z*: calcd for C_17_H_22_N_2_O_2_S [M + H]^+^, 319.1475; found, 319.1472.

##### *tert*-Butyl (3-Methoxy-4-(4-methylthiazol-5-yl)benzyl)carbamate (**42g**)

This compound was prepared using general procedure B and **41g** (type **41**, R = 3-OMe; 0.90 g, 2.85 mmol). The crude product was purified by column chromatography (EtOAc/*n*-hexanes 1:2) to obtain a white solid. Yield: 0.15 g (16%); mp 74–75 °C; *R*_f_ = 0.20 (EtOAc/*n*-hexanes 1:2); ^1^H NMR (400 MHz, CDCl_3_): δ 1.48 (s, 9H), 2.39 (s, 3H), 3.82 (s, 3H), 4.35 (d, *J* = 6.1 Hz, 2H), 4.94 (s, 1H), 6.92 (d, *J* = 6.5 Hz, 2H), 7.25 (d, *J* = 8.2 Hz, 1H), 8.73 (s, 1H); ^13^C NMR (101 MHz, CDCl_3_): δ 16.3, 28.5, 44.8, 55.7, 79.9, 110.4, 119.4, 119.7, 127.0, 132.2, 141.4, 150.4, 151.3, 156.1, 157.3; HRMS (ESI) *m*/*z*: calcd for C_17_H_23_N_2_O_3_S [M + H]^+^, 335.1424; found, 335.1416.

##### *tert*-Butyl (3-Fluoro-4-(4-methylthiazol-5-yl)benzyl)carbamate (**42h**)

Following general procedure B, compound **42h** was obtained from **41h** (type **41**, R = 3-F; 91 mg, 0.3 mmol). The crude product was purified by column chromatography (PE/EtOAc 4:1) to obtain a yellow solid. Yield: 74 mg (77%); mp 106 °C; *R*_f_ = 0.26 (PE/EtOAc 4:1). ^1^H NMR (600 MHz, DMSO-*d*_6_): δ 1.41 (s, 9H), 2.33 (d, *J* = 1.0 Hz, 3H), 4.19 (d, *J* = 6.2 Hz, 2H), 7.16–7.21 (m, 2H), 7.43–7.50 (m, 2H, Ar-H), 9.10 (s, 1H); ^13^C NMR (151 MHz, DMSO-*d*_6_): δ 15.7, 28.2, 42.7, 78.0, 114.2 (d, ^2^*J*_F,C_ = 22.9 Hz), 117.0 (d, ^2^*J*_F,C_ = 15.3 Hz), 123.10 (d, ^3^*J*_F,C_ = 3.2 Hz), 123.7, 132.0 (d, ^3^*J*_F,C_ = 2.8 Hz), 143.9 (d, ^3^*J*_F,C_ = 7.2 Hz), 150.2, 153.1, 155.8, 158.8 (d, ^1^*J*_F,C_ = 246.6 Hz); LC–MS (ESI) (90% H_2_O to 100% MeOH in 10 min, then 100% MeOH to 20 min, DAD 220–400 nm), *t*_R_ = 10.89 min, 98% purity, *m*/*z* calcd for C_16_H_19_FN_2_O_2_S [M + H]^+^, 323.12; found, 323.2. HRMS (ESI) *m*/*z*: calcd for C_16_H_19_FN_2_O_2_S [M + H]^+^, 323.1224; found, 323.1221.

##### *tert*-Butyl (3-Chloro-4-(4-methylthiazol-5-yl)benzyl)carbamate (**42i**)

Following general procedure B, compound **42i** was obtained from **41i** (type **41**, R = 3-Cl; 96 mg, 0.3 mmol). The crude product was purified by column chromatography (gradient of PE/EtOAc 9:1 to 4:1) to obtain a colorless oil. Yield: 584 mg (58%); *R*_f_ = 0.26 (PE/EtOAc 4:1); ^1^H NMR (500 MHz, DMSO-*d*_6_): δ 1.41 (s, 9H), 2.23 (s, 3H), 4.19 (d, *J* = 6.2 Hz, 2H), 7.27–7.31 (m, 1H), 7.42–7.50 (m, 3H), 9.09 (s, 1H); ^13^C NMR (126 MHz, DMSO-*d*_6_): δ 15.5, 28.2, 42.6, 78.1, 125.9, 127.2, 128.0, 128.1, 132.7, 133.1, 143.2, 150.2, 152.9, 155.8; LC–MS (ESI) (90% H_2_O to 100% MeOH in 10 min, then 100% MeOH to 20 min, DAD 220–400 nm), *t*_R_ = 11.9 min, 96% purity, *m*/*z* calcd for C_16_H_19_ClN_2_O_2_S [M + H]^+^, 339.09; found, 339.1. HRMS (ESI) *m*/*z*: calcd for C_16_H_19_ClN_2_O_2_S [M + H]^+^, 339.0929; found, 339,0927.

##### *tert*-Butyl (2,6-Dimethyl-4-(4-methylthiazol-5-yl)benzyl)carbamate (**42j**)

This compound was prepared using general procedure B and **41j** (type **41**, R = 2-Me, 6-Me; 0.91 g, 2.88 mmol). The crude product was purified by column chromatography (EtOAc/*n*-hexanes 1:4) to obtain a pale-yellow solid. Yield: 0.49 g (51%); mp 76–77 °C; *R*_f_ = 0.18 (EtOAc/*n*-hexanes 1:4); ^1^H NMR (400 MHz, CDCl_3_): δ 1.45 (s, 9H), 2.41 (s, 6H), 2.52 (s, 3H), 4.38 (d, *J* = 4.9 Hz, 2H), 4.45 (s, 1H), 7.11 (s, 2H), 8.66 (s, 1H); ^13^C NMR (101 MHz, CDCl_3_): δ 16.3, 19.9, 28.5, 39.0, 79.6, 129.3, 131.4, 131.7, 134.4, 138.1, 148.6, 150.3, 155.8; HRMS (ESI) *m*/*z*: [M + H]^+^ calcd for C_18_H_25_N_2_O_2_S, 333.1631; found, 333.1626.

##### *tert*-Butyl (2,6-Dimethoxy-4-(4-methylthiazol-5-yl)benzyl)carbamate (**42k**)

This compound was prepared using general procedure B and **41k** (type **41**, R = 2-OMe, 6-OMe; 0.35 g, 1.0 mmol). The crude product was purified by column chromatography (EtOAc/*n*-hexanes 1:2) to obtain a pale-yellow solid. Yield: 0.21 g (57%); mp 85–87 °C; *R*_f_ = 0.18 (EtOAc/*n*-hexanes 1:2); ^1^H NMR (400 MHz, CDCl_3_): δ 1.44 (s, 9H), 2.54 (s, 3H), 3.85 (s, 6H), 4.42 (d, *J* = 5.8 Hz, 2H), 5.01 (s, 1H), 6.58 (s, 2H), 8.67 (s, 1H); ^13^C NMR (101 MHz, CDCl_3_): δ 16.3, 28.6, 33.4, 56.1, 79.1, 105.3, 114.9, 132.6, 148.8, 150.4, 155.9, 158.6; HRMS (ESI) *m*/*z*: [M + H]^+^ calcd for C_18_H_25_N_2_O_4_S, 365.1530; found, 365.1526.

##### *tert*-Butyl (2,6-Difluoro-4-(4-methylthiazol-5-yl)benzyl)carbamate (**42l**)

This compound was prepared using general procedure B and **41l** (type **41**, R = 2-F, 6-F; 0.49 g, 1.52 mmol). The crude product was purified by column chromatography (EtOAc/*n*-hexanes 1:4) to obtain a pale-yellow solid. Yield: 0.31 g (59%); mp 66–67 °C; *R*_f_ = 0.32 (EtOAc/*n*-hexanes 1:2); ^1^H NMR (400 MHz, CDCl_3_): δ 1.44 (s, 9H), 2.54 (s, 3H), 4.44 (d, *J* = 4.7 Hz, 2H), 4.95 (s, 1H), 6.94–7.02 (m, 2H), 8.71 (s, 1H); ^13^C NMR (101 MHz, CDCl_3_): δ 16.4, 28.5, 32.6, 80.0, 112.4 (dd, ^2^*J*_F,C_ = 18.9 Hz, ^4^*J*_F,C_ = 7.3 Hz), 114.2 (t, ^2^*J*_F,C_ = 18.4 Hz), 129.6 (t, ^4^*J*_F,C_ = 2.2 Hz), 133.8 (t, ^3^*J*_F,C_ = 10.7 Hz), 149.8, 151.3, 155.5, 161.5 (dd, ^1^*J*_F,C_ = 249.9 Hz, ^3^*J*_F,C_ = 9.3 Hz); HRMS (ESI) *m*/*z*: [M + H]^+^ calcd for C_16_H_19_F_2_N_2_O_2_S, 341.1130; found, 341.1121.

##### *tert*-Butyl (2,6-Dichloro-4-(4-methylthiazol-5-yl)benzyl)carbamate (**42m**)

This compound was prepared using general procedure B and **41m** (type **41**, R = 2-Cl, 6-Cl; 0.42 g, 1.18 mmol). The crude product was purified by column chromatography (EtOAc/*n*-hexanes 1:4) to obtain a pale-yellow solid. Yield: 0.23 g (52%); mp 81–82 °C; *R*_f_ = 0.33 (EtOAc/*n*-hexanes 1:2); ^1^H NMR (400 MHz, CDCl_3_): δ 1.45 (s, 9H), 2.53 (s, 3H), 4.66 (d, *J* = 5.9 Hz, 2H), 4.92 (s, 1H), 7.39 (s, 2H), 8.72 (s, 1H); ^13^C NMR (101 MHz, CDCl_3_): δ 16.3, 28.5, 40.1, 79.9, 128.8, 129.0, 133.7, 133.9, 136.4, 150.1, 151.4, 155.4; HRMS (ESI) *m*/*z*: [M + H]^+^ calcd for C_16_H_19_Cl_2_N_2_O_2_S, 373.0539; found, 373.0533.

##### *tert*-Butyl (2,5-Dimethyl-4-(4-methylthiazol-5-yl)benzyl)carbamate (**42n**)

This compound was prepared using general procedure B and **41n** (type **41**, R = 2-Me, 5-Me; 0.90 g, 2.86 mmol). The crude product was purified by column chromatography (EtOAc/*n*-hexanes 1:4) to obtain a pale-yellow solid. Yield: 0.21 g (22%); mp 85–86 °C; *R*_f_ = 0.38 (EtOAc/*n*-hexanes 1:2); ^1^H NMR (400 MHz, CDCl_3_): δ 1.47 (s, 9H), 2.14 (s, 3H), 2.27 (s, 3H), 2.29 (s, 3H), 4.31 (d, *J* = 5.8 Hz, 2H), 4.78 (s, 1H), 7.03 (s, 1H), 7.15 (s, 1H), 8.72 (s, 1H); ^13^C NMR (101 MHz, CDCl_3_): δ 15.5, 18.5, 19.7, 28.6, 42.6, 79.7, 129.9, 130.2, 133.3, 133.6, 135.6, 137.2, 149.8, 151.1, 155.9; HRMS (ESI) *m*/*z*: [M + H]^+^ calcd for C_18_H_25_N_2_O_2_S, 333.1631; found, 333.1624.

##### *tert*-Butyl *N*-((2,5-Dimethoxy-4-(4-methylthiazol-5-yl)phenyl)methyl)carbamate (**42o**)

This compound was prepared using general procedure B and **41o** (type **41**, R = 2-OMe, 5-OMe; 0.69 g, 2.0 mmol). The crude product was purified by flash chromatography on spherical silica gel (gradient from 0 to 60% EtOAc in petroleum ether) to give a colorless resin. Yield: 0.11 g (16%); *R*_f_ = 0.28 (petroleum ether/EtOAc 2:1); ^1^H NMR (500 MHz, DMSO-*d*_6_): δ 1.41 (s, 9H), 2.30 (s, 3H), 3.69 (s, 3H), 3.75 (s, 3H), 4.13 (d, *J* = 6.2 Hz, 2H), 6.89 (s, 1H), 6.95 (s, 1H), 7.24 (s, 1H), 8.98 (s, 1H); ^13^C NMR (126 MHz, DMSO-*d*_6_): δ 16.1, 28.4, 38.5, 56.1, 56.1, 78.0, 111.7, 114.1, 118.4, 126.8, 129.4, 149.6, 150.2, 150.5, 152.1, 156.0; LC–MS (ESI) (90% H_2_O to 100% MeOH in 10 min, then 100% MeOH to 20 min, DAD 220–400 nm), *t*_R_ = 11.04 min, 99% purity, *m*/*z* calcd for C_18_H_25_N_2_O_4_S [M + H]^+^, 365.15; found, 365.1; HRMS (ESI) *m*/*z*: calcd for C_18_H_25_N_2_O_4_S [M + H]^+^, 365.1530; found, 365.1527.

##### *tert*-Butyl (2,5-Difluoro-4-(4-methylthiazol-5-yl)benzyl)carbamate (**42p**)

This compound was prepared using general procedure B and **41p** (type **41**, R = 2-F, 5-F; 0.91 g, 2.82 mmol). The crude product was purified by column chromatography (EtOAc/*n*-hexanes 1:4) to obtain a pale-yellow solid. Yield: 0.47 g (49%); mp 69–71 °C; *R*_f_ = 0.38 (EtOAc/*n*-hexanes 1:2); ^1^H NMR (400 MHz, CDCl_3_): δ 1.45 (s, 9H), 2.42 (t, *J* = 1.5 Hz, 3H), 4.37 (s, 2H), 5.13 (s, 1H), 7.00–7.08 (m, 1H), 7.15 (dd, *J* = 9.7, 6.2 Hz, 1H), 8.76 (s, 1H); ^13^C NMR (101 MHz, CDCl_3_): δ 16.1, 28.5, 38.3, 80.2, 116.7 (dd, ^2^*J*_F,C_ = 25.7 Hz, ^3^*J*_F,C_ = 5.3 Hz), 118.2 (dd, ^2^*J*_F,C_ = 24.8 Hz, ^3^*J*_F,C_ = 3.0 Hz), 119.7 (dd, ^2^*J*_F,C_ = 18.1 Hz, ^3^*J*_F,C_ = 8.8 Hz), 123.5, 128.6 (dd, ^2^*J*_F,C_ = 17.4 Hz, ^3^*J*_F,C_ = 7.2 Hz), 151.5, 152.2, 155.8 (dd, ^1^*J*_F,C_ = 245.9 Hz, ^4^*J*_F,C_ = 1.6 Hz), 156.0, 156.2 (dd, ^1^*J*_F,C_ = 244.6 Hz, ^4^*J*_F,C_ = 1.8 Hz); HRMS (ESI) *m*/*z*: [M + H]^+^ calcd for C_16_H_19_F_2_N_2_O_2_S, 341.1130; found, 341.1128.

##### *tert*-Butyl (2,5-Dichloro-4-(4-methylthiazol-5-yl)benzyl)carbamate (**42q**)

Following general procedure A, compound **42q** was obtained from **50** (0.22 g, 0.8 mmol). The crude product was purified by column chromatography (gradient of PE/EtOAc 9:1 to 4:1) to obtain a white solid. Yield: 0.10 g (32%); mp 104 °C; *R*_f_ = 0.26 (PE/EtOAc 4:1). ^1^H NMR (500 MHz, DMSO-*d*_6_): δ 1.42 (s, 9H), 2.25 (s, 3H), 4.24 (d, *J* = 6.1 Hz, 2H), 7.47 (s, 1H), 7.53 (t, *J* = 6.0 Hz, 1H), 7.61 (s, 1H), 9.13 (s, 1H); ^13^C NMR (126 MHz, DMSO-*d*_6_): δ 15.5, 28.1, 41.0, 78.4, 125.8, 129.0, 130.1, 130.4, 132.1, 132.7, 139.6, 150.8, 153.5, 155.7; LC–MS (ESI) (90% H_2_O to 100% MeCN in 10 min, then 100% MeCN to 15 min, DAD 200–600 nm), *t*_R_ = 7.48 min, 99% purity, *m*/*z* calcd for C_16_H_18_Cl_2_N_2_O_2_S [M + H]^+^, 373.05; found, 373.1. HRMS (ESI) *m*/*z*: [M + H]^+^ calcd for C_16_H_18_Cl_2_N_2_O_2_S, 373.0539; found, 373.0539.

##### *tert*-Butyl (2,3-Dimethyl-4-(4-methylthiazol-5-yl)benzyl)carbamate (**42r**)

This compound was prepared using general procedure B and **41r** (type **41**, R = 2-Me, 3-Me; 0.80 g, 2.55 mmol). The crude product was purified by column chromatography (EtOAc/*n*-hexanes 1:4) to obtain a white solid. Yield: 0.36 g (42%); mp 58–60 °C; *R*_f_ = 0.35 (EtOAc/*n*-hexanes 1:2); ^1^H NMR (400 MHz, CDCl_3_): δ 1.47 (s, 9H), 2.11 (s, 3H), 2.24 (s, 3H), 2.28 (s, 3H), 4.37 (d, *J* = 5.7 Hz, 2H), 4.77 (s, 1H), 7.08 (d, *J* = 7.8 Hz, 1H), 7.14 (d, *J* = 7.9 Hz, 1H), 8.72 (s, 1H); ^13^C NMR (101 MHz, CDCl_3_): δ 15.5, 15.6, 17.3, 28.6, 43.6, 79.7, 125.6, 129.0, 130.5, 131.2, 135.9, 137.2, 137.3, 149.9, 151.1, 155.8; HRMS (ESI) *m*/*z*: [M + H]^+^ calcd for C_18_H_25_N_2_O_2_S, 333.1631; found, 333.1626.

##### *tert*-Butyl (2,3-Difluoro-4-(4-methylthiazol-5-yl)benzyl)carbamate (**42s**)

Following general procedure B, compound **42s** was obtained from bromoaryl compound **41s** (type **41**, R = 2-F, 3-F; 97 mg, 0.3 mmol). The crude product was purified by flash column chromatography (gradient of PE/EtOAc 4:1 to 1:1) to obtain a colorless oil. Yield: 0.06 g (63%); *R*_f_ = 0.15 (PE/EtOAc 4:1). ^1^H NMR (600 MHz, DMSO-*d*_6_): δ 1.40 (s, 9H), 2.35 (d, *J* = 1.2 Hz, 3H), 4.25 (d, *J* = 6.0 Hz, 2H), 7.20 (t, *J* = 7.4 Hz, 1H), 7.31 (t, *J* = 7.1 Hz, 1H), 7.50 (t, *J* = 6.0 Hz, 1H), 9.15 (s, 1H); ^13^C NMR (151 MHz, DMSO-*d*_6_): δ 15.7, 28.2, 37.0, 78.2, 119.4 (d, ^2^*J*_F,C_ = 12.0 Hz), 122.6, 123.8, 126.4 (d, ^3^*J*_F,C_ = 3.0 Hz), 129.7 (d, ^2^*J*_F,C_ = 11.6 Hz), 146.7 (dd, ^1^*J*_F,C_ = 248.3 Hz, ^2^*J*_F,C_ = 13.6 Hz), 150.8, 153.8, 155.7; LC–MS (ESI) (90% H_2_O to 100% MeCN in 10 min, then 100% MeCN to 15 min, DAD 200–600 nm), *t*_R_ = 6.79 min, 98% purity, *m*/*z* calcd for C_16_H_18_F_2_N_2_O_2_S [M + H]^+^, 341.11; found, 341.4. HRMS (ESI) *m*/*z*: calcd for C_16_H_18_F_2_N_2_O_2_S [M + H]^+^, 341.1130; found, 341.1125.

##### *tert*-Butyl (3-Fluoro-2-hydroxy-4-(4-methylthiazol-5-yl)benzyl)carbamate (**42t**)

Following general procedure A, compound **42t** was obtained from **53** (0.19 g, 0.8 mmol). The crude product was purified by flash column chromatography (gradient of PE/EtOAc 4:1 to 1:1) to obtain a white solid. Yield: 0.22 g (81%); mp 128 °C; *R*_f_ = 0.50 (PE/EtOAc 1:1). ^1^H NMR (500 MHz, DMSO-*d*_6_): δ 1.41 (s, 9H), 2.33 (s, 3H), 4.16 (d, *J* = 6.1 Hz, 2H), 6.87 (t, *J* = 7.3 Hz, 1H), 7.00 (d, *J* = 7.9 Hz, 1H), 7.30 (t, *J* = 5.9 Hz, 1H), 9.08 (s, 1H), 9.83 (s, 1H); ^13^C NMR (126 MHz, DMSO-*d*_6_): δ 15.7 (d, ^5^*J*_F,C_ = 2.5 Hz), 28.2, 38.3, 78.0, 117.7 (d, ^2^*J*_F,C_ = 13.5 Hz), 120.9, 122.7, 124.1, 130.1, 142.3 (d, ^2^*J*_F,C_ = 14.6 Hz), 148.2 (d, ^1^*J*_F,C_ = 241.2 Hz), 149.9, 152.9, 156.0; LC–MS (ESI) (90% H_2_O to 100% MeCN in 10 min, then 100% MeCN to 15 min, DAD 200–600 nm), *t*_R_ = 6.33 min, 97% purity, *m*/*z* calcd for C_16_H_19_FN_2_O_3_S [M + H]^+^, 339.12; found, 339.2. HRMS (ESI) *m*/*z*: [M + H]^+^ calcd for C_16_H_19_FN_2_O_3_S, 339.1173; found, 339.1172.

##### *tert*-Butyl (3-Fluoro-2-methoxy-4-(4-methylthiazol-5-yl)benzyl)carbamate (**42u**)

Compound **42t** (0.27 g, 0.8 mmol) and Cs_2_CO_3_ (0.65 g, 2.0 mmol) were suspended in dry DMF (10 mL). The mixture was stirred at 45 °C for 1 h, after which MeI (0.15 mL, 2.4 mmol) was added. It was further stirred at this temperature for 16 h. The suspension was filtered through a pad of Celite and washed with EtOAc (50 mL). The organic layer was washed with H_2_O (50 mL) and brine (25 mL), dried over Na_2_SO_4_, filtered, and evaporated in vacuo. The crude product was purified by flash column chromatography (gradient of PE/EtOAc 4:1 to 1:1) to obtain a colorless oil. Yield: 0.18 g (63%); *R*_f_ = 0.52 (PE/EtOAc 1:1). ^1^H NMR (600 MHz, DMSO-*d*_6_): δ 1.40 (s, 9H), 2.34 (s, 3H), 3.88 (s, 3H), 4.21 (d, *J* = 6.1 Hz, 2H), 7.12 (d, *J* = 8.1 Hz, 1H), 7.19 (t, *J* = 7.4 Hz, 1H), 7.38 (t, *J* = 6.0 Hz, 1H), 9.11 (s, 1H); ^13^C NMR (151 MHz, DMSO-*d*_6_): δ 15.8, 28.2, 37.9, 61.4 (d, ^4^*J*_F,C_ = 4.9 Hz), 78.0, 118.7 (d, ^2^*J*_F,C_ = 14.0 Hz), 123.2 (d, ^3^*J*_F,C_ = 3.5 Hz), 123.5, 125.8, 135.3, 145.0 (d, ^2^*J*_F,C_ = 11.3 Hz), 150.3, 151.9 (d, ^1^*J*_F,C_ = 247.9 Hz), 153.3, 155.8; LC–MS (ESI) (90% H_2_O to 100% MeCN in 10 min, then 100% MeCN to 15 min, DAD 200–600 nm), *t*_R_ = 6.80 min, 94% purity, *m*/*z* calcd for C_17_H_21_FN_2_O_3_S [M + H]^+^, 353.13; found, 353.2. HRMS (ESI) *m*/*z*: calcd for C_17_H_21_FN_2_O_3_S [M + H]^+^, 353.1330; found, 353.1324.

##### *tert*-Butyl ((4-(4-Methylthiazol-5-yl)naphthalen-1-yl)methyl)carbamate (**42v**)

This compound was prepared using general procedure B and **41v** (type **41**, subst. phenylene = 1,4-naphthylene; 0.52 g, 1.54 mmol). The crude product was purified by column chromatography (EtOAc/*n*-hexanes 1:4) to obtain a white solid. Yield: 0.29 g (53%); mp 53–54 °C; *R*_f_ = 0.22 (EtOAc/*n*-hexanes 1:2); ^1^H NMR (400 MHz, CDCl_3_): δ 1.48 (s, 9H), 2.26 (s, 3H), 4.84 (d, *J* = 5.8 Hz, 2H), 4.95 (s, 1H), 7.43 (d, *J* = 7.3 Hz, 1H), 7.47–7.54 (m, 2H), 7.56–7.62 (m, 1H), 7.71 (dd, *J* = 8.8, 1.2 Hz, 1H), 8.12 (d, *J* = 8.5 Hz, 1H), 8.84 (s, 1H); ^13^C NMR (101 MHz, CDCl_3_): δ 15.8, 28.6, 42.9, 79.9, 124.0, 125.2, 126.6, 126.8, 129.0, 131.7, 133.0, 135.6, 151.0, 151.8, 155.8; HRMS (ESI) *m*/*z*: [M + H]^+^ calcd for C_20_H_23_O_2_N_2_S, 355.1475; found, 355.1472.

##### *tert*-Butyl *N*-((5-(4-Methylthiazol-5-yl)-8-quinolyl)methyl)carbamate (**42w**)

This compound was prepared using general procedure B and **41w** (type **41**, subst. phenylene = quinoline-5,8-diyl; 0.74 g, 2.2 mmol). The crude product was purified by column chromatography (50% EtOAc in petroleum ether). Mass spectrometry analysis indicated the presence of a slight imine impurity. Accordingly, the product was dissolved in CH_2_Cl_2_ (20 mL), and sodium triacetoxyborohydride (0.23 g, 1.1 mmol) was added. After stirring at rt for 16 h, it was subjected to flash chromatography on spherical silica gel (gradient from 0 to 2.5% MeOH in CH_2_Cl_2_) to give a colorless resin. Yield: 0.58 g (75%); *R*_f_ = 0.58 (petroleum ether/EtOAc 1:1); ^1^H NMR (500 MHz, DMSO-*d*_6_): δ 1.42 (s, 9H), 2.16 (s, 3H), 4.84 (d, *J* = 6.2 Hz, 2H), 7.39 (t, *J* = 6.2 Hz, 1H), 7.56–7.64 (m, 1H), 7.65 (d, *J* = 2.8 Hz, 2H), 7.98–8.04 (m, 1H), 8.98 (dd, *J* = 4.2, 1.8 Hz, 1H), 9.18 (s, 1H); ^13^C NMR (126 MHz, DMSO-*d*_6_): δ 15.6, 28.4, 78.0, 122.3, 125.7, 126.7, 127.3, 127.5, 129.5, 133.8, 138.9, 145.5, 150.0, 150.5, 153.4, 156.1; LC–MS (ESI) (90% H_2_O to 100% MeOH in 10 min, then 100% MeOH to 20 min, DAD 220–400 nm), *t*_R_ = 11.03 min, 96% purity, *m*/*z* calcd for C_19_H_22_N_3_O_2_S [M + H]^+^, 356.14; found, 356.2; HRMS (ESI) *m*/*z*: calcd for C_19_H_22_N_3_O_2_S [M + H]^+^, 356.1427; found, 356.1419.

##### *tert*-Butyl (5-Fluoro-2-methyl-4-(4-methylthiazol-5-yl)benzyl)carbamate (**42x**)

Following general procedure B, compound **42x** was obtained from bromoaryl compound **41x** (type **41**, R = 2-Me, 5-F; 0.19 g, 0.6 mmol). The crude product was purified by flash column chromatography (gradient from 10 to 20% EtOAc in petroleum ether) to obtain a yellow oil. Yield: 0.12 g (58%); *R*_f_ = 0.24 (petroleum ether/EtOAc 4:1).; ^1^H NMR (500 MHz, DMSO-*d*_6_): δ 1.41 (s, 9H), 2.26 (s, 3H), 2.32 (s, 3H), 4.13 (d, *J* = 6.0 Hz, 2H), 7.07 (d, *J* = 11.1 Hz, 1H), 7.27 (d, *J* = 7.6 Hz, 1H), 7.43 (t, *J* = 5.9 Hz, 1H), 9.09 (s, 1H); ^13^C NMR (126 MHz, DMSO-*d*_6_): δ 15.6 (d, ^5^*J*_F,C_ = 2.6 Hz), 17.5, 28.2, 40.9, 78.0, 114.0 (d, ^2^*J*_F,C_ = 22.8 Hz), 116.5 (d, ^2^*J*_F,C_ = 15.0 Hz), 123.8, 131.6 (d, ^3^*J*_F,C_ = 3.4 Hz), 132.9 (d, ^3^*J*_F,C_ = 2.4 Hz), 141.1 (d, ^3^*J*_F,C_ = 7.4 Hz), 150.0, 152.9, 155.7, 157.2 (d, ^1^*J*_F,C_ = 244.2 Hz); LC–MS (ESI) (90% H_2_O to 100% MeCN in 10 min, then 100% MeCN to 15 min, DAD 200–600 nm), *t*_R_ = 7.10 min, 99% purity, *m*/*z* calcd for C_17_H_21_FN_2_O_2_S [M + H]^+^, 337.14; found, 337.2. HRMS (ESI) *m*/*z*: calcd for C_17_H_21_FN_2_O_2_S [M + H]^+^, 337.1380; found, 337.1374.

##### *tert*-Butyl (5-Fluoro-2-methoxy-4-(4-methylthiazol-5-yl)benzyl)carbamate (**42y**)

Following general procedure B, compound **42y** was obtained from **41y** (type **41**, R = 2-OMe, 5-F; 0.19 g, 0.6 mmol). The crude product was purified by flash column chromatography (gradient from 20 to 50% EtOAc in petroleum ether) to obtain a colorless oil. Yield: 0.12 g (58%); *R*_f_ = 0.51 (petroleum ether/EtOAc 1:1); ^1^H NMR (500 MHz, DMSO-*d*_6_): δ 1.41 (s, 9H), 2.36 (s, 3H), 3.82 (s, 3H), 4.13 (d, *J* = 6.3 Hz, 2H), 7.00 (d, *J* = 6.0 Hz, 1H), 7.05 (d, *J* = 10.5 Hz, 1H), 7.31 (t, *J* = 6.3 Hz, 1H), 9.10 (s, 1H); ^13^C NMR (126 MHz, DMSO-*d*_6_): δ 15.7 (d, ^5^*J*_F,C_ = 2.5 Hz), 28.2, 38.1, 56.1, 78.0, 113.3 (d, ^3^*J*_F,C_ = 2.5 Hz), 114.3 (d, ^2^*J*_F,C_ = 25.2 Hz), 117.0 (d, ^2^*J*_F,C_ = 16.5 Hz), 123.9, 130.7 (d, ^3^*J*_F,C_ = 6.8 Hz), 150.3, 152.4, 153.0 (d, ^1^*J*_F,C_ = 238.8 Hz), 153.0, 155.8; LC–MS (ESI) (90% H_2_O to 100% MeCN in 10 min, then 100% MeCN to 15 min, DAD 200–600 nm), *t*_R_ = 7.02 min, 97% purity, *m*/*z* calcd for C_17_H_21_FN_2_O_3_S [M + H]^+^, 353.13; found, 353.2. HRMS (ESI) *m*/*z*: calcd for C_17_H_21_FN_2_O_3_S [M + H]^+^, 353.1330; found, 353.1325.

##### Benzyl (2*S*,4*R*)-1-((*S*)-2-((*tert*-Butoxycarbonyl)amino)-3,3-dimethylbutanoyl)-4-hydroxypyrrolidine-2-carboxylate (**44**)^39^

Boc-Tle-OH (4.63 g, 20 mmol) was dissolved in dry DMF (18 mL), and H-Hyp-OBzl × HCl (**43**, 5.15 g, 20 mmol) was added. DIPEA (14 mL, 80 mmol) was added, followed by the addition of HATU (8.36 g, 22 mmol) after 5 min. The mixture was stirred at room temperature for 18 h. The reaction was quenched by the addition of H_2_O (150 mL) and extracted with EtOAc (3 × 150 mL). The combined organic layers were washed with saturated NaHCO_3_ (150 mL) and brine (150 mL), dried over Na_2_SO_4_, filtered, and concentrated. The residue was purified by column chromatography (petroleum ether/EtOAc 1:1) to yield **44** as a white solid. Yield: 5.91 g (68%); mp. 118–120 °C; *R*_f_ = 0.33 (petroleum ether/EtOAc 1:1); ^1^H NMR (500 MHz, DMSO-*d*_6_): δ 0.89 (s, 9H), 1.38 (s, 9H), 1.87–1.96 (m, 1H), 2.09–2.18 (m, 1H), 3.59–3.63 (m, 1H), 3.67 (dd, *J* = 10.7, 4.1 Hz, 1H), 4.15 (d, *J* = 9.4 Hz, 1H), 4.32–4.36 (m, 1H), 4.42 (t, *J* = 8.4 Hz, 1H), 5.07–5.15 (m, 2H), 5.20 (d, *J* = 3.7 Hz, 1H), 6.47 (d, *J* = 9.4 Hz, 1H), 7.29–7.39 (m, 5H); ^13^C NMR (126 MHz, DMSO-*d*_6_): δ 26.1, 28.1, 35.2, 37.2, 55.9, 57.0, 57.8, 58.2, 65.8, 68.8, 78.1, 127.8, 127.9, 128.3, 135.9, 155.3, 170.2, 171.6; LC–MS (ESI) (90% H_2_O to 100% MeOH in 10 min, then 100% MeOH to 20 min, DAD 220–400 nm), *t*_R_ = 11.26 min, 99% purity, *m*/*z* calcd for C_23_H_34_N_2_O_6_ [M + H]^+^, 435.25, found 435.3.

##### Benzyl (2*S*,4*R*)-1-((*S*)-2-(1-Cyanocyclopropane-1-carboxamido)-3,3-dimethylbutanoyl)-4-hydroxypyrrolidine-2-carboxylate (**45**)

Following general procedure C, compound **45** was obtained using Boc-protected amine **44** (1.30 mg, 3.0 mmol) and 1-cyano-1-cyclopropanecarboxylic acid (0.33 mg, 3.0 mmol). The residue was purified by flash column chromatography using a gradient from 0 to 10% MeOH in CH_2_Cl_2_ to afford **45** as a white solid. Yield: 0.81 mg (63%); mp 112–114 °C; *R*_f_ = 0.37 (CH_2_Cl_2_/MeOH 9:1); ^1^H NMR (500 MHz, DMSO-*d*_6_): δ 0.91 (s, 9H), 1.44–1.54 (m, 2H), 1.57–1.65 (m, 2H), 1.89–1.97 (m, 1H), 2.12–2.19 (m, 1H), 3.56–3.60 (m, 1H), 3.65 (dd, *J* = 10.9, 3.9 Hz, 1H), 4.31–4.36 (m, 1H), 4.46 (dd, *J* = 9.2, 7.8 Hz, 1H), 4.52 (d, *J* = 8.9 Hz, 1H), 5.13 (s, 2H), 5.21 (d, *J* = 3.9 Hz, 1H), 7.29–7.41 (m, 6H); ^13^C NMR (126 MHz, DMSO-*d*_6_): δ 13.7, 16.6, 16.7, 25.9, 36.0, 37.1, 56.2, 57.2, 57.9, 66.0, 68.7, 120.0, 127.9, 128.0, 128.3, 135.7, 164.5, 169.1, 171.4; LC–MS (ESI) (90% H_2_O to 100% MeOH in 10 min, then 100% MeOH to 20 min, DAD 220–400 nm), *t*_R_ = 10.76 min, 93% purity, *m*/*z* calcd for C_23_H_29_N_3_O_5_ [M + H]^+^, 428.23; found, 428.4.

##### (2*S*,4*R*)-1-((*S*)-2-(1-Cyanocyclopropane-1-carboxamido)-3,3-dimethylbutanoyl)-4-hydroxypyrrolidine-2-carboxylic Acid (**46**)

Compound **45** (2.17 g, 5.0 mmol) was dissolved in dry EtOH (50 mL) and treated with 10% Pd/C under H_2_ (1 atm, balloon) for 18 h. The reaction mixture was filtered through Celite and concentrated to yield a white solid. This compound was used without further purification.

##### 2,5-Dichloro-4-(4-methylthiazol-5-yl)benzoic Acid (**48**)

Following general procedure B, compound **48** was obtained from 4-bromo-2,5-dichlorobenzoic acid (**47**, 1.05 g, 4.0 mmol). The crude product was purified by column chromatography (petroleum ether/EtOAc/AcOH 1:1:0.05) to obtain a white solid. Yield: 0.36 mg (34%); mp 220–222 °C; *R*_f_ = 0.55 (petroleum ether/EtOAc/AcOH 1:1:0.05); ^1^H NMR (500 MHz, DMSO-*d*_6_): δ 2.27 (s, 3H), 7.73 (s, 1H), 7.99 (s, 1H), 9.16 (s, 1H), 13.81 (br s, 1H); ^13^C NMR (126 MHz, DMSO-*d*_6_): δ 15.5, 125.3, 130.3, 131.4, 132.1, 133.0, 134.2, 134.3, 151.3, 154.0, 165.1; LC–MS (ESI) (90% H_2_O to 100% MeCN in 10 min, then 100% MeCN to 15 min, DAD 200–600 nm), *t*_R_ = 5.14 min, 100% purity, *m*/*z* calcd for C_11_H_7_Cl_2_NO_2_S [M + H]^+^, 287.96; found, 288.0. HRMS (ESI) *m*/*z*: calcd for C_11_H_7_Cl_2_NO_2_S [M + H]^+^, 287.9647; found, 287.9646.

##### 2,5-Dichloro-*N*-methoxy-*N*-methyl-4-(4-methylthiazol-5-yl)benzamide (**49**)

Compound **48** (0.43 g, 1.5 mmol), *N*,*O*-dimethylhydroxylamine hydrochloride (0.30 g, 3.0 mmol), EDC × HCl (0.32 g, 1.65 mmol), and Et_3_N (0.23 mL, 1.65 mmol) were mixed in CH_2_Cl_2_ (15 mL) and stirred at room temperature for 16 h. Subsequently, the crude material was evaporated and subjected to flash column chromatography (gradient from 20% to 40% EtOAc in cyclohexane) to give the title compound as a colorless solid. Yield: 0.28 mg (57%); mp 104–105 °C; *R*_f_ = 0.40 (petroleum ether/EtOAc 1:1); ^1^H NMR (500 MHz, DMSO-*d*_6_): δ 2.27 (s, 3H), 3.31 (s, 3H), 3.53 (s, 3H), 7.72 (s, 1H), 7.83 (s, 1H), 9.16 (s, 1H); ^13^C NMR (126 MHz, DMSO-*d*_6_): δ 15.5, 31.8, 61.3, 125.6, 128.2, 128.6, 132.1, 132.2, 132.9, 137.1, 151.1, 153.8, 165.1; LC–MS (ESI) (90% H_2_O to 100% MeCN in 10 min, then 100% MeCN to 15 min, DAD 200–600 nm), *t*_R_ = 5.69 min, 100% purity, *m*/*z* calcd for C_13_H_12_Cl_2_N_2_O_2_S [M + H]^+^, 331.01; found, 331.1. HRMS (ESI) *m*/*z*: calcd for C_13_H_12_Cl_2_N_2_O_2_S [M + H]^+^, 331.0069; found, 331.0068.

##### 2,5-Dichloro-4-(4-methylthiazol-5-yl)benzaldehyde (**50**)

A Schlenk flask was charged with compound **49** (0.33 g, 1.0 mmol), evacuated, and refilled with argon gas. The material was dissolved in dry THF (15 mL) and cooled to 0 °C. LiAlH_4_ solution (1 M in THF, 0.5 mL) was added dropwise, and the mixture was stirred at this temperature for 1 h. Subsequently, it was cooled to −15 °C, and slowly quenched by the addition of 10% KHSO_4_ solution (50 mL). The aqueous solution was extracted with Et_2_O (50 mL), dried over Na_2_SO_4_, filtered, and concentrated in vacuo. The crude product was filtered through a small plug of silica gel, and the product was eluted with CH_2_Cl_2_. Evaporation of the solid yielded a colorless solid. Yield: 0.30 mg (92%); mp 64 °C; *R*_f_ = 0.31 (petroleum ether/EtOAc 4:1); ^1^H NMR (500 MHz, DMSO-*d*_6_): δ 2.29 (s, 3H), 7.85 (d, *J* = 0.9 Hz, 1H), 8.00 (d, *J* = 1.0 Hz, 1H), 9.20 (d, *J* = 0.9 Hz, 1H), 10.28 (d, *J* = 1.0 Hz, 1H); ^13^C NMR (126 MHz, DMSO-*d*_6_): δ 15.6, 125.2, 130.4, 132.9, 133.1, 134.1, 134.5, 137.0, 151.5, 154.3, 188.4; LC–MS (ESI) (90% H_2_O to 100% MeCN in 10 min, then 100% MeCN to 15 min, DAD 200–600 nm), *t*_R_ = 6.76 min, 100% purity, *m*/*z* calcd for C_11_H_7_Cl_2_NOS [M + H]^+^, 271.97; found, 272.0. HRMS (ESI) *m*/*z*: calcd for C_11_H_7_Cl_2_NOS [M + H]^+^, 271.9698; found, 271.9697.

##### 2-Fluoro-3-(4-methylthiazol-5-yl)phenol (**52**)

Following general procedure B, compound **52** was obtained from 3-bromo-2-fluorophenol (**51**, 0.84 mg, 5.0 mmol). The crude product was purified by column chromatography (gradient of petroleum ether/EtOAc 4:1 to 1:1) to obtain a white solid. Yield: 0.84 mg (80%); mp 168–172 °C; *R*_f_ = 0.48 (petroleum ether/EtOAc 1:1); ^1^H NMR (500 MHz, DMSO-*d*_6_): δ 2.33 (d, *J* = 1.2 Hz, 3H), 6.84 (ddd, *J* = 7.8, 6.3, 1.9 Hz, 1H), 6.99–7.10 (m, 2H), 9.08 (s, 1H), 10.07 (br s, 1H); ^13^C NMR (126 MHz, DMSO-*d*_6_): δ 15.7 (d, ^5^*J*_F,C_ = 2.7 Hz), 118.0 (d, ^3^*J*_F,C_ = 3.2 Hz), 119.7 (d, ^2^*J*_F,C_ = 12.6 Hz), 121.3, 124.1, 124.4 (d, ^3^*J*_F,C_ = 4.5 Hz), 145.6 (d, ^2^*J*_F,C_ = 12.4 Hz), 148.2 (d, ^1^*J*_F,C_ = 243.6 Hz), 150.0, 152.9; LC–MS (ESI) (90% H_2_O to 100% MeOH in 10 min, then 100% MeOH to 20 min, DAD 220–600 nm), *t*_R_ = 4.68 min, 97% purity, *m*/*z* calcd for C_10_H_8_FNOS [M + H]^+^, 210.04; found, 209.9. HRMS (ESI) *m*/*z*: calcd for C_10_H_8_FNOS [M + H]^+^, 210.0383; found, 210.0381.

##### 3-Fluoro-2-hydroxy-4-(4-methylthiazol-5-yl)benzaldehyde (**53**)

Compound **53** (0.73 g, 3.5 mmol) was dissolved in dry THF (30 mL). Et_3_N (0.97 mL, 7.0 mmol) and MgCl_2_ (0.66 g, 7.0 mmol) were added. This mixture was stirred for 10 min at room temperature, after which paraformaldehyde (0.32 g, 10.5 mmol) was introduced, and it was heated to 60 °C for 18 h. After cooling, 10% aqueous KHSO_4_ solution (50 mL) was added, and it was extracted with EtOAc (3 × 50 mL). The combined organic layers were washed with saturated aqueous NH_4_Cl solution (50 mL) and brine (50 mL), dried over Na_2_SO_4_, filtered, and concentrated in vacuo. The crude product was purified by flash chromatography using a gradient from 20 to 40% EtOAc in petroleum ether to give a colorless solid. Yield: 0.19 g (23%); mp 178 °C; *R*_f_ = 0.40 (petroleum ether/EtOAc 9:1); ^1^H NMR (500 MHz, DMSO-*d*_6_): δ 2.39 (s, 3H), 7.07 (dd, *J* = 8.2, 6.3 Hz, 1H), 7.56 (dd, *J* = 8.2, 1.3 Hz, 1H), 9.18 (s, 1H), 10.30 (s, 1H), 11.12 (s, 1H); ^13^C NMR (126 MHz, DMSO-*d*_6_): δ 16.0 (d, ^5^*J*_F,C_ = 2.9 Hz), 121.3, 123.1, 123.8 (d, ^3^*J*_F,C_ = 4.0 Hz), 124.6 (d, ^3^*J*_F,C_ = 2.8 Hz), 125.5 (d, ^2^*J*_F,C_ = 12.5 Hz), 148.6 (d, ^1^*J*_F,C_ = 245.0 Hz), 148.7 (d, ^2^*J*_F,C_ = 15.0 Hz), 151.2, 154.2, 190.3 (d, ^4^*J*_F,C_ = 3.1 Hz); LC–MS (ESI) (90% H_2_O to 100% MeCN in 10 min, then 100% MeCN to 15 min, DAD 200–600 nm), *t*_R_ = 4.08 min, 99% purity, *m*/*z* calcd for C_11_H_8_FN_2_OS [M + H]^+^, 238.03; found, 238.0. HRMS (ESI) *m*/*z*: calcd for C_11_H_8_FN_2_OS [M + H]^+^, 238.0332; found, 238.0331.

##### 1-(4-Bromo-5-fluoro-2-methylphenyl)ethan-1-one (**55c**)

4-Bromo-5-fluoro-2-methylbenzoic acid (type **54**, R = 2-Me, 5-F; 2.5 g, 10.7 mmol) was dissolved in anhydrous CH_2_Cl_2_ (50 mL). TBTU (3.61 g, 11.2 mmol) and E_t3_N (4.5 mL, 32.1 mmol) were added at 0 °C. After 1 h of stirring at 0 °C, *N*,*O*-dimethylhydroxylamine (0.78 g, 12.8 mmol) was added, the mixture was removed from the ice bath and stirred for 18 h at room temperature. H_2_O (50 mL) was added, and the phases were separated. The organic phase was washed with 10% citric acid (50 mL), saturated NaHCO_3_ (50 mL), and brine (50 mL), dried over Na_2_SO_4_, filtered, and concentrated under vacuum. The product was used in the next step without further purification. The resulting Weinreb amide was dissolved in anhydrous THF (50 mL). The solution was cooled to −20 °C, and methylmagnesium iodide (3.0 M in diethyl ether) (10.7 mL, 32.1 mmol) was added dropwise to the solution. After 1 h, the ice bath was removed, and the mixture was allowed to warm up to room temperature overnight. Then, the solution was cooled to 0 °C and quenched with saturated NH_4_Cl (20 mL). The mixture was diluted with Et_2_O, the phases were separated, and the organic phase was dried over Na_2_SO_4_, filtered, and concentrated under vacuum. The crude product was purified by column chromatography (EtOAc/*n*-hexanes 1:1) to obtain a yellow oil. Yield: 2.25 g (96%); *R*_f_ = 0.50 (EtOAc/*n*-hexanes 1:2); ^1^H NMR (400 MHz, CDCl_3_) δ, 2.47 (s, 3H), 2.56 (s, 3H), 7.43 (d, *J* = 7.7 Hz, 1H), 7.45 (d, *J* = 7.7 Hz, 1H); ^13^C NMR (101 MHz, CDCl_3_): δ 20.7, 29.4, 112.6 (d, ^2^*J*_F,C_ = 20.5 Hz), 116.9 (d, ^2^*J*_F,C_ = 22.9 Hz), 135.6 (d, ^3^*J*_F,C_ = 4.0 Hz), 136.7, 137.6 (d, ^3^*J*_F,C_ = 4.7 Hz), 156.9 (d, ^1^*J*_F,C_ = 246.7 Hz), 199.3 (d, ^4^*J*_F,C_ = 1.9 Hz); LC–MS (ESI) (solvent A: 1% CH_3_CN and 0.1% HCO_2_H in double-distilled H_2_O; solvent B: CH_3_CN. Elution gradient: 0 → 1 min, 25% B; 1 → 6 min, 25 → 98% B; 6 → 6.5 min, 98% B; 6.5 → 7 min, 98 → 25% B; 7 → 10 min, 25% B, DAD 220–400 nm), *t*_R_ = 6.84 min, 98% purity, *m*/*z* calcd for C_9_H_8_BrFO [M + H]^+^, 230.97 found, 231.0.

##### 1-(4-Bromo-5-fluoro-2-methoxyphenyl)ethan-1-one (**55d**)

4-Bromo-5-fluoro-2-methoxybenzoic acid (type **54**, R = 2-OMe, 5-F; 2.5 g, 10.0 mmol) was dissolved in anhydrous CH_2_Cl_2_ (50 mL). TBTU (3.21 g, 10.5 mmol) and Et_3_N (4.2 mL, 30.0 mmol) were added at 0 °C. After 1 h of stirring at 0 °C, *N*,*O*-dimethylhydroxylamine (0.73 g, 12.0 mmol) was added, and the mixture was removed from the ice bath and stirred for 18 h at room temperature. H_2_O (50 mL) was added and the phases were separated. The organic phase was washed with 10% citric acid (50 mL), saturated NaHCO_3_ (50 mL), brine (50 mL), dried over Na_2_SO_4_, filtered, and concentrated under vacuum. The product was used in the next step without further purification. The resulting Weinreb amide was dissolved in anhydrous THF (50 mL). The solution was cooled to −20 °C, and methylmagnesium iodide (3.0 M in diethyl ether) (10.0 mL, 30.0 mmol) was added dropwise to the solution. After 1 h, the ice bath was removed, and the mixture was allowed to warm up to room temperature overnight. Then, the solution was cooled to 0 °C and quenched with saturated NH_4_Cl (20 mL). The mixture was diluted with Et_2_O, the phases were separated, and the organic phase was dried over Na_2_SO_4_, filtered, and concentrated under vacuum. The product was used in the next step without further purification. Yield: 2.22 g (90%). *R*_f_ = 0.50 (EtOAc/*n*-hexanes 1:2); ^1^H NMR (400 MHz, CDCl_3_): δ 2.60 (s, 3H), 3.91 (s, 3H), 7.15 (d, *J* = 5.2 Hz, 1H), 7.54 (d, *J* = 8.8 Hz, 1H); ^13^C NMR (101 MHz, CDCl_3_): δ 31.8, 56.4, 114.3 (d, ^2^*J*_F,C_ = 23.0 Hz), 116.8, 117.3 (d, ^2^*J*_F,C_ = 24.9 Hz), 128.0 (d, ^3^*J*_F,C_ = 4.6 Hz), 153.5 (d, ^1^*J*_F,C_ = 241.7 Hz), 155.2 (d, ^4^*J*_F,C_ = 2.2 Hz), 197.2. LC–MS (ESI) (solvent A: 1% CH_3_CN and 0.1% HCO_2_H in double-distilled H_2_O; solvent B: CH_3_CN. Elution gradient: 0 → 1 min, 25% B; 1 → 6 min, 25 → 98% B; 6 → 6.5 min, 98% B; 6.5 → 7 min, 98 → 25% B; 7 → 10 min, 25% B, DAD 220–400 nm), *t*_R_ = 6.57 min, 91% purity, *m*/*z* calcd for C_9_H_8_BrFO_2_ 246.98 [M + H]^+^, found, 247.2.

##### (*R*,*E*)-*N*-(1-(4-Bromo-2-methylphenyl)ethylidene)-2-methylpropane-2-sulfinamide (**56a**)

Following general procedure D, compound **56a** was obtained from 1-(4-bromo-2-methylphenyl)ethanone (type **55**, R = 2-Me; 1.07 g, 5.0 mmol). The crude product was purified by flash column chromatography (gradient from 20 to 50% EtOAc in petroleum ether) to obtain a colorless oil. Yield: 0.36 g (23%); *R*_f_ = 0.55 (PE/EtOAc 2:1). ^1^H NMR (500 MHz, DMSO-*d*_6_): δ 1.19 (s, 9H), 2.35 (s, 3H), 2.62 (s, 3H), 7.41–7.55 (m, 3H); ^13^C NMR (126 MHz, DMSO-*d*_6_): δ 19.8, 21.8, 23.7, 56.1, 122.6, 128.7, 129.2, 133.4, 137.3, 139.8, 180.7; LC–MS (ESI) (90% H_2_O to 100% MeCN in 10 min, then 100% MeCN to 15 min, DAD 200–600 nm), *t*_R_ = 7.20 min, 100% purity, *m*/*z* calcd for C_13_H_18_^79^BrNOS [M + H]^+^, 316.04; found, 316.1.

##### (*R*,*E*)-*N*-(1-(4-Bromo-3-fluorophenyl)ethylidene)-2-methylpropane-2-sulfinamide (**56b**)

This compound was prepared using general procedure D and 1-(4-bromo-3-fluorophenyl)ethan-1-one (type **55**, R = 3-F; 1.08 g, 5.0 mmol). The crude product was purified by flash column chromatography (gradient from 20 to 50% EtOAc in petroleum ether) to obtain a slight yellow oil. Yield: 0.52 g (33%); *R*_f_ = 0.57 (PE/EtOAc 2:1); ^1^H NMR (500 MHz, DMSO-*d*_6_): δ 1.22 (s, 9H), 2.71 (s, 3H), 5.28 (s, 1H), 7.69 (dd, *J* = 8.4, 2.1 Hz, 1H), 7.78–7.87 (m, 2H); ^13^C NMR (126 MHz, DMSO-*d*_6_): δ 22.0, 22.2, 57.1, 111.9 (d, ^2^*J*_F,C_ = 21.4 Hz), 115.0 (d, ^2^*J*_F,C_ = 23.8 Hz), 124.5 (d, ^4^*J*_F,C_ = 3.2 Hz), 133.7, 139.9 (d, ^3^*J*_F,C_ = 6.3 Hz), 158.2 (d, ^1^*J*_F,C_ = 245.4 Hz), 174.5; LC–MS (ESI) (90% H_2_O to 100% MeCN in 10 min, then 100% MeCN to 15 min, DAD 200–600 nm), *t*_R_ = 7.19 min, 99% purity, *m*/*z* calcd for C_12_H_15_^79^BrFNOS [M + H]^+^, 320.01; found, 320.0.

##### (*R*,*E*)-*N*-(1-(4-Bromo-5-fluoro-2-methylphenyl)ethylidene)-2-methylpropane-2-sulfinamide (**56c**)

This compound was prepared using general procedure D and **55c** (type **55**, R = 2-Me, 5-F; 2.13 g, 9.27 mmol). The crude product was purified by column chromatography (EtOAc/*n*-hexanes 1:3) to obtain a yellow oil. Yield: 1.675 g (56%); *R*_f_ = 0.30 (EtOAc/*n*-hexanes 1:3); ^1^H NMR (400 MHz, CDCl_3_): δ 1.23 (s, 9H), 2.36 (s, 3H), 2.67 (s, 3H), 7.08 (d, *J* = 9.0 Hz, 1H), 7.42 (d, *J* = 6.9 Hz, 1H); ^13^C NMR (101 MHz, CDCl_3_): δ 22.1, 22.4, 23.9, 57.2, 109.9 (d, ^2^*J*_F,C_ = 20.7 Hz), 115.1 (d, ^2^*J*_F,C_ = 23.4 Hz), 132.3 (d, ^3^*J*_F,C_ = 3.7 Hz), 135.9, 141.5 (d, ^3^*J*_F,C_ = 5.9 Hz), 157.1 (d, ^1^*J*_F,C_ = 246.8 Hz), 179.1; LC–MS (ESI) (solvent A: 1% CH_3_CN and 0.1% HCO_2_H in double-distilled H_2_O; solvent B: CH_3_CN. Elution gradient: 0 → 1 min, 25% B; 1 → 6 min, 25 → 98% B; 6 → 6.5 min, 98% B; 6.5 → 7 min, 98 → 25% B; 7 → 10 min, 25% B, DAD 220–400 nm), *t*_R_ = 7.22 min, 93% purity, *m*/*z* calcd for C_13_H_17_BrFNOS [M + H]^+^, 334.03; found, 333.9.

##### (*R*,*E*)-*N*-(1-(4-Bromo-5-fluoro-2-methoxyphenyl)ethylidene)-2-methylpropane-2-sulfinamide (**56d**)

This compound was prepared using general procedure D and **55d** (type **55**, R = 2-OMe, 5-F; 2.25 g, 9.11 mmol). The crude product was purified by column chromatography (EtOAc/*n*-hexanes 1:2) to obtain a yellow oil. Yield: 0.433 g (14%); *R*_f_ = 0.40 (EtOAc/*n*-hexanes 1:1); ^1^H NMR (400 MHz, CDCl_3_): δ 1.30 (s, 9H), 2.69 (s, 3H), 3.85 (s, 3H), 7.09 (d, *J* = 5.4 Hz, 1H), 7.29–7.24 (m, 1H); ^13^C NMR (101 MHz, CDCl_3_): δ 22.1, 31.8, 55.4, 56.4, 114.3 (d, ^2^*J*_F,C_ = 22.9 Hz), 116.8 (d, ^3^*J*_F,C_ = 9.3 Hz), 117.3 (d, ^2^*J*_F,C_ = 24.8 Hz), 117.4 (d, ^3^*J*_F,C_ = 8.3 Hz), 146.6 (d, ^1^*J*_F,C_ = 240.2 Hz), 155.2 (d, ^4^*J*_F,C_ = 2.2 Hz), 197.2; LC–MS (ESI) (solvent A: 1% CH_3_CN and 0.1% HCO_2_H in double-distilled H_2_O; solvent B: CH_3_CN. Elution gradient: 0 → 1 min, 25% B; 1 → 6 min, 25 → 98% B; 6 → 6.5 min, 98% B; 6.5 → 7 min, 98 → 25% B; 7 → 10 min, 25% B, DAD 220–400 nm), *t*_R_ = 6.58 min, 79% purity, *m*/*z* calcd for C_13_H_17_BrFNO_2_S [M + Na]^+^, 372.10; found, 372.5.

##### (*R*,*E*)-*N*-(5-Bromo-2,3-dihydro-1*H*-inden-1-ylidene)-2-methylpropane-2-sulfinamide (**56e**)

Following general procedure D, compound **56e** was obtained from 5-bromo-1-indanone (type **55**, R = H, *n* = 1; 5.07 g, 24.0 mmol). The crude product was purified by flash column chromatography (gradient from 20 to 50% EtOAc in petroleum ether) to obtain a brown solid. Yield: 0.52 g (7%); mp 96–99 °C; *R*_f_ = 0.24 (PE/EtOAc 4:1). ^1^H NMR (500 MHz, DMSO-*d*_6_): δ 1.22 (s, 9H), 3.00 (ddd, *J* = 19.3, 6.8, 4.7 Hz, 1H), 3.08–3.15 (m, 2H), 3.25–3.33 (m, 1H), 7.57 (dd, *J* = 8.2, 1.7 Hz, 1H), 7.64 (d, *J* = 8.3 Hz, 1H), 7.77 (d, *J* = 1.7 Hz, 1H); ^13^C NMR (126 MHz, DMSO-*d*_6_): δ 22.0, 28.4, 31.5, 56.7, 124.5, 127.2, 129.1, 130.4, 137.7, 153.2, 182.5; LC–MS (ESI) (90% H_2_O to 100% MeCN in 10 min, then 100% MeCN to 15 min, DAD 200–600 nm), *t*_R_ = 7.29 min, 98% purity, *m*/*z* calcd for C_13_H_16_^79^BrNOS [M + H]^+^, 314.02; found, 314.0. HRMS (ESI) *m*/*z*: calcd for C_13_H_18_^79^BrNOS [M + H]^+^, 314.0209; found, 314.0204.

##### (*R*,*E*)-*N*-(5-Bromo-6-fluoro-2,3-dihydro-1*H*-inden-1-ylidene)-2-methylpropane-2-sulfinamide (**56f**)

This compound was prepared using general procedure D and 5-bromo-6-fluoro-2,3-dihydro-1*H*-inden-1-one (type **55**, R = F, *n* = 1; 1.05 g, 4.58 mmol). The crude product was purified by column chromatography (EtOAc/*n*-hexanes 1:2) to obtain a yellow oil. Yield: 0.390 g (26%); *R*_f_ = 0.23 (EtOAc/*n*-hexanes 1:2); ^1^H NMR (400 MHz, DMSO): δ 1.23 (s, 9H), 3.15–2.96 (m, 3H), 3.31–3.25 (m, 1H), 7.59 (d, *J* = 8.1 Hz, 1H) 7.93 (dd, *J* = 6.2, 1.0 Hz, 1H); ^13^C NMR (101 MHz, CDCl_3_): δ 22.5, 28.5, 32.2, 57.7, 110.2 (d, ^2^*J*_F,C_ = 23.6 Hz), 115.5 (d, ^2^*J*_F,C_ = 22.8 Hz), 130.7, 140.3 (d, ^3^*J*_F,C_ = 7.6 Hz), 146.7 (d, ^4^*J*_F,C_ = 2.8 Hz), 158.6 (d, ^1^*J*_F,C_ = 248.0 Hz), 181.6; LC–MS (ESI) (solvent A: 1% CH_3_CN and 0.1% HCO_2_H in double-distilled H_2_O; solvent B: CH_3_CN. Elution gradient: 0 → 1 min, 25% B; 1 → 6 min, 25 → 98% B; 6 → 6.5 min, 98% B; 6.5 → 7 min, 98 → 25% B; 7 → 10 min, 25% B, DAD 220–400 nm), *t*_R_ = 7.22 min, 96% purity, *m*/*z* calcd for C_13_H_15_BrFNOS [M + H]^+^, 332.01, found, 331.8.

##### (*R*,*E*)-*N*-(6-Bromo-7-fluoro-3,4-dihydronaphthalen-1(2*H*)-ylidene)-2-methylpropane-2-sulfinamide (**56g**)

This compound was prepared using general procedure D and 6-bromo-7-fluoro-3,4-dihydronaphthalen-1(2*H*)-one (type **55**, R = F, *n* = 2; 0.737 g, 3.03 mmol). The crude product was purified by flash chromatography (EtOAc/*n*-hexanes 1:2) to obtain a yellow oil. Yield: 0.145 g (14%); *R*_f_ = 0.20 (EtOAc/*n*-hexanes 1:2); ^1^H NMR (400 MHz, CDCl_3_): δ 1.33 (s, 9H), 2.12–1.87 (m, 2H), 2.82 (t, *J* = 6.0 Hz, 2H), 2.99–3.11 (m, 1H), 3.20–3.32 (m, 1H), 7.42 (dd, *J* = 6.6, 1.0 Hz, 1H), 7.84 (d, *J* = 9.7 Hz, 1H); ^13^C NMR (101 MHz, CDCl_3_): δ 24.9, 25.2, 27.3, 31.1, 53.7, 112.2 (d, ^2^*J*_F,C_ = 21.6 Hz), 114.7 (d, ^2^*J*_F,C_ = 22.3 Hz), 126.4 (d, ^4^*J*_F,C_ = 2.3 Hz), 131.5, 137.6 (d, ^3^*J*_F,C_ = 7.6 Hz), 163.5 (d, *J*_F,C_ = 246.0 Hz), 179.1; LC–MS (ESI) (solvent A: 1% CH_3_CN and 0.1% HCO_2_H in double-distilled H_2_O; solvent B: CH_3_CN. Elution gradient: 0 → 1 min, 25% B; 1 → 6 min, 25 → 98% B; 6 → 6.5 min, 98% B; 6.5 → 7 min, 98 → 25% B; 7 → 10 min, 25% B, DAD 220–400 nm), *t*_R_ = 7.56 min, 85% purity, *m*/*z* calcd for C_14_H_17_BrFNOS [M + H]^+^, 346.03; found, 345.9.

##### (*R*)-*N*-((*S*)-1-(4-Bromo-2-methylphenyl)ethyl)-2-methylpropane-2-sulfinamide (**57a**)

Following general procedure E, compound **57a** was obtained from **56a** (type **56**, R = 2-Me; 0.63 g, 2.0 mmol). The crude product was purified by flash column chromatography (gradient from 20 to 50% EtOAc in petroleum ether) to obtain a colorless oil. Yield: 0.20 g (31%); *R*_f_ = 0.15 (PE/EtOAc 1:1). ^1^H NMR (500 MHz, DMSO-*d*_6_): δ 1.09 (s, 9H), 1.41 (d, *J* = 6.7 Hz, 3H), 2.31 (s, 3H), 4.51–4.60 (m, 1H), 5.30 (d, *J* = 5.2 Hz, 1H), 7.30–7.39 (m, 3H); ^13^C NMR (126 MHz, DMSO-*d*_6_): δ 18.3, 22.6, 23.4, 50.4, 54.9, 119.5, 128.6, 128.7, 132.3, 137.5, 142.0; LC–MS (ESI) (90% H_2_O to 100% MeCN in 10 min, then 100% MeCN to 15 min, DAD 200–600 nm), *t*_R_ = 7.01 min, 100% purity, *m*/*z* calcd for C_13_H_20_^79^BrNOS [M + H]^+^, 318.05; found, 318.1.

##### (*R*)-*N*-((*S*)-1-(4-Bromo-3-fluorophenyl)ethyl)-2-methylpropane-2-sulfinamide (**57b**)

This compound was prepared using general procedure E and **56b** (type **56**, R = 3-F); 0.48 g, 1.5 mmol The crude product was purified by flash column chromatography (50% EtOAc in petroleum ether) to obtain a colorless solid. Yield: 0.31 g (63%); mp 138–139 °C; *R*_f_ = 0.20 (EtOAc/*n*-hexanes 2:1); ^1^H NMR (500 MHz, DMSO-*d*_6_): δ 1.10 (s, 9H), 1.44 (d, *J* = 6.8 Hz, 3H), 4.39–4.47 (m, 1H), 5.46 (d, *J* = 5.5 Hz, 1H), 7.16 (dd, *J* = 8.4, 2.0 Hz, 1H), 7.33–7.38 (m, 1H), 7.65 (t, *J* = 7.8 Hz, 1H); ^13^C NMR (126 MHz, DMSO-*d*_6_): δ 22.5, 24.4, 54.0, 55.0, 105.8 (d, ^2^*J*_F,C_ = 20.8 Hz), 114.8 (d, ^2^*J*_F,C_ = 22.5 Hz), 124.3, 133.1, 147.7 (d, ^3^*J*_F,C_ = 6.2 Hz), 158.0 (d, ^1^*J*_F,C_ = 244.4 Hz); LC–MS (ESI) (90% H_2_O to 100% MeCN in 10 min, then 100% MeCN to 15 min, DAD 200–600 nm), *t*_R_ = 6.66 min, 89% purity, *m*/*z* calcd for C_12_H_17_^79^BrFNOS [M + H]^+^, 322.03; found, 322.0.

##### (*R*)-*N*-((*S*)-1-(4-Bromo-5-fluoro-2-methylphenyl)ethyl)-2-methylpropane-2-sulfinamide (**57c**)

This compound was prepared using general procedure E and **56c** (type **56**, R = 2-Me, 5-F; 1 g, 3.18 mmol). The crude product was purified by column chromatography (EtOAc/*n*-hexanes 2:1) to obtain a colorless oil. Yield: 0.557 g (56%); *R*_f_ = 0.15 (EtOAc/*n*-hexanes 2:1); ^1^H NMR (400 MHz, CDCl_3_): δ 1.22 (s, 9H), 1.46 (d, *J* = 6.6 Hz, 3H), 2.33 (t, *J* = 0.9 Hz, 3H), 3.29 (d, *J* = 3.0 Hz, 1H), 4.75 (qdd, *J* = 6.6, 3.0, 1.7 Hz, 1H), 7.13 (d, *J* = 9.9 Hz, 1H), 7.33 (dd, *J* = 7.0, 0.7 Hz, 1H); ^13^C NMR (101 MHz, CDCl_3_): δ 18.3, 22.5, 24.0, 49.7, 55.7, 107.1 (d, ^2^*J*_F,C_ = 20.8 Hz), 114.2 (d, ^2^*J*_F,C_ = 22.9 Hz), 132.7 (d, ^3^*J*_F,C_ = 3.6 Hz), 134.9, 143.0 (d, ^3^*J*_F,C_ = 5.6 Hz), 157.8 (d, ^1^*J*_F,C_ = 244.7 Hz); LC–MS (ESI) (solvent A: 1% CH_3_CN and 0.1% HCO_2_H in double-distilled H_2_O; solvent B: CH_3_CN. Elution gradient: 0 → 1 min, 25% B; 1 → 6 min, 25 → 98% B; 6 → 6.5 min, 98% B; 6.5 → 7 min, 98 → 25% B; 7 → 10 min, 25% B, DAD 220–400 nm), *t*_R_ = 5.58 min, 93% purity, *m*/*z* calcd for C_13_H_19_BrFNOS [M + Na]^+^, 358.03; found, 358.1.

##### (*R*)-*N*-((*S*)-1-(4-Bromo-5-fluoro-2-methoxyphenyl)ethyl)-2-methylpropane-2-sulfinamide (**57d**)

This compound was prepared using general procedure E and **56d** (type **56**, R = 2-OMe, 5-F; 0.603 g, 1.72 mmol). The crude product was purified by column chromatography (EtOAc/*n*-hexanes 2:1) to obtain a colorless solid. Yield: 0.335 g (55%); mp 76.1–77.0 °C; *R*_f_ = 0.13 (EtOAc/*n*-hexanes 2:1); ^1^H NMR (400 MHz, CDCl_3_): δ 1.21 (s, 9H), 1.47 (d, *J* = 6.7 Hz, 3H), 3.42 (d, *J* = 4.5 Hz, 1H), 3.82 (s, 3H), 4.92–4.80 (m, 1H), 6.99 (d, *J* = 5.6 Hz, 1H, Ar-H), 7.09 (dd, *J* = 9.2, 0.5 Hz, 1H); ^13^C NMR (101 MHz, CDCl_3_): δ 22.6, 23.5, 49.1, 55.7, 56.2, 106.9 (d, ^2^*J*_F,C_ = 22.5 Hz), 114.8 (d, ^2^*J*_F,C_ = 24.8 Hz), 115.3, 133.4 (d, ^3^*J*_F,C_ = 5.4 Hz), 153.1, 153.6 (d, ^1^*J*_F,C_ = 239.9 Hz); LC–MS (ESI) (solvent A: 1% CH_3_CN and 0.1% HCO_2_H in double-distilled H_2_O; solvent B: CH_3_CN. Elution gradient: 0 → 1 min, 25% B; 1 → 6 min, 25 → 98% B; 6 → 6.5 min, 98% B; 6.5 → 7 min, 98 → 25% B; 7 → 10 min, 25% B, DAD 220–400 nm), *t*_R_ = 6.77 min, 92% purity, *m*/*z* calcd for C_13_H_19_BrFNO_2_S [M + CH_3_CN + H]^+^, 393.10; found, 393.6.

##### (*R*)-*N*-((*S*)-5-Bromo-2,3-dihydro-1*H*-inden-1-yl)-2-methylpropane-2-sulfinamide (**57e**)

Following general procedure E, compound **57e** was obtained from **56e** (type **56**, R = H, *n* = 1; 0.47 g, 1.5 mmol). The crude product was purified by flash column chromatography (gradient from 20 to 50% EtOAc in petroleum ether) to obtain a slight yellow solid. Yield: 0.32 g (68%); mp 152–154 °C; *R*_f_ = 0.28 (petroleum ether/EtOAc 1:1). ^1^H NMR (500 MHz, DMSO-*d*_6_): δ 1.16 (s, 9H), 1.92–2.01 (m, 1H), 2.39–2.47 (m, 1H), 2.72–2.81 (m, 1H), 2.91 (ddd, *J* = 16.2, 8.8, 3.2 Hz, 1H), 4.61–4.68 (m, 1H), 5.62 (d, *J* = 9.0 Hz, 1H), 7.21 (d, *J* = 8.0 Hz, 1H), 7.38 (dd, *J* = 8.0, 1.8 Hz, 1H), 7.44 (d, *J* = 1.8 Hz, 1H); ^13^C NMR (126 MHz, DMSO-*d*_6_): δ 22.8, 29.5, 35.2, 55.3, 60.8, 120.4, 126.3, 127.3, 129.0, 144.1, 145.6; LC–MS (ESI) (90% H_2_O to 100% MeCN in 10 min, then 100% MeCN to 15 min, DAD 200–600 nm), *t*_R_ = 6.74 min, 99% purity, *m*/*z* calcd for C_13_H_18_BrNOS [M + H]^+^, 316.04; found, 316.1. HRMS (ESI) *m*/*z*: calcd for C_13_H_18_BrNOS [M + H]^+^, 316.0365; found, 316.0363.

##### (*R*)-*N*-((*S*)-5-Bromo-6-fluoro-2,3-dihydro-1*H*-inden-1-yl)-2-methylpropane-2-sulfinamide (**57f**)

This compound was prepared using general procedure E and **56f** (type **56**, R = F, *n* = 1; 0.390 g, 1.18 mmol). The crude product was purified by column chromatography (EtOAc/*n*-hexanes 2:1) to obtain a colorless solid. Yield: 0.233 g (59%); mp 106–108 °C; *R*_f_ = 0.20 (EtOAc/*n*-hexanes 2:1); ^1^H NMR (400 MHz, CDCl_3_): δ 1.27 (s, 9H), 1.87–2.13 (m, 1H), 2.63–2.87 (m, 2H), 2.88–3.02 (m, 1H), 3.33 (d, *J* = 9.8 Hz, 1H), 4.67–4.95 (m, 1H), 7.07 (dd, *J* = 8.2, 1.1 Hz, 1H), 7.41 (dd, *J* = 6.3, 1.2 Hz, 1H); ^13^C NMR (101 MHz, CDCl_3_): δ 22.8, 29.5, 37.1, 56.2, 62.0, 108.7 (d, ^2^*J*_F,C_ = 22.1 Hz), 112.3 (d, ^2^*J*_F,C_ = 23.3 Hz), 129.4, 139.7 (d, ^4^*J*_F,C_ = 3.0 Hz), 145.3 (d, ^3^*J*_F,C_ = 6.6 Hz), 158.1 (d, ^1^*J*_F,C_ = 245.7 Hz); LC–MS (ESI) (solvent A: 1% CH_3_CN and 0.1% HCO_2_H in double-distilled H_2_O; solvent B: CH_3_CN. Elution gradient: 0 → 1 min, 25% B; 1 → 6 min, 25 → 98% B; 6 → 6.5 min, 98% B; 6.5 → 7 min, 98 → 25% B; 7 → 10 min, 25% B, DAD 220–400 nm), *t*_R_ = 6.72 min, 92% purity, *m*/*z* calcd for C_13_H_17_BrFNOS [M + CH_3_CN + H]^+^, 375.00; found, 374.8.

##### (*R*)-*N*-((*S*)-6-Bromo-7-fluoro-1,2,3,4-tetrahydronaphthalen-1-yl)-2-methylpropane-2-sulfinamide (**57g**)

This compound was prepared using general procedure E and **56g** (type **56**, R = F, *n* = 2; 0,510 g, 1.56 mmol). The crude product was purified by column chromatography (EtOAc/*n*-hexanes 2:1) to obtain a colorless oil. Yield: 0.264 g (52%); *R*_f_ = 0.20 (EtOAc/*n*-hexanes 2:1); ^1^H NMR (400 MHz, CDCl_3_): δ 1.28 (s, 9H), 1.98–1.77 (m, 3H), 2.32–2.38 (m, 1H), 2.82–2.62 (m, 2H), 3.35 (d, *J* = 10.1 Hz, 1H), 4.46–4.30 (m, 1H), 7.16 (dd, *J* = 9.7, 0.9 Hz, 1H), 7.29 (d, *J* = 1.0 Hz, 1H); ^13^C NMR (101 MHz, CDCl_3_): δ 20.1, 22.8, 28.2, 32.8, 55.6, 56.6, 107.8 (d, ^2^*J*_F,C_ = 21.3 Hz), 116.0 (d, ^2^*J*_F,C_ = 22.3 Hz), 133.6, 134.7 (d, ^3^*J*_F,C_ = 3.7 Hz), 139.0 (d, ^3^*J*_F,C_ = 5.6 Hz), 157.2 (d, ^1^*J*_F,C_ = 245.2 Hz); LC–MS (ESI) (solvent A: 1% CH_3_CN and 0.1% HCO_2_H in double-distilled H_2_O; solvent B: CH_3_CN. Elution gradient: 0 → 1 min, 25% B; 1 → 6 min, 25 → 98% B; 6 → 6.5 min, 98% B; 6.5 → 7 min, 98 → 25% B; 7 → 10 min, 25% B, DAD 220–400 nm), *t*_R_ = 7.02 min, 90% purity, *m*/*z* calcd for C_14_H_19_BrFNOS [M + CH_3_CN + H]^+^, 389.00; found, 388.7.

##### (*S*)-1-(4-Bromo-2-methylphenyl)ethan-1-aminium Chloride (**58a**)

Following general procedure F, compound **58a** was obtained from **57a** (type **57**, R = 2-Me; 0.16 g, 0.5 mmol). The product was obtained as a white solid. Yield: 0.12 g (100%); mp >250 °C; ^1^H NMR (500 MHz, DMSO-*d*_6_): δ 1.45 (d, *J* = 6.8 Hz, 3H), 2.35 (s, 3H), 4.50 (q, *J* = 6.8 Hz, 1H), 7.45–7.53 (m, 3H), 8.44 (s, 3H); ^13^C NMR (126 MHz, DMSO-*d*_6_): δ 18.4, 20.0, 45.7, 121.1, 127.5, 129.2, 132.9, 137.1, 138.2.

##### (*S*)-1-(4-Bromo-3-fluorophenyl)ethan-1-aminium Chloride (**58b**)

Following general procedure F, compound **58b** was obtained from **57b** (type **57**, R = 3-F; 0.29 g, 0.9 mmol). The product was obtained as a white solid. Yield: 0.23 g (100%); mp 248–250 °C; ^1^H NMR (600 MHz, DMSO-*d*_6_): δ 1.50 (d, *J* = 6.8 Hz, 3H), 4.44 (q, *J* = 6.8 Hz, 1H), 7.33 (dd, *J* = 8.3, 2.1 Hz, 1H), 7.60 (dd, *J* = 10.1, 2.1 Hz, 1H), 7.79 (t, *J* = 7.8 Hz, 1H), 8.58 (s, 3H); 13C NMR (151 MHz, DMSO-*d*_6_): δ 20.3, 49.0, 107.8 (d, ^2^*J*_F,C_ = 20.8 Hz), 115.5 (d, ^2^*J*_F,C_ = 23.4 Hz), 124.7 (d, ^4^*J*_F,C_ = 3.7 Hz), 133.8, 141.6 (d, ^3^*J*_F,C_ = 7.0 Hz), 158.1 (d, ^1^*J*_F,C_ = 244.9 Hz).

##### (*S*)-1-(4-Bromo-5-fluoro-2-methylphenyl)ethan-1-aminium Chloride (**58c**)

Following general procedure F, compound **58c** was obtained from **57c** (type **57**, R = 2-Me, 5-F; 0.5 g, 1.5 mmol). The product was obtained as a white solid and was used in the next step without further characterization. Yield: 0.40 g (100%); mp 138–140 °C.

##### (*S*)-1-(4-Bromo-5-fluoro-2-methoxyphenyl)ethan-1-aminium Chloride (**58d**)

Following general procedure F, compound **58d** was obtained from **57d** (type **57**, R = 2-OMe, 5-F; 0.320 g, 0.9 mmol). The product was obtained as a white solid and was used in the next step without further characterization. Yield: 0.26 g (100%); mp 169–172 °C.

##### (*S*)-5-Bromo-2,3-dihydro-1*H*-inden-1-aminium Chloride (**58e**)

Following general procedure F, compound **58e** was obtained from **57e** (type **57**, R = H, *n* = 1; 0.32 g, 1.0 mmol). The product was obtained as a white solid. Yield: 0.25 g (100%); mp >250 °C; ^1^H NMR (500 MHz, DMSO-*d*_6_): δ 1.94–2.08 (m, 1H), 2.40–2.48 (m, 1H), 2.83–2.94 (m, 1H), 3.02–3.12 (m, 1H), 4.66 (dd, *J* = 7.9, 5.5 Hz, 1H), 7.49 (d, *J* = 7.6 Hz, 1H), 7.53–7.61 (m, 2H), 8.53 (s, 3H); ^13^C NMR (126 MHz, DMSO-*d*_6_): δ 29.7, 30.3, 54.0, 122.2, 126.9, 127.8, 129.5, 138.7, 146.9.

##### (*S*)-5-Bromo-6-fluoro-2,3-dihydro-1*H*-inden-1-aminium Chloride (**58f**)

Following general procedure F, compound **58f** was obtained from **57f** (type **57**, R = F, *n* = 1; 0.23 g, 0.7 mmol). The product was obtained as a white solid and used in the next step without further characterization. Yield: 0.183 g (100%); mp 161–163 °C.

##### (*S*)-6-Bromo-7-fluoro-1,2,3,4-tetrahydronaphthalen-1-aminium Chloride (**58g**)

Following general procedure F, compound **58g** was obtained from **57g** (type **57**, R = F, *n* = 2; 0.24 g, 0.7 mmol). The product was obtained as a white solid and was used in the next step without further characterization. Yield: 0.193 g (100%); mp 167–169 °C.

##### *tert*-Butyl (*S*)-(1-(4-Bromo-2-methylphenyl)ethyl)carbamate (**59a**)

Following general procedure G, compound **59a** was obtained from **58a** (type **58**, R = 2-Me; 0.12 g, 0.5 mmol). The product was obtained as a white solid. Yield: 0.16 g (100%); mp 152–154 °C; *R*_f_ = 0.20 (petroleum ether/EtOAc 9:1). ^1^H NMR (500 MHz, DMSO-*d*_6_): δ 1.22 (d, *J* = 6.9 Hz, 3H), 1.34 (s, 9H), 2.30 (s, 3H), 4.70–4.78 (m, 1H), 7.26 (d, *J* = 8.3 Hz, 1H), 7.31–7.39 (m, 2H), 7.45 (d, *J* = 7.9 Hz, 1H); ^13^C NMR (126 MHz, DMSO-*d*_6_): δ 18.1, 21.4, 28.2, 45.6, 77.7, 119.0, 127.1, 128.8, 132.1, 136.9, 143.4, 154.6; LC–MS (ESI) (90% H_2_O to 100% MeCN in 10 min, then 100% MeCN to 15 min, DAD 200–600 nm), *t*_R_ = 8.05 min, 100% purity, *m*/*z* calcd for C_14_H_20_BrNO_2_ [M + H]^+^, 314.07; found, 314.1.

##### *tert*-Butyl (*S*)-(1-(4-Bromo-3-fluorophenyl)ethyl)carbamate (**59b**)

This compound was prepared using general procedure G and **58b** (type **58**, R = 3-F; 0.23 g, 0.9 mmol). The product was obtained as a white solid. Yield: 0.28 g (100%); mp 124–126 °C; *R*_f_ = 0.9 (CH_2_Cl_2_/MeOH 9:1); ^1^H NMR (500 MHz, DMSO-*d*_6_): δ 1.28 (d, *J* = 7.0 Hz, 3H), 1.36 (s, 9H), 4.57–4.65 (m, 1H), 7.10 (dd, *J* = 8.2, 1.9 Hz, 1H), 7.24–7.30 (m, 1H), 7.43 (d, *J* = 8.0 Hz, 1H), 7.63 (t, *J* = 7.8 Hz, 1H); ^13^C NMR (126 MHz, DMSO-*d*_6_): δ 22.4, 28.1, 48.9, 77.9, 105.4 (d, ^2^*J*_F,C_ = 20.7 Hz), 114.0 (d, ^2^*J*_F,C_ = 22.4 Hz), 123.5 (d, ^4^*J*_F,C_ = 3.2 Hz), 133.1, 148.2 (d, ^3^*J*_F,C_ = 7.9 Hz), 154.7, 158.1 (d, ^1^*J*_F,C_ = 244.4 Hz); LC–MS (ESI) (90% H_2_O to 100% MeCN in 10 min, then 100% MeCN to 15 min, DAD 200–600 nm), *t*_R_ = 7.75 min, 98% purity, *m*/*z* calcd for C_13_H_17_^79^BrFNO_2_ [M – H]^+^, 316.03; found, 316.0.

##### *tert*-Butyl (*S*)-(1-(4-Bromo-5-fluoro-2-methylphenyl)ethyl)carbamate (**59c**)

This compound was prepared using general procedure G and **58c** (type **58**, R = 2-Me, 5-F; 0.504 g, 1.88 mmol). The product was obtained as a white solid. Yield: 0.615 g (98%); mp 79–80 °C; *R*_f_ = 0.9 (CH_2_Cl_2_/MeOH 9:1); ^1^H NMR (400 MHz, CDCl_3_): δ 1.35 (d, *J* = 6.7 Hz, 3H). 1.41 (s, 9H), 2.32 (s, 3H), 4.74–4.90 (m, 2H), 7.04 (d, *J* = 9.8 Hz, 1H), 7.31 (dd, *J* = 7.0, 0.8 Hz, 1H); ^13^C NMR (101 MHz, CDCl_3_): δ 18.1, 21.7, 27.4, 31.2, 79.8, 106.6 (d, ^2^*J*_F,C_ = 21.5 Hz), 112.5 (d, ^2^*J*_F,C_ = 22.8 Hz), 132.2, 135.0, 146.8, 154.8, 157.8 (d, ^1^*J*_F,C_ = 244.6 Hz); LC–MS (ESI) (solvent A: 1% CH_3_CN and 0.1% HCOOH in double-distilled H_2_O; solvent B: CH_3_CN. Elution gradient: 0 → 1 min, 25% B; 1 → 6 min, 25 → 98% B; 6 → 6.5 min, 98% B; 6.5 → 7 min, 98 → 25% B; 7 → 10 min, 25% B, DAD 220–400 nm), *t*_R_ = 7.61 min, 96% purity, *m*/*z* calcd for C_14_H_19_BrFNO_2_ [M + H]^+^, 332.07; found, 332.1.

##### *tert*-Butyl (*S*)-(1-(4-Bromo-5-fluoro-2-methoxyphenyl)ethyl)carbamate (**59d**)

This compound was prepared using general procedure G and **58d** (type **58**, R = 2-OMe, 5-F; 0.240 g, 0.71 mmol). The product was obtained as a white solid. Yield: 0.239 g (97%); mp 86–88 °C; *R*_f_ = 0.9 (CH_2_Cl_2_/MeOH 9:1); ^1^H NMR (400 MHz, CDCl_3_): δ 1.36 (d, *J* = 6.9 Hz, 3H), 1.42 (s, 9H), 3.83 (s, 3H), 4.86–4.97 (m, 1H), 5.02–5.14 (m, 1H), 7.04–6.93 (m, 2H); ^13^C NMR (101 MHz, CDCl_3_): δ 21.4, 28.4, 46.8, 56.1, 79.6, 106.5 (d, ^2^*J*_F,C_ = 22.7 Hz), 114.4 (d, ^2^*J*_F,C_ = 24.4 Hz), 115.4 (d, ^3^*J*_F,C_ = 9.01 Hz), 133.7, 153.0 (d, ^4^*J*_F,C_ = 2.4 Hz), 153.6 (d, ^1^*J*_F,C_ = 240.3 Hz), 154.9; LC–MS (ESI) (solvent A: 1% CH_3_CN and 0.1% HCO_2_H in double-distilled H_2_O; solvent B: CH_3_CN. Elution gradient: 0 → 1 min, 25% B; 1 → 6 min, 25 → 98% B; 6 → 6.5 min, 98% B; 6.5 → 7 min, 98 → 25% B; 7 → 10 min, 25% B, DAD 220–400 nm), *t*_R_ = 7.47 min, 90% purity, *m*/*z* calcd for C_14_H_19_BrFNO_3_ [M + H]^+^, 348.06; found, 348.02.

##### *tert*-Butyl (*S*)-(5-Bromo-2,3-dihydro-1*H*-inden-1-yl)carbamate (**59e**)

Following general procedure G, compound **59e** was obtained from **58e** (type **58**, R = H, *n* = 1; 0.25 g, 1.0 mmol). The product was obtained as a white solid. Yield: 0.31 g (100%); mp 122–124 °C; *R*_f_ = 0.33 (petroleum ether/EtOAc 9:1); ^1^H NMR (500 MHz, DMSO-*d*_6_): δ 1.42 (s, 9H), 1.76–1.86 (m, 1H), 2.28–2.37 (m, 1H), 2.70–2.79 (m, 1H), 2.89 (ddd, *J* = 16.1, 8.7, 3.1 Hz, 1H), 4.89–4.96 (m, 1H), 7.12 (d, *J* = 8.1 Hz, 1H), 7.26 (d, *J* = 8.5 Hz, 1H), 7.35 (d, *J* = 8.0 Hz, 1H), 7.41 (d, *J* = 1.8 Hz, 1H); ^13^C NMR (126 MHz, DMSO-*d*_6_): δ 28.2, 29.3, 32.5, 54.6, 77.8, 120.2, 125.6, 127.3, 129.1, 144.1, 145.6, 155.5; LC–MS (ESI) (90% H_2_O to 100% MeCN in 10 min, then 100% MeCN to 15 min, DAD 200–600 nm), *t*_R_ = 8.09 min, 100% purity, *m*/*z* calcd for C_14_H_18_BrNO_2_ [M + Na]^+^, 334.04; found, 334.1. HRMS (ESI) *m*/*z*: calcd for C_14_H_18_BrNO_2_ [M + H]^+^, 312.0594; found, 312.0589.

##### *tert*-Butyl (*S*)-(5-Bromo-6-fluoro-2,3-dihydro-1*H*-inden-1-yl)carbamate (**59f**)

This compound was prepared using general procedure G and **58f** (type **58**, R = F, *n* = 1; 0.236 g, 0.85 mmol). The product was obtained as a white solid. Yield: 0.285 g (97%); mp 80–82 °C; *R*_f_ = 0.9 (CH_2_Cl_2_/MeOH 9:1); ^1^H NMR (400 MHz, CDCl_3_): δ 1.49 (s, 9H), 1.77–1.85 (m, 1H), 2.66–2.49 (m, 1H), 3.06–2.65 (m, 2H), 4.73 (d, *J* = 8.9 Hz, 1H), 5.13 (q, *J* = 8.2 Hz, 1H), 7.22–7.01 (m, 1H), 7.32–7.44 (m, 1H); ^13^C NMR (101 MHz, CDCl_3_): δ 28.4, 29.3, 34.7, 55.6, 79.9, 108.3 (d, ^2^*J*_F,C_ = 22.0 Hz), 112.1 (d, ^2^*J*_F,C_ = 23.2 Hz), 129.2, 139.8 (d, ^3^*J*_F,C_ = 3.0 Hz), 145.9, 155.5, 158.2 (d, ^1^*J*_F,C_ = 245.7 Hz); LC–MS (ESI) (solvent A: 1% CH_3_CN and 0.1% HCO_2_H in double-distilled H_2_O; solvent B: CH_3_CN. Elution gradient: 0 → 1 min, 25% B; 1 → 6 min, 25 → 98% B; 6 → 6.5 min, 98% B; 6.5 → 7 min, 98 → 25% B; 7 → 10 min, 25% B, DAD 220–400 nm), *t*_R_ = 7.58 min, 94% purity, *m*/*z* calcd for C_14_H_17_BrFNO [M + Na]^+^, 352.03; found, 351.9.

##### *tert*-Butyl (*S*)-(6-Bromo-7-fluoro-1,2,3,4-tetrahydronaphthalen-1-yl)carbamate (**59g**)

This compound was prepared using general procedure G and **58g** (type **58**, R = F, *n* = 2; 0.251 g, 0.94 mmol). The product was obtained as a white solid. Yield: 0.254 g (82%); mp 79.7–80.6 °C; *R*_f_ = 0.9 (CH_2_Cl_2_/MeOH 9:1); ^1^H NMR (400 MHz, CDCl_3_): δ 1.48 (s, 9H), 1.75–1.65 (m, 1H), 1.85–1.75 (m, 2H), 2.11–1.99 (m, 1H), 2.71 (q, *J* = 6.8 Hz, 2H), 4.81–4.69 (m, 2H), 7.12 (d, *J* = 9.5 Hz, 1H), 7.25 (d, *J* = 1.0 Hz, 1H); ^13^C NMR (101 MHz, CDCl_3_): δ 20.2, 28.4, 28.5, 30.2, 48.5, 79.8, 107.5 (d, ^2^*J*_F,C_ = 20.9 Hz), 115.8 (d, ^2^*J*_F,C_ = 22.2 Hz), 133.5, 134.5 (d, ^3^*J*_F,C_ = 3.9 Hz), 139.0, 155.5, 157.4 (d, ^1^*J*_F,C_ = 245.4 Hz); LC–MS (ESI) (solvent A: 1% CH_3_CN and 0.1% HCO_2_H in double-distilled H_2_O; Solvent B: CH_3_CN. Elution gradient: 0 → 1 min, 25% B; 1 → 6 min, 25 → 98% B; 6 → 6.5 min, 98% B; 6.5 → 7 min, 98 → 25% B; 7 → 10 min, 25% B, DAD 220–400 nm), *t*_R_ = 7.87 min, 95% purity, *m*/*z* calcd for C_15_H_19_BrFNO_2_ [M + Na]^+^, 366.06; found, 366.1.

##### *tert*-Butyl (*S*)-(1-(2-Methyl-4-(4-methylthiazol-5-yl)phenyl)ethyl)carbamate (**60a**)

Following general procedure B, compound **60a** was obtained from **59a** (type **59**, R = 2-Me; 0.16 g, 0.5 mmol). The crude product was purified by flash column chromatography (gradient from 20 to 50% EtOAc in petroleum ether) to obtain a colorless oil. Yield: 0.04 g (22%); *R*_f_ = 0.18 (PE/EtOAc 4:1). ^1^H NMR (600 MHz, DMSO-*d*_6_): δ 1.27 (d, *J* = 7.0 Hz, 3H), 1.36 (s, 9H), 2.36 (s, 3H), 2.45 (s, 3H), 4.79–4.86 (m, 1H), 7.23 (d, *J* = 1.9 Hz, 1H), 7.30 (dd, *J* = 8.1, 2.0 Hz, 1H), 7.42 (d, *J* = 8.0 Hz, 1H), 7.47 (d, *J* = 8.0 Hz, 1H), 8.96 (s, 1H); ^13^C NMR (151 MHz, DMSO-*d*_6_): δ 16.0, 18.5, 21.7, 28.3, 45.8, 77.7, 125.5, 126.6, 129.4, 130.4, 131.2, 134.8, 143.8, 147.6, 151.3, 154.7; LC–MS (ESI) (90% H_2_O to 100% MeCN in 10 min, then 100% MeCN to 15 min, DAD 200–600 nm), *t*_R_ = 7.39 min, 98% purity, *m*/*z* calcd for C_18_H_24_N_2_O_2_S [M + H]^+^, 333.16; found, 333.2.

##### *tert*-Butyl (*S*)-(1-(3-Fluoro-4-(4-methylthiazol-5-yl)phenyl)ethyl)carbamate (**60b**)

This compound was prepared using general procedure B and **59b** (type **59**, R = 3-F; 0.30 g, 0.9 mmol). The crude product was purified by flash column chromatography (gradient from 20 to 50% EtOAc in petroleum ether) to obtain a colorless solid. Yield: 0.12 g (38%); mp 112–114 °C; *R*_f_ = 0.14 (petroleum ether/EtOAc 4:1); ^1^H NMR (600 MHz, DMSO-*d*_6_): δ 1.33 (s, 3H), 1.38 (s, 9H), 2.33 (s, 3H), 4.65–4.72 (m, 1H), 7.23 (dd, *J* = 8.0, 1.6 Hz, 1H), 7.27 (d, *J* = 11.5 Hz, 1H), 7.42–7.49 (m, 2H), 9.09 (s, 1H); ^13^C NMR (151 MHz, DMSO-*d*_6_): δ 15.7, 22.6, 28.2, 49.1, 77.9, 113.3 (d, ^2^*J*_F,C_ = 22.8 Hz), 116.8 (d, ^2^*J*_F,C_ = 15.7 Hz), 122.2 (d, ^4^*J*_F,C_ = 2.2 Hz), 123.8, 131.9, 149.1, 150.1, 153.1, 154.8, 158.8 (d, ^2^*J*_F,C_ = 246.5 Hz); LC–MS (ESI) (90% H_2_O to 100% MeCN in 10 min, then 100% MeCN to 15 min, DAD 200–600 nm), *t*_R_ = 7.11 min, 96% purity, *m*/*z* calcd for C_17_H_21_FN_2_O_2_S [M – H]^+^, 337.14; found, 337.2.

##### *tert*-Butyl (*S*)-(1-(5-Fluoro-2-methyl-4-(4-methylthiazol-5-yl)phenyl)ethyl)carbamate (**60c**)

This compound was prepared using general procedure B and **59c** (type **59**, R = 2-Me, 5-F; 0.610 g, 1.83 mmol). The crude product was purified by column chromatography (EtOAc/*n*-hexanes 1:2) to obtain a waxy solid. Yield: 0.210 g (33%); *R*_f_ = 0.4 (EtOAc/*n*-hexanes 1:1); ^1^H NMR (400 MHz, CDCl_3_): δ 1.39–1.49 (m, 12H), 2.37 (s, 3H), 2.43 (d, *J* = 1.4 Hz, 3H), 4.75–4.89 (m, 1H), 4.90–5.00 (m, 1H), 7.03–7.21 (m, 2H), 8.76 (s, 1H); ^13^C NMR (101 MHz, CDCl_3_): δ 16.0, 18.2, 21.8, 28.4, 30.0, 79.9, 112.1 (d, ^2^*J*_F,C_ = 23.3 Hz), 124.6, 128.0, 128.4, 130.9, 133.9, 150.8, 151.6, 154.9, 158.5 (d, ^1^*J*_F,C_ = 247.5 Hz); LC–MS (ESI) (solvent A: 1% CH_3_CN and 0.1% HCO_2_H in double-distilled H_2_O; solvent B: CH_3_CN. Elution gradient: 0 → 1 min, 25% B; 1 → 6 min, 25 → 98% B; 6 → 6.5 min, 98% B; 6.5 → 7 min, 98 → 25% B; 7 → 10 min, 25% B, DAD 220–400 nm), *t*_R_ = 7.03 min, 96% purity, *m*/*z* calcd for C_18_H_23_FN_2_O_2_S [M + H]^+^, 351.1; found, 350.6.

##### *tert*-Butyl (*S*)-(1-(5-Fluoro-2-methoxy-4-(4-methylthiazol-5-yl)phenyl)ethyl)carbamate (**60d**)

This compound was prepared using general procedure B and **59d** (type **59**, R = 2-OMe, 5-F; 0.263 g, 0.75 mmol). The crude product was purified by column chromatography (EtOAc/*n*-hexanes 1:2) to obtain a waxy solid. Yield: 0.156 g (56%). *R*_f_ = 0.40 (EtOAc/*n*-hexanes 1:1); ^1^H NMR (400 MHz, CDCl_3_): δ 1.33–1.52 (m, 12H), 2.45 (d, *J* = 1.4 Hz, 3H), 3.85 (s, 3H), 4.91–5.03 (m, 1H), 5.11–5.25 (m, 1H), 6.79 (d, *J* = 5.9 Hz, 1H), 7.05 (d, *J* = 10.1 Hz, 1H), 8.77 (s, 1H); ^13^C NMR (101 MHz, CDCl_3_): δ 16.1, 21.6, 28.4, 46.9, 56.0, 79.6, 113.7, 114.4 (d, ^2^*J*_F,C_ = 24.8 Hz), 117.7 (d, ^2^*J*_F,C_ = 16.9 Hz), 124.7, 134.9, 150.9, 151.7, 152.5 (d, ^4^*J*_F,C_ = 2.2 Hz), 154.0 (d, ^1^*J*_F,C_ = 242.1 Hz), 155.0; LC–MS (ESI) (solvent A: 1% CH_3_CN and 0.1% HCO_2_H in double-distilled H_2_O; solvent B: CH_3_CN. Elution gradient: 0 → 1 min, 25% B; 1 → 6 min, 25 → 98% B; 6 → 6.5 min, 98% B; 6.5 → 7 min, 98 → 25% B; 7 → 10 min, 25% B, DAD 220–400 nm), *t*_R_ = 6.88 min, 95% purity, *m*/*z* calcd for C_18_H_23_FN_2_O_3_S [M + H]^+^, 367.1; found, 366.8.

##### *tert*-Butyl (*S*)-(5-(4-Methylthiazol-5-yl)-2,3-dihydro-1*H*-inden-1-yl)carbamate (**60e**)

Following general procedure B, compound **60e** was obtained from **59e** (type **59**, R = H, *n* = 1; 0.47 g, 1.0 mmol). The crude product was purified by flash column chromatography (gradient from 20 to 50% EtOAc in petroleum ether) to obtain a pale yellow solid. Yield: 0.32 g (68%); mp 114 °C; *R*_f_ = 0.60 (PE/EtOAc 1:1); ^1^H NMR (500 MHz, DMSO-*d*_6_): δ 1.44 (s, 9H), 1.79–1.90 (m, 1H), 2.33–2.41 (m, 1H), 2.44 (s, 3H), 2.75–2.85 (m, 1H), 2.90–2.98 (m, 1H), 4.98–5.05 (m, 1H), 7.21–7.35 (m, 4H), 8.97 (s, 1H); ^13^C NMR (126 MHz, DMSO-*d*_6_): δ 15.9, 28.2, 29.5, 32.6, 54.9, 77.8, 124.1, 125.0, 127.3, 130.4, 131.5, 143.6, 144.6, 147.6, 151.3, 155.6; LC–MS (ESI) (90% H_2_O to 100% MeCN in 10 min, then 100% MeCN to 15 min, DAD 200–600 nm), *t*_R_ = 7.55 min, 100% purity, *m*/*z* calcd for C_18_H_22_N_2_O_2_S [M + H]^+^, 331.15; found, 331.2. HRMS (ESI) *m*/*z*: calcd for C_18_H_22_N_2_O_2_S [M + H]^+^, 331.1475; found, 331.1470.

##### *tert*-Butyl (*S*)-(6-Fluoro-5-(4-methylthiazol-5-yl)-2,3-dihydro-1*H*-inden-1-yl)carbamate (**60f**)

This compound was prepared using general procedure B and **60f** (type **60**, R = F, *n* = 1; 0.210 g, 0.64 mmol). The crude product was purified by column chromatography (EtOAc/*n*-hexanes 1:1) to obtain a waxy solid. Yield: 0.115 g (52%); *R*_f_ = 0.3 (EtOAc/*n*-hexanes 1:1); ^1^H NMR (400 MHz, CDCl_3_): δ 1.50 (s, 9H), 1.81–1.89 (m, 1H), 2.42 (d, *J* = 1.4 Hz, 3H), 2.60–2.68 (m, 1H), 2.79–2.89 (m, 1H), 2.91–2.99 (m, 1H), 4.77 (d, *J* = 8.9 Hz, 1H), 5.22 (q, *J* = 8.2 Hz, 1H), 7.14 (dd, *J* = 9.5, 1.0 Hz, 1H), 7.19 (d, *J* = 6.7 Hz, 1H), 8.76 (s, 1H); ^13^C NMR (101 MHz, CDCl_3_): δ 15.9, 28.4, 29.4, 34.7, 55.8, 79.9, 111.8 (d, ^2^*J*_F,C_ = 23.3 Hz), 118.9 (d, ^2^*J*_F,C_ = 16.9 Hz), 124.9, 127.8 (d, ^3^*J*_F,C_ = 5.7 Hz), 138.6 (d, ^4^*J*_F,C_ = 2.6 Hz), 146.6 (d, ^3^*J*_F,C_ = 7.4 Hz), 150.8, 151.6, 155.6, 159.1 (d, ^1^*J*_F,C_ = 247.5 Hz); LC–MS (ESI) (solvent A: 1% CH_3_CN and 0.1% HCO_2_H in double-distilled H_2_O; solvent B: CH_3_CN. Elution gradient: 0 → 1 min, 25% B; 1 → 6 min, 25 → 98% B; 6 → 6.5 min, 98% B; 6.5 → 7 min, 98 → 25% B; 7 → 10 min, 25% B, DAD 220–400 nm), *t*_R_ = 7.04 min, 97% purity, *m*/*z* calcd for C_18_H_21_FN_2_O_2_S [M + H]^+^, 349.14; found, 349.1.

##### *tert*-Butyl (*S*)-(7-Fluoro-6-(4-methylthiazol-5-yl)-1,2,3,4-tetrahydronaphthalen-1-yl)carbamate (**60g**)

This compound was prepared using general procedure B and **59g** (type **59**, R = F, *n* = 2; 0.254 g, 0.74 mmol). The crude product was purified by column chromatography (EtOAc/*n*-hexanes 1:2) to obtain a white solid. Yield: 0.11 g (44%); mp 108–109 °C; *R*_f_ = 0.25 (EtOAc/*n*-hexanes 1:2); ^1^H NMR (400 MHz, CDCl_3_): δ 1.50 (s, 9H), 1.71–1.79 (m, 1H), 1.86 (dd, *J* = 10.9, 5.9 Hz), 2.11 (d, *J* = 8.4 Hz, 1H), 2.43 (s, 3H), 2.74–2.78 (m, 2H), 4.79 (d, *J* = 9.2 Hz, 1H), 4.82–4.97 (m, 1H), 7.07 (d, *J* = 7.4 Hz, 1H), 7.17 (d, *J* = 10.7 Hz, 1H), 8.76 (s, 1H); ^13^C NMR (101 MHz, CDCl_3_): δ 16.0, 16.0, 20.3, 28.4, 30.3, 48.6, 79.8, 115.3 (d, ^2^*J*_F,C_ = 22.3 Hz), 118.4 (d, ^2^*J*_F,C_ = 16.0 Hz), 124.5, 132.3 (d, ^4^*J*_F,C_ = 2.6 Hz), 133.1 (d, ^3^*J*_F,C_ = 3.5 Hz), 140.2 (d, ^3^*J*_F,C_ = 6.6 Hz), 150.8, 151.6, 155.5, 158.1 (d, ^1^*J*_F,C_ = 247.5 Hz); LC–MS (ESI) (solvent A: 1% CH_3_CN and 0.1% HCO_2_H in double-distilled H_2_O; solvent B: CH_3_CN. Elution gradient: 0 → 1 min, 25% B; 1 → 6 min, 25 → 98% B; 6 → 6.5 min, 98% B; 6.5 → 7 min, 98 → 25% B; 7 → 10 min, 25% B, DAD 220–400 nm), *t*_R_ = 7.33 min, 95% purity, *m*/*z* calcd for C_19_H_23_FN_2_O_2_S [M + H]^+^, 363.1, found, 362.6.

##### Benzyl (2*S*,4*R*)-1-((*S*)-2-(1-Fluorocyclopropane-1-carboxamido)-3,3-dimethylbutanoyl)-4-hydroxypyrrolidine-2-carboxylate (**61**)

Following general procedure C, compound **61** was obtained using Boc-protected amine **44** (102 mg, 0.3 mmol) and 1-fluoro-1-cyclopropanecarboxylic acid (101 mg, 0.3 mmol). The residue was purified by flash column chromatography using a gradient from 0 to 10% MeOH in CH_2_Cl_2_ to afford **61** as a white solid. Yield: 90 mg (51%); mp 118–120 °C; *R*_f_ = 0.42 (CH_2_Cl_2_/MeOH 9:1); ^1^H NMR (600 MHz, DMSO-*d*_6_): δ 0.92 (s, 9H), 1.17–1.25 (m, 2H), 1.30–1.41 (m, 2H), 1.90–1.97 (m, 1H), 2.13–2.19 (m, 1H), 3.59–3.64 (m, 1H), 3.67 (dd, *J* = 10.8, 3.9 Hz, 1H), 4.32–4.36 (m, 1H), 4.46 (dd, *J* = 9.2, 7.8 Hz, 1H), 4.59 (d, *J* = 9.2 Hz, 1H), 5.13 (dd, *J* = 12.3, 12.1, 2H), 5.23 (d, *J* = 3.9 Hz, 1H), 7.26 (dd, *J* = 9.3, 2.9 Hz, 1H), 7.31–7.39 (m, 5H); ^13^C NMR (151 MHz, DMSO-*d*_6_): δ 12.7 (d, ^2^*J*_F,C_ = 10.3 Hz), 12.9 (d, ^2^*J*_F,C_ = 10.2 Hz), 26.0, 35.8, 37.2, 56.3, 56.4, 57.9, 66.0, 68.8, 78.0 (d, ^1^*J*_F,C_ = 232.7 Hz), 127.9, 128.0, 128.4, 135.8, 168.1 (d, ^2^*J*_F,C_ = 20.9 Hz), 169.3, 171.5; LC–MS (ESI) (90% H_2_O to 100% MeCN in 10 min, then 100% MeCN to 15 min, DAD 200–600 nm), *t*_R_ = 6.39 min, 99% purity, *m*/*z* calcd for C_22_H_29_FN_2_O_5_ [M + H]^+^, 421.21; found, 421.3.

##### (2*S*,4*R*)-1-((*S*)-2-(1-Fluorocyclopropane-1-carboxamido)-3,3-dimethylbutanoyl)-4-hydroxypyrrolidine-2-carboxylic Acid (**62**)

Compound **62** (2.10 g, 5 mmol) was dissolved in dry EtOH (50 mL) and treated with 10% m/m Pd/C under H_2_ (1 atm, balloon) for 18 h. The reaction mixture was filtered through Celite and was concentrated to yield a white solid. This compound was used in the next step without further purification and characterization.

##### 3-Bromo-2-methoxybenzaldehyde (**64**)

3-Bromo-2-hydroxybenzaldehyde (**63**, 5.03 g, 25 mmol) and Li_2_CO_3_ (4.6 g, 62.5 mmol) were suspended in dry DMF (40 mL). The mixture was stirred at 45 °C for 1 h, after which MeI (2.3 mL, 37.5 mmol) was added. It was further stirred at this temperature for 16 h. The slightly yellow suspension was filtered through a pad of Celite, and it was washed with EtOAc (400 mL). The organic layer was washed with H_2_O (400 mL) and brine (200 mL), dried over Na_2_SO_4_, filtered, and evaporated in vacuo. The crude product was purified by column chromatography (5% EtOAc in cyclohexane) to give the title compound as a yellowish solid. Yield: 4.56 g (84%); mp 30–32 °C; *R*_f_ = 0.48 (petroleum ether/EtOAc 19:1); ^1^H NMR (500 MHz, DMSO-*d*_6_): δ 3.93 (s, 3H), 7.27 (t, *J* = 7.8 Hz, 1H), 7.76 (dd, *J* = 7.9, 1.7 Hz, 1H), 7.97 (dd, *J* = 7.9, 1.6 Hz, 1H), 10.24 (s, 1H); ^13^C NMR (126 MHz, DMSO-*d*_6_): δ 63.5, 117.9, 126.4, 128.3, 130.9, 139.5, 159.3, 189.5; LC–MS (ESI) (90% H_2_O to 100% MeCN in 10 min, then 100% MeCN to 15 min, DAD 200–600 nm), *t*_R_ = 5.68 min, 99% purity, *m*/*z* calcd for C_8_H_7_^79^BrO_2_ [M + H]^+^, 214.97; found, 215.1.

##### 3-Bromo-2-methoxyphenol (**65**)

Trifluoroacetic anhydride (16 mL, 117 mmol) was added to a mixture of 35% aqueous H_2_O_2_ (1.93 mL, 22.5 mmol) in CH_2_Cl_2_ (20 mL) at 0 °C, and it was stirred at this temperature for 1 h. In a separate flask, aldehyde **64** (3.22 g, 15 mmol) and KH_2_PO_4_ (40.83 g, 300 mmol) were suspended in CH_2_Cl_2_ (150 mL) and cooled to 0 °C. Subsequently, the first solution containing the in situ generated peracid was added dropwise, and the combined mixture was stirred at 0 °C for 30 min. The reaction was quenched by the addition of 40% NaHSO_3_ solution (100 mL) and H_2_O (50 mL), the organic layer was separated, and the aqueous solution was extracted again with CH_2_Cl_2_ (150 mL). The combined organic layers were dried over Na_2_SO_4_, filtered, and concentrated in vacuo. The remaining residue was dissolved in MeOH (80 mL), and concd. HCl (8 drops) was added. The mixture was stirred at rt for 45 min, after which the solvent was evaporated, and it was further dried under high vacuum. The crude product was purified by column chromatography (gradient from 10 to 15% EtOAc in cyclohexane) to give the title compound as a colorless oil. Yield: 2.71 g (89%); *R*_f_ = 0.34 (petroleum ether/EtOAc 9:1); ^1^H NMR (500 MHz, DMSO-*d*_6_): δ 3.72 (s, 3H), 6.79–6.89 (m, 2H), 6.94–7.03 (m, 1H), 9.74 (s, 1H); ^13^C NMR (126 MHz, DMSO-*d*_6_): δ 59.9, 116.6, 116.9, 122.9, 125.5, 144.9, 151.7; LC–MS (ESI) (90% H_2_O to 100% MeCN in 10 min, then 100% MeCN to 15 min, DAD 200–600 nm), *t*_R_ = 4.90 min, 99% purity, *m*/*z* calcd for C_7_H_7_^79^BrO_2_ [M + H]^+^, 202.97; found, 202.9.

##### 4-Bromo-2-hydroxy-3-methoxybenzaldehyde (**66**)

Compound **65** (2.03 g, 10 mmol) was dissolved in dry THF (20 mL), and Et_3_N (2.78 mL, 20 mmol) and MgCl_2_ (1.90 g, 20 mmol) were added. This mixture was stirred for 10 min at rt, after which paraformaldehyde (0.90 g, 30 mmol) was introduced, and it was heated to 60 °C for 16 h. After cooling, 10% KHSO_4_ solution (50 mL) was added, and it was extracted with EtOAc (2 × 50 mL). The combined organic layers were washed with saturated NH_4_Cl solution and brine (each 50 mL), dried over Na_2_SO_4_, filtered, and concentrated in vacuo. The crude product was purified by flash chromatography on spherical silica gel (80 g, 30 μm, gradient from 25 to 100% CH_2_Cl_2_ in petroleum ether) to give a colorless semi-solid. Yield: 0.62 g (27%); *R*_f_ = 0.28 (petroleum ether/CH_2_Cl_2_ 3:1); ^1^H NMR (600 MHz, DMSO-*d*_6_): δ 3.78 (s, 3H), 7.21 (d, *J* = 8.5 Hz, 1H), 7.37 (d, *J* = 8.4 Hz, 1H), 10.19 (s, 1H), 10.87 (br s, 1H); ^13^C NMR (151 MHz, DMSO-*d*_6_): δ 60.6, 123.4, 123.5, 124.5, 125.8, 146.1, 154.6, 191.9; LC–MS (ESI) (90% H_2_O to 100% MeCN in 10 min, then 100% MeCN to 15 min, DAD 200–600 nm), *t*_R_ = 5.03 min, 98% purity, *m*/*z* calcd for C_8_H_7_^79^BrO_3_ [M + H]^+^, 230.97; found, 231.0.

##### 4-Bromo-2,5-dichloro-*N*-methoxy-*N*-methylbenzamide (**67**)

4-Bromo-2,5-dichlorobenzoic acid (**47**, 2.00 g, 7.4 mmol), *N*,*O*-dimethylhydroxylamine hydrochloride (1.44 g, 14.8 mmol), EDC × HCl (1.56 g, 8.14 mmol), and Et_3_N (1.13 mL, 8.14 mmol) were mixed in CH_2_Cl_2_ (75 mL) and stirred at room temperature for 16 h. Subsequently, the crude material was subjected to column chromatography (gradient from 10 to 20% EtOAc in cyclohexane) to give the title compound as a colorless solid. Yield: 2.18 g (94%); mp 104–106 °C; *R*_f_ = 0.30 (petroleum ether/EtOAc 8:1); ^1^H NMR (500 MHz, DMSO-*d*_6_): δ 3.27 (s, 3H), 3.47 (s, 3H), 7.85 (s, 1H), 8.04 (s, 1H); ^13^C NMR (126 MHz, DMSO-*d*_6_): δ 32.0, 61.4, 123.0, 128.9, 129.2, 132.4, 133.9, 136.4, 164.9; LC–MS (ESI) (90% H_2_O to 100% MeOH in 10 min, then 100% MeOH to 20 min, DAD 220–400 nm), *t*_R_ = 10.79 min, 93% purity, *m*/*z* calcd for C_9_H_9_^81^BrCl_2_NO_2_ [M + H]^+^, 313.92; found, 314.0; HRMS (ESI) *m*/*z*: calcd for C_9_H_8_^79^BrCl_2_NO_2_ [M + H]^+^, 311.9188; found, 311.9182.

##### 4-Bromo-2,5-dichlorobenzaldehyde (**68**)

A Schlenk flask was charged with compound **67** (2.13 g, 6.8 mmol), evacuated, and refilled with argon gas. The material was dissolved in dry THF (30 mL) and cooled to 0 °C. LiAlH_4_ solution (1 M in THF, 3.4 mL) was added dropwise, and the mixture was stirred at this temperature for 1 h. Subsequently, it was cooled to −15 °C and slowly quenched by the addition of 10% KHSO_4_ solution (100 mL). The aqueous solution was extracted with Et_2_O (2 × 100 mL) dried over Na_2_SO_4_, filtered, and concentrated in vacuo. The crude product was filtered through a small plug of silica gel, and the product was eluted with CH_2_Cl_2_. Evaporation of the solid yielded a colorless solid. Yield: 1.71 g (99%); mp 96–100 °C; *R*_f_ = 0.78 (petroleum ether/EtOAc 19:1); ^1^H NMR (500 MHz, DMSO-*d*_6_): δ 7.96 (s, 1H), 8.17 (s, 1H), 10.19 (s, 1H); ^13^C NMR (126 MHz, DMSO-*d*_6_): δ 128.8, 130.6, 132.9, 133.4, 134.8, 135.6, 188.4; LC–MS (ESI) (90% H_2_O to 100% MeOH in 10 min, then 100% MeOH to 20 min, DAD 220–400 nm), *t*_R_ = 11.31 min, 87% purity, *m*/*z* calcd for C_7_H_4_^81^BrCl_2_O [M + H]^+^, 252.88; mass not found; HRMS (ESI) *m*/*z*: calcd for C_7_H_3_^81^BrCl_2_O [M + H]^+^, 252.8817; found, 252.8828.

##### 4-Bromo-3-fluoro-2-hydroxybenzaldehyde (**69**)

3-Bromo-2-fluorophenol (**51**, 5.0 g, 26 mmol) was dissolved in dry THF (50 mL) and Et_3_N (7.2 mL, 52 mmol) as well as MgCl_2_ (4.95 g, 52 mmol) were added. The mixture was stirred at room temperature for 10 min, after which paraformaldehyde (2.34 g, 78 mmol) was introduced. The combined mixture was stirred under an argon atmosphere at 65 °C for 16 h. After cooling, it was diluted with 10% KHSO_4_ solution (100 mL) and extracted with EtOAc (3 × 50 mL). The combined organic layers were washed with NH_4_Cl solution and brine (each 50 mL), dried over Na_2_SO_4_, filtered, and concentrated in vacuo. The crude product was purified by column chromatography (petroleum ether/EtOAc 19:1) to give the title compound as a colorless solid. Yield: 4.79 g (87%); mp 120–122 °C; *R*_f_ = 0.38 (petroleum ether/EtOAc 19:1); ^1^H NMR (600 MHz, DMSO-*d*_6_): δ 7.25 (dd, *J* = 8.5, 5.8 Hz, 1H), 7.41 (dd, *J* = 8.5, 1.7 Hz, 1H), 10.23 (s, 1H), 11.33 (br s, 1H); ^13^C NMR (151 MHz, DMSO-*d*_6_): δ 116.0 (d, ^2^*J*_F,C_ = 18.7 Hz), 123.1, 123.9–125.8 (m), 147.3–150.8 (m), 190.1; LC–MS (ESI) (90% H_2_O to 100% MeOH in 10 min, then 100% MeOH to 20 min, DAD 220–420 nm), *t*_R_ = 8.63 min, 98% purity, *m*/*z* calcd for C_7_H_4_^79^BrFO_2_ [M + H]^+^, 218.95; found, 218.2; HRMS (ESI) *m*/*z*: calcd for C_7_H_4_^79^BrFO_2_ [M – H]^−^, 216.9306; found, 216.9301.

### Molecular Docking and Binding Site Analysis

Molecular docking of the synthesized compounds in the co-crystal structure of ligand VH298 and VCB (PDB: 5LLI)^13^ was performed using the program GOLD.^55^ Protein preparation was performed in Hermes, where hydrogen atoms were added with default settings. The docking calculations included structural water molecules, Wat406, Wat436, Wat440, Wat450, and Wat456. Two setups were used, (1) considering Wat406 and Wat450 and (2) considering all the five structural waters. In particular, Wat406 and Wat450 were recognized to play an important role in the correct molecular recognition of VH298 and related ligands.^13,19^ Prior to the docking calculations, conformations of the synthesized derivatives were generated and geometrically optimized using the MMFF94 force field. The binding site at VCB was defined within a 10 Å radius of the coordinates of the bound VH298. Each molecule was docked 10 times in the binding site using a genetic algorithm as a search engine with the following settings, population size 100, selection pressure 1.1, number of operations 100,000, number of islands 5, niche size 2, crossover frequency 95, mutation frequency 95, and migration frequency 10. The “spin” option was enabled to allow GOLD to automatically optimize the orientation of the hydrogen atoms of the included water molecules during docking. ChemPLP evaluated the obtained docking solutions. This scorning function, integrated into GOLD, uses the ChemScore hydrogen bonding term and several linear potentials to model the van der Waals and repulsion terms.^56^ Both settings of the docking tool GOLD were validated^57^ by successfully redocking the VH298 ligand in the VCB complex (Figure S2). LigandScout was used to visualize and evaluate the obtained docking solutions and to generate structure-based pharmacophores.^58^ LigandScout was also employed for the Apo Site Grid analysis with the default settings used. Here, the binding site of VCB was scanned with molecular probes such as a hydrophobic probe, and the contours of the corresponding MIFs were subsequently derived.^31^ The apo site feature also allows the determination of buriedness; a parameter which evaluates the accessibility of regions within the binding site.

### Single-Crystal X-ray Diffraction

The X-ray crystallographic data collection for **59b** was performed on a Bruker D8-Venture diffractometer (Photon I detector) at 169(2) K. The diffractometer was equipped with a low-temperature device (Oxford Cryostream 800, Oxford Cryosystems) and used mirror optic monochromated Cu Kα radiation (λ = 1.54178 Å). Intensities were measured by fine-slicing ϕ- and ω-scans and corrected for background, polarization, and Lorentz effects. Semi-empirical absorption corrections were applied for all data sets by using Bruker’s SADABS program. The structure was solved by intrinsic phasing methods and refined anisotropically by the least-squares procedure implemented in the ShelX program system.^59,60^ The hydrogen atoms were included isotropically using the riding model on the bound carbon atoms. The Flack parameter (0.09(5)) and the Bayesian statistics on Bijvoet differences (P2(true) = 1.000; P3(true) = 1.000; P3(rac-twin) = 0.2 × 10^–182^; and P3(false) = 0.000)^61^ unambiguously confirm the absolute configuration of **59b**.

CCDC 2236731 contains the supplementary crystallographic data for this paper. The data can be obtained free of charge from The Cambridge Crystallographic Data Centre via www.ccdc.cam.ac.uk/getstructures.

### Determination of Physicochemical Properties

#### Log *D*_7.4_ Measurement

The determination of the log *D*_7.4_ values was performed by a chromatographic method as described previously.^41,62^ The system was calibrated by plotting the retention times of six different drugs (atenolol, metoprolol, labetalol, diltiazem, triphenylene, and permethrin) versus their literature-known log *D*_7.4_ values (*R*^2^ = 0.99). Subsequently, the mean retention times (*n* = 2) of the analytes were taken to calculate their log *D*_7.4_ values.

#### PPB Studies

PPB was estimated by correlating the logarithmic retention times of the analytes on a CHIRALPAK HSA 50 × 3 mm, 5 μm column with the literature-known % PPB values (converted into log *K* values) of the following drugs: warfarin, ketoprofen, budesonide, nizatidine, indomethacin, acetylsalicylic acid, carbamazepine, piroxicam, nicardipine, and cimetidine.^63^ Samples were dissolved in MeCN/DMSO 9:1 to achieve a final concentration of 0.5 mg/mL. The mobile phase A was 50 mM ammonium acetate adjusted to pH 7.4 with 10% NaOH, while mobile phase B was *i*PrOH. The flow rate was set to 1.0 mL/min, the UV detector was set to 254 nm, and the column temperature was kept at 30 °C. After injecting 3 μL of the sample, a linear gradient from 100% A to 30% *i*PrOH in 5.4 min was applied. From 5.4 to 18 min, 30% *i*PrOH was kept, followed by switching back to 100% A in 1.0 min and a re-equilibration time of 6 min. With the aid of the calibration line (*R*^2^ = 0.94), the log *K* values of new substances were calculated and converted to their % PPB values.

### Biophysical Methods

#### FP Binding Assay

FP competitive binding assays were performed using a PHERAstar FS (BMG LABTECH) with fluorescence excitation and emission wavelengths of λ = 485 and λ = 520 nm, respectively. Assays were run in triplicates of three independent experiments using 384-well plates (Corning 3820), with each well solution containing 15 nM VCB protein, 10 nM 5,6-carboxyfluorescein (FAM)-labeled HIF-1α peptide (FAM-Asp-Glu-Ala-Leu-Ala-Hyp-Tyr-Ile-Pro-Met-Asp-Asp-Asp-Phe-Gln-Leu-Arg-Ser-Phe-NH_2_, “JC9”), and decreasing concentrations of VHL ligands (14-point and 2-fold serial dilution starting from 100 μM VHL ligand). All components were dissolved from stock solutions using 100 mM Bis–Tris, 100 mM NaCl, 1 mM DTT, pH 7.0, to yield a final assay volume of 15 μL. DMSO was added as appropriate to ensure a final concentration of 2% (v/v). Control wells containing VCB and JC9 with no compound (zero displacement), or JC9, in the absence of protein (maximum displacement) were also included to allow for normalization. Percentage displacement values were obtained by normalization of controls and were plotted against log[compound]. The IC_50_ values were determined for each titration using non-linear regression analysis with Prism GraphPad (Table S1). Dissociation constants *K*_d_ for the compound–VCB interaction were back-calculated from the measured IC_50_ values by using a displacement binding model as described.^16,64^

#### Surface Plasmon Resonance

To perform SPR measurements, 10 mM stock solution of VHL inhibitors was diluted 100-fold in DMSO to achieve a 100 μM final stock concentration. The ligand stock solution was diluted in SPR buffer (20 mM HEPES, 150 mM NaCl, 1 mM TCEP, 0.005% Tween 20, pH 7.0) to obtain the final concentration of 2% (v/v) DMSO. Final concentrations from 1 μM to 1.4 nM of the VHL inhibitors were prepared on a 96-well plate (7-point, 3-fold serial dilution starting from 1 μM VHL ligand). The experiments were performed at 20 °C on a Biacore T100 (GE Healthcare, Biacore, Uppsala, Sweden) equipped with a streptavidin-functionalized sensor chip (Series S Sensor Chip SA, Cytiva). The system was flushed with running buffer (20 mM HEPES, 150 mM NaCl, 1 mM TCEP, 0.005% Tween 20, and 2% DMSO, pH 7.0). Biotinylated VCB protein (50 nM) was immobilized onto the sensor chip at 10 μL/min for using the automated wizard in the T200 control software to reach the required immobilization levels. The solutions were injected individually using 60 and 160 s association and dissociation times, respectively. Reference flow-cell response was subtracted from the sample response with immobilized VCB protein to correct for systematic noise and baseline drift. Data were solvent-corrected by an eight-point solvent correction, and the response from the blank injections was used to double-reference the binding data. For the determination of binding constants, processed kinetic data were fitted to a 1:1 interaction model using the Biacore Insight Evaluation Software (version 3.0.12.15655).

### Biological Methods

#### Protein Expression and Purification

A plasmid containing pVHL_54–213_ with an N-terminal His6 tag and a duet plasmid containing EloB_1–104_ and EloC_17–112_ were used to generate a complex of pVHL/EloB:EloC as described previously.^14^ All proteins were co-expressed from their respective plasmids in *Escherichia coli* BL21 (DE3) at 24 °C for 16 h. *E. coli* cells were lysed using a pressure cell homogenizer (Stansted Fluid Power) and lysate-clarified by centrifugation. His6-tagged VBC was purified on a HisTrapFF affinity column (GE Healthcare) by elution with an imidazole gradient. The His6 tag was removed using TEV protease and the untagged complex was dialyzed into low imidazole concentration buffer. VBC was then flowed through the HisTrapFF column a second time, allowing impurities to bind as the complex eluted without binding. VBC was then additionally purified by anion exchange and size-exclusion chromatography using MonoQ and Superdex-75 columns (GE Healthcare), respectively. All chromatography purification steps were performed using Äkta FPLC purification systems (GE Healthcare) at 4 °C or room temperature. The final purified complex was stored in 20 mM HEPES, pH 7, 150 mM sodium chloride, and 1 mM DTT.

#### Cell Culture

HeLa, HEK 293, and U2OS cell lines, purchased from ATCC, were cultured in Dulbecco’s modified Eagle’s medium (DMEM, Gibco) supplemented with 10% fetal bovine serum (FBS, Gibco), l-glutamine (2 mM, Gibco), and 100 μg/mL penicillin/streptomycin (Gibco). HeLa and U2OS cell lines stably expressing an HRE-luciferase reporter, kindly gifted from Sonia Rocha’s laboratory, were cultured in DMEM (Gibco) supplemented with 10% FBS (Gibco), l-glutamine (2 mM, Gibco), 100 μg/mL penicillin/streptomycin (Gibco), and 0.5 μg/mL puromycin (InvivoGen). All cell lines were maintained in a humidified incubator at 37 °C and 5% CO_2_ for no more than 30 passages. Cells were routinely tested for mycoplasma contamination.

#### Cell Treatments for Immunoblotting

HeLa and HEK 293 cells were plated in six-well plates at varying densities (1–5 × 10^5^ cells/mL) 24–42 h before treatment depending on the experimental setup. Cells were treated in fresh medium with the indicated compounds under indicated conditions with a final DMSO concentration of 1% (v/v). After compound treatment, the medium was removed, and the cells were washed with ice-cold phosphate-buffered saline (PBS) and lysed on ice with 100 μL of RIPA lysis and extraction buffer (Thermo Fisher Scientific) supplemented with a complete EDTA-free protease inhibitor cocktail (Roche). Cells were incubated for 15 min on ice and then detached from the surface by scraping. After removal of the insoluble fraction by centrifugation at 15,000 *g* at 4 °C for 15 min, supernatants were stored at −80 °C. The protein concentration was determined by bicinchoninic acid (BCA) assay (Pierce).

#### Quantitative Immunoblotting

Cell lysates containing a quarter of a volume of 4× NuPAGE LDS sample buffer (NP0007) supplemented with 10% β-mercaptoethanol were heated at 95 °C for 5 min. Samples (30 μg) were loaded onto precast 4–12% bis–tris midi 26W gels (Thermo Fisher Scientific) and resolved at 90 V for 10 min and then 130 V for 1.5 h with a NuPAGE MOPS SDS running buffer (Thermo Fisher Scientific). Proteins were electrophoretically transferred onto a 0.45 μm nitrocellulose membrane (GE Healthcare, Amersham Protran Supported 0.45 mm NC) at 90 V for 90 min on ice in a transfer buffer (48 mM tris base and 39 mM glycine supplemented with 20% ethanol). The transferred membrane was blocked with 5% (w/v) skim milk powder dissolved in tris-buffered saline with Tween (TBS-T) (50 mM tris base, 150 mM sodium chloride (NaCl), and 0.1% (v/v) Tween-20) at room temperature for 1 h. Western blot images were obtained through detection with anti-HIF-1α (BD Biosciences, #610959, clone 54, 1:1000) and anti-hydroxy-HIF-1α (Hyp564) (Cell Signaling Technology; #3434, 1:1000) antibodies. Following overnight incubation with the primary antibodies at 4 °C, the membranes were washed two times for 10 min with TBS-T and then incubated with secondary antibodies (IRDye 800CW donkey anti-rabbit secondary antibody (LI-COR #926-32213, 1:5000) or IRDye 800CW donkey anti-mouse secondary antibody (Li-COR #926-32212, 1:5000) and hFABTM rhodamine anti-tubulin antibody (Bio-Rad, 12004165, 1:10,000)) for 1 h at room temperature and protected from light. Thereafter, the membranes were washed with TBS-T three times for 10 min, and protein bands were acquired using a ChemiDoc MP imaging system (Bio-Rad). Band quantification was performed using Image Lab software and reported as a relative amount as the ratio of each protein band relative to the lane’s loading control. The values obtained were then normalized to the VH298 vehicle control.

#### HRE-Luciferase Reporter Assay

HeLa and U2OS cells stably expressing an HRE-luciferase reporter were seeded in 12-well plates at 2.4–3 × 10^5^ cells/mL 24 h prior to treatment with compounds at the indicated concentrations or with 1% (v/v) DMSO as a control in fresh medium for 32 h. After compound treatment, the medium was removed, and the cells were washed twice with 0.5 mL of ice-cold PBS, lysed on ice with passive lysis buffer (Promega, E1941), and subjected to one freeze–thaw cycle. Luciferase assays were performed according to the manufacturer’s instructions (Promega), and activity was measured using a PHERAstar FSX (BMG LABTECH) plate reader. Results were normalized for protein concentration determined by BCA (Pierce) and reported as a means and SEM from three biological replicates.

#### Quantitative Real-Time PCR

HeLa cells were seeded in six-well plates at 7.5 × 10^5^ cells/well 24 h prior to treatment with compounds at the indicated concentrations or with 1% (v/v) DMSO as a control in fresh medium for 16 h. After compound treatment, the medium was removed, and the cells were washed twice with 1 mL of ice-cold PBS, then lysed in RLT lysis buffer (Qiagen RNeasy kit, 74104) supplemented with 1% (v/v) β-mercaptoethanol, and stored at −80 °C. mRNA was extracted from cell lysates using the RNeasy Mini Kit in combination with QIAshredders (Qiagen, 79654) for cell lysate homogenization and on-column treatment with RNase-Free DNase (Qiagen, 79256) and reverse-transcribed using the iScript cDNA Synthesis kit (Bio-Rad, 1708891). Real-time PCR was performed using iTaq Universal SYBR Green Supermix (Bio-Rad, 1725121) in a C1000 Touch Thermal Cycler (Bio-Rad). mRNA levels were calculated based on averaged *C*_*t*_ values from three technical replicates, normalized to mRNA levels of β-actin, and reported as mean from two biological replicates.

#### NanoBRET Target Engagement Assay

For VHL target engagement experiments in live and permeabilized cells, HEK 293 cells were transiently transfected with the VHL–NanoLuc fusion vector (Promega, N275A) following the manufacturer’s protocol, transferred to a tissue-culture-treated flask, and incubated in a humidified, 37 °C/5% CO_2_ incubator for 20–24 h. Following transfection, the cells were washed with PBS, harvested by trypsinization, and resuspended in Opti-MEM. The cells were seeded into white non-binding surface 96-well plates (Corning, 3600) at a density of 2 × 10^4^ cells per well. Cells in both permeabilized and live mode were equilibrated for 30 min with energy-transfer probes and the indicated test compound before NanoBRET measurements. For permeabilized mode measurements, the cells were treated with 50 μg/mL digitonin (Sigma-Aldrich, D141), test compounds at decreasing concentrations (eight concentrations with a 3-fold serial dilution starting from 31.6 μM) and 500 nM NanoBRET VHL tracer (Promega, N292A). NanoBRET NanoGlo Substrate (Promega, N157C) was added according to the manufacturer’s recommended protocol directly before measuring filtered luminescence on a PHERAstar FSX (BMG LABTECH) plate reader equipped with a 450 nm bandpass filter (donor) and a 600 nm long-pass filter (acceptor) using 1.0 s integration time. For live mode measurements, the cells were treated with test compounds at decreasing concentrations (eight concentrations with a 3-fold serial dilution starting from 31.6 μM) and 1 μM NanoBRET VHL tracer. NanoBRET NanoGlo Substrate and an Extracellular NanoLuc Inhibitor (Promega, N2160) were added directly before the detection step. The NanoBRET ratio of each well was expressed in milliBRET according to the equation: mBRET = [(signal at 610 nM/signal at 450 nM) × 1000]. The fractional occupancy was calculated by normalizing the NanoBRET ratio against the one of DMSO. The availability index (AI) was calculated by normalizing the relative binding affinity (RBA), RBA = IC_50_ (live)/IC_50_ (perm), against the RBA of VH298. AI values > 1 indicate reduced intracellular availability of compounds compared to VH298.

#### Illustrations

Figure 1 was prepared using BioRender software (http://www.biorender.com).

### X-ray Crystallography

For VCB crystals, 2 μL of VCB (∼5 mg/mL) was mixed with 2 μL of liquor solution and grown at room temperature using a hanging-drop vapor diffusion method. The liquor solutions were composed of 0.1 M sodium cacodylate, pH 6.0–6.3, 15–20% polyethylene glycol 3350, 0.2 M magnesium acetate, and 10 mM DTT. Crystals were soaked overnight in 1.25 mM solutions of the ligand in 1–10% DMSO, 4–40% isopropanol, and 50–95% liquor solution. Crystals did not require further cryoprotection and were flash-frozen in liquid nitrogen. All X-ray data were collected at 100 K at the Diamond (beamline I04) synchrotron facilities. Outputs from the autoPROC pipeline (indexing, integration, scaling, and merging) were taken forward for molecular replacement. Molecular replacement, refinement, and small-molecule-restraint generation were carried out using the PHENIX software package.

### Dihedral Angle Calculations

Torsional energy profiles of the phenylene core of ligands **30**, **33**, and **37** were calculated with MacroModel (Schrödinger package, version 13.1, OPLS4) using an aqueous solvation model.

## Supporting Information Available

VCB binding site with the VH298 ligand and positions of amino acid residues in the RHS portion; validation docking of VH298 considering two or five structural water molecules; overlay of predicted binding modes of compounds **24**, **32**, and **33** in the VCB binding site with two or five structural water molecules; predicted binding modes of compounds **24**, **32**, and **33** in the VCB binding site with two or five structural water molecules; two- and one-dimensional dihedral angle coordinate scans of compounds **30**, **33**, and **37**; immunoblots of HIF-1α-OH stabilization in HeLa and HEK 293 cells treated with selected VHL inhibitors; dose-dependent immunoblots of HIF-1α and HIF-1α-OH stabilization in HEK 293 cells treated with VHL inhibitor **30**; HRE-luciferase reporter assay with **30** and **33** in U2OS cells; molecular plot of the X-ray crystal structure of **59b**; mean IC_50_ values obtained from the FP assay; values *k*_on_ and *k*_off_ for selected VHL ligands determined by SPR; synthesis of building blocks **41α–ε**; unsuccessful transformations of 4-bromobenzylamine derivatives; ^1^H and ^13^C NMR assignments; ^1^H and ^13^C NMR spectra; further NMR spectra of compound **14** and **30**; LC–MS traces of final compounds; and crystallographic data for co-crystal structures of VCB in complex with **30**, **33**, and **37** (PDF)
X-ray crystallographic data (CIF)
Molecular formula strings (CSV)
PDB docking coordinates (ZIP)

## Supplementary Material

jm3c00434_si_001.pdf

jm3c00434_si_002.cif

## Accession Codes

Atomic coordinates have been deposited in the Protein Data Bank and will be released upon article publication. Accession codes of VCB in complex with compounds **30** (PDB: 8CQK), **33** (PDB: 8CQL), and **37** (PDB: 8CQE).

## Abbreviations

Boc: *tert*-butyloxycarbonyl
BCA: bicinchoninic acid
BRET: bioluminescence resonance energy transfer
CA9: carbonic anhydrase 9
CRL2^VHL^: Cullin2 RING VHL E3 ubiquitin ligase complex
DTT: dithiothreitol
DIPEA: *N*,*N*-diisopropylethylamine
EloB: Elongin B
EloC: Elongin C
FAM: 5,6-carboxyfluorescein
FBS: fetal bovine serum
FP: fluorescence polarization
HATU: *O*-(7-azabenzotriazol-1-yl)-*N*,*N*,*N*′,*N*′-tetramethyluronium hexafluorophosphate
HEK: human embryonic kidney
HIF: hypoxia-inducible factor
HRE: hypoxia-responsive element
Hyp: hydroxyproline
LHS: left-hand side
MIF: molecular interaction field
PHD: prolyl hydroxylase
PPB: plasma protein binding
PBS: phosphate-buffered saline
PROTAC: proteolysis-targeting chimera
PCR: polymerase chain reaction
Rbx1: RING-box protein 1
RHS: right-hand side
SPR: surface plasmon resonance
SAR: structure–activity relationship
TFA: trifluoroacetic acid
Tle: *tert*-leucine
UPS: ubiquitin–proteasome system
VCB: VHL/Elongin C/Elongin B complex
VHL: von Hippel–Lindau
