# Supplementary material for: Expanding the Structural Diversity at the Phenylene Core of Ligands for the von Hippel–Lindau E3 Ubiquitin Ligase: Development of Highly Potent Hypoxia-Inducible Factor-1α Stabilizers
Source: J Med Chem. 2023 Sep 14;66(18):12776–811. doi: 10.1021/acs.jmedchem.3c00434 (PMC10544018; doi:10.1021/acs.jmedchem.3c00434)
Supplement: Supplementary file 1 — jm3c00434_si_001.pdf [file jm3c00434_si_001.pdf]

SUPPORTING INFORMATION

Expanding the Structural Diversity at the Phenylene Core of Ligands for the von Hippel-Lindau (VHL) E3 Ubiquitin Ligase: Development of Highly Potent Hypoxia-Inducible Factor-1α (HIF-1α) Stabilizers

Lan Phuong Vu,[§,‡] Claudia J. Diehl,[‡,#] Ryan Casement,[‡,#] Adam G. Bond,[‡,#] Christian Steinebach,[§] Nika Strašek,[Ψ] Aleša Bricelj,[Ψ] Andrej Perdih,[χ,Ψ] Gregor Schnakenburg,[⊥] Izidor Sosič,[Ψ] Alessio Ciulli*[,‡] and Michael Gütschow*[,§]

[§] Pharmaceutical Institute, Pharmaceutical & Medicinal Chemistry, University of Bonn, An der Immenburg 4, 53121 Bonn, Germany

[‡] Centre for Targeted Protein Degradation, School of Life Sciences, University of Dundee, 1 James Lindsay Place, Dundee DD1 5JJ, Scotland, U.K.

[Ψ] Faculty of Pharmacy, University of Ljubljana, Aškerčeva 7, SI-1000 Ljubljana, Slovenia

[χ] National Institute of Chemistry, Hajdrihova 19, SI-1000 Ljubljana, Slovenia

[⊥] Institute of Inorganic Chemistry, University of Bonn, Gerhard-Domagk-Straße 1, 53121 Bonn, Germany

[#] These authors contributed equally.

***** Email: a.ciulli@dundee.ac.uk (A.C.)

***** Email: guetschow@uni-bonn.de (M.G.)

Table of Contents

Supporting Figures, Schemes and Tables

Figure S1. VCB binding site with the VH298 ligand and positions of amino acid residues in the RHS portion.......... S3

Figure S2. Validation docking of VH298 considering two or five structural water molecules.......... S3

Figure S3. Overlay of predicted binding modes of compounds **24**, **32** and **33** in the VCB binding site with two or five structural water molecules.......... S4

Figure S4. Predicted binding modes of active compounds **24**, **32**, and **33** in the binding site of VHL with five structural water molecules.......... S5

Table S1. Mean IC_{50} values obtained from the FP assay.......... S6

Table S2. Values k_{on} and k_{off} for selected VHL ligands determined by SPR.......... S7

Figure S5. Two- and one-dimensional dihedral angle coordinate scans of compounds **30**, **33** and **37**.......... S8

Figure S6. Immunoblots of HIF-1α-OH stabilization in HeLa and HEK 293 cells treated with selected VHL inhibitors.......... S9

Figure S7. Dose-dependent immunoblots of HIF-1α and HIF-1α-OH stabilization in HEK 293 cells treated with VHL inhibitor **30**.......... S10

Figure S8. HRE-luciferase reporter assay with **30** and **33** in U2OS cells.......... S11

Scheme S1. Synthesis of building blocks **41α-ε**.......... S12

Scheme S2. Unsuccessful transformations of 4-bromobenzylamine derivatives.......... S12

Figure S9. Molecular plot of the X-ray crystal structure of **59b**.......... S13

^{1}H and ^{13}C NMR Assignments.......... S14

^{1}H and ^{13}C NMR Spectra.......... S58

Further NMR Spectra of Compound **14**.......... S94

Further NMR Spectra of Compound **30**.......... S96

LC-MS Traces of Final Compounds.......... S98

Crystallographic Data for Co-crystal Structures of VCB in Complex with **30**, **33**, and **37**.......... S116

Supporting Figures, Schemes and Tables

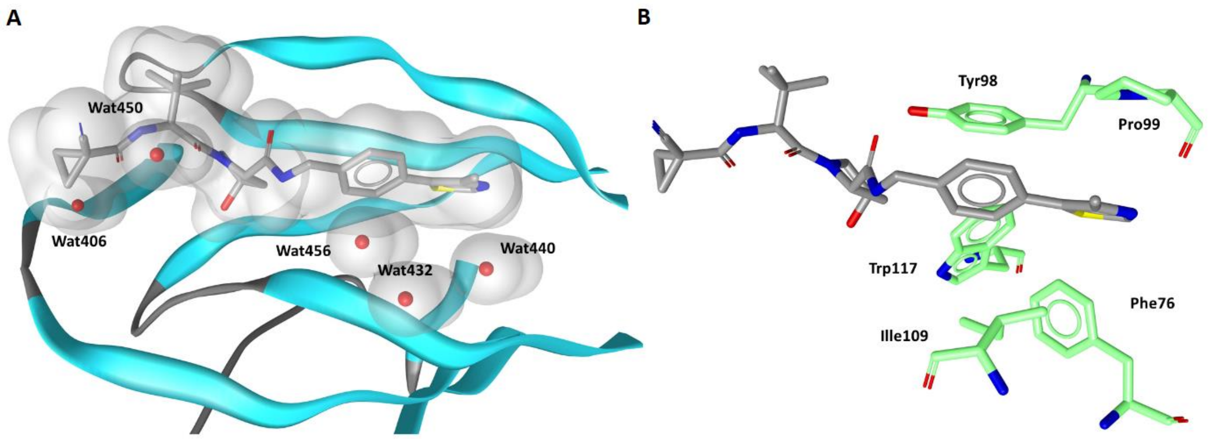

Figure S1. (A) Defined binding site of VCB and positions of the VH298 ligand as well as structural water molecules included in the docking experiments, all depicted with van der Waals surface (PDB: 5LLI). (B) Positions of the amino acid residues in the RHS portion of the binding pocket that surround the VH298 phenylene core.

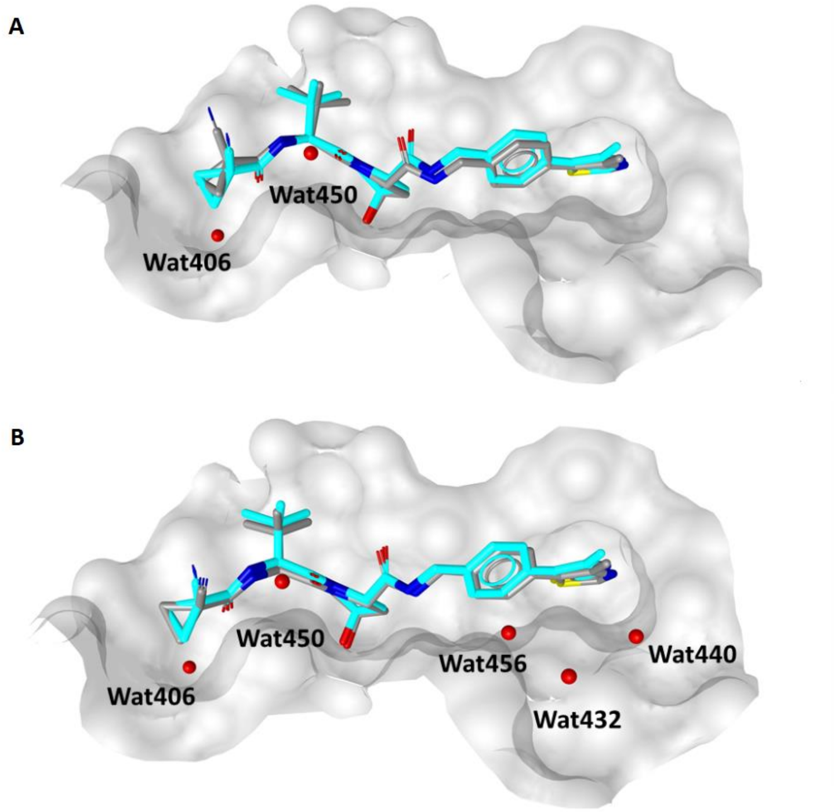

Figure S2. Validation docking of the VH298 ligand (PDB: 5LLI). The docked (gray) *versus* experimental pose (cyan) are shown for both settings considering (A) two (Wat406 and Wat450) or (B) five structural water molecules (Wat406 and Wat450, Wat456, Wat432 and Wat440) included in the docking.

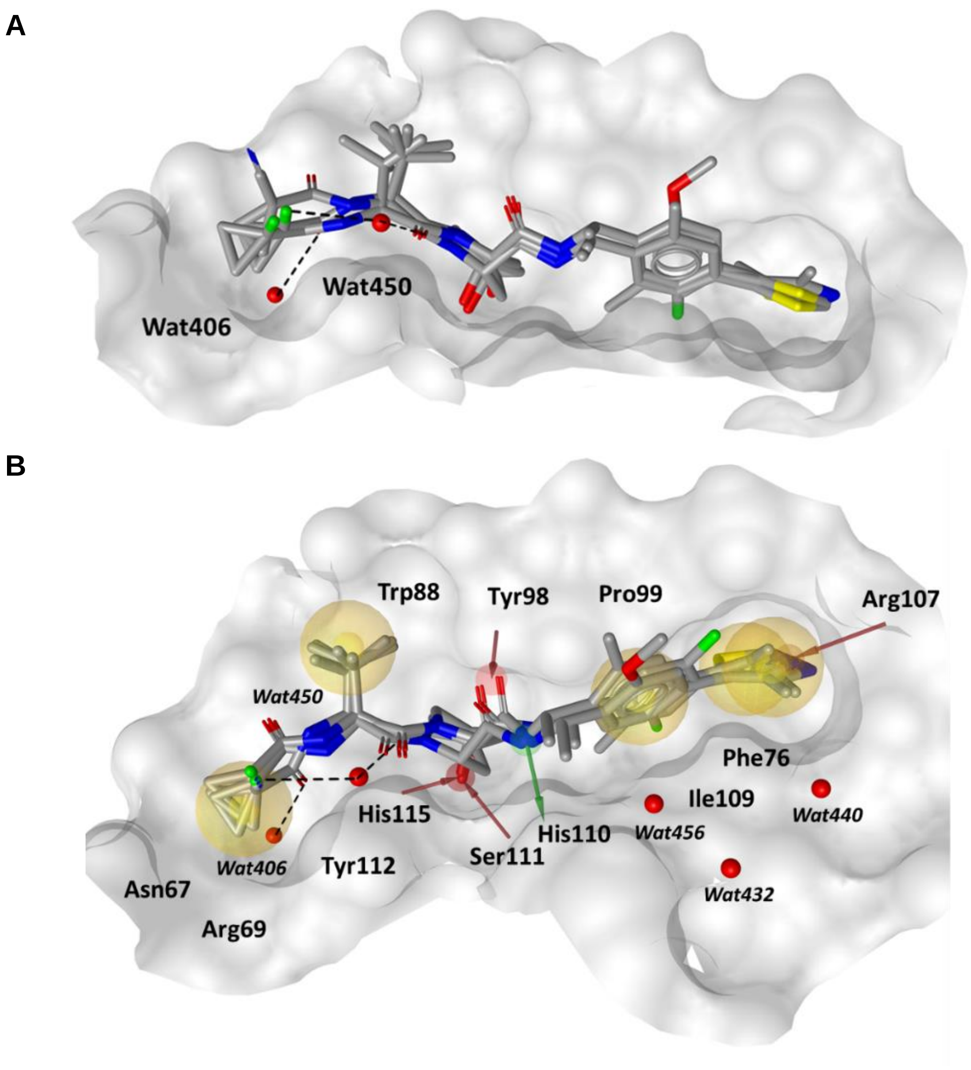

Figure S3. Overlay of predicted binding modes of compounds **24**, **32**, and **33** in the binding site of VCB obtained by docking settings with (A) two structural water molecules (red dots). (B) Docking settings obtained with five structural water molecules. Red and green arrows denote engaged hydrogen bond acceptors and donors, respectively, and yellow spheres indicate areas of hydrophobic interactions.

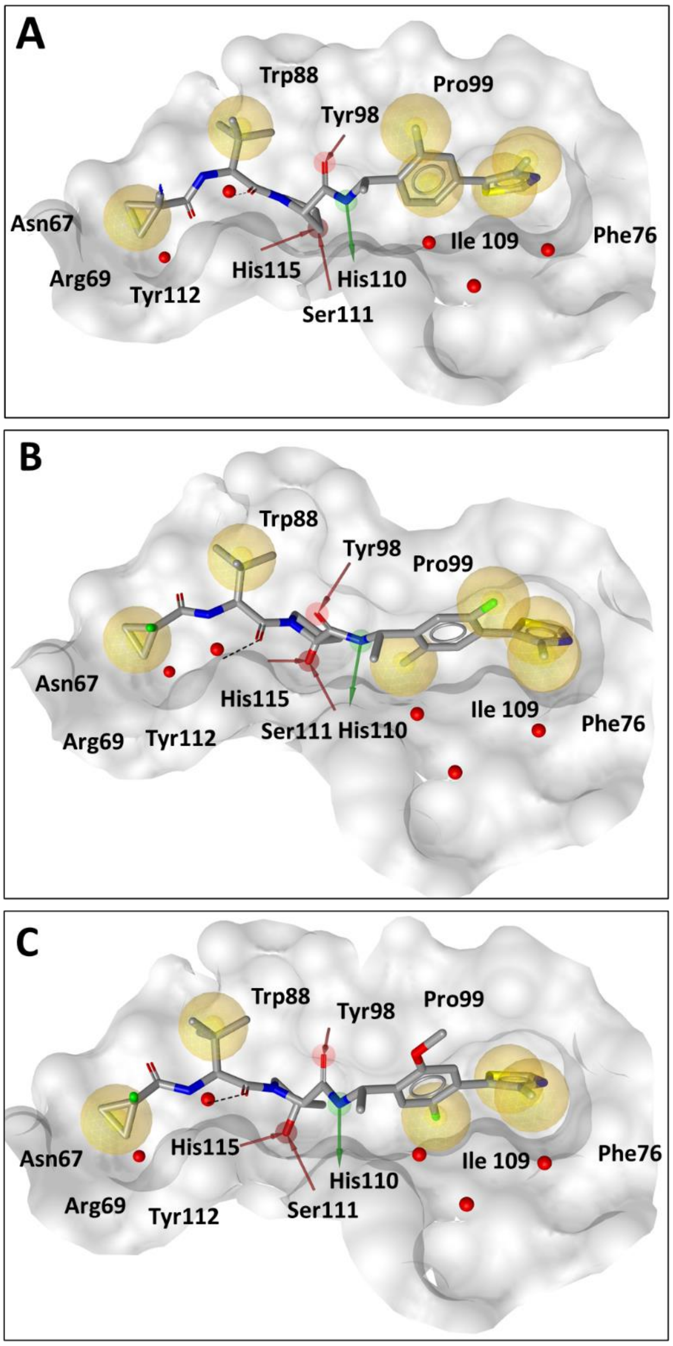

Figure S4. The structure-based pharmacophore as derived by molecular docking. Predicted binding modes of active compounds **24** (A), **32** (B), and **33** (C) in the binding site of VHL are shown with the considered five structural water molecules (red dots). Red and green arrows denote engaged hydrogen bond acceptors and donors, respectively, and yellow spheres indicate areas of hydrophobic interactions.

Table S1. Mean IC_{50} values obtained from the FP assay.

Cmpd	IC_{50} (nM) ± S.E.M
**1**	683 ± 56
**2**	668 ± 33
**3**	837 ± 35
**4**	1357 ± 56
**5**	1141 ± 131
**6**	2262 ± 73
**7**	742 ± 14
**8**	444 ± 18
**9**	1029 ± 127
**10**	21101 ± 2179
**11**	32130 ± 2148
**12**	11020 ± 747
**13**	42027 ± 1919
**14**	2741 ± 122
**15**	6533 ± 428
**16**	1444 ± 198
**17**	4557 ± 208
**18**	1653 ± 100
**19**	7445 ± 624
**20**	727 ± 7
**21**	1558 ± 250
**22**	2024 ± 271
**23**	1666 ± 375
**24**	329 ± 51
**25**	718 ± 95
**26**	426 ± 72
**27**	617 ± 117
**28**	310 ± 65
**29**	528 ± 106
**30**	144 ± 30
**31**	291 ± 61
**32**	220 ± 33
**33**	260 ± 38
**34**	15907 ± 934
**35**	16647 ± 2129
**36**	10633 ± 2028
**37**	4402 ±1062

Table S2. Second-order on-rate constants for association (k_{on}), and first-order off-rate constants for dissociation (k_{off}) for selected VHL ligands determined by SPR. Data are means of duplicate measurements.

Inhibitor	k_{on} ($M^{-1}s^{-1}$)	k_{off} (s^{-1})
**26**	1.68×10^{6}	6.15×10^{-2}
**27**	1.29×10^{6}	8.00×10^{-2}
**28**	1.32×10^{6}	4.19×10^{-2}
**29**	1.00×10^{6}	7.08×10^{-2}
**30**	1.41×10^{6}	3.55×10^{-2}
**31**	1.69×10^{6}	8.75×10^{-2}
**32**	1.12×10^{6}	5.54×10^{-2}
**33**	1.38×10^{6}	6.72×10^{-2}

30

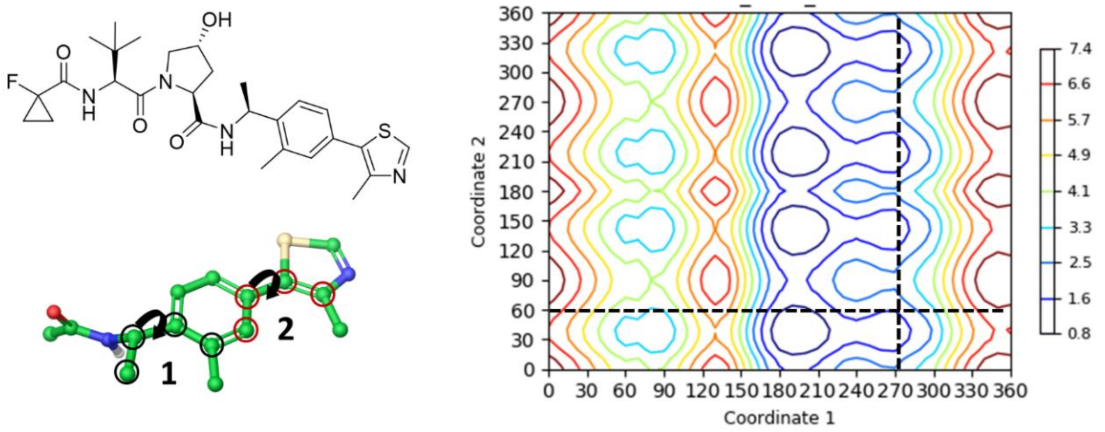

33

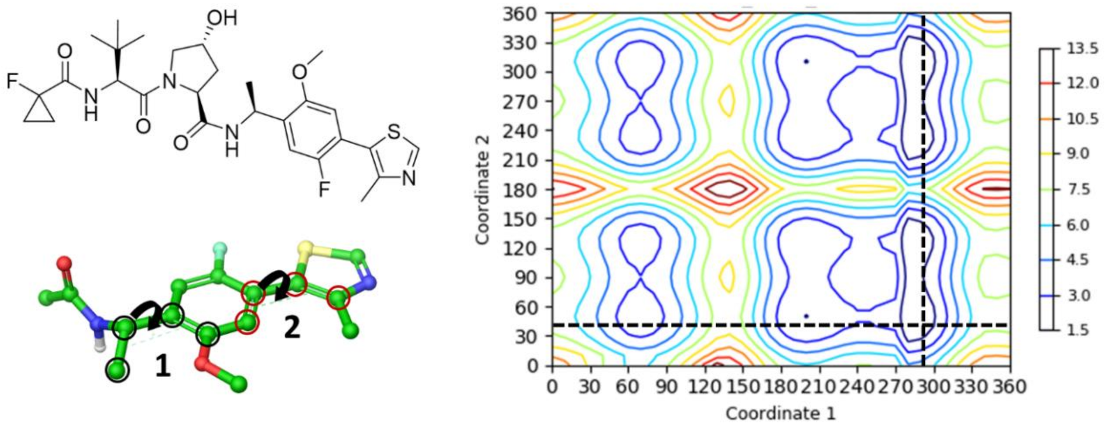

37

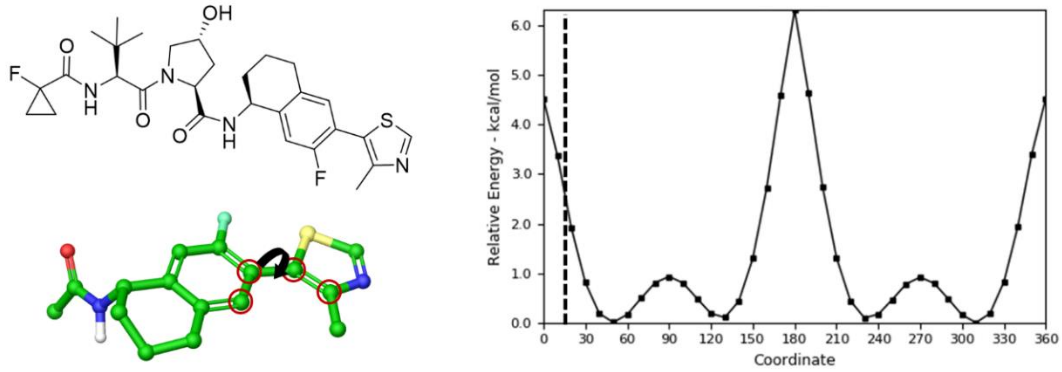

Figure S5. Two- and one-dimensional dihedral angle coordinate scans (MacroModel) compared to measured experimental dihedrals around the phenylene core. The color code of the 2D scans refers to relative energy (kcal/mol). The average of experimental values from the 4 protomers in each asymmetric unit is depicted by a dashed line. Viewing direction is from right to left. The direction of rotation is counterclockwise for a given dihedral. For both rotations (1 and 2), the front part of the structure is the rotator, the back part the stator. At 0°, the indicated atoms are in one plane. Average dihedral values: compound **30** (271°, 60°), compound **33** (290°, 41°), compound **37** (15°).

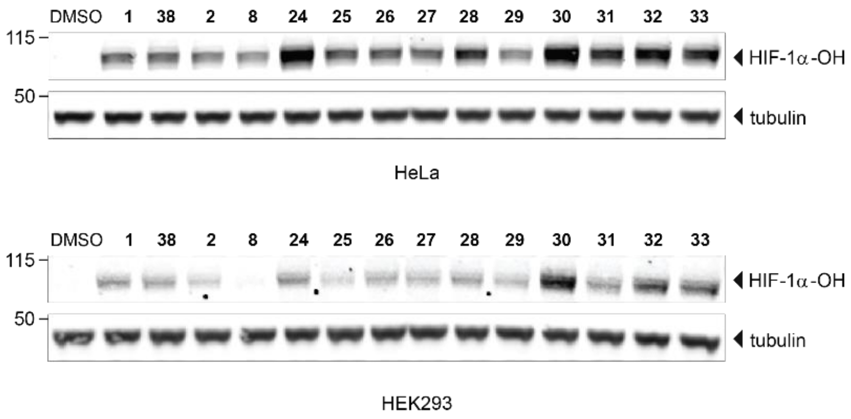

Figure S6. Immunoblots of HIF-1α-OH stabilization in HeLa and HEK 293 cells treated with 50 μM of selected VHL inhibitors and 1% DMSO for 2 h. The shown blots are representative of two independent experiments.

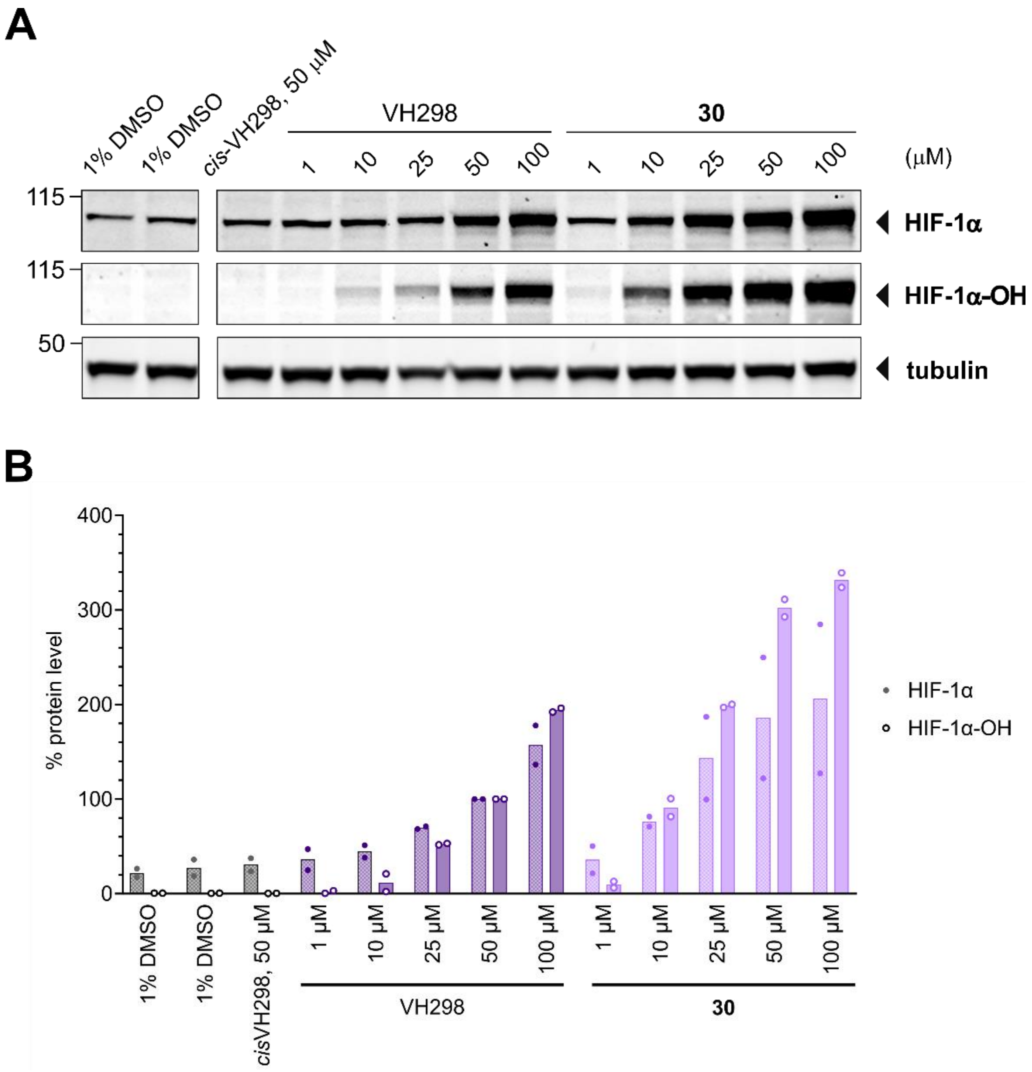

Figure S7. Dose-dependent treatments of HEK 293 cells with increasing concentrations of the VHL inhibitors **30** and **1** (VH298), 50 µM *cis*VH298 and 1% DMSO for 1 h. (A) Representative immunoblots of HIF-1α and HIF-1α-OH (Pro564) stabilization. (B) Quantification. HIF-1α/tubulin and HIF-1α-OH/tubulin protein ratios were normalized to those observed with **1** (VH298) at 50 µM (100%). Mean values of two biological replicates are depicted.

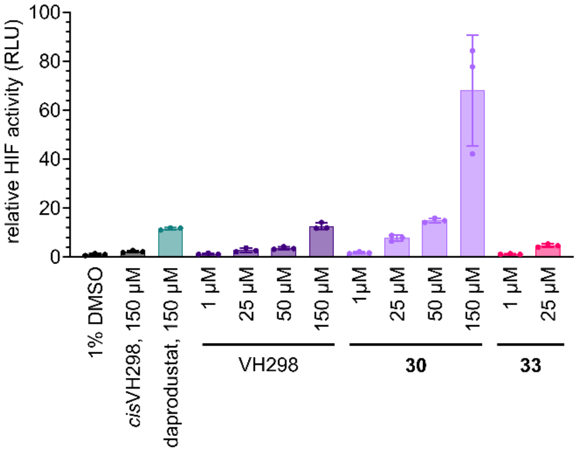

Figure S8. HRE-luciferase reporter assay with VHL inhibitors **30**, **33**, and **1** (VH298), 150 µM daprodustat, 150 µM *cis*VH298 and 1% DMSO. U2OS cells stably expressing an HRE-luciferase reporter plasmid were treated under indicated conditions for 32 h. The results of treatments with **33** at 50 µM and 150 µM had to be excluded due to apparent cytotoxicity of **33** at these concentrations. The graph depicts mean ± SEM of three biological replicates.

Scheme S1. Synthesis of building blocks 41α, 41β, 41γ, 41δ and 41ε[a]

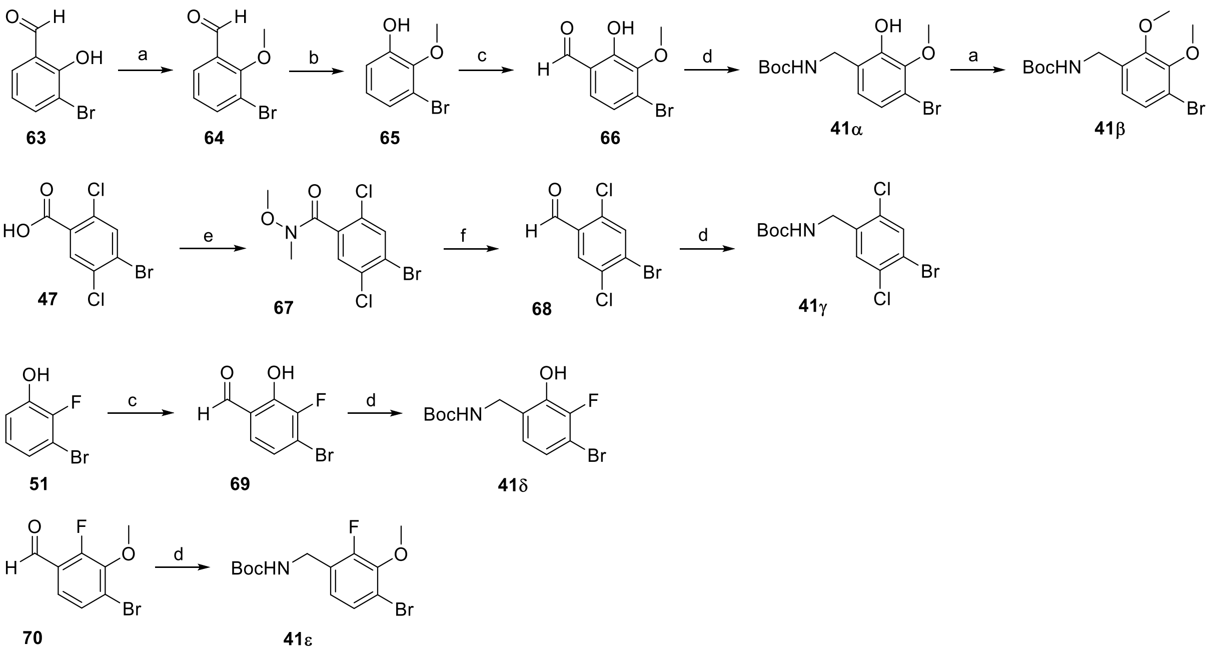

[a]Reagents and conditions: (a) (i) Li_2CO_3, DMF, 45 °C, 1 h; (ii) MeI, DMF, rt, 18 h; (b) (i) trifluoroacetic anhydride, H_2O_2, CH_2Cl_2, 0 °C, 1 h; (ii) **64**, KH_2PO_4, CH_2Cl_2, 0 °C; 30 min; (c) $(CH_2O)_n$, Et_3N, $MgCl_2$, THF, reflux, 18 h; (d) *tert*-butyl carbamate, Et_3SiH, TFA, CH_2Cl_2, MeCN, rt, 18 h; (e) *N*,*O*-dimethylhydroxylamine, EDC, Et_3N, CH_2Cl_2, rt, 18 h; (f) $LiAlH_4$, THF, 0 °C, 1 h.

Scheme S2. Unsuccessful transformations of 4-bromobenzylamine derivatives to Heck coupling products of type 42 because of low conversions (functional group tolerance) or limited starting material[a]

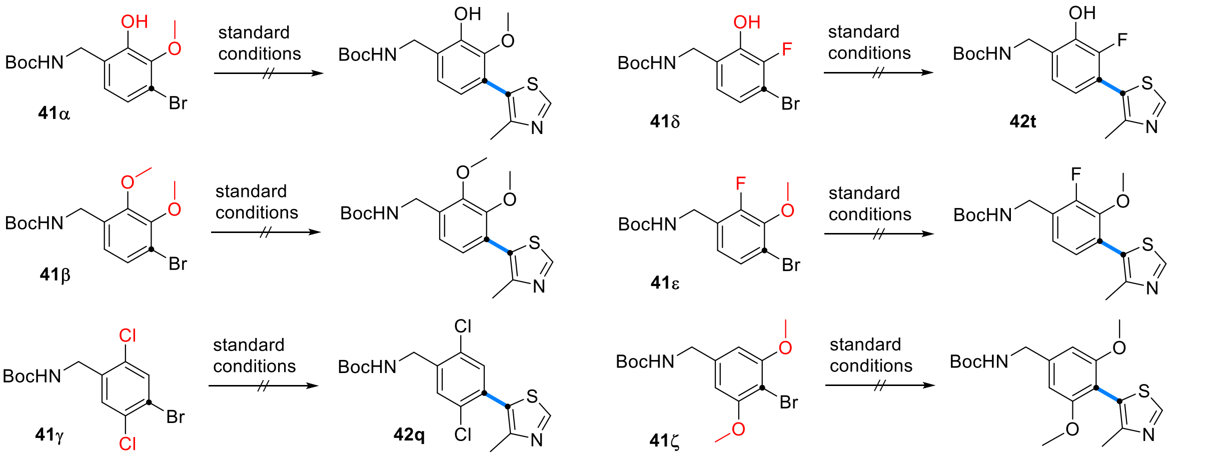

[a]Standard conditions: 4-methylthiazole, KOAc, $PdCl_2(PPh_3)_2$, dimethylacetamide, 130 °C, 4 h.

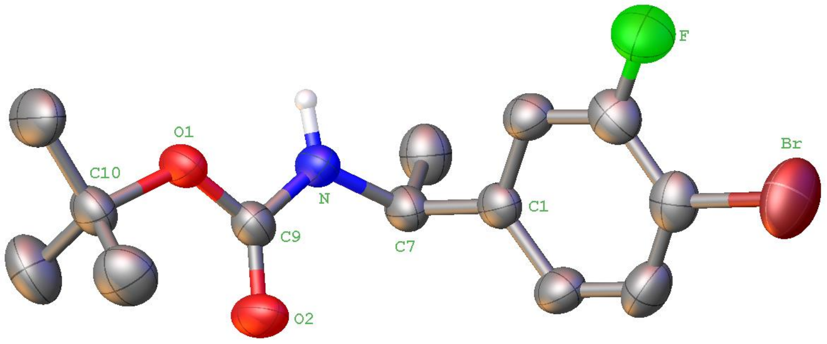

Figure S9. Molecular plot of the X-ray crystal structure of **59b** demonstrating the benzylic methyl group in the (*S*)-configuration.

^{1}H and ^{13}C NMR Assignments

*(2S,4R)-1-((S)-2-(1-Cyanocyclopropane-1-carboxamido)-3,3-dimethylbutanoyl)-4-hydroxy-N-(2-methyl-4-(4-methylthiazol-5-yl)benzyl)pyrrolidine-2-carboxamide (**2**).*

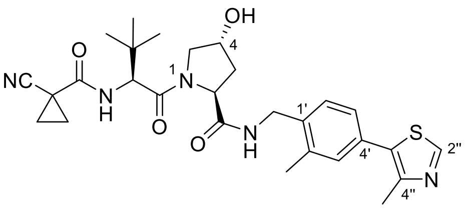

^{1}H NMR (600 MHz, DMSO-d_6) δ 0.95 (s, 9H, $C(CH_3)_3$), 1.46 – 1.53 (m, 2H, CH_2CCN), 1.58 – 1.66 (m, 2H, CH_2CCN), 1.88 – 1.94 (m, 1H, 3-H), 2.03 – 2.10 (m, 1H, 3-H), 2.30 (s, 3H, CH_3), 2.44 (s, 3H, CH_3), 3.12 – 3.16 (m, 1H, 5-H), 3.60 – 3.65 (m, 1H, 5-H), 4.22 (dd, J = 15.5, 5.4 Hz, 1H), 4.31 – 4.37 (m, 2H), 4.47 – 4.54 (m, 2H) (2-H, 4-H, NHC$\underline{H}$, NHC$\underline{H}_2$), 5.14 (d, J = 3.7 Hz, 1H, OH), 7.23 (dd, J = 7.9, 1.9 Hz, 1H), 7.28 (d, J = 2.0 Hz, 1H), 7.35 (d, J = 8.9 Hz, 1H), 7.41 (d, J = 7.8 Hz, 1H) (Ar-H, CONH), 8.49 (t, J = 5.7 Hz, 1H, CONH), 8.97 (s, 1H, 2″-H); ^{13}C NMR (126 MHz, DMSO-d_6) δ 12.4, 13.7 ($\underline{C}H_2CCN$), 15.9 ($CH_2\underline{C}CN$), 16.7 (CH_3), 18.1 (CH_3), 26.1 ($C(\underline{C}H_3)_3$), 36.2 ($\underline{C}(CH_3)_3$), 37.9 (C-3), 41.8 ($NHCH_2$), 56.6, 57.3, 58.7 (C-2, C-5, NHCH), 68.9 (C-4), 120.1 (CN), 126.1, 127.9, 129.8, 130.3, 131.1 (C-3′, C-4′, C-5′, C-6′, C-5″), 136.4 (C-2′), 136.9 (C-1′), 147.6 (C-4″), 151.3 (C-2″), 164.4, 168.6, 171.4 (CO).

*(2S,4R)-1-((S)-2-(1-Cyanocyclopropane-1-carboxamido)-3,3-dimethylbutanoyl)-4-hydroxy-N-(2-methoxy-4-(4-methylthiazol-5-yl)benzyl)pyrrolidine-2-carboxamide (**3**).*

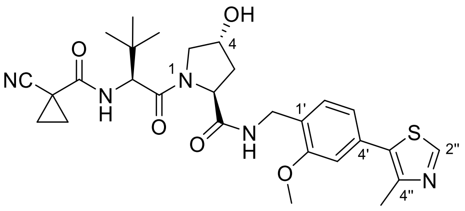

^{1}H NMR (600 MHz, DMSO-d_6) δ 0.95 (s, 9H, $C(CH_3)_3$), 1.46 – 1.53 (m, 2H, CH_2CCN), 1.59 – 1.66 (m, 2H, CH_2CCN), 1.89 – 1.94 (m, 1H, 3-H), 2.05 – 2.10 (m, 1H, 3-H), 2.47 (s, 3H, CH_3), 3.56 (d, J = 10.8 Hz, 1H, 5-H), 3.63 (dd, J = 10.8, 3.9 Hz, 1H, 5-H), 3.85 (s, 3H, OCH_3), 4.18 – 4.30 (m, 2H), 4.31 – 4.36 (m, 1H), 4.48 – 4.54 (m, 2H) (2-H, 4-H, NHC$\underline{H}$, NHC$\underline{H}_2$), 5.14 (d, J = 3.6 Hz, 1H, OH), 6.96 (dd, J = 7.7, 1.6 Hz, 1H), 7.01 – 7.04 (m, 1H), 7.38 (dd, J = 23.8, 8.3 Hz, 2H (Ar-H, CONH), 8.48 (t, J = 6.0 Hz, 1H, CONH), 8.98 (s, 1H, 2″-H); ^{13}C NMR (151 MHz, DMSO-d_6) δ 13.7 ($CH_2\underline{C}CN$), 16.0 (CH_3), 16.6, 16.8 ($\underline{C}H_2CCN$), 26.1 ($C(\underline{C}H_3)_3$), 36.2 ($\underline{C}(CH_3)_3$), 37.1 (C-3, $NHCH_2$), 37.8, 55.5, 56.6, 57.3, 58.8 (OCH_3, C-2, C-5, NHCH), 68.9 (C-4), 110.9 (C-3′), 120.1 (C-5′), 120.7 (CN), 126.8 (C-1′), 127.9 (C-6′), 131.0, 131.3 (C-4′, C-5″), 147.9 (C-4″), 151.4 (C-2″), 156.5 (C-2′), 164.4, 168.7, 171.7 (CO).

*(2S,4R)-1-((S)-2-(1-Cyanocyclopropane-1-carboxamido)-3,3-dimethylbutanoyl)-N-(2-fluoro-4-(4-methylthiazol-5-yl)benzyl)-4-hydroxypyrrolidine-2-carboxamide (**4**).*

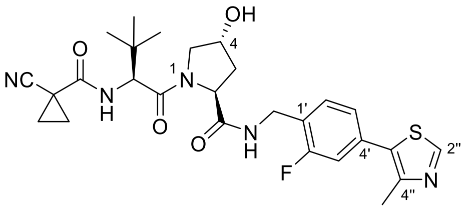

^{1}H NMR (600 MHz, DMSO-d_6) δ 0.94 (s, 9H, C(CH_3)$_3$), 1.45 – 1.53 (m, 2H, CH_2CCN), 1.58 – 1.67 (m, 2H, CH_2CCN), 1.86 – 1.92 (m, 1H, 3-H), 2.04 – 2.09 (m, 1H, 3-H), 2.46 (s, 3H, CH_3), 3.57 (dt, J = 11.0, 1.7 Hz, 1H, 5-H), 3.63 (dd, J = 10.8, 3.9 Hz, 1H, 5-H), 4.26 – 4.40 (m, 3H), 4.46 – 4.54 (m, 2H) (2-H, 4-H, NHC$\underline{H}$, NHC$\underline{H}_2$), 5.15 (d, J = 3.7 Hz, 1H, OH), 7.22 (dd, J = 7.9, 1.8 Hz, 1H), 7.27 – 7.40 (m, 2H), 7.54 (t, J = 8.0 Hz, 1H) (Ar-H, CONH), 8.64 (t, J = 5.9 Hz, 1H, CONH), 9.02 (s, 1H, 2″-H); ^{13}C NMR (151 MHz, DMSO-d_6) δ 13.7 ($CH_2\underline{C}$CN), 15.9 (CH_3), 16.6, 16.8 ($\underline{C}H_2$CCN), 26.0 (C($\underline{C}H_3$)$_3$), 35.9 (d, $^{3}J_{F,C}$ = 4.1 Hz, NHCH_2), 36.2 ($\underline{C}$(CH_3)$_3$), 37.8 (C-3), 56.6, 57.3, 58.7 (C-2, C-5, NHCH), 68.9 (C-4), 115.2 (d, $^{2}J_{F,C}$ = 22.5 Hz, C-3′), 120.1 (CN), 124.7 (d, $^{4}J_{F,C}$ = 2.4 Hz, C-5′), 125.7 (d, $^{2}J_{F,C}$ = 14.3 Hz, C-1′), 129.8 (d, $^{3}J_{F,C}$ = 5.2 Hz, C-6′), 132.0, 132.0 (C-4′, C-5″), 148.5 (C-4″), 152.0 (C-2″), 159.7 (d, $^{1}J_{F,C}$ = 245.4 Hz, C-2′), 164.4, 168.7, 171.8 (CO).

*(2S,4R)-N-(2-Chloro-4-(4-methylthiazol-5-yl)benzyl)-1-((S)-2-(1-cyanocyclopropane-1-carboxamido)-3,3-dimethylbutanoyl)-4-hydroxypyrrolidine-2-carboxamide (**5**).*

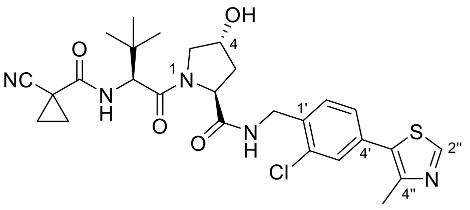

^{1}H NMR (600 MHz, DMSO-d_6) δ 0.94 (s, 9H, C(CH_3)$_3$), 1.47 – 1.54 (m, 2H, CH_2CCN), 1.59 – 1.67 (m, 2H, CH_2CCN), 1.92 (ddd, J = 13.1, 9.0, 4.5 Hz, 1H, 3-H), 2.06 – 2.12 (m, 1H, 3-H), 2.45 (s, 3H, CH_3), 3.55 – 3.59 (m, 1H, 5-H), 3.64 (dd, J = 10.8, 3.9 Hz, 1H, 5-H), 4.30 (dd, J = 16.4, 5.7 Hz, 1H), 4.34 – 4.37 (m, 1H), 4.39 (dd, J = 16.5, 6.2 Hz, 1H), 4.49 – 4.55 (m, 2H) (2-H, 4-H, NHC$\underline{H}$, NHC$\underline{H}_2$), 5.16 (d, J = 3.6 Hz, 1H, OH), 7.34 – 7.40 (m, 2H), 7.54 (d, J = 1.9 Hz, 1H), 7.61 (d, J = 8.0 Hz, 1H) (Ar-H, CONH), 8.72 (t, J = 6.0 Hz, 1H, CONH), 9.03 (s, 1H, 2″-H); ^{13}C NMR (151 MHz, DMSO-d_6) δ 13.7 ($CH_2\underline{C}$CN), 15.9 (CH_3), 16.6, 16.8 ($\underline{C}H_2$CCN), 26.1 (C($\underline{C}H_3$)$_3$), 36.2 ($\underline{C}$(CH_3)$_3$), 37.8 (C-3), 56.6, 57.3, 58.8 (C-2, C-5, NHCH), 68.9 (C-4), 120.1 (CN), 127.5, 128.9, 129.1, 129.5, 131.7, 132.2 (C-1′, C-3′, C-4′, C-5′, C-5″, C-6′), 136.0 (C-2′), 148.6 (C-4″), 152.1 (C-2″), 164.4, 168.8, 171.9 (CO); the signal for NHCH_2 is missing (overlapping solvent peak).

*(2S,4R)-1-((S)-2-(1-Cyanocyclopropane-1-carboxamido)-3,3-dimethylbutanoyl)-4-hydroxy-N-(3-methyl-4-(4-methylthiazol-5-yl)benzyl)pyrrolidine-2-carboxamide (**6**).*

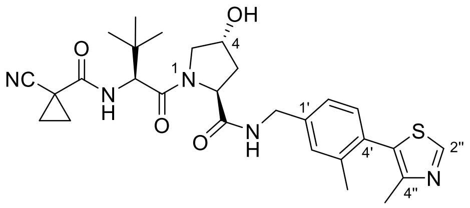

^{1}H NMR (600 MHz, DMSO-d_6) δ 0.95 (s, 9H, C(CH_3)$_3$), 1.46 – 1.52 (m, 2H, CH_2CCN), 1.58 – 1.65 (m, 2H, CH_2CCN), 1.88 – 1.93 (m, 1H, 3-H), 2.05 – 2.10 (m, 1H, 3-H), 2.12 (s, 3H, CH_3), 2.17 (s, 3H, CH_3), 3.55 – 3.59 (m, 1H, 5-H), 3.64 (dd, *J* = 10.8, 3.9 Hz, 1H, 5-H), 4.18 – 4.22 (m, 1H), 4.35 (ddt, *J* = 6.1, 4.2, 2.3 Hz, 1H), 4.41 (dd, *J* = 15.7, 6.5 Hz, 1H), 4.46 – 4.50 (m, 1H), 4.52 (d, *J* = 8.9 Hz, 1H) (2-H, 4-H, NHC$\underline{H}$, NHC$\underline{H_2}$), 5.15 (d, *J* = 3.6 Hz, 1H, OH), 7.14 – 7.20 (m, 2H), 7.33 (dd, *J* = 5.3, 3.6 Hz, 2H) (Ar-H, CONH), 8.59 (t, *J* = 6.0 Hz, 1H, CONH), 9.04 (s, 1H, 2″-H); ^{13}C NMR (151 MHz, DMSO-d_6) δ 13.6 ($CH_2\underline{C}$CN), 15.2 (CH_3), 16.6, 16.8 ($\underline{C}H_2$CCN), 19.7 (CH_3), 26.1 (C($\underline{C}H_3$)$_3$), 36.3 ($\underline{C}$(CH_3)$_3$), 37.8 (C-3), 41.7 (NHCH_2), 56.6, 57.3, 58.9 (C-2, C-5, NHCH), 68.9 (C-4), 120.0 (CN), 124.5, 128.7, 128.9, 129.6, 130.8 (C-3′, C-4′, C-5′, C-6′, C-5″), 136.9 (C-2′), 140.1 (C-1′), 149.0 (C-4″), 152.2 (C-2″), 164.3, 168.6, 171.6 (CO).

*(2S,4R)-1-((S)-2-(1-Cyanocyclopropane-1-carboxamido)-3,3-dimethylbutanoyl)-4-hydroxy-N-(3-methoxy-4-(4-methylthiazol-5-yl)benzyl)pyrrolidine-2-carboxamide (**7**).*

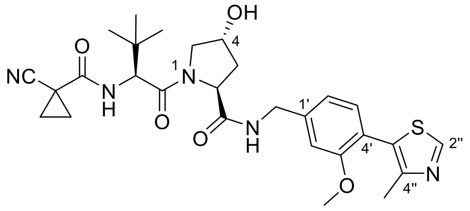

^{1}H NMR (600 MHz, DMSO-d_6) δ 0.95 (s, 9H, C(CH_3)$_3$), 1.45 – 1.53 (m, 2H, CH_2CCN), 1.59 – 1.67 (m, 2H, CH_2CCN), 1.87 – 1.94 (m, 1H, 3-H), 2.06 – 2.12 (m, 1H, 3-H), 2.27 (s, 3H, CH_3), 3.57 (d, *J* = 10.9 Hz, 1H, 5-H), 3.64 (dd, *J* = 10.9, 3.8 Hz, 1H, 5-H), 3.84 (s, 3H, OCH_3), 4.19 (dd, *J* = 15.8, 5.1 Hz, 1H), 4.33 – 4.38 (m, 1H), 4.46 – 4.53 (m, 3H) (2-H, 4-H, NHC$\underline{H}$, NHC$\underline{H_2}$), 5.16 (d, *J* = 3.6 Hz, 1H, OH), 6.93 – 6.97 (m, 1H), 7.14 – 7.17 (m, 1H), 7.22 (d, *J* = 7.7 Hz, 1H), 7.28 (d, *J* = 8.9 Hz, 1H) (Ar-H, CONH), 8.64 (dd, *J* = 6.9, 5.1 Hz, 1H, CONH), 8.98 (s, 1H, 2″-H); ^{13}C NMR (151 MHz, DMSO-d_6) δ 13.6 ($CH_2\underline{C}$CN), 15.9 (CH_3), 16.6, 16.8 ($\underline{C}H_2$CCN), 26.1 (C($\underline{C}H_3$)$_3$), 36.4 ($\underline{C}$(CH_3)$_3$), 37.8, 41.8 (C-3, NHCH_2), 55.6, 56.7, 57.4, 59.0 (OCH_3, C-2, C-5, NHCH), 68.9 (C-4), 110.2 (C-2′), 117.9, 118.9 (C-5′, C-6′), 120.1 (CN), 126.7 (C-1′), 131.1 (C-4′), 142.0 (C-5″), 149.3 (C-4″), 151.9 (C-2″), 156.6 (C-3′), 164.3, 168.7, 171.5 (CO).

*(2S,4R)-1-((S)-2-(1-Cyanocyclopropane-1-carboxamido)-3,3-dimethylbutanoyl)-N-(3-fluoro-4-(4-methylthiazol-5-yl)benzyl)-4-hydroxypyrrolidine-2-carboxamide (**8**).*

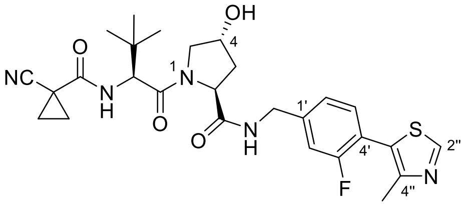

^{1}H NMR (600 MHz, DMSO-d_6) δ 0.95 (s, 9H, C(CH_3)$_3$), 1.46 – 1.53 (m, 2H, CH_2CCN), 1.58 – 1.66 (m, 2H, CH_2CCN), 1.88 – 1.94 (m, 1H, 3-H), 2.05 – 2.11 (m, 1H, 3-H), 2.32 (d, J = 1.1 Hz, 3H, CH_3), 3.56 – 3.59 (m, 1H, 5-H), 3.64 (dd, J = 10.8, 3.8 Hz, 1H, 5-H), 4.24 (dd, J = 16.1, 5.6 Hz, 1H), 4.34 – 4.37 (m, 1H), 4.43 – 4.54 (m, 3H) (2-H, 4-H, NHC$\underline{H}$, NHC$\underline{H}_2$), 5.16 (d, J = 3.6 Hz, 1H, OH), 7.22 – 7.24 (m, 1H), 7.31 – 7.36 (m, 2H), 7.40 (t, J = 7.8 Hz, 1H) (Ar-H, CONH), 8.70 (t, J = 6.1 Hz, 1H, CONH), 9.09 (s, 1H, 2″-H); ^{13}C NMR (151 MHz, DMSO-d_6) δ 13.7 ($CH_2\underline{C}$CN), 15.7 (CH_3), 16.6, 16.8 ($\underline{C}H_2$CCN), 26.0 (C($\underline{C}H_3$)$_3$), 36.2 ($\underline{C}$(CH_3)$_3$), 37.8, 41.4 (C-3, NHCH_2), 56.6, 57.3, 58.9 (C-2, C-5, NHCH), 68.9 (C-4), 114.4 (d, $^2J_{F,C}$ = 23.0 Hz, C-2′), 116.9 (d, $^2J_{F,C}$ = 15.3 Hz, C-4′), 120.1 (CN), 123.1 (d, $^3J_{F,C}$ = 2.9 Hz, C-5′), 123.8 (C-5″), 131.7 (d, $^4J_{F,C}$ = 2.3 Hz, C-6′), 143.2 (d, $^3J_{F,C}$ = 7.6 Hz, C-1′), 150.1 (C-4″), 153.1 (C-2″), 158.9 (d, $^1J_{F,C}$ = 246.6 Hz, C-3′), 164.4, 168.7, 171.9 (CO).

*(2S,4R)-N-(3-Chloro-4-(4-methylthiazol-5-yl)benzyl)-1-((S)-2-(1-cyanocyclopropane-1-carboxamido)-3,3-dimethylbutanoyl)-4-hydroxypyrrolidine-2-carboxamide (**9**).*

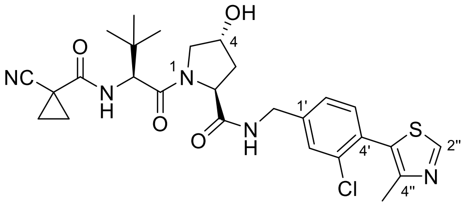

^{1}H NMR (600 MHz, DMSO-d_6) δ 0.95 (s, 9H, C(CH_3)$_3$), 1.46 – 1.54 (m, 2H, CH_2CCN), 1.58 – 1.66 (m, 2H, CH_2CCN), 1.90 (ddd, J = 13.1, 9.1, 4.4 Hz, 1H, 3-H), 2.05 – 2.11 (m, 1H, 3-H), 2.23 (s, 3H, CH_3), 3.54 – 3.58 (m, 1H, 5-H), 3.64 (dd, J = 10.8, 3.8 Hz, 1H, 5-H), 4.24 (dd, J = 16.0, 5.4 Hz, 1H), 4.33 – 4.38 (m, 1H), 4.43 – 4.53 (m, 3H) (2-H, 4-H, NHC$\underline{H}$, NHC$\underline{H}_2$), 5.15 (d, J = 3.6 Hz, 1H, OH), 7.31 – 7.40 (m, 3H), 7.60 (d, J = 1.6 Hz, 1H) (Ar-H, CONH), 8.69 (t, J = 6.1 Hz, 1H, CONH), 9.09 (s, 1H, 2″-H); ^{13}C NMR (151 MHz, DMSO-d_6) δ 13.6 ($CH_2\underline{C}$CN), 15.5 (CH_3), 16.6, 16.8 ($\underline{C}H_2$CCN), 26.1 (C($\underline{C}H_3$)$_3$), 36.3 ($\underline{C}$(CH_3)$_3$), 37.8 (C-3), 41.3 (NHCH_2), 56.6, 57.3, 58.9 (C-2, C-5, NHCH), 68.9 (C-4), 120.0 (CN), 125.9, 127.3, 128.0, 128.2, 132.4, 133.3 (C-1′, C-2′, C-4′, C-5′, C-5″, C-6′), 142.6 (C-3′), 150.2 (C-4″), 152.9 (C-2″), 164.3, 168.7, 171.8 (CO).

*(2S,4R)-1-((S)-2-(1-Cyanocyclopropane-1-carboxamido)-3,3-dimethylbutanoyl)-N-(2,6-dimethyl-4-(4-methylthiazol-5-yl)benzyl)-4-hydroxypyrrolidine-2-carboxamide (**10**).*

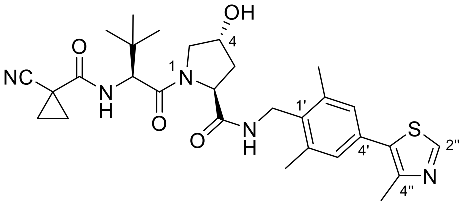

^{1}H NMR (600 MHz, DMSO-d_6) δ 0.96 (s, 9H, $C(CH_3)_3$), 1.44 – 1.52 (m, 2H, CH_2CCN), 1.56 – 1.66 (m, 2H, CH_2CCN), 1.82 – 1.89 (m, 1H, 3-H), 1.96 – 2.03 (m, 1H, 3-H), 2.35 (s, 6H, Ar-CH_3), 2.46 (s, 3H, CH_3), 3.51 – 3.56 (m, 1H, 5-H), 3.64 (dd, *J* = 10.8, 4.0 Hz, 1H, 5-H), 4.24 (dd, *J* = 14.0, 4.5 Hz, 1H), 4.30 – 4.34 (m, 1H), 4.38 – 4.42 (m, 2H), 4.51 (d, *J* = 8.9 Hz, 1H) (2-H, 4-H, $NHC\underline{H}$, $NHC\underline{H}_2$), 5.10 (d, *J* = 3.6 Hz, 1H, OH), 7.16 (s, 2H, Ar-H), 7.28 (d, *J* = 8.9 Hz, 1H, CONH), 8.08 (t, *J* = 5.0 Hz, 1H, CONH), 8.97 (s, 1H, 2″-H); ^{13}C NMR (151 MHz, DMSO-d_6) δ 13.7 ($CH_2\underline{C}CN$), 16.0 (CH_3), 16.6, 16.8 ($\underline{C}H_2CCN$), 19.3 (Ar-CH_3), 26.1 ($C(\underline{C}H_3)_3$), 36.2 ($\underline{C}(CH_3)_3$), 36.7, 37.9 (C-3, $NHCH_2$), 56.6, 57.3, 58.6 (C-2, C-5, NHCH), 68.8 (C-4), 120.1 (CN), 128.3, 130.3, 131.1, 134.7, 138.2 (C-1′, C-2′, C-3′, C-4′, C-5′, C-5″, C-6′), 147.7 (C-4″), 151.4 (C-2″), 164.4, 168.5, 170.9 (CO).

*(2S,4R)-1-((S)-2-(1-Cyanocyclopropane-1-carboxamido)-3,3-dimethylbutanoyl)-N-(2,6-dimethoxy-4-(4-methylthiazol-5-yl)benzyl)-4-hydroxypyrrolidine-2-carboxamide (**11**).*

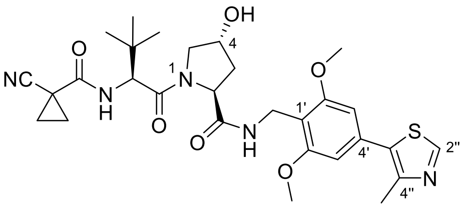

^{1}H NMR (500 MHz, DMSO-d_6) δ 0.94 (s, 9H, $C(CH_3)_3$), 1.43 – 1.52 (m, 2H, CH_2CCN), 1.55 – 1.67 (m, 2H, CH_2CCN), 1.84 – 1.99 (m, 2H, 3-H), 3.51 (d, *J* = 9.7 Hz, 1H, 5-H), 3.61 (dd, *J* = 10.8, 4.3 Hz, 1H, 5-H), 3.82 (s, 6H, OCH_3), 4.20 (dd, *J* = 13.1, 3.8 Hz, 1H), 4.27 – 4.32 (m, 1H), 4.35 (dd, *J* = 13.1, 5.6 Hz, 1H), 4.44 (t, *J* = 7.9 Hz, 1H), 4.50 (d, *J* = 8.9 Hz, 1H) (2-H, 4-H, $NHC\underline{H}$, $NHC\underline{H}_2$), 5.06 (d, *J* = 3.8 Hz, 1H, OH), 6.72 (s, 2H, Ar-H), 7.26 (d, *J* = 8.9 Hz, 1H, CONH), 7.65 (t, *J* = 4.7 Hz, 1H, CONH), 9.00 (s, 1H, 2″-H); ^{13}C NMR (126 MHz, DMSO-d_6) δ 13.6 ($CH_2\underline{C}CN$), 16.0 (CH_3), 16.6, 16.7 ($\underline{C}H_2CCN$), 26.0 ($C(\underline{C}H_3)_3$), 31.4 ($NHCH_2$), 36.1 ($\underline{C}(CH_3)_3$), 37.5 (C-3), 56.0, 57.3, 58.4 (C-2, C-5, NHCH), 68.7 (C-4), 105.1, 113.5 (C-3′, C-4’, C-5′), 120.0 (CN), 131.4, 132.2 (C-1′, C-5″), 148.2 (C-4″), 151.6 (C-2″), 158.5 (C-2′, C-6′), 164.3, 168.6, 170.5 (CO); the signal for CH_3 is missing (overlapping solvent peak).

*(2S,4R)-1-((S)-2-(1-Cyanocyclopropane-1-carboxamido)-3,3-dimethylbutanoyl)-N-(2,6-difluoro-4-(4-methylthiazol-5-yl)benzyl)-4-hydroxypyrrolidine-2-carboxamide (**12**).*

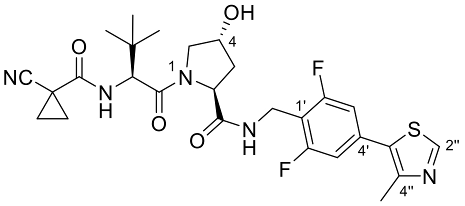

^{1}H NMR (500 MHz, DMSO-d_6) δ 0.92 (s, 9H, C(CH_3)$_3$), 1.43 – 1.53 (m, 2H, CH_2CCN), 1.56 – 1.67 (m, 2H, CH_2CCN), 1.79 – 1.87 (m, 1H, 3-H), 1.94 – 2.01 (m, 1H, 3-H), 2.48 (s, 3H, CH_3), 3.52 (d, *J* = 10.8 Hz, 1H, 5-H), 3.61 (dd, *J* = 10.8, 4.0 Hz, 1H, 5-H), 4.23 – 4.32 (m, 2H), 4.37 – 4.46 (m, 2H), 4.49 (d, *J* = 8.9 Hz, 1H) (2-H, 4-H, NHC$\underline{H}$, NHC$\underline{H}_2$), 5.09 (d, *J* = 3.7 Hz, 1H, OH), 7.21 – 7.29 (m, 3H) (Ar-H, CONH), 8.38 (t, *J* = 5.3 Hz, 1H, CONH), 9.06 (s, 1H, 2″-H); ^{13}C NMR (126 MHz, DMSO-d_6) δ 13.6 ($CH_2\underline{C}$CN), 16.0 (CH_3), 16.6, 16.7 ($\underline{C}H_2$CCN), 26.1 (C($\underline{C}H_3$)$_3$), 30.4 (NHCH_2), 36.1 ($\underline{C}$(CH_3)$_3$), 37.6 (C-3), 56.5, 57.3, 58.5 (C-2, C-5, NHCH), 68.7 (C-4), 111.9 (d, $^{2}J_{F,C}$ = 14.1 Hz), 111.9 (d, $^{2}J_{F,C}$ = 27.3 Hz, C-3′, C-5′), 113.6 (t, $^{2}J_{F,C}$ = 19.8 Hz, C-1′), 120.0 (CN), 128.8 (C-4″), 133.2 (t, $^{3}J_{F,C}$ = 11.0 Hz, C-4′), 149.3 (C-5″), 152.6 (C-2″), 160.9 (dd, $^{1}J_{F,C}$ = 248.7 Hz, $^{3}J_{F,C}$ = 9.7 Hz, C-2′, C-6′), 164.3, 168.5, 170.9 (CO).

*(2S,4R)-1-((S)-2-(1-Cyanocyclopropane-1-carboxamido)-3,3-dimethylbutanoyl)-N-(2,6-dichloro-4-(4-methylthiazol-5-yl)benzyl)-4-hydroxypyrrolidine-2-carboxamide (**13**).*

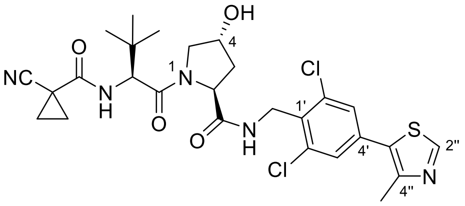

^{1}H NMR (500 MHz, DMSO-d_6) δ 0.96 (s, 9H, C(CH_3)$_3$), 1.45 – 1.53 (m, 2H, CH_2CCN), 1.57 – 1.65 (m, 2H, CH_2CCN), 1.85 – 1.92 (m, 1H, 3-H), 1.96 – 2.03 (m, 1H, 3-H), 2.48 (s, 3H, CH_3), 3.51 – 3.55 (m, 1H, 5-H), 3.63 (dd, *J* = 10.7, 4.1 Hz, 1H, 5-H), 4.29 – 4.34 (m, 1H), 4.41 – 4.52 (m, 3H), 4.63 (dd, *J* = 13.8, 5.8 Hz, 1H) (2-H, 4-H, NHC$\underline{H}$, NHC$\underline{H}_2$), 5.09 (d, *J* = 3.7 Hz, 1H, OH), 7.27 (d, *J* = 8.8 Hz, 1H, CONH), 7.61 (s, 2H, Ar-H), 8.20 (dd, *J* = 5.7, 3.7 Hz, 1H, CONH), 9.08 (s, 1H, 2″-H); ^{13}C NMR (126 MHz, DMSO-d_6) δ 13.6 ($CH_2\underline{C}$CN), 15.9 (CH_3), 16.6, 16.8 ($\underline{C}H_2$CCN), 26.1 (C($\underline{C}H_3$)$_3$), 36.1 ($\underline{C}$(CH_3)$_3$), 37.8, 38.4 (C-3, NHCH_2), 56.5, 57.3, 58.5 (C-2, C-5, NHCH), 68.7 (C-4), 120.0 (CN), 128.0, 128.4, 132.9, 133.5, 135.9 (C-1′, C-2′, C-3′, C-4′, C-5′, C-5″, C-6′), 149.6 (C-4″), 152.9 (C-2″), 164.3, 168.5, 170.8 (CO).

*(2S,4R)-1-((S)-2-(1-Cyanocyclopropane-1-carboxamido)-3,3-dimethylbutanoyl)-N-(2,5-dimethyl-4-(4-methylthiazol-5-yl)benzyl)-4-hydroxypyrrolidine-2-carboxamide (**14**).*

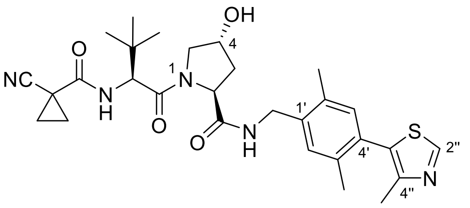

^{1}H NMR (500 MHz, DMSO-d_6) δ 0.95 (s, 9H, C(CH_3)$_3$), 1.46 – 1.53 (m, 2H, CH_2CCN), 1.58 – 1.67 (m, 2H, CH_2CCN), 1.91 (ddd, *J* = 13.2, 9.0, 4.5 Hz, 1H, 3-H), 2.05 – 2.08 (m, 1H, 3-H), 2.09 (s, 3H, CH_3), 2.17 (s, 3H, CH_3), 2.22 (s, 3H, CH_3), 3.56 (d, *J* = 10.7 Hz, 1H, 5-H), 3.64 (dd, *J* = 10.8, 3.9 Hz, 1H, 5-H), 4.15 (dd, *J* = 15.6, 5.2 Hz, 1H), 4.32 – 4.38 (m, 2H), 4.48 – 4.53 (m, 2H) (2-H, 4-H, NHC$\underline{H}$, NHC$\underline{H}_2$), 5.13 (d, *J* = 3.6 Hz, 1H, OH), 7.04 (s, 1H, Ar-H), 7.32 (d, *J* = 8.8 Hz, 1H, CONH), 7.36 (s, 1H, Ar-H), 8.47 (t, *J* = 5.8 Hz, 1H, CONH), 9.02 (s, 1H, 2″-H); ^{13}C NMR (126 MHz, DMSO-d_6) δ 13.6 ($CH_2\underline{C}$CN), 15.2 (CH_3), 16.6, 16.8 ($\underline{C}H_2$CCN), 17.8, 19.1 (Ar-CH_3), 26.0 (C($\underline{C}H_3$)$_3$), 36.3 ($\underline{C}$(CH_3)$_3$), 37.9 (C-3), 56.6, 57.3, 58.9 (C-2, C-5, NHCH), 68.9 (C-4), 120.0 (CN), 128.6, 129.3, 129.6, 132.1, 132.9, 134.1 (C-2′, C-3′, C-4′, C-5′, C-5″, C-6′), 137.5 (C-1′), 148.8 (C-4″), 152.0 (C-2″), 164.3, 168.6, 171.4 (CO); the signal for NHCH_2 is missing (overlapping solvent peak).

*(2S,4R)-1-((S)-2-(1-Cyanocyclopropane-1-carboxamido)-3,3-dimethylbutanoyl)-N-(2,5-dimethoxy-4-(4-methylthiazol-5-yl)benzyl)-4-hydroxypyrrolidine-2-carboxamide (**15**).*

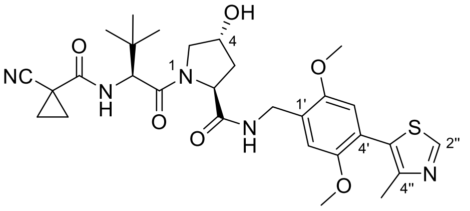

^{1}H NMR (500 MHz, DMSO-d_6) δ 0.93 (s, 9H, C(CH_3)$_3$), 1.46 – 1.52 (m, 2H, CH_2CCN), 1.59 – 1.67 (m, 2H, CH_2CCN), 1.88 – 1.95 (m, 1H, 3-H), 2.07 – 2.13 (m, 1H, 3-H), 2.31 (s, 3H, CH_3), 3.57 (d, *J* = 10.9 Hz, 1H, 5-H), 3.64 (dd, *J* = 10.9, 3.7 Hz, 1H, 5-H), 3.78 (s, 3H, OCH_3), 3.83 (s, 3H, OCH_3), 4.13 (dd, *J* = 16.5, 5.0 Hz, 1H), 4.34 – 4.37 (m, 1H), 4.41 (dd, *J* = 16.6, 7.0 Hz, 1H), 4.49 – 4.55 (m, 2H) (2-H, 4-H, NHC$\underline{H}$, NHC$\underline{H}_2$), 5.15 (d, *J* = 3.6 Hz, 1H, OH), 6.90 (s, 1H, Ar-H), 7.23 (s, 1H, Ar-H), 7.25 (d, *J* = 8.9 Hz, 1H, CONH), 8.55 (dd, *J* = 7.0, 5.1 Hz, 1H, CONH), 8.98 (s, 1H, 2″-H); ^{13}C NMR (126 MHz, DMSO-d_6) δ 13.5 ($CH_2\underline{C}$CN), 15.9 (CH_3), 16.6, 16.9 ($\underline{C}H_2$CCN), 26.0 (C($\underline{C}H_3$)$_3$), 36.4 ($\underline{C}$(CH_3)$_3$), 36.9, 37.8 (C-3, NHCH_2), 55.9, 56.2, 56.7, 57.4, 59.1 (OCH_3, C-2, C-5, NHCH), 68.9 (C-4), 111.7, 113.6 (C-3′, C-6′), 118.0, 120.0, 126.8, 128.9 (C-1′, C-4′, C-5″, CN), 149.4, 149.9, 150.6, 151.9 (C-2′, C-2″, C-4″, C-5′), 164.2, 168.7, 171.6 (CO).

*(2S,4R)-1-((S)-2-(1-Cyanocyclopropane-1-carboxamido)-3,3-dimethylbutanoyl)-N-(2,5-difluoro-4-(4-methylthiazol-5-yl)benzyl)-4-hydroxypyrrolidine-2-carboxamide (**16**).*

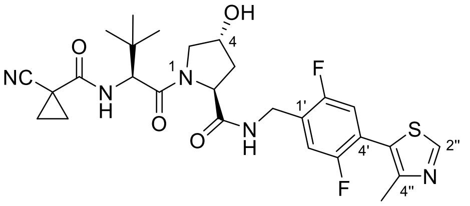

^{1}H NMR (600 MHz, DMSO-d_6) δ 0.93 (s, 9H, $C(CH_3)_3$), 1.46 – 1.53 (m, 2H, CH_2CCN), 1.58 – 1.66 (m, 2H, CH_2CCN), 1.87 – 1.93 (m, 1H, 3-H), 2.05 – 2.10 (m, 1H, 3-H), 2.34 (d, *J* = 1.0 Hz, 3H, CH_3), 3.55 – 3.59 (m, 1H, 5-H), 3.64 (dd, *J* = 10.8, 3.9 Hz, 1H, 5-H), 4.23 (dd, *J* = 16.3, 5.3 Hz, 1H), 4.34 – 4.38 (m, 1H), 4.41 – 4.53 (m, 3H) (2-H, 4-H, NHC$\underline{H}$, NHC$\underline{H}_2$), 5.17 (d, *J* = 3.6 Hz, 1H, OH), 7.35 (d, *J* = 8.9 Hz, 1H), 7.38 (dd, *J* = 9.9, 6.0 Hz, 1H), 7.49 (dd, *J* = 10.4, 6.2 Hz, 1H) (Ar-H, CONH), 8.76 (t, *J* = 6.0 Hz, 1H, CONH), 9.12 (s, 1H, 2″-H); ^{13}C NMR (151 MHz, DMSO-d_6) δ 13.7 ($CH_2\underline{C}CN$), 15.7 (d, $^5J_{F,C}$ = 2.0 Hz, CH_3), 16.6, 16.8 ($\underline{C}H_2CCN$), 26.0 ($C(\underline{C}H_3)_3$), 35.9 (d, $^3J_{F,C}$ = 3.6 Hz, $NHCH_2$), 36.2 ($\underline{C}(CH_3)_3$), 37.7 (C-3), 56.6, 57.3, 58.9 (C-2, C-5, NHCH), 68.9 (C-4), 116.1 (d, $^3J_{F,C}$ = 5.1 Hz), 116.3 (d, $^3J_{F,C}$ = 4.9 Hz, C-5′, C-6′), 117.7 (dd, *J* = 25.1, 2.2 Hz), 118.4 (dd, $^2J_{F,C}$ = 18.2 Hz, $^3J_{F,C}$ = 9.1 Hz, C-4′), 120.1 (CN), 122.7 (C-5″), 129.0 (dd, $^2J_{F,C}$ = 17.2 Hz, $^3J_{F,C}$ = 7.9 Hz, C-1′), 150.7 (C-4″), 153.6 (C-2″), 155.2 (dd, $^1J_{F,C}$ = 242.5 Hz, $^2J_{F,C}$ = 25.9 Hz, C-2′, C-3′), 164.4, 168.8, 172.2 (CO).

*(2S,4R)-1-((S)-2-(1-Cyanocyclopropane-1-carboxamido)-3,3-dimethylbutanoyl)-N-(2,5-dichloro-4-(4-methylthiazol-5-yl)benzyl)-4-hydroxypyrrolidine-2-carboxamide (**17**).*

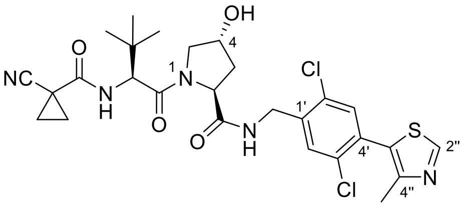

^{1}H NMR (600 MHz, DMSO-d_6) δ 0.93 (s, 9H, $C(CH_3)_3$), 1.45 – 1.52 (m, 2H, CH_2CCN), 1.58 – 1.66 (m, 2H, CH_2CCN), 1.87 – 1.94 (m, 1H, 3-H), 2.06 – 2.13 (m, 1H, 3-H), 2.24 (s, 3H, CH_3), 3.57 (d, *J* = 10.8 Hz, 1H, 5-H), 3.64 (dd, *J* = 10.9, 3.7 Hz, 1H, 5-H), 4.22 (dd, *J* = 16.7, 5.2 Hz, 1H), 4.34 – 4.39 (m, 1H), 4.44 – 4.54 (m, 3H) (2-H, 4-H, NHC$\underline{H}$, NHC$\underline{H}_2$), 5.18 (d, *J* = 3.5 Hz, 1H, OH), 7.33 (d, *J* = 8.9 Hz, 1H, CONH), 7.59 (s, 1H, Ar-H), 7.87 (s, 1H, Ar-H), 8.82 (dd, *J* = 6.8, 5.3 Hz, 1H, CONH), 9.12 (s, 1H, 2″-H); ^{13}C NMR (151 MHz, DMSO-d_6) δ 13.6 ($CH_2\underline{C}CN$), 15.4 (CH_3), 16.6, 16.8 ($\underline{C}H_2CCN$), 26.1 ($C(\underline{C}H_3)_3$), 36.4 ($\underline{C}(CH_3)_3$), 37.7 (C-3), 56.6, 57.3, 59.1 (C-2, C-5, NHCH), 69.0 (C-4), 119.9 (CN), 126.0, 129.5, 129.9, 130.1, 132.4, 132.5 (C-2′, C-3′, C-4′, C-5′, C-5″, C-6′), 139.0 (C-1′), 150.7 (C-4″), 153.4 (C-2″), 164.3, 168.7, 172.2 (CO); the signal for $NHCH_2$ is missing (overlapping solvent peak).

*(2S,4R)-1-((S)-2-(1-Cyanocyclopropane-1-carboxamido)-3,3-dimethylbutanoyl)-N-(2,3-dimethyl-4-(4-methylthiazol-5-yl)benzyl)-4-hydroxypyrrolidine-2-carboxamide (**18**).*

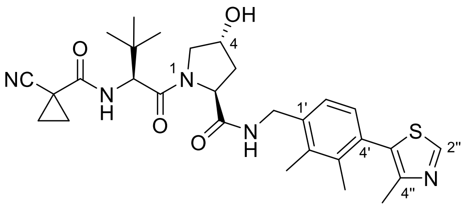

^{1}H NMR (500 MHz, DMSO-d_6) δ 0.95 (s, 9H, C(CH_3)$_3$), 1.43 – 1.54 (m, 2H, CH_2CCN), 1.58 – 1.66 (m, 2H, CH_2CCN), 1.87 – 1.94 (m, 1H, 3-H), 2.03 – 2.09 (m, 4H, 3-H, CH_3), 2.15 (s, 3H, CH_3), 2.20 (s, 3H, CH_3), 3.56 (d, *J* = 10.8 Hz, 1H, 5-H), 3.64 (dd, *J* = 10.8, 3.9 Hz, 1H, 5-H), 4.28 (dd, *J* = 15.4, 5.5 Hz, 1H), 4.31 – 4.38 (m, 2H), 4.50 (dd, *J* = 16.1, 8.4 Hz, 2H) (2-H, 4-H, NHC$\underline{H}$, NHC$\underline{H}_2$), 5.12 (d, *J* = 3.6 Hz, 1H, OH), 7.02 (d, *J* = 7.9 Hz, 1H), 7.24 (d, *J* = 7.9 Hz, 1H), 7.32 (d, *J* = 8.8 Hz, 1H) (Ar-H, CONH), 8.43 (t, *J* = 5.8 Hz, 1H, CONH), 9.03 (s, 1H, 2″-H); ^{13}C NMR (126 MHz, DMSO-d_6) δ 13.6 ($CH_2\underline{C}$CN), 14.9 (CH_3), 15.1 (CH_3), 16.5, 16.7 ($\underline{C}H_2$CCN), 16.8 (CH_3), 26.0 (C($\underline{C}H_3$)$_3$), 36.2 ($\underline{C}$(CH_3)$_3$), 37.9 (C-3), 41.1 (NHCH_2), 56.6, 57.3, 58.7 (C-2, C-5, NHCH), 68.8 (C-4), 120.0 (CN), 125.3, 128.0, 129.1, 130.7, 135.2, 135.78 (C-2′, C-3′, C-4′, C-5′, C-5″, C-6′), 137.4 (C-1′), 148.9 (C-4″), 152.0 (C-2″), 164.3, 168.6, 171.2 (CO).

*(2S,4R)-1-((S)-2-(1-Cyanocyclopropane-1-carboxamido)-3,3-dimethylbutanoyl)-N-(2,3-difluoro-4-(4-methylthiazol-5-yl)benzyl)-4-hydroxypyrrolidine-2-carboxamide (**19**).*

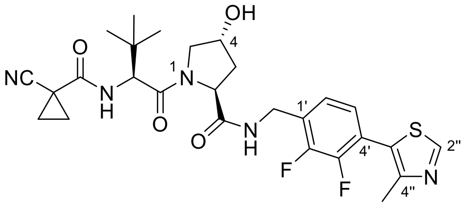

^{1}H NMR (600 MHz, DMSO-d_6 δ 0.93 (s, 9H, C(CH_3)$_3$), 1.45 – 1.54 (m, 2H, CH_2CCN), 1.58 – 1.66 (m, 2H, CH_2CCN), 1.86 – 1.93 (m, 1H, 3-H), 2.04 – 2.10 (m, 1H, 3-H), 2.35 (s, 3H, CH_3), 3.54 – 3.58 (m, 1H, 5-H), 3.63 (dd, *J* = 10.8, 3.9 Hz, 1H, 5-H), 4.30 – 4.39 (m, 2H), 4.42 (dd, *J* = 15.9, 6.1 Hz, 1H), 4.45 – 4.54 (m, 2H) (2-H, 4-H, NHC$\underline{H}$, NHC$\underline{H}_2$), 5.15 (d, *J* = 3.6 Hz, 1H, OH), 7.17 – 7.25 (m, 1H), 7.30 – 7.39 (m, 2H) (Ar-H, CONH), 8.70 (t, *J* = 6.0 Hz, 1H, CONH), 9.14 (s, 1H, 2″-H); ^{13}C NMR (151 MHz, DMSO-d_6) δ 13.7 ($CH_2\underline{C}$CN), 15.7 (CH_3), 16.6, 16.8 ($\underline{C}H_2$CCN), 26.0 (C($\underline{C}H_3$)$_3$), 35.9 (NHCH_2), 36.1 ($\underline{C}$(CH_3)$_3$), 37.7 (C-3), 56.6, 57.3, 58.7 (C-2, C-5, NHCH), 68.85 (C-4), 119.4 (d, $^{2}J_{F,C}$ = 11.7 Hz, C-4′), 120.1 (CN), 122.6 (d, $^{3}J_{F,C}$ = 2.3 Hz, C-5″), 124.1 (d, $^{3}J_{F,C}$ = 3.6 Hz), 126.0 (d, $^{3}J_{F,C}$ = 2.9 Hz, C-5′, C-6′), 129.0 (d, $^{2}J_{F,C}$ = 11.8 Hz, C-1′), 146.5 (dd, $^{1}J_{F,C}$ = 189.9 Hz, $^{2}J_{F,C}$ = 13.2 Hz, C-2′), 148.1 (dd, $^{1}J_{F,C}$ = 189.2 Hz, $^{2}J_{F,C}$ = 13.3 Hz, C-3′), 150.7 (C-4″), 153.8 (C-2″), 164.4, 168.7, 171.9 (CO).

*(2S,4R)-1-((S)-2-(1-Cyanocyclopropane-1-carboxamido)-3,3-dimethylbutanoyl)-N-(3-fluoro-2-hydroxy-4-(4-methylthiazol-5-yl)benzyl)-4-hydroxypyrrolidine-2-carboxamide (**20**).*

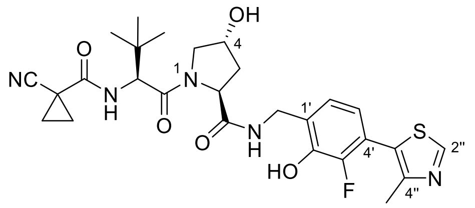

^{1}H NMR (600 MHz, DMSO-d_6) δ 0.93 (s, 9H, C(CH_3)$_3$), 1.45 – 1.53 (m, 2H, CH_2CCN), 1.57 – 1.65 (m, 2H, CH_2CCN), 1.86 – 1.94 (m, 1H, 3-H), 2.02 – 2.09 (m, 1H, 3-H), 2.32 (s, 3H, CH_3), 3.52 – 3.58 (m, 1H, 5-H), 3.62 (dd, *J* = 10.8, 3.9 Hz, 1H, 5-H), 4.20 – 4.29 (m, 2H), 4.31 – 4.36 (m, 1H), 4.45 – 4.53 (m, 2H) (2-H, 4-H, NHC<u>H</u>, NHC<u>H</u>$_2$), 5.14 (d, *J* = 3.7 Hz, 1H, OH), 6.78 (dd, *J* = 7.9, 6.6 Hz, 1H), 7.14 (d, *J* = 8.0 Hz, 1H), 7.32 (d, *J* = 8.9 Hz, 1H) (Ar-H, CONH), 8.64 (t, *J* = 6.1 Hz, 1H, CONH), 9.07 (s, 1H, 2″-H), 9.95 (s, 1H, Ar-OH); ^{13}C NMR (151 MHz, DMSO-d_6) δ 13.7 (CH_2<u>C</u>CN), 15.8 (CH_3), 16.6, 16.8 (<u>C</u>H$_2$CCN), 26.0 (C(<u>C</u>H$_3$)$_3$), 36.2 (<u>C</u>(CH_3)$_3$), 37.4, 37.8 (C-3, NHCH$_2$), 56.6, 57.3, 58.7 (C-2, C-5, NHCH), 68.9 (C-4), 117.8 (d, $^2J_{F,C}$ = 13.2 Hz, C-4′), 120.1 (CN), 120.7 (C-5″), 123.3 (d, $^3J_{F,C}$ = 2.6 Hz, C-5′), 124.2 (C-6′), 129.26 (d, $^3J_{F,C}$ = 1.7 Hz, C-1′), 142.6 (d, $^2J_{F,C}$ = 14.4 Hz, C-2′), 148.3 (d, $^1J_{F,C}$ = 241.3 Hz, C-3′), 149.9 (C-4″), 152.94 (C-2″), 164.4, 168.7, 172.2 (CO).

*(2S,4R)-1-((S)-2-(1-Cyanocyclopropane-1-carboxamido)-3,3-dimethylbutanoyl)-N-(3-fluoro-2-methoxy-4-(4-methylthiazol-5-yl)benzyl)-4-hydroxypyrrolidine-2-carboxamide (**21**).*

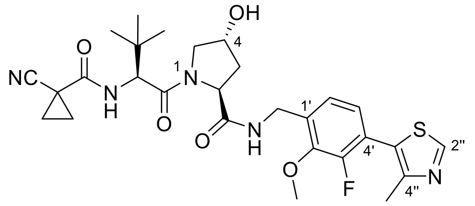

^{1}H NMR (600 MHz, DMSO-d_6) δ 0.94 (s, 9H, C(CH_3)$_3$), 1.46 – 1.54 (m, 2H, CH_2CCN), 1.58 – 1.65 (m, 2H, CH_2CCN), 1.87 – 1.94 (m, 1H, 3-H), 2.04 – 2.10 (m, 1H, 3-H), 2.34 (s, 3H, CH_3), 3.55 – 3.59 (m, 1H, 5-H), 3.63 (dd, *J* = 10.8, 3.9 Hz, 1H, 5-H), 3.90 (s, 3H, OCH_3), 4.27 – 4.40 (m, 3H), 4.46 – 4.54 (m, 2H) (2-H, 4-H, NHC<u>H</u>, NHC<u>H</u>$_2$), 5.14 (d, *J* = 3.7 Hz, 1H, OH), 7.07 – 7.11 (m, 1H), 7.27 (d, *J* = 8.1 Hz, 1H), 7.34 (d, *J* = 8.9 Hz, 1H) (Ar-H, CONH), 8.57 (t, *J* = 6.0 Hz, 1H, CONH), 9.11 (s, 1H, 2″-H); ^{13}C NMR (151 MHz, DMSO-d_6) δ 13.7 (CH_2<u>C</u>CN), 15.7 (CH_3), 16.6, 16.8 (<u>C</u>H$_2$CCN), 26.0 (C(<u>C</u>H$_3$)$_3$), 36.2 (<u>C</u>(CH_3)$_3$), 36.9, 37.8 (C-3, NHCH$_2$), 56.6, 57.3, 58.8 (C-2, C-5, NHCH), 61.3 (d, $^4J_{F,C}$ = 4.8 Hz, OCH_3), 68.9 (C-4), 118.7 (C-5″), 120.1 (CN), 123.6 (d, $^2J_{F,C}$ = 10.2 Hz, C-4′), 123.6, 125.5, 134.5 (C-1′, C-5′, C-6′), 145.1 (d, $^2J_{F,C}$ = 11.4 Hz, C-2′), 150.3 (C-4″), 151.8 (d, $^1J_{F,C}$ = 248.1 Hz, C-3′) 153.3 (C-2″), 164.4, 168.7, 171.7 (CO).

*(2S,4R)-1-((S)-2-(1-Cyanocyclopropane-1-carboxamido)-3,3-dimethylbutanoyl)-4-hydroxy-N-((4-(4-methylthiazol-5-yl)naphthalen-1-yl)methyl)pyrrolidine-2-carboxamide (**22**).*

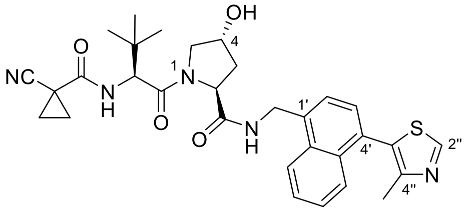

^{1}H NMR (500 MHz, DMSO-d_6) δ 0.96 (s, 9H, $C(CH_3)_3$), 1.45 – 1.56 (m, 2H, CH_2CCN), 1.57 – 1.66 (m, 2H, CH_2CCN), 1.91 – 2.00 (m, 1H, 3-H), 2.03 – 2.10 (m, 1H, 3-H), 2.17 (s, 3H, CH_3), 3.55 – 3.60 (m, 1H, 5-H), 3.66 (dd, *J* = 10.8, 4.0 Hz, 1H, 5-H), 4.34 – 4.41 (m, 1H), 4.48 – 4.58 (m, 2H), 4.76 – 4.85 (m, 2H) (2-H, 4-H, NHCH, $NHCH_2$), 5.13 (d, *J* = 3.7 Hz, 1H, OH), 7.33 (d, *J* = 8.9 Hz, 1H), 7.46 (d, *J* = 7.2 Hz, 1H), 7.54 – 7.69 (m, 4H), 8.13 – 8.19 (m, 1H) (Ar-H, CONH), 8.68 (t, *J* = 5.8 Hz, 1H, CONH), 9.16 (s, 1H, 2″-H); ^{13}C NMR (126 MHz, DMSO-d_6) δ 13.7 (CH_2CCN), 15.4 (CH_3), 16.6, 16.8 (CH_2CCN), 26.1 ($C(CH_3)_3$), 36.2 ($C(CH_3)_3$), 37.9 (C-3), 56.6, 57.3, 58.8 (C-2, C-5, NHCH), 68.9 (C-4), 120.0 (CN), 124.1, 124.5, 125.5, 126.4, 126.7, 127.7, 128.4, 128.7, 131.0, 131.8, 135.9 (C-Ar, C-5″), 150.0 (C-4″), 152.8 (C-2″), 164.4, 168.6, 171.5 (CO); the signal for $NHCH_2$ is missing (overlapping solvent peak).

*(2S,4R)-1-((S)-2-(1-Cyanocyclopropane-1-carboxamido)-3,3-dimethylbutanoyl)-4-hydroxy-N-((5-(4-methylthiazol-5-yl)quinolin-8-yl)methyl)pyrrolidine-2-carboxamide (**23**).*

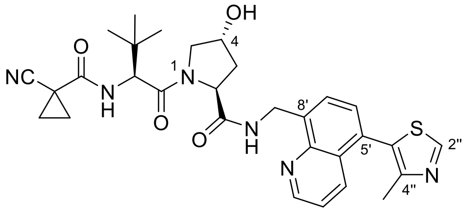

^{1}H NMR (500 MHz, DMSO-d_6) δ 0.93 (s, 9H, $C(CH_3)_3$), 1.45 – 1.56 (m, 2H, CH_2CCN), 1.58 – 1.66 (m, 2H, CH_2CCN), 1.95 – 2.01 (m, 1H, 3-H), 2.07 – 2.15 (m, 1H, 3-H), 2.18 (s, 3H, CH_3), 3.58 (d, *J* = 10.8 Hz, 1H, 5-H), 3.65 (dd, *J* = 10.9, 3.9 Hz, 1H, 5-H), 4.34 – 4.39 (m, 1H), 4.53 (d, *J* = 8.9 Hz, 1H), 4.59 (t, *J* = 8.2 Hz, 1H), 4.90 – 5.03 (m, 2H) (2-H, 4-H, NHCH, $NHCH_2$), 5.15 (d, *J* = 3.6 Hz, 1H, OH), 7.36 (d, *J* = 8.9 Hz, 1H), 7.54 (d, *J* = 7.4 Hz, 1H), 7.61 (dd, *J* = 8.5, 4.1 Hz, 1H), 7.88 (d, *J* = 7.4 Hz, 1H), 8.03 (dd, *J* = 8.6, 1.7 Hz, 1H), 8.68 (t, *J* = 6.1 Hz, 1H) (Ar-H, CONH), 9.00 (dd, *J* = 4.2, 1.7 Hz, 1H, CONH), 9.19 (s, 1H, 2″-H); ^{13}C NMR (126 MHz, DMSO-d_6) δ 13.7 (CH_2CCN), 15.5 (CH_3), 16.6, 16.7 (CH_2CCN), 26.0 ($C(CH_3)_3$), 36.2 ($C(CH_3)_3$), 37.8 (C-3), 56.6, 57.3, 58.9 (C-2, C-5, NHCH), 68.9 (C-4), 120.1 (CN), 122.1, 126.2, 126.5, 127.1, 127.3, 129.2, 133.7, 137.9 (C-Ar, C-5″), 145.4, 149.9 (C-2′, C-8a′), 150.3 (C-4″), 153.2 (C-2″), 164.4, 168.8, 171.9 (CO); the signal for $NHCH_2$ is missing (overlapping solvent peak).

*(2S,4R)-1-((S)-2-(1-Cyanocyclopropane-1-carboxamido)-3,3-dimethylbutanoyl)-4-hydroxy-N-((S)-1-(2-methyl-4-(4-methylthiazol-5-yl)phenyl)ethyl)pyrrolidine-2-carboxamide (**24**).*

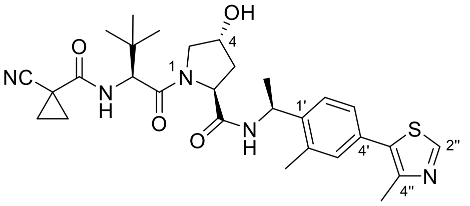

^{1}H NMR (600 MHz, DMSO-d_6) δ 0.95 (s, 9H, $C(CH_3)_3$), 1.35 (d, J = 6.9 Hz, 3H, $CHC\underline{H}_3$), 1.47 – 1.53 (m, 2H, CH_2CCN), 1.59 – 1.67 (m, 2H, CH_2CCN), 1.70 – 1.77 (m, 1H), 2.01 – 2.06 (m, 1H, 3-H), 2.33 (s, 3H, CH_3), 2.45 (s, 3H, Ar-CH_3), 3.49 – 3.59 (m, 2H, 5-H), 4.24 – 4.28 (m, 1H), 4.46 (t, J = 8.3 Hz, 1H), 4.50 (d, J = 8.9 Hz, 1H) (2-H, 4-H, $NHC\underline{H}$), 5.02 – 5.09 (m, 1H, $C\underline{H}CH_3$), 5.11 (d, J = 3.6 Hz, 1H, OH), 7.23 – 7.26 (m, 1H), 7.28 – 7.32 (m, 2H), 7.39 (d, J = 8.0 Hz, 1H) (Ar-H, CONH), 8.43 (d, J = 7.7 Hz, 1H, CONH), 8.97 (s, 1H, 2″-H); ^{13}C NMR (151 MHz, DMSO-d_6) δ 13.6 ($CH_2\underline{C}CN$), 16.0 (CH_3), 16.6, 16.8 ($\underline{C}H_2CCN$), 18.5 (CH_3), 21.0 ($CH\underline{C}H_3$), 26.1 ($C(\underline{C}H_3)_3$), 36.2 ($\underline{C}(CH_3)_3$), 37.6 (C-3), 44.4 ($\underline{C}HCH_3$), 56.6, 57.3, 58.6 (C-2, C-5, NHCH), 68.7 (C-4), 125.3, 126.6, 129.6, 130.5, 131.1 (C-3′, C-4′, C-5′, C-6′, C-5″), 135.5 (C-2′), 142.6 (C-1′), 147.6 (C-4″), 151.3 (C-2″), 164.3, 168.5, 170.1 (CO).

*(2S,4R)-1-((S)-2-(1-Cyanocyclopropane-1-carboxamido)-3,3-dimethylbutanoyl)-N-((S)-1-(3-fluoro-4-(4-methylthiazol-5-yl)phenyl)ethyl)-4-hydroxypyrrolidine-2-carboxamide (**25**).*

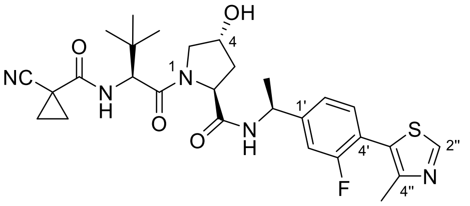

^{1}H NMR (600 MHz, DMSO-d_6) δ 0.95 (s, 9H, $C(CH_3)_3$), 1.39 (d, J = 7.0 Hz, 3H, $CHC\underline{H}_3$), 1.47 – 1.53 (m, 2H, CH_2CCN), 1.59 – 1.67 (m, 2H, CH_2CCN), 1.75 – 1.81 (m, 1H, 3-H), 2.06 – 2.11 (m, 1H, 3-H), 2.33 (s, 3H, CH_3), 3.51 – 3.61 (m, 2H, 5-H), 4.27 – 4.32 (m, 1H), 4.47 (dd, J = 9.0, 7.7 Hz, 1H), 4.51 (d, J = 8.9 Hz, 1H) (2-H, 4-H, $NHC\underline{H}$), 4.89 – 4.97 (m, 1H, $C\underline{H}CH_3$), 5.13 (d, J = 3.6 Hz, 1H, OH), 7.20 – 7.32 (m, 3H), 7.44 (t, J = 7.8 Hz, 1H) (Ar-H, CONH), 8.50 (d, J = 7.6 Hz, 1H, CONH), 9.10 (s, 1H, 2″-H); ^{13}C NMR (151 MHz, DMSO-d_6) δ 13.7 ($CH_2\underline{C}CN$), 15.7 (d, $^{5}J_{F,C}$ = 1.9 Hz, CH_3), 16.6, 16.8 ($\underline{C}H_2CCN$), 22.3 ($CH\underline{C}H_3$), 26.1 ($C(\underline{C}H_3)_3$), 36.2 ($\underline{C}(CH_3)_3$), 37.7 (C-3), 47.6 ($\underline{C}HCH_3$), 56.6, 57.3, 58.7 (C-2, C-5, NHCH), 68.8 (C-4), 113.3 (d, $^{2}J_{F,C}$ = 22.9 Hz, C-2′), 116.9 (d, $^{2}J_{F,C}$ = 15.3 Hz, C-4′), 120.1 (CN), 122.1 (d, $^{3}J_{F,C}$ = 2.8 Hz, C-5″), 123.8 (C-6′), 131.9 (d, $^{2}J_{F,C}$ = 2.5 Hz, C-5′), 148.5 (d, $^{3}J_{F,C}$ = 7.3 Hz, C-1′), 150.1 (C-4″), 153.1 (C-2″), 158.8 (d, $^{1}J_{F,C}$ = 246.4 Hz, C-3′), 164.3, 168.6, 170.5 (CO).

*(2S,4R)-1-((S)-2-(1-Fluorocyclopropane-1-carboxamido)-3,3-dimethylbutanoyl)-4-hydroxy-N-(2-methyl-4-(4-methylthiazol-5-yl)benzyl)pyrrolidine-2-carboxamide (**26**).*

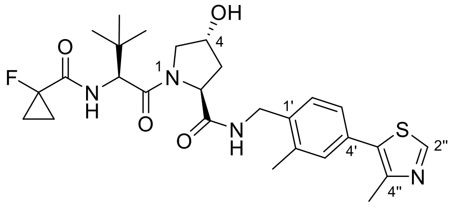

^{1}H NMR (500 MHz, DMSO-d_6) δ 0.97 (s, 9H, $(CH_3)_3$), 1.17 – 1.26 (m, 2H, CH_2CF), 1.29 – 1.43 (m, 2H, CH_2CF), 1.87 – 1.96 (m, 1H, 3-H), 2.04 – 2.12 (m, 1H, 3-H), 2.30 (s, 3H, CH_3), 2.45 (s, 3H, Ar-CH_3), 3.60 (d, J = 10.8 Hz, 1H, 5-H), 3.66 (dd, J = 10.7, 3.9 Hz, 1H, 5-H), 4.21 (dd, J = 15.6, 5.3 Hz, 1H), 4.31 – 4.38 (m, 2H), 4.50 (t, J = 8.2 Hz, 1H), 4.56 – 4.62 (m, 1H) (2-H, 4-H, NHC$\underline{H}$, NHC$\underline{H}_2$), 5.13 (d, J = 3.7 Hz, 1H, OH), 7.20 – 7.30 (m, 3H), 7.41 (d, J = 7.9 Hz, 1H) (Ar-H, CONH), 8.47 (t, J = 5.7 Hz, 1H, CONH), 8.97 (s, 1H, 2″-H); ^{13}C NMR (126 MHz, DMSO-d_6) δ 12.6 (d, $^2J_{F,C}$ = 10.5 Hz, CH_2CF), 12.9 (d, $^2J_{F,C}$ = 10.1 Hz, CH_2CF), 15.9 (CH_3), 18.4 (Ar-CH_3), 26.1 (C($\underline{C}H_3)_3$), 36.0 ($\underline{C}(CH_3)_3$), 37.9 (C-3), 54.8 (NHCH_2), 56.5, 56.6, 58.7 (C-2, C-5, NHCH), 68.9 (C-4), 78.1 (d, $^1J_{F,C}$ = 232.6 Hz, CF), 126.1, 127.9, 129.0, 130.2, 131.1 (C-3′, C-4′, C-5′, C-5″, C-6′), 136.3, 136.9 (C-1′, C-2′), 147.63 (C-4″), 151.3 (C-2″), 168.0 (d, $^2J_{F,C}$ = 20.1 Hz), 168.8 , 171.4 (CO).

*(2S,4R)-N-(3-Fluoro-4-(4-methylthiazol-5-yl)benzyl)-1-((S)-2-(1-fluorocyclopropane-1-carboxamido)-3,3-dimethylbutanoyl)-4-hydroxypyrrolidine-2-carboxamide (**27**).*

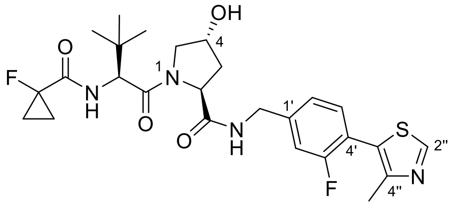

^{1}H NMR (600 MHz, DMSO-d_6) δ 0.96 (s, 9H, C$(CH_3)_3$), 1.17 – 1.25 (m, 2H, CH_2CF), 1.31 – 1.41 (m, 2H, CH_2CF), 1.88 – 1.94 (m, 1H, 3-H), 2.05 – 2.12 (m, 1H, 3-H), 2.32 (s, 3H, CH_3), 3.58 – 3.63 (m, 1H, 5-H), 3.67 (dd, J = 10.8, 3.9 Hz, 1H, 5-H), 4.23 (dd, J = 16.1, 5.5 Hz, 1H), 4.33 – 4.38 (m, 1H), 4.44 – 4.51 (m, 2H), 4.55 – 4.61 (m, 1H) (2-H, 4-H, NHC$\underline{H}$, NHC$\underline{H}_2$), 5.16 (d, J = 3.6 Hz, 1H, OH), 7.19 – 7.28 (m, 2H), 7.33 (dd, J = 11.4, 1.6 Hz, 1H), 7.40 (t, J = 7.8 Hz, 1H) (Ar-H, CONH), 8.69 (t, J = 6.1 Hz, 1H, CONH), 9.09 (s, 1H, 2″-H); ^{13}C NMR (151 MHz, DMSO-d_6) δ 12.7 (d, $^2J_{F,C}$ = 10.1 Hz, CH_2CF), 12.9 (d, $^2J_{F,C}$ = 10.2 Hz, CH_2CF), 15.7 (CH_3), 26.1 (C($\underline{C}H_3)_3$), 36.0 ($\underline{C}(CH_3)_3$), 37.8 (C-3), 41.4 (NHCH_2), 56.5, 56.6, 58.8 (C-2, C-5, NHCH), 68.9 (C-4), 78.1 (d, $^1J_{F,C}$ = 231.9 Hz, CF), 114.4 (d, $^2J_{F,C}$ = 23.0 Hz, C-2′), 116.8 (d, $^2J_{F,C}$ = 15.4 Hz, C-4′), 123.1 (d, $^3J_{F,C}$ = 3.3 Hz, C-5″), 123.8 (C-6′), 131.7 (d, $^3J_{F,C}$ = 2.8 Hz, C-5′), 143.2 (d, $^3J_{F,C}$ = 7.6 Hz, C-1′), 150.1 (C-4″), 153.1 (C-2″), 158.9 (d, $^2J_{F,C}$ = 246.3 Hz, C-3′), 168.0 (d, $^2J_{F,C}$ = 21.0 Hz), 168.9, 171.9 (CO).

*(2S,4R)-N-(5-Fluoro-2-methyl-4-(4-methylthiazol-5-yl)benzyl)-1-((S)-2-(1-fluorocyclopropane-1-carboxamido)-3,3-dimethylbutanoyl)-4-hydroxypyrrolidine-2-carboxamide (**28**).*

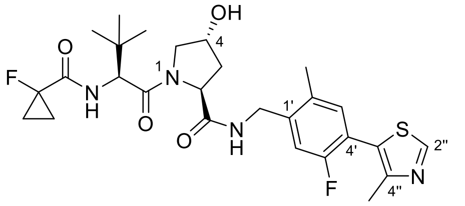

^{1}H NMR (500 MHz, DMSO-d_6) δ 0.95 (s, 9H, $C(CH_3)_3$), 1.18 – 1.28 (m, 2H, CH_2CF), 1.30 – 1.42 (m, 2H, CH_2CF), 1.87 – 1.96 (m, 1H, 3-H), 2.05 – 2.12 (m, 1H, 3-H), 2.26 (s, 3H, CH_3), 2.32 (d, J = 1.2 Hz, 3H, CH_3), 3.61 (d, J = 10.8 Hz, 1H, 5-H), 3.67 (dd, J = 10.8, 3.8 Hz, 1H, 5-H), 4.14 (dd, J = 16.2, 5.2 Hz, 1H), 4.33 – 4.42 (m, 2H), 4.51 (t, J = 8.2 Hz, 1H), 4.56 – 4.62 (m, 1H) (2-H, 4-H, NHC$\underline{H}$, NHC$\underline{H}_2$), 5.16 (d, J = 3.6 Hz, 1H, OH), 7.21 – 7.28 (m, 2H), 7.35 (d, J = 11.3 Hz, 1H) (Ar-H, CONH), 8.62 (t, J = 5.9 Hz, 1H, CONH), 9.08 (s, 1H, 2″-H); ^{13}C NMR (126 MHz, DMSO-d_6) δ 12.6 (d, $^{2}J_{F,C}$ = 10.2 Hz, $\underline{C}H_2CF$), 12.9 (d, $^{2}J_{F,C}$ = 10.1 Hz, $\underline{C}H_2CF$), 15.6 (d, $^{5}J_{F,C}$ = 2.8 Hz, CH_3), 17.5 (Ar-CH_3), 26.1 ($C(\underline{C}H_3)_3$), 36.0 ($NHCH_2$), 37.8 (C-3), 56.5, 56.6, 58.8 (C-2, C-5, NHCH), 68.9 (C-4), 78.1 (d, $^{1}J_{F,C}$ = 232.3 Hz, CF), 114.4 (d, $^{2}J_{F,C}$ = 23.7 Hz, C-2′), 116.3 (d, $^{2}J_{F,C}$ = 15.2 Hz, C-4′), 123.9 (C-6′), 131.5 (d, $^{3}J_{F,C}$ = 3.4 Hz, C-5″), 132.7 (d, $^{3}J_{F,C}$ = 2.3 Hz, C-5′), 140.4 (d, $^{3}J_{F,C}$ = 7.2 Hz, C-1′), 149.9 (C-4″), 152.8 (C-2″), 157.4 (d, $^{1}J_{F,C}$ = 244.0 Hz, C-3′), 168.0 (d, $^{2}J_{F,C}$ = 20.2 Hz), 168.9, 171.8 (CO).

*(2S,4R)-N-(5-Fluoro-2-methoxy-4-(4-methylthiazol-5-yl)benzyl)-1-((S)-2-(1-fluorocyclopropane-1-carboxamido)-3,3-dimethylbutanoyl)-4-hydroxypyrrolidine-2-carboxamide (**29**).*

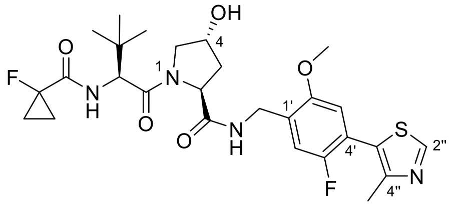

^{1}H NMR (600 MHz, DMSO-d_6) δ 0.94 (s, 9H, $C(CH_3)_3$), 1.17 – 1.27 (m, 2H, CH_2CF), 1.30 – 1.41 (m, 2H, CH_2CF), 1.87 – 1.96 (m, 1H, 3-H), 2.04 – 2.11 (m, 1H, 3-H), 2.33 (d, J = 1.2 Hz, 3H, CH_3), 3.60 (d, J = 10.8 Hz, 1H, 5-H), 3.65 (dd, J = 10.8, 3.8 Hz, 1H, 5-H), 4.11 (dd, J = 16.8, 5.3 Hz, 1H), 4.25 – 4.39 (m, 2H), 4.49 (t, J = 8.3 Hz, 1H), 4.58 (d, J = 9.2 Hz, 1H) (2-H, 4-H, NHC$\underline{H}$, NHC$\underline{H}_2$), 5.15 (d, J = 3.6 Hz, 1H, OH), 6.97 (d, J = 5.9 Hz, 1H), 7.24 (dd, J = 9.2, 2.8 Hz, 1H), 7.36 (d, J = 10.6 Hz, 1H) (Ar-H, CONH), 8.60 (t, J = 6.0 Hz, 1H, CONH), 9.08 (s, 1H, 2″-H); ^{13}C NMR (151 MHz, DMSO-d_6) δ 12.7 (d, $^{2}J_{F,C}$ = 10.4 Hz, $\underline{C}H_2CF$), 12.9 (d, $^{2}J_{F,C}$ = 10.1 Hz, $\underline{C}H_2CF$), 15.7 (CH_3), 26.1 ($C(\underline{C}H_3)_3$), 36.0 ($\underline{C}(CH_3)_3$), 37.1 (C-3), 37.8 ($NHCH_2$), 56.1, 56.5, 56.6, 58.9 (OCH_3, C-2, C-5, NHCH), 68.9 (C-4), 78.2 (d, $^{1}J_{F,C}$ = 232.3 Hz, CF), 112.9 (C-5″), 114.9 (d, $^{2}J_{F,C}$ = 25.7 Hz, C-2′), 116.7b(d, $^{2}J_{F,C}$ = 16.7 Hz, C-4′), 124.1 (C-5′), 130.0 (d, $^{3}J_{F,C}$ = 7.4 Hz, C-1′), 150.2 (C-4″), 152.3 (C-6′), 153.0 (C-2″), 153.3 (d, $^{1}J_{F,C}$ = 238.8 Hz, C-3′), 168.0 (d, $^{2}J_{F,C}$ = 20.6 Hz), 169.0, 172.1 (CO).

*(2S,4R)-1-((S)-2-(1-Fluorocyclopropane-1-carboxamido)-3,3-dimethylbutanoyl)-4-hydroxy-N-((S)-1-(2-methyl-4-(4-methylthiazol-5-yl)phenyl)ethyl)pyrrolidine-2-carboxamide (**30**).*

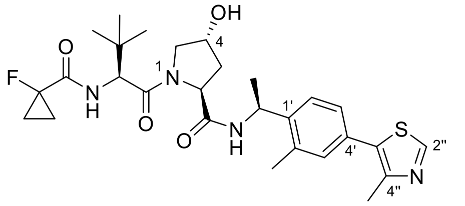

^{1}H NMR (500 MHz, DMSO-d_6) δ 0.97 (s, 9H, $C(CH_3)_3$), 1.19 – 1.28 (m, 2H, CH_2CF), 1.32 – 1.41 (m, 5H, CH_2CF, $CHC\underline{H}_3$), 1.71 – 1.78 (m, 1H, 3-H), 2.01 – 2.07 (m, 1H, 3-H), 2.33 (s, 3H, CH_3), 2.46 (s, 3H, CH_3), 3.53 – 3.61 (m, 2H, 5-H), 4.24 – 4.30 (m, 1H), 4.46 (t, J = 8.2 Hz, 1H), 4.55 – 4.60 (m, 1H) (2-H, 4-H, NHC$\underline{H}$), 5.03 – 5.09 (m, 1H, $C\underline{H}CH_3$), 5.10 (d, J = 3.6 Hz, 1H, OH), 7.21 – 7.26 (m, 2H), 7.31 (dd, J = 8.0, 2.0 Hz, 1H), 7.39 (d, J = 8.0 Hz, 1H) (Ar-H, CONH), 8.40 (d, J = 7.7 Hz, 1H, CONH), 8.97 (s, 1H, 2″-H); ^{13}C NMR (126 MHz, DMSO-d_6) δ 12.6 (d, $^{2}J_{F,C}$ = 10.4 Hz, $\underline{C}H_2CF$), 12.9 (d, $^{2}J_{F,C}$ = 10.4 Hz, $\underline{C}H_2CF$), 16.0 (CH_3), 18.5 (CH_3), 21.0 ($CH\underline{C}H_3$), 26.2 ($C(\underline{C}H_3)_3$), 36.0 ($\underline{C}(CH_3)_3$), 37.6 (C-3), 44.4 ($\underline{C}HCH_3$), 56.5, 56.5, 58.6 (C-2, C-5, NHCH), 68.7 (C-4), 78.1 (d, $^{1}J_{F,C}$ = 232.5 Hz, $CH_2\underline{C}F$), 125.3, 126.5, 129.6, 130.5, 131.1, 135.5 (C-2′, C-3′, C-4′, C-5′, C-5″, C-6′), 142.5 (C-1′), 147.6 (C-4″), 151.3 (C-2″), 167.9 (d, $^{2}J_{F,C}$ = 20.1 Hz), 168.7, 170.1 (CO).

*(2S,4R)-N-((S)-1-(3-Fluoro-4-(4-methylthiazol-5-yl)phenyl)ethyl)-1-((S)-2-(1-fluorocyclopropane-1-carboxamido)-3,3-dimethylbutanoyl)-4-hydroxypyrrolidine-2-carboxamide (**31**).*

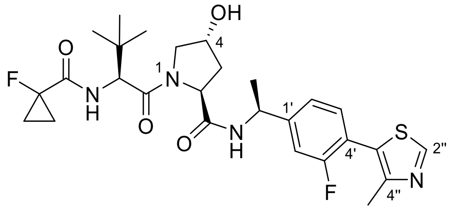

^{1}H NMR (500 MHz, DMSO-d_6) δ 0.97 (s, 9H, $C(CH_3)_3$), 1.19 – 1.24 (m, 2H, CH_2CF), 1.32 – 1.45 (m, 5H, CH_2CF, $CHC\underline{H}_3$), 1.75 – 1.82 (m, 1H, 3-H), 2.06 – 2.11 (m, 1H, 3-H), 2.34 (d, J = 1.1 Hz, 3H, CH_3), 3.54 – 3.63 (m, 2H, 5-H), 4.28 – 4.32 (m, 1H), 4.47 (t, J = 8.3 Hz, 1H), 4.58 (dd, J = 9.3, 1.3 Hz, 1H) (2-H, 4-H, NHC$\underline{H}$), 4.90 – 4.97 (m, 1H, $C\underline{H}CH_3$), 5.13 (d, J = 3.6 Hz, 1H, OH), 7.17 – 7.29 (m, 3H), 7.42 – 7.47 (m, 1H) (Ar-H, CONH), 8.47 (d, J = 7.6 Hz, 1H, CONH), 9.09 (s, 1H, 2″-H); ^{13}C NMR (126 MHz, DMSO-d_6) δ 12.6 (d, $^{2}J_{F,C}$ = 10.2 Hz, $\underline{C}H_2CF$), 12.9 (d, $^{2}J_{F,C}$ = 10.1 Hz, $\underline{C}H_2CF$), 15.7 (d, $^{5}J_{F,C}$ = 2.6 Hz, CH_3), 22.3 ($CH\underline{C}H_3$), 26.2 ($C(\underline{C}H_3)_3$), 36.0 ($\underline{C}(CH_3)_3$), 37.7 (C-3), 47.5 ($\underline{C}HCH_3$), 56.5, 56.6, 58.6 (C-2, C-5, NHCH), 68.8 (C-4), 78.1 (d, $^{1}J_{F,C}$ = 232.4 Hz, $CH_2\underline{C}F$), 113.3 (d, $^{2}J_{F,C}$ = 22.8 Hz, C-2′), 116.9 (d, $^{2}J_{F,C}$ = 15.4 Hz, C-4′), 122.1 (d, $^{3}J_{F,C}$ = 3.0 Hz, C-5″), 123.7 (C-6′), 131.9 (d, $^{3}J_{F,C}$ = 2.8 Hz, C-5′), 148.4 (d, $^{3}J_{F,C}$ = 7.1 Hz, C-1′), 150.1 (C-4″), 153.1 (C-2″), 158.8 (d, $^{1}J_{F,C}$ = 246.5 Hz, C-3′), 168.0 (d, $^{2}J_{F,C}$ = 20.2 Hz), 168.8, 170.5 (CO).

*(2S,4R)-N-((S)-1-(5-Fluoro-2-methyl-4-(4-methylthiazol-5-yl)phenyl)ethyl)-1-((S)-2-(1-fluorocyclopropane-1-carboxamido)-3,3-dimethylbutanoyl)-4-hydroxypyrrolidine-2-carboxamide (**32**).*

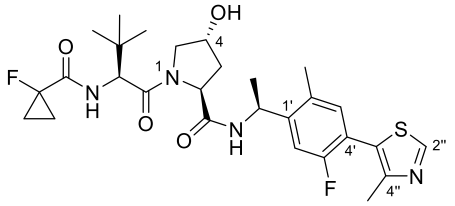

^{1}H NMR (600 MHz, DMSO-d_6) δ 0.96 (s, 9H, $C(CH_3)_3$), 1.19 – 1.23 (m, 2H, CH_2CF), 1.31 – 1.41 (m, 5H, CH_2CF, $CHC\underline{H}_3$), 1.70 – 1.77 (m, 1H, 3-H), 2.03 – 2.09 (m, 1H, 3-H), 2.30 (s, 3H, CH_3), 2.33 (s, 3H, CH_3), 3.54 – 3.61 (m, 2H, 5-H), 4.26 – 4.31 (m, 1H), 4.44 (t, J = 8.3 Hz, 1H), 4.57 (d, J = 9.3 Hz, 1H) (2-H, 4-H, $NHC\underline{H}$), 5.00 – 5.07 (m, 1H, $C\underline{H}CH_3$), 5.13 (d, J = 3.6 Hz, 1H, OH), 7.20 – 7.29 (m, 3H, Ar-H, CONH), 8.44 (d, J = 7.7 Hz, 1H, CONH), 9.09 (s, 1H, 2″-H); ^{13}C NMR (151 MHz, DMSO-d_6) δ 12.6 (d, $^{2}J_{F,C}$ = 10.1 Hz, $\underline{C}H_2CF$), 12.9 (d, $^{2}J_{F,C}$ = 10.4 Hz, $\underline{C}H_2CF$), 15.8 (d, $^{5}J_{F,C}$ = 3.1 Hz, CH_3), 17.6 (CH_3), 20.8 ($CH\underline{C}H_3$), 26.2 ($C(\underline{C}H_3)_3$), 36.0 ($\underline{C}(CH_3)_3$), 37.6 (C-3), 44.6 ($\underline{C}HCH_3$), 56.5, 56.6, 58.7 (C-2, C-5, NHCH), 68.8 (C-4), 78.1 (d, $^{1}J_{F,C}$ = 232.4 Hz, $CH_2\underline{C}F$), 112.4 (d, $^{2}J_{F,C}$ = 23.0 Hz, C-2′), 116.6 (d, $^{2}J_{F,C}$ = 15.2 Hz, C-4′), 123.8 (C-5″), 131.2 (d, $^{4}J_{F,C}$ = 3.2 Hz, C-5′), 133.2 (C-6′), 146.0 (d, $^{3}J_{F,C}$ = 6.5 Hz, C-1′), 150.0 (C-4″), 153.0 (C-2″), 157.5 (d, $^{1}J_{F,C}$ = 244.1 Hz, C-3′), 168.0 (d, $^{2}J_{F,C}$ = 19.9 Hz), 168.8, 170.3 (CO).

*(2S,4R)-N-((S)-1-(5-Fluoro-2-methoxy-4-(4-methylthiazol-5-yl)phenyl)ethyl)-1-((S)-2-(1-fluorocyclopropane-1-carboxamido)-3,3-dimethylbutanoyl)-4-hydroxypyrrolidine-2-carboxamide (**33**).*

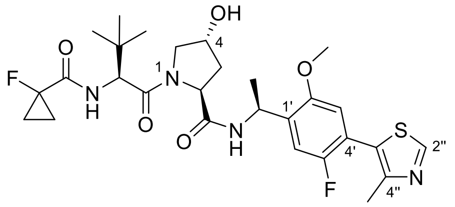

^{1}H NMR (600 MHz, DMSO-d_6) δ 0.97 (s, 9H, $C(CH_3)_3$), 1.19 – 1.25 (m, 2H, CH_2CF), 1.28 – 1.40 (m, 5H, CH_2CF, $CHC\underline{H}_3$), 1.74 – 1.81 (m, 1H, 3-H), 2.07 – 2.13 (m, 1H, 3-H), 2.36 (s, 3H, CH_3), 3.54 – 3.62 (m, 2H, 5-H), 3.83 (s, 3H, OCH_3), 4.29 – 4.32 (m, 1H), 4.48 (t, J = 8.3 Hz, 1H), 4.58 (d, J = 9.2 Hz, 1H) (2-H, 4-H, $NHC\underline{H}$), 5.11 – 5.18 (m, 2H, OH, $C\underline{H}CH_3$), 7.01 (d, J = 6.1 Hz, 1H), 7.17 (d, J = 10.6 Hz, 1H), 7.25 (dd, J = 9.3, 2.9 Hz, 1H) (Ar-H, CONH), 8.42 (d, J = 7.9 Hz, 1H, CONH), 9.10 (s, 1H, 2″-H); ^{13}C NMR (151 MHz, DMSO-d_6) δ 12.6 (d, $^{2}J_{F,C}$ = 10.4 Hz, $\underline{C}H_2CF$), 12.9 (d, $^{2}J_{F,C}$ = 10.2 Hz, $\underline{C}H_2CF$), 15.8 (d, $^{5}J_{F,C}$ = 1.9 Hz, CH_3), 21.1 ($CH\underline{C}H_3$), 26.2 ($C(\underline{C}H_3)_3$), 36.1 ($\underline{C}(CH_3)_3$), 37.6 (C-3), 42.7 ($\underline{C}HCH_3$), 56.2, 56.5, 56.6, 58.6 (OCH_3, C-2, C-5, NHCH), 68.8 (C-4), 78.1 (d, $^{1}J_{F,C}$ = 232.6 Hz, $CH_2\underline{C}F$), 113.0 (d, $^{2}J_{F,C}$ = 25.1 Hz, C-2′), 113.6 (C-5″), 117.0 (d, $^{2}J_{F,C}$ = 16.5 Hz, C-4′), 123.9 (C-5′), 135.7 (d, $^{3}J_{F,C}$ = 6.5 Hz, C-1′), 150.3 (C-4″), 151.9 (C-6′), 153.1 (C-2″), 153.2 (d, $^{1}J_{F,C}$ = 238.9 Hz, C-3′), 168.0 (d, $^{2}J_{F,C}$ = 20.2 Hz), 168.8, 170.3 (CO).

*(2S,4R)-1-((S)-2-(1-Cyanocyclopropane-1-carboxamido)-3,3-dimethylbutanoyl)-4-hydroxy-N-((S)-5-(4-methylthiazol-5-yl)-2,3-dihydro-1H-inden-1-yl)pyrrolidine-2-carboxamide (**34**).*

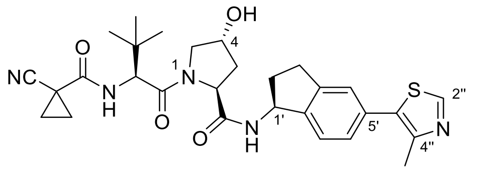

^{1}H NMR (500 MHz, DMSO-d_6) δ 0.97 (s, 9H, $C(CH_3)_3$), 1.45 – 1.53 (m, 2H, CH_2CCN), 1.57 – 1.65 (m, 2H, CH_2CCN), 1.85 – 1.92 (m, 1H, CH_2CH_2), 1.92 – 1.99 (m, 1H, CH_2CH_2), 2.01 – 2.10 (m, 1H, CH_2CH_2), 2.39 – 2.46 (m, 4H, CH_2CH_2, CH_3), 2.80 – 2.89 (m, 1H, 3-H), 2.93 – 3.02 (m, 1H, 3-H), 3.52 – 3.58 (m, 1H, 5-H), 3.65 (dd, *J* = 10.8, 3.9 Hz, 1H, 5-H), 4.31 – 4.37 (m, 1H), 4.38 – 4.45 (m, 1H), 4.52 (d, *J* = 8.9 Hz, 1H) (2-H, 4-H, NHC<u>H</u>), 5.11 (d, *J* = 3.7 Hz, 1H, OH), 5.23 – 5.30 (m, 1H, 1′-H), 7.19 – 7.25 (m, 1H), 7.26 – 7.31 (m, 2H), 7.34 – 7.36 (m, 1H) (Ar-H, CONH), 8.34 (d, *J* = 8.3 Hz, 1H, CONH), 8.96 (d, *J* = 1.8 Hz, 1H, 2″-H); ^{13}C NMR (126 MHz, DMSO-d_6) δ 13.6 (<u>C</u>CN), 15.9 (CH_3), 16.6, 16.8 (<u>C</u>H_2CCN), 26.1 (C(<u>C</u>H_3)$_3$), 29.6, 32.7 (CH_2CH_2), 36.2 (<u>C</u>(CH_3)$_3$), 38.1 (C-3), 53.4, 56.6, 57.3, 58.7 (<u>C</u>HCH_2CH_2, C-2, C-5, NHCH), 68.8 (C-4), 120.0 (CN), 124.2, 125.1, 127.3, 130.6, 131.4 (C-Ar), 143.8, 144.1 (C-2′, C-4′), 147.7 (C-4″), 151.3 (C-2″), 164.3, 168.6, 171.2 (CO).

*(2S,4R)-1-((S)-2-(1-Cyanocyclopropane-1-carboxamido)-3,3-dimethylbutanoyl)-N-((S)-6-fluoro-5-(4-methylthiazol-5-yl)-2,3-dihydro-1H-inden-1-yl)-4-hydroxypyrrolidine-2-carboxamide (**35**).*

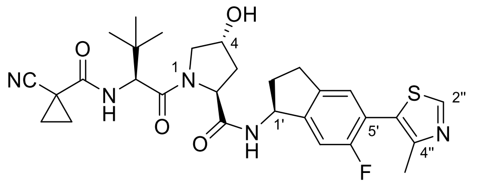

^{1}H NMR (600 MHz, DMSO-d_6) δ 0.97 (s, 9H, $C(CH_3)_3$), 1.47 – 1.53 (m, 2H, CH_2CCN), 1.58 – 1.66 (m, 2H, CH_2CCN), 1.91 – 2.00 (m, 2H, CH_2CH_2), 2.05 – 2.11 (m, 1H, CH_2CH_2), 2.32 (s, 3H, CH_3), 2.41 – 2.47 (m, 1H, CH_2CH_2), 2.79 – 2.86 (m, 1H, 3-H), 2.92 – 2.98 (m, 1H, 3-H), 3.53 – 3.58 (m, 1H, 5-H), 3.66 (dd, *J* = 10.8, 3.9 Hz, 1H, 5-H), 4.34 – 4.37 (m, 1H), 4.42 (dd, *J* = 8.9, 7.7 Hz, 1H), 4.52 (d, *J* = 8.9 Hz, 1H) (2-H, 4-H, NHC<u>H</u>), 5.14 (d, *J* = 3.6 Hz, 1H, OH), 5.23 – 5.28 (m, 1H, 1′-H), 7.06 (d, *J* = 9.7 Hz, 1H), 7.30 – 7.36 (m, 2H) (Ar-H, CONH), 8.43 (d, *J* = 8.0 Hz, 1H, CONH), 9.09 (s, 1H, 2″-H); ^{13}C NMR (151 MHz, DMSO-d_6) δ 13.7 (CH_2<u>C</u>CN), 15.7 (d, $^{5}J_{F,C}$ = 3.0 Hz, CH_3), 16.6, 16.8 (<u>C</u>H_2CCN), 26.1 (C(<u>C</u>H_3)$_3$), 29.0, 33.0 (CH_2CH_2), 36.2 (<u>C</u>(CH_3)$_3$), 38.0 (C-3), 53.8, 56.6, 57.3, 58.8 (C-1′, C-2, C-5, NHCH), 68.84 (C-4), 111.3 (d, $^{2}J_{F,C}$ = 23.3 Hz, C-6′), 117.7 (d, $^{2}J_{F,C}$ = 16.7 Hz, C-4′), 120.1 (CN), 124.3, 127.6 (C-2′, C-5″), 139.1 (C-4″), 147.2 (d, $^{3}J_{F,C}$ = 7.7 Hz, C-1′), 153.0 (C-2″), 158.1 (d, $^{1}J_{F,C}$ = 244.4 Hz, C-5′), 164.4, 168.6, 171.3 (CO).

*(2S,4R)-N-((S)-6-Fluoro-5-(4-methylthiazol-5-yl)-2,3-dihydro-1H-inden-1-yl)-1-((S)-2-(1-fluorocyclopropane-1-carboxamido)-3,3-dimethylbutanoyl)-4-hydroxypyrrolidine-2-carboxamide (**36**).*

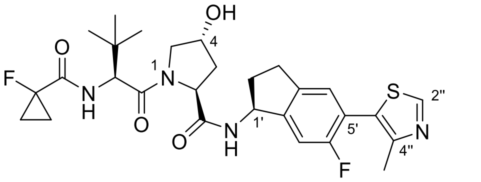

^{1}H NMR (600 MHz, DMSO-d_6) δ 0.98 (s, 9H, $C(CH_3)_3$), 1.16 – 1.25 (m, 2H, CH_2CF), 1.30 – 1.42 (m, 2H, CH_2CF), 1.89 – 2.00 (m, 2H, CH_2CF), 2.05 – 2.11 (m, 1H, CH_2CH_2), 2.32 (s, 3H, CH_3), 2.40 – 2.46 (m, 1H, CH_2CH_2), 2.77 – 2.86 (m, 1H, 3-H), 2.91 – 2.98 (m, 1H, 3-H), 3.56 – 3.61 (m, 1H, 5-H), 3.68 (dd, J = 10.8, 3.9 Hz, 1H, 5-H), 4.34 – 4.38 (m, 1H), 4.42 (t, J = 8.2 Hz, 1H), 4.57 – 4.61 (m, 1H) (2-H, 4-H, NHC$\underline{H}$), 5.14 (d, J = 3.6 Hz, 1H, OH), 5.22 – 5.29 (m, 1H, 1′-H), 7.04 – 7.09 (m, 1H), 7.26 (dd, J = 9.4, 2.8 Hz, 1H), 7.32 – 7.36 (m, 1H) (Ar-H, CONH), 8.41 (d, J = 8.0 Hz, 1H, CONH), 9.09 (s, 1H, 2″-H); ^{13}C NMR (151 MHz, DMSO-d_6) δ 12.6 (d, $^{2}J_{F,C}$ = 10.3 Hz, $\underline{C}H_2CF$), 12.9 (d, $^{2}J_{F,C}$ = 9.9 Hz, $\underline{C}H_2CF$), 15.7 (d, $^{5}J_{F,C}$ = 2.6 Hz, CH_3), 26.2 ($C(\underline{C}H_3)_3$), 29.0, 33.0 (CH_2CH_2), 36.0 ($\underline{C}(CH_3)_3$), 38.0 (C-3), 53.8, 56.5, 56.6, 58.8 (C-1′, C-2, C-5, NHCH), 68.8 (C-4), 78.1 (d, $^{1}J_{F,C}$ = 232.7 Hz, CH_2CF), 111.3 (d, $^{2}J_{F,C}$ = 23.2 Hz, C-6′), 117.7 (d, $^{2}J_{F,C}$ = 16.3 Hz, C-4′), 124.3, 127.6 (C-3′,C-5″), 139.1 (d, $^{4}J_{F,C}$ = 2.6 Hz, C-2′), 147.2 (d, $^{3}J_{F,C}$ = 7.6 Hz, C-1′), 150.1 (C-4″), 153.0 (C-2″), 158.1 (d, $^{1}J_{F,C}$ = 244.5 Hz, C-5′), 168.0 (d, $^{2}J_{F,C}$ = 20.2 Hz), 168.8, 171.3 (CO).

*(2S,4R)-N-((S)-7-Fluoro-6-(4-methylthiazol-5-yl)-1,2,3,4-tetrahydronaphthalen-1-yl)-1-((S)-2-(1-fluorocyclopropane-1-carboxamido)-3,3-dimethylbutanoyl)-4-hydroxypyrrolidine-2-carboxamide (**37**).*

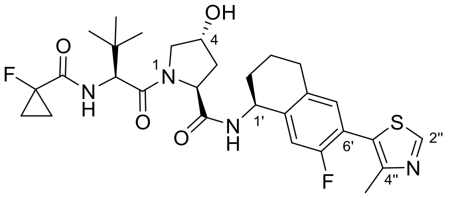

^{1}H NMR (600 MHz, DMSO-d_6) δ 0.98 (s, 9H, $C(CH_3)_3$), 1.13 – 1.28 (m, 4H), 1.30 – 1.41 (m, 2H), 1.68 – 1.78 (m, 2H), 1.87 – 1.94 (m, 2H), 1.95 – 2.00 (m, 1H), 2.04 – 2.11 (m, 1H) (CH_2CF, $CH_2CH_2CH_2$, 3-H), 2.32 (s, 3H, CH_3), 3.55 – 3.61 (m, 1H, 5-H), 3.69 (dd, J = 10.7, 4.0 Hz, 1H, 5-H), 4.34 – 4.39 (m, 1H), 4.43 (t, J = 8.2 Hz, 1H), 4.55 – 4.62 (m, 1H) (2-H, 4-H, N$\underline{H}$CH), 4.90 – 4.96 (m, 1H, 1′-H), 5.14 (d, J = 3.6 Hz, 1H, OH), 6.95 – 7.04 (m, 1H), 7.19 – 7.29 (m, 2H) (Ar-H, CONH), 8.41 (d, J = 8.5 Hz, 1H, CONH), 9.09 (s, 1H, 2″-H); ^{13}C NMR (151 MHz, DMSO-d_6) δ 12.7 (d, $^{2}J_{F,C}$ = 10.3 Hz, $\underline{C}H_2CF$), 12.9 (d, $^{2}J_{F,C}$ = 10.4 Hz, $\underline{C}H_2CF$), 15.7 (d, $^{5}J_{F,C}$ = 2.7 Hz), 26.2 ($C(\underline{C}H_3)_3$), 27.8, 29.1 (CH_2), 36.0 ($\underline{C}(CH_3)_3$), 37.9 (CH_2), 38.2 (C-3), 45.8, 46.6, 56.6 (d, J = 5.3 Hz), 58.9 (C-1′, C-2, C-5, NHCH), 68.8 (C-4), 78.1 (d, $^{1}J_{F,C}$ = 232.3 Hz, $CH_2\underline{C}F$), 114.6 (d, $^{2}J_{F,C}$ = 22.0 Hz, C-6′), 117.3 (d, $^{2}J_{F,C}$ = 15.5 Hz, C-4′), 123.8 (C-5″), 132.0 (C-3′), 133.6 (d, $^{3}J_{F,C}$ = 3.2 Hz, C-1′), 140.6 (C-2′), 150.1 (C-4″), 153.1 (C-2″), 157.1 (d, $^{1}J_{F,C}$ = 244.7 Hz, C-5′), 168.0 (d, $^{2}J_{F,C}$ = 20.1 Hz), 168.8 , 171.0 (CO).

4-Bromo-5-fluoro-2-methoxybenzaldehyde (type ***40****, R = 2-OMe, 5-F).*

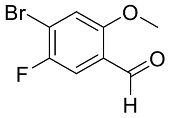

^{1}H NMR (600 MHz, DMSO-*d*$_6$) δ 3.93 (s, 3H, CH_3), 7.53 (d, *J* = 8.4 Hz, 1H, 3-H), 7.62 (d, *J* = 5.3 Hz, 1H, 6-H), 10.24 (s, 1H, CHO); ^{13}C NMR (151 MHz, DMSO-*d*$_6$) 57.2 (CH_3), 114.2 (d, $^{2}J_{F,C}$ = 24.2 Hz, C-4), 116.6 (d, $^{2}J_{F,C}$ = 23.0 Hz, C-6), 118.5 (C-3), 124.5 (d, $^{3}J_{F,C}$ = 5.3 Hz, C-1), 152.9 (d, $^{1}J_{F,C}$ = 239.8 Hz, C-5), 158.0 (C-2), 187.9 (CHO).

*tert-Butyl (4-Bromo-3-methoxybenzyl)carbamate (****41g****).*

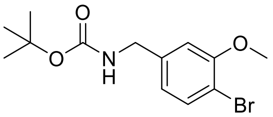

^{1}H NMR (400 MHz, DMSO-*d*$_6$) δ 1.39 (s, 9H, $C(CH_3)_3$), 3.82 (s, 3H, OCH_3), 4.10 (d, *J* = 6.2 Hz, 2H, CH_2), 6.76 (dd, *J* = 8.1, 1.9 Hz, 1H, Ar-H), 6.99 (d, *J* = 1.8 Hz, 1H, Ar-H), 7.42 (t, *J* = 6.2 Hz, 1H, NH), 7.49 (d, *J* = 8.1 Hz, 1H, Ar-H); ^{13}C NMR (101 MHz, DMSO-*d*$_6$) δ 28.2 ($C(\underline{C}H_3)_3$), 43.1 (CH_2), 56.0 (OCH_3), 77.9 ($\underline{C}(CH_3)_3$), 108.5 (C-4), 111.3 (C-2), 120.3 (C-6), 132.6, 141.6 (C-1, C-5), 155.2 (CO), 155.8 (C-3).

*tert-Butyl N-((4-Bromo-3-fluoro-phenyl)methyl)carbamate (****41h****).*

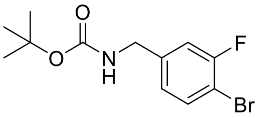

^{1}H NMR (500 MHz, DMSO-*d*$_6$) δ 1.38 (s, 9H, $C(CH_3)_3$), 4.10 (d, *J* = 6.1 Hz, 2H, CH_2), 7.03 (dd, *J* = 1.9, 8.2 Hz, 1H, 2-H), 7.19 (dd, *J* = 2.0, 9.9 Hz, 1H, 5-H), 7.42 (t, *J* = 6.4 Hz, 1H, 6-H), 7.63 (t, *J* = 7.8 Hz, 1H, NH); ^{13}C NMR (126 MHz, DMSO-*d*$_6$) δ 28.3 ($C(\underline{C}H_3)_3$), 42.7 (CH_2), 78.2 ($\underline{C}(CH_3)_3$), 105.8 (d, $^{2}J_{F,C}$ = 20.8 Hz, C-4), 115.2 (d, $^{2}J_{F,C}$ = 22.3 Hz, C-2), 124.7 (d, $^{3}J_{F,C}$ = 3.3 Hz, C-6), 133.4 (C-5), 143.1 (d, $^{3}J_{F,C}$ = 6.1 Hz, C-1), 155.9 (CO), 158.2 (d, $^{1}J_{F,C}$ = 244.6 Hz, C-3).

*tert-Butyl (4-Bromo-2,6-dimethylbenzyl)carbamate (****41j****).*

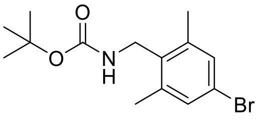

^{1}H NMR (400 MHz, DMSO-*d*$_6$) δ 1.37 (s, 9H, $C(CH_3)_3$), 2.30 (s, 6H, CH_3), 4.11 (d, *J* = 5.4 Hz, 2H, CH_2), 7.02 (t, *J* = 5.3 Hz, 1H, NH), 7.20 (s, 2H, Ar-H); ^{13}C NMR (101 MHz, DMSO-*d*$_6$) δ 19.1 (CH_3), 28.2 ($C(\underline{C}H_3)_3$), 37.8 (CH_2), 77.7 ($\underline{C}(CH_3)_3$), 119.8 (C-4), 130.2 (C-2, C-6), 134.9, 140.0 (C-1, C-3, C-5), 155.5 (CO).

*tert-Butyl (4-Bromo-2,6-dimethoxybenzyl)carbamate (**41k**).*

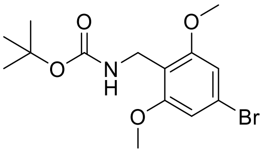

^{1}H NMR (400 MHz, DMSO-d_6) δ 1.36 (s, 9H, $C(CH_3)_3$), 3.77 (s, 6H, OCH_3), 4.09 (d, J = 5.1 Hz, 2H, CH_2), 6.40 (t, J = 5.2 Hz, 1H, NH), 6.82 (s, 2H, Ar-H); ^{13}C NMR (101 MHz, DMSO-d_6) δ 28.3 ($C(\underline{C}H_3)_3$), 32.6 (CH_2), 56.2 (OCH_3), 77.4 ($\underline{C}(CH_3)_3$), 107.5 (C-3, C-5), 113.3 (C-1), 121.5 (C-4), 155.2 (CO), 158.9 (C-2, C-6).

*tert-Butyl (4-Bromo-2,6-difluorobenzyl)carbamate (**41l**).*

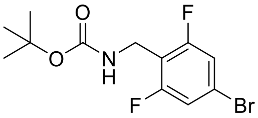

^{1}H NMR (400 MHz, DMSO-d_6) δ 1.35 (s, 9H, $C(CH_3)_3$), 4.12 (d, J = 5.5 Hz, 2H, CH_2), 7.31 (t, J = 5.6 Hz, 1H, NH), 7.43 (d, J = 6.9 Hz, 2H, Ar-H); ^{13}C NMR (101 MHz, DMSO-d_6) δ 28.2 ($C(\underline{C}H_3)_3$), 31.8 (CH_2), 78.0 ($\underline{C}(CH_3)_3$), 114.6 (t, $^2J_{F,C}$ = 19.3 Hz, C-1), 115.3 (dd, $^2J_{F,C}$ = 20.6 Hz, $^3J_{F,C}$ = 8.8 Hz, C-3, C-5), 120.4 (t, $^2J_{F,C}$ = 12.9 Hz, C-4), 155.2 (CO), 161.0 (dd, $^1J_{F,C}$ = 251.7 Hz, $^3J_{F,C}$ = 9.6 Hz, C-2, C-6).

*tert-Butyl (4-Bromo-2,6-dichlorobenzyl)carbamate (**41m**).*

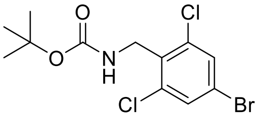

^{1}H NMR (400 MHz, DMSO-d_6) δ 1.37 (s, 9H, $C(CH_3)_3$), 4.32 (d, J = 4.9 Hz, 2H, CH_2), 7.13 (t, J = 5.1 Hz, 1H, NH), 7.76 (s, 2H, 3-H, 5-H); ^{13}C NMR (101 MHz, DMSO-d_6) δ 28.2 ($C(\underline{C}H_3)_3$), 40.2 (CH_2), 77.9 ($\underline{C}(CH_3)_3$), 121.1, 130.8, 133.3 (C-2, C-3, C-4, C-5, C-6), 136.5 (C1), 155.2 (CO).

tert-Butyl (4-Bromo-2,5-dimethylbenzyl)carbamate (***41n***).

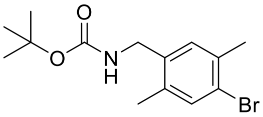

^{1}H NMR (400 MHz, DMSO-d_6) δ 1.39 (s, 9H, $C(CH_3)_3$), 2.20 (s, 3H, CH_3), 2.28 (s, 3H, CH_3), 4.03 (d, J = 6.0 Hz, 2H, CH_2), 7.11 (s, 1H, Ar-H), 7.29 (t, J = 6.0 Hz, 1H, NH), 7.35 (s, 1H, Ar-H); ^{13}C NMR (101 MHz, DMSO-d_6) δ 17.6 (CH_3), 22.0 (CH_3), 28.2 ($C(\underline{C}H_3)_3$), 40.9 (CH_2), 77.8 ($\underline{C}(CH_3)_3$), 121.8 (C-4), 129.8, 132.9, 133.9, 135.3, 137.4 (C-1, C-2, C-3, C-5, C-6), 155.7 (CO).

*tert-Butyl (4-Bromo-2,5-difluorobenzyl)carbamate (**41p**).*

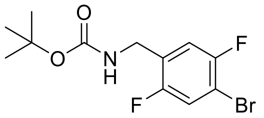

^{1}H NMR (400 MHz, DMSO-d_6) δ 1.38 (s, 9H, $C(CH_3)_3$), 4.12 (d, J = 6.0 Hz, 2H, CH_2), 7.23 (dd, J = 9.1, 6.3 Hz, 1H, Ar-H), 7.46 (t, J = 6.0 Hz, 1H, NH), 7.68 (dd, J = 9.1, 5.7 Hz, 1H, Ar-H); ^{13}C NMR (101 MHz, DMSO-d_6) δ 28.1 ($C(\underline{C}H_3)_3$), 36.4 (d, $^4J_{F,C}$ = 3.7 Hz, CH_2), 78.3 ($\underline{C}(CH_3)_3$), 106.3 (dd, $^2J_{F,C}$ = 23.5 Hz, $^3J_{F,C}$ = 10.1 Hz, C-2), 116.2 (dd, $^2J_{F,C}$ = 25.3 Hz, $^3J_{F,C}$ = 5.5 Hz, C-4), 120.0 (d, $^2J_{F,C}$ = 27.2 Hz, C-5), 128.8 (dd, $^2J_{F,C}$ = 17.5 Hz, $^3J_{F,C}$ = 6.5 Hz, C-1), 154.8 (dd, $^1J_{F,C}$ = 241.75 Hz, $^4J_{F,C}$ = 3.1 Hz, C-6), 155.1 (dd, $^1J_{F,C}$ = 245.1 Hz, $^4J_{F,C}$ = 2.1 Hz, C-3), 155.6 (CO).

*tert-Butyl (4-Bromo-2,3-dimethylbenzyl)carbamate (**41r**).*

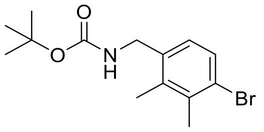

^{1}H NMR (400 MHz, DMSO-d_6) δ 1.39 (s, 9H, $C(CH_3)_3$), 2.22 (s, 3H, CH_3), 2.34 (s, 3H, CH_3), 4.08 (d, J = 6.0 Hz, 2H, CH_2), 6.96 (d, J = 8.3 Hz, 1H, Ar-H), 7.31 (t, J = 6.0 Hz, 1H, NH), 7.39 (d, J = 8.3 Hz, 1H, Ar-H); ^{13}C NMR (101 MHz, DMSO-d_6) δ 15.7 (CH_3), 19.6 (CH_3), 28.2 ($C(\underline{C}H_3)_3$), 41.9 (CH_2), 77.8 ($\underline{C}(CH_3)_3$), 123.1, 126.7, 129.2 (C-4, C-5, C-6), 135.3, 136.5, 137.3 (C-1, C-2, C-3), 155.6 (CO).

*tert-Butyl (4-Bromo-2,3-difluorobenzyl)carbamate (**41s**).*

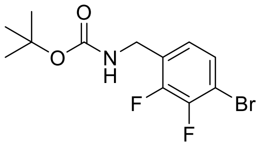

^{1}H NMR (500 MHz, DMSO-d_6) δ 1.38 (s, 9H, $C(CH_3)_3$), 4.17 (d, J = 6.0 Hz, 2H, CH_2), 7.05 – 7.15 (m, 1H, Ar-H), 7.41 – 7.48 (m, 1H, Ar-H), 7.49 – 7.55 (m, 1H, NH); ^{13}C NMR (126 MHz, DMSO-d_6) δ 28.1 ($C(\underline{C}H_3)_3$), 36.8 (CH_2), 78.2 ($\underline{C}(CH_3)_3$), 107.3 (d, $^2J_{F,C}$ = 17.5 Hz, C-4), 125.0 (d, $^3J_{F,C}$ = 4.9 Hz, C-6), 127.8 (d, $^3J_{F,C}$ = 4.0 Hz, C-5), 129.2 (d, $^2J_{F,C}$ = 11.8 Hz, C-1), 146.4 (dd, $^1J_{F,C}$ = 136.0 Hz, $^2J_{F,C}$ = 14.0 Hz, C-2), 148.4 (dd, $^1J_{F,C}$ = 140.3 Hz, $^2J_{F,C}$ = 14.0 Hz, C-3), 155.6 (CO);

*tert-Butyl ((4-Bromonaphthalen-1-yl)methyl)carbamate (**41v**).*

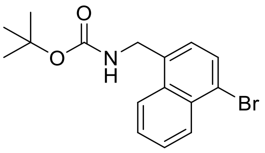

^{1}H NMR (400 MHz, DMSO-d_6) δ 1.40 (s, 9H, $C(CH_3)_3$), 4.58 (d, J = 6.0 Hz, 2H, CH_2), 7.32 (d, J = 7.7 Hz, 1H, Ar-H), 7.52 (t, J = 6.0 Hz, 1H, NH), 7.69 (dddd, J = 19.1, 8.3, 6.8, 1.3 Hz, 2H, Ar-H), 7.86 (d, J = 7.7 Hz, 1H, Ar-H), 8.15 – 8.21 (m, 2H, Ar-H); ^{13}C NMR (101 MHz, DMSO-d_6) δ 28.2 ($C(\underline{C}H_3)_3$),

41.2 (CH_2), 78.0 ($\underline{C}(CH_3)_3$), 120.9, 124.3, 125.6, 127.0, 127.2, 127.6 (C-2, C-3, C-5, C-6, C-7, C-8), 129.6, 131.1, 132.0, 135.9 (C-1, C-4, C-4a, C-8a), 155.7 (CO).

*tert-Butyl N-((5-Bromo-8-quinolyl)methyl)carbamate (**41w**).*

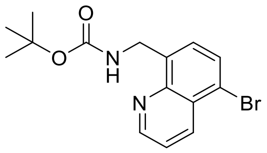

^{1}H NMR (500 MHz, DMSO-d_6) δ 1.41 (s, 9H, $C(CH_3)_3$), 4.75 (d, J = 6.1 Hz, 2H, CH_2), 7.36 (t, J = 6.0 Hz, 1H, NH), 7.52 (d, J = 7.8 Hz, 1H, 7-H), 7.69 – 7.75 (m, 1H, 3-H), 7.96 (d, J = 7.6 Hz, 1H, 6-H), 8.48 – 8.54 (m, 1H, 4-H), 8.97 – 9.02 (m, 1H, 2-H); ^{13}C NMR (126 MHz, DMSO-d_6) δ 28.4 ($C(\underline{C}H_3)_3$), 78.1 ($\underline{C}(CH_3)_3$), 119.5 (C-5), 123.1 (C-3), 126.6 (C-7), 127.2 (C-4a), 130.3 (C-6), 135.2 (C-4), 138.2 (C-8), 146.1 (C-8a), 150.6 (C-2), 156.0 (CO); the signal for CH_2 is missing.

*tert-Butyl (4-Bromo-5-fluoro-2-methylbenzyl)carbamate (**41x**).*

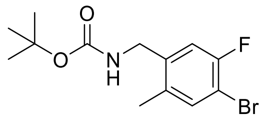

^{1}H NMR (500 MHz, DMSO-d_6) δ 1.40 (s, 9H, $C(CH_3)_3$), 2.23 (s, 3H, CH_3), 4.06 (d, J = 6.0 Hz, 2H, CH_2), 7.07 (d, J = 10.0 Hz, 1H), 7.34 – 7.40 (m, 1H), 7.48 (d, J = 7.2 Hz, 1H) (3-H, 6-H, NH); ^{13}C NMR (126 MHz, DMSO-d_6) δ 17.3 (CH_3), 28.1 ($C(\underline{C}H_3)_3$), 40.8 (CH_2), 78.0 ($\underline{C}(CH_3)_3$), 105.0 (d, $^2J_{F,C}$ = 20.5 Hz, C-4), 114.7 (d, $^2J_{F,C}$ = 22.7 Hz, C-6), 133.4 (d, $^3J_{F,C}$ = 3.5 Hz, C-3), 134.0 (C-2), 140.2 (d, $^3J_{F,C}$ = 5.9 Hz, C-1), 155.7 (CO), 156.5 (d, $^1J_{F,C}$ = 241.8 Hz, C-5).

*tert-Butyl (4-Bromo-5-fluoro-2-methoxybenzyl)carbamate (**41y**).*

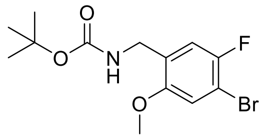

^{1}H NMR (500 MHz, DMSO-d_6) δ 1.39 (s, 9H, $C(CH_3)_3$), 3.81 (s, 3H, OCH_3), 4.04 (d, J = 6.1 Hz, 2H, CH_2), 7.05 (d, J = 9.4 Hz, 1H), 7.21 – 7.30 (m, 2H) (3-H, 6-H, NH); ^{13}C NMR (126 MHz, DMSO-d_6) δ 28.1 ($C(\underline{C}H_3)_3$), 37.9 (CH_2), 56.3 (OCH_3), 78.0 ($\underline{C}(CH_3)_3$), 105.4 (d, $^2J_{F,C}$ = 22.4 Hz, C-4), 114.7 (d, $^2J_{F,C}$ = 24.6 Hz, C-6), 115.0 (C-3), 129.7 (d, $^3J_{F,C}$ = 5.6 Hz, C-1), 152.6 (d, $^1J_{F,C}$ = 236.7 Hz, C-5), 153.1 (C-2), 155.7 (CO).

*tert-Butyl N-((4-Bromo-2-hydroxy-3-methoxyphenyl)methyl)carbamate (**41α**).*

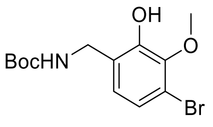

^{1}H NMR (500 MHz, DMSO-d_6) δ 1.38 (s, 9H, $C(CH_3)_3$), 3.70 (s, 3H, OCH_3), 4.05 (d, J = 6.1 Hz, 2H, CH_2), 6.79 (d, J = 8.5 Hz, 1H, 6-H), 7.00 (d, J = 8.2 Hz, 1H, 5-H), 7.21 (t, J = 6.2 Hz, 1H, NH), 9.40 (s, 1H, OH); ^{13}C NMR (126 MHz, DMSO-d_6) δ 28.4 ($C(\underline{C}H_3)_3$), 38.5 (CH_2), 60.4 (OCH_3), 78.1 ($\underline{C}(CH_3)_3$), 114.5 (C-4), 122.4 (C-6), 124.1 (C-5), 128.0 (C-1), 144.7 (C-3), 148.6 (C-2), 156.2 (CO).

*tert-Butyl N-((4-Bromo-2,3-dimethoxyphenyl)methyl)carbamate (**41β**).*

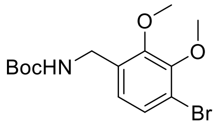

^{1}H NMR (500 MHz, DMSO-d_6) δ 1.38 (d, J = 1.3 Hz, 9H, $C(CH_3)_3$), 3.78 (dd, J = 1.4, 10.4 Hz, 6H, OCH_3), 4.09 (d, J = 6.0 Hz, 2H, CH_2), 6.90 (d, J = 8.5 Hz, 1H), 7.30 – 7.35 (m, 2H) (5-H, 6-H, NH); ^{13}C NMR (126 MHz, DMSO-d_6) δ 28.4 ($C(\underline{C}H_3)_3$), 38.0 (CH_2), 60.5, 60.8 (OCH_3), 78.0 ($\underline{C}(CH_3)_3$), 115.1 (C-4), 124.3 (C-6), 127.5 (C-5), 134.4 (C-1), 149.8 (C-3), 151.4 (C-2), 155.8 (CO).

*tert-Butyl N-((4-Bromo-2,5-dichlorophenyl)methyl)carbamate (**41γ**).*

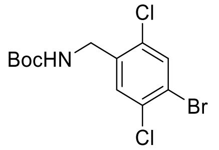

^{1}H NMR (600 MHz, $CDCl_3$) δ 1.41 (s, 9H, CH_3), 5.64 (s, 2H, CH_2), 6.20 (t, J = 7.8 Hz, 1H, NH), 7.59 (s, 1H, Ar-H), 7.62 (s, 1H, Ar-H); ^{13}C NMR (151 MHz, $CDCl_3$) δ 28.3 ($C(\underline{C}H_3)_3$), 59.5 (CH_2), 80.8 ($\underline{C}(CH_3)_3$), 122.5 (C-4), 129.4 (C-5), 131.3 (C-6), 133.3 (C-2), 134.3 (C-1), 137.8 (C-3), 154.3 (CO).

*tert-Butyl N-((4-Bromo-3-fluoro-2-hydroxyphenyl)methyl)carbamate (**41δ**).*

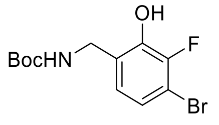

^{1}H NMR (500 MHz, DMSO-d_6) δ 1.38 (s, 9H, $C(CH_3)_3$), 4.07 (d, J = 6.2 Hz, 2H, CH_2), 6.86 (d, J = 8.5 Hz, 1H, 6-H), 7.06 (dd, J = 8.3, 6.2 Hz, 1H, 5-H), 7.26 (t, J = 6.2 Hz, 1H, NH), 10.03 (s, 1H, OH); ^{13}C NMR (126 MHz, DMSO-d_6) δ 28.3 ($C(\underline{C}H_3)_3$), 38.3 (CH_2), 78.2 ($\underline{C}(CH_3)_3$), 106.4 (d, $^2J_{F,C}$ = 18.5 Hz, C-4), 122.3 (C-1), 124.0 (d, $^3J_{F,C}$ = 3.5 Hz, C-5), 129.7 (C-6), 143.1 (d, $^2J_{F,C}$ = 14.8 Hz, C-2), 148.2 (d, $^1J_{F,C}$ = 239.2 Hz, C-3), 156.1 (CO).

*tert-Butyl N-((4-Bromo-2-fluoro-3-methoxyphenyl)methyl)carbamate (**41ε**).*

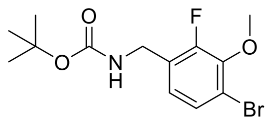

^{1}H NMR (600 MHz, DMSO-d_6) δ 1.38 (s, 9H, $C(CH_3)_3$), 3.84 (s, 3H, OCH_3), 4.13 (d, J = 6.0 Hz, 2H, CH_2), 6.97 (t, J = 7.8 Hz, 1H, NH), 7.37 – 7.43 (m, 2H, Ar-H); ^{13}C NMR (151 MHz, DMSO-d_6) δ 28.3 ($C(\underline{C}H_3)_3$), 37.1 (d, J = 5.2 Hz, CH_2), 61.5 (d, J = 4.3 Hz, OCH_3), 78.3 ($\underline{C}(CH_3)_3$), 115.0 (C-5), 124.5 (d, $^3J_{F,C}$ = 4.5 Hz), 127.9 (d, $^3J_{F,C}$ = 4.2 Hz, C-4, C-6), 128.6 (d, $^2J_{F,C}$ = 13.4 Hz, C-1), 144.6 (d, $^2J_{F,C}$ = 13.2 Hz, C-3), 153.5 (d, $^1J_{F,C}$ = 249.6 Hz, C-2), 155.8 (CO).

*tert-Butyl (2-Fluoro-4-(4-methylthiazol-5-yl)benzyl)carbamate (**42d**).*

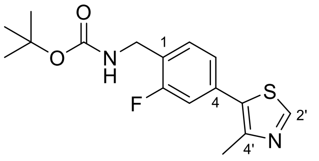

^{1}H NMR (600 MHz, DMSO-d_6) δ 1.40 (s, 9H, $C(CH_3)_3$), 2.46 (s, 3H, CH_3), 4.21 (d, J = 6.0 Hz, 2H, CH_2), 7.31 (d, J = 9.4 Hz, 2H), 7.38 (t, J = 7.8 Hz, 1H), 7.43 (t, J = 6.1 Hz, 1H) (Ar-H, CONH), 9.02 (s, 1H, 2′-H); ^{13}C NMR (151 MHz, DMSO-d_6) δ 15.9 (CH_3), 28.2 ($C(\underline{C}H_3)_3$), 36.9 (CH_2), 78.0 ($\underline{C}(CH_3)_3$), 115.3 (d, $^2J_{F,C}$ = 22.6 Hz, C-3), 125.0 (d, $^4J_{F,C}$ = 3.2 Hz, C-5′), 126.5 (d, $^2J_{F,C}$ = 15.1 Hz, C-1), 129.6 (d, $^3J_{F,C}$ = 5.2 Hz, C-6), 129.8 (d, $^4J_{F,C}$ = 1.7 Hz, C-5), 132.0 (d, $^3J_{F,C}$ = 8.5 Hz, C-4), 148.5 (C-4′), 152.0 (C-2′), 155.7 (CO), 159.7 (d, $^1J_{F,C}$ = 245.5 Hz, C-2).

*tert-Butyl (2-Chloro-4-(4-methylthiazol-5-yl)benzyl)carbamate (**42e**).*

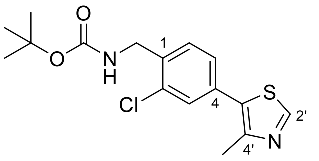

^{1}H NMR (500 MHz, DMSO-d_6) δ 1.41 (s, 9H, $C(CH_3)_3$), 2.46 (s, 3H, CH_3), 4.24 (d, J = 6.1 Hz, 2H, CH_2), 7.40 (d, J = 8.0 Hz, 1H), 7.44 – 7.49 (m, 2H), 7.53 (d, J = 1.8 Hz, 1H) (Ar-H, CONH), 9.02 (s, 1H, 2′-H); ^{13}C NMR (126 MHz, DMSO-d_6) δ 15.9 (CH_3), 28.2 ($C(\underline{C}H_3)_3$), 41.0 ($NHCH_2$), 78.1 ($\underline{C}(CH_3)_3$), 127.8, 128.6, 128.9, 129.5, 131.7, 132.1 (C-2, C-3, C-4, C-5, C-5′, C-6), 136.7 (C-1), 148.6 (C-4′), 152.1 (C-2′), 155.7 (CO).

*tert-Butyl (3-Methyl-4-(4-methylthiazol-5-yl)benzyl)carbamate (**42f**).*

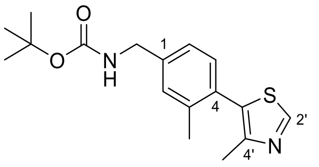

^{1}H NMR (500 MHz, DMSO-d_6) δ 1.41 (s, 9H, $C(CH_3)_3$), 2.12 (s, 3H, CH_3), 2.17 (s, 3H, CH_3), 4.15 (d, J = 6.2 Hz, 2H, CH_2), 7.13 (dd, J = 8.0, 1.7 Hz, 1H, Ar-H), 7.19 – 7.22 (m, 2H, Ar-H), 7.38 (t, J = 5.8 Hz, 1H, CONH), 9.04 (s, 1H, 2′-H); ^{13}C NMR (126 MHz, DMSO-d_6) δ 15.2 (CH_3), 19.7 (Ar-CH_3), 28.2

(C(C̲H$_3$)$_3$), 43.0 (NHCH$_2$), 77.8 (C̲(CH$_3$)$_3$), 124.4, 128.7, 128.8, 129.5, 130.9, 136.9 (C-2, C-3, C-4, C-5, C-5′, C-6), 140.8 (C-1), 149.0 (C-4′), 152.1 (C-2′), 155.8 (CO).

*tert-Butyl (3-Methoxy-4-(4-methylthiazol-5-yl)benzyl)carbamate (**42g**).*

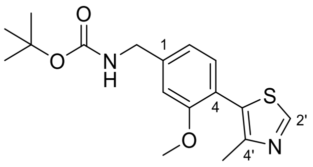

^{1}H NMR (400 MHz, CDCl$_3$) δ 1.48 (s, 9H, C(CH$_3$)$_3$), 2.39 (s, 3H, CH$_3$), 3.82 (s, 3H, OCH$_3$), 4.35 (d, J = 6.1 Hz, 2H, CH$_2$), 4.94 (s, 1H, NH), 6.92 (d, J = 6.5 Hz, 2H, Ar-H), 7.25 (d, J = 8.2 Hz, 1H, Ar-H), 8.73 (s, 1H, 2′-H); ^{13}C NMR (101 MHz, CDCl$_3$) δ 16.3 (CH$_3$), 28.5 (C(C̲H$_3$)$_3$), 44.8 (CH$_2$), 55.7 (OCH$_3$), 79.9 (C̲(CH$_3$)$_3$), 110.4, 119.4, 119.7, 127.0, 132.2 (C-2, C-4, C-5, C-6, C-5′), 141.4 (C-1), 150.4 (C-4′), 151.3 (C-2′), 156.1 (C-3), 157.3 (CO).

*tert-Butyl (3-Fluoro-4-(4-methylthiazol-5-yl)benzyl)carbamate (**42h**).*

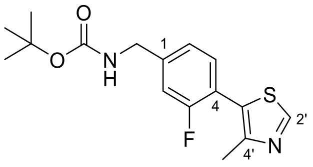

^{1}H NMR (600 MHz, DMSO-d_6) δ 1.41 (s, 9H, C(CH$_3$)$_3$), 2.33 (d, J = 1.0 Hz, 3H, CH$_3$), 4.19 (d, J = 6.2 Hz, 2H, CH$_2$), 7.16 – 7.21 (m, 2H), 7.43 – 7.50 (m, 2H) (Ar-H, CONH), 9.10 (s, 1H, 2′-H); ^{13}C NMR (151 MHz, DMSO-d_6) δ 15.7 (CH$_3$), 28.2 (C(C̲H$_3$)$_3$), 42.7 (NHCH$_2$), 78.0 (C̲(CH$_3$)$_3$), 114.2 (d, $^2J_{F,C}$ = 22.9 Hz, C-2), 117.0 (d, $^2J_{F,C}$ = 15.3 Hz, C-4), 123.1 (d, $^3J_{F,C}$ = 3.2 Hz, C-5′), 123.7 (C-6), 132.0 (d, $^3J_{F,C}$ = 2.8 Hz, C-5), 143.9 (d, $^3J_{F,C}$ = 7.2 Hz, C-1), 150.2 (C-4′), 153.1 (C-2′), 155.8 (CO), 158.8 (d, $^1J_{F,C}$ = 246.6 Hz, C-3).

*tert-Butyl (3-Chloro-4-(4-methylthiazol-5-yl)benzyl)carbamate (**42i**).*

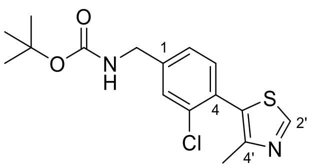

^{1}H NMR (500 MHz, DMSO-d_6) δ 1.41 (s, 9H, C(CH$_3$)$_3$), 2.23 (s, 3H, CH$_3$), 4.19 (d, J = 6.2 Hz, 2H, CH$_2$), 7.27 – 7.31 (m, 1H), 7.42 – 7.50 (m, 3H) (Ar-H, CONH), 9.09 (s, 1H, 2′-H); ^{13}C NMR (126 MHz, DMSO-d_6) δ 15.5 (CH$_3$), 28.2 (C(C̲H$_3$)$_3$), 42.6 (NHCH$_2$), 78.1 (C̲(CH$_3$)$_3$), 125.9, 127.2, 128.0, 128.1, 132.7, 133.1 (C-2, C-3, C-4, C-5, C-5′, C-6), 143.2 (C-1), 150.2 (C-4′), 152.9 (C-2′), 155.8 (CO).

*tert-Butyl (2,6-Dimethyl-4-(4-methylthiazol-5-yl)benzyl)carbamate (**42j**).*

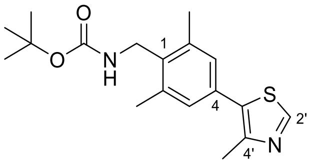

^{1}H NMR (400 MHz, $CDCl_3$) δ 1.45 (s, 9H, $C(CH_3)_3$), 2.41 (s, 6H, CH_3), 2.52 (s, 3H, CH_3), 4.38 (d, *J* = 4.9 Hz, 2H, CH_2), 4.45 (s, 1H, NH), 7.11 (s, 2H, Ar-H), 8.66 (s, 1H, 2′-H); ^{13}C NMR (101 MHz, $CDCl_3$) δ 16.3 (CH_3), 19.9 (CH_3), 28.5 ($C(\underline{C}H_3)_3$), 39.0 (CH_2), 79.6 ($\underline{C}(CH_3)_3$), 129.3, 131.4, 131.7, 134.4 (C-2, C-3, C-4, C-5, C-6, C-5′), 138.1 (C-1), 148.6 (C-4′), 150.3 (C-2′), 155.8 (CO).

*tert-Butyl (2,6-Dimethoxy-4-(4-methylthiazol-5-yl)benzyl)carbamate (**42k**).*

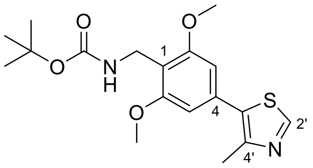

^{1}H NMR (400 MHz, $CDCl_3$) δ 1.44 (s, 9H, $C(CH_3)_3$), 2.54 (s, 3H, CH_3), 3.85 (s, 6H, OCH_3), 4.42 (d, *J* = 5.8 Hz, 2H, CH_2), 5.01 (s, 1H, NH), 6.58 (s, 2H, Ar-H), 8.67 (s, 1H, 2′-H); ^{13}C NMR (101 MHz, $CDCl_3$) δ 16.3 (CH_3), 28.6 ($C(\underline{C}H_3)_3$), 33.4 (CH_2), 56.1 (OCH_3), 79.1 ($\underline{C}(CH_3)_3$), 105.3 (C-3, C-5), 114.9, 132.6 (C-4, C-5′), 148.8 (C-1), 150.4 (C-2′, C-4′), 155.9 (CO), 158.6 (C-2, C-6).

*tert-Butyl (2,6-Difluoro-4-(4-methylthiazol-5-yl)benzyl)carbamate (**42l**).*

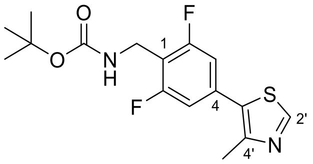

^{1}H NMR (400 MHz, $CDCl_3$) δ 1.44 (s, 9H, $C(CH_3)_3$), 2.54 (s, 3H, CH_3), 4.44 (d, *J* = 4.7 Hz, 2H, CH_2), 4.95 (s, 1H, NH), 6.94 – 7.02 (m, 2H, Ar-H), 8.71 (s, 1H, 2′-H); ^{13}C NMR (101 MHz, $CDCl_3$) δ 16.4 (CH_3), 28.5 ($C(\underline{C}H_3)_3$), 32.6 (CH_2), 80.0 ($\underline{C}(CH_3)_3$), 112.4 (dd, $^2J_{F,C}$ = 18.9 Hz, $^4J_{F,C}$ = 7.3 Hz, C-3, C-5), 114.2 (t, $^2J_{F,C}$ = 18.4 Hz, C-1), 129.6 (t, $^4J_{F,C}$ = 2.2 Hz, C-5′), 133.8 (t, $^3J_{F,C}$ = 10.7 Hz, C-4), 149.8 (C-4′), 151.3 (C-2′), 155.5 (CO), 161.5 (dd, $^1J_{F,C}$ = 249.9 Hz, $^3J_{F,C}$ = 9.3 Hz, C-2, C-6).

*tert-Butyl (2,6-Dichloro-4-(4-methylthiazol-5-yl)benzyl)carbamate (**42m**).*

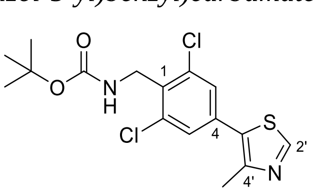

^{1}H NMR (400 MHz, $CDCl_3$) δ 1.45 (s, 9H, $C(CH_3)_3$), 2.53 (s, 3H, CH_3), 4.66 (d, *J* = 5.9 Hz, 2H, CH_2), 4.92 (s, 1H, NH), 7.39 (s, 2H, Ar-H), 8.72 (s, 1H, 2′-H); ^{13}C NMR (101 MHz, $CDCl_3$) δ 16.3 (CH_3),

28.5 ($C(\underline{C}H_3)_3$), 40.1 (CH_2), 79.9 ($\underline{C}(CH_3)_3$), 128.8, 129.0, 133.7, 133.9 (C-2, C-3, C-4, C-5, C-6, C-5′), 136.4 (C-1), 150.1 (C-4′), 151.4 (C-2′), 155.4 (CO).

*tert-Butyl (2,5-Dimethyl-4-(4-methylthiazol-5-yl)benzyl)carbamate (**42n**).*

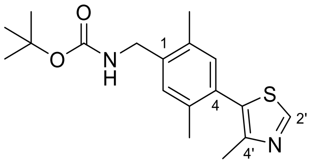

^{1}H NMR (400 MHz, $CDCl_3$) δ 1.47 (s, 9H, $C(CH_3)_3$), 2.14 (s, 3H, CH_3), 2.27 (s, 3H, CH_3), 2.29 (s, 3H, CH_3), 4.31 (d, J = 5.8 Hz, 2H, CH_2), 4.78 (s, 1H, NH), 7.03 (s, 1H, Ar-H), 7.15 (s, 1H, Ar-H), 8.72 (s, 1H, 2′-H); ^{13}C NMR (101 MHz, $CDCl_3$) δ 15.5 (CH_3), 18.5 (CH_3), 19.7 (CH_3), 28.6 ($C(\underline{C}H_3)_3$), 42.6 (CH_2), 79.7 ($\underline{C}(CH_3)_3$), 129.9, 130.2, 133.3, 133.6, 135.6 (C-2, C-3, C-4, C-5, C-6, C-5′), 137.2 (C-1), 149.8 (C-4′), 151.1 (C-2′), 155.9 (CO).

tert-Butyl N-((2,5-Dimethoxy-4-(4-methylthiazol-5-yl)phenyl)methyl)carbamate (***42o***).

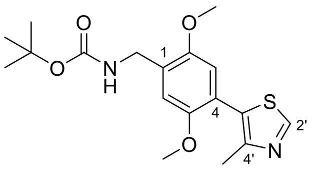

^{1}H NMR (500 MHz, DMSO-d_6) δ 1.41 (s, 9H, $C(CH_3)_3$), 2.30 (s, 3H, CH_3), 3.69 (s, 3H), 3.75 (s, 3H, OCH_3), 4.13 (d, J = 6.2 Hz, 2H, CH_2), 6.89 (s, 1H), 6.95 (s, 1H) (6-H, NH), 7.24 (s, 1H, 3-H), 8.98 (s, 1H, 2′-H); ^{13}C NMR (126 MHz, DMSO-d_6) δ 16.1 (CH_3), 28.4 ($C(\underline{C}H_3)_3$), 38.5 (CH_2), 56.1, 56.1 (OCH_3), 78.0 ($\underline{C}(CH_3)_3$), 111.7 (C-3), 114.1 (C-6), 118.4, 126.8, 129.4 (C-1, C-2, C-4), 149.6, 150.2, 150.5, 152.1 (CO, C-4′, C-5, C-5′), 156.0 (C-2′).

*tert-Butyl (2,5-Difluoro-4-(4-methylthiazol-5-yl)benzyl)carbamate (**42p**).*

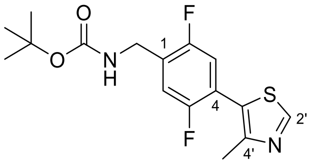

^{1}H NMR (400 MHz, $CDCl_3$) δ 1.45 (s, 9H, $C(CH_3)_3$), 2.42 (t, J = 1.5 Hz, 3H, CH_3), 4.37 (s, 2H, CH_2), 5.13 (s, 1H, NH), 7.00 – 7.08 (m, 1H, Ar-H), 7.15 (dd, J = 9.7, 6.2 Hz, 1H, Ar-H), 8.76 (s, 1H, 2′-H); ^{13}C NMR (101 MHz, $CDCl_3$) δ 16.1 (CH_3), 28.5 ($C(\underline{C}H_3)_3$), 38.3 (CH_2), 80.2 ($\underline{C}(CH_3)_3$), 116.7 (dd, $^2J_{F,C}$ = 25.7 Hz, $^3J_{F,C}$ = 5.3 Hz, C-6), 118.2 (dd, $^2J_{F,C}$ = 24.8 Hz, $^3J_{F,C}$ = 3.0 Hz, C-3), 119.7 (dd, $^2J_{F,C}$ = 18.1 Hz, $^3J_{F,C}$ = 8.8 Hz, C-4), 123.5 (C-5′), 128.6 (dd, $^2J_{F,C}$ = 17.4 Hz, $^3J_{F,C}$ = 7.2 Hz, C-1), 151.5 (C-4′), 152.2 (C-2′), 155.8 (dd, $^1J_{F,C}$ = 245.9 Hz, $^4J_{F,C}$ = 1.6 Hz, C-2), 156.0 (CO), 156.2 (dd, $^1J_{F,C}$ = 244.6 Hz, $^4J_{F,C}$ = 1.8 Hz, C-5).

*tert-Butyl (2,5-Dichloro-4-(4-methylthiazol-5-yl)benzyl)carbamate (**42q**).*

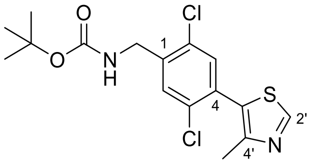

^{1}H NMR (500 MHz, DMSO-d_6) δ 1.42 (s, 9H, $C(CH_3)_3$), 2.25 (s, 3H, CH_3), 4.24 (d, *J* = 6.1 Hz, 2H, CH_2), 7.47 (s, 1H, Ar-H), 7.53 (t, *J* = 6.0 Hz, 1H, CONH), 7.61 (s, 1H, Ar-H), 9.13 (s, 1H, 2′-H); ^{13}C NMR (126 MHz, DMSO-d_6) δ 15.5 (CH_3), 28.1 (C($\underline{C}H_3$)$_3$), 41.0 (CH_2), 78.4 ($\underline{C}$(CH_3)$_3$), 125.8, 129.0, 130.1, 130.4, 132.1, 132.7 (C-2, C-3, C-4, C-5, C-5′, C-6), 139.6 (C-1), 150.8 (C-4′), 153.5 (C-2′), 155.7 (CO).

*tert-Butyl (2,3-Dimethyl-4-(4-methylthiazol-5-yl)benzyl)carbamate (**42r**).*

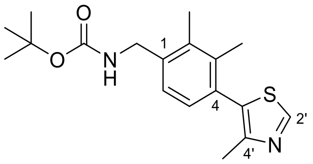

^{1}H NMR (400 MHz, $CDCl_3$) δ 1.47 (s, 9H, $C(CH_3)_3$), 2.11 (s, 3H, CH_3), 2.24 (s, 3H, CH_3), 2.28 (s, 3H, CH_3), 4.37 (d, *J* = 5.7 Hz, 2H, CH_2), 4.77 (s, 1H, NH), 7.08 (d, *J* = 7.8 Hz, 1H, Ar-H), 7.14 (d, *J* = 7.9 Hz, 1H, Ar-H), 8.72 (s, 1H, 2′-H); ^{13}C NMR (101 MHz, $CDCl_3$) δ 15.5 (CH_3), 15.6 (CH_3), 17.3 (CH_3), 28.6 (C($\underline{C}H_3$)$_3$), 43.6 (CH_2), 79.7 ($\underline{C}$(CH_3)$_3$), 125.6, 129.0, 130.5, 131.2, 135.9, 137.2 (C-2, C-3, C-4, C-5, C-6, C-5′), 137.3 (C-1), 149.9 (C-4′), 151.1 (C-2′), 155.8 (CO).

*tert-Butyl (2,3-Difluoro-4-(4-methylthiazol-5-yl)benzyl)carbamate (**42s**).*

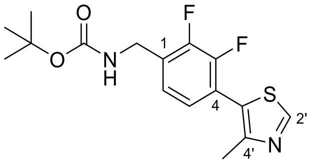

^{1}H NMR (600 MHz, DMSO-d_6) δ 1.40 (s, 9H, $C(CH_3)_3$), 2.35 (d, *J* = 1.2 Hz, 3H, CH_3), 4.25 (d, *J* = 6.0 Hz, 2H, CH_2), 7.20 (t, *J* = 7.4 Hz, 1H, Ar-H), 7.31 (t, *J* = 7.1 Hz, 1H, Ar-H), 7.50 (t, *J* = 6.0 Hz, 1H, CONH), 9.15 (s, 1H, 2′-H); ^{13}C NMR (151 MHz, DMSO-d_6) δ 15.7 (CH_3), 28.2 (C($\underline{C}H_3$)$_3$), 37.0 (CH_2), 78.2 ($\underline{C}$(CH_3)$_3$), 119.4 (d, $^2J_{F,C}$ = 12.0 Hz, C-4), 122.6, 123.8, 126.4 (d, $^3J_{F,C}$ = 3.0 Hz, C-5, C-5′, C-6), 129.7 (d, $^2J_{F,C}$ = 11.6 Hz, C-1), 146.7 (dd, $^1J_{F,C}$ = 248.3 Hz, $^2J_{F,C}$ = 13.6 Hz, C-2, C-3), 150.8 (C-4′), 153.8 (C-2′), 155.7 (CO).

*tert-Butyl (3-Fluoro-2-hydroxy-4-(4-methylthiazol-5-yl)benzyl)carbamate (**42t**).*

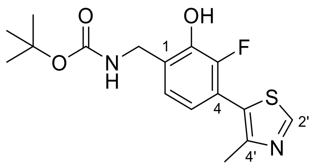

^{1}H NMR (500 MHz, DMSO-d_6) δ 1.41 (s, 9H, C(CH_3)$_3$), 2.33 (s, 3H, CH_3), 4.16 (d, *J* = 6.1 Hz, 2H, CH_2), 6.87 (t, *J* = 7.3 Hz, 1H, Ar-H), 7.00 (d, *J* = 7.9 Hz, 1H, Ar-H), 7.30 (t, *J* = 5.9 Hz, 1H, CONH), 9.08 (s, 1H, 2′-H), 9.83 (s, 1H, OH); ^{13}C NMR (126 MHz, DMSO-d_6) δ 15.7 (d, $^5J_{F,C}$ = 2.5 Hz, CH_3), 28.2 (C($\underline{C}H_3$)$_3$), 38.3 (CH_2), 78.0 ($\underline{C}$(CH_3)$_3$), 117.7 (d, $^2J_{F,C}$ = 13.5 Hz, C-4), 120.9, 122.7, 124.12, 130.1 (C-1, C-5, C-5′, C-6), 142.3 (d, $^2J_{F,C}$ = 14.6 Hz, C-2), 148.2 (d, $^1J_{F,C}$ = 241.2 Hz, C-3), 149.9 (C-4′), 152.9 (C-2′), 156.0 (CO).

*tert-Butyl (3-Fluoro-2-methoxy-4-(4-methylthiazol-5-yl)benzyl)carbamate (**42u**).*

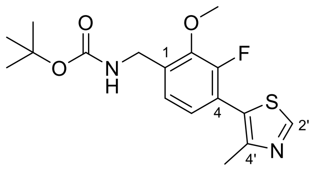

^{1}H NMR (600 MHz, DMSO-d_6) δ 1.40 (s, 9H, C(CH_3)$_3$), 2.34 (s, 3H, CH_3), 3.88 (s, 3H, OCH_3), 4.21 (d, *J* = 6.1 Hz, 2H, CH_2), 7.12 (d, *J* = 8.1 Hz, 1H, Ar-H), 7.19 (t, *J* = 7.4 Hz, 1H, Ar-H), 7.38 (t, *J* = 6.0 Hz, 1H, CONH), 9.11 (s, 1H, 2′-H); ^{13}C NMR (151 MHz, DMSO-d_6) δ 15.8 (CH_3), 28.2 (C($\underline{C}H_3$)$_3$), 37.9 (CH_2), 61.4 (d, $^4J_{F,C}$ = 4.9 Hz, OCH_3), 78.0 ($\underline{C}$(CH_3)$_3$), 118.7 (d, $^2J_{F,C}$ = 14.0 Hz, C-4), 123.2 (d, $^3J_{F,C}$ = 3.5 Hz), 123.5, 125.8 (C-5, C-5′, C-6), 135.3 (C-1), 145.0 (d, $^2J_{F,C}$ = 11.3 Hz, C-2), 150.3 (C-4′), 151.9 (d, $^1J_{F,C}$ = 247.9 Hz, C-3), 153.3 (C-2′), 155.8 (CO).

*tert-Butyl ((4-(4-Methylthiazol-5-yl)naphthalen-1-yl)methyl)carbamate (**42v**).*

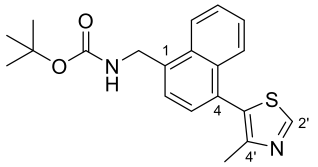

^{1}H NMR (400 MHz, $CDCl_3$) δ 1.48 (s, 9H, C(CH_3)$_3$), 2.26 (s, 3H, CH_3), 4.84 (d, *J* = 5.8 Hz, 2H, CH_2), 4.95 (s, 1H, NH), 7.43 (d, *J* = 7.3 Hz, 1H, Ar-H), 7.47 – 7.54 (m, 2H, Ar-H), 7.56 – 7.62 (m, 1H, Ar-H), 7.71 (dd, *J* = 8.8, 1.2 Hz, 1H, Ar-H), 8.12 (d, *J* = 8.5 Hz, 1H, Ar-H), 8.84 (s, 1H, 2′-H); ^{13}C NMR (101 MHz, $CDCl_3$) δ 15.8 (CH_3), 28.6 (C($\underline{C}H_3$)$_3$), 42.9 (CH_2), 79.9 ($\underline{C}$(CH_3)$_3$), 124.0, 125.2, 126.6, 126.8 (C-2, C-3, C-5, C-5′, C-6, C-7, C-8), 129.0, 131.7, 133.0, 135.6 (C-1, C-4, C-4a, C-8a), 151.0 (C-4′), 151.8 (C-2′), 155.8 (CO).

*tert-Butyl N-((5-(4-Methylthiazol-5-yl)-8-quinolyl)methyl)carbamate (**42w**).*

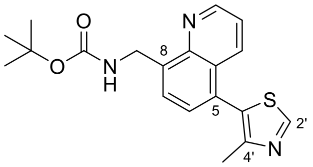

^{1}H NMR (500 MHz, DMSO-d_6) δ 1.42 (s, 9H, $C(CH_3)_3$), 2.16 (s, 3H, CH_3), 4.84 (d, J = 6.2 Hz, 2H, CH_2), 7.39 (t, J = 6.2 Hz, 1H, NH), 7.56 – 7.64 (m, 1H), 7.65 (d, J = 2.8 Hz, 2H) (3-H, 6-H, 7-H), 7.98 – 8.04 (m, 1H, 4-H), 8.98 (dd, J = 4.2, 1.8 Hz, 1H, 2-H), 9.18 (s, 1H, 2′-H); ^{13}C NMR (126 MHz, DMSO-d_6) δ 15.6 (Ar-CH_3), 28.4 (C($\underline{C}H_3)_3$), 78.0 ($\underline{C}(CH_3)_3$), 122.3 (C-3), 125.7 (C-6), 126.7 (C-5), 127.3, 127.5 (C-7, C-8), 129.5 (C-4a), 133.8 (C-4), 138.9 (C-5′), 145.5 (C-2′), 150.0 (C-2), 150.5 (C-4′), 153.4 (CO), 156.1 (C-8a); the signal for CH_2 is missing (overlapping solvent peak).

*tert-Butyl (5-Fluoro-2-methyl-4-(4-methylthiazol-5-yl)benzyl)carbamate (**42x**).*

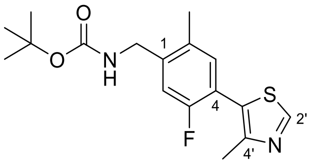

^{1}H NMR (500 MHz, DMSO-d_6) δ 1.41 (s, 9H, $C(CH_3)_3$), 2.26 (s, 3H, CH_3), 2.32 (s, 3H, CH_3), 4.13 (d, J = 6.0 Hz, 2H, CH_2), 7.07 (d, J = 11.1 Hz, 1H, Ar-H), 7.27 (d, J = 7.6 Hz, 1H, Ar-H), 7.43 (t, J = 5.9 Hz, 1H, NH), 9.09 (s, 1H, 2′-H); ^{13}C NMR (126 MHz, DMSO-d_6) δ 15.6 (d, $^{5}J_{F,C}$ = 2.6 Hz, CH_3), 17.5 (CH_3), 28.2 (C($\underline{C}H_3)_3$), 40.9 (CH_2), 78.0 ($\underline{C}(CH_3)_3$), 114.0 (d, $^{2}J_{F,C}$ = 22.8 Hz, C-6), 116.5 (d, $^{2}J_{F,C}$ = 15.0 Hz, C-4), 123.8 (C-2), 131.6 (d, $^{3}J_{F,C}$ = 3.4 Hz, C-3), 132.9 (d, $^{3}J_{F,C}$ = 2.4 Hz, C-5′), 141.1 (d, $^{3}J_{F,C}$ = 7.4 Hz, C-3), 150.0 (C-4′), 152.9 (C-2′), 155.7 (CO), 157.2 (d, $^{1}J_{F,C}$ = 244.2 Hz, C-5).

*tert-Butyl (5-Fluoro-2-methoxy-4-(4-methylthiazol-5-yl)benzyl)carbamate (**42y**).*

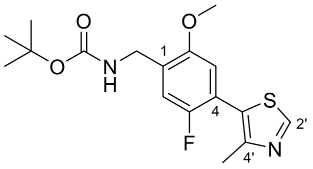

^{1}H NMR (500 MHz, DMSO-d_6) δ 1.41 (s, 9H, $C(CH_3)_3$), 2.36 (s, 3H, CH_3), 3.82 (s, 3H, OCH_3), 4.13 (d, J = 6.3 Hz, 2H, CH_2), 7.00 (d, J = 6.0 Hz, 1H), 7.05 (d, J = 10.5 Hz, 1H) (3-H, 6-H), 7.31 (t, J = 6.3 Hz, 1H, NH), 9.10 (s, 1H, 2′-H); ^{13}C NMR (126 MHz, DMSO-d_6) δ 15.7 (d, $^{5}J_{F,C}$ = 2.5 Hz, CH_3), 28.2 (C($\underline{C}H_3)_3$), 38.1 (CH_2), 56.1 (OCH_3), 78.0 ($\underline{C}(CH_3)_3$), 113.3 (d, $^{3}J_{F,C}$ = 2.5 Hz, C-3), 114.3 (d, $^{2}J_{F,C}$ = 25.2 Hz, C-6), 117.0 (d, $^{2}J_{F,C}$ = 16.5 Hz, C-4), 123.9 (C-5′), 130.7 (d, $^{3}J_{F,C}$ = 6.8 Hz, C-1), 150.3 (C-4′), 152.4 (C-2′), 153.0 (d, $^{1}J_{F,C}$ = 238.8 Hz, C-5), 153.0 (C-2), 155.8 (CO).

*Benzyl (2S,4R)-1-((S)-2-((tert-Butoxycarbonyl)amino)-3,3-dimethylbutanoyl)-4-hydroxypyrrolidine-2-carboxylate (**44**).*

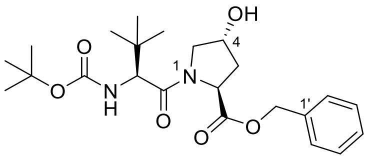

^{1}H NMR (500 MHz, DMSO-d_6) δ 0.89 (s, 9H, $C(CH_3)_3$), 1.38 (s, 9H, $OC(CH_3)_3$), 1.87 – 1.96 (m, 1H, 3-H), 2.09 – 2.18 (m, 1H, 3-H), 3.59 – 3.63 (m, 1H, 5-H), 3.67 (dd, J = 10.7, 4.1 Hz, 1H, 5-H), 4.15 (d, J = 9.4 Hz, 1H), 4.32 – 4.36 (m, 1H), 4.42 (t, J = 8.4 Hz, 1H) (2-H, 4-H, $NHC\underline{H}$), 5.07 – 5.15 (m, 2H, Ar-CH_2), 5.20 (d, J = 3.7 Hz, 1H, OH), 6.47 (d, J = 9.4 Hz, 1H, CONH), 7.29 – 7.39 (m, 5H, Ar-H); ^{13}C NMR (126 MHz, DMSO-d_6) δ 26.1 ($C(\underline{C}H_3)_3$), 28.1 ($OC(\underline{C}H_3)_3$), 35.2 ($\underline{C}(CH_3)_3$), 37.2 (C-3), 55.9, 57.8, 58.2, 65.8, 68.8 (C-2, C-4, C-5, NHCH, Ar-CH_2), 78.1 ($O\underline{C}(CH_3)_3$), 127.8 (C-2′, C-6′), 127.9 (C-4′), 128.3 (C-3′, C-5′), 135.9 (C-1′), 155.3 (CONH), 170.2, 171.6 (CO).

*Benzyl (2S,4R)-1-((S)-2-(1-Cyanocyclopropane-1-carboxamido)-3,3-dimethylbutanoyl)-4-hydroxypyrrolidine-2-carboxylate (**45**).*

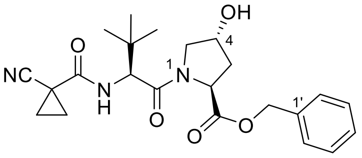

^{1}H NMR (500 MHz, DMSO-d_6) δ 0.91 (s, 9H, $C(CH_3)_3$), 1.44 – 1.54 (m, 2H, CH_2CCN), 1.57 – 1.65 (m, 2H, CH_2CCN), 1.89 – 1.97 (m, 1H, 3-H), 2.12 – 2.19 (m, 1H, 3-H), 3.56 – 3.60 (m, 1H, 5-H), 3.65 (dd, J = 10.9, 3.9 Hz, 1H, 5-H), 4.31 – 4.36 (m, 1H), 4.46 (dd, J = 9.2, 7.8 Hz, 1H), 4.52 (d, J = 8.9 Hz, 1H) (2-H, 4-H, NHCH), 5.13 (s, 2H, Ar-CH_2), 5.21 (d, J = 3.9 Hz, 1H, OH), 7.29 – 7.41 (m, 6H, CONH, Ar-H); ^{13}C NMR (126 MHz, DMSO-d_6) δ 13.7 ($CH_2\underline{C}CN$), 16.6, 16.7 ($\underline{C}H_2CCN$), 25.9 ($C(\underline{C}H_3)_3$), 36.0 ($\underline{C}(CH_3)_3$), 37.1 (C-3), 56.2, 57.2, 57.9, 66.0, 68.7 (C-2, C-4, C-5, NHCH, Ar-CH_2), 120.0 (CN), 127.9 (C-2′, C-6′), 128.0 (C-4′), 128.3 (C-3′, C-5′), 135.7 (C-1′), 164.5, 169.1, 171.4 (CO).

*2,5-Dichloro-4-(4-methylthiazol-5-yl)benzoic Acid (**48**).*

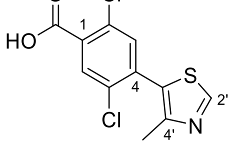

^{1}H NMR (500 MHz, DMSO-d_6) δ 2.27 (s, 3H, CH_3), 7.73 (s, 1H, Ar-H), 7.99 (s, 1H, Ar-H), 9.16 (s, 1H, 2′-H), 13.81 (br s, 1H, OH); ^{13}C NMR (126 MHz, DMSO-d_6) δ 15.5 (CH_3), 125.3, 130.3, 131.4, 132.1, 133.0, 134.2, 134.3 (C-1, C-2, C-3, C-4, C-5, C-5′, C-6), 151.3 (C-4′), 154.0 (C-2′), 165.1 (CO_2H).

*2,5-Dichloro-N-methoxy-N-methyl-4-(4-methylthiazol-5-yl)benzamide (**49**).*

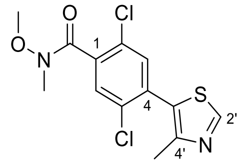

^{1}H NMR (500 MHz, DMSO-d_6) δ 2.27 (s, 3H, CH_3), 3.31 (s, 3H, CH_3), 3.53 (s, 3H, CH_3), 7.72 (s, 1H, Ar-H), 7.83 (s, 1H, Ar-H), 9.16 (s, 1H, 2′-H); ^{13}C NMR (126 MHz, DMSO-d_6) δ 15.5 (CH_3), 31.8 (NCH_3), 61.3 (OCH_3), 125.6, 128.2, 128.6, 132.1, 132.2, 132.9, 137.1 (C-1, C-2, C-3, C-4, C-5, C-5′, C-6), 151.1 (C-4′), 153.8 (C-2′), 165.1 (CO).

*2,5-Dichloro-4-(4-methylthiazol-5-yl)benzaldehyde (**50**).*

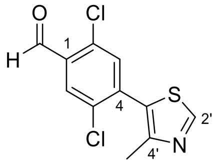

^{1}H NMR (500 MHz, DMSO-d_6) δ 2.29 (s, 3H, CH_3), 7.85 (d, J = 0.9 Hz, 1H, Ar-H), 8.00 (d, J = 1.0 Hz, 1H, Ar-H), 9.20 (d, J = 0.9 Hz, 1H, 2′-H), 10.28 (d, J = 1.0 Hz, 1H, CHO); ^{13}C NMR (126 MHz, DMSO-d_6) δ 15.6 (CH_3), 125.2, 130.4, 132.9, 133.1, 134.1, 134.5, 137.0 (C-1, C-2, C-3, C-4, C-5, C-5′, C-6), 151.5 (C-4′), 154.3 (C-2′), 188.4 (CHO).

*2-Fluoro-3-(4-methylthiazol-5-yl)phenol (**52**).*

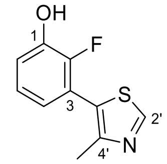

^{1}H NMR (500 MHz, DMSO-d_6) δ 2.33 (d, J = 1.2 Hz, 3H, CH_3), 6.84 (ddd, J = 7.8, 6.3, 1.9 Hz, 1H, Ar-H), 6.99 – 7.10 (m, 2H, Ar-H), 9.08 (s, 1H, 2′-H), 10.07 (br s, 1H, OH); ^{13}C NMR (126 MHz, DMSO-d_6) δ 15.7 (d, $^{5}J_{F,C}$ = 2.7 Hz, CH_3), 118.0 (d, $^{3}J_{F,C}$ = 3.2 Hz, C-6), 119.7 (d, $^{2}J_{F,C}$ = 12.6 Hz, C-3), 121.3, 124.1, 124.4 (d, $^{3}J_{F,C}$ = 4.5 Hz, C-4, C-5, C-5′), 145.6 (d, $^{2}J_{F,C}$ = 12.4 Hz, C-1), 148.2 (d, $^{1}J_{F,C}$ = 243.6 Hz, C-2), 150.0 (C-4′), 152.9 (C-2′).

*3-Fluoro-2-hydroxy-4-(4-methylthiazol-5-yl)benzaldehyde (**53**).*

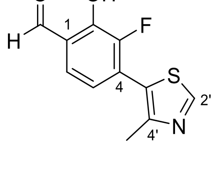

^{1}H NMR (500 MHz, DMSO-d_6) δ 2.39 (s, 3H, CH_3), 7.07 (dd, J = 8.2, 6.3 Hz, 1H, Ar-H), 7.56 (dd, J = 8.2, 1.3 Hz, 1H, Ar-H), 9.18 (s, 1H, 2′-H), 10.30 (s, 1H, CHO), 11.12 (s, 1H, OH); ^{13}C NMR (126 MHz,

DMSO-d_6) δ 16.0 (d, $^5J_{F,C}$ = 2.9 Hz, CH_3), 121.3, 123.1 (C-5′, C-6), 123.8 (d, $^3J_{F,C}$ = 4.0 Hz, C-5), 124.6 (d, $^3J_{F,C}$ = 2.8 Hz, C-1), 125.5 (d, $^2J_{F,C}$ = 12.5 Hz, C-4), 148.6 (d, $^1J_{F,C}$ = 245.0 Hz, C-3), 148.7 (d, $^2J_{F,C}$ = 15.0 Hz, C-2), 151.2 (C-4′), 154.2 (C-2′), 190.3 (d, $^4J_{F,C}$ = 3.1 Hz, CHO).

*1-(4-Bromo-5-fluoro-2-methylphenyl)ethan-1-one (**55c**).*

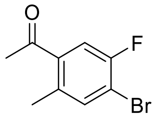

^{1}H NMR (400 MHz, $CDCl_3$) δ, 2.47 (s, 3H, CH_3), 2.56 (s, 3H, CH_3), 7.43 (d, J = 7.7 Hz, 1H, Ar-H), 7.45 (d, J = 7.7 Hz, 1H, Ar-H); ^{13}C NMR (101 MHz, $CDCl_3$) δ 20.7 (CH_3), 29.4 (CH_3), 112.6 (d, $^2J_{F,C}$ = 20.5 Hz, C-4), 116.9 (d, $^2J_{F,C}$ = 22.9 Hz, C-6), 135.6 (d, $^3J_{F,C}$ = 4.0 Hz), 136.7, 137.6 (d, $^3J_{F,C}$ = 4.7 Hz, C-1, C-2, C-3), 156.9 (d, $^1J_{F,C}$ = 246.7 Hz, C-5), 199.3 (d, $^4J_{F,C}$ = 1.9 Hz, CO).

*1-(4-Bromo-5-fluoro-2-methoxyphenyl)ethan-1-one (**55d**)*

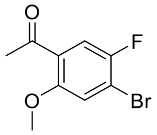

^{1}H NMR (400 MHz, $CDCl_3$) δ 2.60 (s, 3H, CH_3), 3.91 (s, 3H, OCH_3), 7.15 (d, J = 5.2 Hz, 1H, Ar-H), 7.54 (d, J = 8.8 Hz, 1H, Ar-H); ^{13}C NMR (101 MHz, $CDCl_3$) δ 31.8 (CH_3), 56.4 (OCH_3), 114.3 (d, $^2J_{F,C}$ = 23.0 Hz), 116.8, 117.3 (d, $^2J_{F,C}$ = 24.9 Hz, C-3, C-4, C-6), 128.0 (d, $^3J_{F,C}$ = 4.6 Hz, C-1), 153.5 (d, $^1J_{F,C}$ = 241.7 Hz, C-5), 155.2 (d, $^4J_{F,C}$ = 2.2 Hz, C-2), 197.2 (CO).

*(R,E)-N-(1-(4-Bromo-2-methylphenyl)ethylidene)-2-methylpropane-2-sulfinamide (**56a**).*

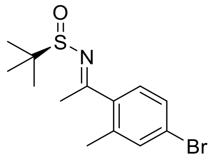

^{1}H NMR (500 MHz, DMSO-d_6) δ 1.19 (s, 9H, $C(CH_3)_3$), 2.35 (s, 3H, CH_3), 2.62 (s, 3H, CH_3), 7.41 – 7.55 (m, 3H, Ar-H); ^{13}C NMR (126 MHz, DMSO-d_6) δ 19.8 (CH_3), 21.8 ($C(\underline{C}H_3)_3$), 23.7 (CH_3), 56.1 ($\underline{C}(CH_3)_3$), 122.6 (C-4), 128.7, 129.2, 133.4, 137.3 (C-1, C-3, C-5, C-6), 139.8 (C-2), 180.7 (NC).

*(R,E)-N-(1-(4-Bromo-3-fluorophenyl)ethylidene)-2-methylpropane-2-sulfinamide (**56b**).*

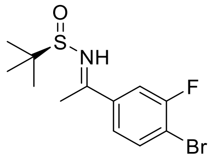

^{1}H NMR (500 MHz, DMSO-d_6) δ 1.22 (s, 9H, $C(CH_3)_3$), 2.71 (s, 3H, CH_3), 5.28 (s, 1H, NH), 7.69 (dd, J = 8.4, 2.1 Hz, 1H, Ar-H), 7.78 – 7.87 (m, 2H, Ar-H); ^{13}C NMR (126 MHz, DMSO-d_6) δ 22.0 ($C(\underline{C}H_3)_3$),

22.2 (CH_3), 57.1 (<u>C</u>(CH_3)$_3$), 111.9 (d, $^2J_{F,C}$ = 21.4 Hz, C-4), 115.0 (d, $^2J_{F,C}$ = 23.8 Hz, C-2), 124.5 (d, $^4J_{F,C}$ = 3.2 Hz, C-6), 133.7 (C-5), 139.9 (d, $^3J_{F,C}$ = 6.3 Hz, C-1), 158.2 (d, $^1J_{F,C}$ = 245.4 Hz, C-3), 174.5 (NC).

*(R,E)-N-(1-(4-Bromo-5-fluoro-2-methylphenyl)ethylidene)-2-methylpropane-2-sulfinamide (**56c**).*

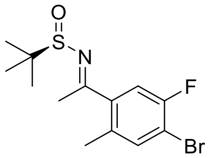

^{1}H NMR (400 MHz, $CDCl_3$) δ 1.23 (s, 9H, C(CH_3)$_3$), 2.36 (s, 3H, CH_3), 2.67 (s, 3H, CH_3), 7.08 (d, J = 9.0 Hz, 1H, Ar-H), 7.42 (d, J = 6.9 Hz, 1H, Ar-H). ^{13}C NMR (101 MHz, $CDCl_3$) δ 22.1 (CH_3), 22.4 (C(<u>C</u>H_3)$_3$), 23.9 (CH_3), 57.2 (<u>C</u>(CH_3)$_3$), 109.9 (d, $^2J_{F,C}$ = 20.7 Hz, C-4), 115.1 (d, $^2J_{F,C}$ = 23.4 Hz, C-6), 132.3 (d, $^3J_{F,C}$ = 3.7 Hz, C-1), 135.9 (C-2), 141.5 (d, $^3J_{F,C}$ = 5.9 Hz, C-3), 157.1 (d, $^1J_{F,C}$ = 246.8 Hz, C-5), 179.1 (NC).

*(R,E)-N-(1-(4-Bromo-5-fluoro-2-methoxyphenyl)ethylidene)-2-methylpropane-2-sulfinamide (**56d**).*

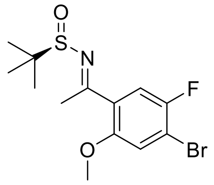

^{1}H NMR (400 MHz, $CDCl_3$) δ 1.30 (s, 9H, C(CH_3)$_3$), 2.69 (s, 3H, CH_3), 3.85 (s, 3H, OCH_3), 7.09 (d, J = 5.4 Hz, 1H, Ar-H), 7.29 – 7.24 (m, 1H, Ar-H); ^{13}C NMR (101 MHz, $CDCl_3$) δ 22.1 (C(<u>C</u>H_3)$_3$), 31.8 (CH_3), 55.39 (OCH_3), 56.39 (<u>C</u>(CH_3)$_3$), 114.28 (d, $^2J_{F,C}$ = 22.9 Hz, C-4), 116.77 (d, $^3J_{F,C}$ = 9.3 Hz, C-1), 117.3 (d, $^2J_{F,C}$ = 24.8 Hz, C-6), 117.4 (d, $^3J_{F,C}$ = 8.3 Hz, C-3), 146.6 (d, $^1J_{F,C}$ = 240.2 Hz, C-5), 155.2 (d, $^4J_{F,C}$ = 2.2 Hz, C-2), 197.2 (NC).

*(R,E)-N-(5-Bromo-2,3-dihydro-1H-inden-1-ylidene)-2-methylpropane-2-sulfinamide (**56e**).*

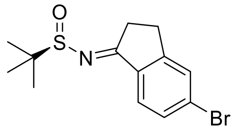

^{1}H NMR (500 MHz, DMSO-d_6) δ 1.22 (s, 9H, C(CH_3)$_3$), 3.00 (ddd, J = 19.3, 6.8, 4.7 Hz, 1H, CH_2), 3.08 – 3.15 (m, 2H, CH_2), 3.25 – 3.33 (m, 1H, CH_2), 7.57 (dd, J = 8.2, 1.7 Hz, 1H, Ar-H), 7.64 (d, J = 8.3 Hz, 1H, Ar-H), 7.77 (d, J = 1.7 Hz, 1H, Ar-H); ^{13}C NMR (126 MHz, DMSO-d_6) δ 22.0 (C(<u>C</u>H_3)$_3$), 28.4 (CH_2), 31.5 (CH_2), 56.7 (<u>C</u>(CH_3)$_3$), 124.5, 127.2, 129.1, 130.4 (C-4, C-5, C-6, C-7), 137.7 (C-7a), 153.2 (C-3a), 182.5 (NC).

*(R,E)-N-(5-Bromo-6-fluoro-2,3-dihydro-1H-inden-1-ylidene)-2-methylpropane-2-sulfinamide (**56f**).*

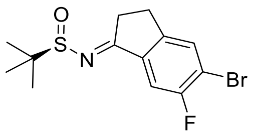

^{1}H NMR (400 MHz, DMSO) δ 1.23 (s, 9H, C(CH_3)$_3$), 3.15 – 2.96 (m, 3H, CH_2), 3.31 – 3.25 (m, 1H, CH_2), 7.59 (d, *J* = 8.1 Hz, 1H, Ar-H) 7.93 (dd, *J* = 6.2, 1.0 Hz, 1H, Ar-H); ^{13}C NMR (101 MHz, $CDCl_3$) δ 22.5 (C($\underline{C}H_3$)$_3$), 28.5 (CH_2), 32.2 (CH_2), 57.7 ($\underline{C}$(CH_3)$_3$), 110.2 (d, $^2J_{F,C}$ = 23.6 Hz, C-5), 115.5 (d, $^2J_{F,C}$ = 22.8 Hz, C-7), 130.7 (C-4), 140.3 (d, $^3J_{F,C}$ = 7.6 Hz, C-7a), 146.7 (d, $^4J_{F,C}$ = 2.8 Hz, C-3a), 158.6 (d, $^1J_{F,C}$ = 248.0 Hz, C-6), 181.6 (NC).

*(R,E)-N-(6-Bromo-7-fluoro-3,4-dihydronaphthalen-1(2H)-ylidene)-2-methylpropane-2-sulfinamide (**56g**).*

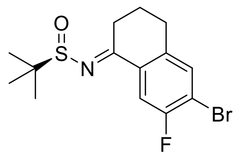

^{1}H NMR (400 MHz, $CDCl_3$) δ 1.33 (s, 9H, C(CH_3)$_3$), 2.12 – 1.87 (m, 2H, CH_2), 2.82 (t, *J* = 6.0 Hz, 2H, CH_2), 2.99 – 3.11 (m, 1H, CH_2), 3.20 – 3.32 (m, 1H, CH_2), 7.42 (dd, *J* = 6.6, 1.0 Hz, 1H, Ar-H), 7.84 (d, *J* = 9.7 Hz, 1H, Ar-H); ^{13}C NMR (101 MHz, $CDCl_3$) δ 24.9 (C($\underline{C}H_3$)$_3$), 25.2 (CH_2), 27.3 (CH_2), 31.1 (CH_2), 53.7 ($\underline{C}$(CH_3)$_3$), 112.2 (d, $^2J_{F,C}$ = 21.6 Hz, C-6), 114.7 (d, $^2J_{F,C}$ = 22.3 Hz, C-8), 126.4 (d, $^4J_{F,C}$ = 2.3 Hz, C-4a), 131.5 (C-5), 137.6 (d, $^3J_{F,C}$ = 7.6 Hz, C-8a), 163.5 (d, $J_{F,C}$ = 246.0 Hz, C-7), 179.1 (NC).

*(R)-N-((S)-1-(4-Bromo-2-methylphenyl)ethyl)-2-methylpropane-2-sulfinamide (**57a**).*

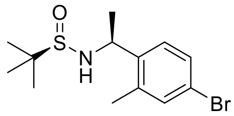

^{1}H NMR (500 MHz, DMSO-d_6) δ 1.09 (s, 9H, C(CH_3)$_3$), 1.41 (d, *J* = 6.7 Hz, 3H, CH_3), 2.31 (s, 3H, CH_3), 4.51 – 4.60 (m, 1H, NHC$\underline{H}$), 5.30 (d, *J* = 5.2 Hz, 1H, NH), 7.30 – 7.39 (m, 3H, Ar-H); ^{13}C NMR (126 MHz, DMSO-d_6) δ 18.3 (CH_3), 22.6 (C($\underline{C}H_3$)$_3$), 23.4 (CH_3), 50.4 (NHCH), 54.9 ($\underline{C}$(CH_3)$_3$), 119.5 (C-4), 128.6, 128.7, 132.3, 137.5 (C-2, C-3, C-5, C-6), 142.0 (C-1).

*(R)-N-((S)-1-(4-Bromo-3-fluorophenyl)ethyl)-2-methylpropane-2-sulfinamide (**57b**).*

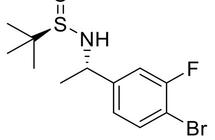

^{1}H NMR (500 MHz, DMSO-d_6) δ 1.10 (s, 9H, C(CH_3)$_3$), 1.44 (d, *J* = 6.8 Hz, 3H, CH_3), 4.39 – 4.47 (m, 1H, CH), 5.46 (d, *J* = 5.5 Hz, 1H, NH), 7.16 (dd, *J* = 8.4, 2.0 Hz, 1H, Ar-H), 7.33 – 7.38 (m, 1H, Ar-H), 7.65 (t, *J* = 7.8 Hz, 1H, Ar-H); ^{13}C NMR (126 MHz, DMSO-d_6) δ 22.5 (C($\underline{C}H_3$)$_3$), 24.4 (CH_3), 54.0

(CH), 55.0 (C(CH$_3$)$_3$), 105.8 (d, $^2J_{F,C}$ = 20.8 Hz, C-4), 114.8 (d, $^2J_{F,C}$ = 22.5 Hz, C-2), 124.3 (C-6), 133.1 (C-5), 147.7 (d, $^3J_{F,C}$ = 6.2 Hz, C-1), 158.0 (d, $^1J_{F,C}$ = 244.4 Hz, C-3).

*(R)-N-((S)-1-(4-Bromo-5-fluoro-2-methylphenyl)ethyl)-2-methylpropane-2-sulfinamide (**57c**).*

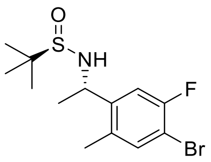

^{1}H NMR (400 MHz, CDCl$_3$) δ 1.22 (s, 9H, C(CH$_3$)$_3$), 1.46 (d, *J* = 6.6 Hz, 3H, CH$_3$), 2.33 (t, *J* = 0.9 Hz, 3H, CH$_3$), 3.29 (d, *J* = 3.0 Hz, 1H, NH), 4.75 (qdd, *J* = 6.6, 3.0, 1.7 Hz, 1H, CH), 7.13 (d, *J* = 9.9 Hz, 1H, Ar-H), 7.33 (dd, *J* = 7.0, 0.7 Hz, 1H, Ar-H); ^{13}C NMR (101 MHz, CDCl$_3$) δ 18.3 (CH$_3$), 22.5 (C(CH$_3$)$_3$), 24.0 (CH$_3$), 49.7 (CH), 55.7 (C(CH$_3$)$_3$), 107.1 (d, $^2J_{F,C}$ = 20.8 Hz, C-4), 114.2 (d, $^2J_{F,C}$ = 22.9 Hz, C-6), 132.7 (d, $^3J_{F,C}$ = 3.6 Hz, C-3), 134.9 (C-2), 143.0 (d, $^3J_{F,C}$ = 5.6 Hz, C-1), 157.8 (d, $^1J_{F,C}$ = 244.7 Hz, C-5).

*(R)-N-((S)-1-(4-Bromo-5-fluoro-2-methoxyphenyl)ethyl)-2-methylpropane-2-sulfinamide (**57d**).*

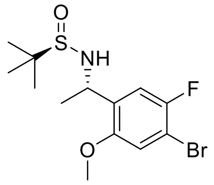

^{1}H NMR (400 MHz, CDCl$_3$) δ 1.21 (s, 9H, C(CH$_3$)$_3$), 1.47 (d, *J* = 6.7 Hz, 3H, CH$_3$), 3.42 (d, *J* = 4.5 Hz, 1H, NH), 3.82 (s, 3H, OCH$_3$), 4.92 – 4.80 (m, 1H, CH), 6.99 (d, *J* = 5.6 Hz, 1H, Ar-H), 7.09 (dd, *J* = 9.2, 0.5 Hz, 1H, Ar-H); ^{13}C NMR (101 MHz, CDCl$_3$) δ 22.6 (C(CH$_3$)$_3$), 23.5 (CH$_3$), 49.1 (CH), 55.7 (OCH$_3$), 56.2 (C(CH$_3$)$_3$), 106.9 (d, $^2J_{F,C}$ = 22.5 Hz, C-4), 114.8 (d, $^2J_{F,C}$ = 24.8 Hz, C-6), 115.3 (C-3), 133.4 (d, $^3J_{F,C}$ = 5.4 Hz, C-1), 153.1 (C-2), 153.6 (d, $^1J_{F,C}$ = 239.9 Hz, C-5).

*(R)-N-((S)-5-Bromo-2,3-dihydro-1H-inden-1-yl)-2-methylpropane-2-sulfinamide (**57e**).*

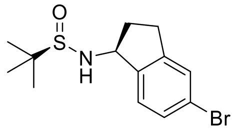

^{1}H NMR (500 MHz, DMSO-*d*$_6$) δ 1.16 (s, 9H, C(CH$_3$)$_3$), 1.92 – 2.01 (m, 1H, CH$_2$), 2.39 – 2.47 (m, 1H, CH$_2$), 2.72 – 2.81 (m, 1H, CH$_2$), 2.91 (ddd, *J* = 16.2, 8.8, 3.2 Hz, 1H, CH$_2$), 4.61 – 4.68 (m, 1H, 1-H), 5.62 (d, *J* = 9.0 Hz, 1H, NH), 7.21 (d, *J* = 8.0 Hz, 1H), 7.38 (dd, *J* = 8.0, 1.8 Hz, 1H), 7.44 (d, *J* = 1.8 Hz, 1H, Ar-H); ^{13}C NMR (126 MHz, DMSO-*d*$_6$) δ 22.8 (C(CH$_3$)$_3$), 29.5 (CH$_2$), 35.2 (CH$_2$), 55.3 (C(CH$_3$)$_3$), 60.8 (NHCH), 120.4 (C-5), 126.3, 127.3, 129.0 (C-4, C-6, C-7), 144.1 (C-7a), 145.6 (C-3a).

*(R)-N-((S)-5-Bromo-6-fluoro-2,3-dihydro-1H-inden-1-yl)-2-methylpropane-2-sulfinamide (**57f**).*

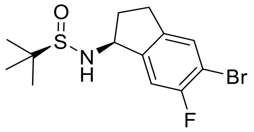

^{1}H NMR (400 MHz, $CDCl_3$) δ 1.27 (s, 9H, $C(CH_3)_3$), 1.87 – 2.13 (m, 1H, CH_2), 2.63 – 2.87 (m, 2H, CH_2), 2.88 – 3.02 (m, 1H, CH_2), 3.33 (d, J = 9.8 Hz, 1H, NH), 4.67 – 4.95 (m, 1H, CH), 7.07 (dd, J = 8.2, 1.1 Hz, 1H, Ar-H), 7.41 (dd, J = 6.3, 1.2 Hz, 1H, Ar-H). ^{13}C NMR (101 MHz, $CDCl_3$) δ 22.8 ($C(CH_3)_3$), 29.5 (CH_2), 37.1 (CH_2), 56.2 ($\underline{C}(CH_3)_3$), 62.0 (C-1), 108.7 (d, $^{2}J_{F,C}$ = 22.1 Hz, C-5), 112.3 (d, $^{2}J_{F,C}$ = 23.3 Hz, C-7), 129.4 (C-4), 139.7 (d, $^{4}J_{F,C}$ = 3.0 Hz, C-3a), 145.3 (d, $^{3}J_{F,C}$ = 6.6 Hz, C-7a), 158.1 (d, $^{1}J_{F,C}$ = 245.7 Hz, C-6).

*(R)-N-((S)-6-Bromo-7-fluoro-1,2,3,4-tetrahydronaphthalen-1-yl)-2-methylpropane-2-sulfinamide (**57g**).*

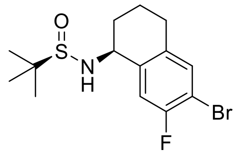

^{1}H NMR (400 MHz, $CDCl_3$) δ 1.28 (s, 9H, $C(CH_3)_3$), 1.98 – 1.77 (m, 3H, CH_2), 2.32 – 2.38 (m, 1H, CH_2), 2.82 – 2.62 (m, 2H, CH_2), 3.35 (d, J = 10.1 Hz, 1H, NH), 4.46 – 4.30 (m, 1H, CH), 7.16 (dd, J = 9.7, 0.9 Hz, 1H, Ar-H), 7.29 (d, J = 1.0 Hz, 1H, Ar-H); ^{13}C NMR (101 MHz, $CDCl_3$) δ 20.1 (CH_2), 22.8 ($C(\underline{C}H_3)_3$), 28.2 (CH_2), 32.8 (CH_2), 55.6 (C-1), 56.6 ($\underline{C}(CH_3)_3$), 107.8 (d, $^{2}J_{F,C}$ = 21.3 Hz, C-6), 116.0 (d, $^{2}J_{F,C}$ = 22.3 Hz, C-8), 133.6 (C-4a), 134.7 (d, $^{3}J_{F,C}$ = 3.7 Hz, C-5), 139.0 (d, $^{3}J_{F,C}$ = 5.6 Hz, C-8a), 157.2 (d, $^{1}J_{F,C}$ = 245.2 Hz, C-7).

*(S)-1-(4-Bromo-2-methylphenyl)ethan-1-aminium Chloride (**58a**).*

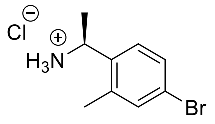

^{1}H NMR (500 MHz, DMSO-d_6) δ 1.45 (d, J = 6.8 Hz, 3H, CH_3), 2.35 (s, 3H, CH_3), 4.50 (q, J = 6.8 Hz, 1H, CH), 7.45 – 7.53 (m, 3H, Ar-H), 8.44 (s, 3H, NH_3); ^{13}C NMR (126 MHz, DMSO-d_6) δ 18.4 (CH_3), 20.0 (CH_3), 45.7 (CH), 121.1 (C-4), 127.5, 129.2, 132.9, 137.1, 138.2 (C-1, C-2, C-3, C-5, C-6).

*(S)-1-(4-Bromo-3-fluorophenyl)ethan-1-aminium Chloride (**58b**).*

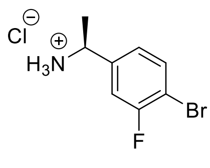

^{1}H NMR (600 MHz, DMSO-d_6) δ 1.50 (d, J = 6.8 Hz, 3H, CH_3), 4.44 (q, J = 6.8 Hz, 1H, CH), 7.33 (dd, J = 8.3, 2.1 Hz, 1H, Ar-H), 7.60 (dd, J = 10.1, 2.1 Hz, 1H, Ar-H), 7.79 (t, J = 7.8 Hz, 1H, Ar-H), 8.58

(s, 3H, NH_3); 13C NMR (151 MHz, DMSO-d6) δ 20.3 (CH_3), 49.0 (CH), 107.8 (d, $^2J_{F,C}$ = 20.8 Hz, C-4), 115.5 (d, $^2J_{F,C}$ = 23.4 Hz, C-2), 124.7 (d, $^4J_{F,C}$ = 3.7 Hz, C-6), 133.8 (C-5), 141.6 (d, $^3J_{F,C}$ = 7.0 Hz, C-1), 158.1 (d, $^1J_{F,C}$ = 244.9 Hz, C-3).

*(S)-5-Bromo-2,3-dihydro-1H-inden-1-aminium Chloride (**58e**).*

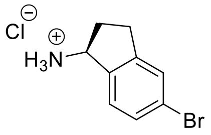

^{1}H NMR (500 MHz, DMSO-d_6) δ 1.94 – 2.08 (m, 1H, CH_2), 2.40 – 2.48 (m, 1H, CH_2), 2.83 – 2.94 (m, 1H, CH_2), 3.02 – 3.12 (m, 1H, CH_2), 4.66 (dd, J = 7.9, 5.5 Hz, 1H, 1-H), 7.49 (d, J = 7.6 Hz, 1H, Ar-H), 7.53 – 7.61 (m, 2H, Ar-H), 8.53 (s, 3H, NH_3); ^{13}C NMR (126 MHz, DMSO-d_6) δ 29.7 (CH_2), 30.3 (CH_2), 54.0 (C-1), 122.2 (C-5), 126.9, 127.8, 129.5 (C-4, C-6, C-7), 138.7 (C-7a), 146.9 (C-3a).

*tert-Butyl (S)-(1-(4-Bromo-2-methylphenyl)ethyl)carbamate (**59a**).*

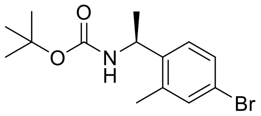

^{1}H NMR (500 MHz, DMSO-d_6) δ 1.22 (d, J = 6.9 Hz, 3H, CH_3), 1.34 (s, 9H, $C(CH_3)_3$), 2.30 (s, 3H, CH_3), 4.70 – 4.78 (m, 1H, CH), 7.26 (d, J = 8.3 Hz, 1H), 7.31 – 7.39 (m, 2H), 7.45 (d, J = 7.9 Hz, 1H) (Ar-H, NH); ^{13}C NMR (126 MHz, DMSO-d_6) δ 18.1 (CH_3), 21.4 (CH_3), 28.2 ($C(\underline{C}H_3)_3$), 45.6 (CH), 77.7 ($\underline{C}(CH_3)_3$), 119.0 (C-4), 127.1, 128.8, 132.1, 136.9 (C-2, C-3, C-5, C-6), 143.4 (C-1), 154.6 (CO).

*tert-Butyl (S)-(1-(4-Bromo-3-fluorophenyl)ethyl)carbamate (**59b**).*

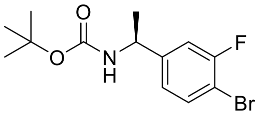

^{1}H NMR (500 MHz, DMSO-d_6) δ 1.28 (d, J = 7.0 Hz, 3H, CH_3), 1.36 (s, 9H, $C(\underline{C}H_3)_3$), 4.57 – 4.65 (m, 1H, CH), 7.10 (dd, J = 8.2, 1.9 Hz, 1H), 7.24 – 7.30 (m, 1H), 7.43 (d, J = 8.0 Hz, 1H), 7.63 (t, J = 7.8 Hz, 1H) (Ar-H, NH); ^{13}C NMR (126 MHz, DMSO-d_6) δ 22.4 (CH_3), 28.1 ($C(\underline{C}H_3)_3$), 48.9 (CH), 77.9 ($\underline{C}(CH_3)_3$), 105.4 (d, $^2J_{F,C}$ = 20.7 Hz, C-4), 114.0 (d, $^2J_{F,C}$ = 22.4 Hz, C-2), 123.5 (d, $^4J_{F,C}$ = 3.2 Hz, C-6), 133.1 (C-5), 148.2 (d, $^3J_{F,C}$ = 7.9 Hz, C-1), 154.7 (CO), 158.1 (d, $^1J_{F,C}$ = 244.4 Hz, C-3).

*tert-Butyl (S)-(1-(4-Bromo-5-fluoro-2-methylphenyl)ethyl)carbamate (**59c**).*

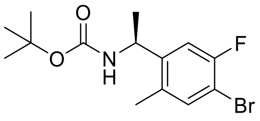

^{1}H NMR (400 MHz, $CDCl_3$) δ 1.35 (d, J = 6.7 Hz, 3H, CH_3). 1.41 (s, 9H, $C(CH_3)_3$), 2.32 (s, 3H, CH_3), 4.74 – 4.90 (m, 2H, CH, NH), 7.04 (d, J = 9.8 Hz, 1H, Ar-H), 7.31 (dd, J = 7.0, 0.8 Hz, 1H, Ar-H); ^{13}C

NMR (101 MHz, $CDCl_3$) δ 18.1 (CH_3), 21.7 (CH_3), 27.4 ($C(\underline{C}H_3)_3$), 31.2 (CH), 79.8 ($\underline{C}(CH_3)_3$), 106.6 (d, $^2J_{F,C}$ = 21.5 Hz, C-6), 112.5 (d, $^2J_{F,C}$ = 22.8 Hz, C-4), 132.2 (C-2), 135.0 (C-3), 146.8 (C-1), 154.8 (CO), 157.8 (d, $^1J_{F,C}$ = 244.6 Hz, C-5).

*tert-Butyl (S)-(1-(4-Bromo-5-fluoro-2-methoxyphenyl)ethyl)carbamate (**59d**).*

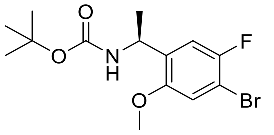

^{1}H NMR (400 MHz, $CDCl_3$) δ 1.36 (d, J = 6.9 Hz, 3H, CH_3), 1.42 (s, 9H, $C(CH_3)_3$), 3.83 (s, 3H, OCH_3), 4.86 – 4.97 (m, 1H, CH), 5.02 – 5.14 (m, 1H, NH), 7.04 – 6.93 (m, 2H, Ar-H); ^{13}C NMR (101 MHz, $CDCl_3$) δ 21.4 (CH_3), 28.4 ($C(\underline{C}H_3)_3$), 46.8 (CH), 56.1 (OCH_3), 79.6 ($\underline{C}(CH_3)_3$), 106.5 (d, $^2J_{F,C}$ = 22.7 Hz, C-4), 114.4 (d, $^2J_{F,C}$ = 24.4 Hz, C-6), 115.4 (d, $^3J_{F,C}$ = 9.01 Hz, C-3), 133.7 (C-1), 153.0 (d, $^4J_{F,C}$ = 2.4 Hz, C-2), 153.6 (d, $^1J_{F,C}$ = 240.3 Hz, C-5), 154.9 (CO).

*tert-Butyl (S)-(5-Bromo-2,3-dihydro-1H-inden-1-yl)carbamate (**59e**).*

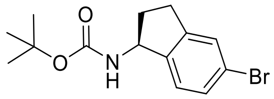

^{1}H NMR (500 MHz, DMSO-d_6) δ 1.42 (s, 9H, $C(CH_3)_3$), 1.76 – 1.86 (m, 1H, CH_2), 2.28 – 2.37 (m, 1H, CH_2), 2.70 – 2.79 (m, 1H, CH_2), 2.89 (ddd, J = 16.1, 8.7, 3.1 Hz, 1H, CH_2), 4.89 – 4.96 (m, 1H, 1-H), 7.12 (d, J = 8.1 Hz, 1H), 7.26 (d, J = 8.5 Hz, 1H), 7.35 (d, J = 8.0 Hz, 1H), 7.41 (d, J = 1.8 Hz, 1H) (Ar-H, NH); ^{13}C NMR (126 MHz, DMSO-d_6) δ 28.2 ($C(\underline{C}H_3)_3$), 29.3 (CH_2), 32.5 (CH_2), 54.6 (C-1), 77.8 ($\underline{C}(CH_3)_3$), 120.2 (C-5), 125.6, 127.3, 129.1 (C-4, C-6, C-7), 144.1 (C-7a), 145.6 (C-3a), 155.5 (CO).

*tert-Butyl (S)-(5-Bromo-6-fluoro-2,3-dihydro-1H-inden-1-yl)carbamate (**59f**).*

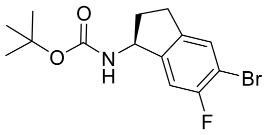

^{1}H NMR (400 MHz, $CDCl_3$) δ 1.49 (s, 9H, $C(CH_3)_3$), 1.77 – 1.85 (m, 1H, CH_2), 2.66 – 2.49 (m, 1H, CH_2), 3.06 – 2.65 (m, 2H, CH_2), 4.73 (d, J = 8.9 Hz, 1H, NH), 5.13 (q, J = 8.2 Hz, 1H, 1-H), 7.22 – 7.01 (m, 1H, Ar-H), 7.32 – 7.44 (m, 1H, Ar-H); ^{13}C NMR (101 MHz, $CDCl_3$) δ 28.4 ($C(\underline{C}H_3)_3$), 29.3 (CH_2), 34.7 (CH_2), 55.6 (C-1), 79.9 ($\underline{C}(CH_3)_3$), 108.3 (d, $^2J_{F,C}$ = 22.0 Hz, C-5), 112.1 (d, $^2J_{F,C}$ = 23.2 Hz, C-7), 129.2 (C-4), 139.8 (d, $^3J_{F,C}$ = 3.0 Hz, C-7a), 145.9 (C-3a), 155.5 (CO), 158.2 (d, $^1J_{F,C}$ = 245.7 Hz, C-6).

*tert-Butyl (S)-(6-Bromo-7-fluoro-1,2,3,4-tetrahydronaphthalen-1-yl)carbamate (**59g**).*

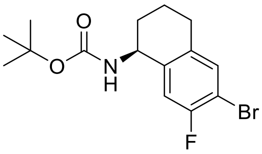

^{1}H NMR (400 MHz, $CDCl_3$) δ 1.48 (s, 9H, $C(CH_3)_3$), 1.75 – 1.65 (m, 1H, CH_2), 1.85 – 1.75 (m, 2H, CH_2), 2.11 – 1.99 (m, 1H, CH_2), 2.71 (q, J = 6.8 Hz, 2H, CH_2), 4.81 – 4.69 (m, 2H, 1-H, NH), 7.12 (d, J = 9.5 Hz, 1H, Ar-H), 7.25 (d, J = 1.0 Hz, 1H, Ar-H); ^{13}C NMR (101 MHz, $CDCl_3$) δ 20.2 (CH_2), 28.4 ($C(\underline{C}H_3)_3$), 28.5 (CH_2), 30.2 (CH_2), 48.5 (C-1), 79.8 ($\underline{C}(CH_3)_3$), 107.5 (d, $^{2}J_{F,C}$ = 20.9 Hz, C-6), 115.8 (d, $^{2}J_{F,C}$ = 22.2 Hz, C-8), 133.5 (C-4a), 134.5 (d, $^{3}J_{F,C}$ = 3.9 Hz, C-5), 139.0 (C-8a), 155.5 (CO), 157.4 (d, $^{1}J_{F,C}$ = 245.4 Hz, C-7).

*tert-Butyl (S)-(1-(2-Methyl-4-(4-methylthiazol-5-yl)phenyl)ethyl)carbamate (**60a**).*

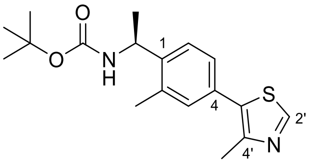

^{1}H NMR (600 MHz, DMSO-d_6) δ 1.27 (d, J = 7.0 Hz, 3H, CH_3), 1.36 (s, 9H, $C(CH_3)_3$), 2.36 (s, 3H, CH_3), 2.45 (s, 3H, CH_3), 4.79 – 4.86 (m, 1H, CH), 7.23 (d, J = 1.9 Hz, 1H), 7.30 (dd, J = 8.1, 2.0 Hz, 1H), 7.42 (d, J = 8.0 Hz, 1H), 7.47 (d, J = 8.0 Hz, 1H) (Ar-H, NH), 8.96 (s, 1H, 2′-H); ^{13}C NMR (151 MHz, DMSO-d_6) δ 16.0 (CH_3), 18.5 (CH_3), 21.7 (CH_3), 28.3 ($C(\underline{C}H_3)_3$), 45.8 (CH), 77.7 ($\underline{C}(CH_3)_3$), 125.5, 126.6, 129.4, 130.4, 131.2, 134.8 (C-2, C-3, C-4, C-5, C-5′, C-6), 143.8 (C-1), 147.6 (C-4′), 151.3 (C-2′), 154.7 (CO).

*tert-Butyl (S)-(1-(3-Fluoro-4-(4-methylthiazol-5-yl)phenyl)ethyl)carbamate (**60b**).*

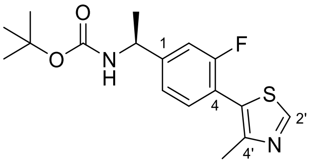

^{1}H NMR (600 MHz, DMSO-d_6) δ 1.33 (s, 3H, CH_3), 1.38 (s, 9H, $C(CH_3)_3$), 2.33 (s, 3H, CH_3), 4.65 – 4.72 (m, 1H, CH), 7.23 (dd, J = 8.0, 1.6 Hz, 1H), 7.27 (d, J = 11.5 Hz, 1H), 7.42 – 7.49 (m, 2H) (Ar-H, NH), 9.09 (s, 1H, 2′-H); ^{13}C NMR (151 MHz, DMSO-d_6) δ 15.7 (CH_3), 22.6 (CH_3), 28.2 ($C(\underline{C}H_3)_3$) , 49.1 (CH), 77.9 ($\underline{C}(CH_3)_3$), 113.3 (d, $^{2}J_{F,C}$ = 22.8 Hz, C-2), 116.8 (d, $^{2}J_{F,C}$ = 15.7 Hz, C-4), 122.2 (d, $^{4}J_{F,C}$ = 2.2 Hz, C-6), 123.8, 131.9 (C-5, C-5′), 149.1 (C-1), 150.1 (C-4′), 153.1 (C-2′), 154.8 (CO), 158.8 (d, $^{2}J_{F,C}$ = 246.5 Hz, C-3).

*tert-Butyl (S)-(1-(5-Fluoro-2-methyl-4-(4-methylthiazol-5-yl)phenyl)ethyl)carbamate (**60c**).*

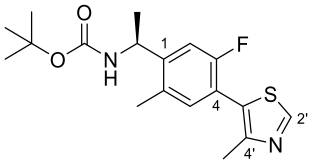

^{1}H NMR (400 MHz, $CDCl_3$) δ 1.39 – 1.49 (m, 12H, $C(CH_3)_3$, CH_3), 2.37 (s, 3H, CH_3), 2.43 (d, J = 1.4 Hz, 3H, CH_3), 4.75 – 4.89 (m, 1H, NH), 4.90 – 5.00 (m, 1H, CH), 7.03 – 7.21 (m, 2H, Ar-H), 8.76 (s, 1H, 2′-H); ^{13}C NMR (101 MHz, $CDCl_3$) δ 16.0 (CH_3), 18.2 (CH_3), 21.8 (CH_3), 28.4 ($C(\underline{C}H_3)_3$), 30.0 (CH), 79.9 ($\underline{C}(CH_3)_3$), 112.1 (d, $^{2}J_{F,C}$ = 23.3 Hz, C-6), 124.6, 128.0 128.4, 130.9, 133.9 (C-1, C-2, C-3, C-4, C-5′), 150.78 (C-4′), 151.6 (C-2′), 154.9 (CO), 158.5 (d, $^{1}J_{F,C}$ = 247.5 Hz, C-5).

*tert-Butyl (S)-(1-(5-Fluoro-2-methoxy-4-(4-methylthiazol-5-yl)phenyl)ethyl)carbamate (**60d**).*

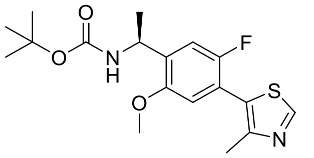

^{1}H NMR (400 MHz, $CDCl_3$) δ 1.33 – 1.52 (m, 12H, $C(CH_3)_3$, CH_3), 2.45 (d, J = 1.4 Hz, 3H, CH_3), 3.85 (s, 3H, OCH_3), 4.91 –5.03 (m, 1H, NH), 5.11 – 5.25 (m, 1H, CH), 6.79 (d, J = 5.9 Hz, 1H, Ar-H), 7.05 (d, J = 10.1 Hz, 1H, Ar-H), 8.77 (s, 1H, 2′-H); ^{13}C NMR (101 MHz, $CDCl_3$) δ 16.1 (CH_3), 21.6 (CH_3), 28.4 ($C(\underline{C}H_3)_3$), 46.9 (CH), 56.0 (OCH_3), 79.6 ($\underline{C}(CH_3)_3$), 113.7 (C-3), 114.4 (d, $^{2}J_{F,C}$ = 24.8 Hz, C-6), 117.7 (d, $^{2}J_{F,C}$ = 16.9 Hz, C-4), 124.7 (C-5′), 134.9 (C-1), 150.9 (C-4′), 151.7 (C-2′), 152.5 (d, $^{4}J_{F,C}$ = 2.2 Hz, C-2), 154.0 (d, $^{1}J_{F,C}$ = 242.1 Hz, C-5), 155.0 (CO).

*tert-Butyl (S)-(5-(4-Methylthiazol-5-yl)-2,3-dihydro-1H-inden-1-yl)carbamate (**60e**).*

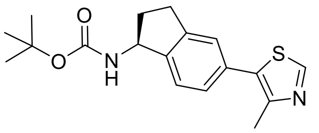

^{1}H NMR (500 MHz, DMSO-d_6) δ 1.44 (s, 9H, $C(CH_3)_3$), 1.79 – 1.90 (m, 1H, CH_2), 2.33 – 2.41 (m, 1H, CH_2), 2.44 (s, 3H, CH_3), 2.75 – 2.85 (m, 1H, CH_2), 2.90 – 2.98 (m, 1H, CH_2), 4.98 – 5.05 (m, 1H, 1-H), 7.21 – 7.35 (m, 4H, Ar-H, NH), 8.97 (s, 1H, 2′-H); ^{13}C NMR (126 MHz, DMSO-d_6) δ 15.9 (CH_3), 28.2 ($C(\underline{C}H_3)_3$), 29.5 (CH_2), 32.6 (CH_2), 54.9 (C-1), 77.8 ($\underline{C}(CH_3)_3$), 124.1 (C-5′), 125.0, 127.3, 130.4, 131.5 (C-4, C-5, C-6, C-7), 143.6 (C-7a), 144.6 (C-3a), 147.6 (C-4′), 151.3 (C-2′), 155.6 (CO).

*tert-Butyl (S)-(6-Fluoro-5-(4-methylthiazol-5-yl)-2,3-dihydro-1H-inden-1-yl)carbamate (**60f**).*

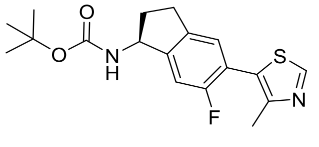

^{1}H NMR (400 MHz, $CDCl_3$) δ 1.50 (s, 9H, $C(CH_3)_3$), 1.81 – 1.89 (m, 1H, CH_2), 2.42 (d, *J* = 1.4 Hz, 3H, CH_3), 2.60 – 2.68 (m, 1H, CH_2), 2.79 – 2.89 (m, 1H, CH_2), 2.91 – 2.99 (m, 1H, CH_2), 4.77 (d, *J* = 8.9 Hz, 1H, NH), 5.22 (q, *J* = 8.2 Hz, 1H, CH), 7.14 (dd, *J* = 9.5, 1.0 Hz, 1H, Ar-H), 7.19 (d, *J* = 6.7 Hz, 1H, Ar-H), 8.76 (s, 1H, 2′-H); ^{13}C NMR (101 MHz, $CDCl_3$) δ 15.9 (CH_3), 28.4 (C($\underline{C}H_3$)$_3$), 29.4 (CH_2), 34.7 (CH_2), 55.8 (C-1), 79.9 ($\underline{C}$(CH_3)$_3$), 111.8 (d, $^2J_{F,C}$ = 23.3 Hz, C-7), 118.9 (d, $^2J_{F,C}$ = 16.9 Hz, C-5), 124.9 (C-5′), 127.8 (d, $^3J_{F,C}$ = 5.7 Hz, C-7a), 138.6 (d, $^4J_{F,C}$ = 2.6 Hz, C-3a), 146.6 (d, $^3J_{F,C}$ = 7.4 Hz, C-4), 150.8 (C-4′), 151.6 (C-2′), 155.6 (CO), 159.1 (d, $^1J_{F,C}$ = 247.5 Hz, C-6).

*tert-Butyl (S)-(7-Fluoro-6-(4-methylthiazol-5-yl)-1,2,3,4-tetrahydronaphthalen-1-yl)carbamate (**60g**).*

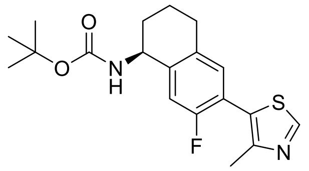

^{1}H NMR (400 MHz, $CDCl_3$) δ 1.50 (s, 9H, $C(CH_3)_3$), 1.71 – 1.79 (m, 1H, CH_2), 1.86 (dd, *J* = 10.9, 5.9 Hz, 2H, CH_2), 2.11 (d, *J* = 8.4 Hz, 1H, CH_2), 2.43 (s, 3H, CH_3), 2.74 – 2.78 (m, 2H, CH_2), 4.79 (d, *J* = 9.2 Hz, 1H, NH), 4.82 – 4.97 (m, 1H, 1-H), 7.07 (d, *J* = 7.4 Hz, 1H, Ar-H), 7.17 (d, *J* = 10.7 Hz, 1H, Ar-H), 8.76 (s, 1H, 2′-H); ^{13}C NMR (101 MHz, $CDCl_3$) δ 16.0 (CH_3), 16.0 (CH_2), 20.3 (CH_2), 28.4 (C($\underline{C}H_3$)$_3$), 30.3 (CH_2), 48.6 (C-1), 79.8 ($\underline{C}$(CH_3)$_3$), 115.3 (d, $^2J_{F,C}$ = 22.3 Hz, C-8), 118.4 (d, $^2J_{F,C}$ = 16.0 Hz, C-6), 124.5 (C-5′), 132.3 (d, $^4J_{F,C}$ = 2.6 Hz, C-4a), 133.1 (d, $^3J_{F,C}$ = 3.5 Hz, C-5), 140.2 (d, $^3J_{F,C}$ = 6.6 Hz, C-8a), 150.8 (C-4′), 151.6 (C-2′), 155.5 (CO), 158.1 (d, $^1J_{F,C}$ = 247.5 Hz, C-7).

*Benzyl (2S,4R)-1-((S)-2-(1-Fluorocyclopropane-1-carboxamido)-3,3-dimethylbutanoyl)-4-hydroxypyrrolidine-2-carboxylate (**61**).*

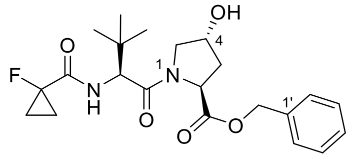

^{1}H NMR (600 MHz, DMSO-*d*$_6$) δ 0.92 (s, 9H, $C(CH_3)_3$), 1.17 – 1.25 (m, 2H, CH_2CF), 1.30 – 1.41 (m, 2H, CH_2CF), 1.90 – 1.97 (m, 1H, 3-H), 2.13 – 2.19 (m, 1H, 3-H), 3.59 – 3.64 (m, 1H, 5-H), 3.67 (dd, *J* = 10.8, 3.9 Hz, 1H, 5-H), 4.32 – 4.36 (m, 1H), 4.46 (dd, *J* = 9.2, 7.8 Hz, 1H), 4.59 (d, *J* = 9.2 Hz, 1H) (2-H, 4-H, NHC$\underline{H}$), 5.13 (dd, *J* = 12.3, 12.1, 2H, Ar-CH_2), 5.23 (d, *J* = 3.9 Hz, 1H, OH), 7.26 (dd, *J* = 9.3, 2.9 Hz, 1H, CONH), 7.31 – 7.39 (m, 5H, Ar-H); ^{13}C NMR (151 MHz, DMSO-*d*$_6$) δ 12.7 (d, $^2J_{F,C}$ = 10.3 Hz, $\underline{C}H_2CF$), 12.9 (d, $^2J_{F,C}$ = 10.2 Hz, $\underline{C}H_2CF$), 26.0 (C($\underline{C}H_3$)$_3$), 35.8 ($\underline{C}$(CH_3)$_3$), 37.2 (C-3), 56.3, 56.4, 57.9, 66.0, 68.8 (C-2, C-4, C-5, NHCH, Ar-CH_2), 78.0 (d, $^1J_{F,C}$ = 232.7 Hz, CF), 127.9 (C-2′, C-6′), 128.0 (C-4′), 128.4 (C-3′, C-5′), 135.8 (C-1′), 168.1 (d, $^2J_{F,C}$ = 20.9 Hz), 169.3, 171.5 (CO).

*3-Bromo-2-methoxybenzaldehyde (**64**).*

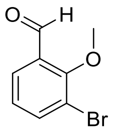

^{1}H NMR (500 MHz, DMSO-*d*$_6$) δ 3.93 (s, 3H, CH_3), 7.27 (t, *J* = 7.8 Hz, 1H, 5-H), 7.76 (dd, *J* = 7.9, 1.7 Hz, 1H, 4-H), 7.97 (dd, *J* = 7.9, 1.6 Hz, 1H, 6-H), 10.24 (s, 1H, CHO); ^{13}C NMR (126 MHz, DMSO-*d*$_6$) δ 63.5 (CH_3), 117.9 (C-3), 126.4 (C-5), 128.3 (C-1), 130.9 (C-6), 139.5 (C-4), 159.3 (C-2), 189.5 (CHO).

*3-Bromo-2-methoxyphenol (**65**).*

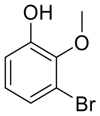

^{1}H NMR (500 MHz, DMSO-*d*$_6$) δ 3.72 (s, 3H, CH_3), 6.79 – 6.89 (m, 2H, 4-H, 6-H), 6.94 – 7.03 (m, 1H, 5-H), 9.74 (s, 1H, OH); ^{13}C NMR (126 MHz, DMSO-*d*$_6$) δ 59.9 (CH_3), 116.6, 116.9 (C-3, C-6), 122.9 (C-5), 125.5 (C-4), 144.9 (C-2), 151.7 (C-1).

*4-Bromo-2-hydroxy-3-methoxybenzaldehyde (**66**).*

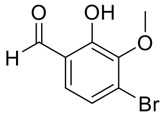

^{1}H NMR (600 MHz, DMSO-*d*$_6$) δ 3.78 (s, 3H, CH_3), 7.21 (d, *J* = 8.5 Hz, 1H, 5-H), 7.37 (d, *J* = 8.4 Hz, 1H, 6-H), 10.19 (s, 1H, OH), 10.87 (br s, 1H, CHO); ^{13}C NMR (151 MHz, DMSO-*d*$_6$) δ 60.6 (CH_3), 123.4, 123.5 (C-1, C-4), 124.5 (C-5), 125.8 (C-6), 146.1 (C-3), 154.6 (C-2), 191.9 (CHO).

*4-Bromo-2,5-dichloro-N-methoxy-N-methylbenzamide (**67**).*

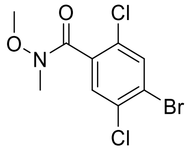

^{1}H NMR (500 MHz, DMSO-*d*$_6$) δ 3.27 (s, 3H, NCH_3), 3.47 (s, 3H, OCH_3), 7.85 (s, 1H, 6-H), 8.04 (s, 1H, 3-H); ^{13}C NMR (126 MHz, DMSO-*d*$_6$) δ 32.0 (NCH_3), 61.4 (OCH_3), 123.0 (C-4), 128.9 (C-6), 129.2 (C-5), 132.4 (C-2), 133.9 (C-1), 136.4 (C-3), 164.9 (CO).

*4-Bromo-2,5-dichlorobenzaldehyde (**68**).*

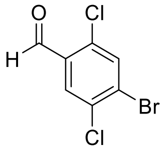

^{1}H NMR (500 MHz, DMSO-d_6) δ 7.96 (s, 1H, 3-H), 8.17 (s, 1H, 6-H), 10.19 (s, 1H, CHO); ^{13}C NMR (126 MHz, DMSO-d_6) δ 128.8 (C-4), 130.6 (C-5), 132.7 (C-6), 133.4 (C-1), 134.8 (C-3), 135.6 (C-2), 188.4 (CO).

*4-Bromo-3-fluoro-2-hydroxybenzaldehyde (**69**).*

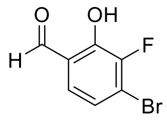

^{1}H NMR (600 MHz, DMSO-d_6) δ 7.25 (dd, J = 8.5, 5.8 Hz, 1H, 5-H), 7.41 (dd, J = 8.5, 1.7 Hz, 1H, 6-H), 10.23 (s, 1H, OH), 11.33 (br s, 1H, CHO); ^{13}C NMR (151 MHz, DMSO-d_6) δ 116.0 (d, $^{2}J_{F,C}$ = 18.7 Hz, C-4), 123.1 (C-1), 123.9 – 125.8 (m, C-5, C-6), 147.3 – 150.8 (m, C-2, C-3), 190.1 (CO).

^{1}H and ^{13}C NMR Spectra

^{1}H and ^{13}C NMR Spectra of compound **2**

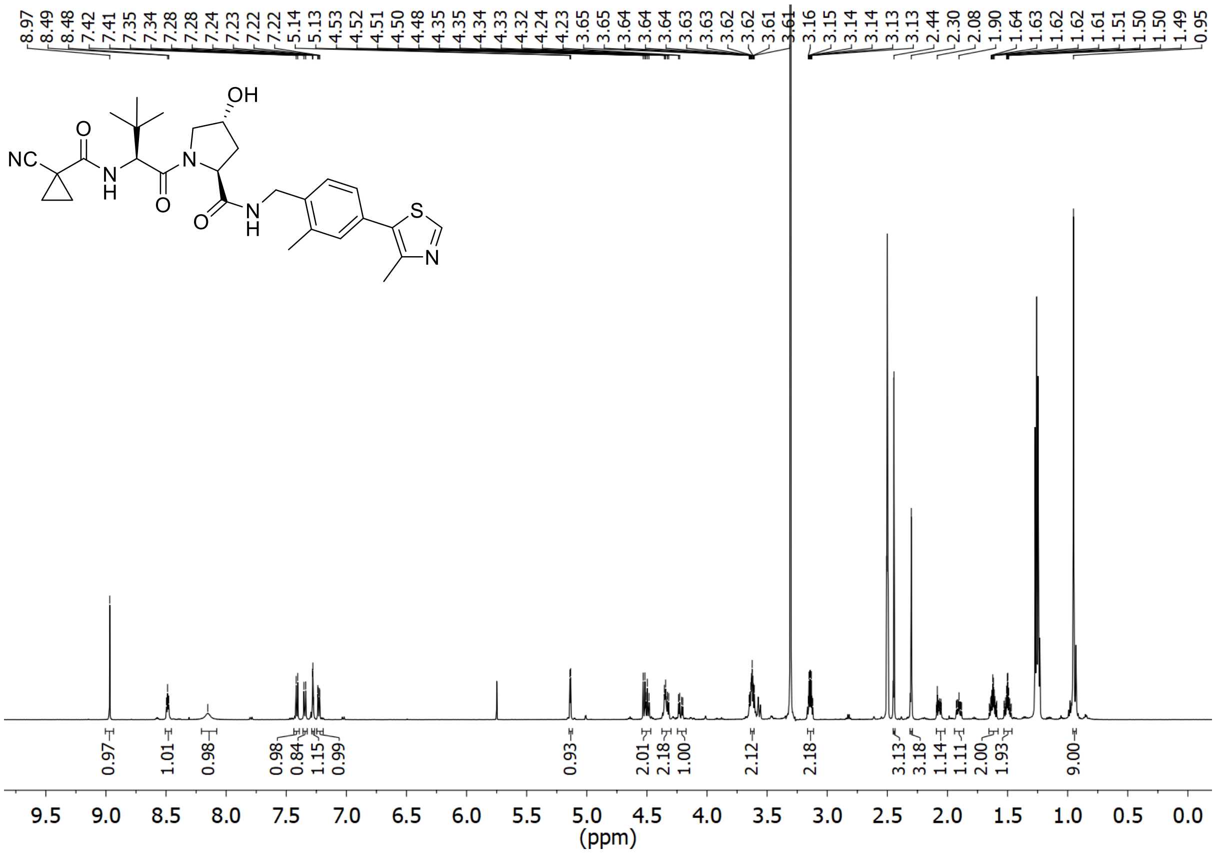

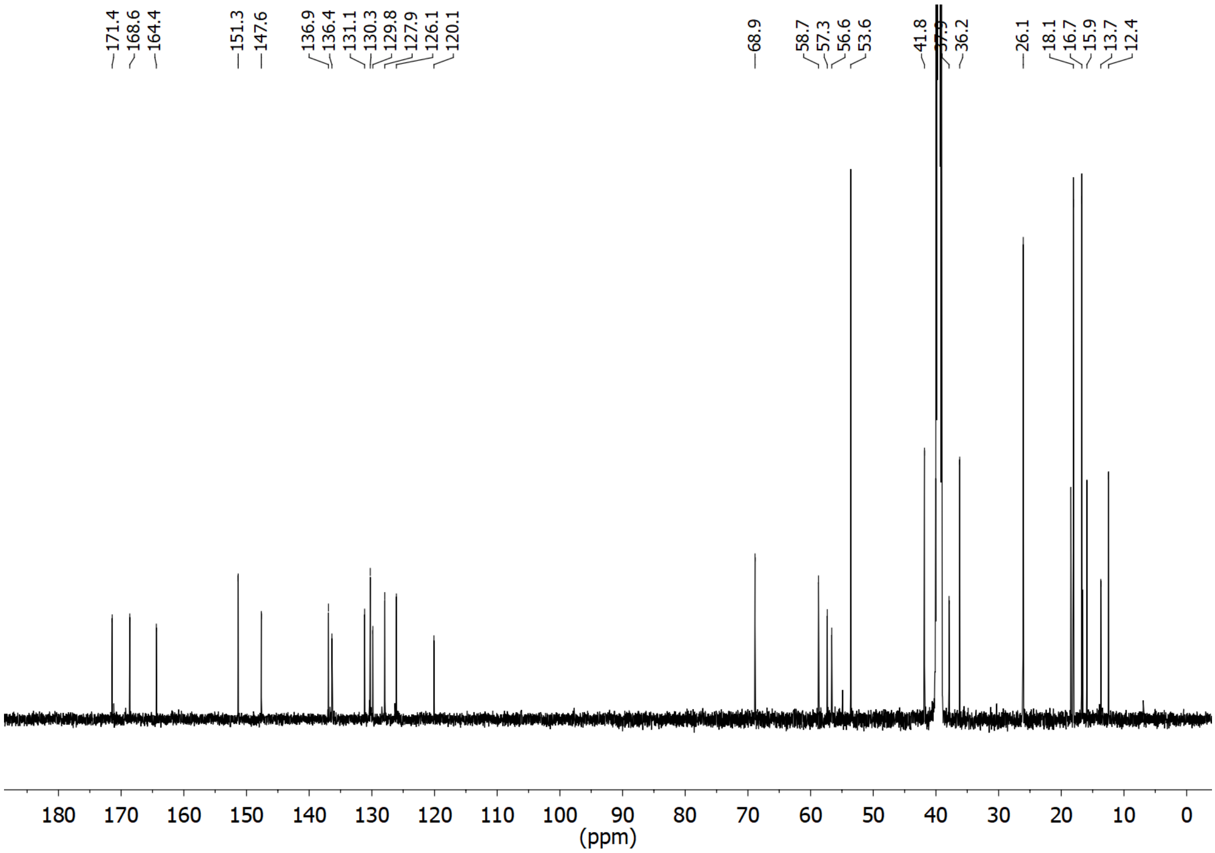

^{1}H and ^{13}C NMR Spectra of compound **3**

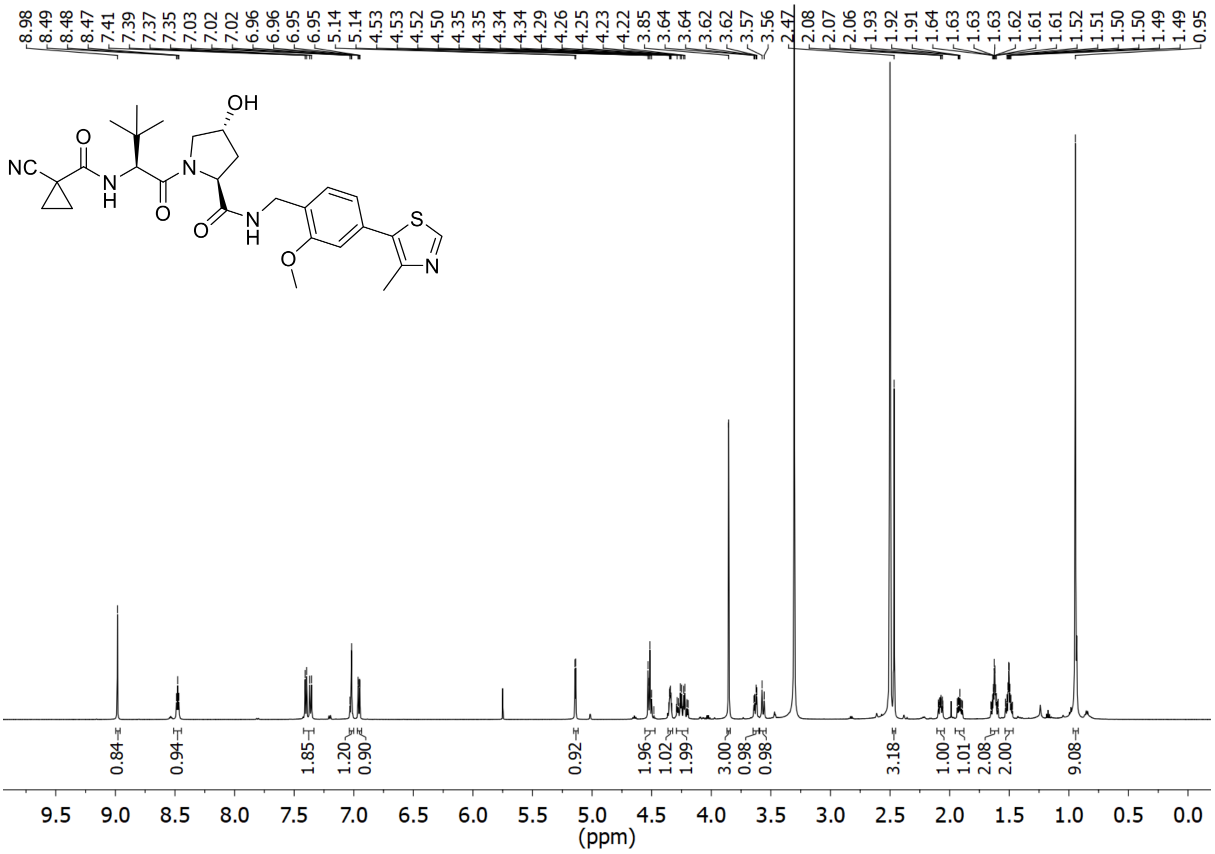

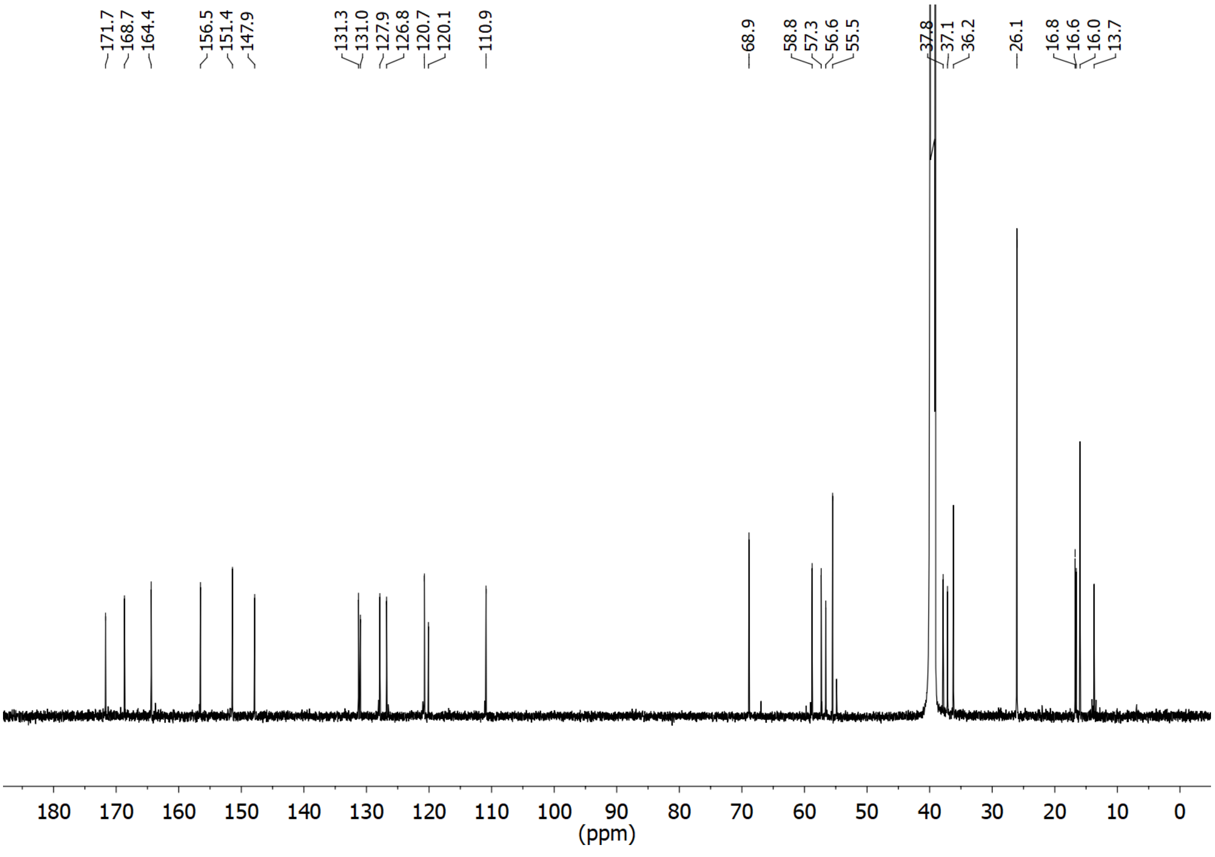

^{1}H and ^{13}C NMR Spectra of compound **4**

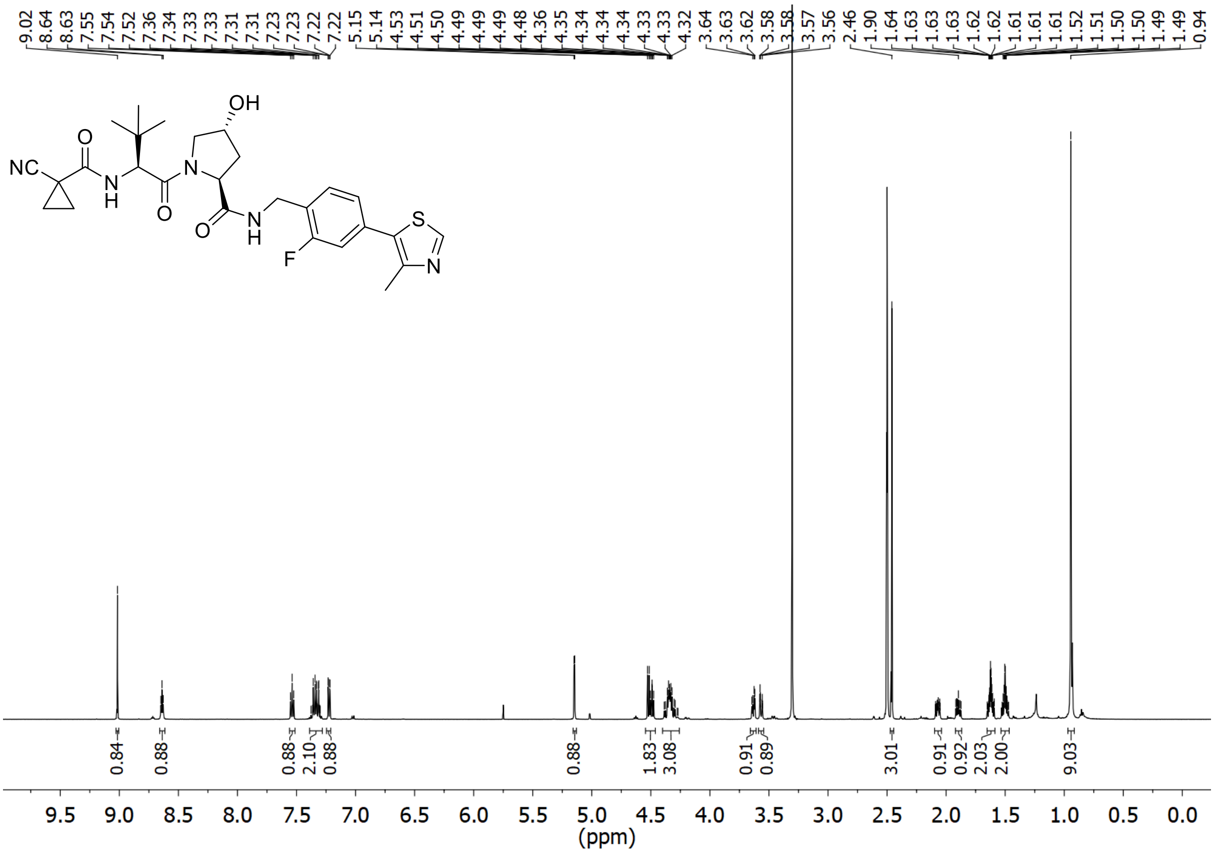

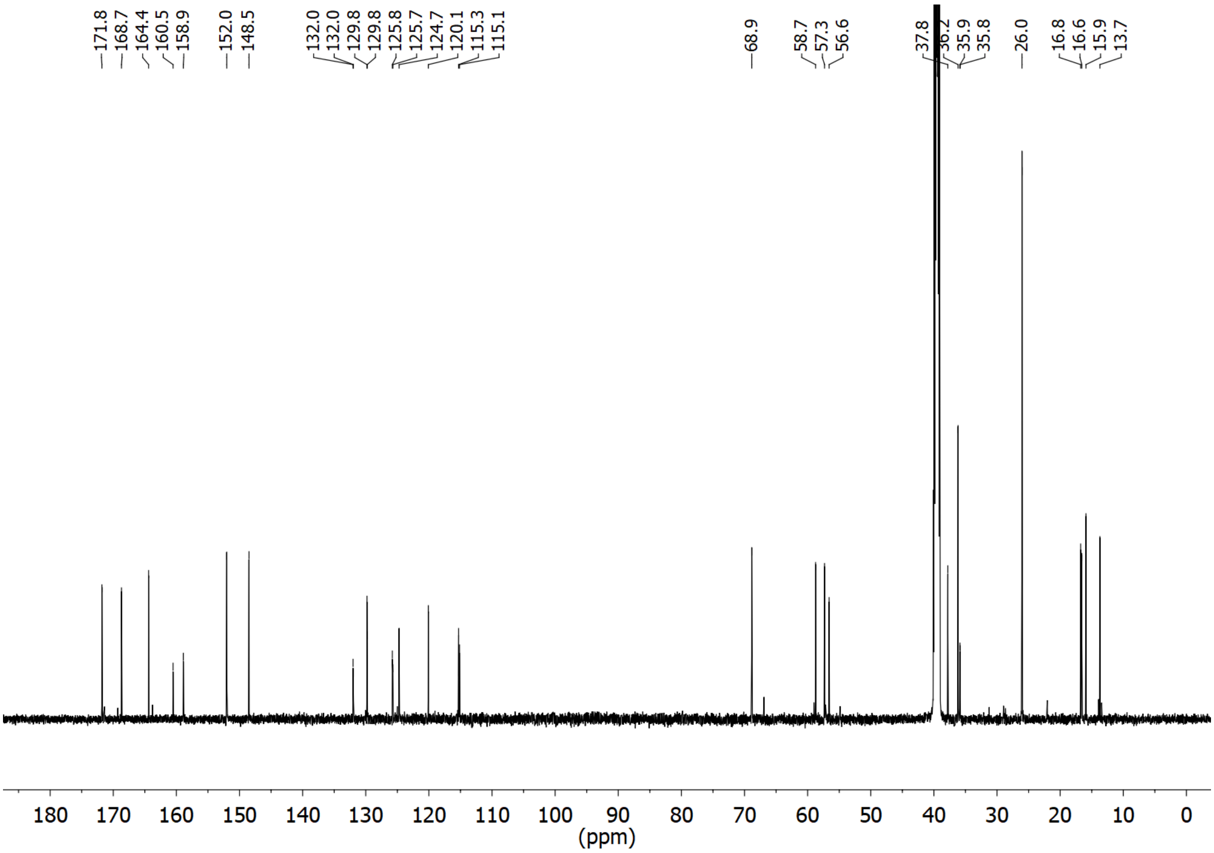

^{1}H and ^{13}C NMR Spectra of compound **5**

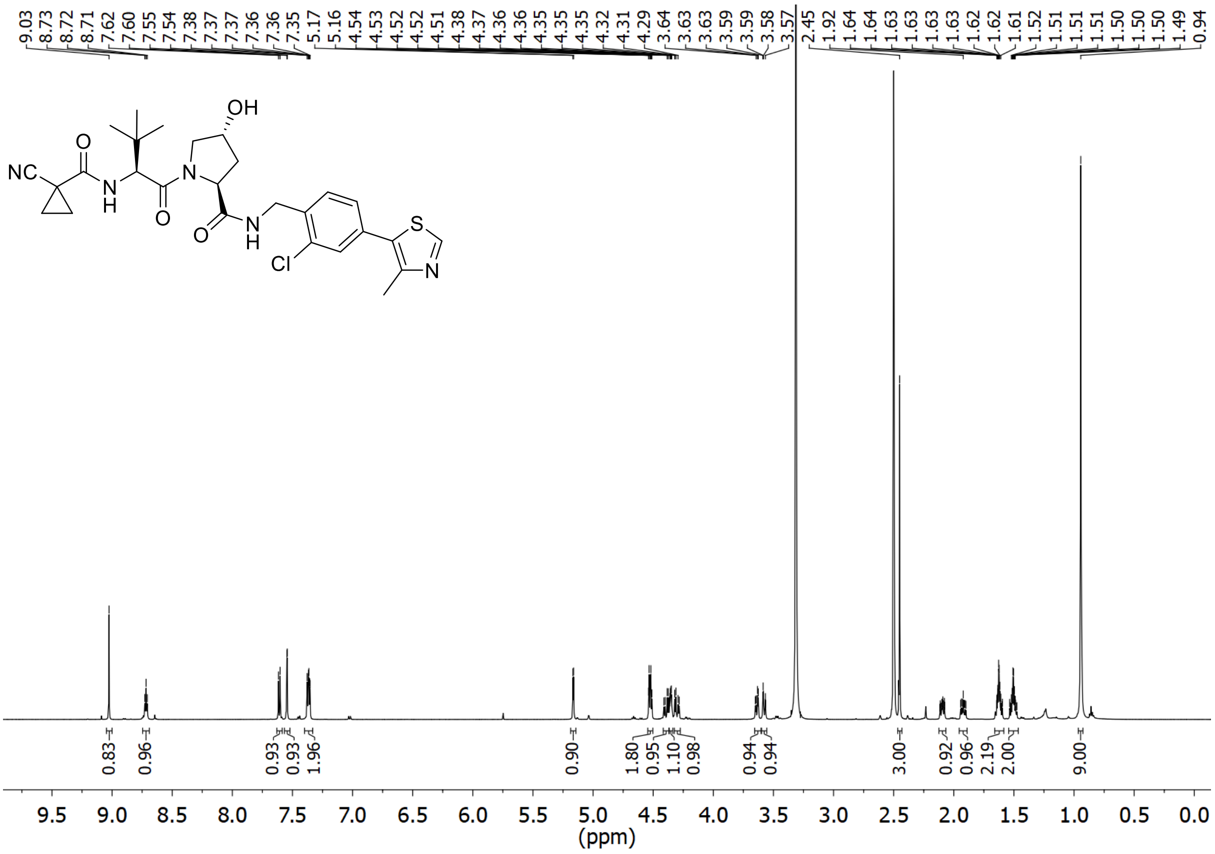

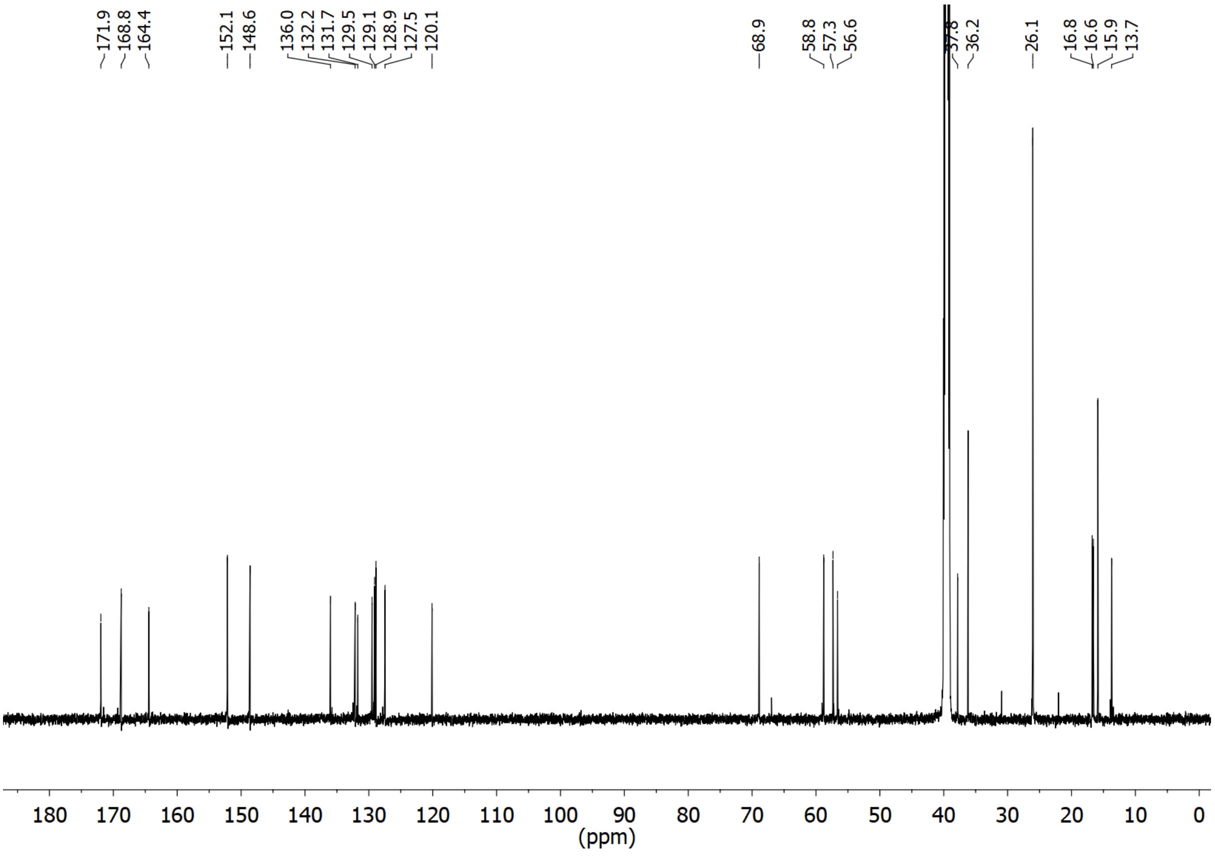

^{1}H and ^{13}C NMR Spectra of compound **6**

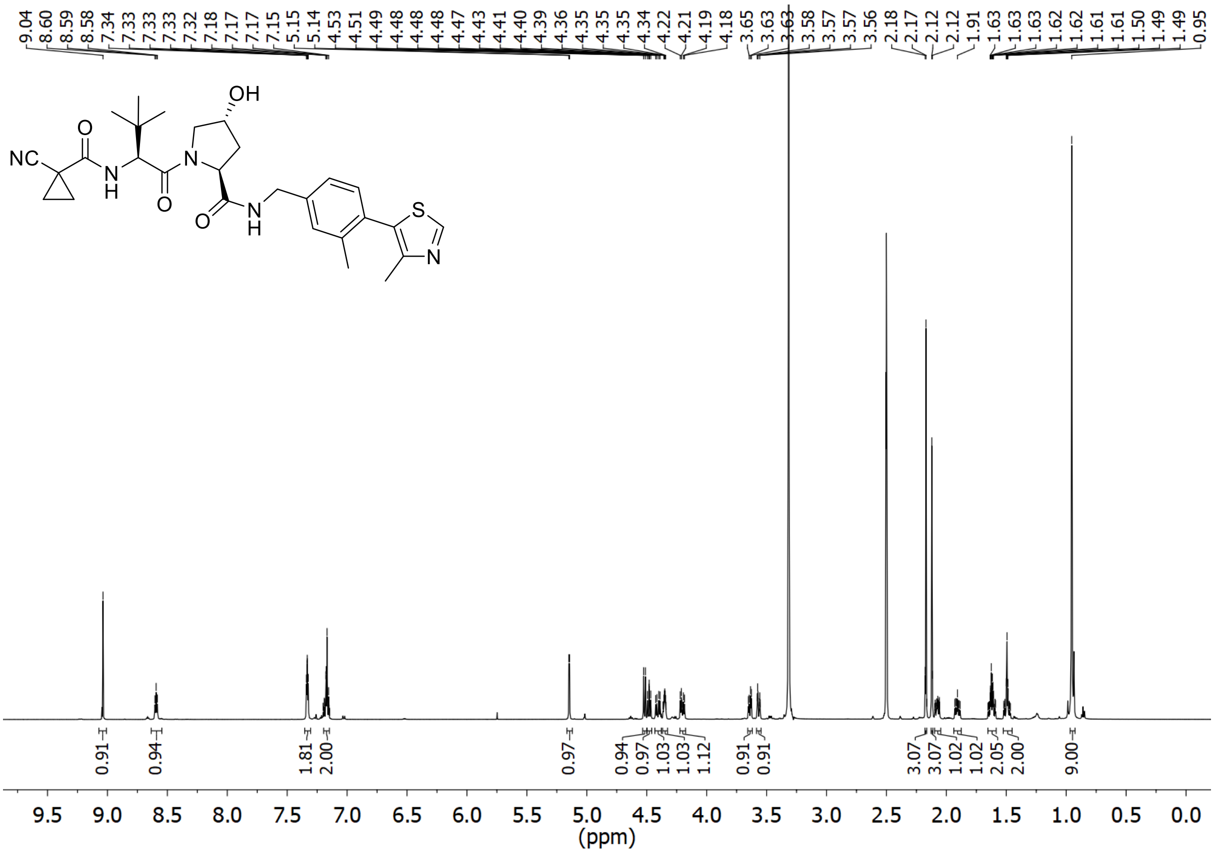

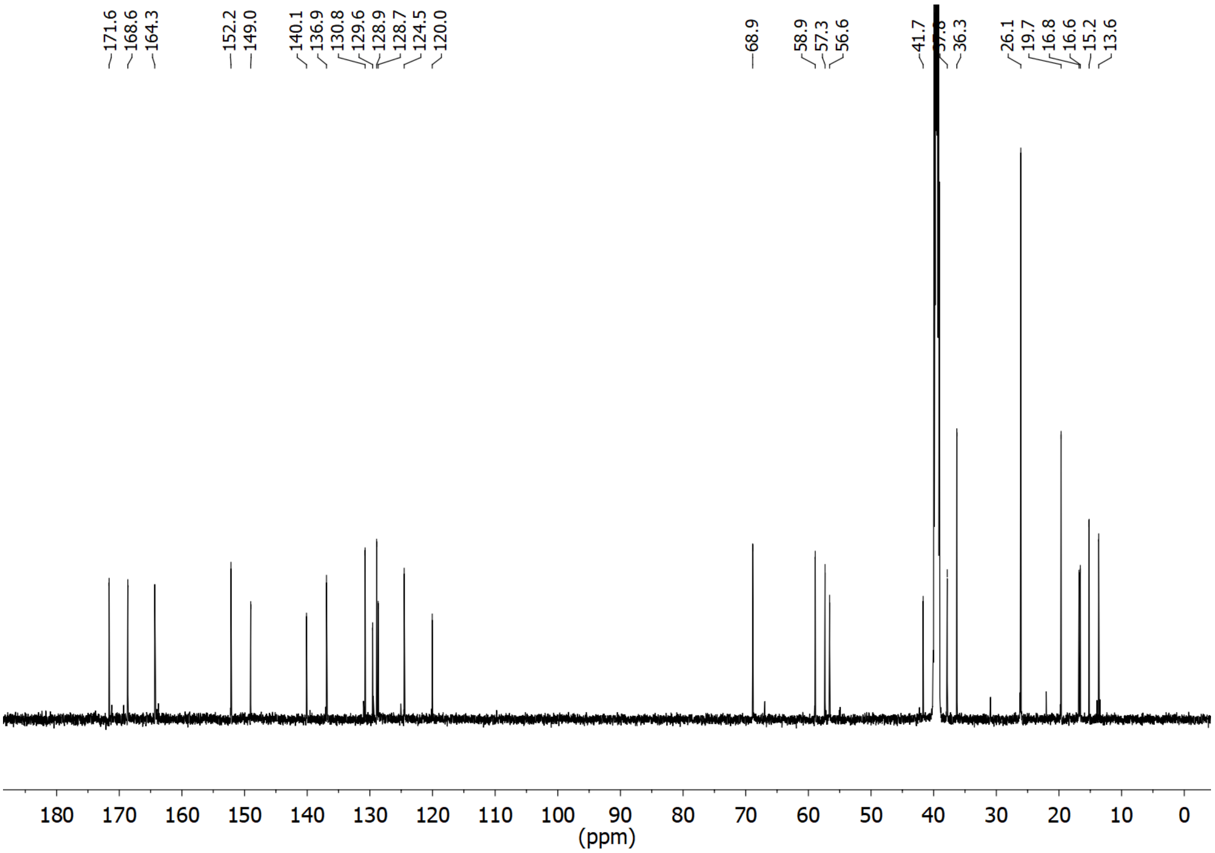

^{1}H and ^{13}C NMR Spectra of compound **7**

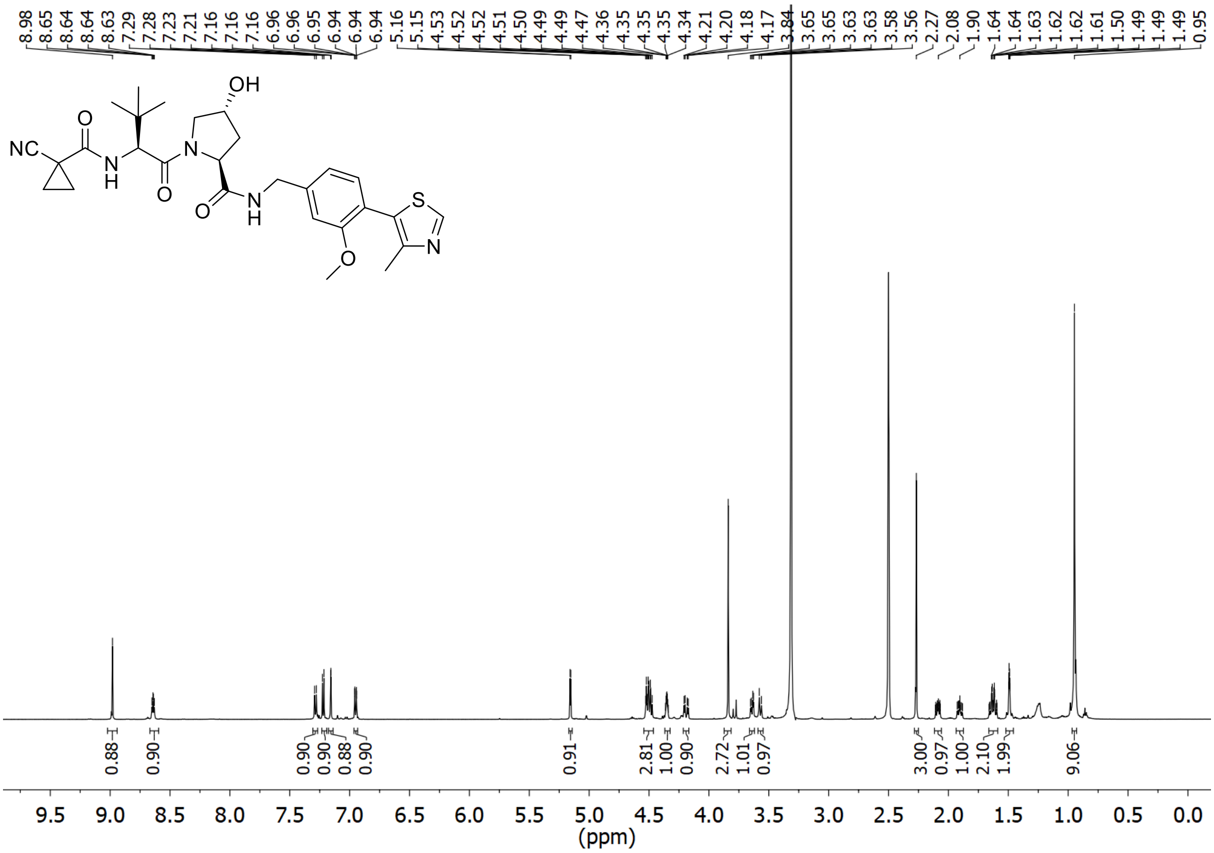

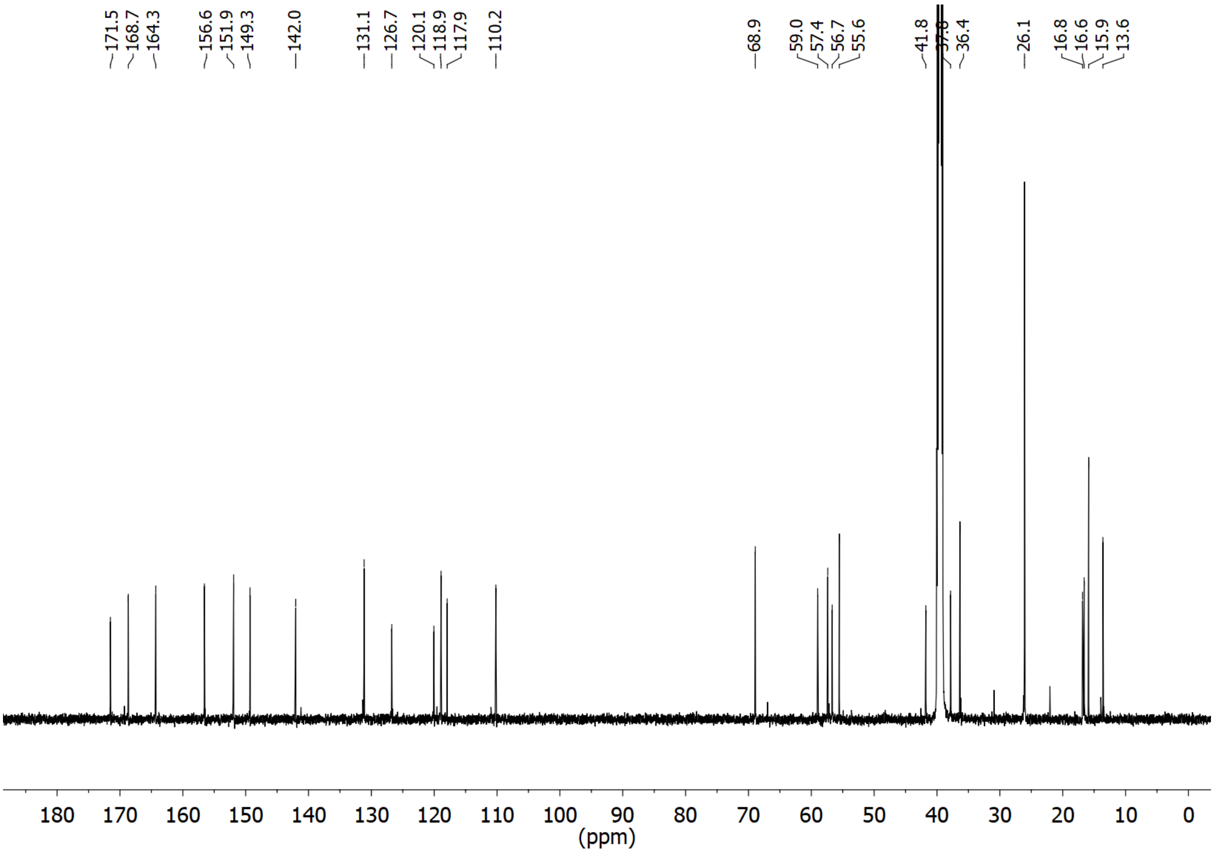

^{1}H and ^{13}C NMR Spectra of compound **8**

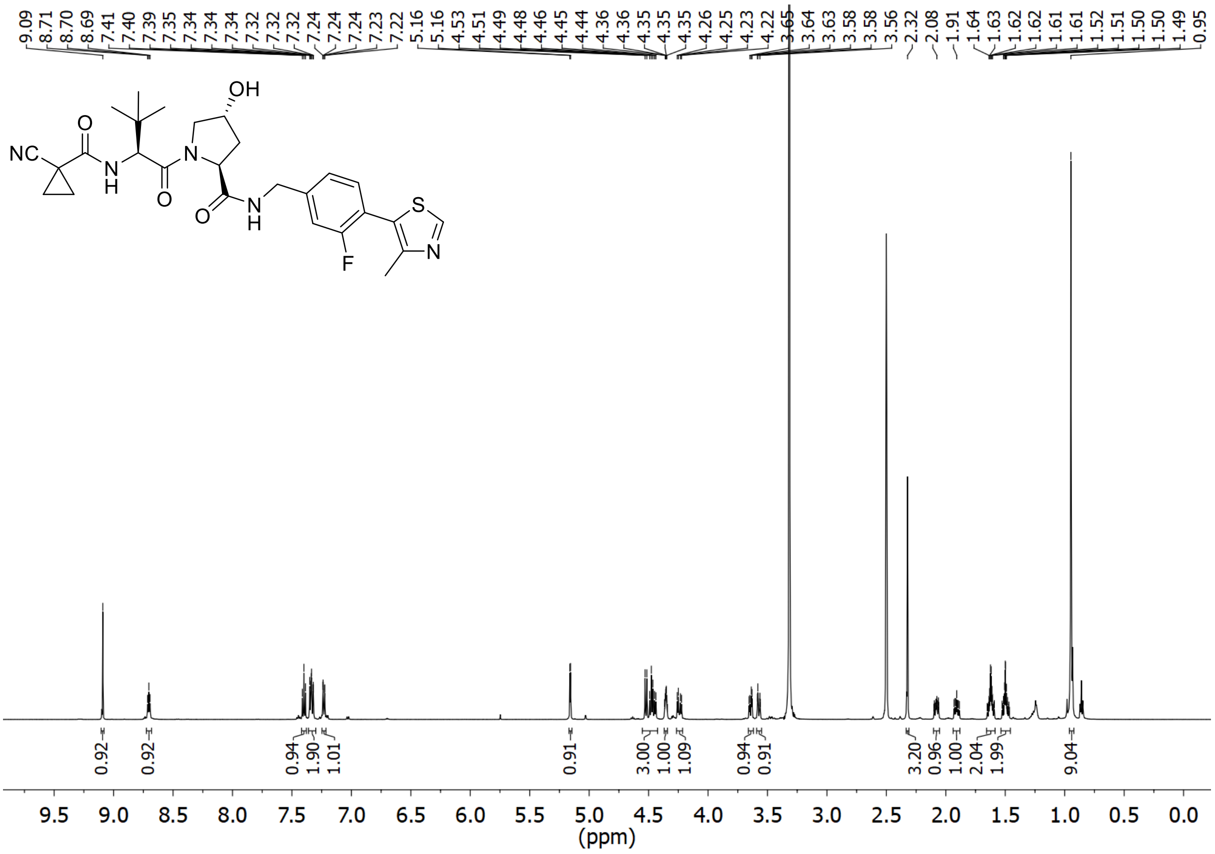

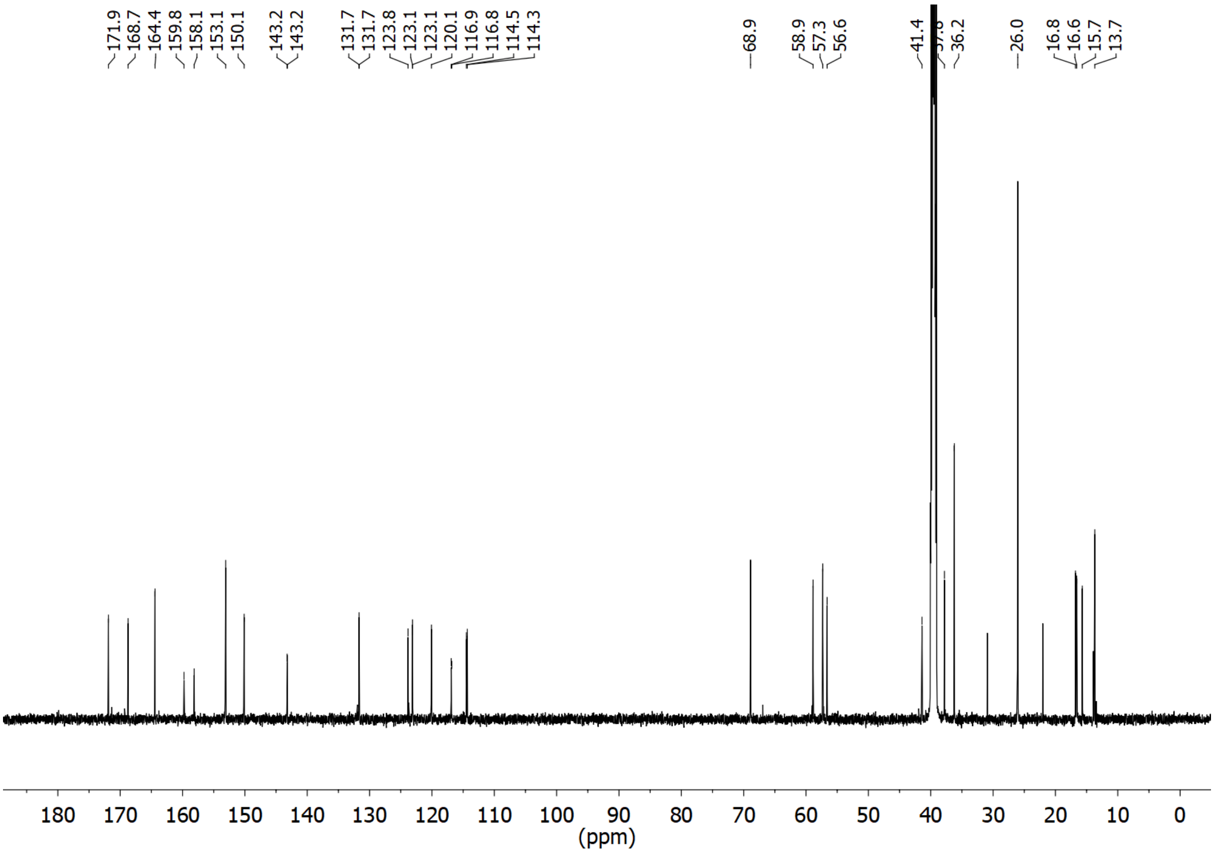

^{1}H and ^{13}C NMR Spectra of compound **9**

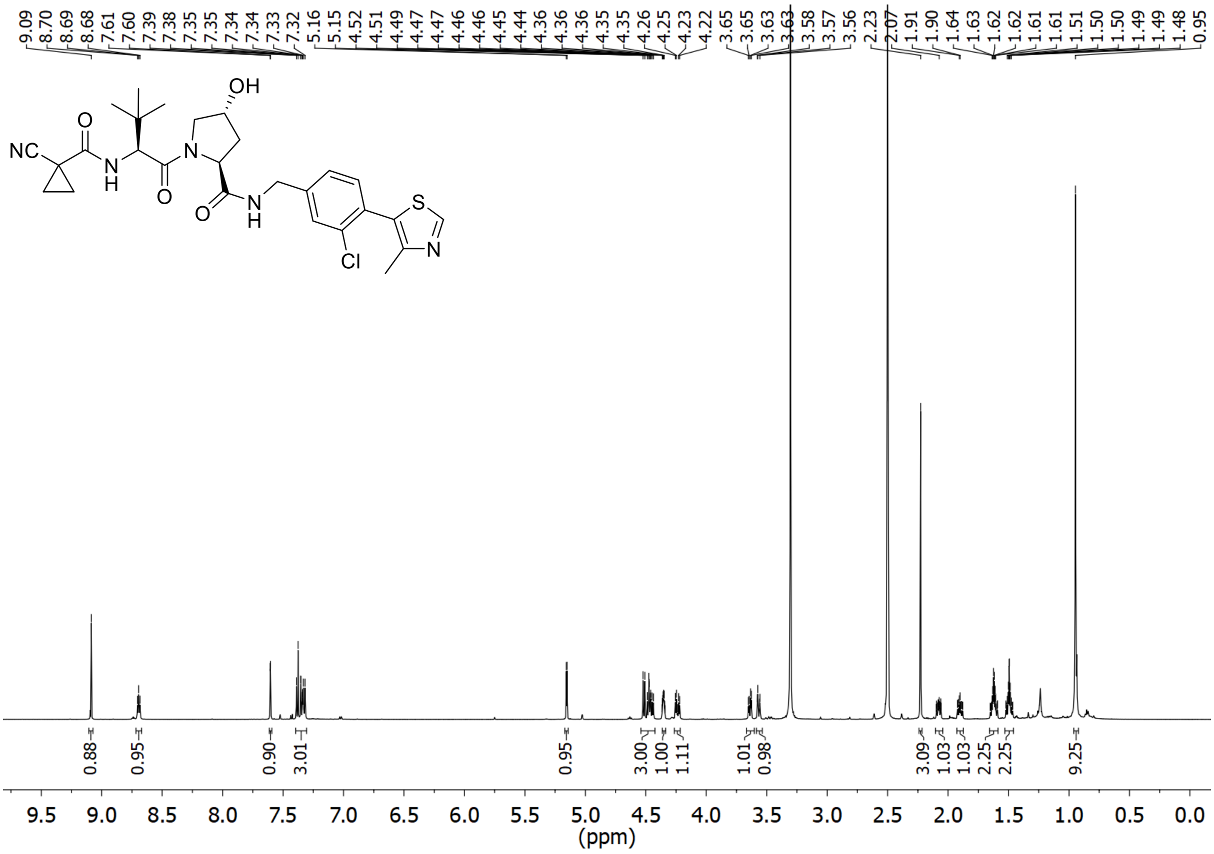

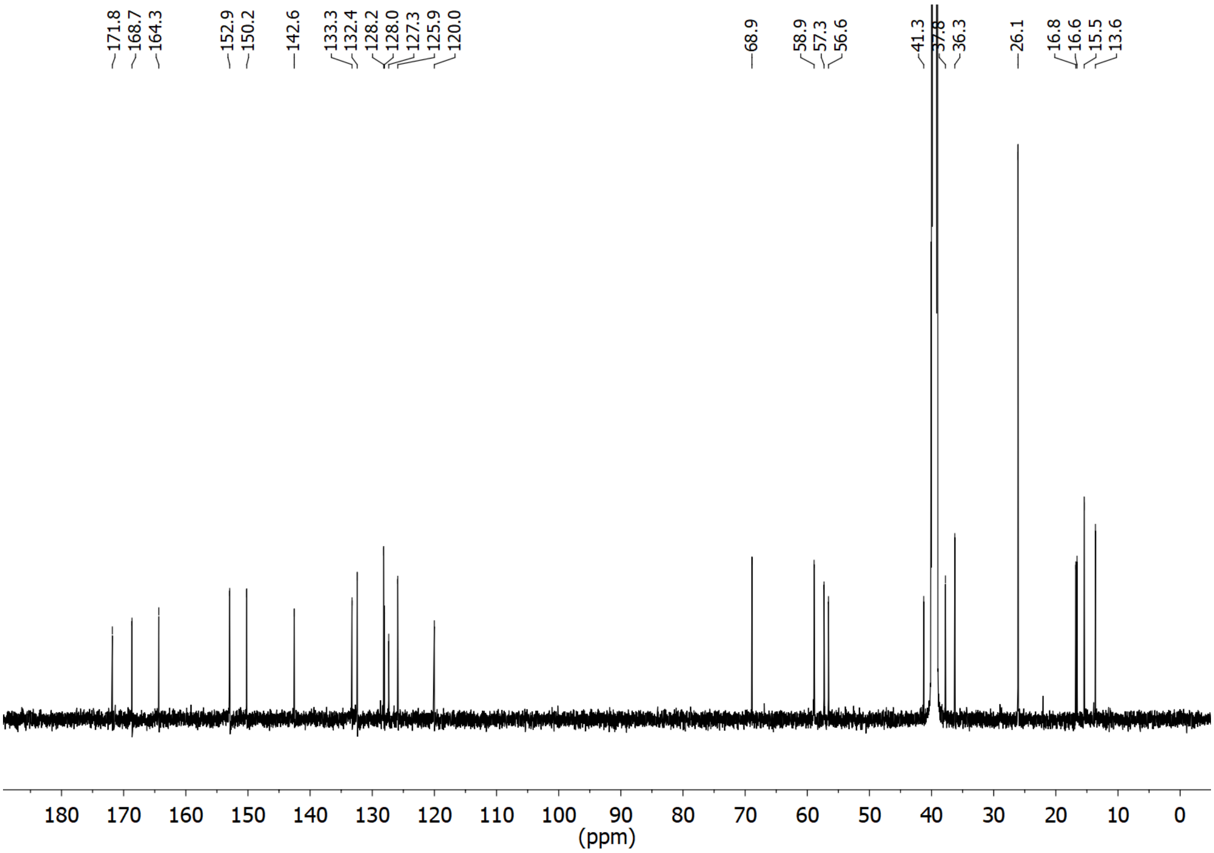

^{1}H and ^{13}C NMR Spectra of compound **10**

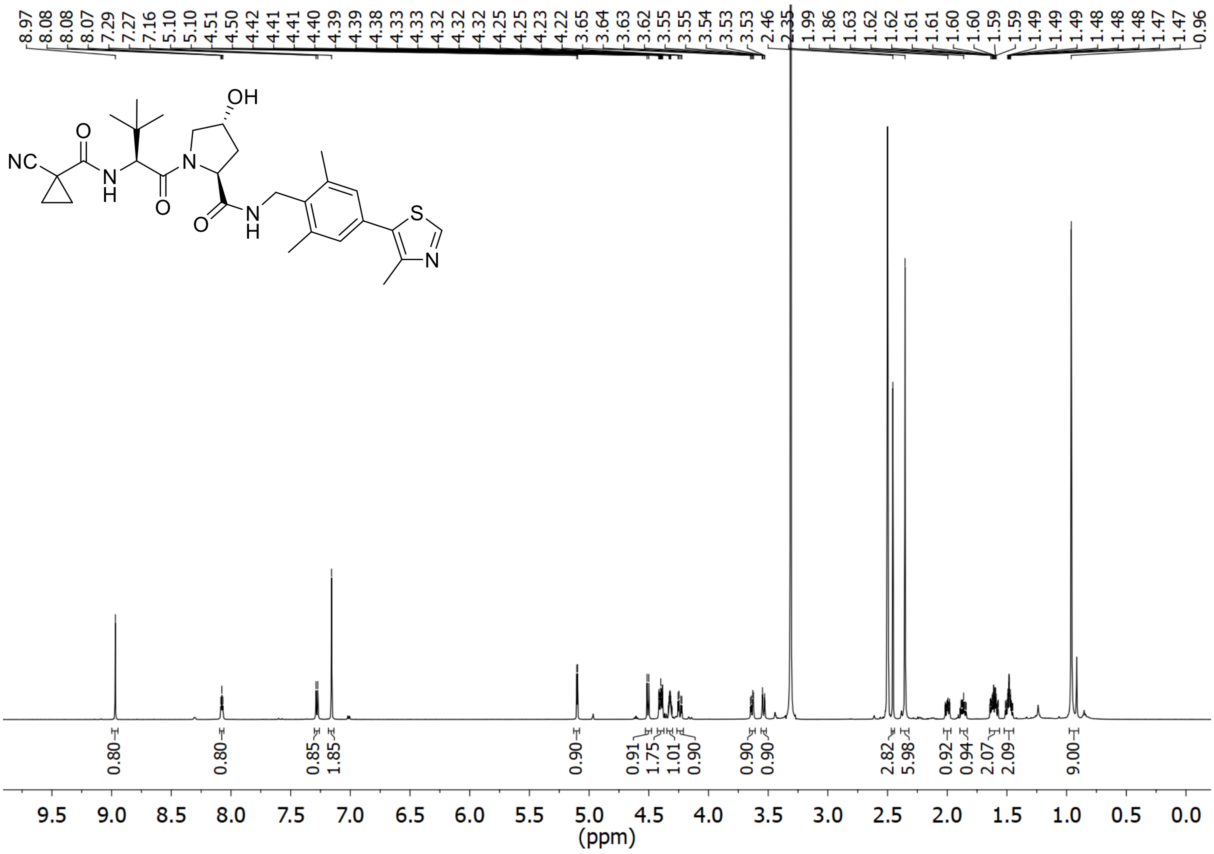

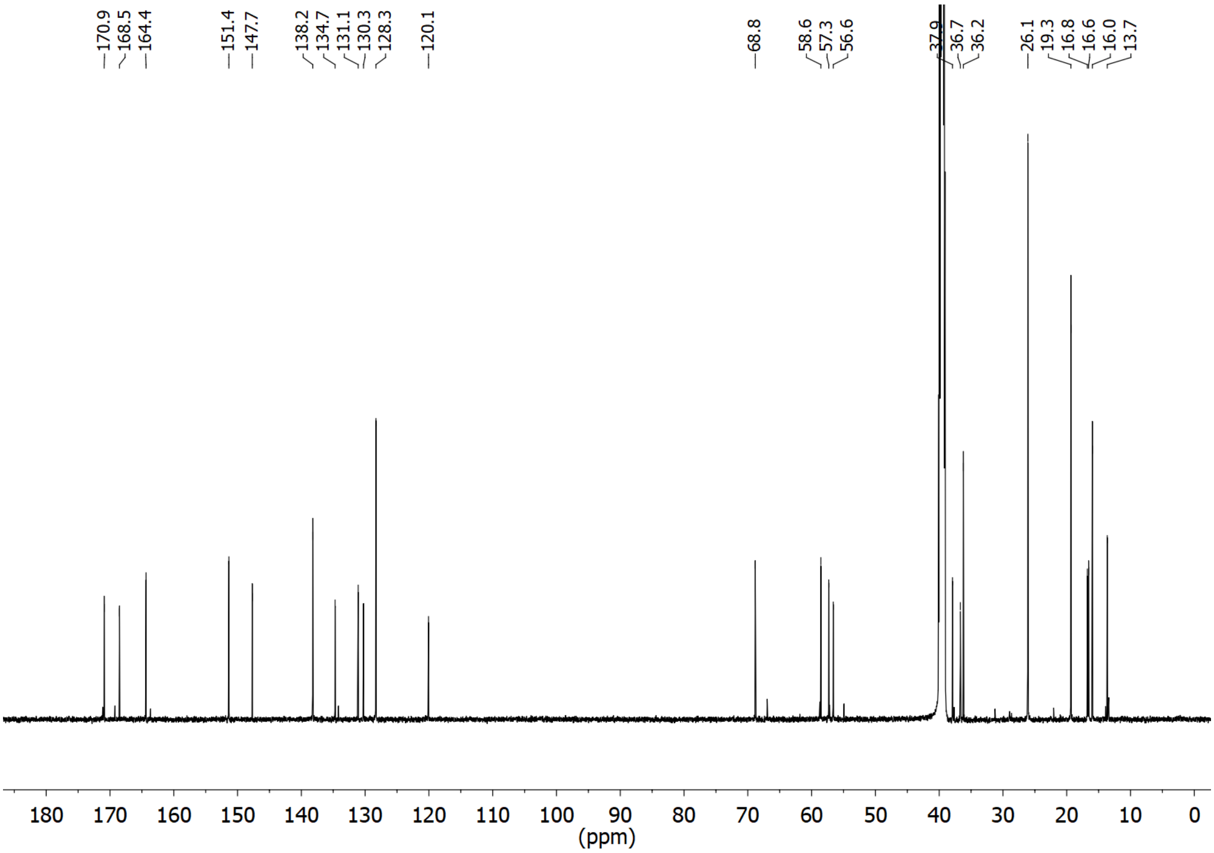

^{1}H and ^{13}C NMR Spectra of compound **11**

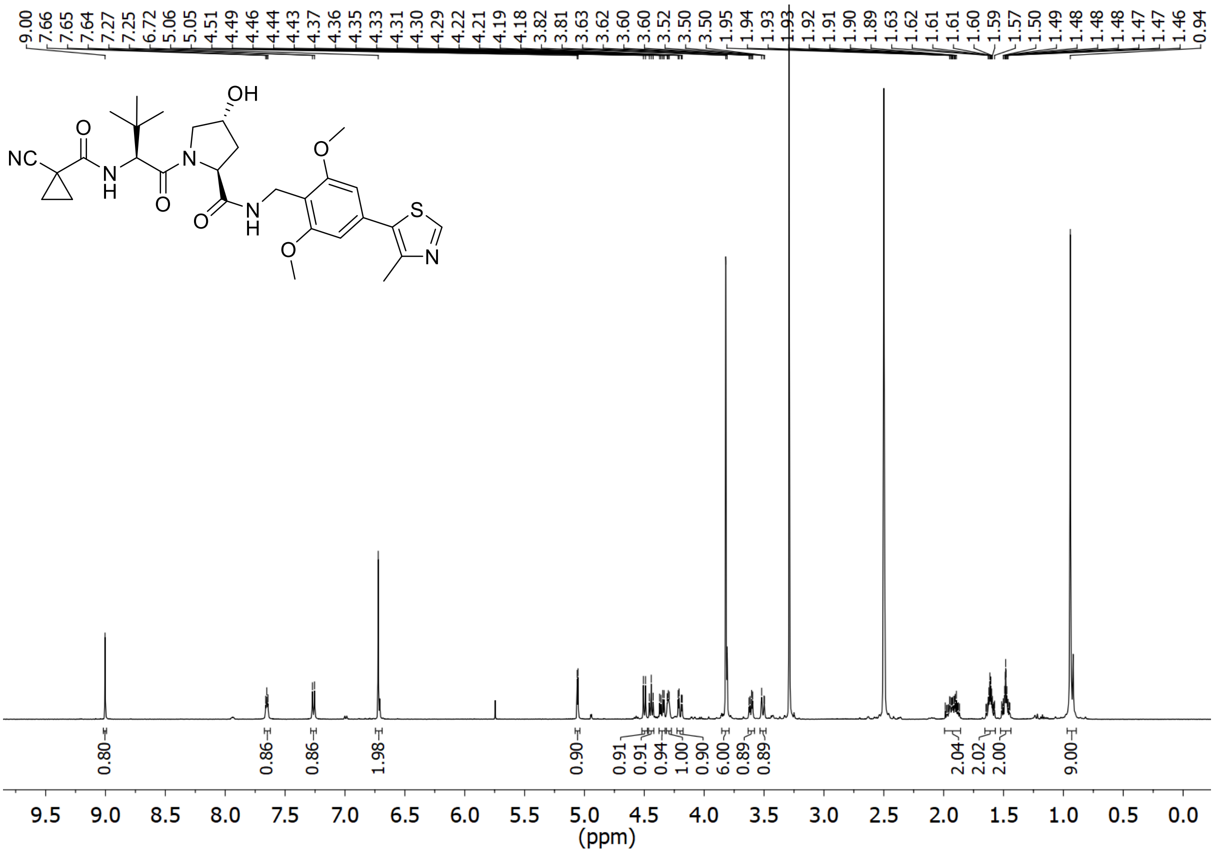

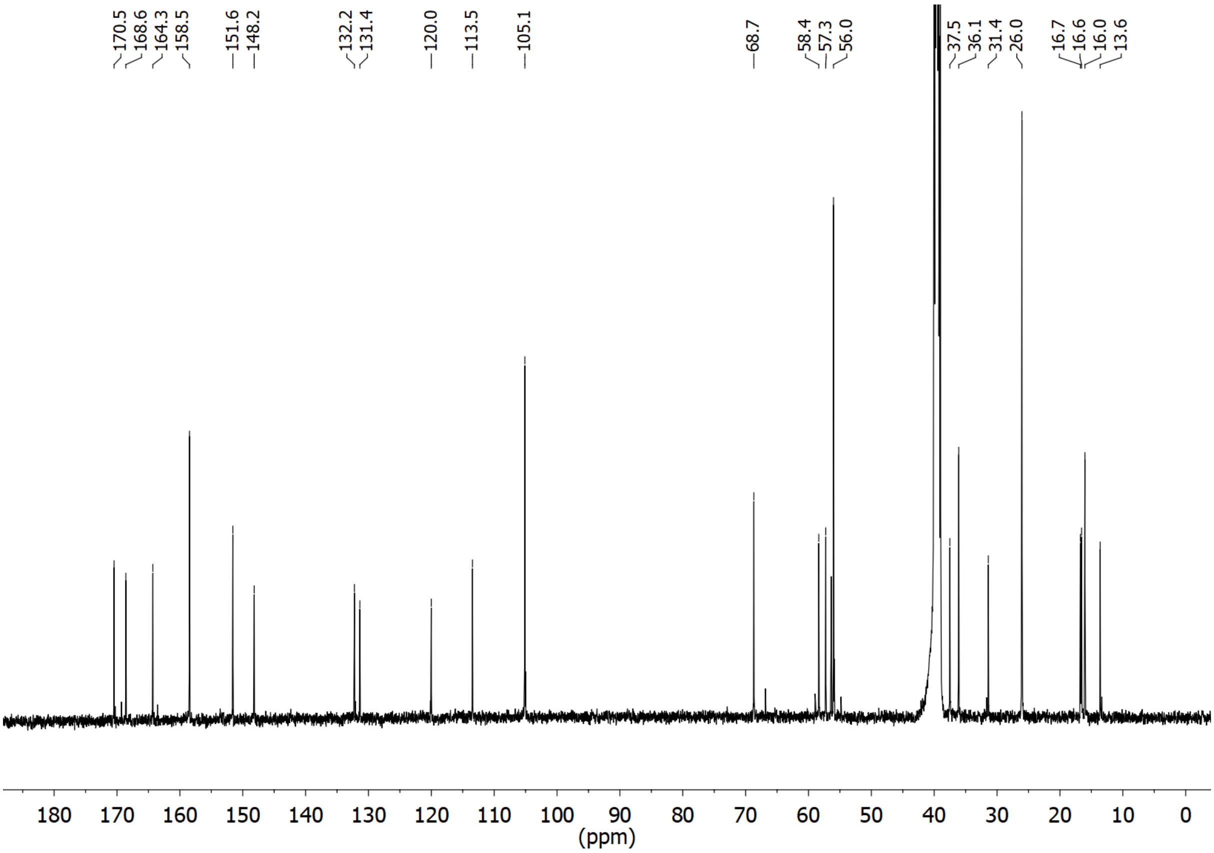

^{1}H and ^{13}C NMR Spectra of compound **12**

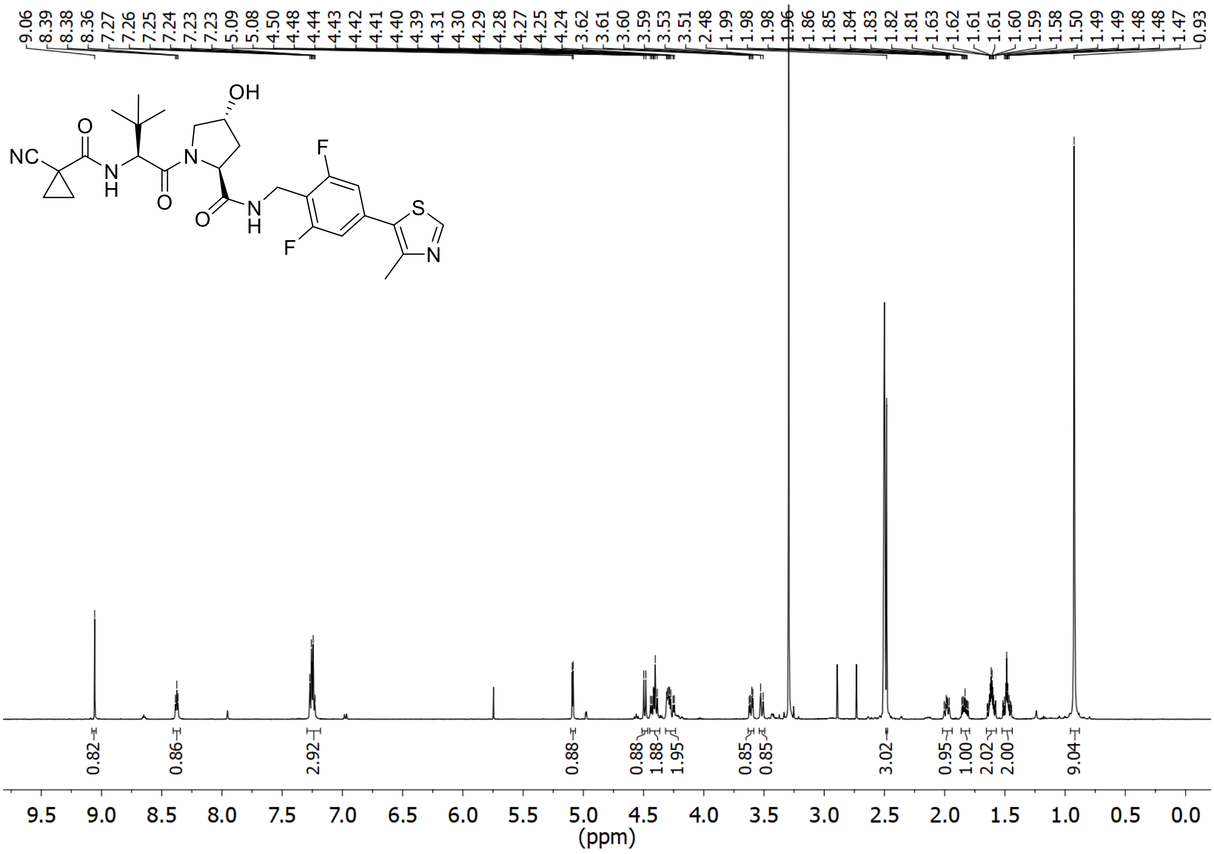

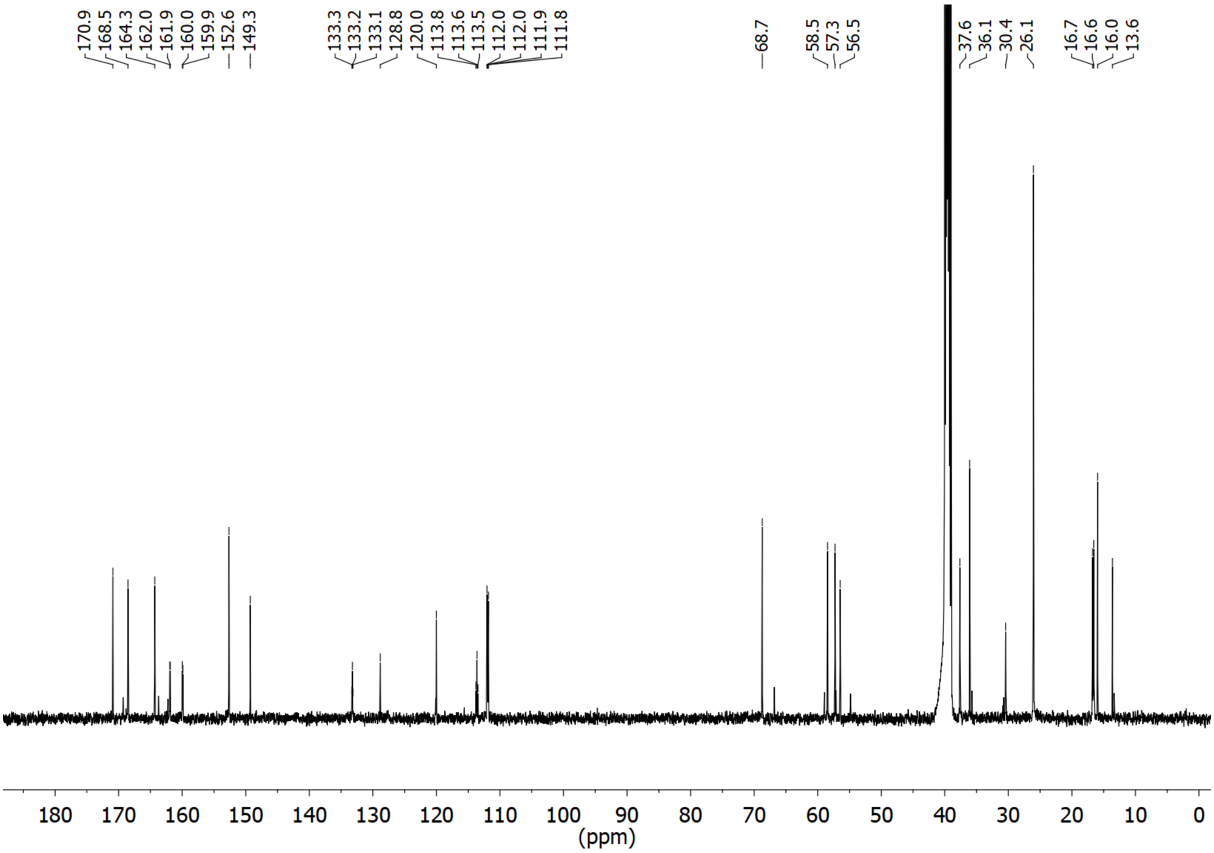

^{1}H and ^{13}C NMR Spectra of compound **13**

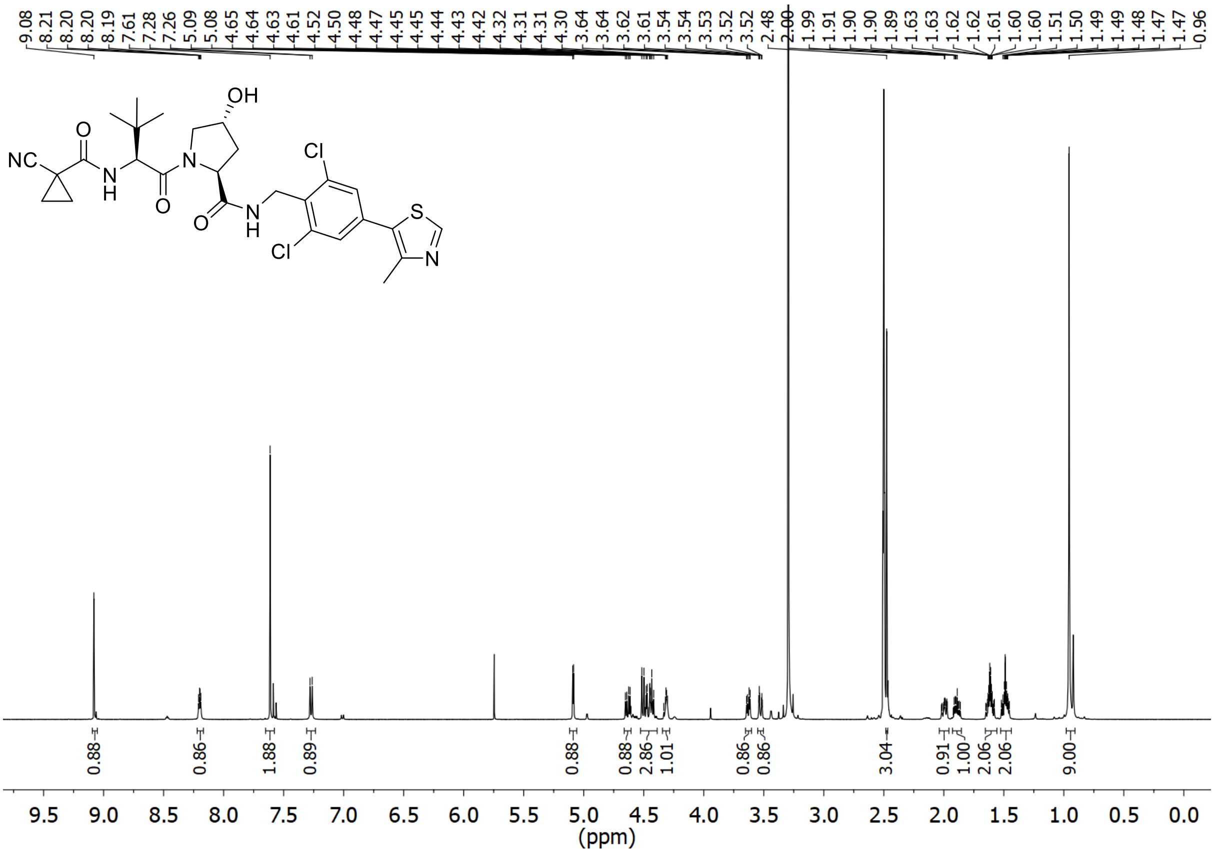

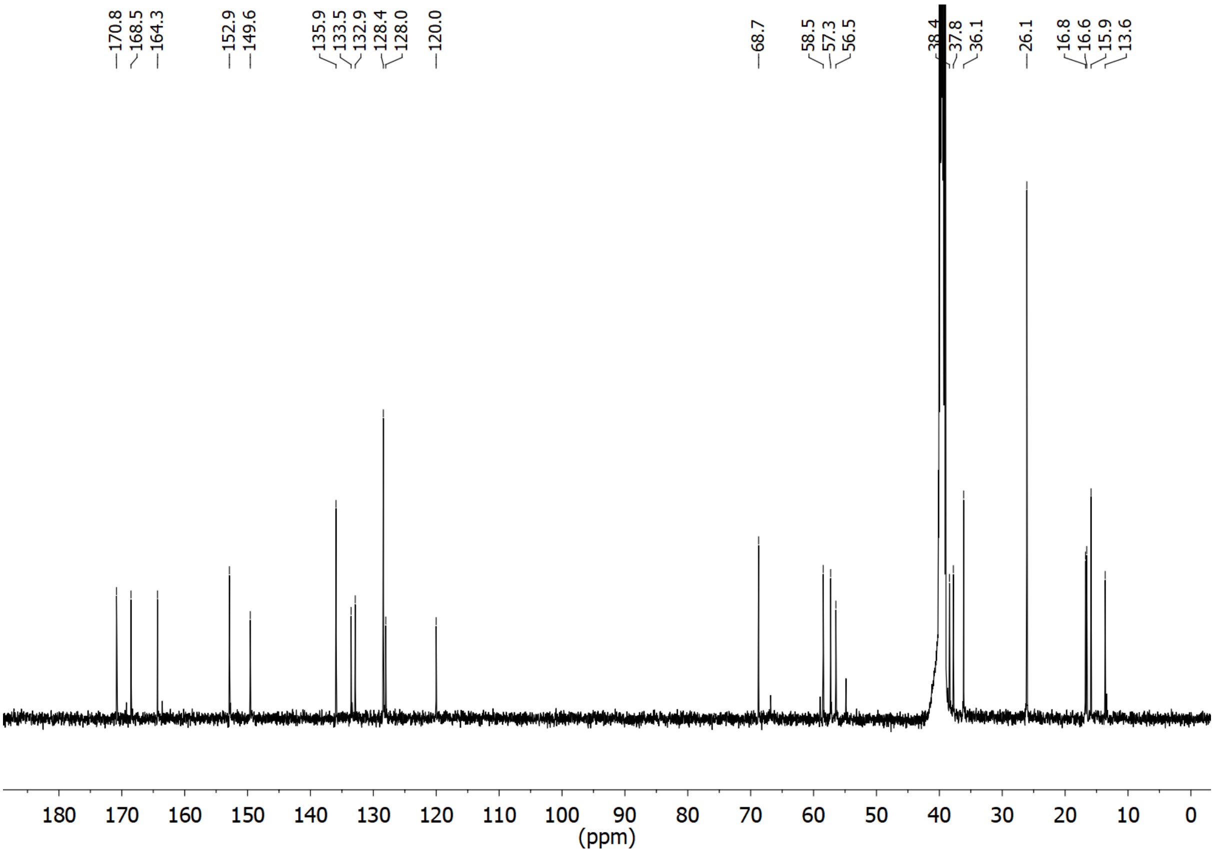

^{1}H and ^{13}C NMR Spectra of compound **14**

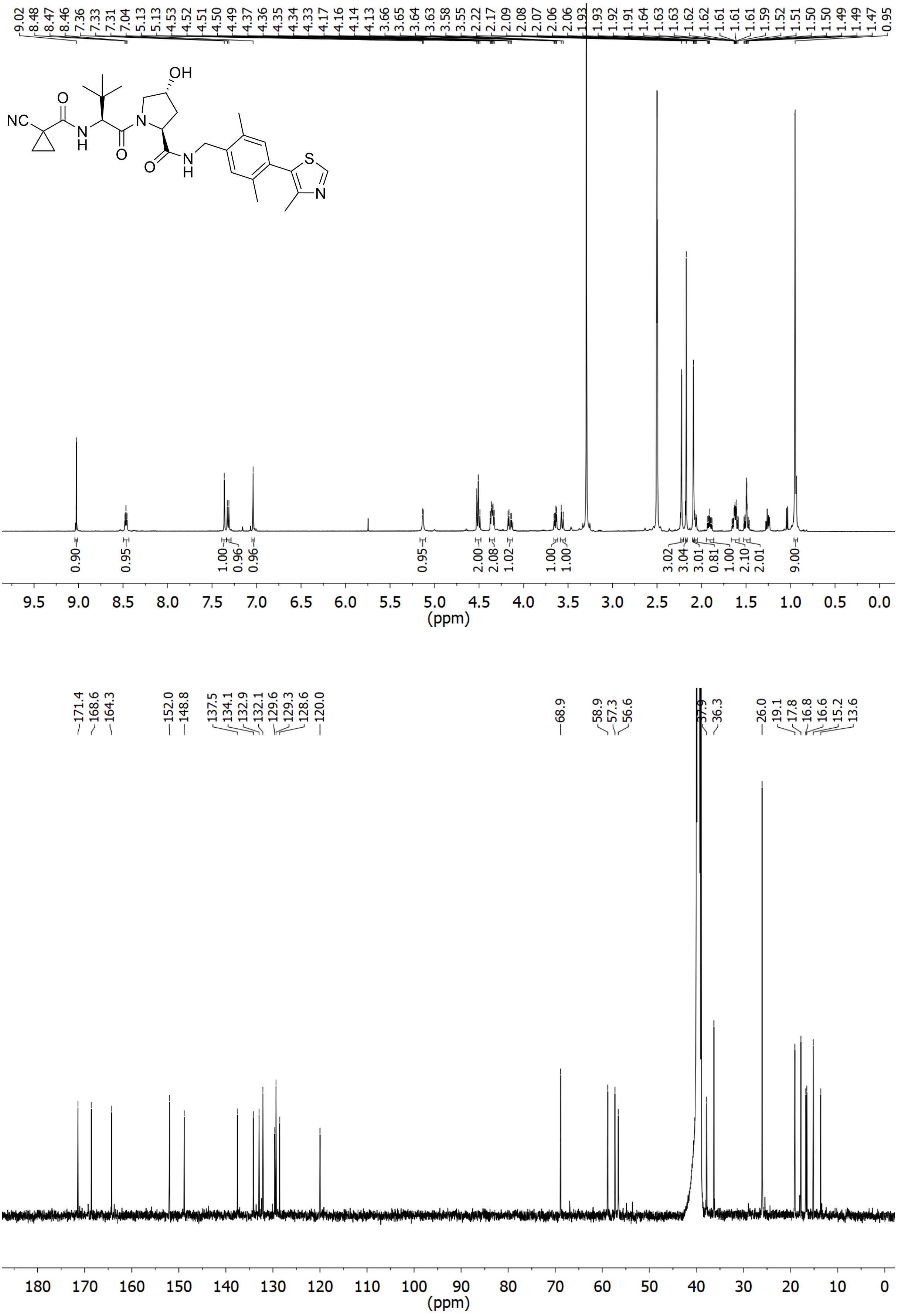

^{1}H and ^{13}C NMR Spectra of compound **15**

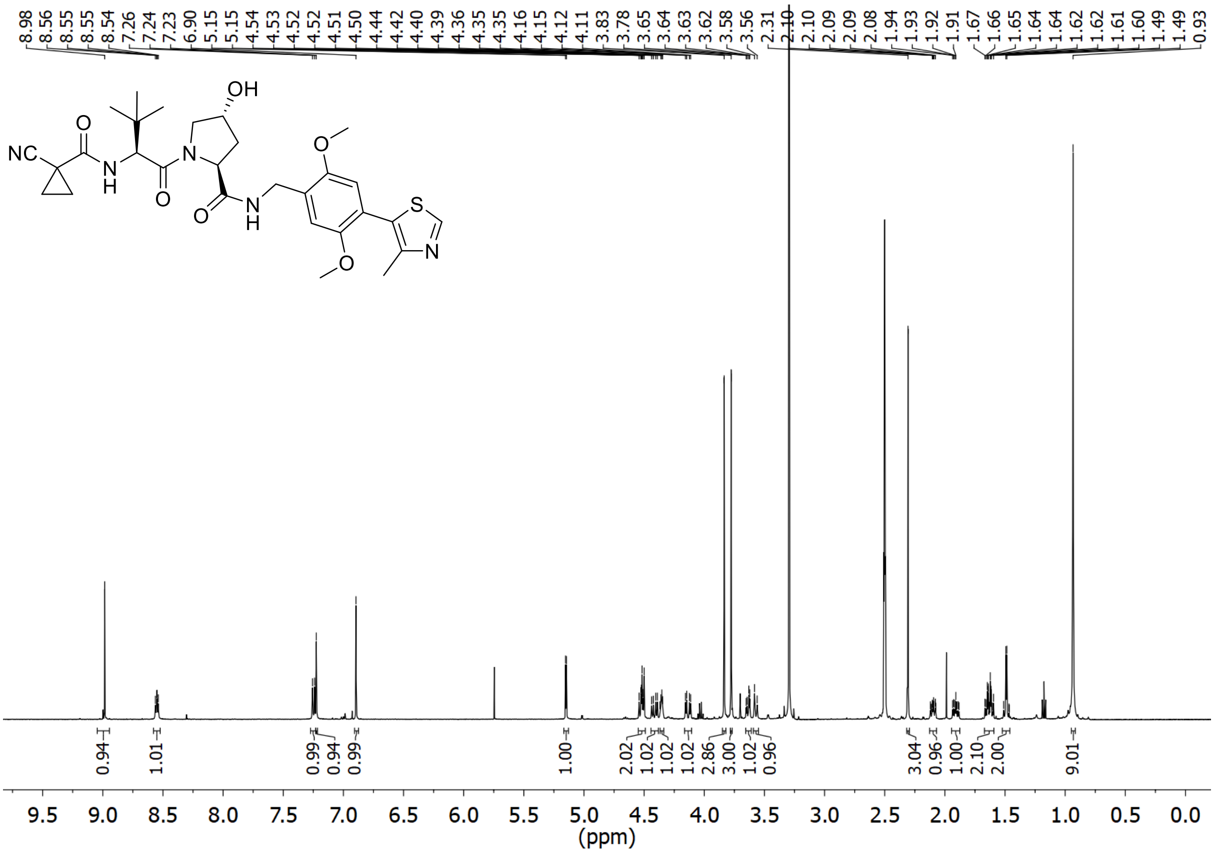

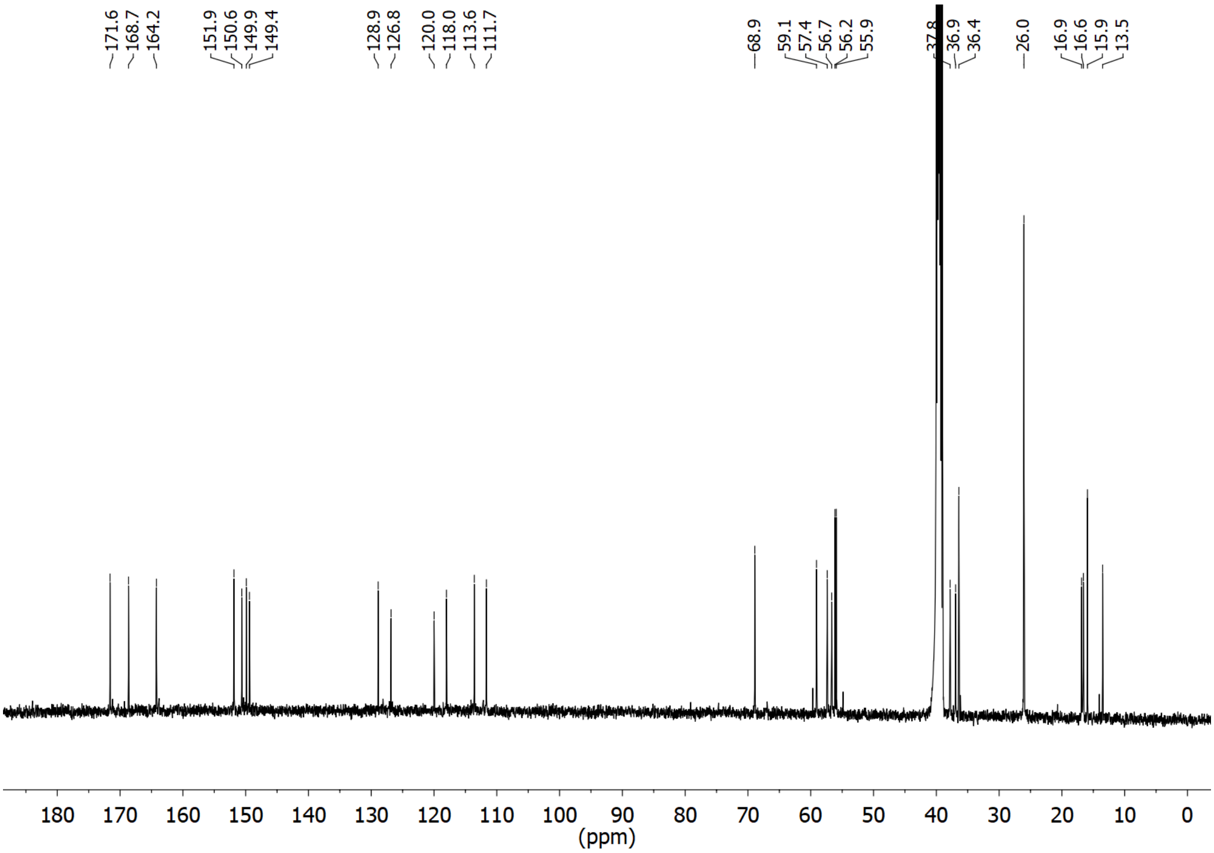

^{1}H and ^{13}C NMR Spectra of compound **16**

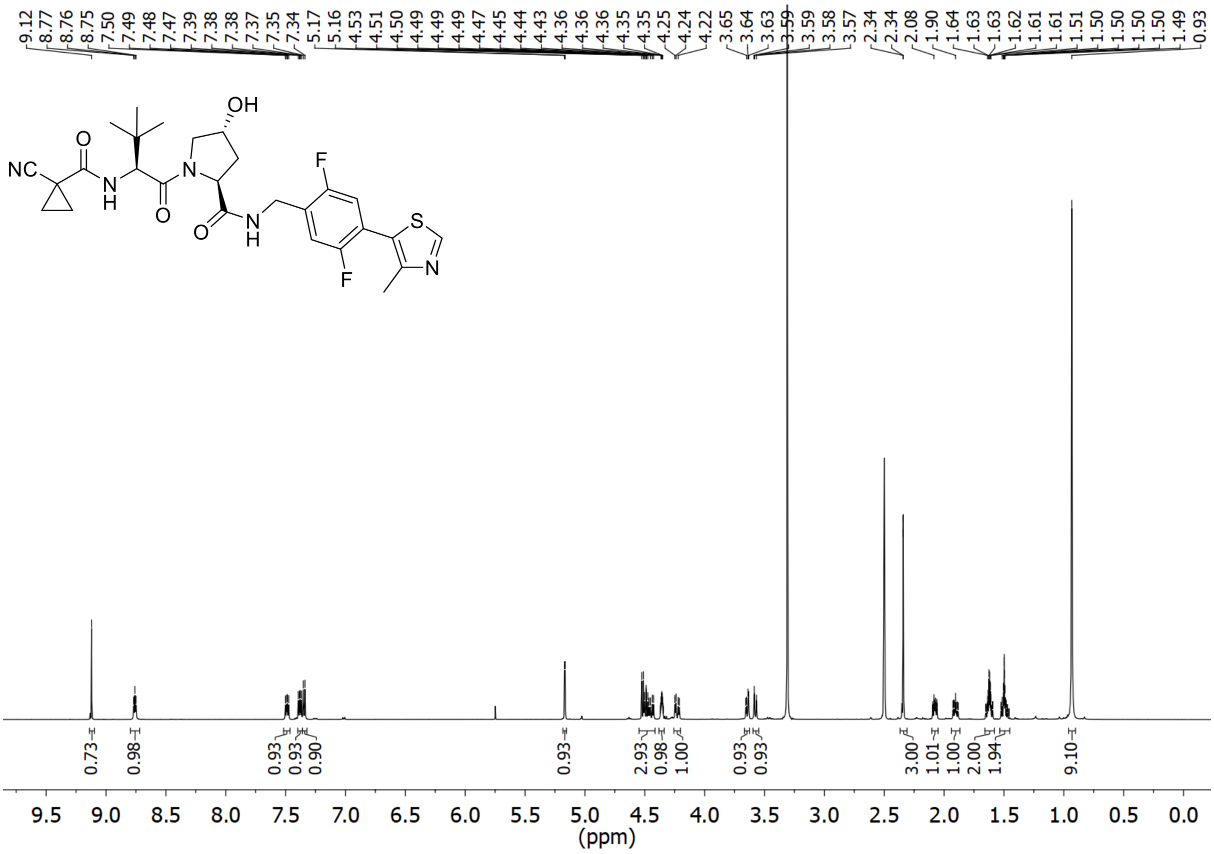

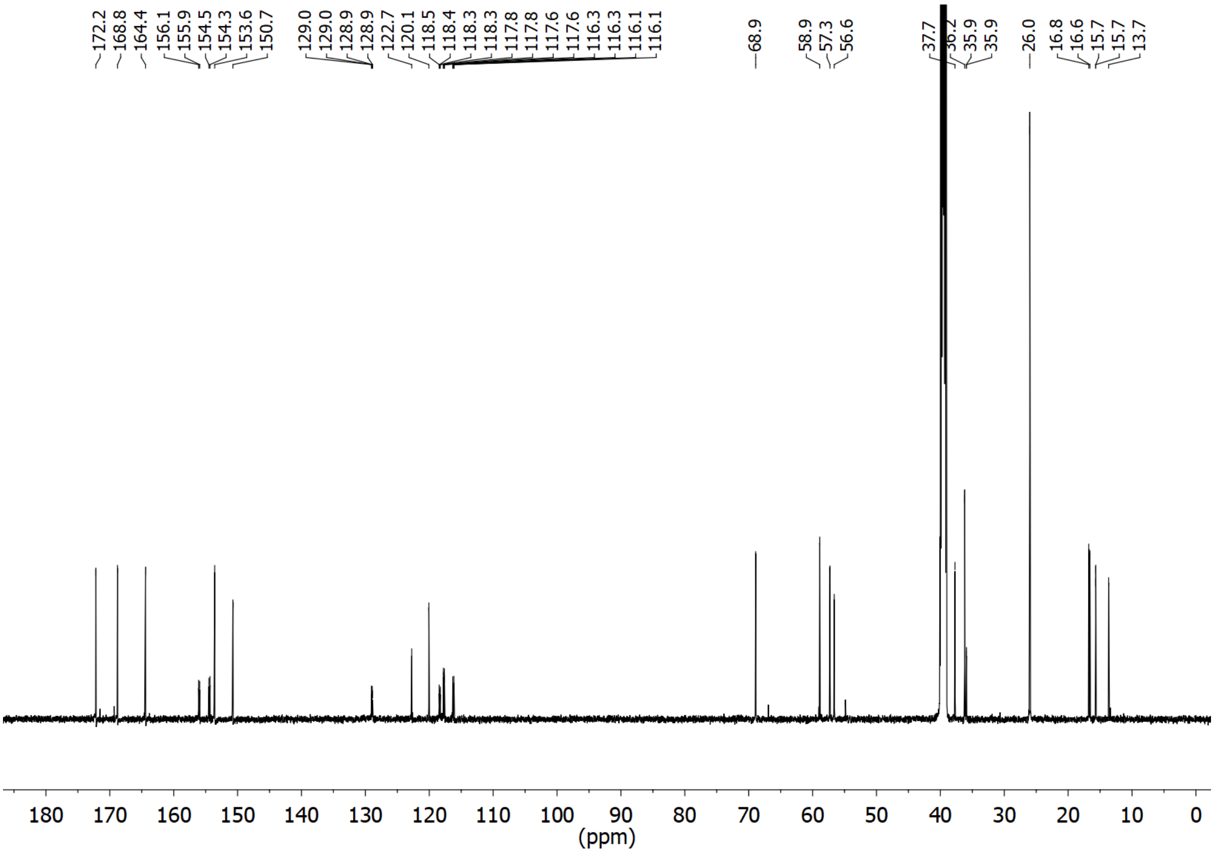

^{1}H and ^{13}C NMR Spectra of compound **17**

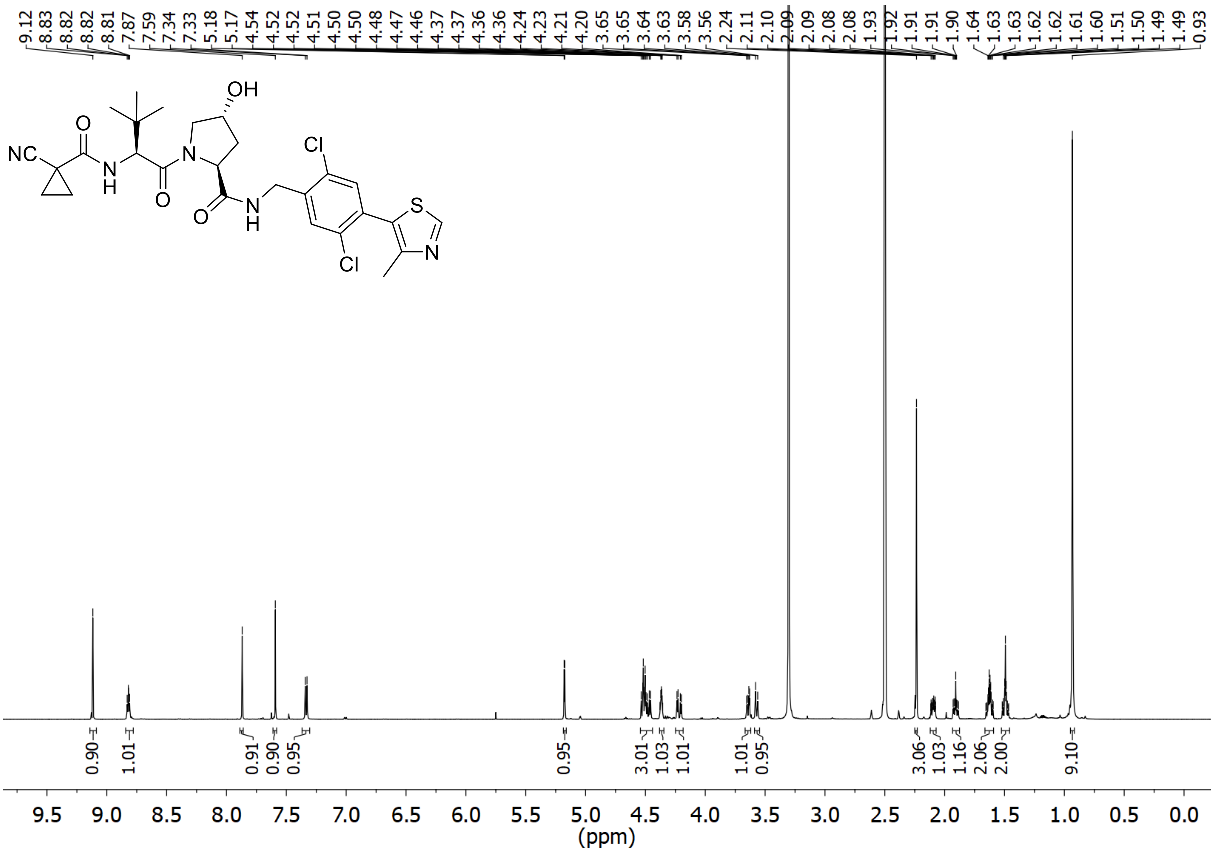

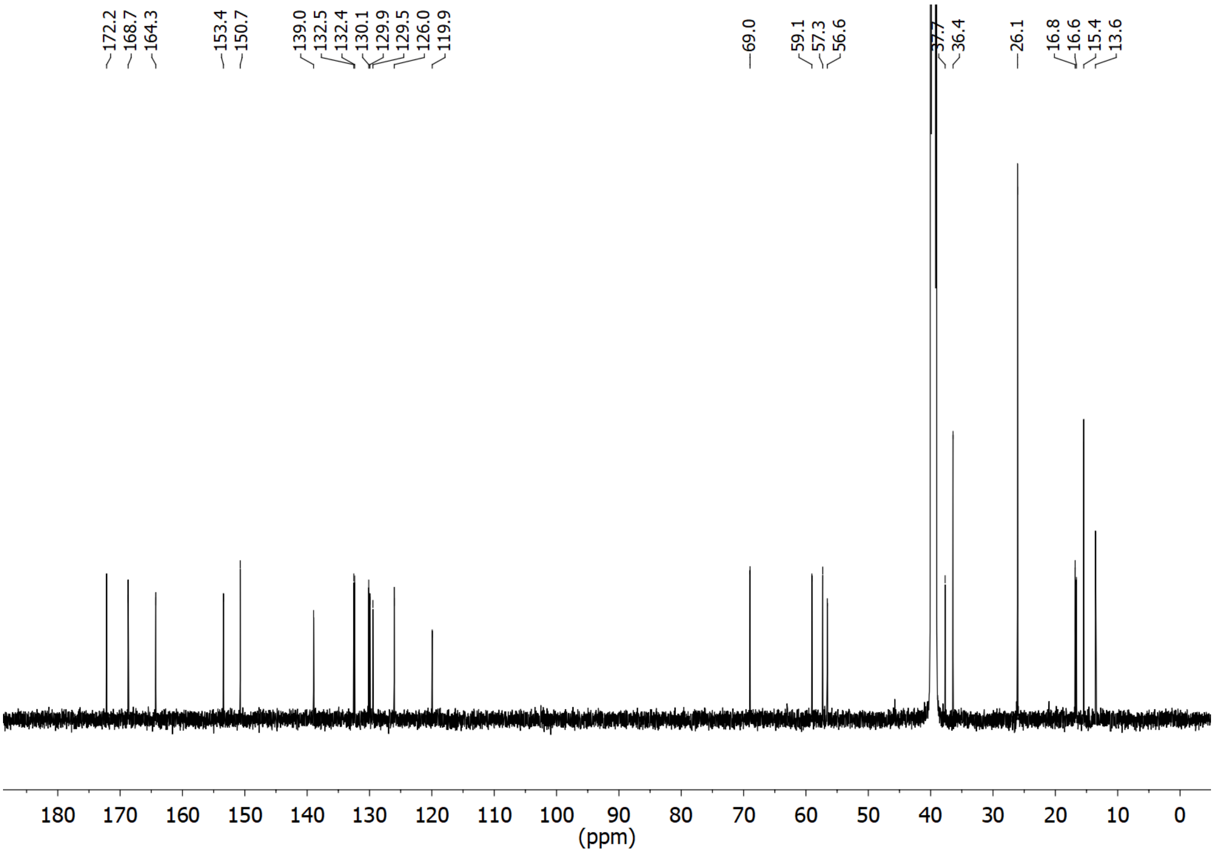

^{1}H and ^{13}C NMR Spectra of compound **18**

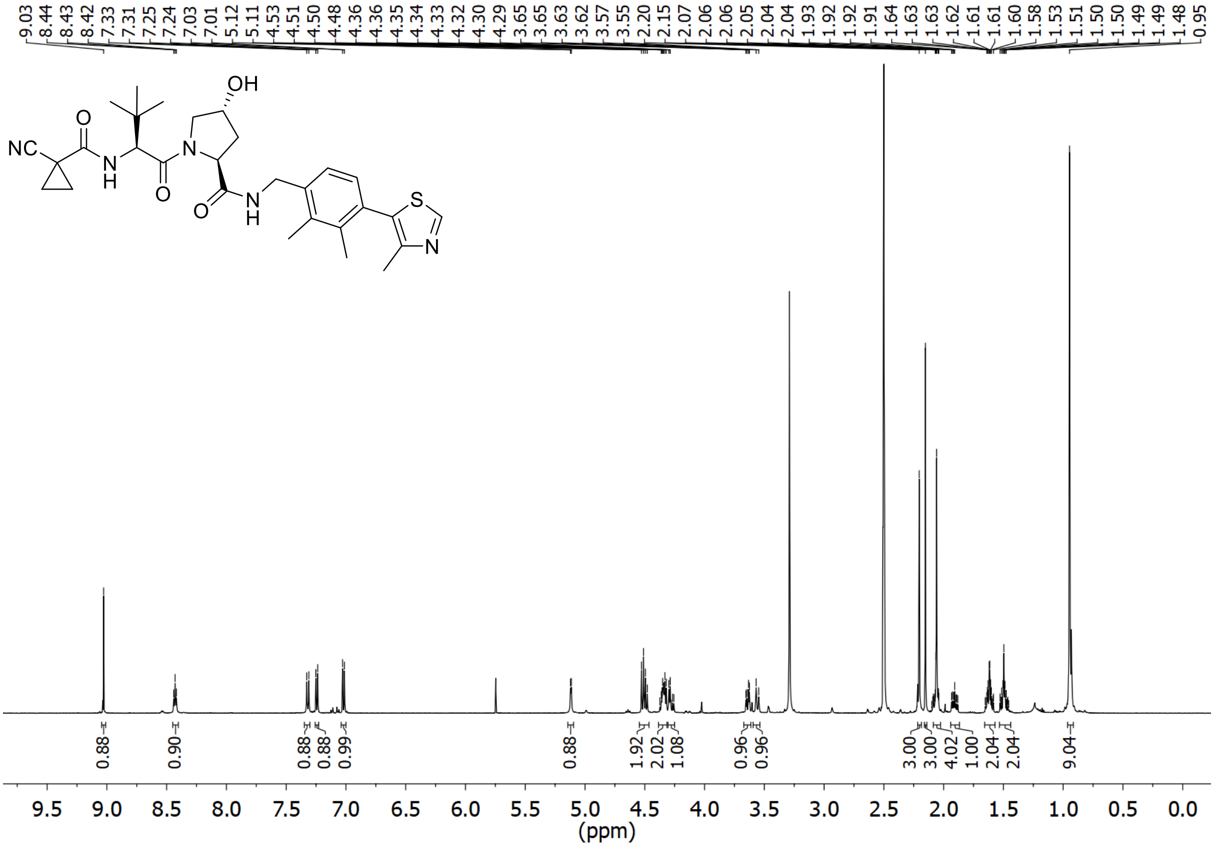

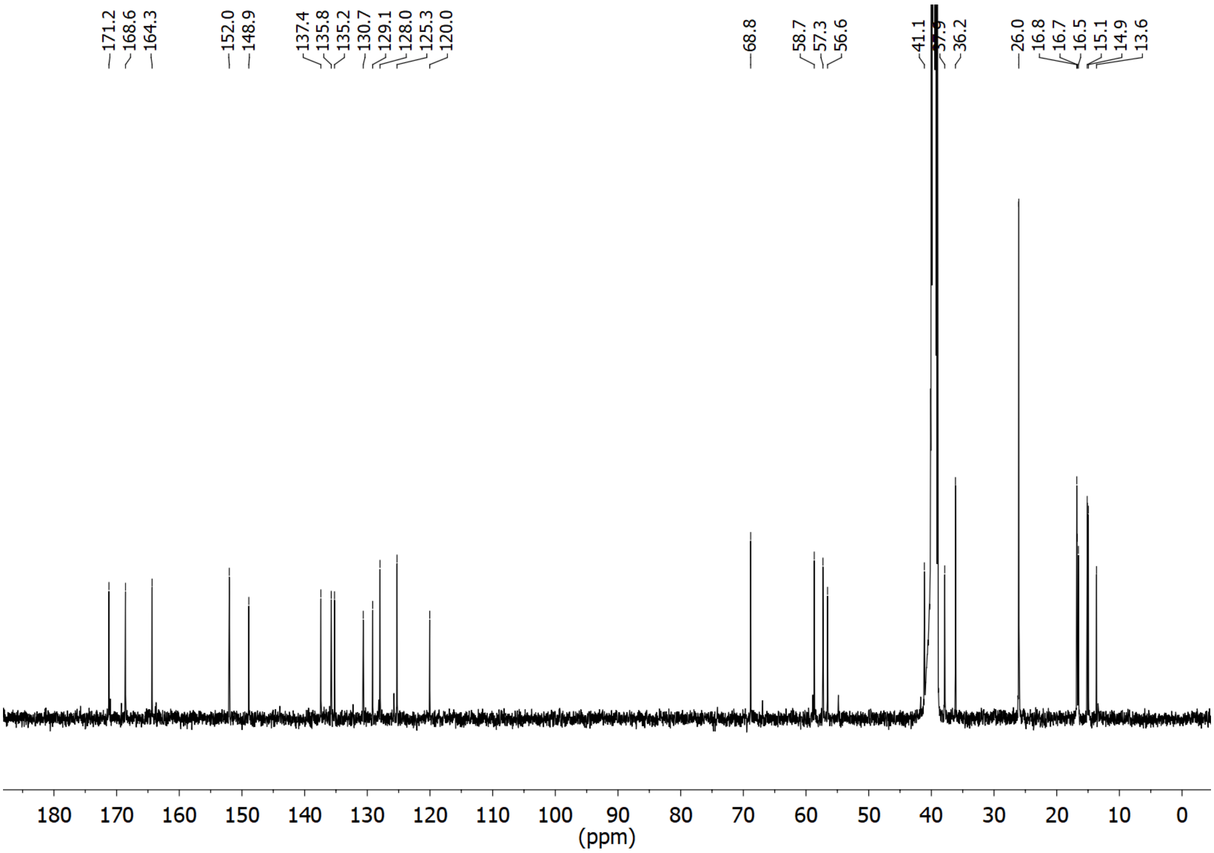

^1H and ^{13}C NMR Spectra of compound **19**

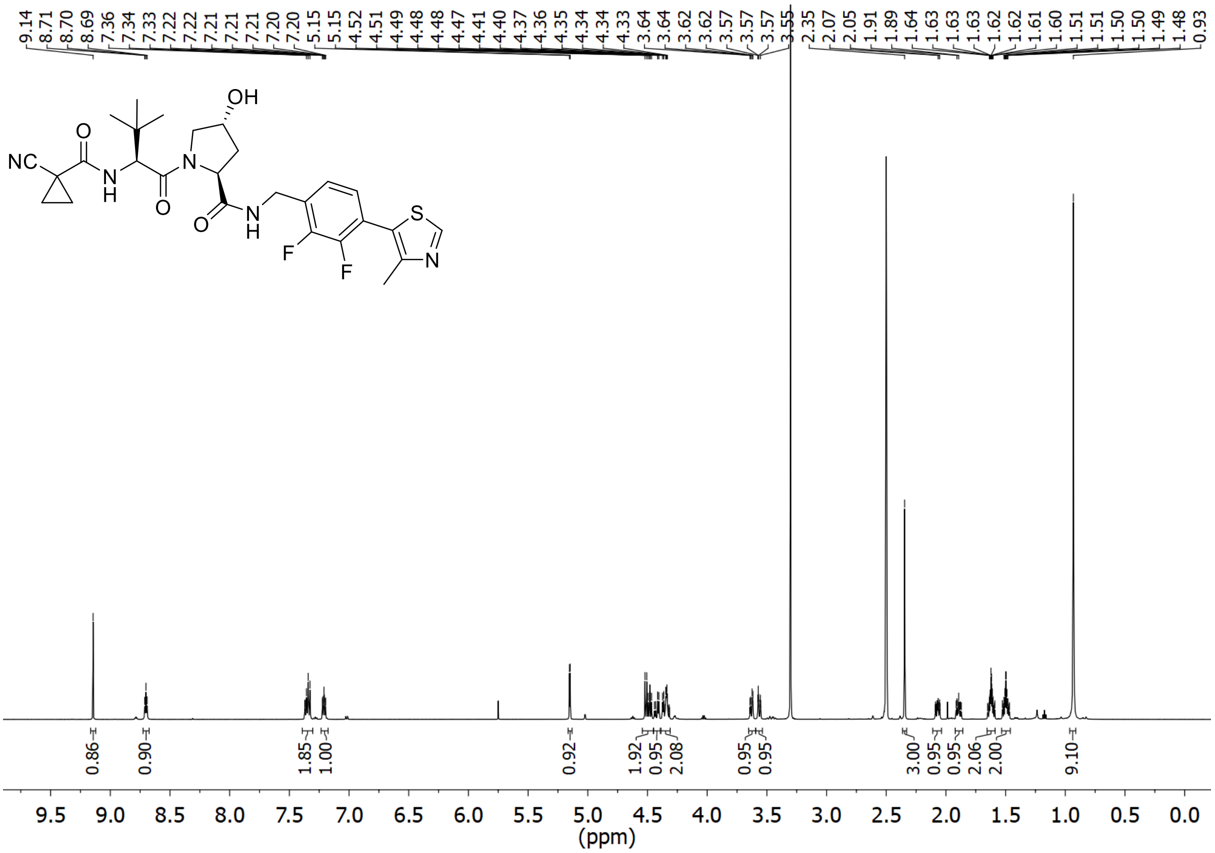

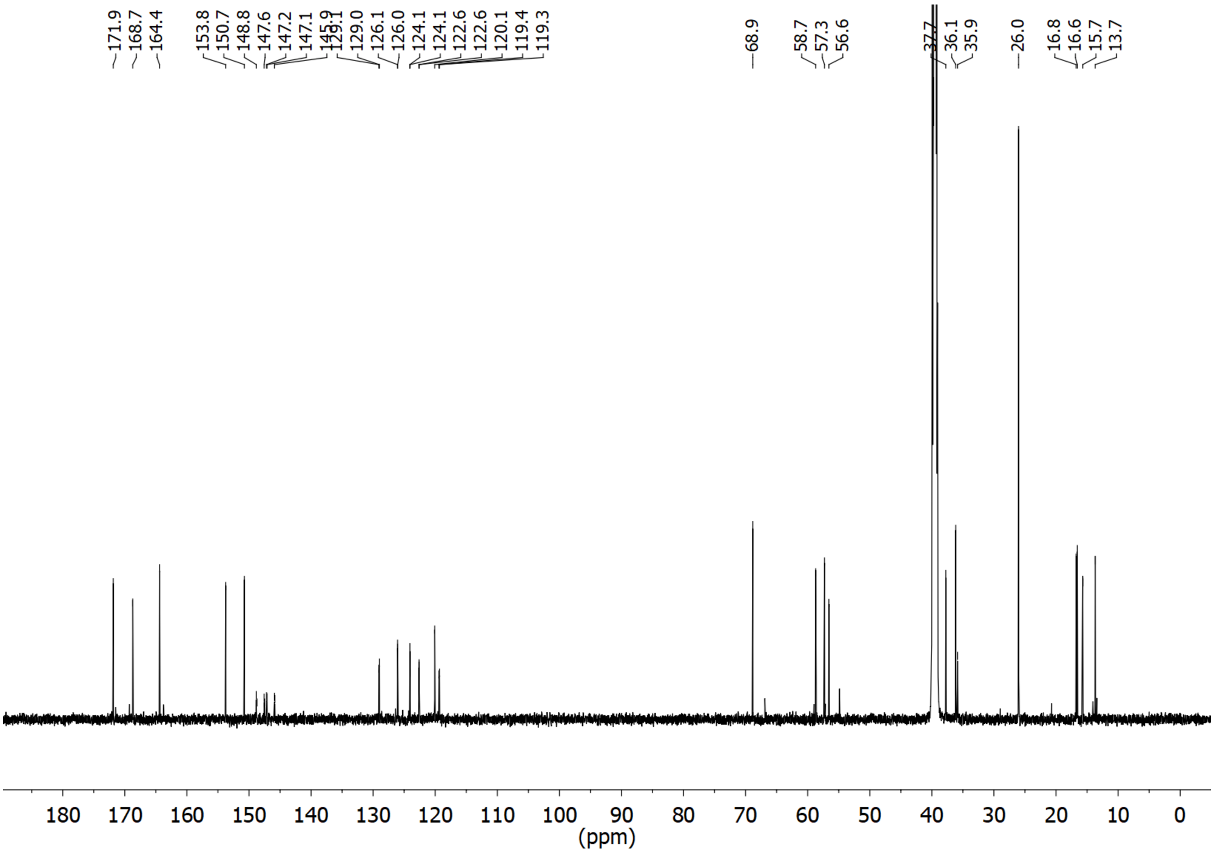

^{1}H and ^{13}C NMR Spectra of compound **20**

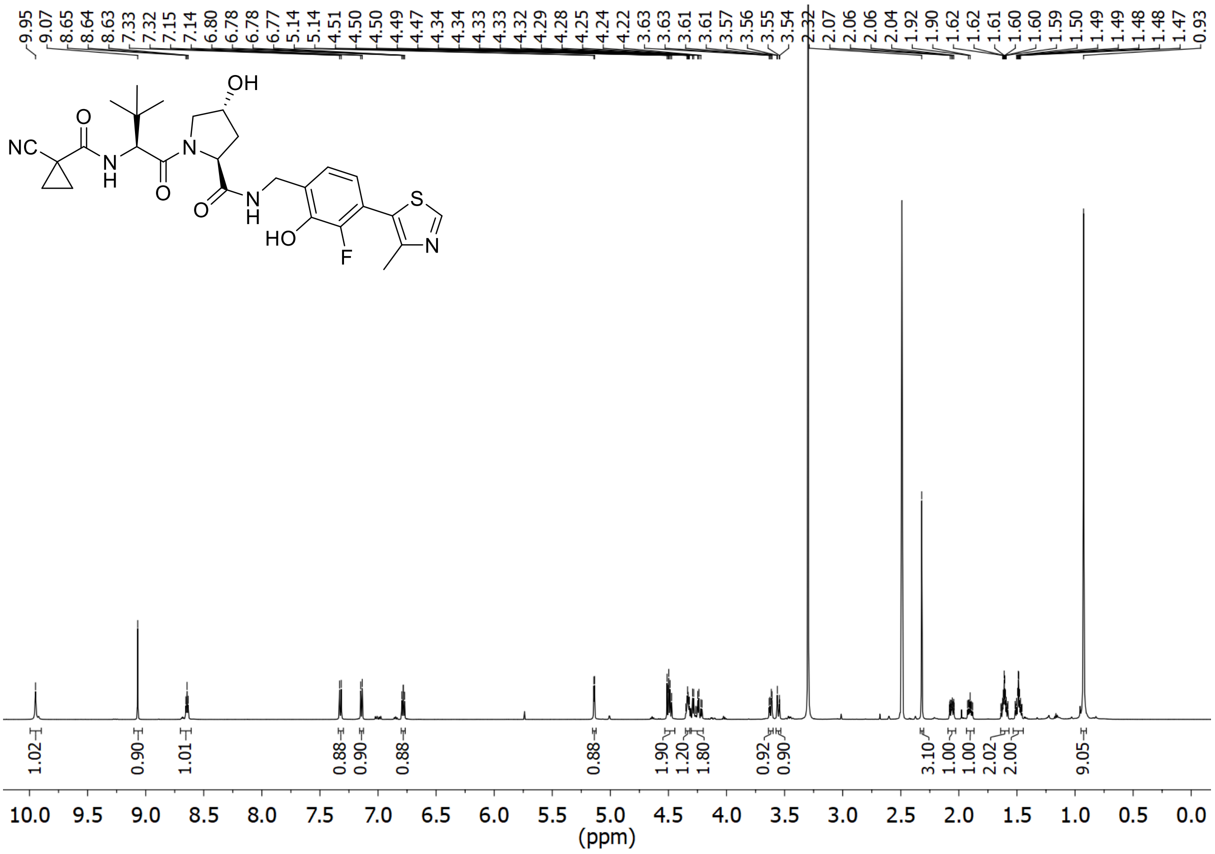

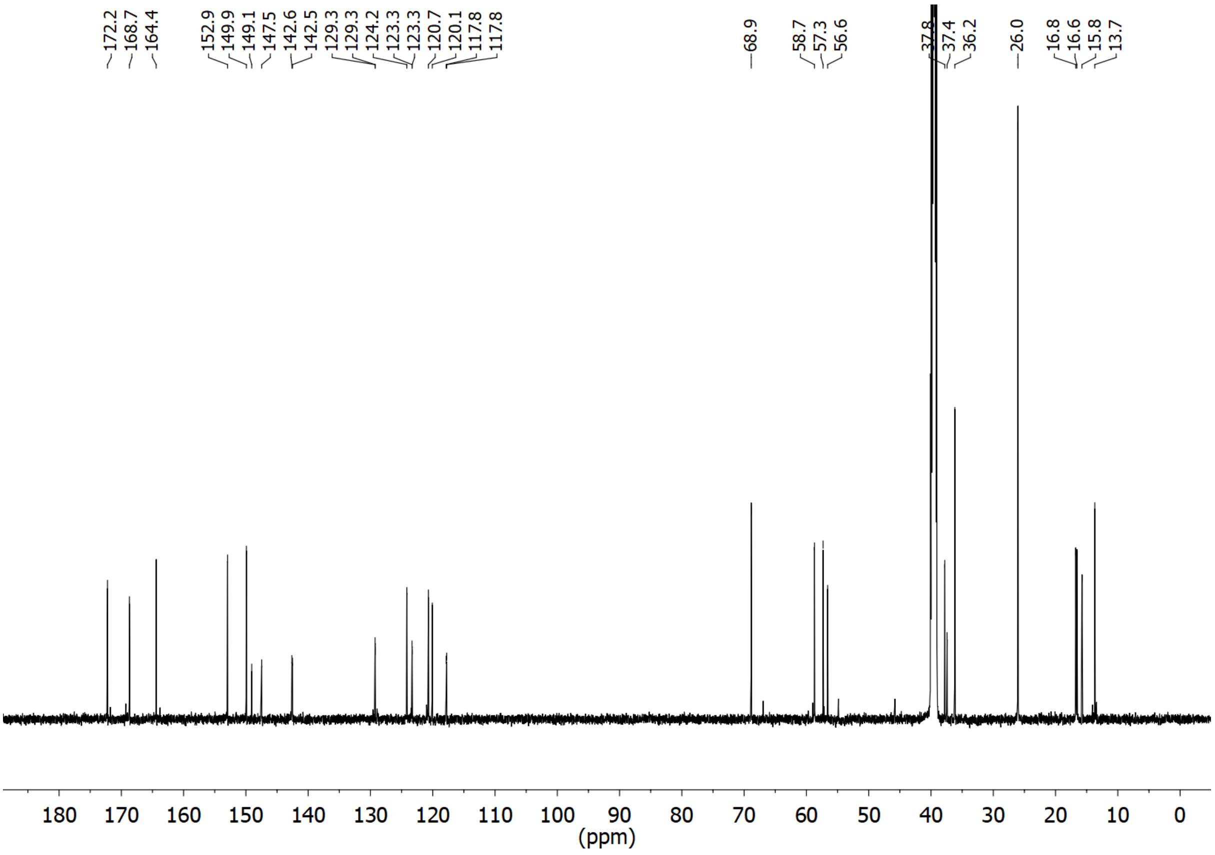

^{1}H and ^{13}C NMR Spectra of compound **21**

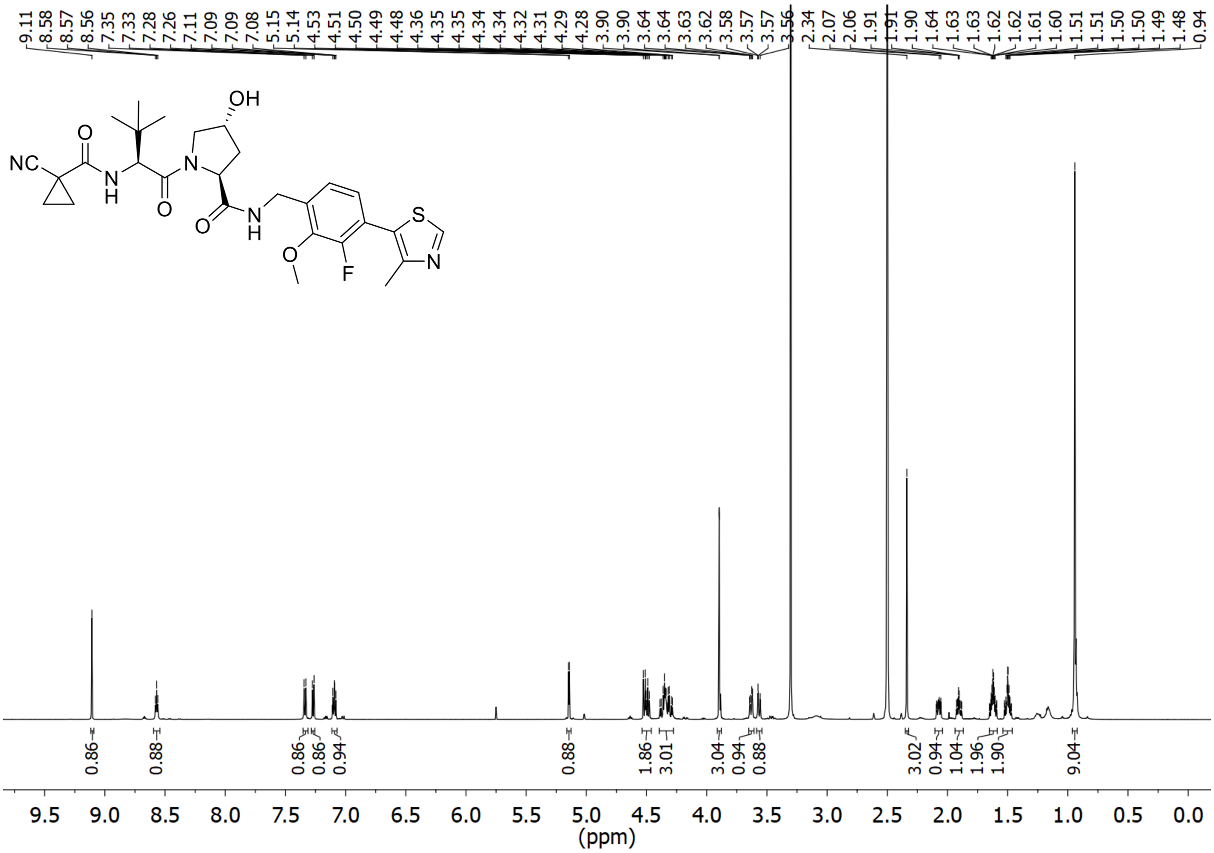

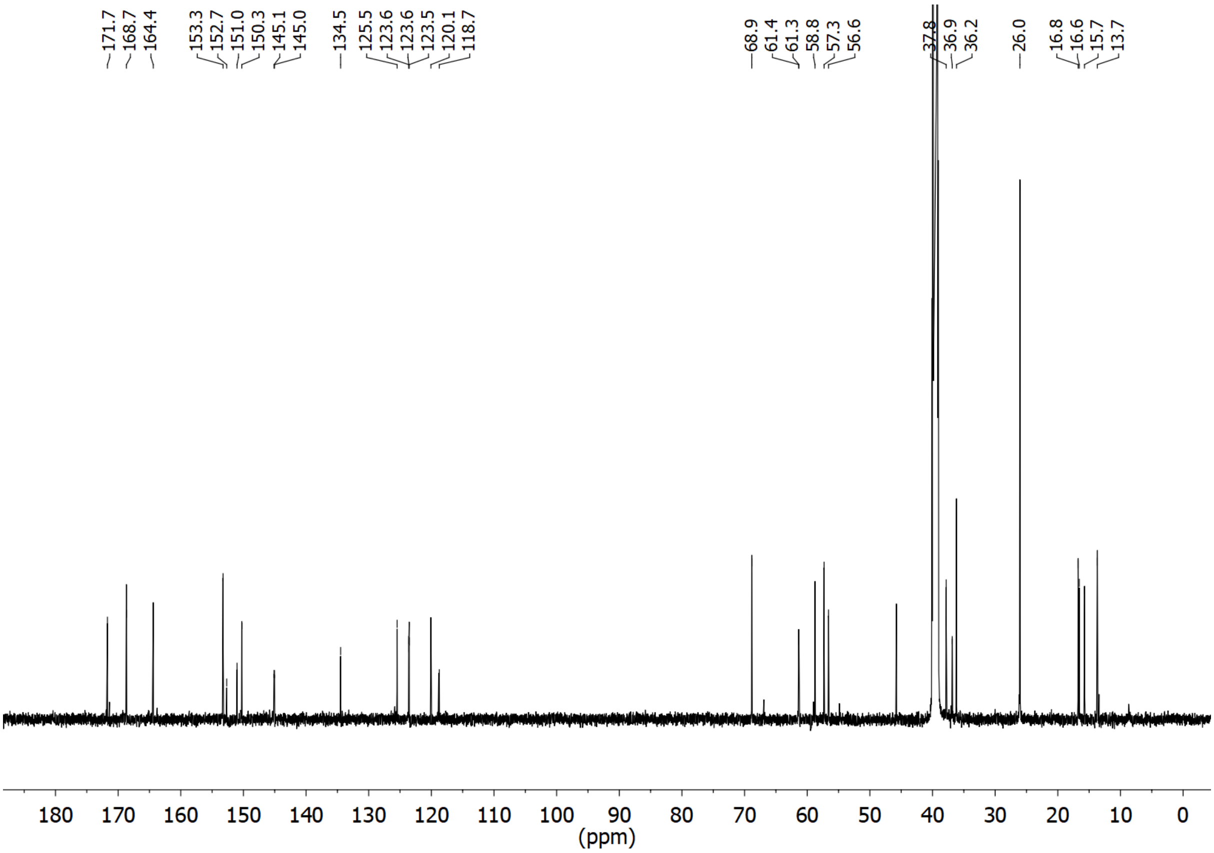

^{1}H and ^{13}C NMR Spectra of compound **22**

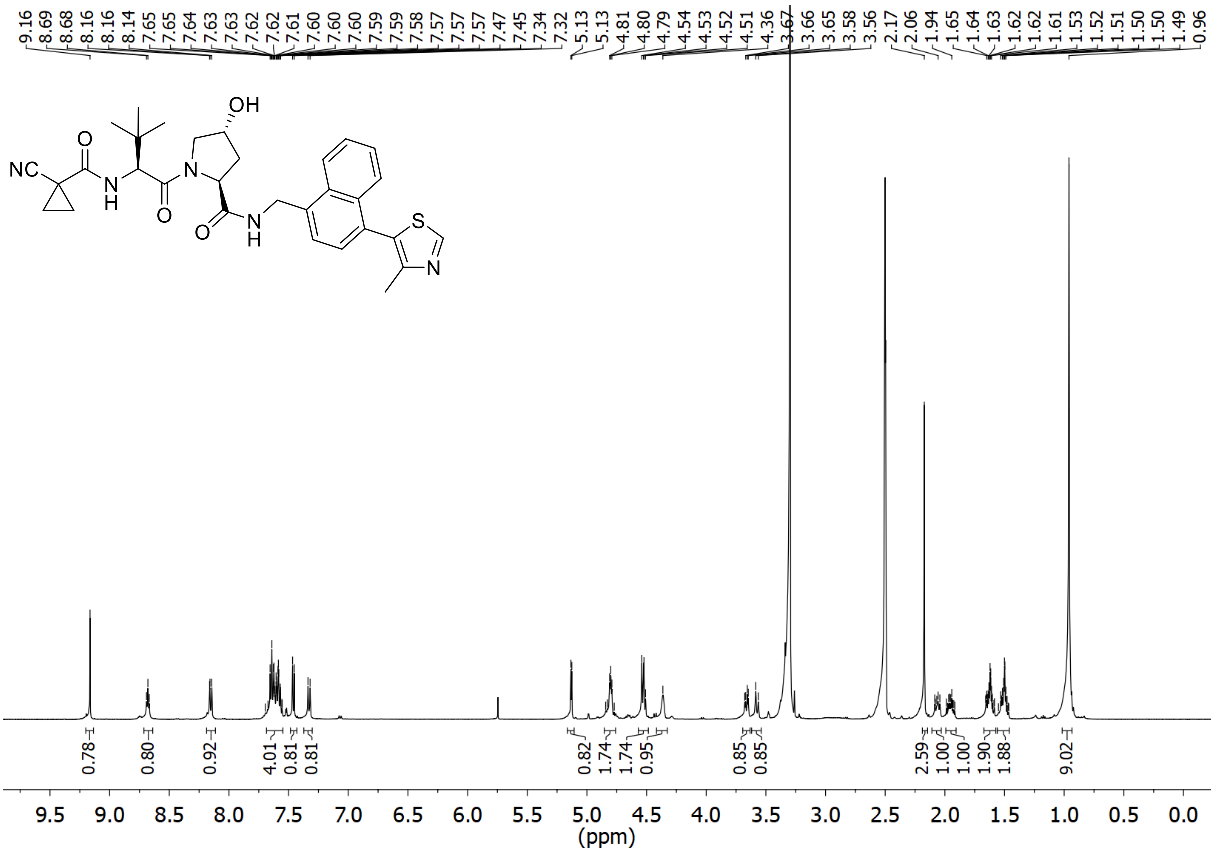

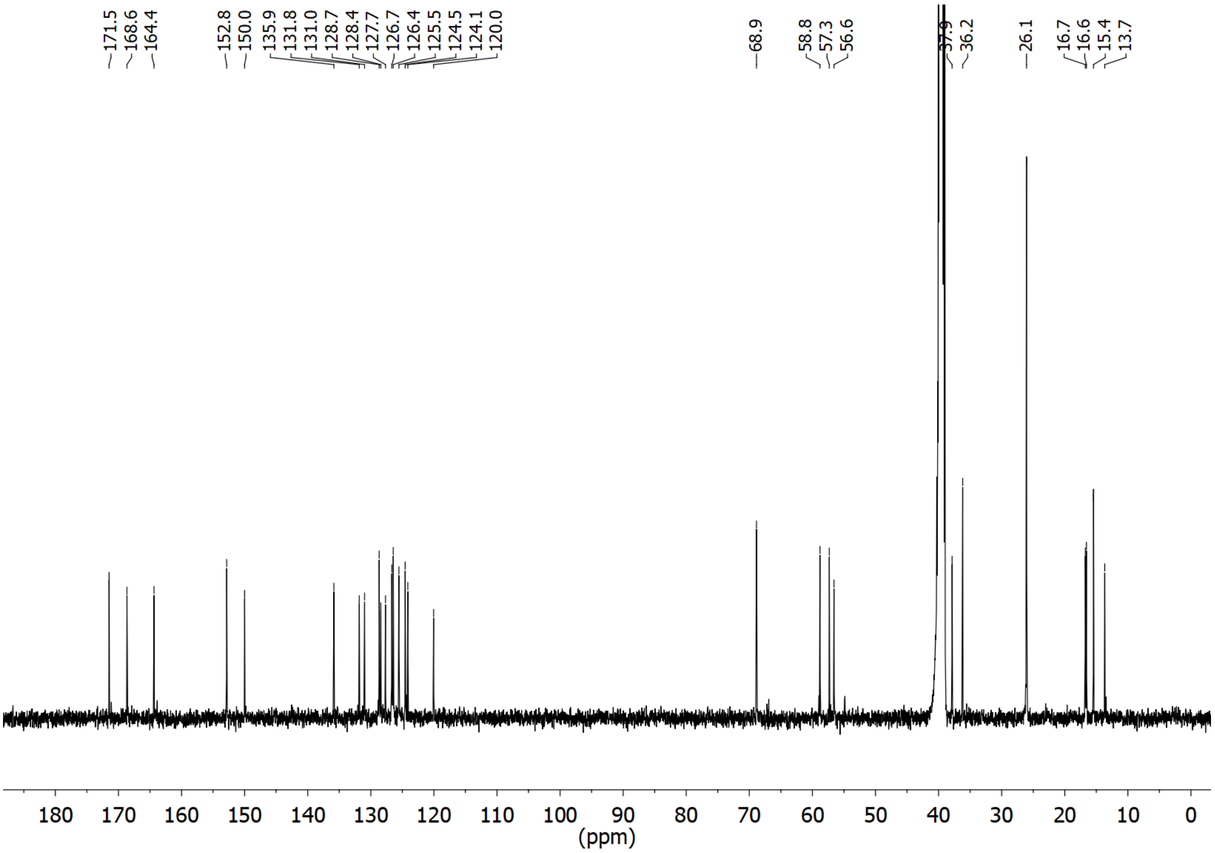

^{1}H and ^{13}C NMR Spectra of compound **23**

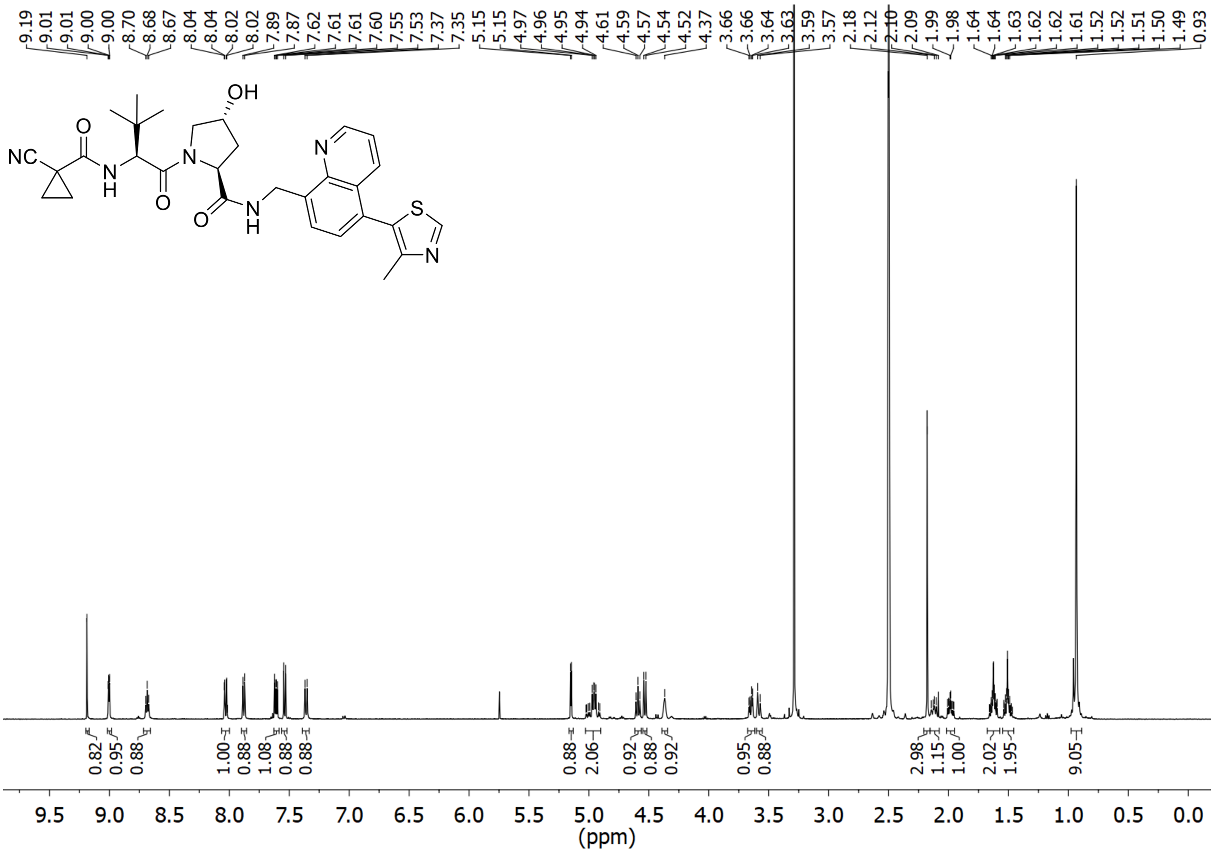

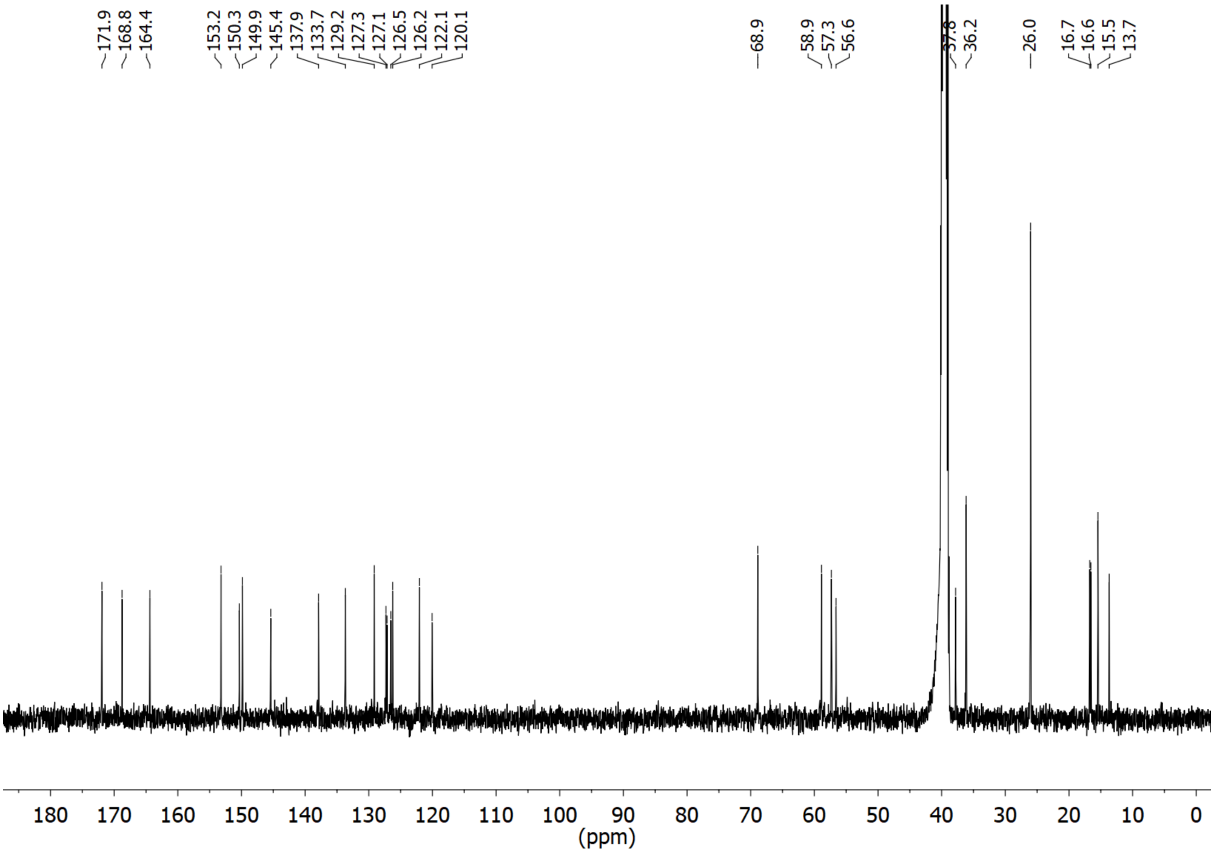

^{1}H and ^{13}C NMR Spectra of compound **24**

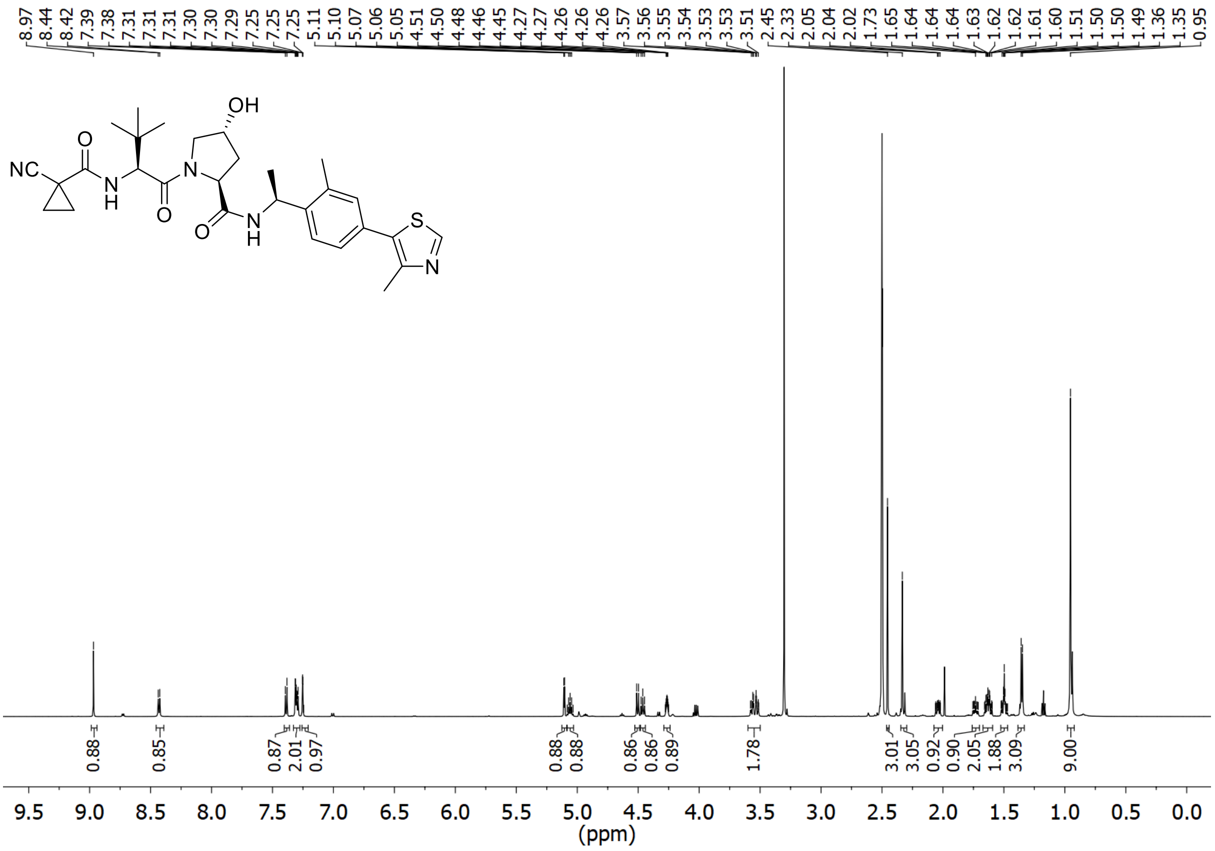

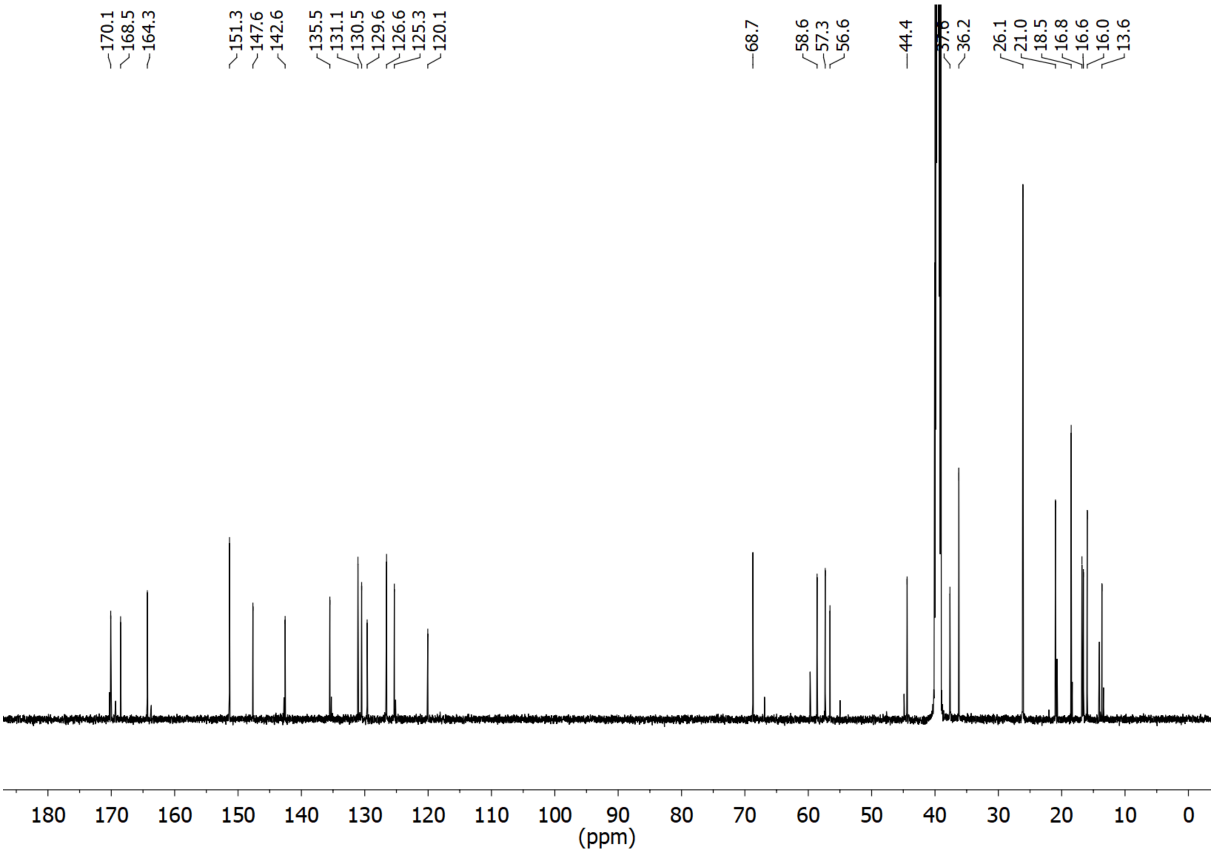

^{1}H and ^{13}C NMR Spectra of compound **25**

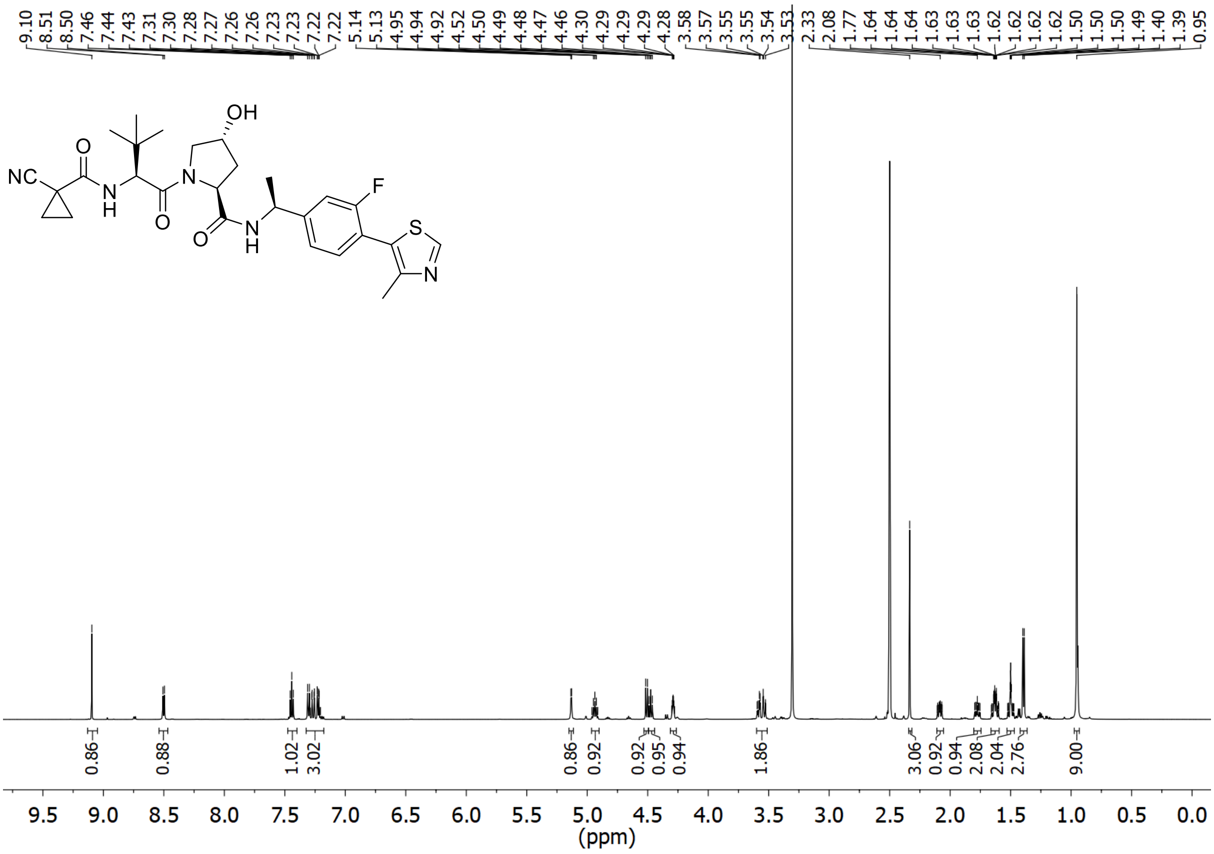

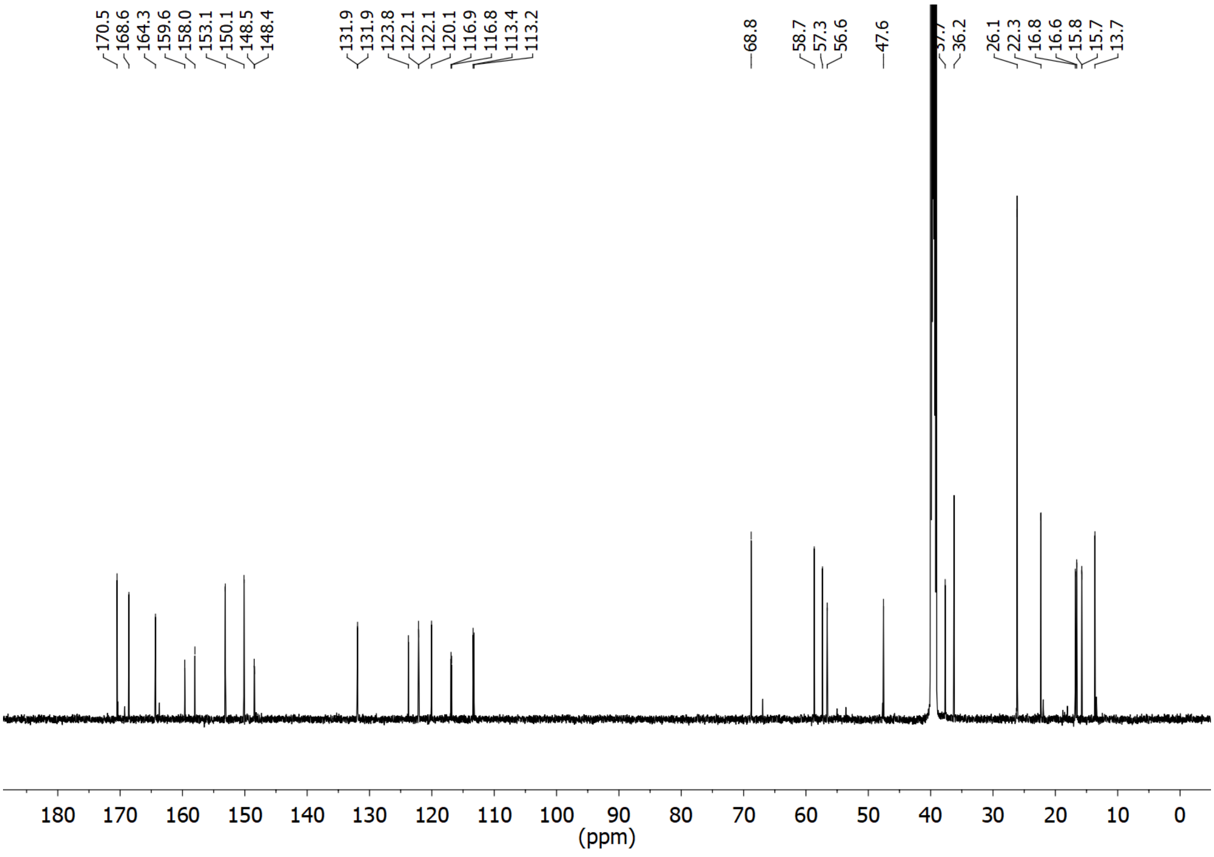

^{1}H and ^{13}C NMR Spectra of compound **26**

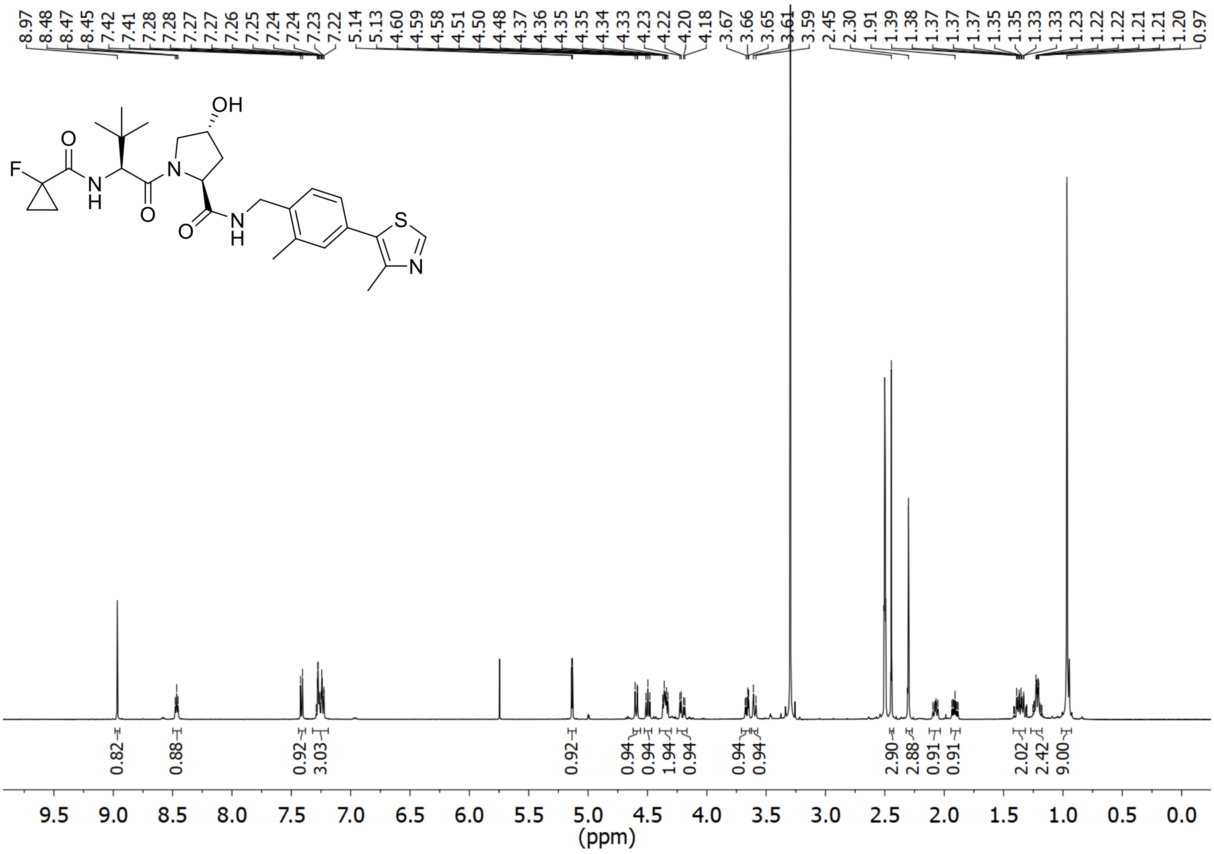

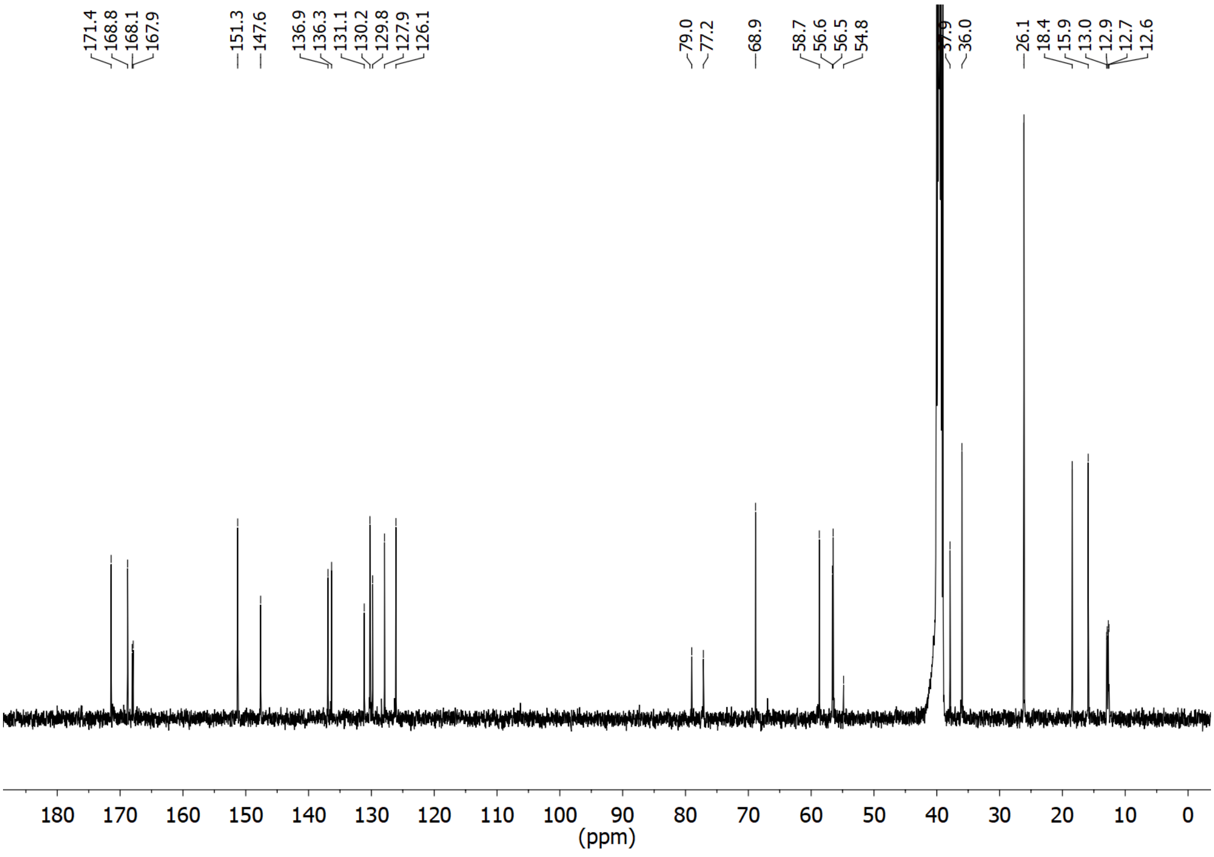

^{1}H and ^{13}C NMR Spectra of compound **27**

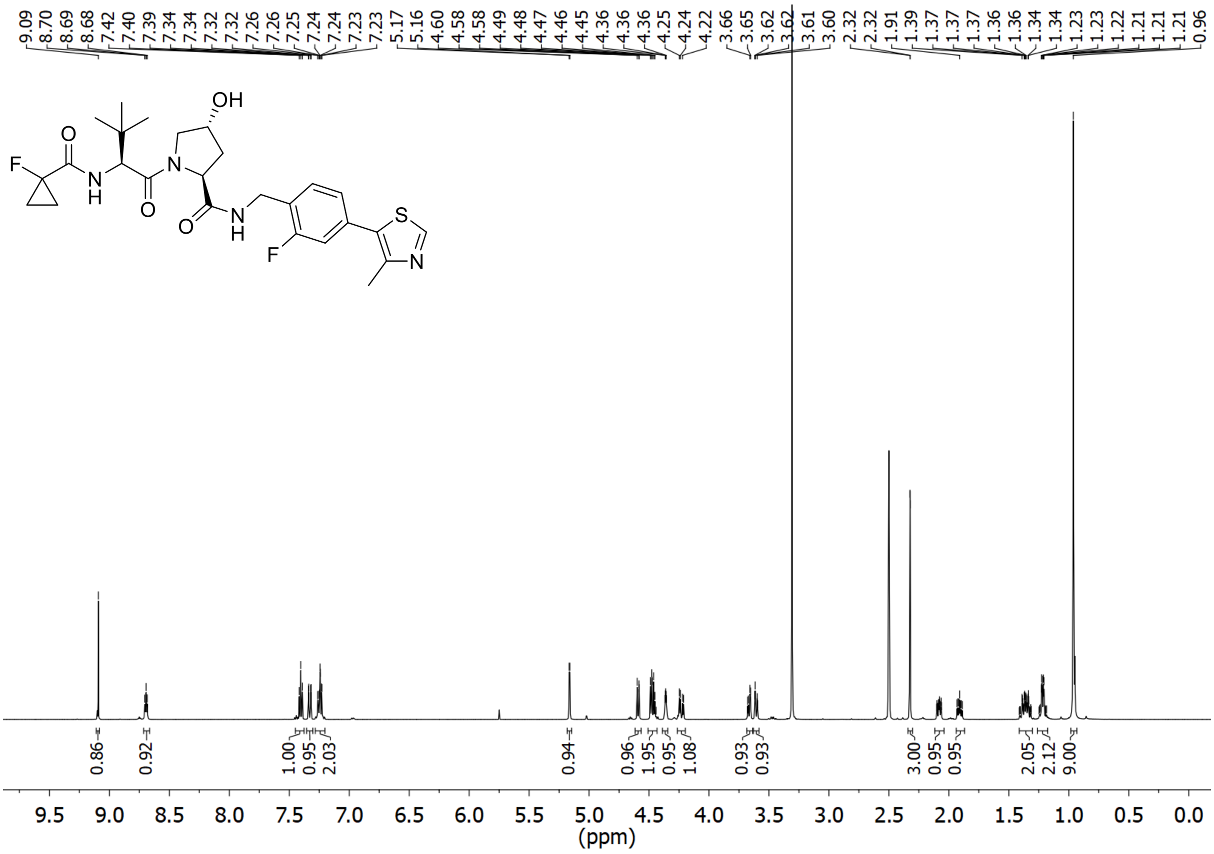

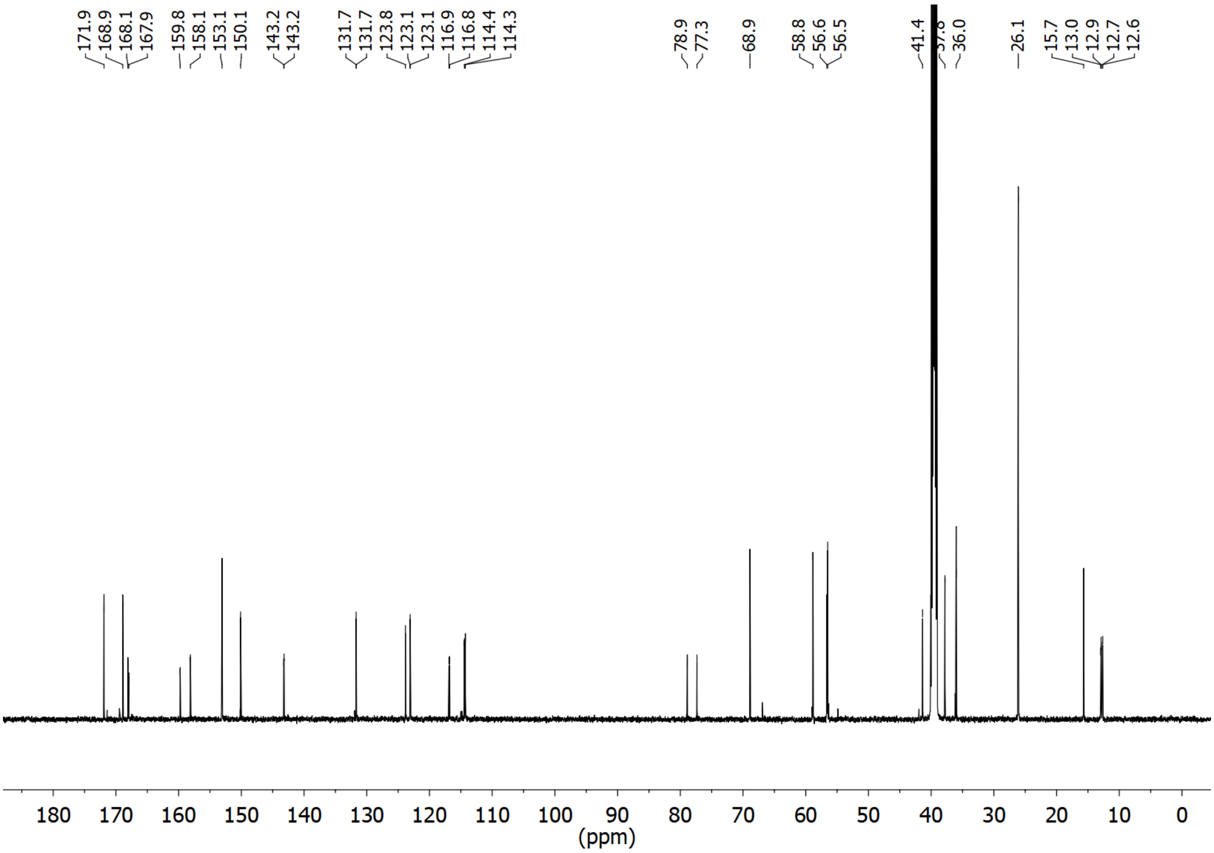

^{1}H and ^{13}C NMR Spectra of compound **28**

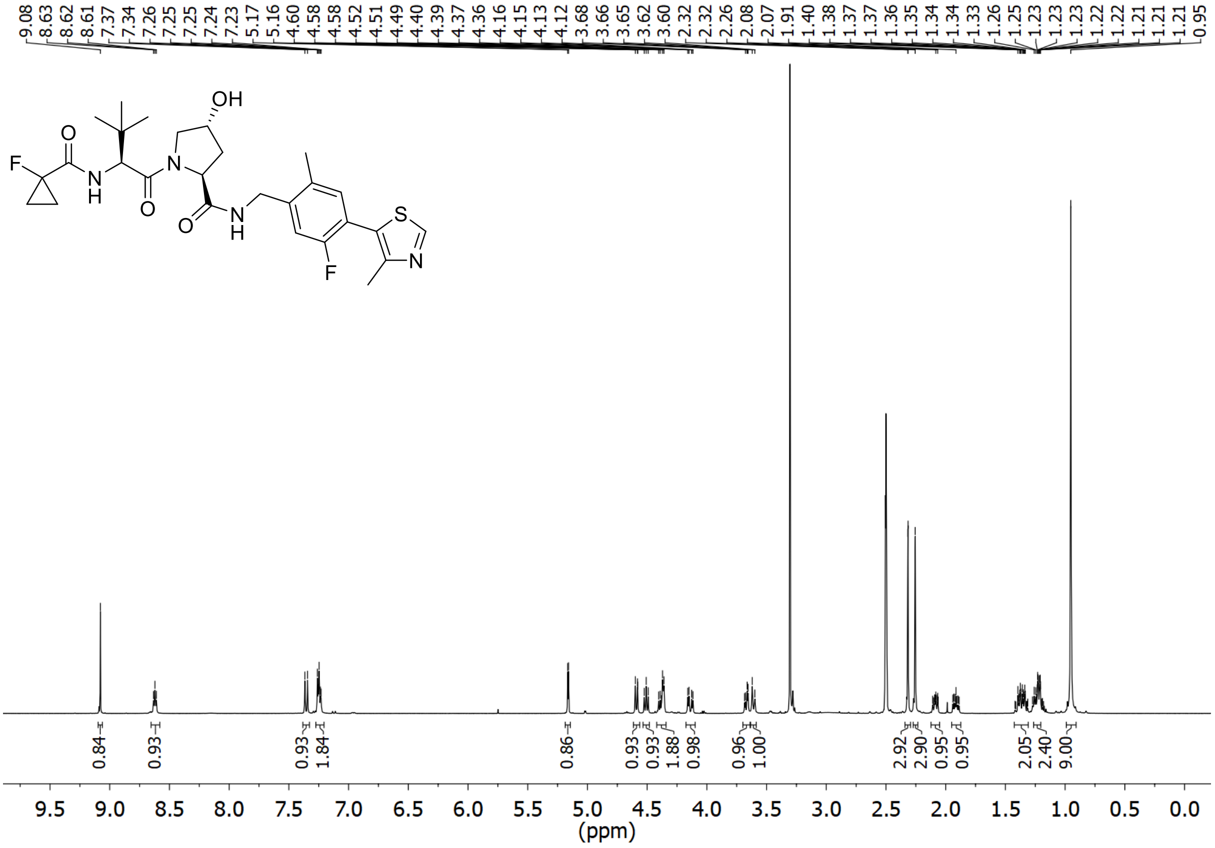

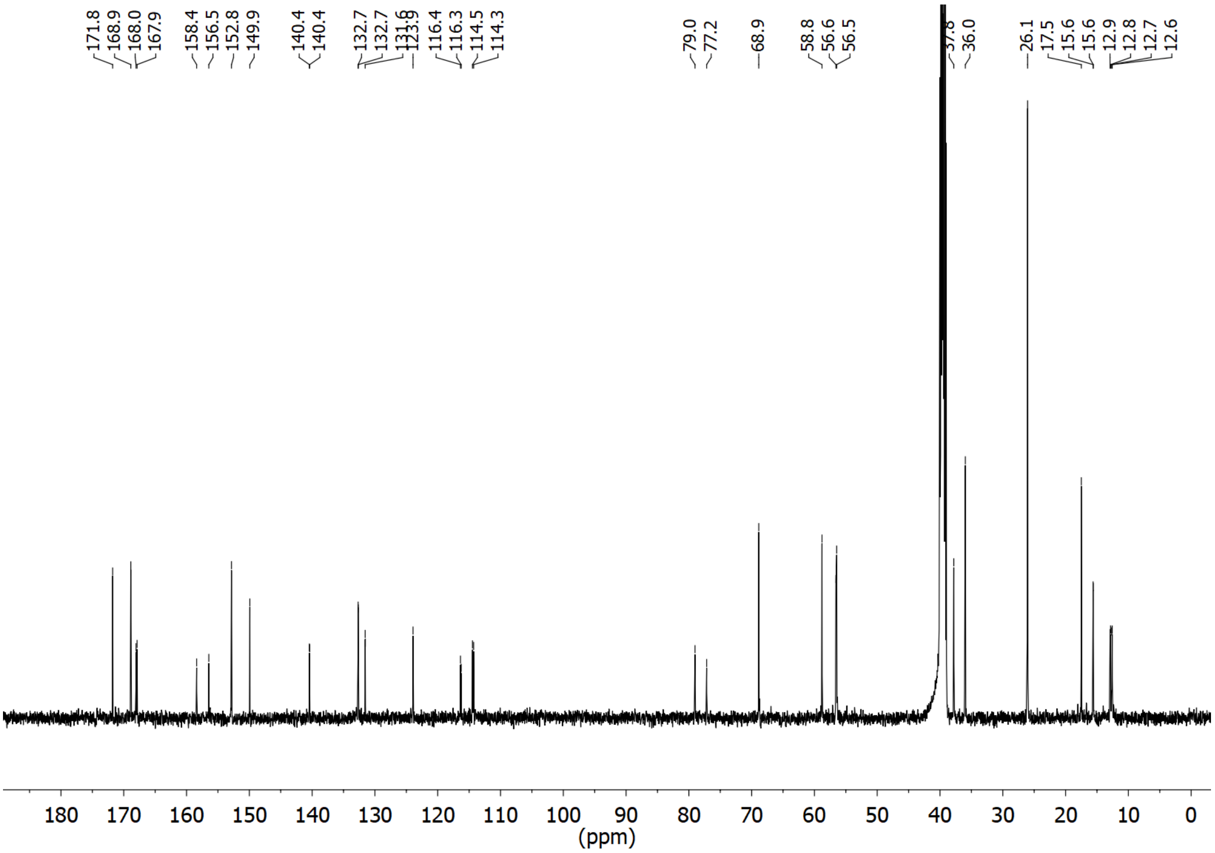

^{1}H and ^{13}C NMR Spectra of compound **29**

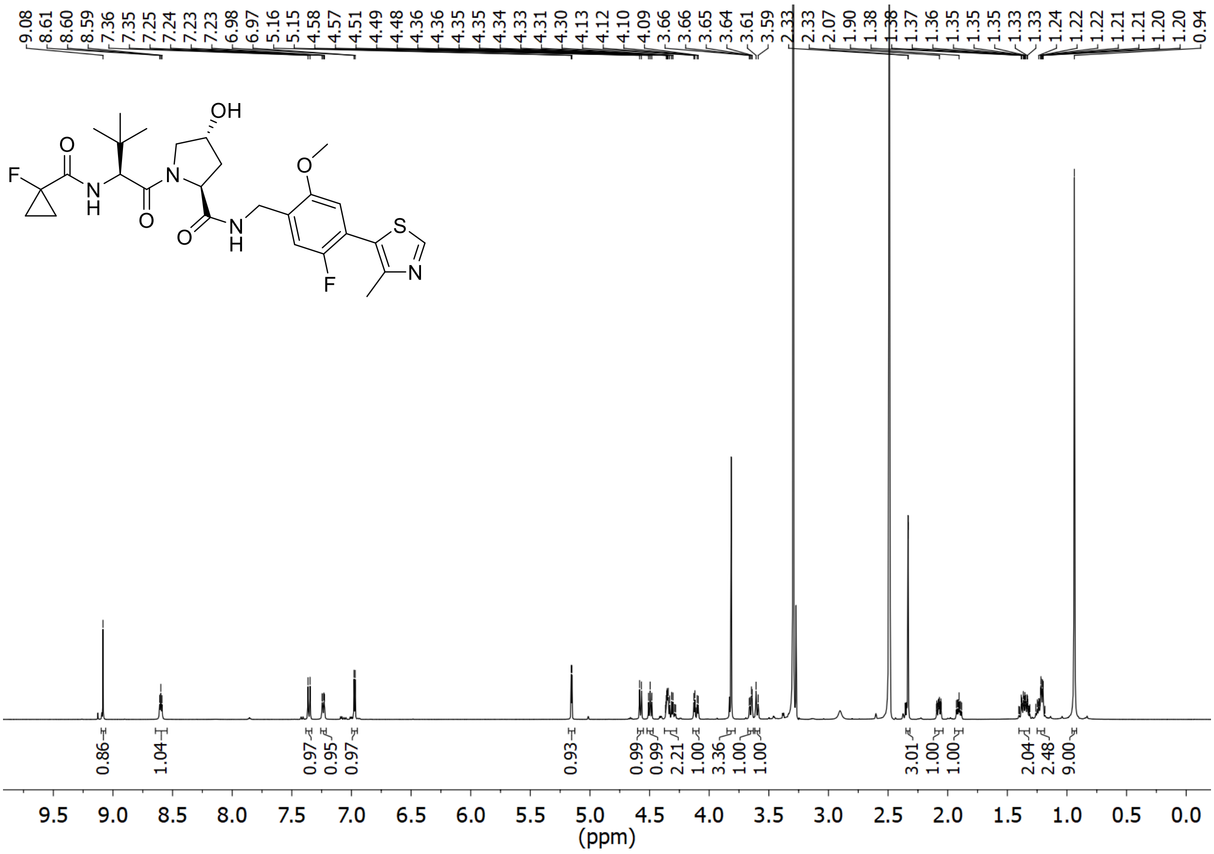

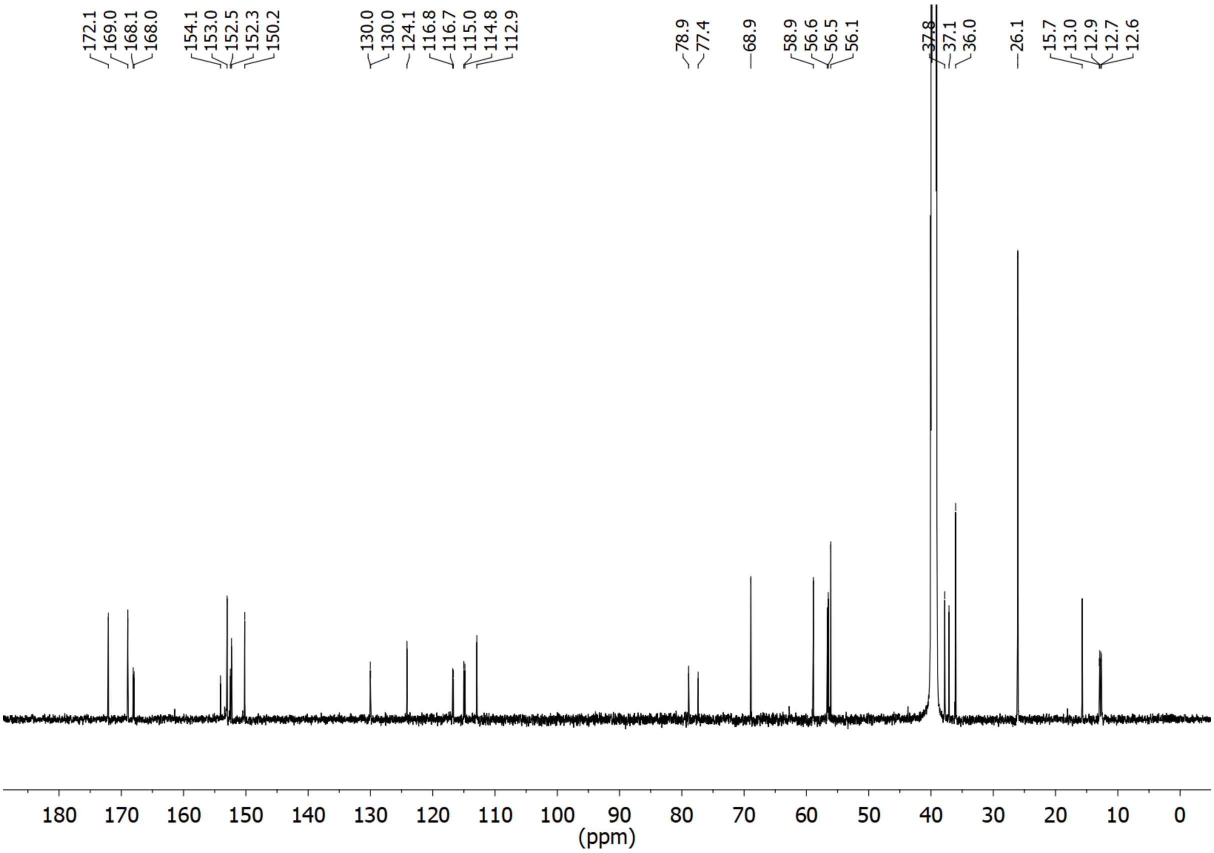

^{1}H and ^{13}C NMR Spectra of compound **30**

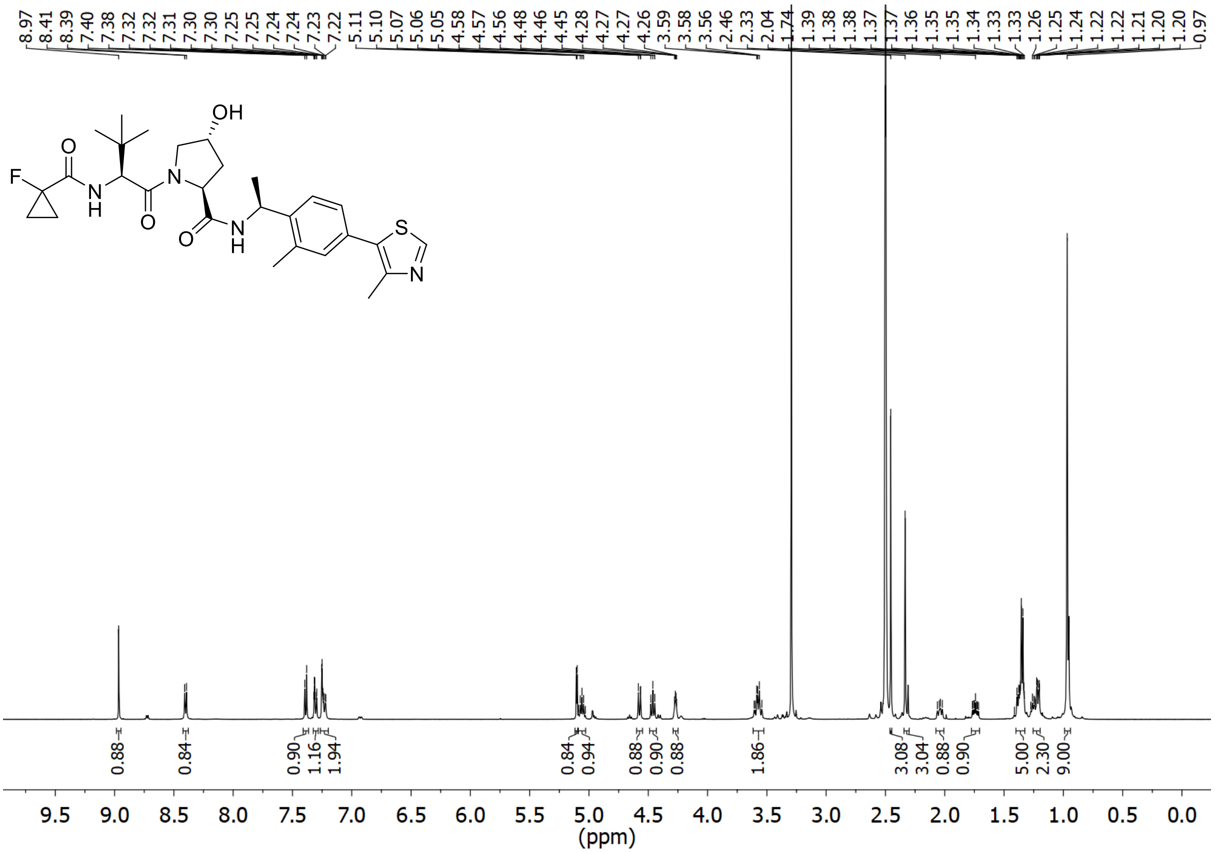

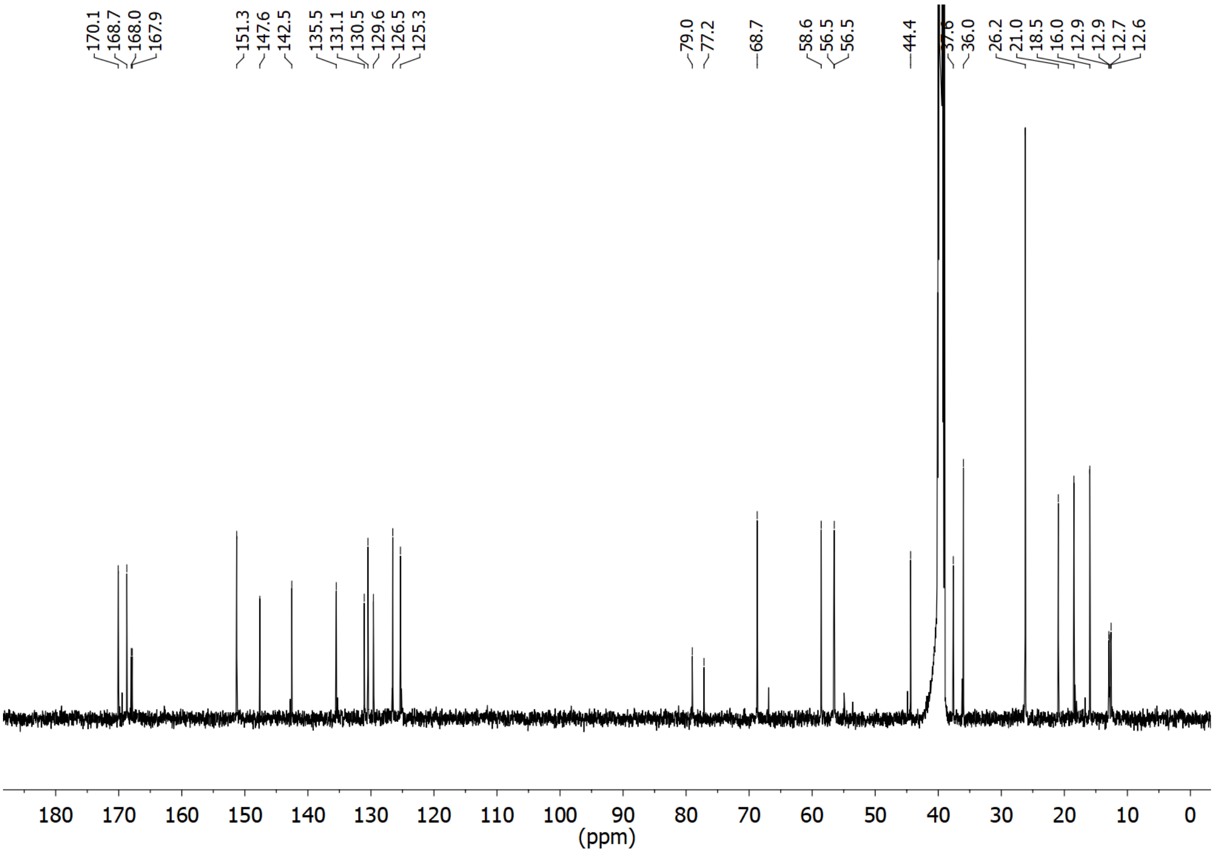

^{1}H and ^{13}C NMR Spectra of compound **31**

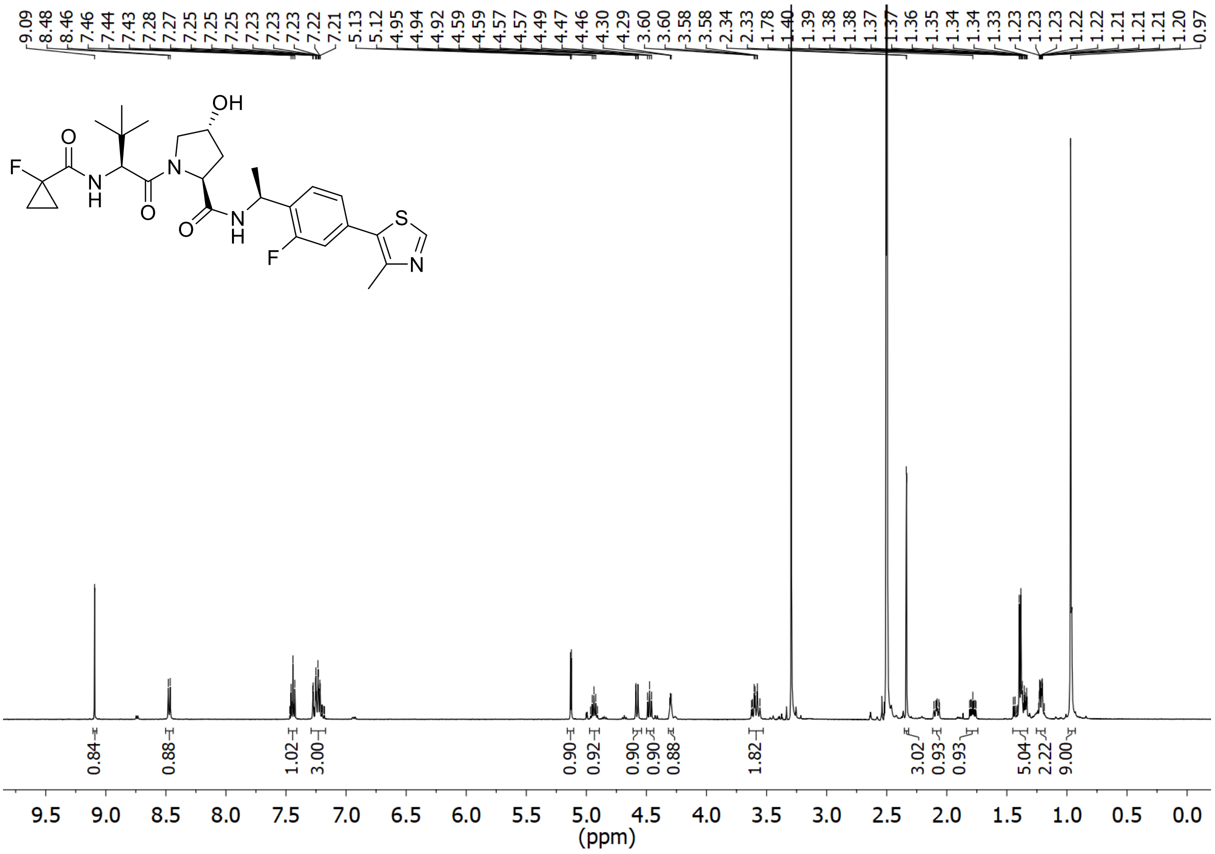

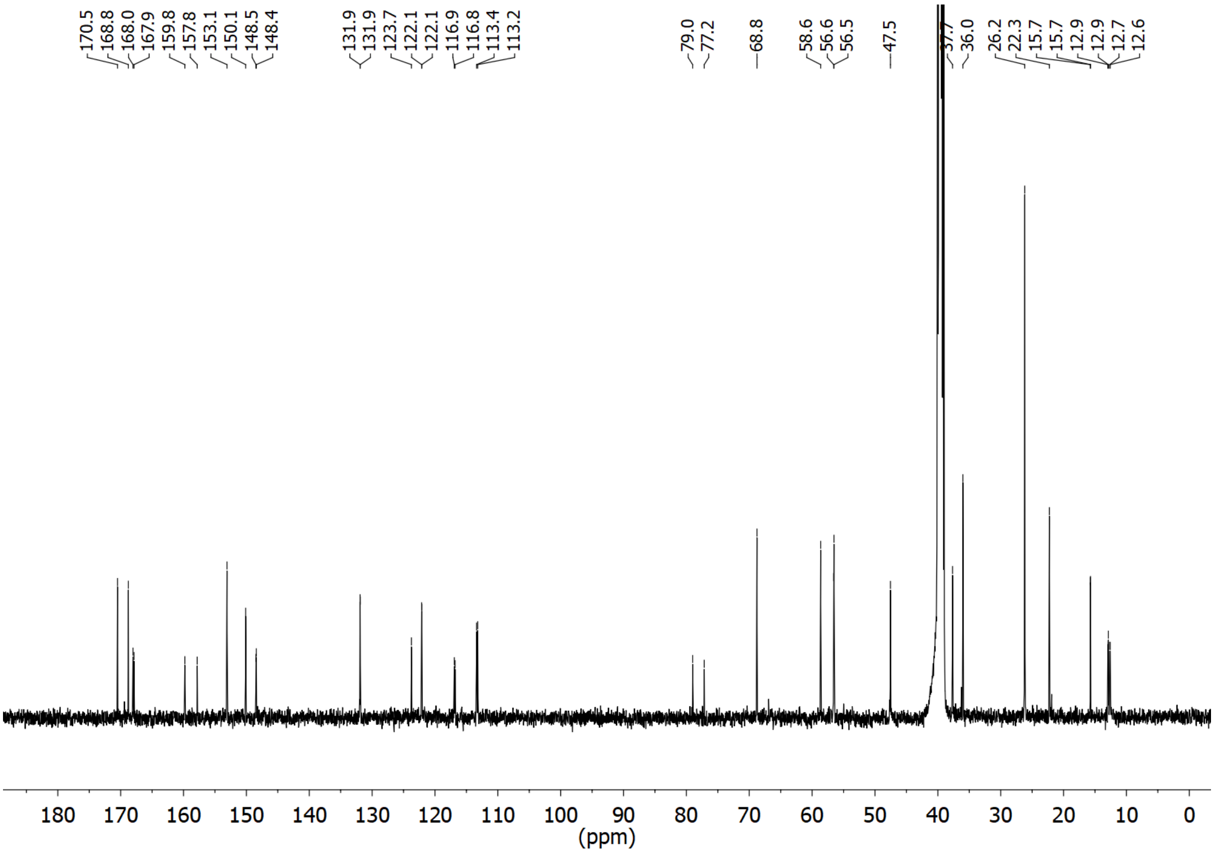

^{1}H and ^{13}C NMR Spectra of compound **32**

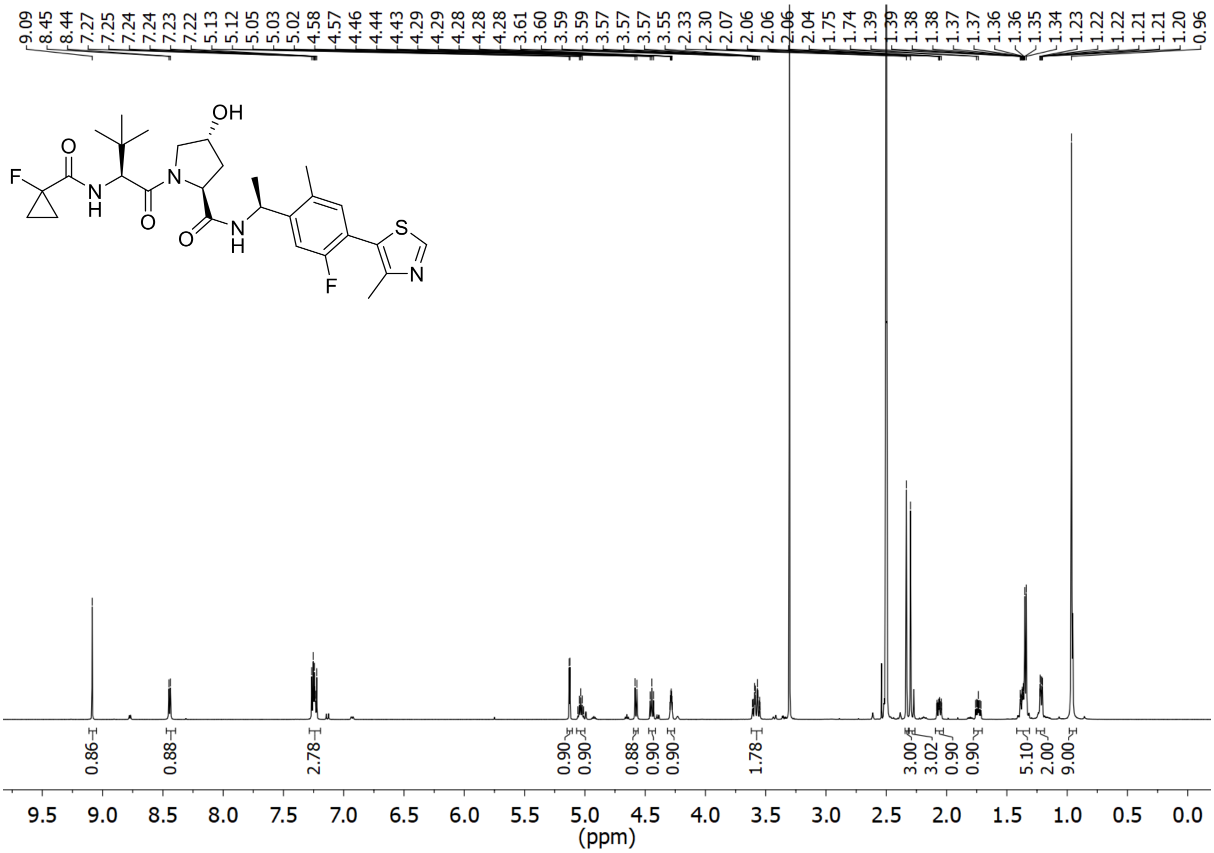

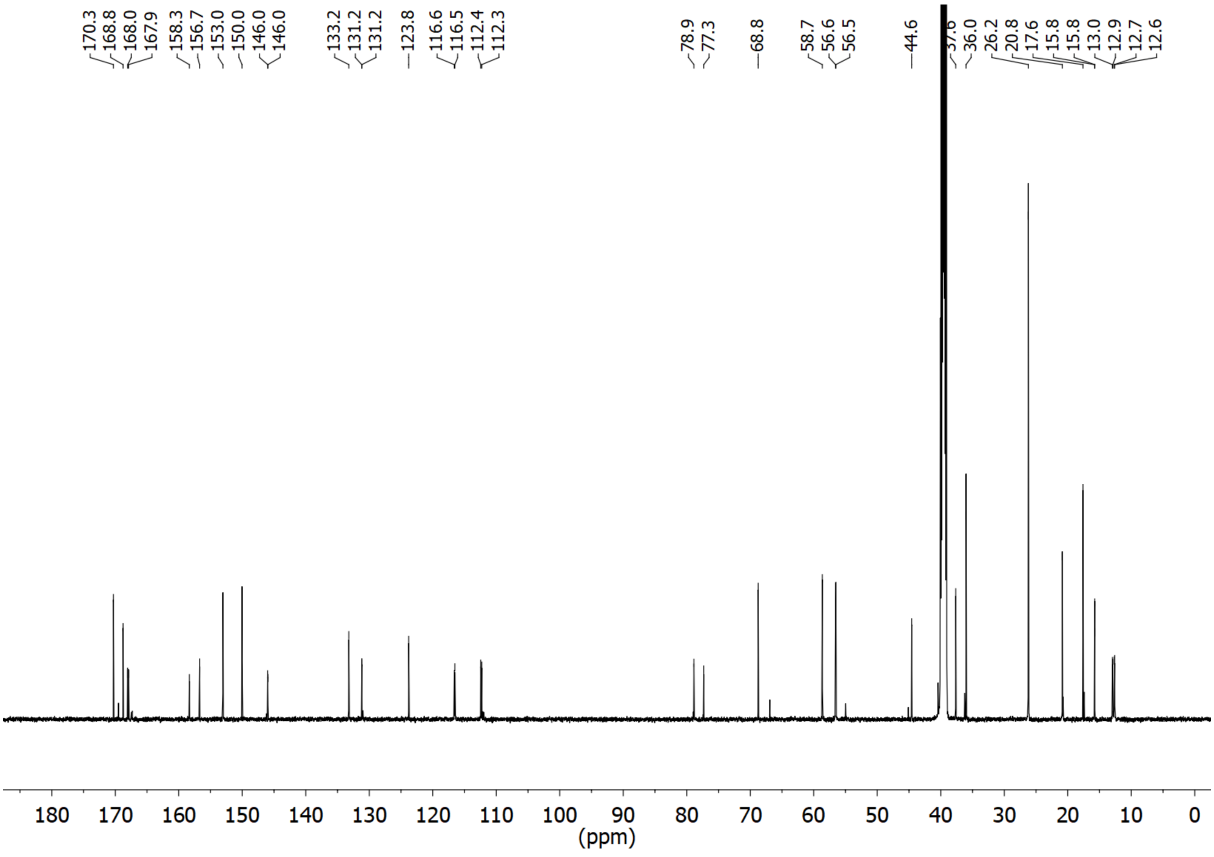

^{1}H and ^{13}C NMR Spectra of compound **33**

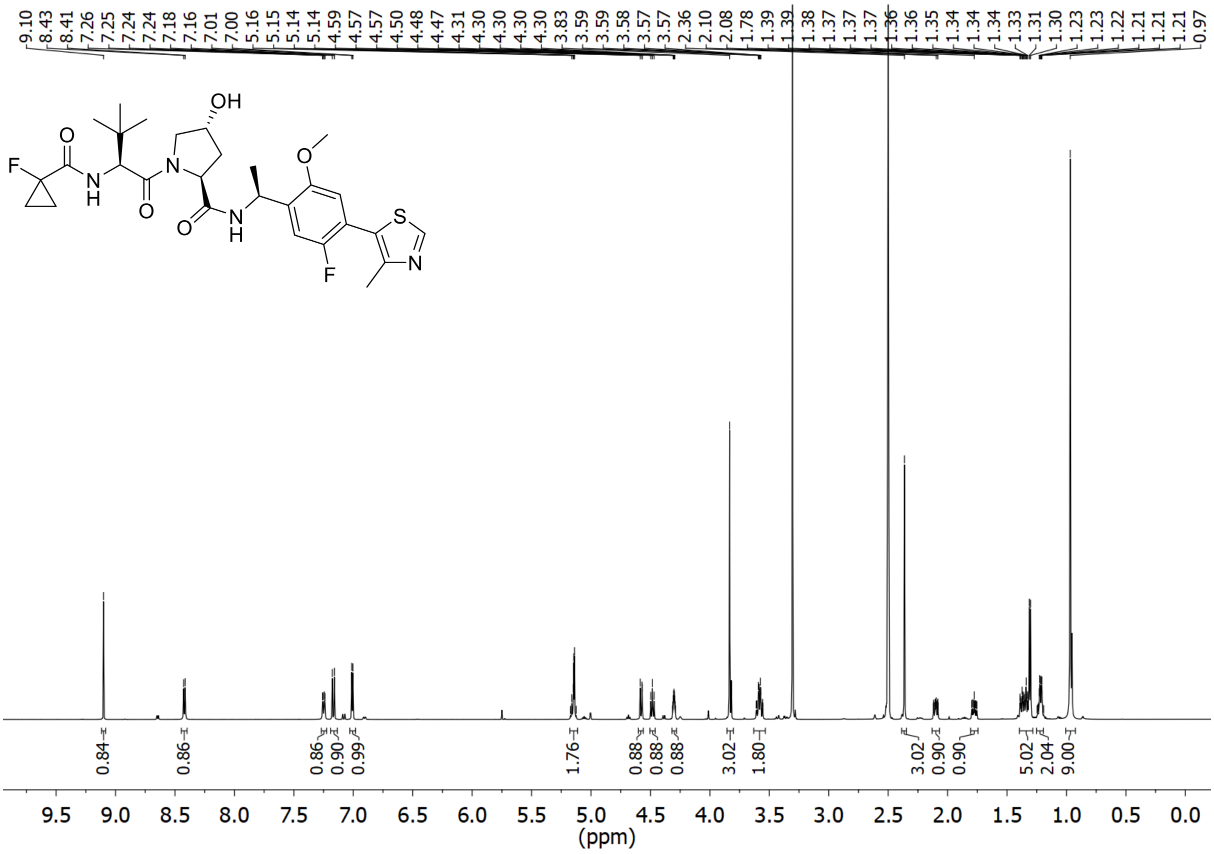

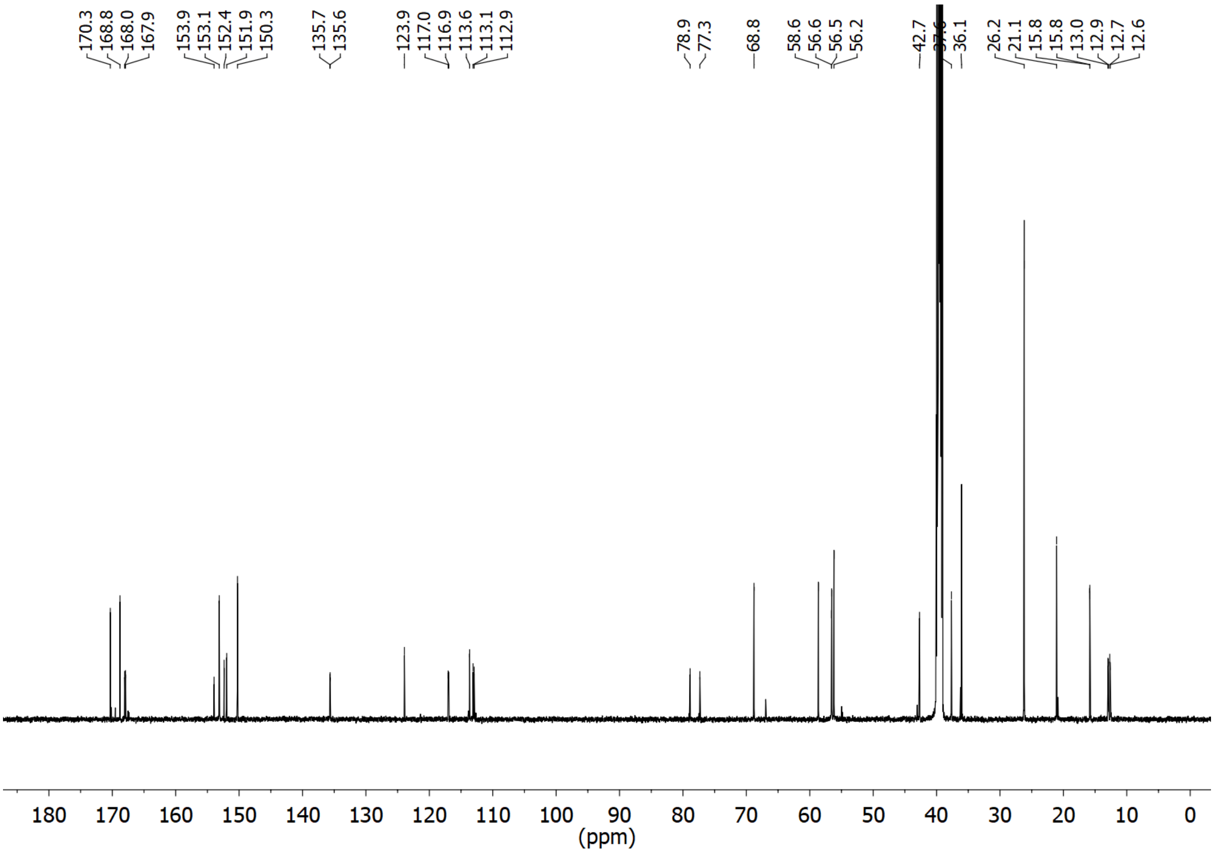

^{1}H and ^{13}C NMR Spectra of compound **34**

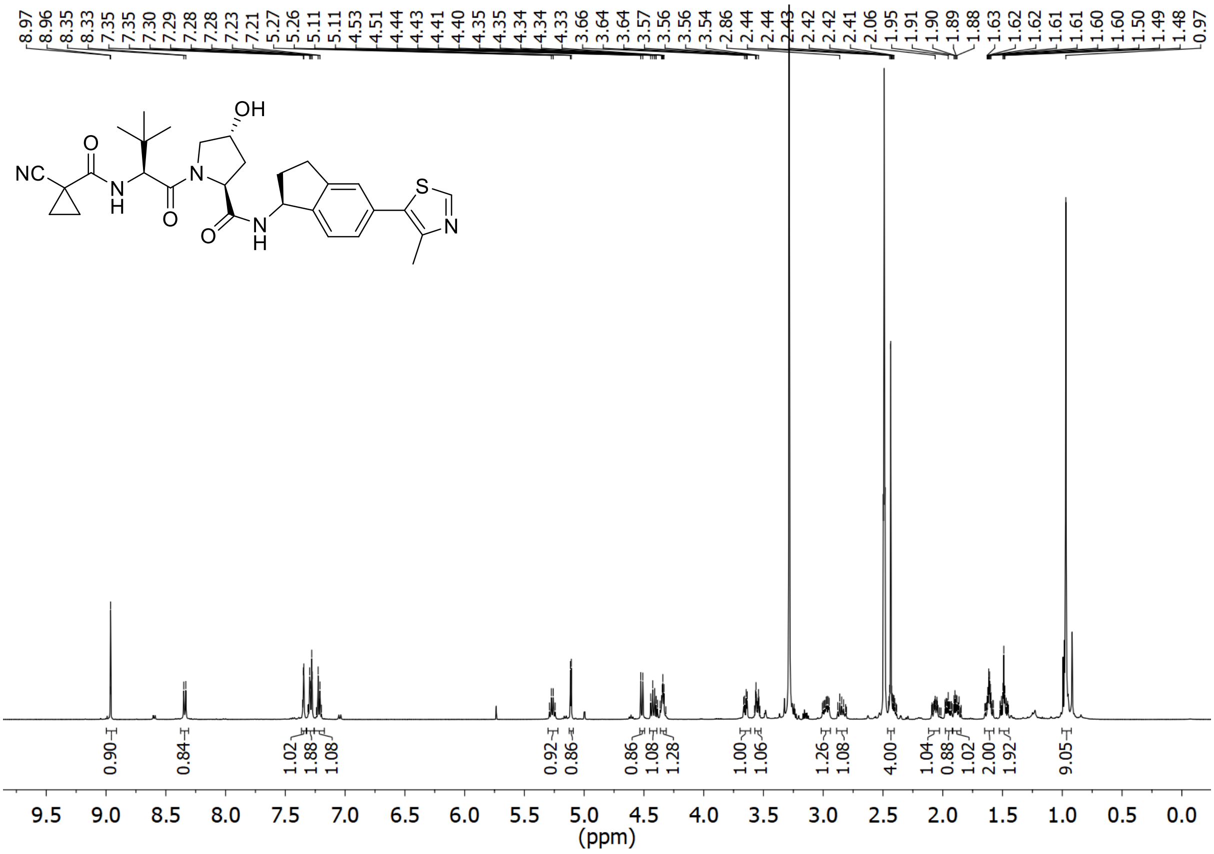

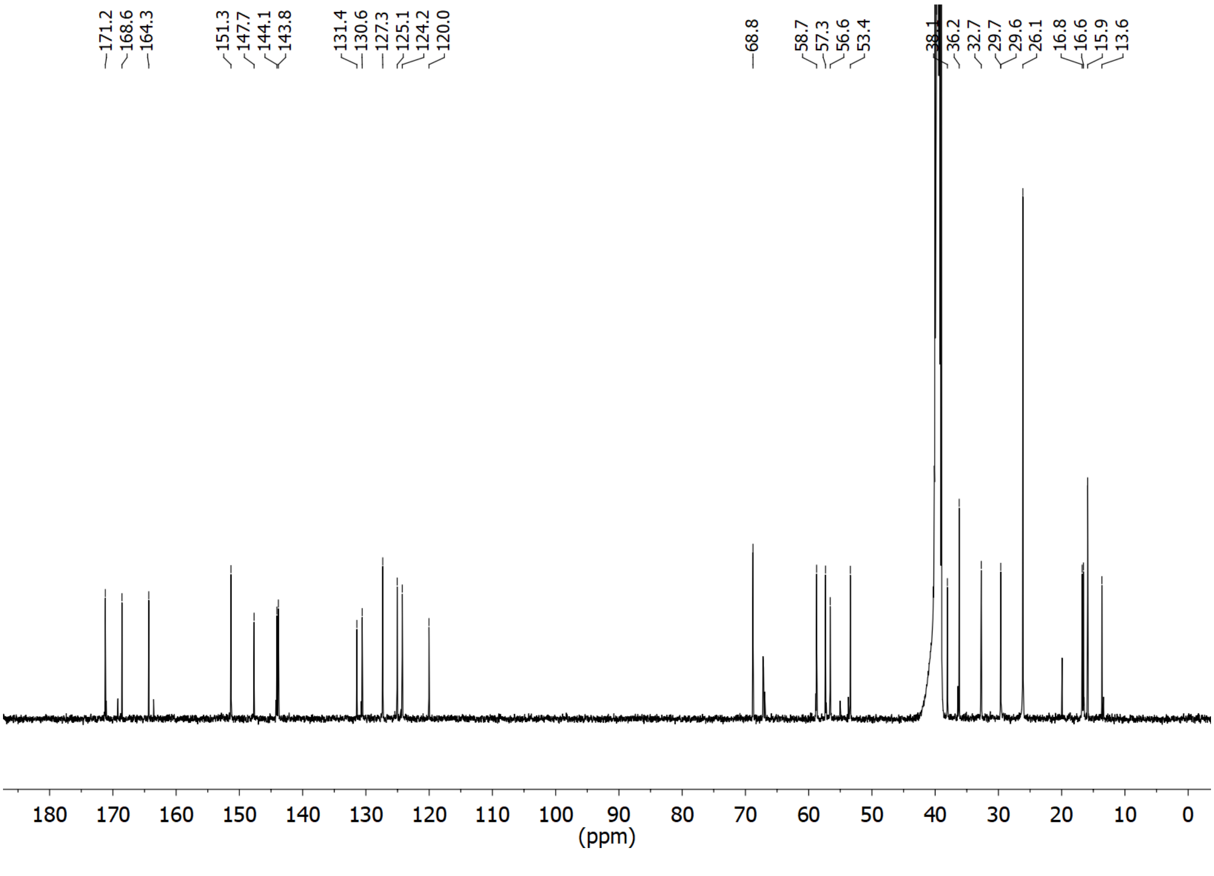

^{1}H and ^{13}C NMR Spectra of compound **35**

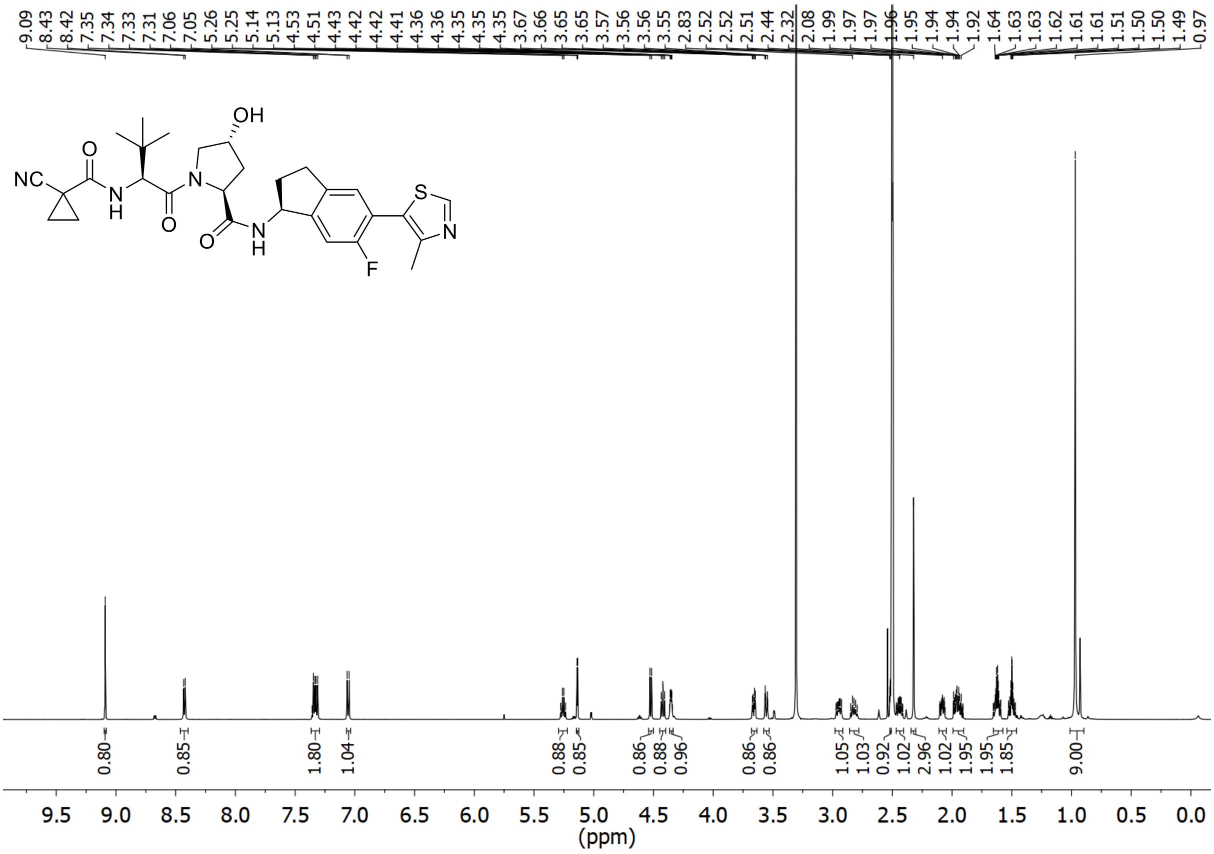

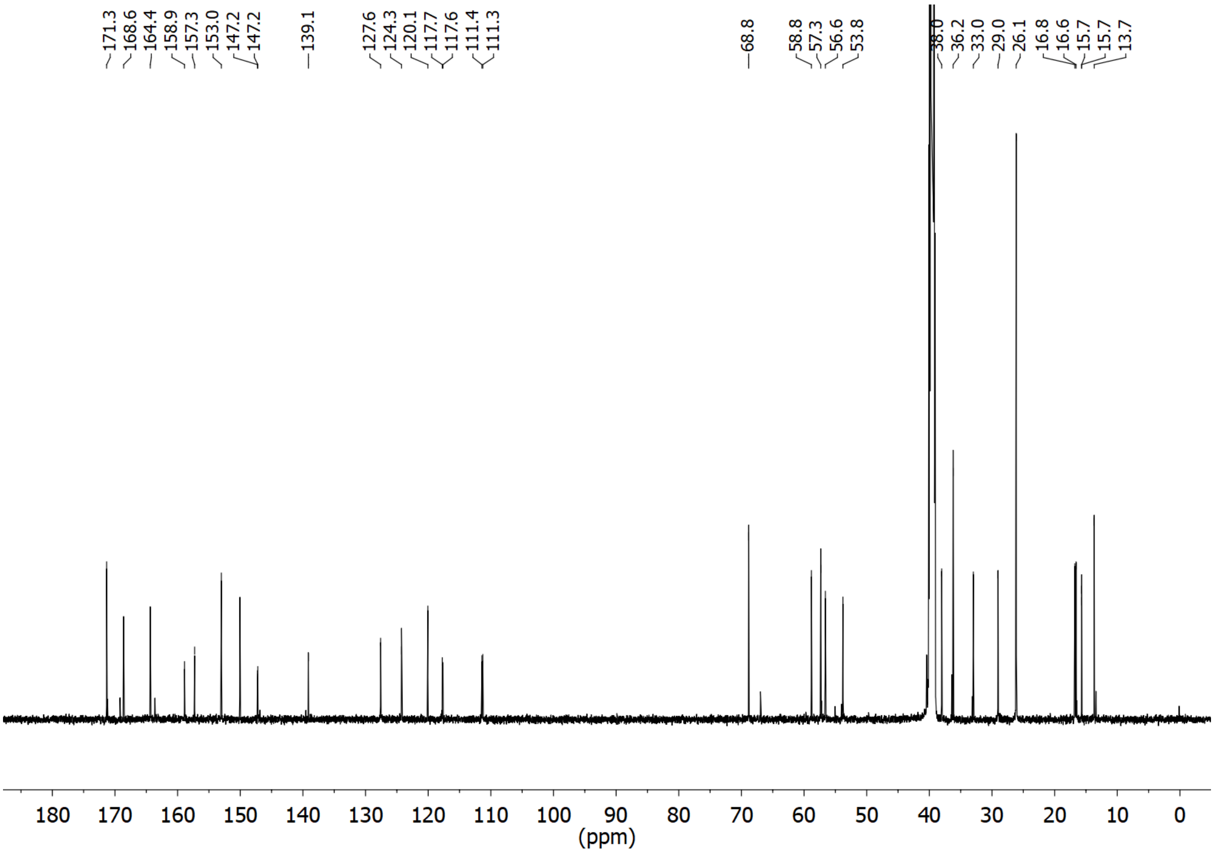

^{1}H and ^{13}C NMR Spectra of compound **36**

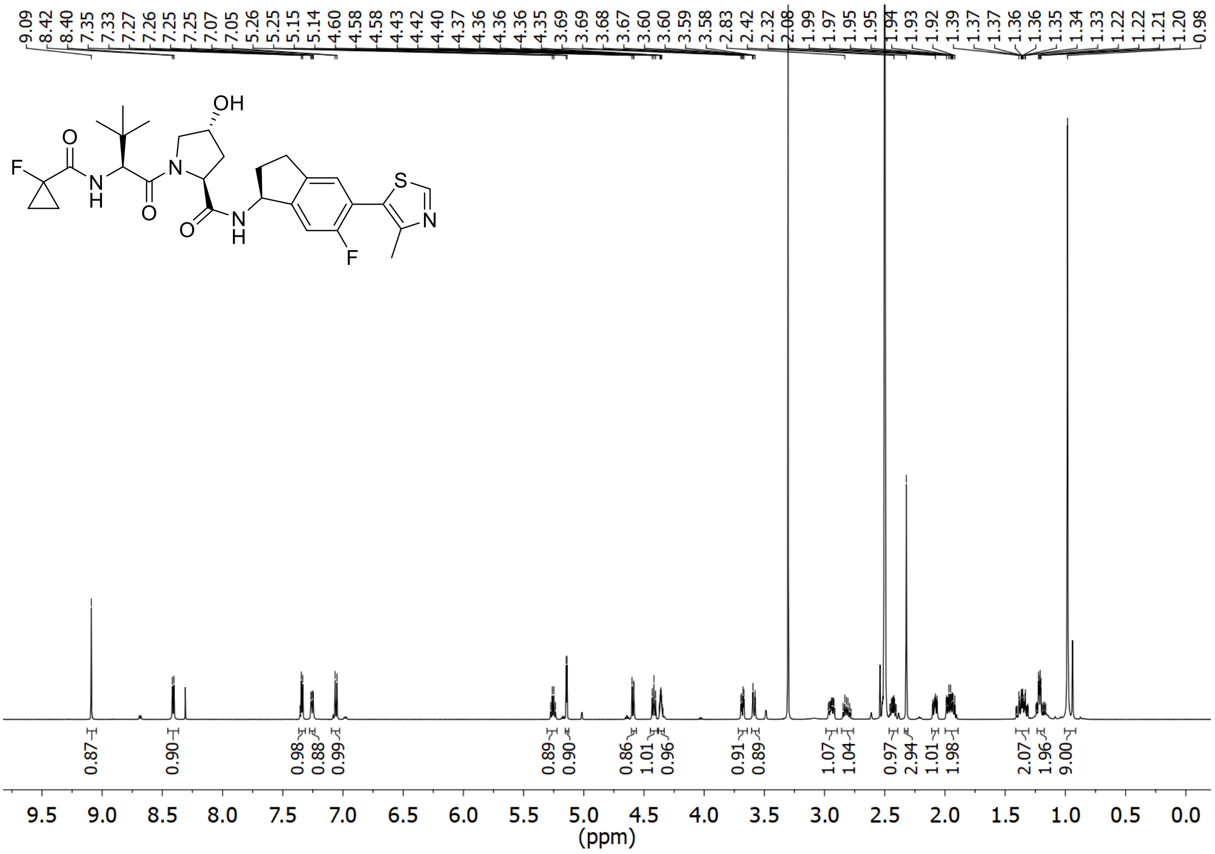

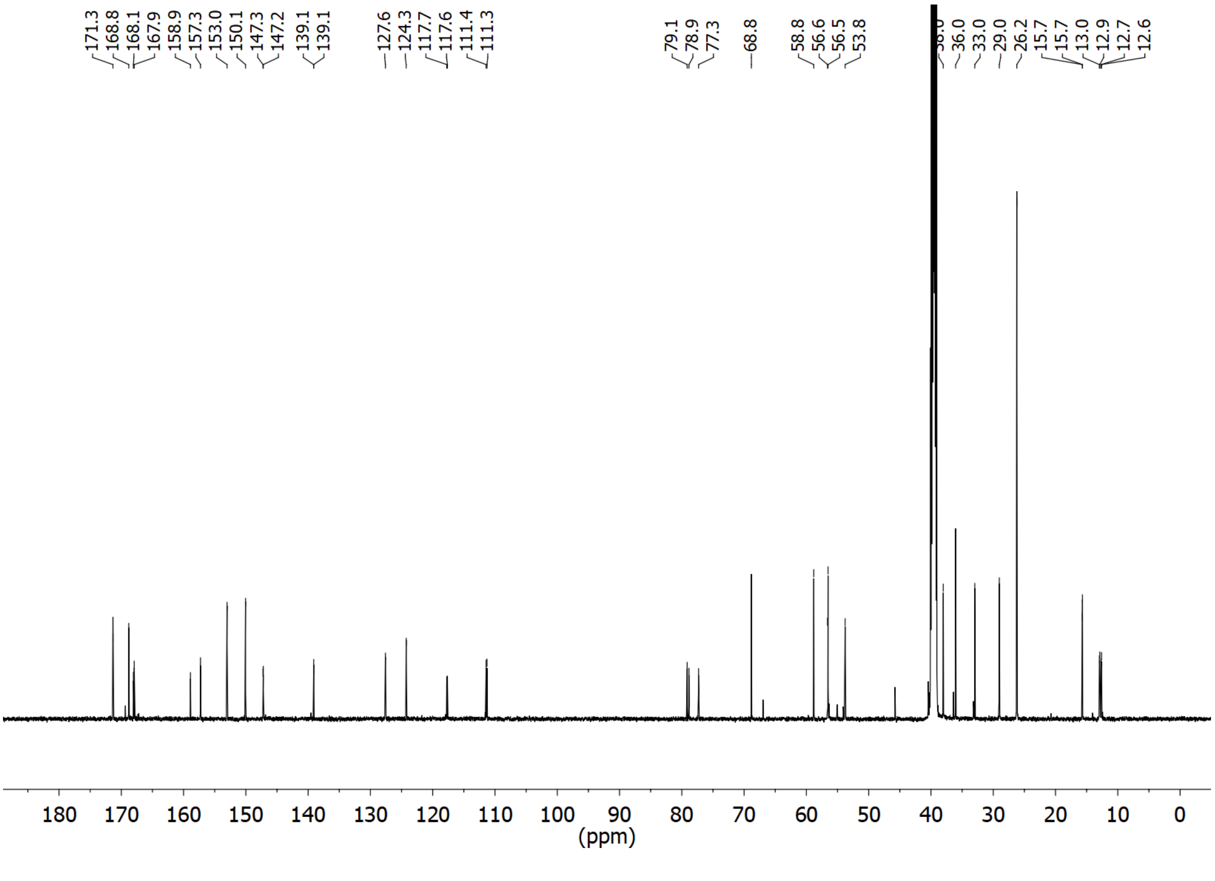

^{1}H and ^{13}C NMR Spectra of compound **37**

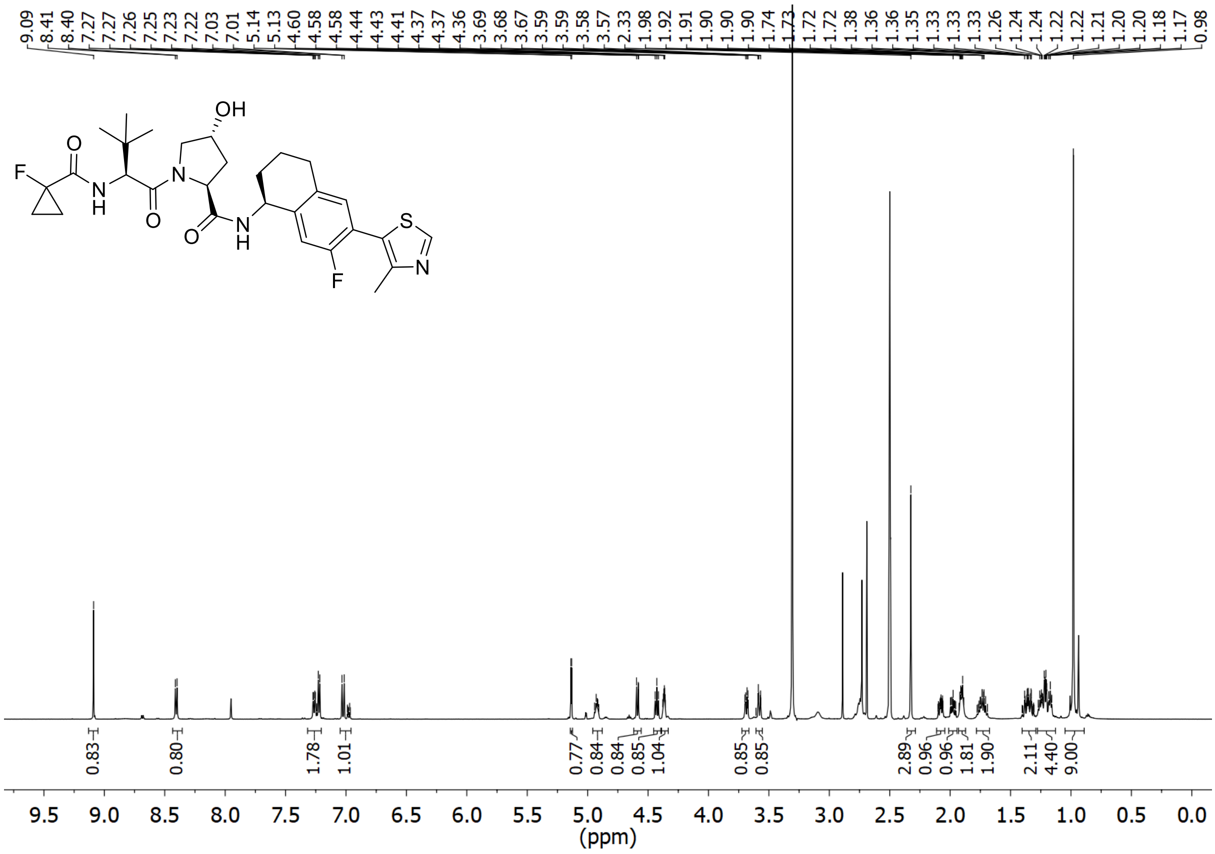

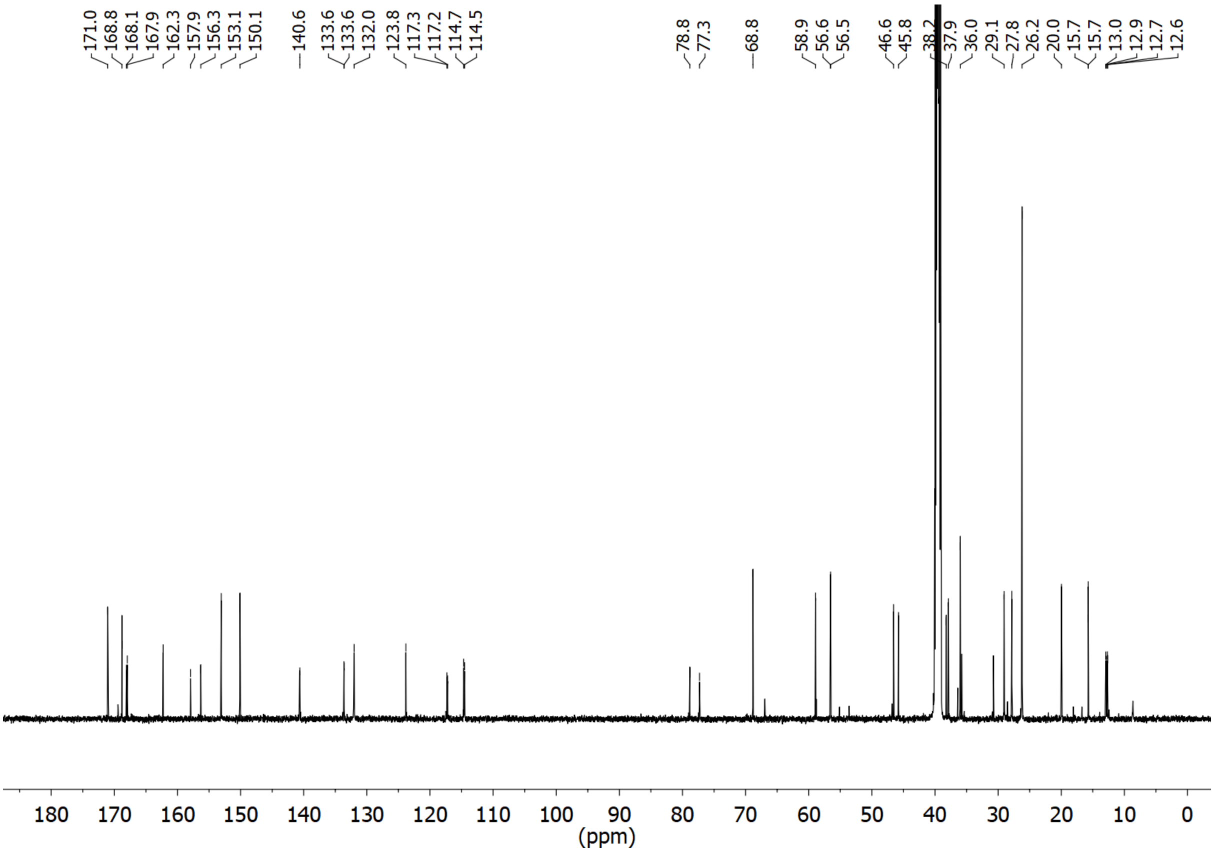

Further NMR Spectra of Compound 14

^{13}C DEPT-135

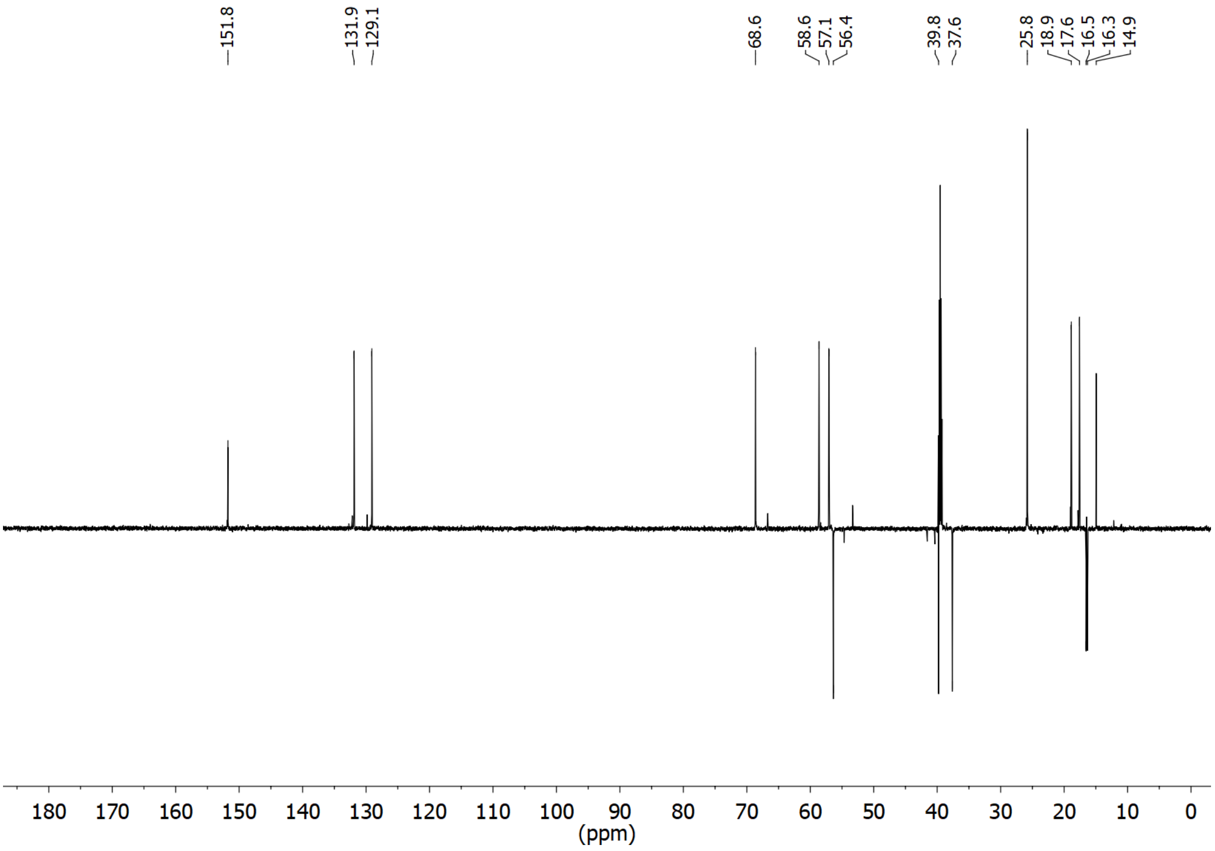

^{1}H-^{13}C HSQC

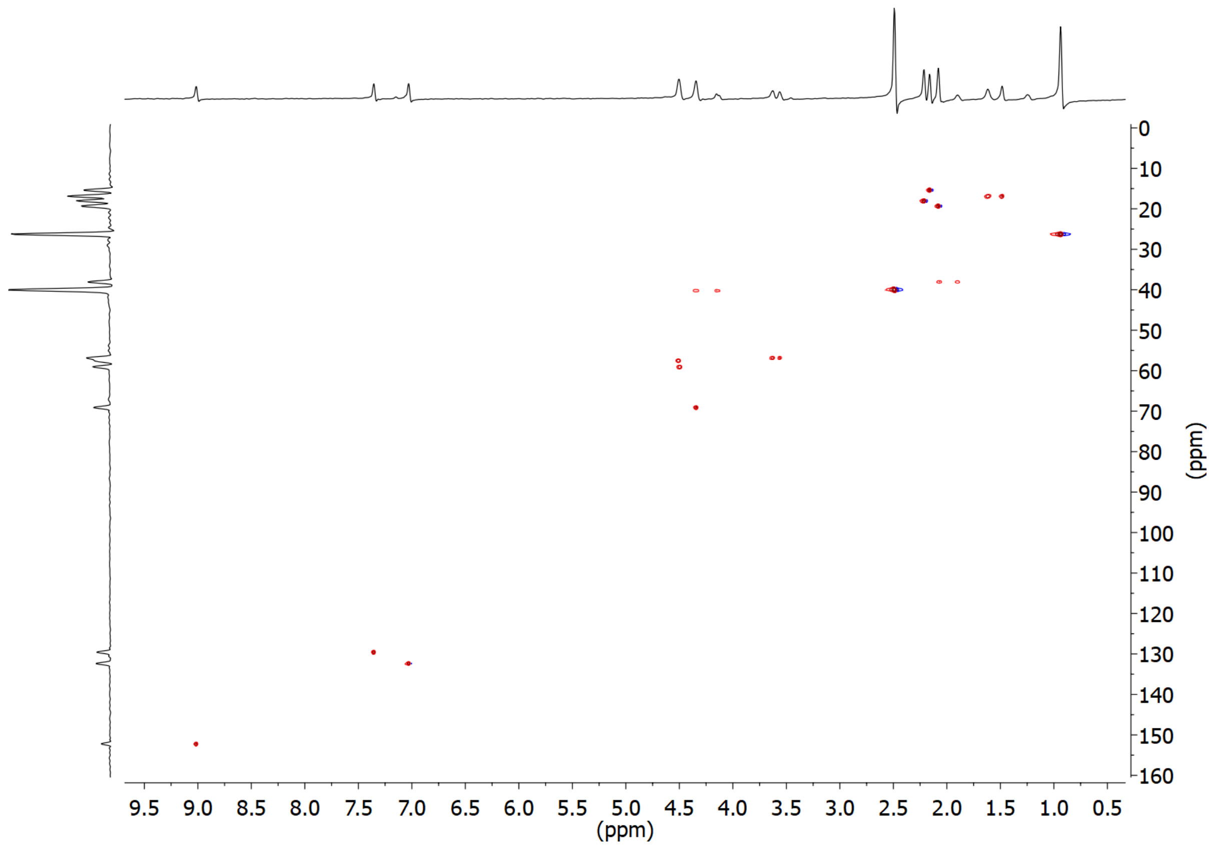

^{1}H-^{13}C HMBC

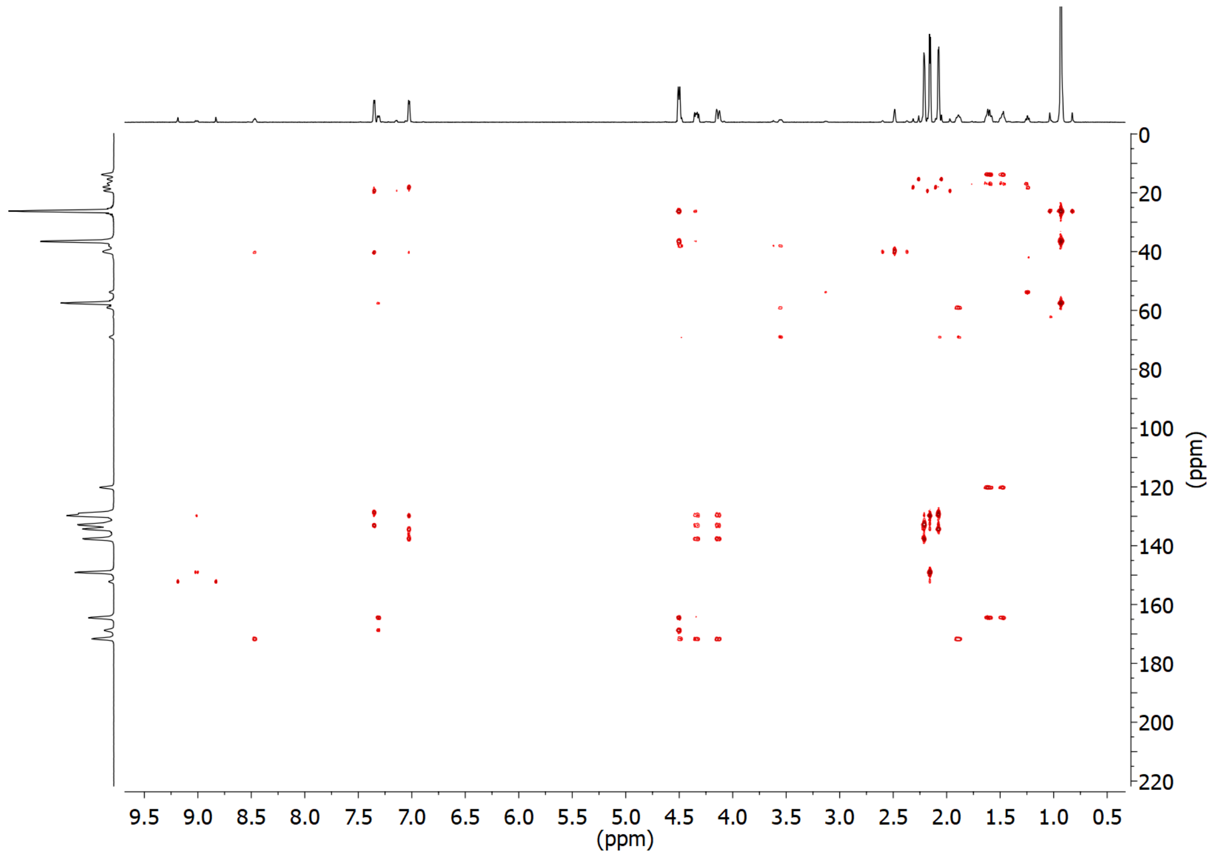

Further NMR Spectra of Compound 30

^{13}C DEPT-135

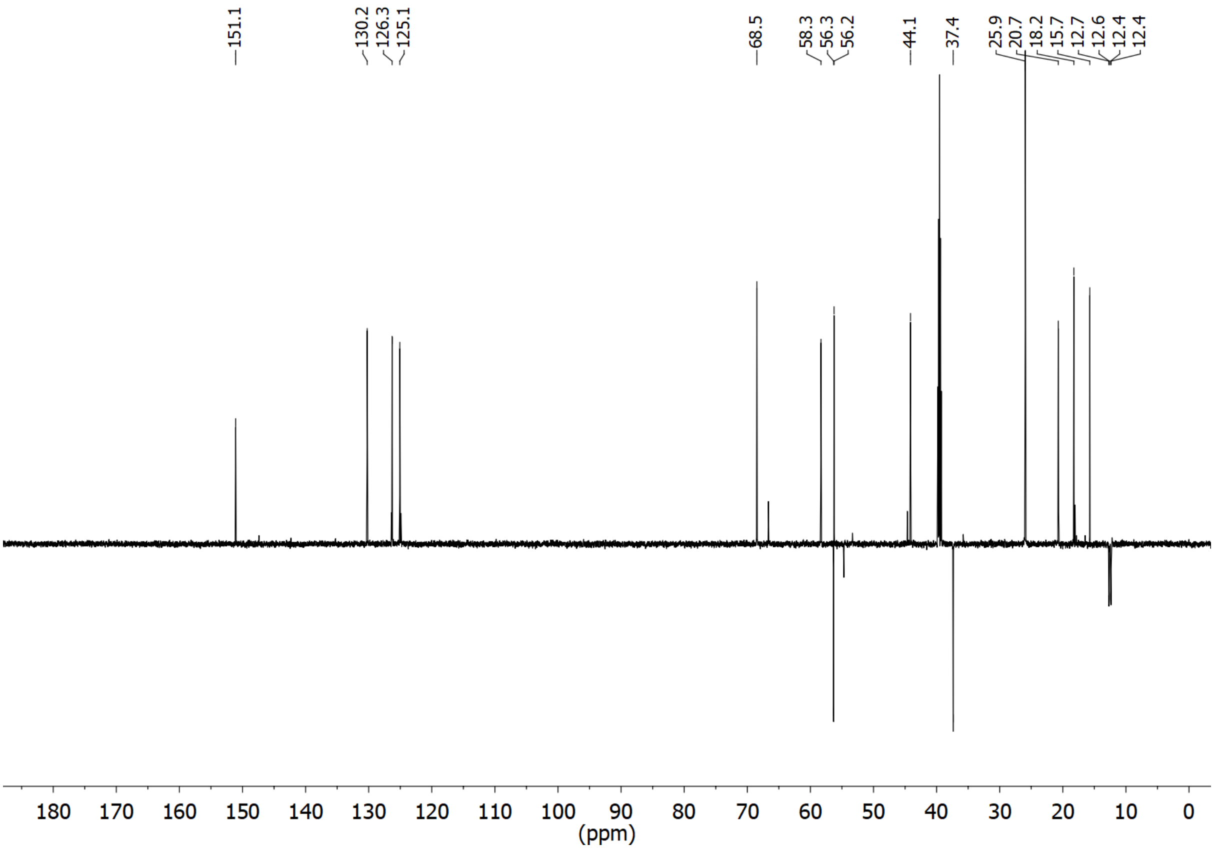

^{1}H-^{13}C HSQC spectrum

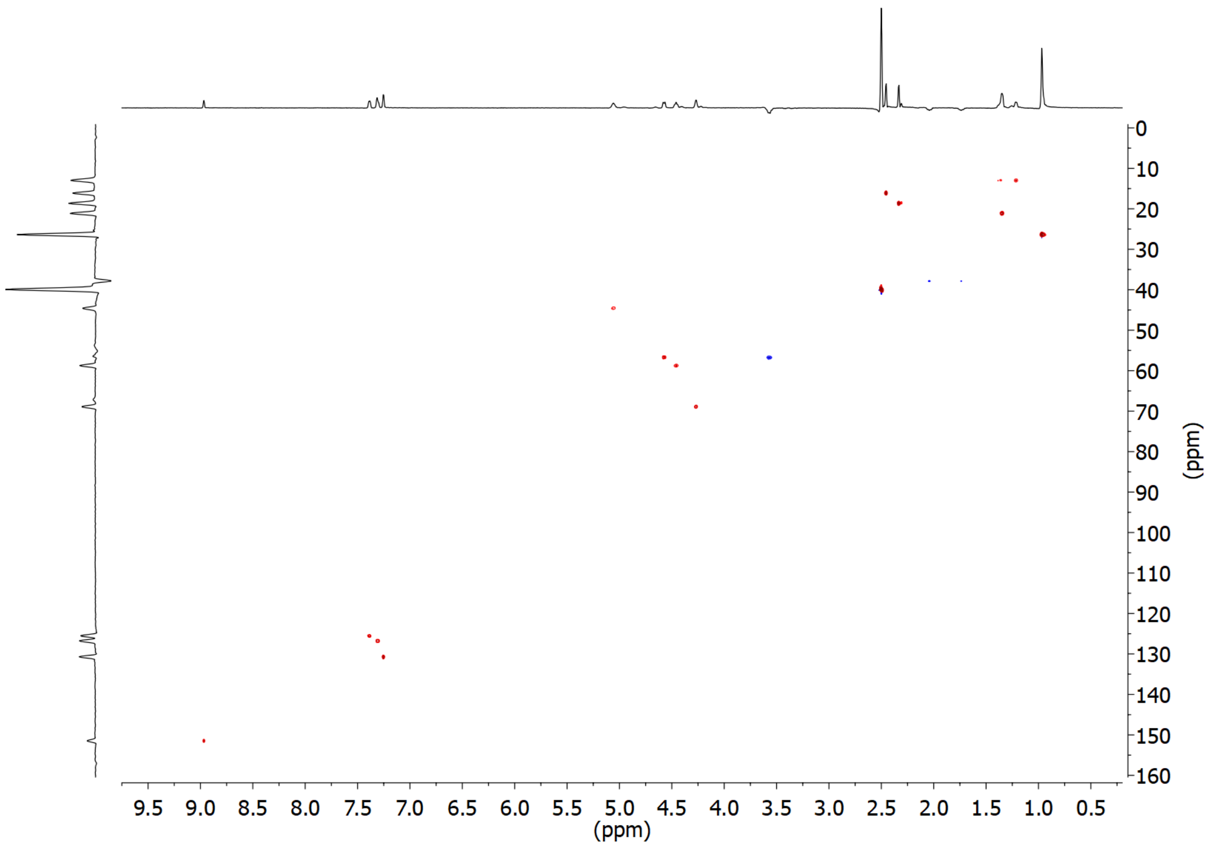

^{1}H-^{13}C HMBC spectrum

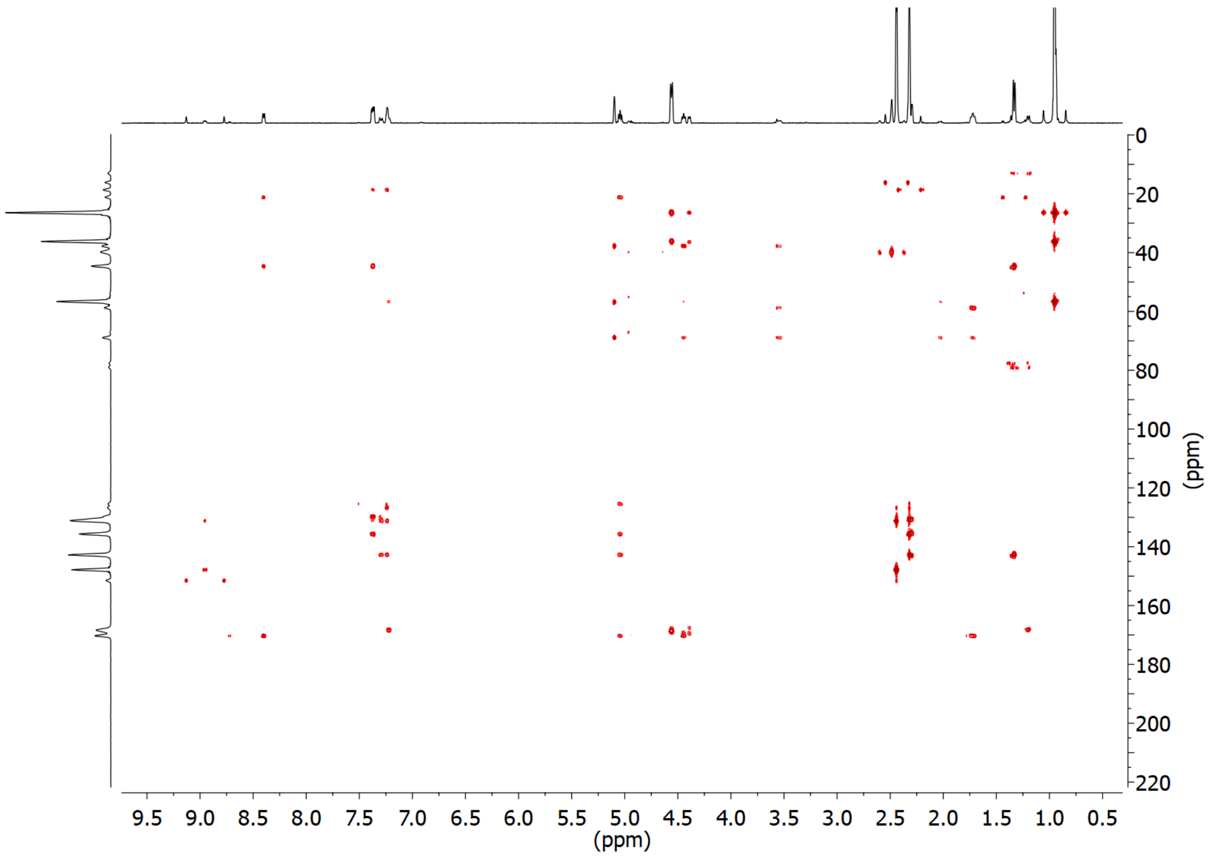

LC-MS Traces of Final Compounds

Compound **2**

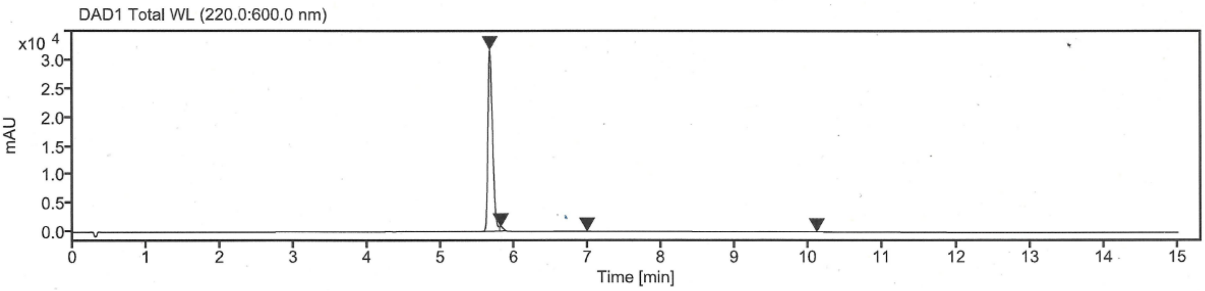

Signal: DAD1 Total WL (220.0:600.0 nm)

RT [min]	Peak MS Base Peak m/z	Area	Area%	Max Peak%	Height
5.677	536.400	138584.6639	96.9509	100.000	31824.908
5.820		2295.6403	1.6060	1.656	730.158
6.995		1535.9832	1.0745	1.108	80.029
10.119		526.8274	0.3686	0.380	48.944
	Sum	142943.1149			

purity = 97%

Compound **3**

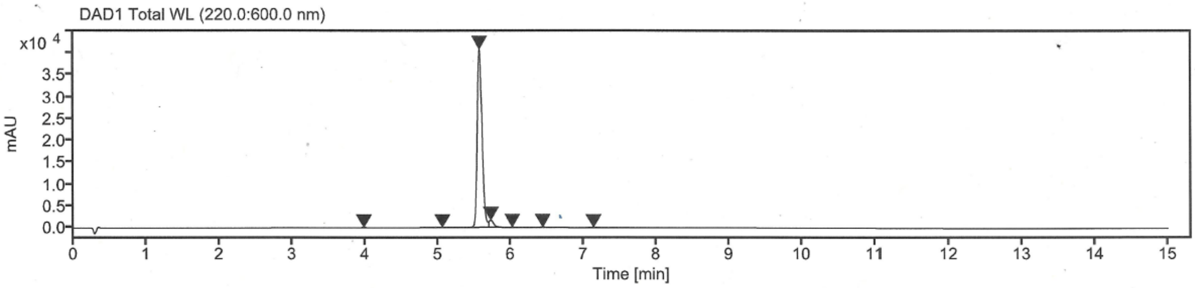

Signal: DAD1 Total WL (220.0:600.0 nm)

RT [min]	Peak MS Base Peak m/z	Area	Area%	Max Peak%	Height
3.988		176.0099	0.0905	0.094	32.252
5.066		310.9287	0.1598	0.167	25.241
5.576		186477.0282	95.8397	100.000	40656.491
5.729		6690.5045	3.4386	3.588	1621.728
6.026		174.8962	0.0899	0.094	39.854
6.444		446.0723	0.2293	0.239	68.811

purity = 96%

Compound **4**

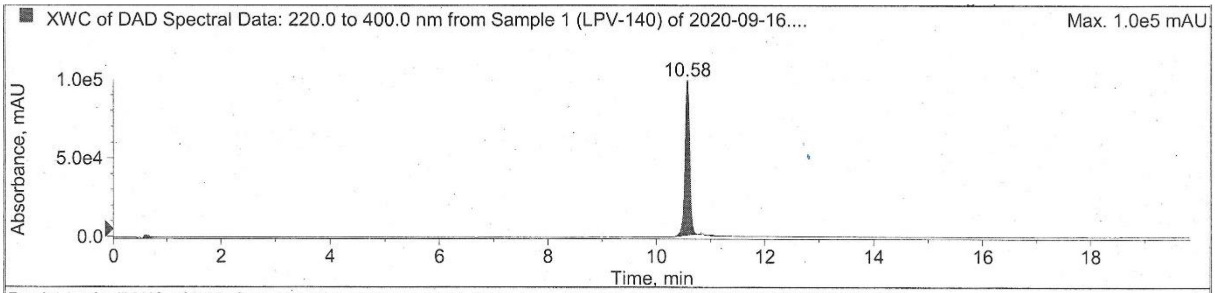

Peak List for "XWC of DAD Spectral Data: 220.0 to 400.0 nm from Sample 1 (LPV-140) of 2020-09-16.wiff"

	Time (min)	Area (mAU x min)	%Area	Height (mAU)	%Height	Width (min)	Baseline Type
1	0.6005	1.6903e4	2.5716	2217.1896	2.1581	0.3000	Base to Base
2	9.0280	595.8949	0.0907	107.7641	0.1049	0.2133	Base to Base
3	10.5769	6.3205e5	96.1608	9.8742e4	96.1097	0.4533	Base to Base
4	10.8426	5185.9035	0.7890	1123.8774	1.0939	0.1600	Base to Base
5	11.0139	1718.9395	0.2615	384.7005	0.3744	0.1667	Base to Base
6	11.4085	831.0986	0.1264	163.3526	0.1590	0.1800	Base to Base

purity = (96.1608 / 97.3449) × 100%

Compound **5**

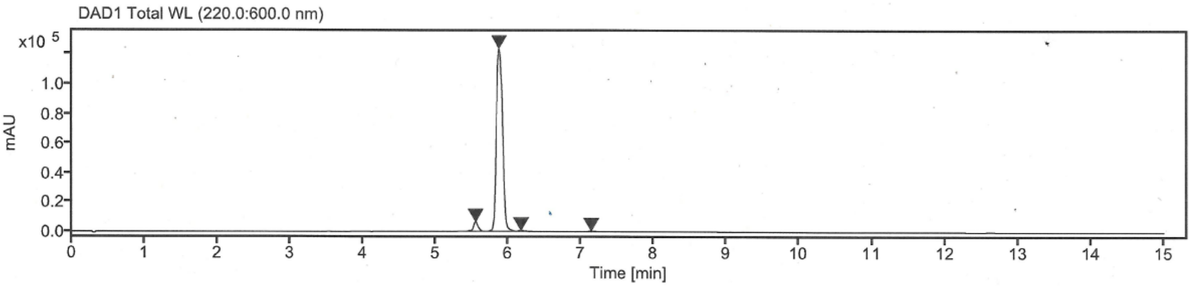

Signal: DAD1 Total WL (220.0:600.0 nm)

RT [min]	Peak MS Base Peak m/z	Area	Area%	Max Peak%	Height
5.557		29286.1973	3.6987	3.862	6494.562
5.880	556.300	758411.2994	95.7845	100.000	123944.991
6.183	558.400	2626.3175	0.3317	0.346	593.346
7.149		1465.3126	0.1851	0.193	336.348
	Sum	791789.1268			

purity = 96%

Compound **6**

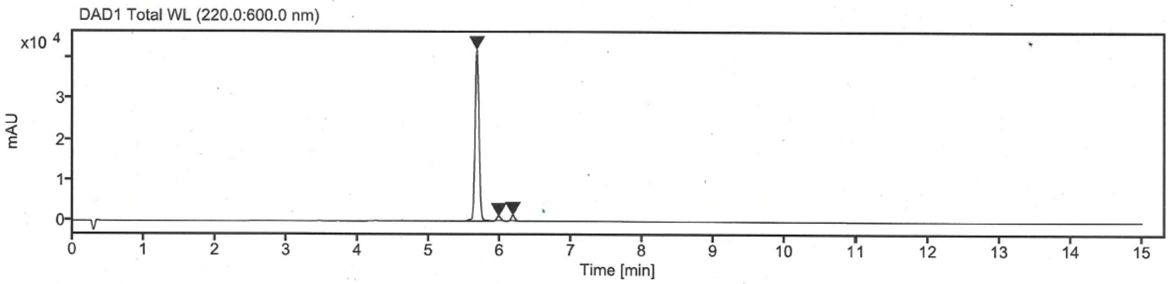

Signal:	DAD1 Total WL (220.0:600.0 nm)				
RT [min]	Peak MS Base Peak m/z	Area	Area%	Max Peak%	Height
5.682		160986.6255	95.0853	100.000	42109.943
5.989		3752.3575	2.2163	2.331	1108.525
6.188		4568.5856	2.6984	2.838	1379.317
	Sum	169307.5686			

purity = 95%

Compound **7**

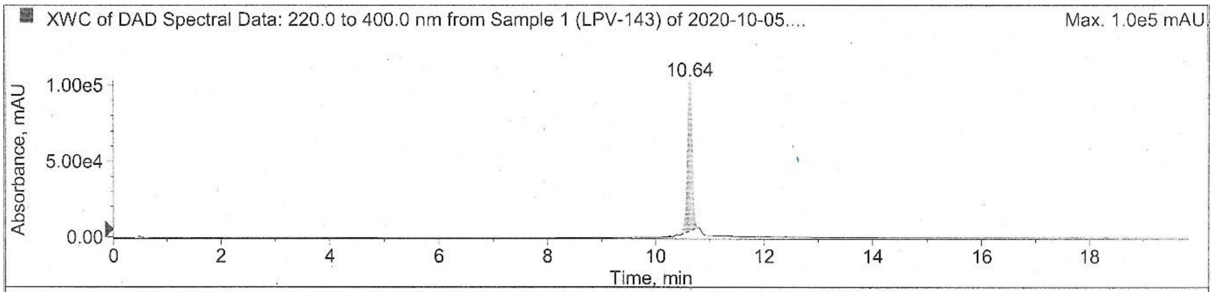

Peak List for "XWC of DAD Spectral Data: 220.0 to 400.0 nm from Sample 1 (LPV-143) of 2020-10-05.wiff"

	Time (min)	Area (mAU x min)	%Area	Height (mAU)	%Height	Width (min)	Baseline Type
1	0.3705	2991.4304	0.4553	493.7375	0.4809	0.2000	Base to Base
2	0.4773	1.0669e4	1.6239	1499.8512	1.4609	0.2533	Base to Base
3	8.9100	1372.3693	0.2089	221.9750	0.2162	0.2400	Base to Base
4	9.6134	1434.5455	0.2184	287.8176	0.2803	0.1867	Base to Base
5	10.2662	1488.7993	0.2266	314.0309	0.3059	0.2067	Base to Base
6	10.6378	6.3640e5	96.8649	9.9333e4	96.7523	0.3133	Base to Base
7	11.3769	2641.4143	0.4020	516.9066	0.5035	0.2000	Base to Base

purity = (96.8649 / 97.9208) × 100%

Compound **8**

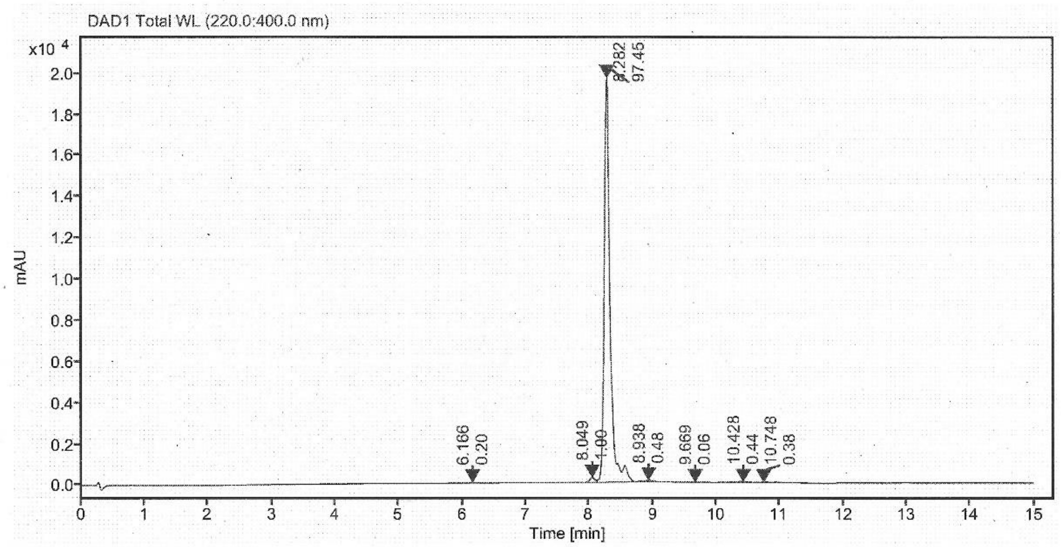

Signal: DAD1 Total WL (220.0:400.0 nm)

RT [min]	Peak MS Base Peak m/z	Type	Width [min]	Col0	Area	Height	Area%
6.166		MM m	0.4183		255.0543	43.8320	0.2025
8.049		MM m	0.2988		1256.5602	226.7534	0.9975
8.282	540.200	MM m	0.6175		122756.1530	19621.3007	97.4490
8.938		MM m	0.4183		599.1860	68.5664	0.4757
9.669		MM m	0.2191		70.2365	14.8888	0.0558
10.428		MM m	0.4781		559.1965	38.0160	0.4439
10.748		MM m	0.8765		473.2927	15.4997	0.3757
				Sum	125969.6793		

purity = 97%

Compound **9**

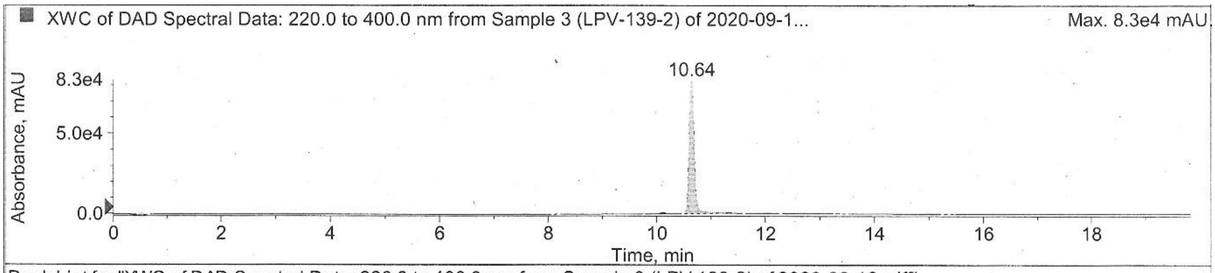

Peak List for "XWC of DAD Spectral Data: 220.0 to 400.0 nm from Sample 3 (LPV-139-2) of 2020-09-16.wiff"

	Time (min)	Area (mAU x min)	%Area	Height (mAU)	%Height	Width (min)	Baseline Type
1	0.4053	4712.8112	0.8285	801.8093	0.9146	0.1933	Base to Base
2	0.5269	1.0510e4	1.8478	1630.0800	1.8594	0.2333	Base to Base
3	7.5677	237.2684	0.0417	29.3388	0.0335	0.2600	Base to Base
4	8.1252	620.8835	0.1092	92.9436	0.1060	0.2867	Base to Base
5	9.0603	298.4947	0.0525	43.3571	0.0495	0.3133	Base to Base
6	9.7778	350.1989	0.0616	72.5227	0.0827	0.1733	Base to Base
7	10.1195	5667.4017	0.9963	940.5868	1.0729	0.3400	Base to Base
8	10.6428	5.4181e5	95.2516	8.3383e4	95.1151	0.8533	Base to Base
9	11.3332	593.1445	0.1043	136.1096	0.1553	0.1533	Base to Base
10	11.5686	125.7900	0.0221	29.0817	0.0332	0.1467	Base to Base
11	12.0228	1638.7393	0.2881	164.0048	0.1871	0.4133	Base to Base
12	12.3096	417.1911	0.0733	84.2370	0.0961	0.1667	Valley
13	12.3975	366.9807	0.0645	64.0124	0.0730	0.2000	Valley
14	13.2671	695.9483	0.1223	80.4433	0.0918	0.3933	Base to Base
15	14.9879	536.0018	0.0942	78.0872	0.0891	0.2733	Base to Base
16	15.2727	238.6024	0.0419	35.7201	0.0407	0.3133	Base to Base

purity = (95.2516 / 97.3237) × 100%

Compound **10**

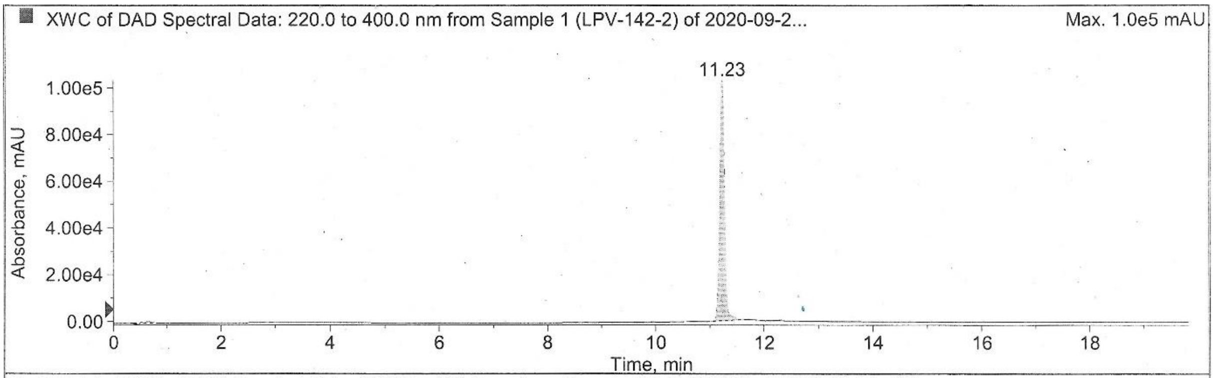

Peak List for "XWC of DAD Spectral Data: 220.0 to 400.0 nm from Sample 1 (LPV-142-2) of 2020-09-24.wiff"

	Time (min)	Area (mAU x min)	%Area	Height (mAU)	%Height	Width (min)	Baseline Type
1	0.4061	2967.7583	0.4506	639.5046	0.6027	0.1467	Base to Base
2	0.6443	6556.5489	0.9956	912.6413	0.8601	0.2800	Base to Base
3	9.9459	538.3397	0.0817	65.0391	0.0613	0.5133	Base to Base
4	10.5603	395.9705	0.0601	80.8596	0.0762	0.1467	Base to Base
5	10.7170	1386.7563	0.2106	200.6913	0.1891	0.2467	Base to Base
6	11.2258	6.4284e5	97.6136	1.0319e5	97.2513	0.7267	Base to Base
7	11.7127	443.1068	0.0673	127.7039	0.1204	0.1267	Base to Base
8	11.8610	638.1222	0.0969	128.9147	0.1215	0.1467	Base to Base
9	12.2098	320.4536	0.0487	110.1333	0.1038	0.1000	Base to Base
10	12.3080	2468.6523	0.3749	651.0488	0.6136	0.1533	Base to Base

purity = (97.6136 / 98.5538) × 100%

Compound **11**

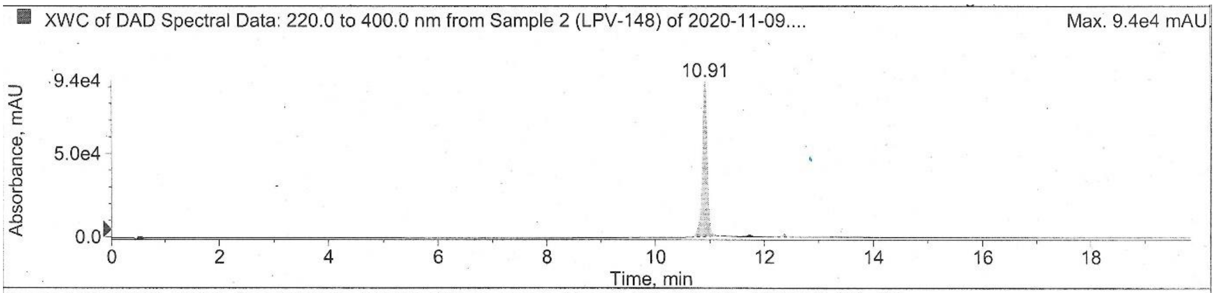

Peak List for "XWC of DAD Spectral Data: 220.0 to 400.0 nm from Sample 2 (LPV-148) of 2020-11-09.wiff"

	Time (min)	Area (mAU x min)	%Area	Height (mAU)	%Height	Width (min)	Baseline Type
1	0.5320	1.0334e4	1.7000	1471.1662	1.4924	0.2200	Base to Base
2	10.9121	5.7501e5	94.5930	9.3702e4	95.0551	0.5533	Base to Base
3	11.7376	6399.0062	1.0527	1242.2887	1.2602	0.3067	Base to Base
4	12.3700	9879.5527	1.6252	1878.6251	1.9057	0.4200	Base to Base
5	13.2657	6255.4771	1.0291	282.4279	0.2865	1.7000	Base to Base

purity = (94.5930 / 98.3) × 100%

Compound **12**

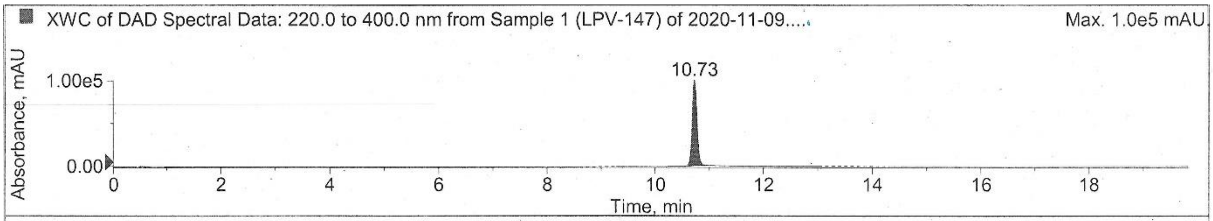

Peak List for "XWC of DAD Spectral Data: 220.0 to 400.0 nm from Sample 1 (LPV-147) of 2020-11-09.wiff"

	Time (min)	Area (mAU x min)	%Area	Height (mAU)	%Height	Width (min)	Baseline Type
1	0.5368	8992.4828	1.3003	1352.5046	1.3121	0.1867	Base to Base
2	8.7535	1.0090e4	1.4591	669.5371	0.6496	0.6667	Base to Base
3	10.7284	6.6538e5	96.2140	1.0068e5	97.6716	0.5133	Base to Base
4	13.2800	7099.8622	1.0266	377.9374	0.3667	1.2867	Base to Base

purity = (96.2140 / 98.6997) × 100%

Compound **13**

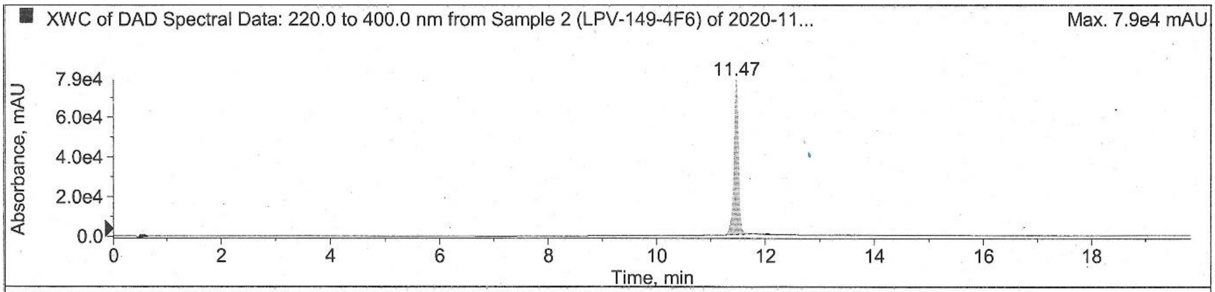

Peak List for "XWC of DAD Spectral Data: 220.0 to 400.0 nm from Sample 2 (LPV-149-4F6) of 2020-11-25.wiff"

	Time (min)	Area (mAU x min)	%Area	Height (mAU)	%Height	Width (min)	Baseline Type
1	0.5192	9888.9315	2.0576	1460.9420	1.8241	0.2200	Base to Base
2	11.4717	4.6868e5	97.5197	7.8243e4	97.6931	0.5467	Base to Base
3	12.0401	2031.6452	0.4227	386.6594	0.4828	0.2267	Base to Base

purity = (97.5197 / 97.9424) × 100%

Compound **14**

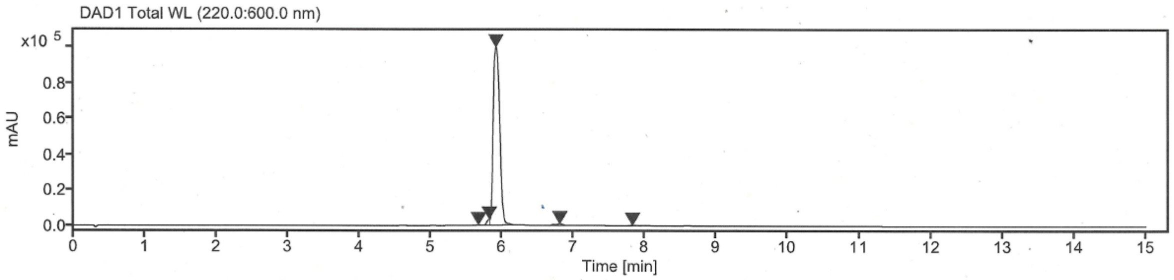

Signal:	DAD1 Total WL (220.0:600.0 nm)				
RT [min]	Peak MS Base Peak m/z	Area	Area%	Max Peak%	Height
5.676		1550.1939	0.2391	0.246	274.648
5.828	552.400	8979.2950	1.3852	1.426	3336.845
5.924		629745.7626	97.1514	100.000	100260.774
6.816		7432.5230	1.1466	1.180	1022.135
7.838		503.0438	0.0776	0.080	202.564
	Sum	648210.8182			

purity = 97%

Compound **15**

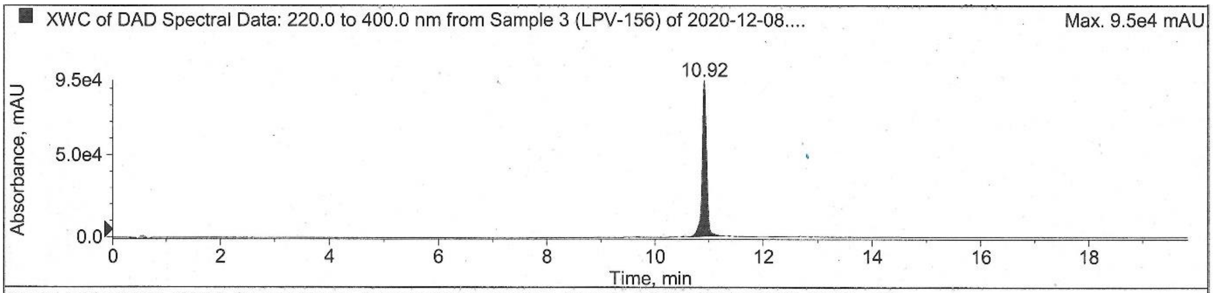

Peak List for "XWC of DAD Spectral Data: 220.0 to 400.0 nm from Sample 3 (LPV-156) of 2020-12-08.wiff"

	Time (min)	Area (mAU x min)	%Area	Height (mAU)	%Height	Width (min)	Baseline Type
1	0.4102	4043.9654	0.6805	734.7209	0.7395	0.1800	Base to Base
2	0.5282	1.2638e4	2.1268	1731.0293	1.7423	0.2400	Base to Base
3	9.6695	1208.2197	0.2033	180.3551	0.1815	0.2667	Base to Base
4	10.5492	3910.2986	0.6581	787.9631	0.7931	0.2733	Base to Base
5	10.9150	5.6629e5	95.3000	9.4734e4	95.3490	0.5933	Base to Base
6	11.3015	2386.6311	0.4016	355.5633	0.3579	0.3067	Base to Base
7	12.2279	1185.6471	0.1995	222.4589	0.2239	0.2667	Base to Base
8	12.6834	2555.8364	0.4301	608.9085	0.6129	0.3600	Base to Base

purity = (95.3000 / 97.1927) × 100%

Compound **16**

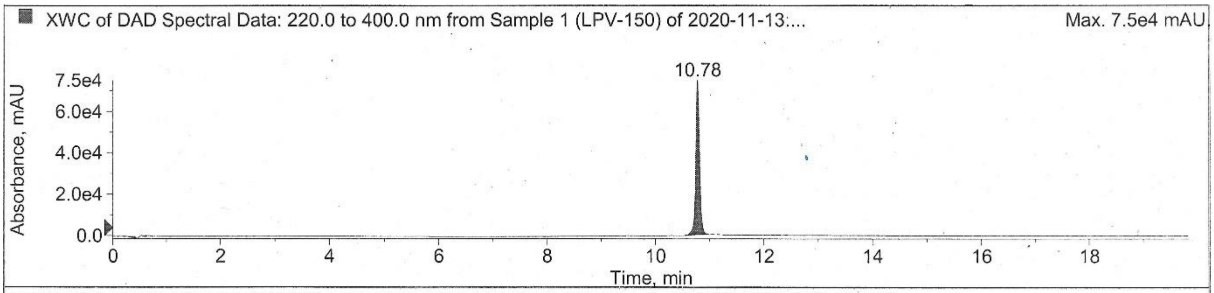

Peak List for "XWC of DAD Spectral Data: 220.0 to 400.0 nm from Sample 1 (LPV-150) of 2020-11-13.wiff"

	Time (min)	Area (mAU x min)	%Area	Height (mAU)	%Height	Width (min)	Baseline Type
1	0.3969	5074.0424	1.2366	826.9147	1.0718	0.1867	Base to Base
2	0.6542	5722.1591	1.3945	817.3904	1.0594	0.2667	Base to Base
3	10.7839	3.9767e5	96.9142	7.5097e4	97.3355	0.4800	Base to Base
4	11.0658	835.1354	0.2035	196.2531	0.2544	0.2133	Base to Base
5	12.2127	1030.7231	0.2512	215.1457	0.2789	0.2067	Base to Base

purity = (96.9142 / 97.3789) × 100%

Compound **17**

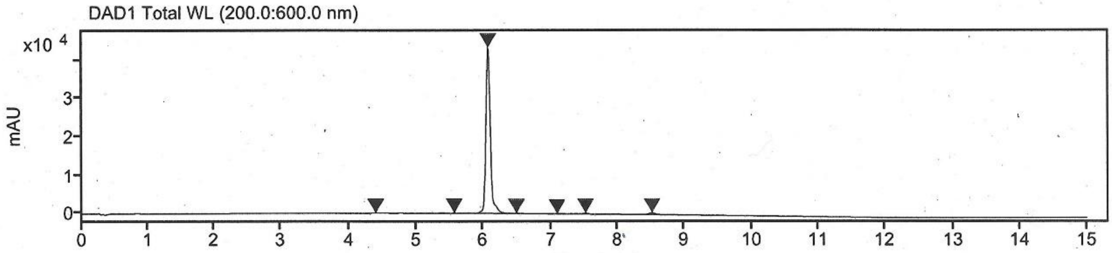

Signal: DAD1 Total WL (200.0:600.0 nm)

RT [min]	Peak MS Base Peak m/z	Area	Area%	Max Peak%	Height
4.396		226.3860	0.1095	0.112	44.039
5.563		745.5780	0.3605	0.369	62.541
6.068		201848.9761	97.6076	100.000	43149.784
6.486		224.8447	0.1087	0.111	43.962
7.093		102.1343	0.0494	0.051	33.775
7.520		1095.7713	0.5299	0.543	239.790
8.513		2552.7072	1.2344	1.265	464.509
	Sum	206796.3975			

purity = 98%

Compound **18**

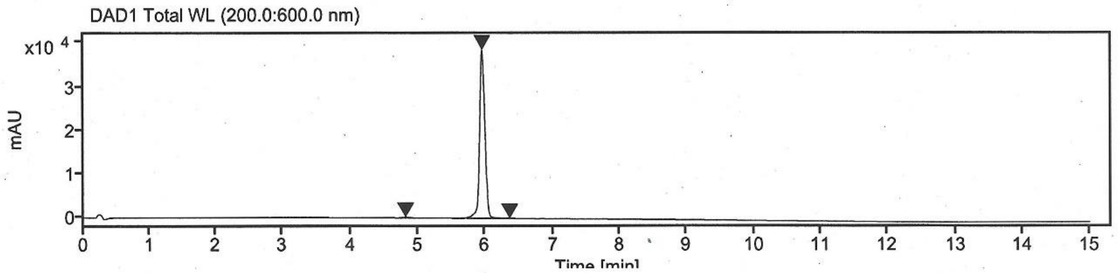

Signal: DAD1 Total WL (200.0:600.0 nm)

RT [min]	Name	Area	Area%	Max Peak%	Height	Type
4.816		769.4192	0.3570	0.358	149.907	MM m
5.956		214708.5224	99.6302	100.000	38526.762	MM m
6.359		27.4521	0.0127	0.013	16.994	MM m
	Sum	215505.3936				

purity = 100%

Compound **19**

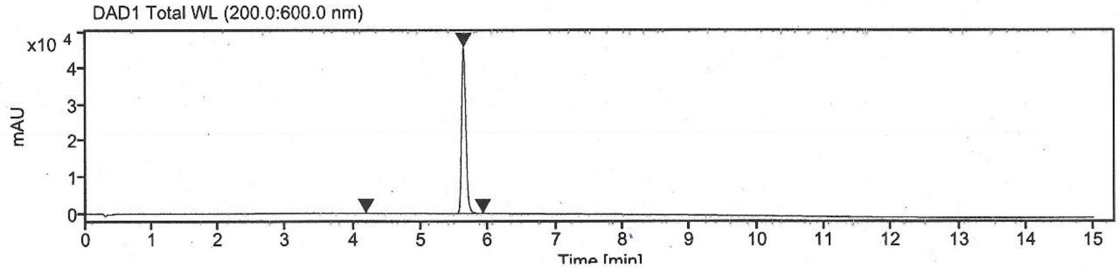

Signal: DAD1 Total WL (200.0:600.0 nm)

RT [min]	Peak MS Base Peak m/z	Area	Area%	Max Peak%	Height
4.183		247.6575	0.1268	0.127	54.610
5.632	558.500	194878.7949	99.7613	100.000	46000.590
5.912		218.5805	0.1119	0.112	69.433
	Sum	195345.0329			

purity = 100%

Compound **20**

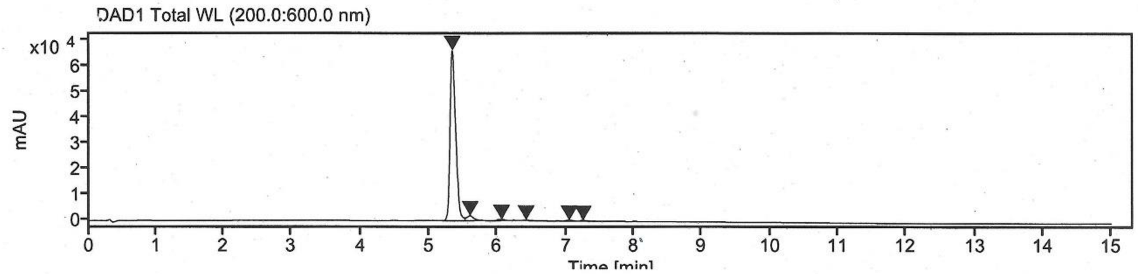

Signal:	DAD1 Total WL (200.0:600.0 nm)				
RT [min]	Peak MS Base Peak m/z	Area	Area%	Max Peak%	Height
5.342		384771.9121	94.5330	100.000	66192.190
5.598		13567.5275	3.3333	3.526	1861.586
6.056		3248.8546	0.7982	0.844	455.978
6.416		1625.2204	0.3993	0.422	242.923
7.046		1904.7760	0.4680	0.495	325.869
7.253		1905.7229	0.4682	0.495	283.359
	Sum	407024.0135			

purity = 95%

Compound **21**

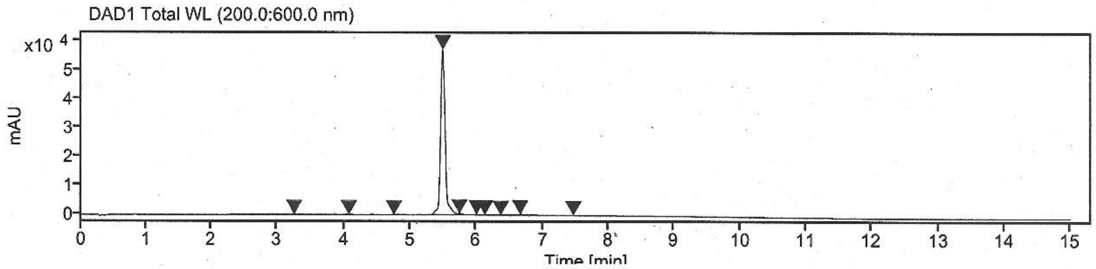

Signal:	DAD1 Total WL (200.0:600.0 nm)				
RT [min]	Peak MS Base Peak m/z	Area	Area%	Max Peak%	Height
3.250		201.2085	0.0739	0.075	42.175
4.073		260.2270	0.0956	0.097	55.153
4.751		99.2523	0.0364	0.037	30.672
5.490		268300.0752	98.5283	100.000	56735.841
5.740		1340.4138	0.4922	0.500	374.359
6.000		427.0610	0.1568	0.159	111.220
6.125		81.7147	0.0300	0.030	18.270
6.364		244.4375	0.0898	0.091	32.222
6.658		930.5620	0.3417	0.347	196.404

purity = 99%

Compound **22**

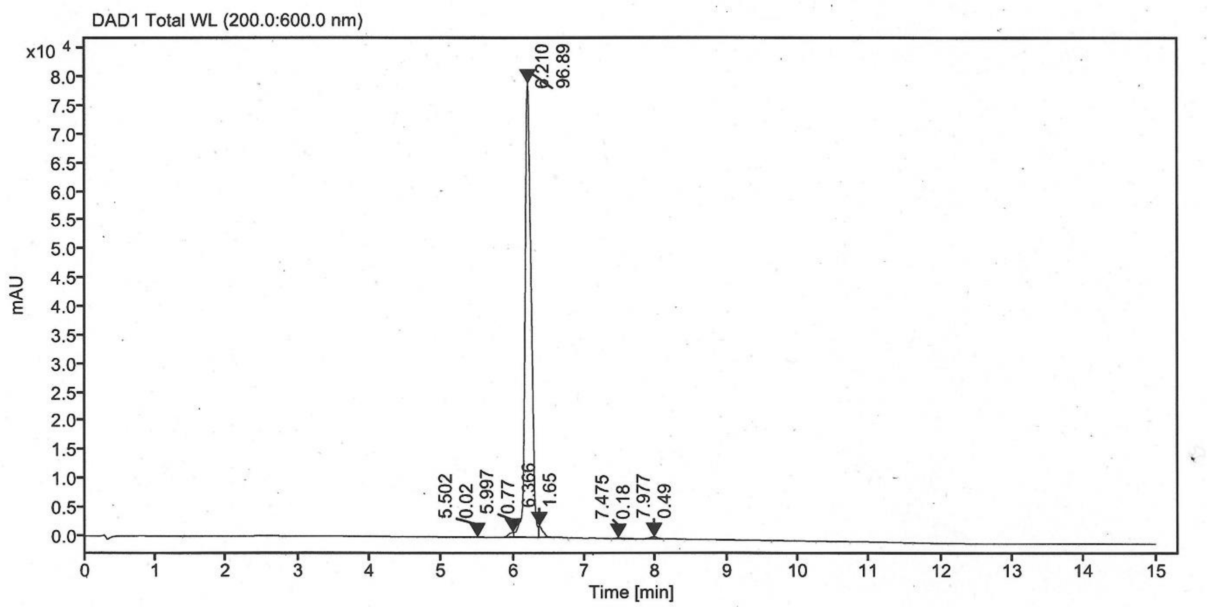

Signal: DAD1 Total WL (200.0:600.0 nm)

RT [min]	Peak MS Base Peak m/z	Type	Width [min]	Col0	Area	Height	Area%
5.502		MM m	0.1361		111.8480	22.1501	0.0233
5.997		MM m	0.1408		3689.3814	789.3578	0.7677
6.210	574.400	MM m	0.3477		465621.3614	79091.5362	96.8880
6.366		MM m	0.1663		7924.4086	1952.4803	1.6489
7.475		MM m	0.3924		875.3600	119.4012	0.1821
7.977		MM m	0.4858		2354.5182	325.9724	0.4899
				Sum	480576.8776		

purity = 97%

Compound **23**

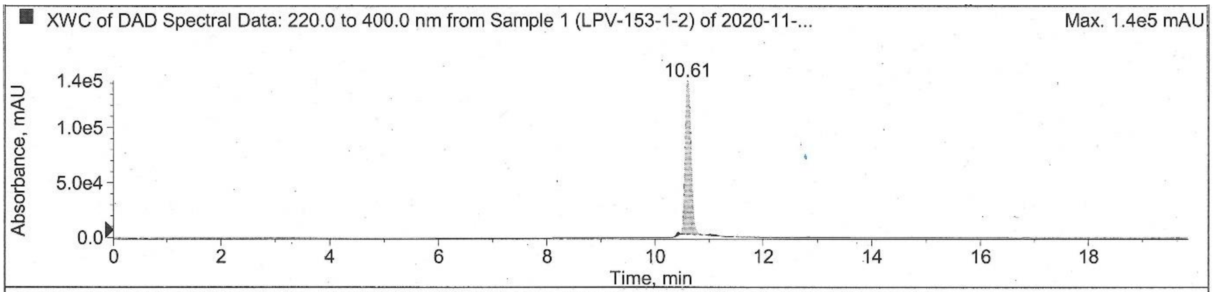

Peak List for "XWC of DAD Spectral Data: 220.0 to 400.0 nm from Sample 1 (LPV-153-1-2) of 2020-11-30.wiff"

	Time (min)	Area (mAU x min)	%Area	Height (mAU)	%Height	Width (min)	Baseline Type
1	0.4071	5048.7501	0.4671	780.3473	0.5344	0.2000	Base to Base
2	0.5448	1.0347e4	0.9574	1369.3865	0.9377	0.2333	Base to Base
3	10.4313	9229.6953	0.8540	2654.9029	1.8180	0.1133	Base to Base
4	10.6098	1.0417e6	96.3870	1.3946e5	95.5016	0.4600	Base to Base
5	11.0188	1.0247e4	0.9481	997.0890	0.6828	0.3200	Base to Base
6	11.4545	1611.8757	0.1491	354.7437	0.2429	0.1600	Base to Base
7	12.8327	2564.8255	0.2373	412.7495	0.2826	0.4600	Base to Base

purity = (96.3870 / 98.5755) × 100%

Compound **24**

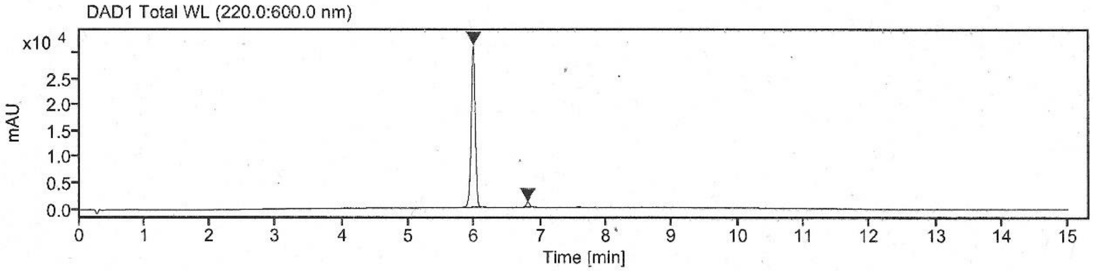

Signal: DAD1 Total WL (220.0:600.0 nm)

RT [min]	Peak MS Base Peak m/z	Area	Area%	Max Peak%	Height
5.982	552.400	132103.3165	97.4531	100.000	30964.213
6.804		3452.5304	2.5469	2.614	931.063
	Sum	135555.8468			

purity = 97%

Compound **25**

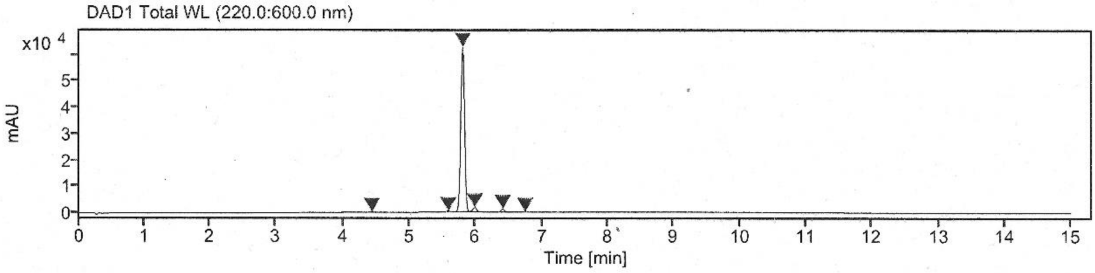

Signal: DAD1 Total WL (220.0:600.0 nm)

RT [min]	Peak MS Base Peak m/z	Area	Area%	Max Peak%	Height
4.433		553.8883	0.2149	0.226	74.506
5.586		1355.4533	0.5260	0.553	275.008
5.804		245056.5965	95.0885	100.000	62583.658
5.985		6153.9519	2.3879	2.511	1514.988
6.406		4109.6560	1.5947	1.677	1108.633
6.746		484.7860	0.1881	0.198	63.433
	Sum	257714.3320			

purity = 95%

Compound **26**

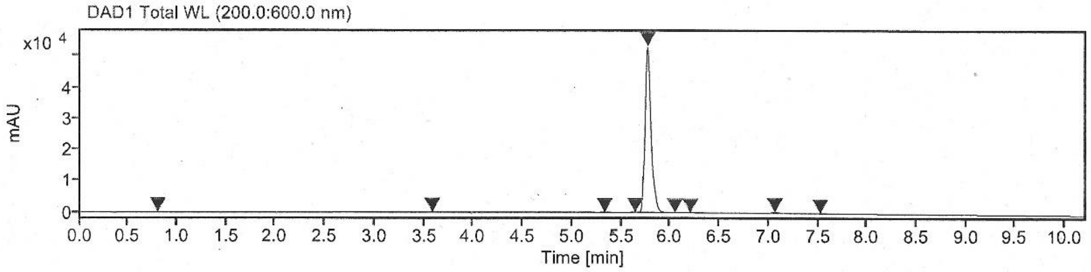

Signal: DAD1 Total WL (200.0:600.0 nm)

RT [min]	Peak MS Base Peak m/z	Area	Area%	Max Peak%	Height
0.805		137.5086	0.0587	0.060	31.118
3.583		427.4896	0.1825	0.185	64.506
5.333		212.3401	0.0907	0.092	43.466
5.643		59.8057	0.0255	0.026	37.178
5.769	529.300	230762.2199	98.5146	100.000	53423.339
6.046		67.1080	0.0286	0.029	21.585
6.204		460.2248	0.1965	0.199	51.014
7.063		1685.4657	0.7195	0.730	364.515
7.524		429.3840	0.1833	0.186	68.158
	Sum	234241.5465			

purity = 99%

Compound **27**

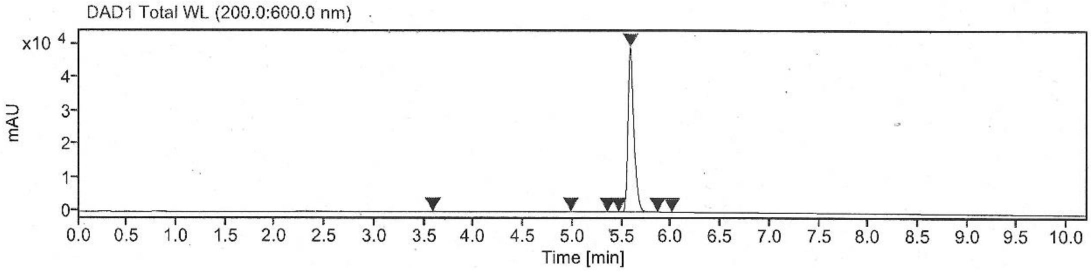

Signal: DAD1 Total WL (200.0:600.0 nm)

RT [min]	Peak MS Base Peak m/z	Area	Area%	Max Peak%	Height
3.585		154.5091	0.0745	0.075	40.010
4.983		330.7712	0.1594	0.160	65.579
5.352		6.4436	0.0031	0.003	3.051
5.468		104.9723	0.0506	0.051	40.346
5.588		206825.2896	99.6677	100.000	49272.991
5.860		48.6678	0.0235	0.024	19.045
6.007		44.1265	0.0213	0.021	13.803
	Sum	207514.7800			

purity = 100%

Compound **28**

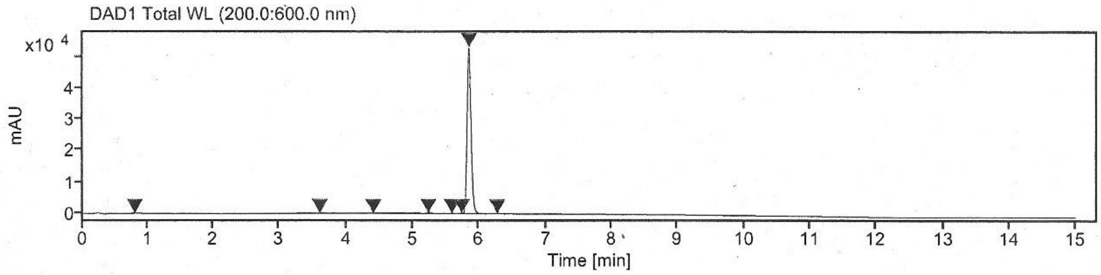

Signal:	DAD1 Total WL (200.0:600.0 nm)				
RT [min]	Peak MS Base Peak m/z	Area	Area%	Max Peak%	Height
0.808		164.4316	0.0761	0.077	35.325
3.604		178.7683	0.0827	0.083	53.196
4.407		191.8365	0.0888	0.089	51.750
5.232		197.0585	0.0912	0.092	52.004
5.586		128.1022	0.0593	0.060	37.442
5.733		307.3874	0.1422	0.143	107.147
5.845	549.400	214869.1108	99.4202	100.000	52991.103
6.268		85.4838	0.0396	0.040	30.738
	Sum	216122.1791			

purity = 99%

Compound **29**

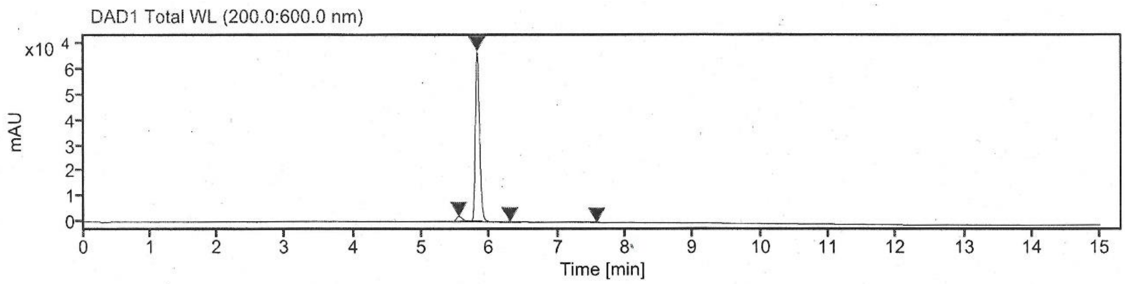

Signal:	DAD1 Total WL (200.0:600.0 nm)				
RT [min]	Peak MS Base Peak m/z	Area	Area%	Max Peak%	Height
5.542		12358.8998	4.1450	4.342	2033.382
5.816		284645.3134	95.4650	100.000	67014.266
6.292		760.4387	0.2550	0.267	105.758
7.574		402.5648	0.1350	0.141	100.157
	Sum	298167.2168			

purity = 95%

Compound **30**

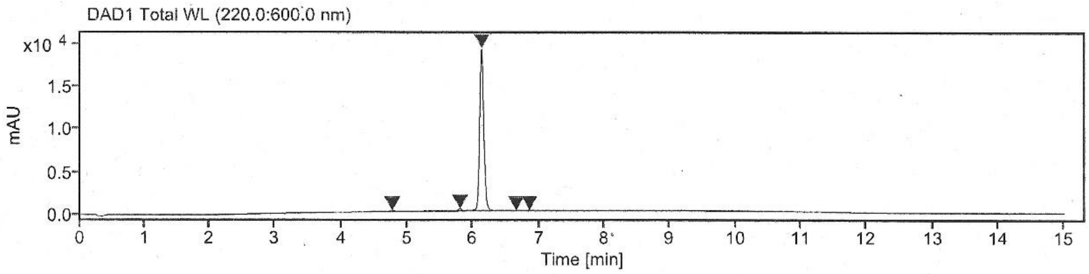

Signal: DAD1 Total WL (220.0:600.0 nm)

RT [min]	Peak MS Base Peak m/z	Area	Area%	Max Peak%	Height
4.769		469.2136	0.5538	0.564	115.625
5.799		875.0732	1.0329	1.052	238.811
6.128	545.400	83170.7947	98.1728	100.000	18989.677
6.651		84.4037	0.0996	0.101	12.419
6.852		119.2649	0.1408	0.143	30.602
	Sum	84718.7501			

purity = 98%

Compound **31**

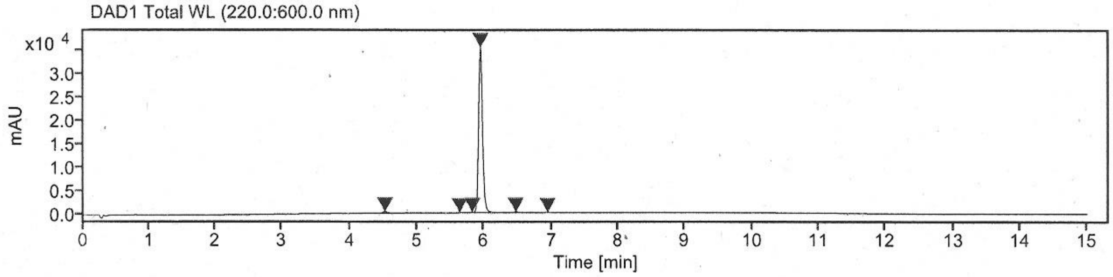

Signal: DAD1 Total WL (220.0:600.0 nm)

RT [min]	Peak MS Base Peak m/z	Area	Area%	Max Peak%	Height
4.522		1344.4522	0.9067	0.923	326.693
5.632		118.3895	0.0798	0.081	34.277
5.818		409.9148	0.2765	0.281	98.643
5.943		145692.2478	98.2569	100.000	35167.645
6.466		598.4529	0.4036	0.411	123.800
6.941		113.3862	0.0765	0.078	21.261
	Sum	148276.8434			

purity = 98%

Compound **32**

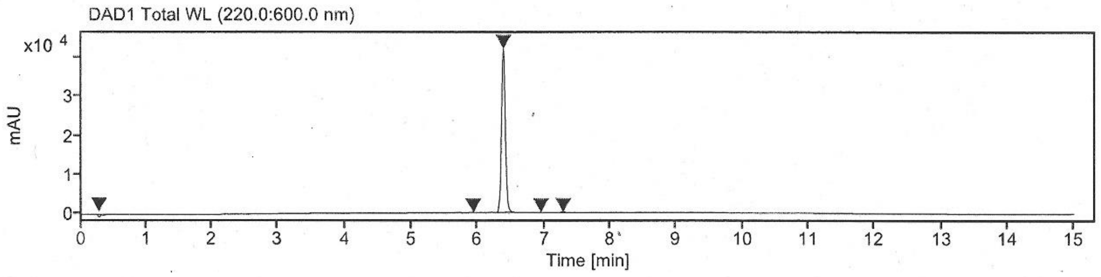

Signal: DAD1 Total WL (220.0:600.0 nm)

RT [min]	Peak MS Base Peak m/z	Area	Area%	Max Peak%	Height
0.281		2603.1494	1.5007	1.528	673.194
5.929		141.8345	0.0818	0.083	37.290
6.384		170376.2702	98.2198	100.000	41615.328
6.950		29.7090	0.0171	0.017	8.053
7.286		313.2743	0.1806	0.184	90.714
	Sum	173464.2372			

purity = 98%

Compound **33**

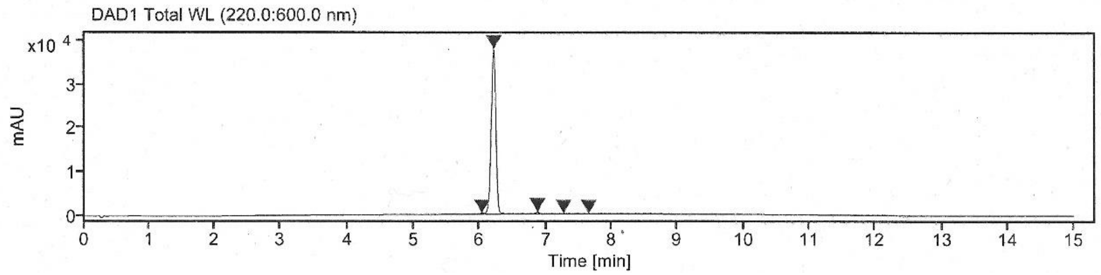

Signal: DAD1 Total WL (220.0:600.0 nm)

RT [min]	Peak MS Base Peak m/z	Area	Area%	Max Peak%	Height
6.029		423.1174	0.2276	0.231	152.376
6.209	577.400	183498.9180	98.6949	100.000	37584.234
6.871	559.400	1846.1896	0.9930	1.006	455.576
7.265		83.4034	0.0449	0.045	9.550
7.652		73.8505	0.0397	0.040	14.584
	Sum	185925.4790			

purity = 99%

Compound **34**

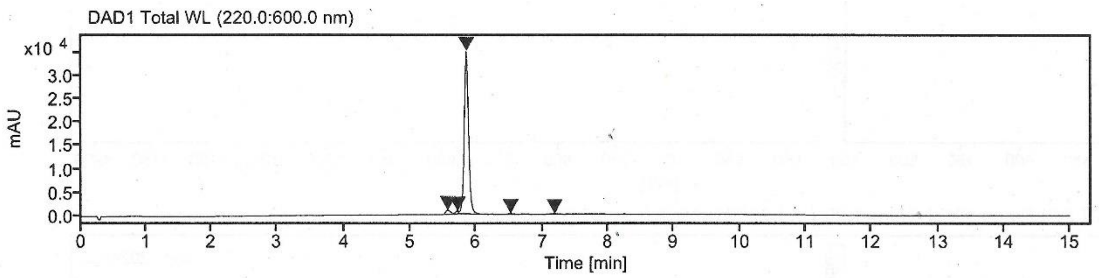

Signal: DAD1 Total WL (220.0:600.0 nm)

RT [min]	Peak MS Base Peak m/z	Area	Area%	Max Peak%	Height
5.566		4356.9262	2.6977	2.825	831.450
5.715		1856.0350	1.1492	1.203	560.423
5.850		154220.8514	95.4892	100.000	34745.931
6.516		622.9425	0.3857	0.404	148.924
7.184		449.2928	0.2782	0.291	69.778
	Sum	161506.0479			

purity = 95%

Compound **35**

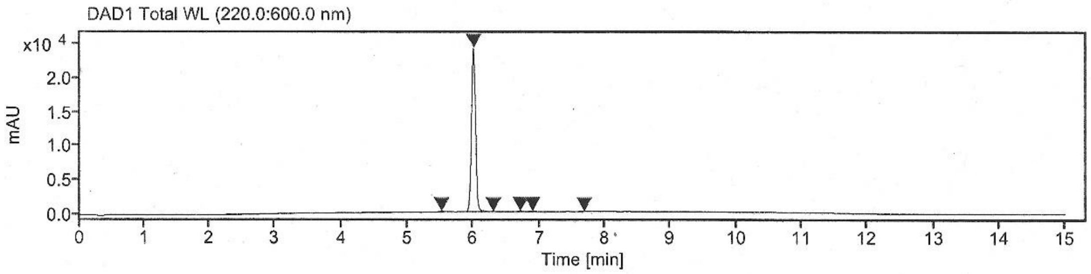

Signal: DAD1 Total WL (220.0:600.0 nm)

RT [min]	Peak MS Base Peak m/z	Area	Area%	Max Peak%	Height
5.515		625.6175	0.6096	0.617	79.458
6.000	566.400	101387.1888	98.7908	100.000	23936.322
6.298		25.2446	0.0246	0.025	16.445
6.709		263.0018	0.2563	0.259	68.614
6.895		256.8122	0.2502	0.253	50.021
7.686		70.2927	0.0685	0.069	15.176
	Sum	102628.1576			

purity = 99%

Compound **36**

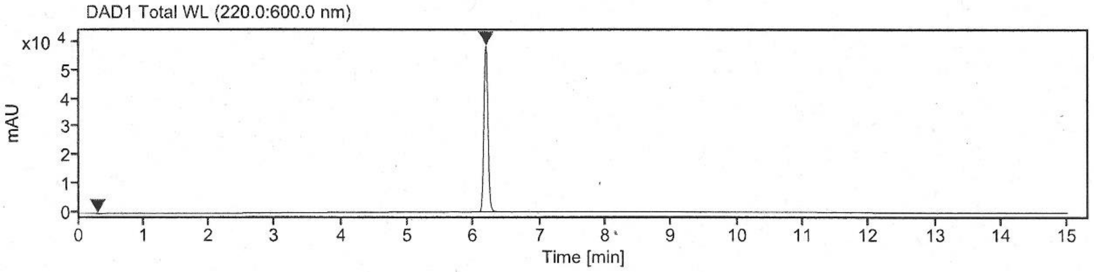

Signal: DAD1 Total WL (220.0:600.0 nm)

RT [min]	Peak MS Base Peak m/z	Area	Area%	Max Peak%	Height
0.298		566.9431	0.2372	0.238	161.200
6.183		238424.0197	99.7628	100.000	58028.555
	Sum	238990.9628			

purity = 100%

Compound **37**

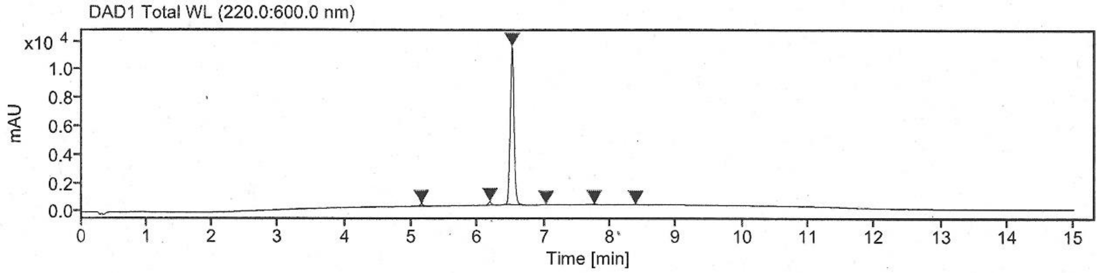

Signal: DAD1 Total WL (220.0:600.0 nm)

RT [min]	Peak MS Base Peak m/z	Area	Area%	Max Peak%	Height
5.144		505.7938	1.1024	1.146	145.656
6.179		847.1941	1.8464	1.920	226.645
6.512		44127.4040	96.1734	100.000	11115.265
7.024		41.1874	0.0898	0.093	8.557
7.757		282.1872	0.6150	0.639	45.981
8.383		79.4119	0.1731	0.180	20.680
	Sum	45883.1785			

purity = 96%

Crystallographic Data for Co-crystal Structures of VCB in Complex with Ligands 30, 33, and 37

	**Compound 33**	**Compound 30**	**Compound 37**
**Wavelength**	0.9999	0.9999	0.9999
**Resolution range**	64.48 - 2.38 (2.465 - 2.38)	57.32 - 2.62 (2.714 - 2.62)	90.05 - 2.85 (2.952 - 2.85)
**Space group**	P 41 21 2	P 41 21 2	P 41 21 2
**Unit cell**	92.688 92.688 359.622 90 90 90	93.284 93.284 363.262 90 90 90	92.971 92.971 362.047 90 90 90
**Total reflections**	413146 (40987)	739187 (62168)	981408 (95612)
**Unique reflections**	64113 (6121)	49525 (4843)	38322 (3705)
**Multiplicity**	6.4 (6.6)	14.9 (12.8)	25.6 (25.7)
**Completeness (%)**	99.46 (98.06)	99.74 (99.71)	99.66 (99.60)
**Mean I/sigma(I)**	6.16 (1.01)	7.03 (1.05)	5.16 (1.18)
**Wilson B-factor**	42.94	54.57	46.69
**R-merge**	0.2541 (2.517)	0.2948 (2.643)	0.8607 (5.587)
**CC1/2**	0.997 (0.338)	0.996 (0.429)	0.989 (0.438)
**Reflections used in refinement**	63803 (6123)	49411 (4839)	38198 (3700)
**Reflections used for R-free**	3224 (280)	2424 (239)	1917 (191)
**R-work**	0.2273 (0.3068)	0.2454 (0.3516)	0.2490 (0.3390)
**R-free**	0.2734 (0.3581)	0.2889 (0.3728)	0.2955 (0.3880)
**macromolecules**	10345	10319	10427
**ligands**	160	152	160
**solvent**	232	96	1
**Protein residues**	1336	1334	1332
**RMS(bonds)**	0.165	0.159	0.003
**RMS(angles)**	1.62	1.41	0.74
**Ramachandran favored (%)**	97.79	97.47	97.23
**Ramachandran allowed (%)**	2.13	2.37	2.61
**Ramachandran outliers (%)**	0.08	0.16	0.16
**Rotamer outliers (%)**	0.28	0.18	1.34
**Clashscore**	6.89	6.96	6.86
**Average B-factor**	47.84	54.31	47.13
**macromolecules**	48.02	54.45	47.32
**ligands**	41.05	49.22	34.98
**solvent**	44.36	47.26	23.44
**PDB Code**	8CQL	8CQK	8CQE

Statistics for the highest-resolution shell are shown in parentheses.
